# Supplementary material for: Building Resident Quality Improvement Knowledge and Engagement Through a Longitudinal, Mentored, and Experiential Learning-Based Quality Improvement Curriculum
Source: MedEdPORTAL. 2023 Apr 18;19:11310. doi: 10.15766/mep_2374-8265.11310 (PMC10110773; doi:10.15766/mep_2374-8265.11310)
Supplement: Supplementary file 1 — Session 1 Slides.pptxSession 1 Workbook.pptxSession 2 Slides.pptxSession 2 Workbook.pptxSession 3 Slides.pptxSession 4 Work-in-Progress Presentation Template.pptxSession 5 Slides.pptxQI Charter Template.docxFaculty Milestones.docxFaculty Guide.docxResident Survey.docx [file mep_2374-8265.11310-s001.zip › F. Session 4 Work-in-Progress Presentation Template.pptx]

## Slide 1
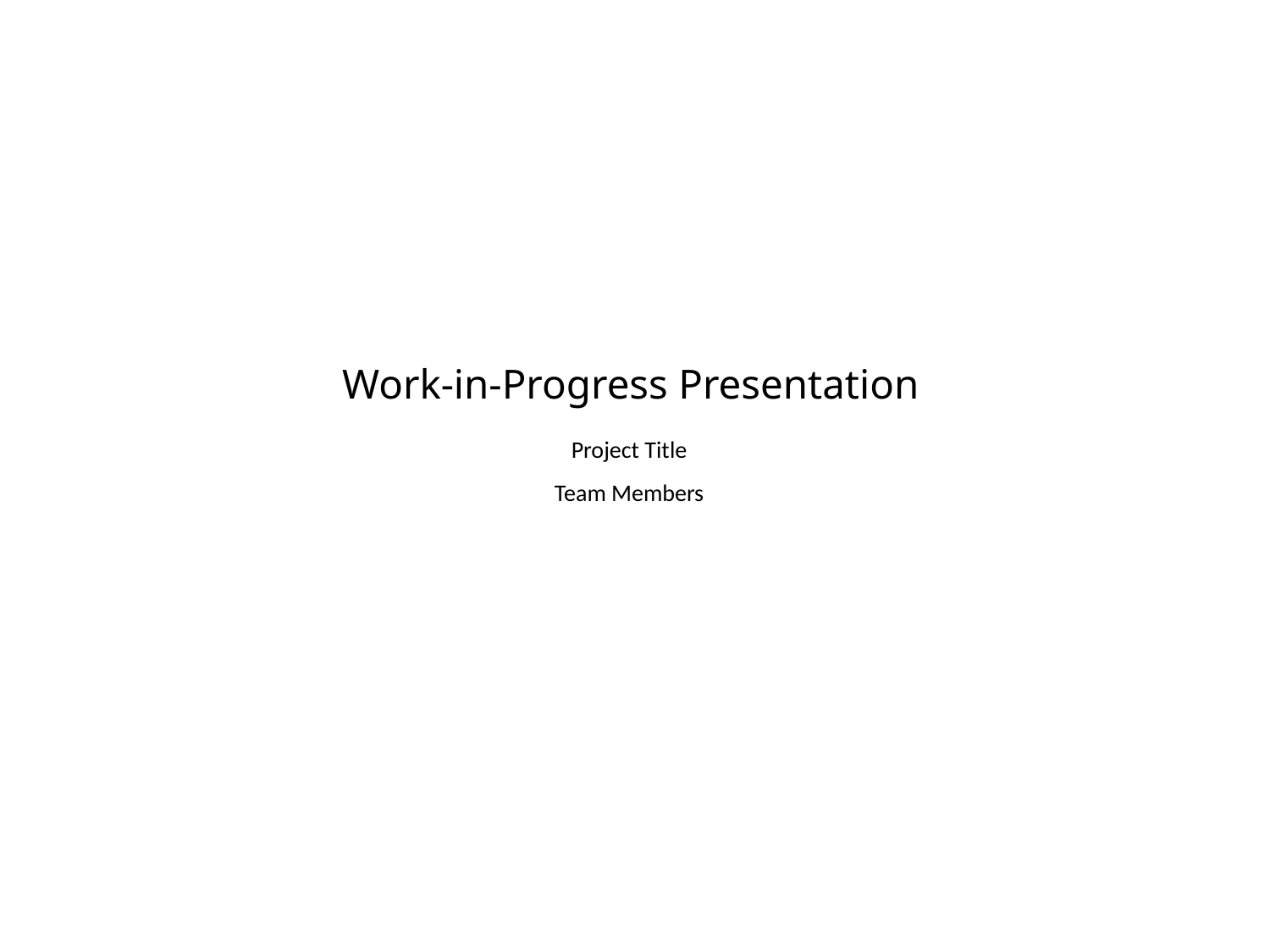

Work-in-Progress Presentation
Project Title
Team Members

## Slide 2
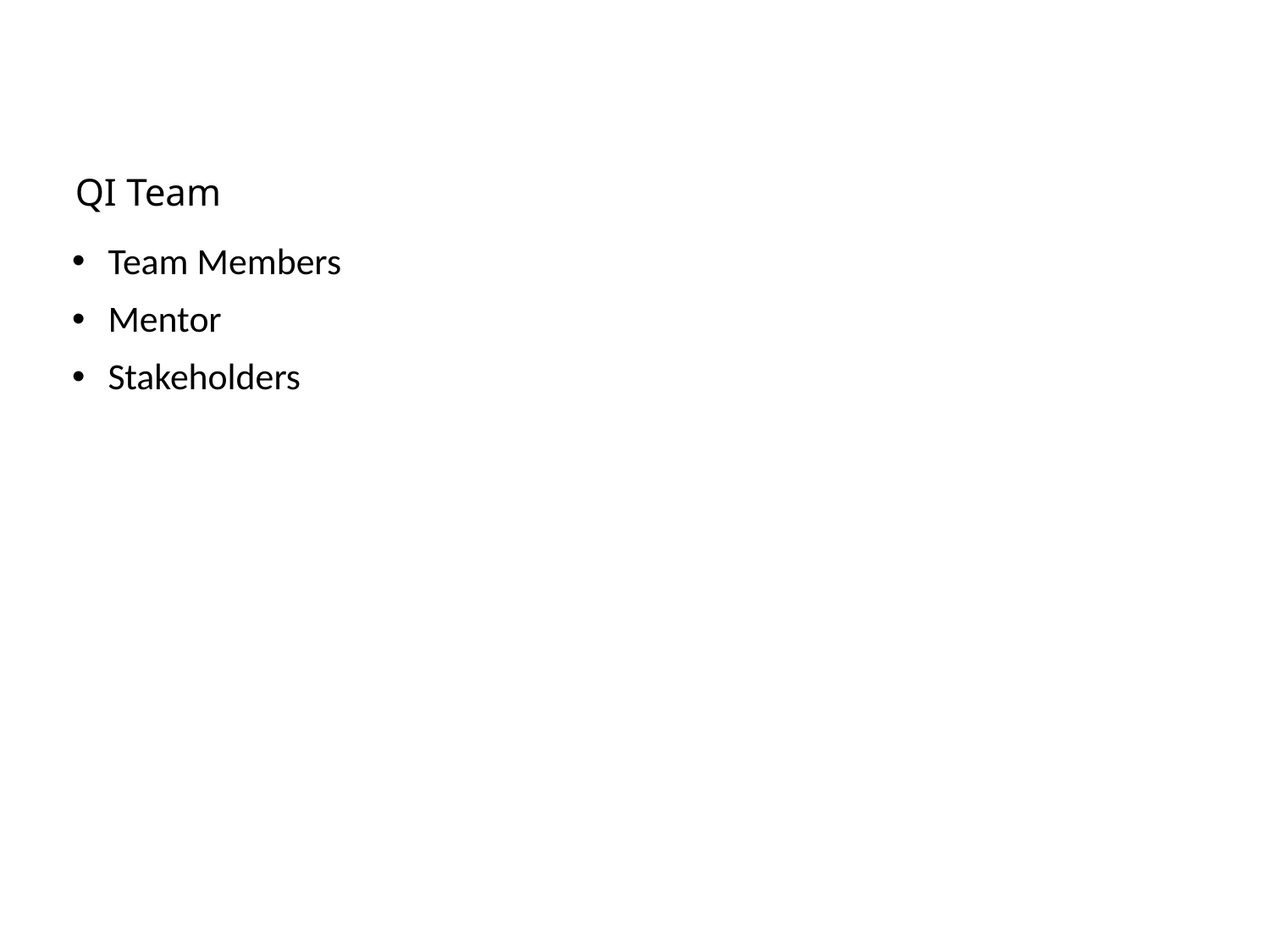

QI Team
Team Members
Mentor
Stakeholders

## Slide 3
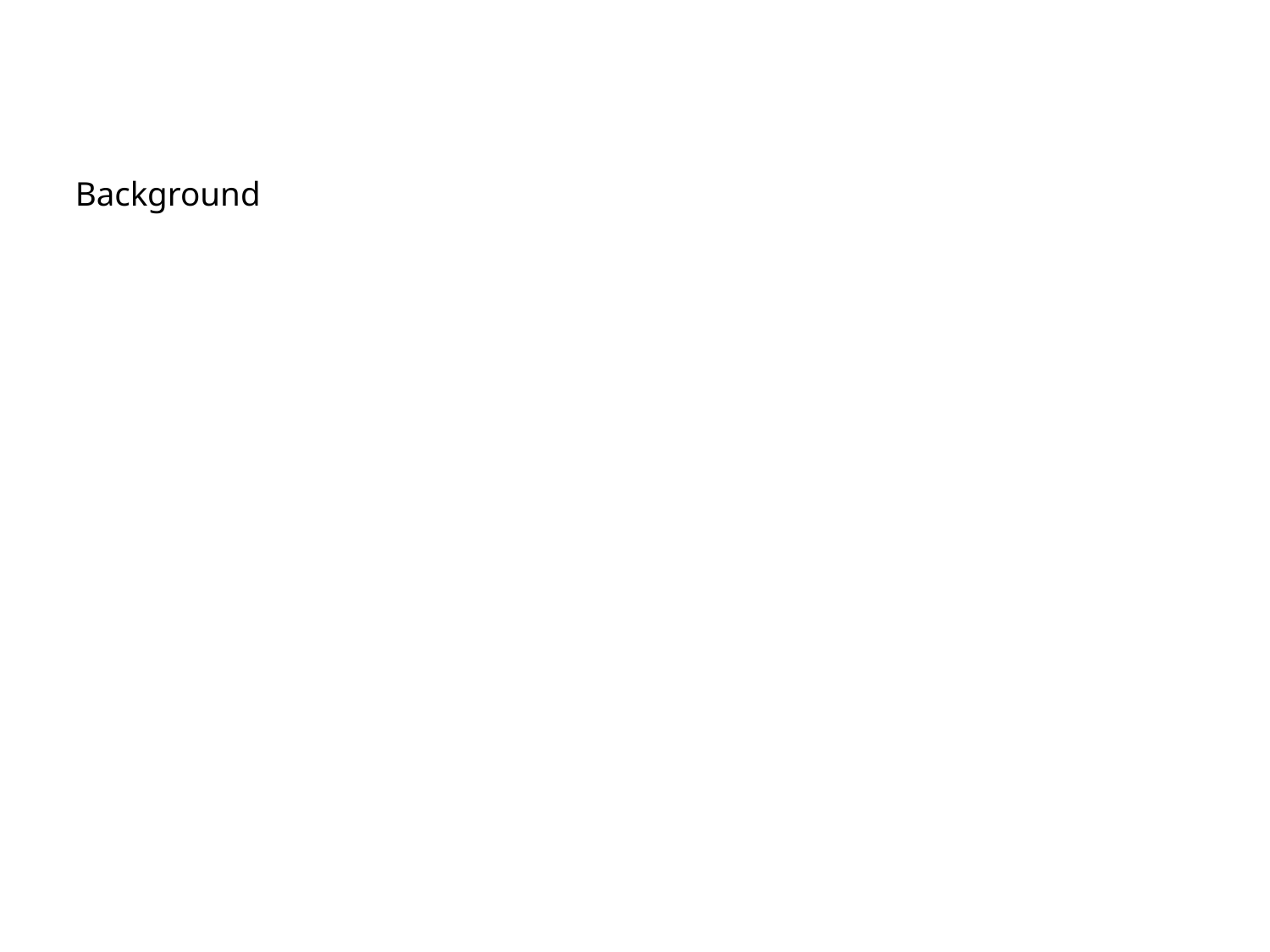

Background

## Slide 4
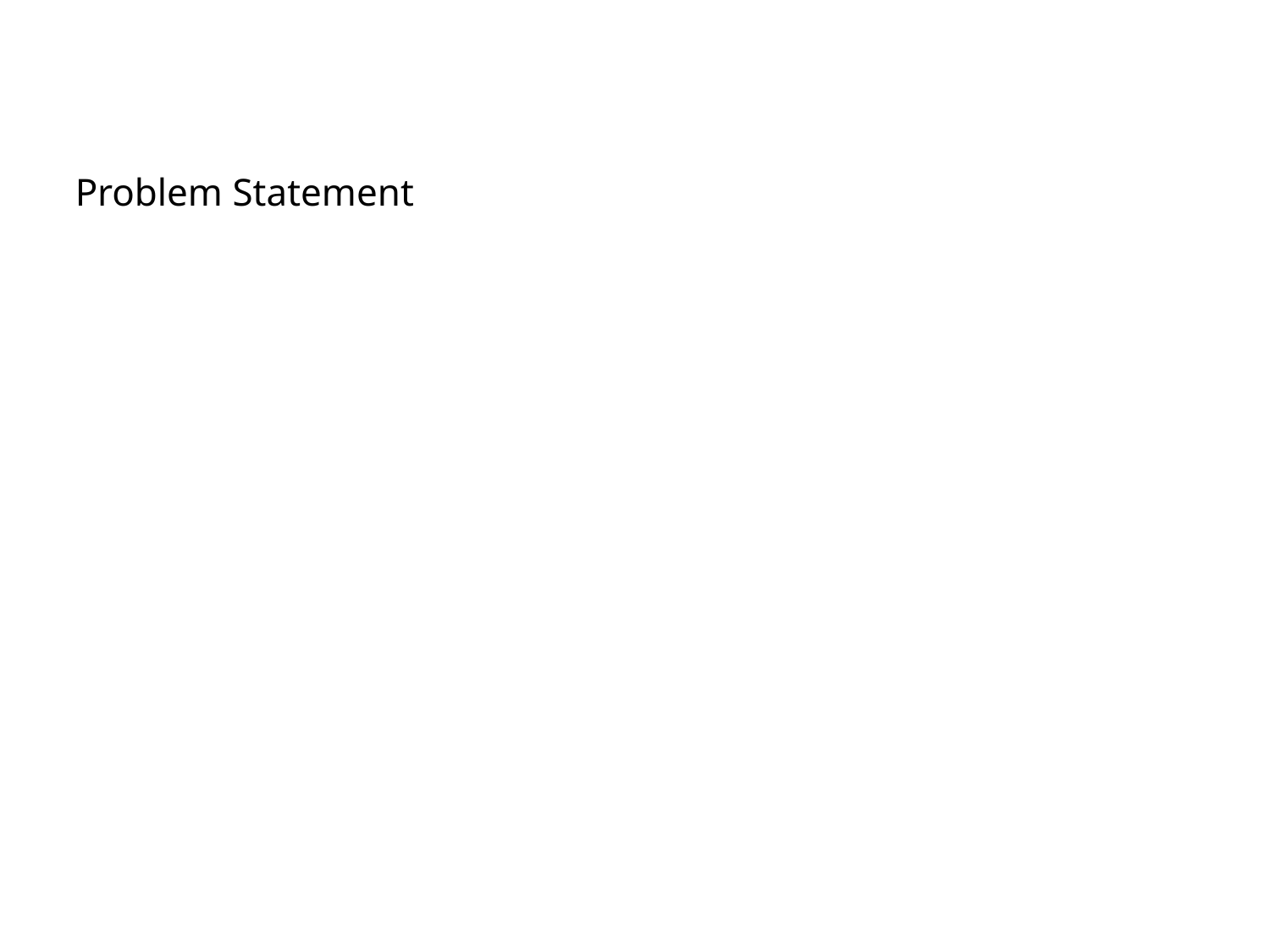

Problem Statement

## Slide 5
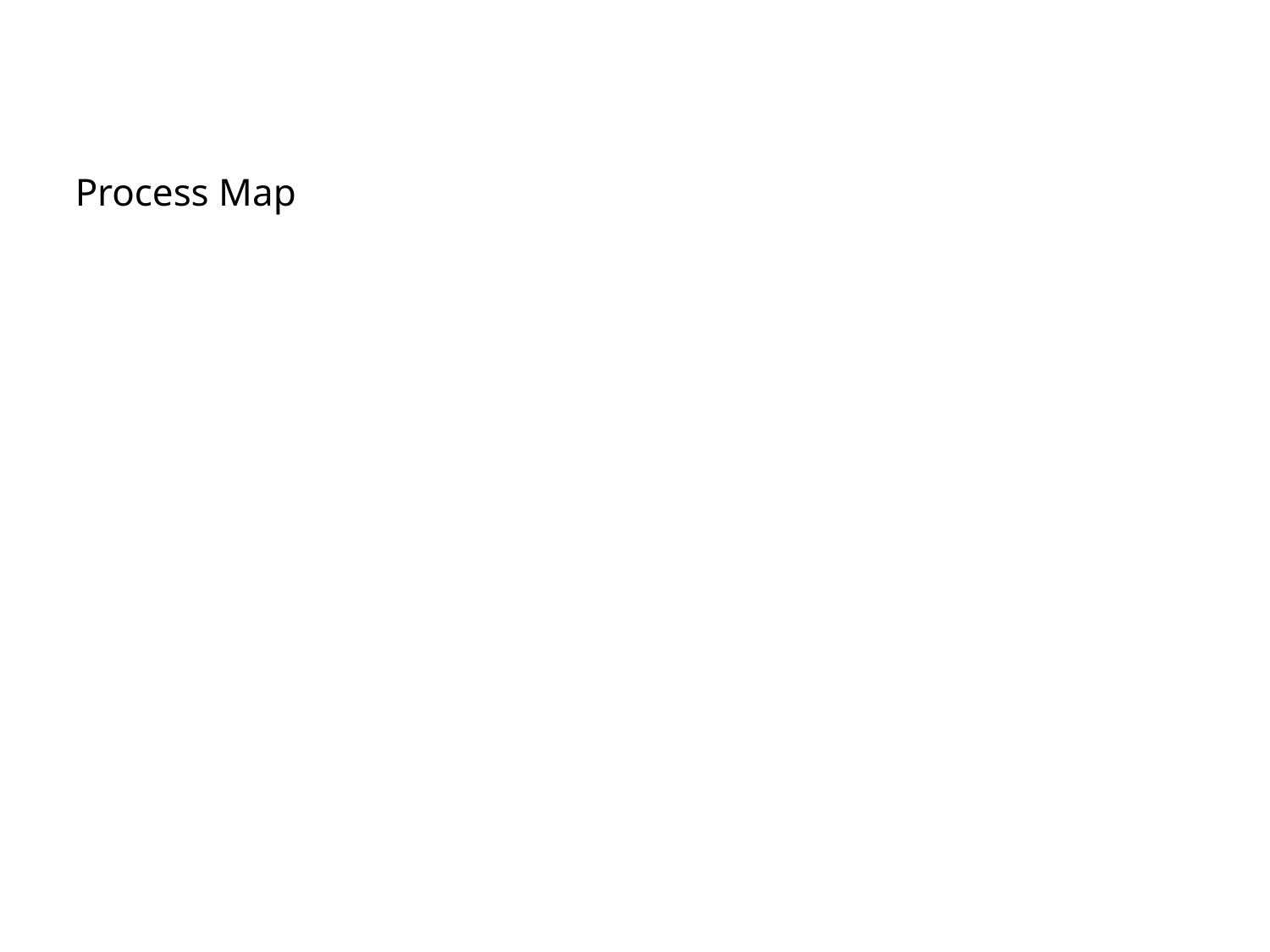

Process Map

## Slide 6
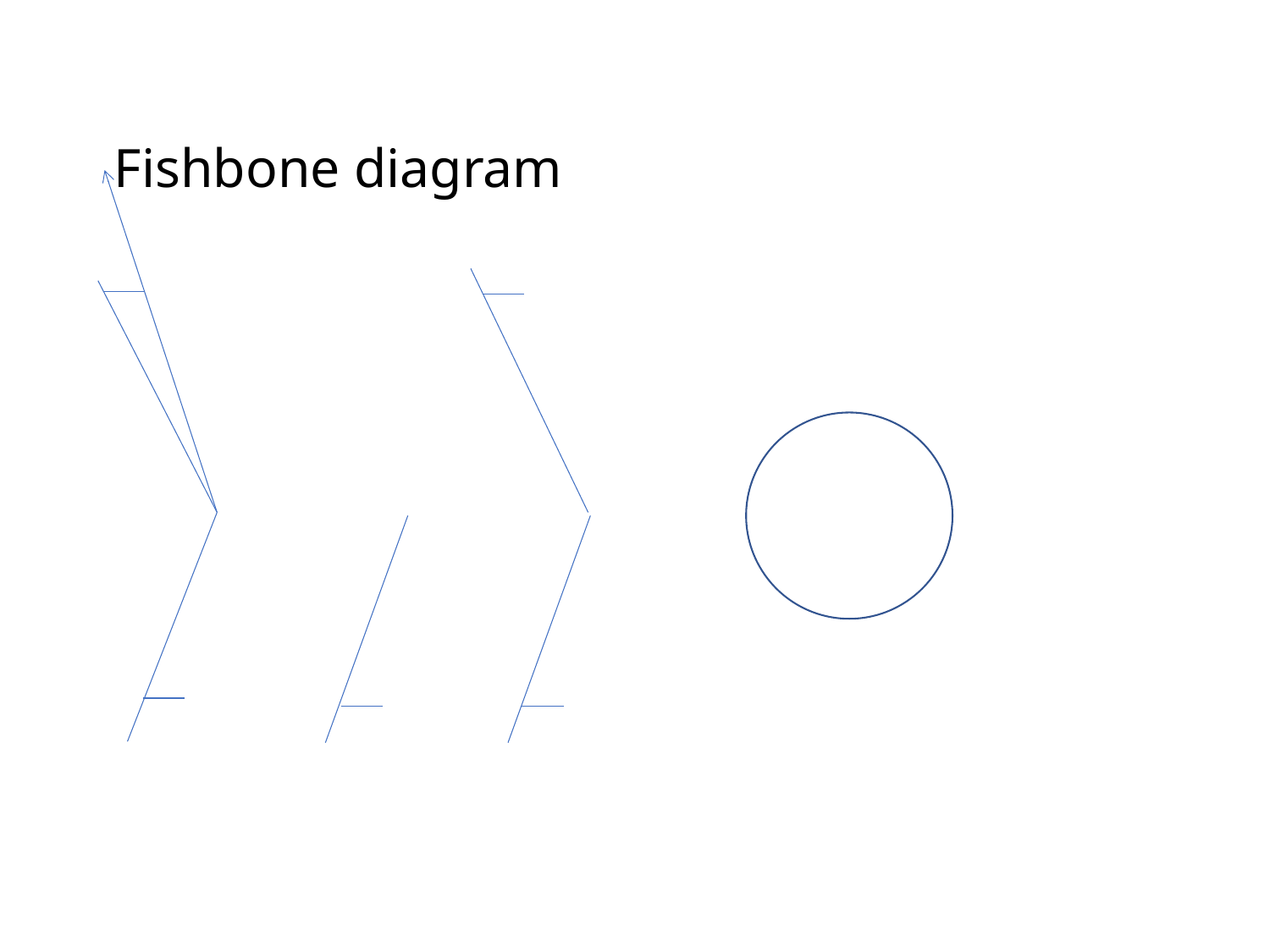

Fishbone diagram

## Slide 7
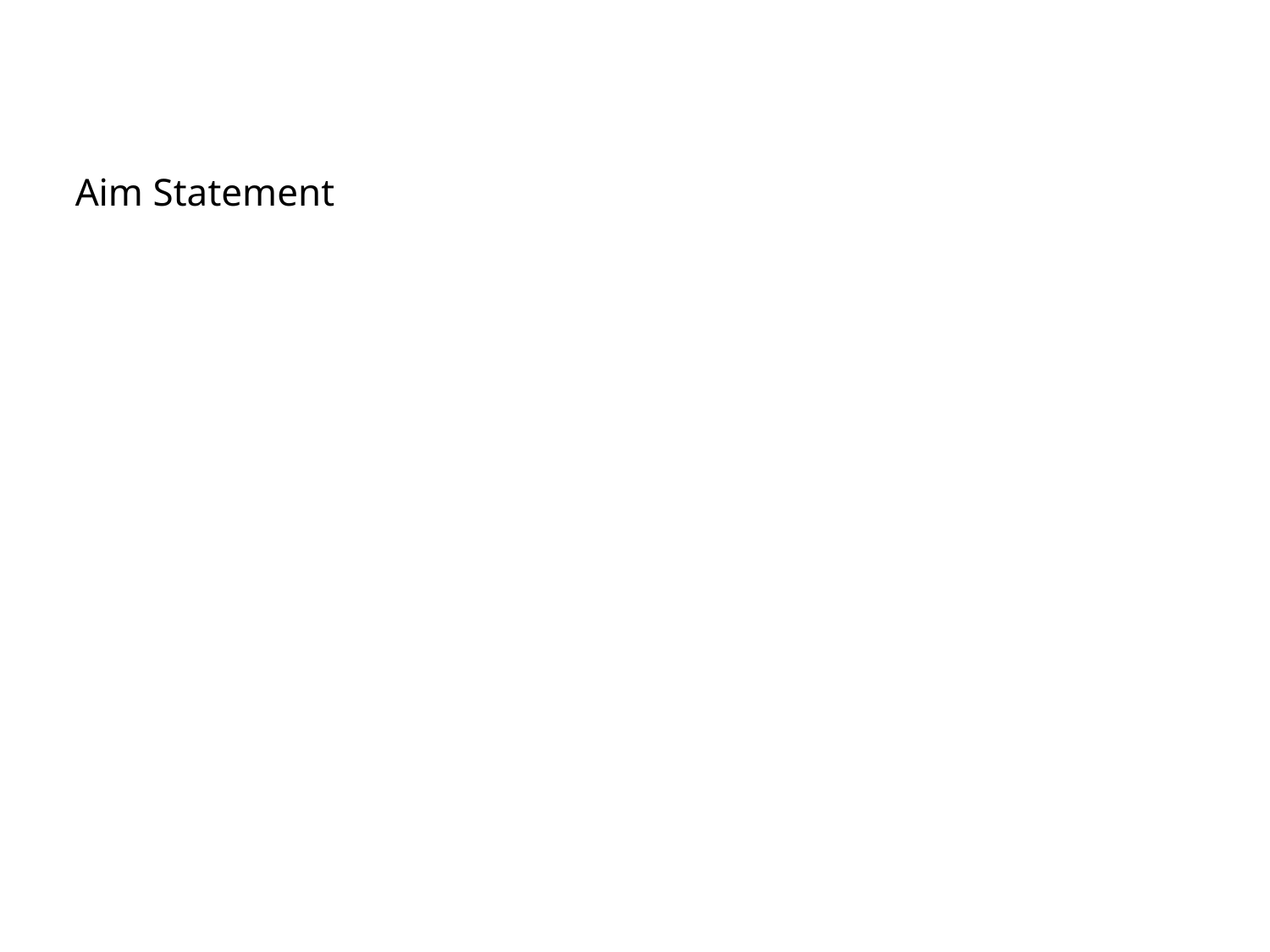

Aim Statement

## Slide 8
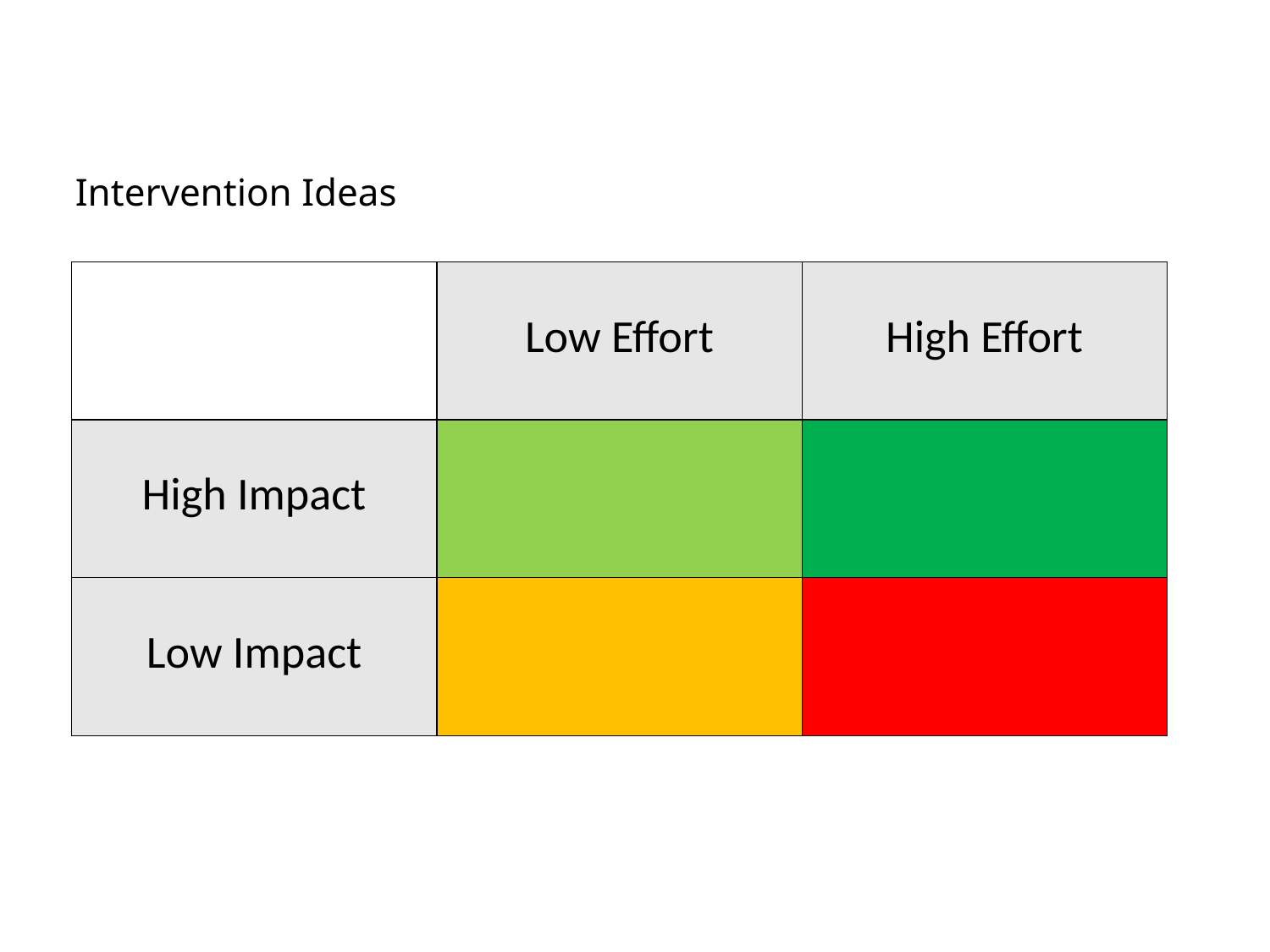

Intervention Ideas
| ​ | Low Effort​ | High Effort​ |
| --- | --- | --- |
| High Impact​ | ​ | ​ |
| Low Impact​ | ​ | ​ |

## Slide 9
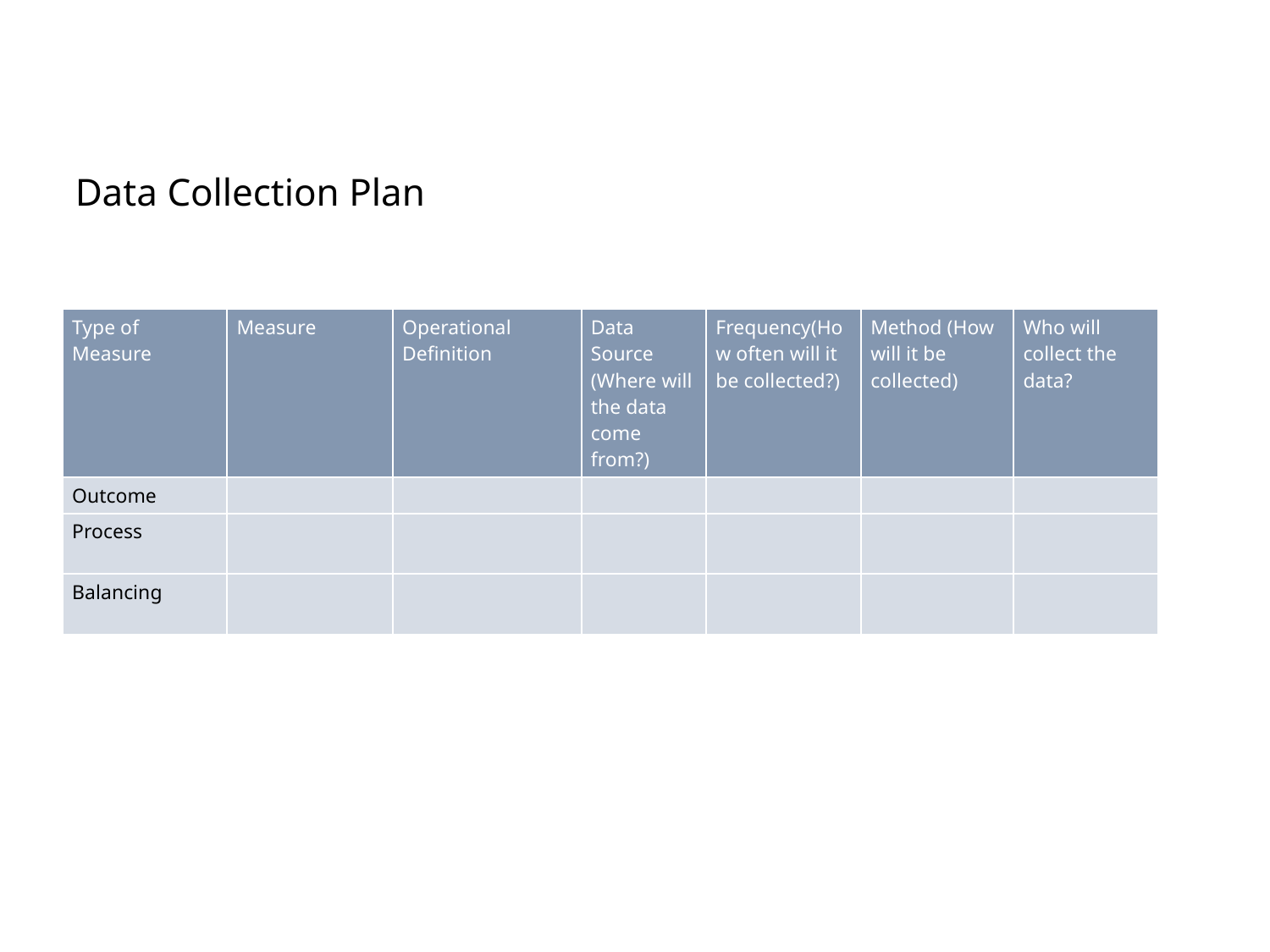

Data Collection Plan
| Type of Measure​ | Measure​ | Operational Definition​ | Data Source​ (Where will the data come from?)​ | Frequency(How often will it be collected?)​ ​ | Method (How will it be collected)​ ​ | Who will collect the data?​ |
| --- | --- | --- | --- | --- | --- | --- |
| ​Outcome | ​ | ​ | ​ | ​ | ​ | ​ |
| ​Process | ​ | ​ | ​ | ​ | ​ | ​ |
| ​Balancing | ​ | ​ | ​ | ​ | ​ | ​ |

## Slide 10
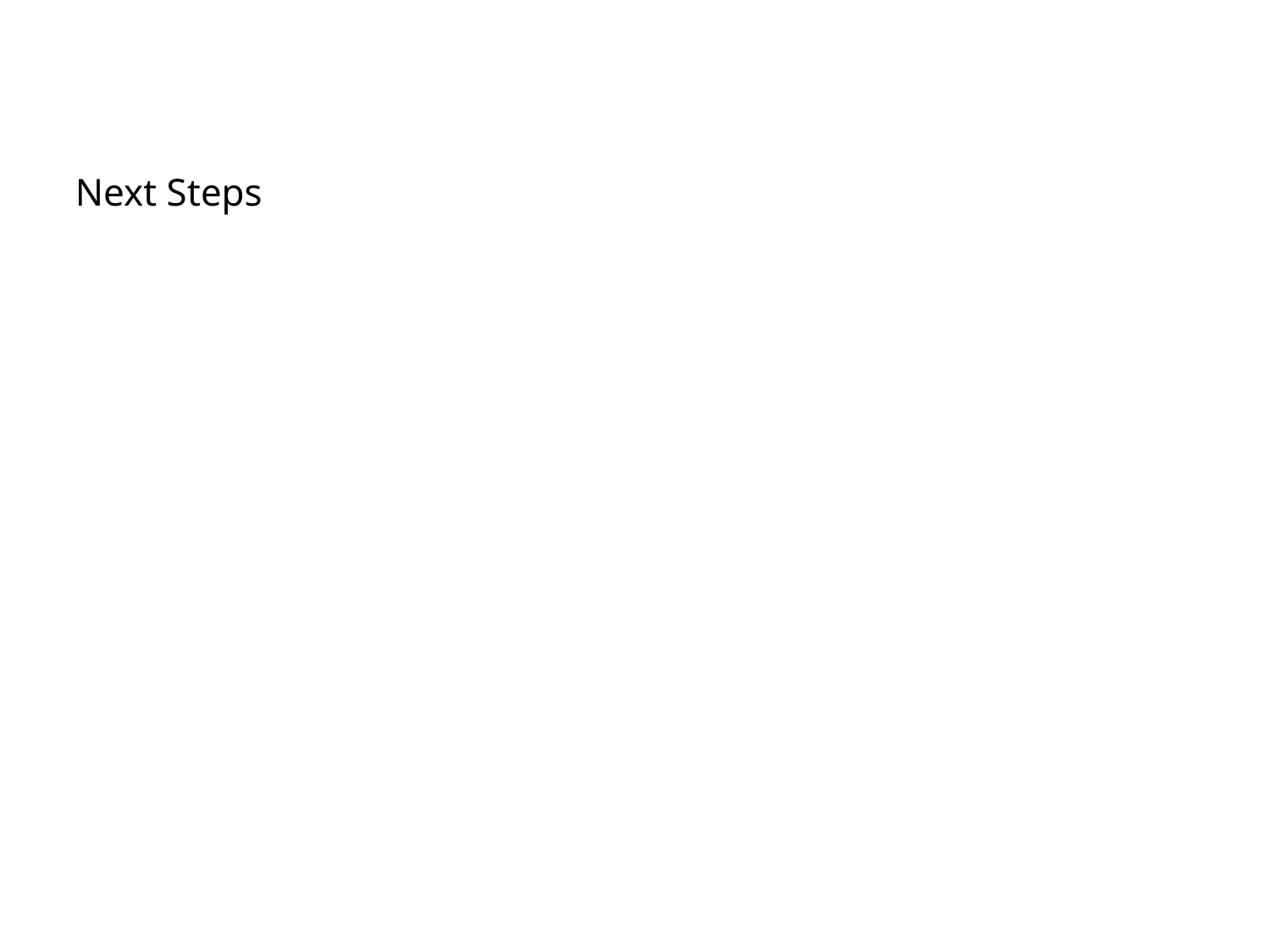

Next Steps

## Slide 11
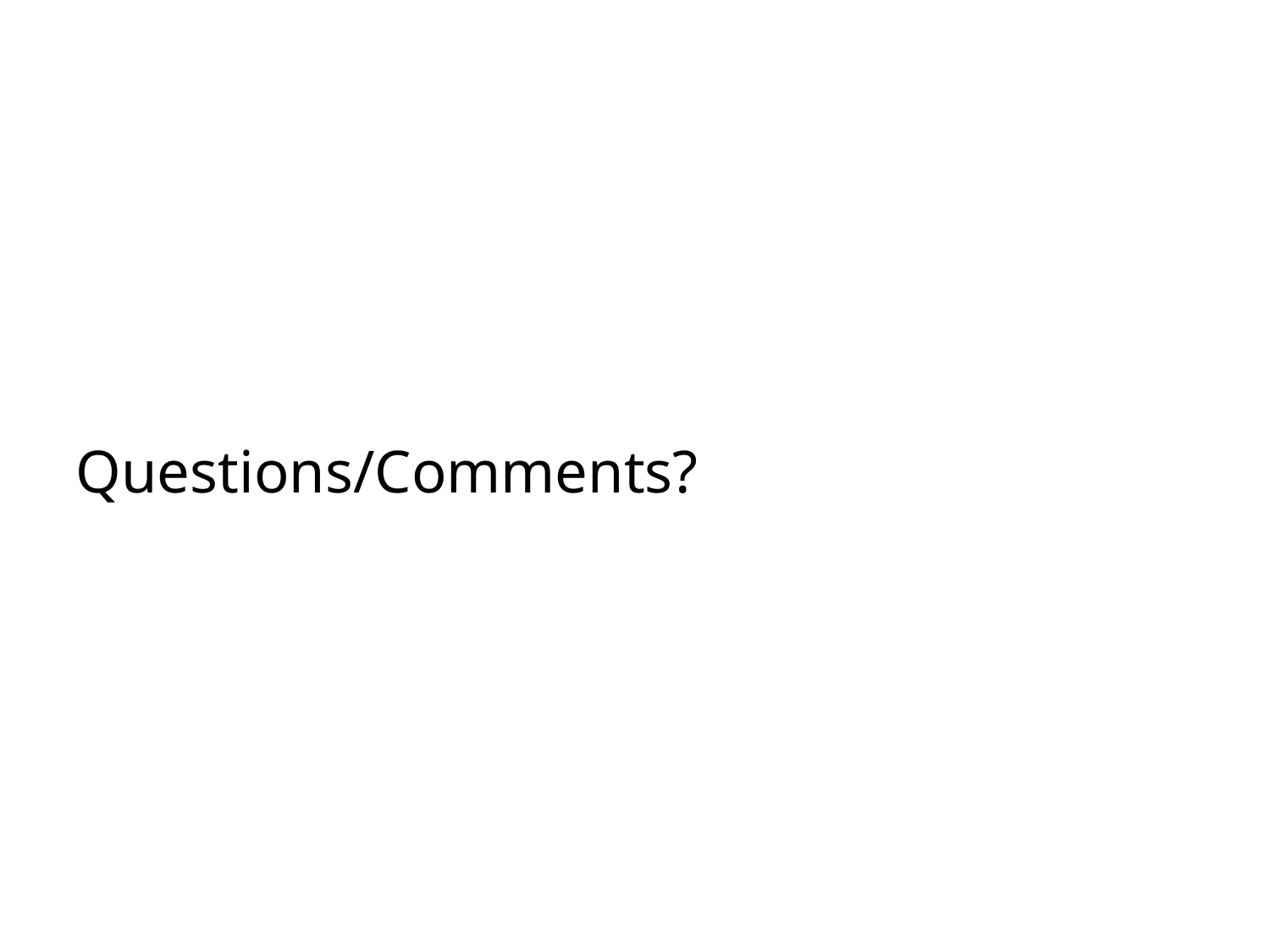

Questions/Comments?
