# Supplementary material for: Building Resident Quality Improvement Knowledge and Engagement Through a Longitudinal, Mentored, and Experiential Learning-Based Quality Improvement Curriculum
Source: MedEdPORTAL. 2023 Apr 18;19:11310. doi: 10.15766/mep_2374-8265.11310 (PMC10110773; doi:10.15766/mep_2374-8265.11310)
Supplement: Supplementary file 1 — Session 1 Slides.pptxSession 1 Workbook.pptxSession 2 Slides.pptxSession 2 Workbook.pptxSession 3 Slides.pptxSession 4 Work-in-Progress Presentation Template.pptxSession 5 Slides.pptxQI Charter Template.docxFaculty Milestones.docxFaculty Guide.docxResident Survey.docx [file mep_2374-8265.11310-s001.zip › G. Session 5 Slides.pptx]

## Slide 1
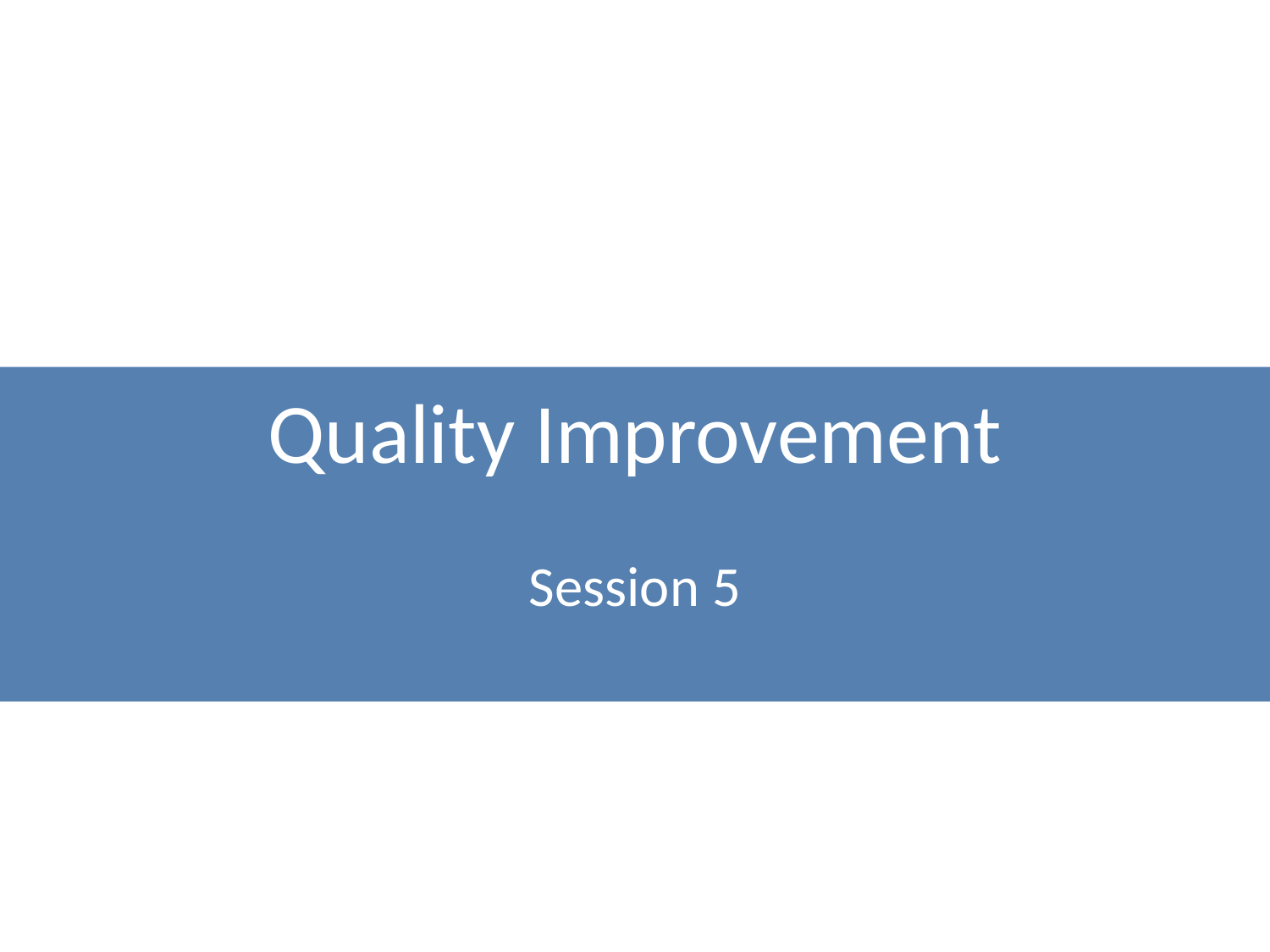

# Quality Improvement Session 5

## Slide 2
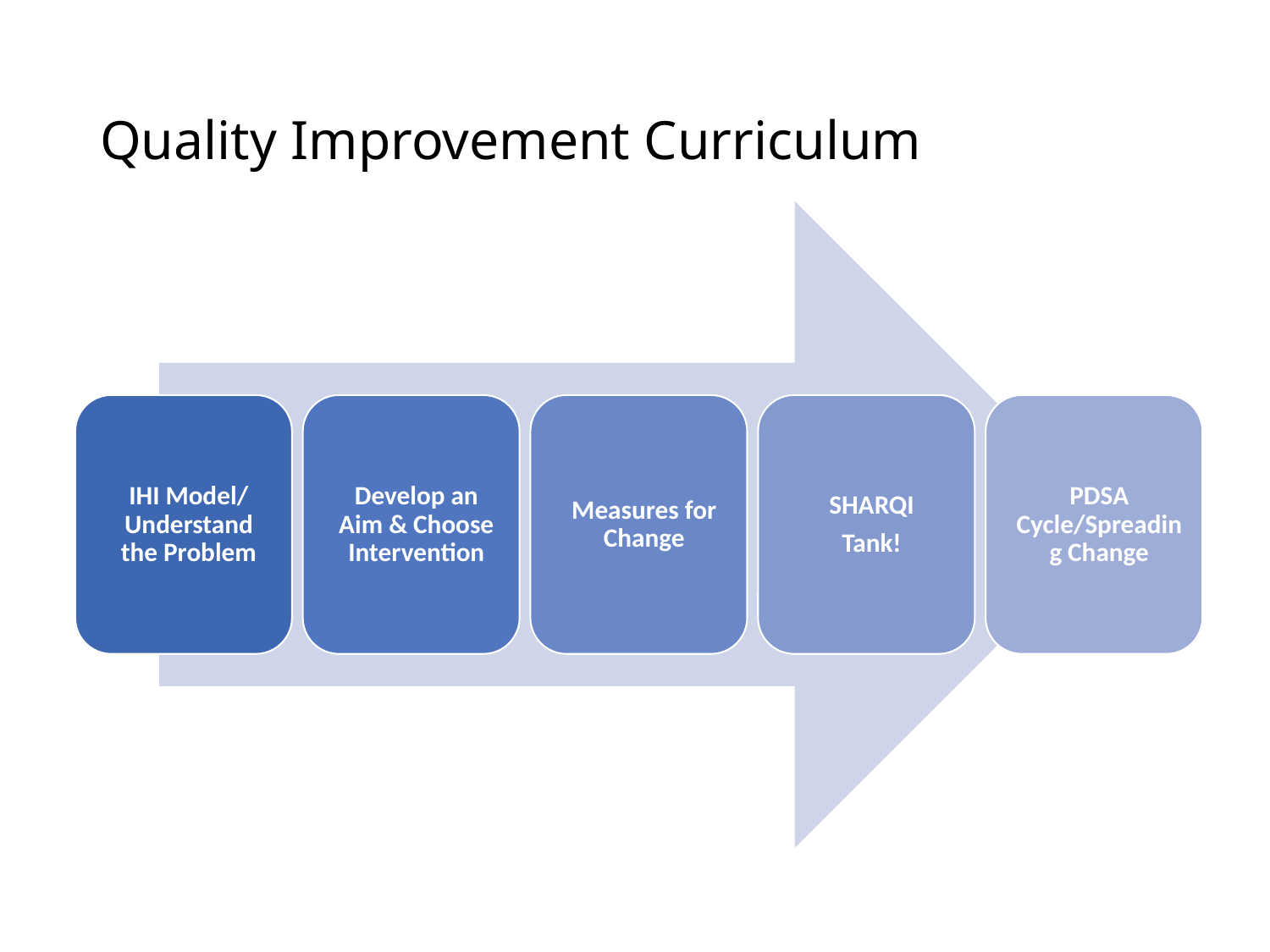

# Quality Improvement Curriculum

## Slide 3
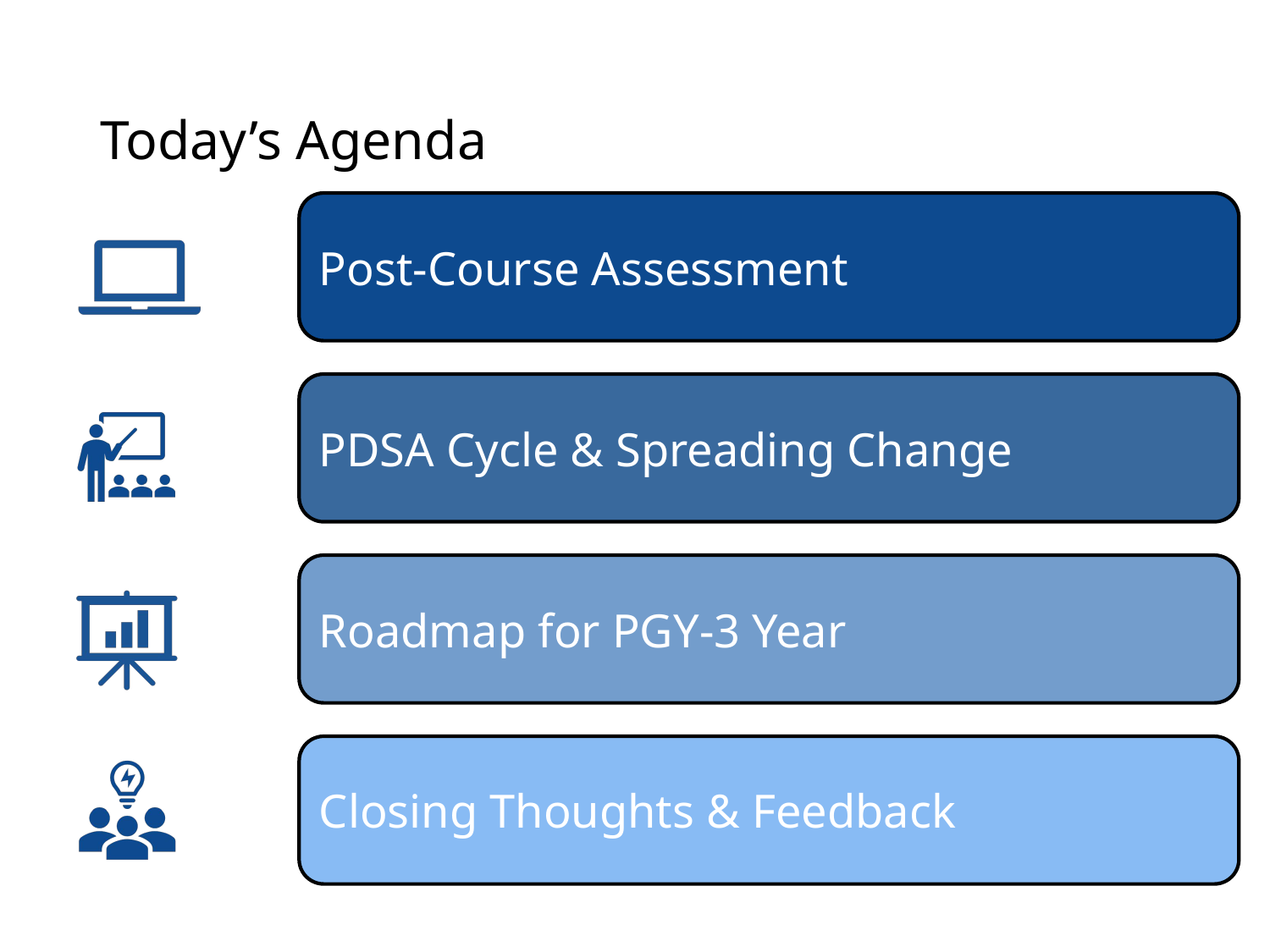

# Today’s Agenda
Post-Course Assessment
PDSA Cycle & Spreading Change
Roadmap for PGY-3 Year
Closing Thoughts & Feedback

## Slide 4
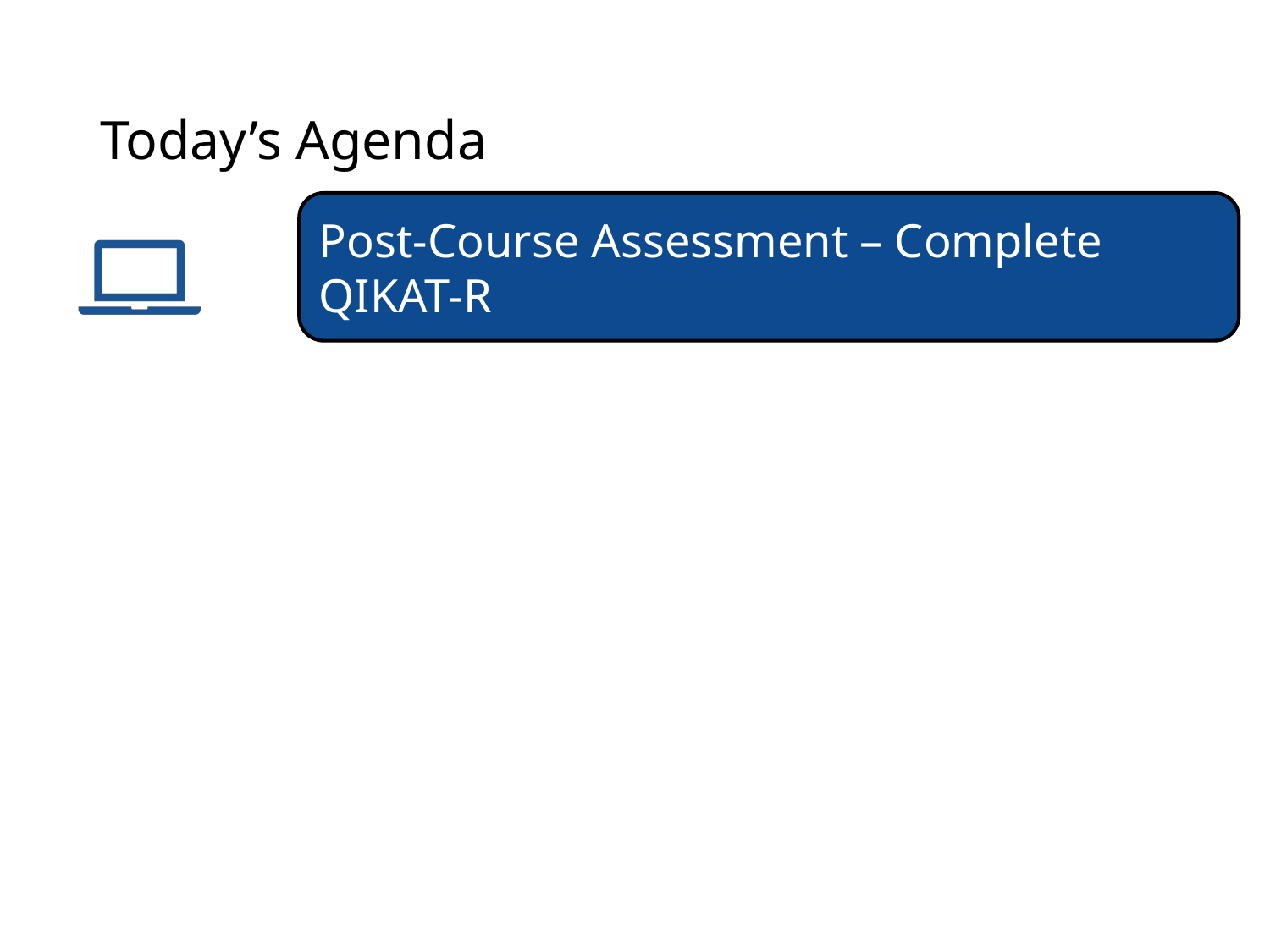

# Today’s Agenda
Post-Course Assessment – Complete QIKAT-R

## Slide 5
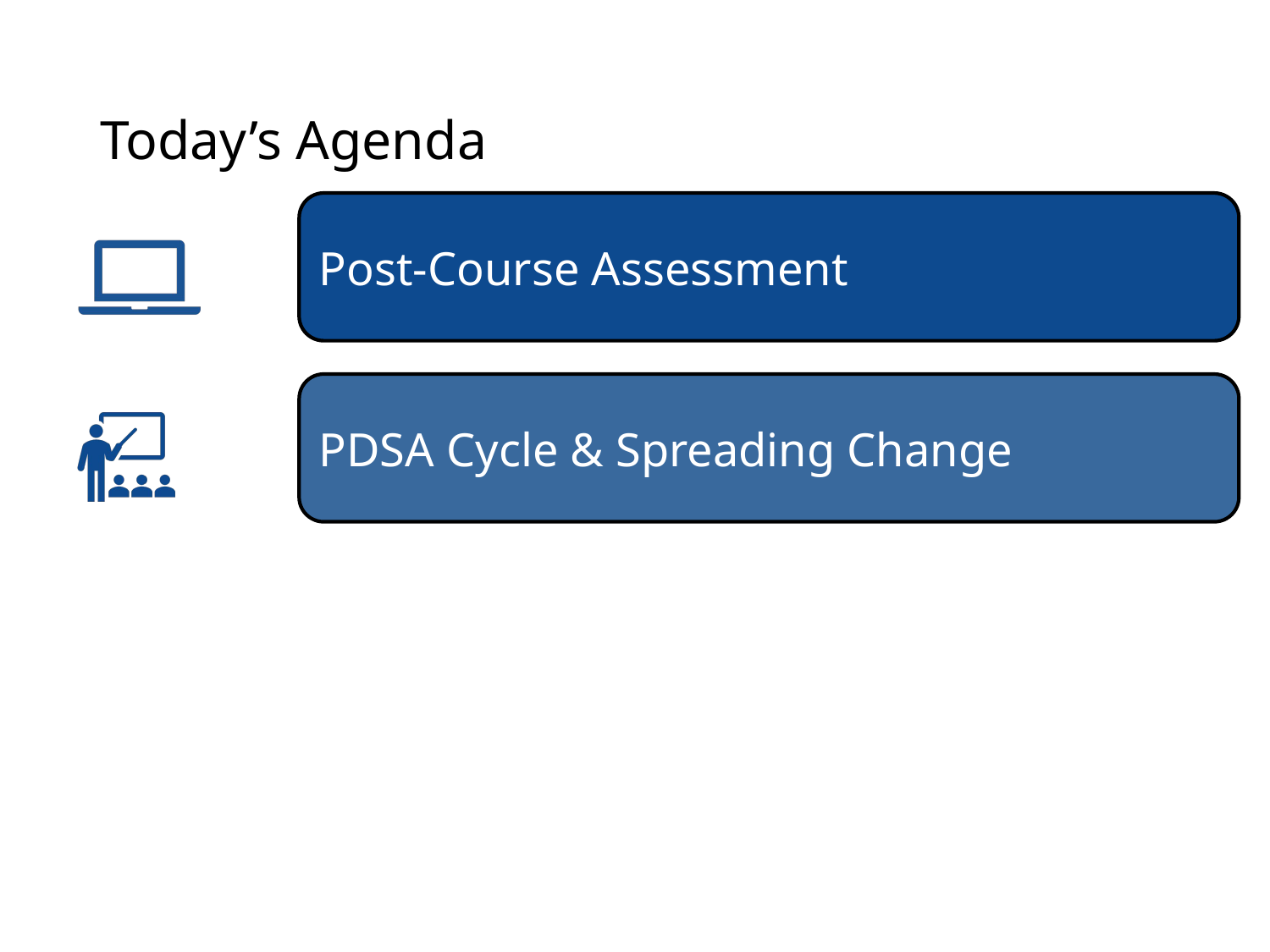

# Today’s Agenda
Post-Course Assessment
PDSA Cycle & Spreading Change

## Slide 6
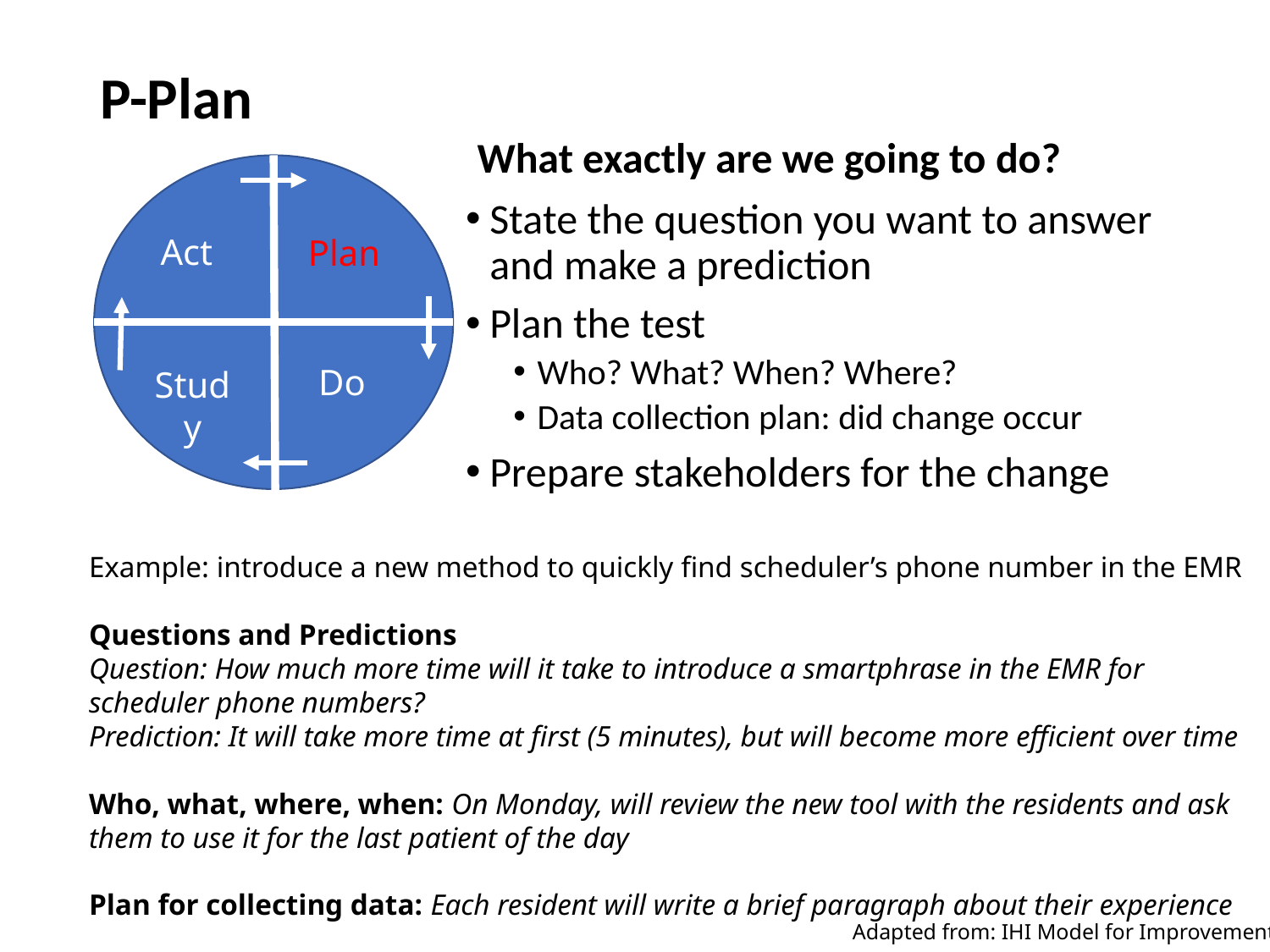

# P-Plan
 What exactly are we going to do?
State the question you want to answer and make a prediction
Plan the test
Who? What? When? Where?
Data collection plan: did change occur
Prepare stakeholders for the change
Act
Plan
Do
Study
Example: introduce a new method to quickly find scheduler’s phone number in the EMR
Questions and Predictions
Question: How much more time will it take to introduce a smartphrase in the EMR for scheduler phone numbers?
Prediction: It will take more time at first (5 minutes), but will become more efficient over time
Who, what, where, when: On Monday, will review the new tool with the residents and ask them to use it for the last patient of the day
Plan for collecting data: Each resident will write a brief paragraph about their experience
Adapted from: IHI Model for Improvement

## Slide 7
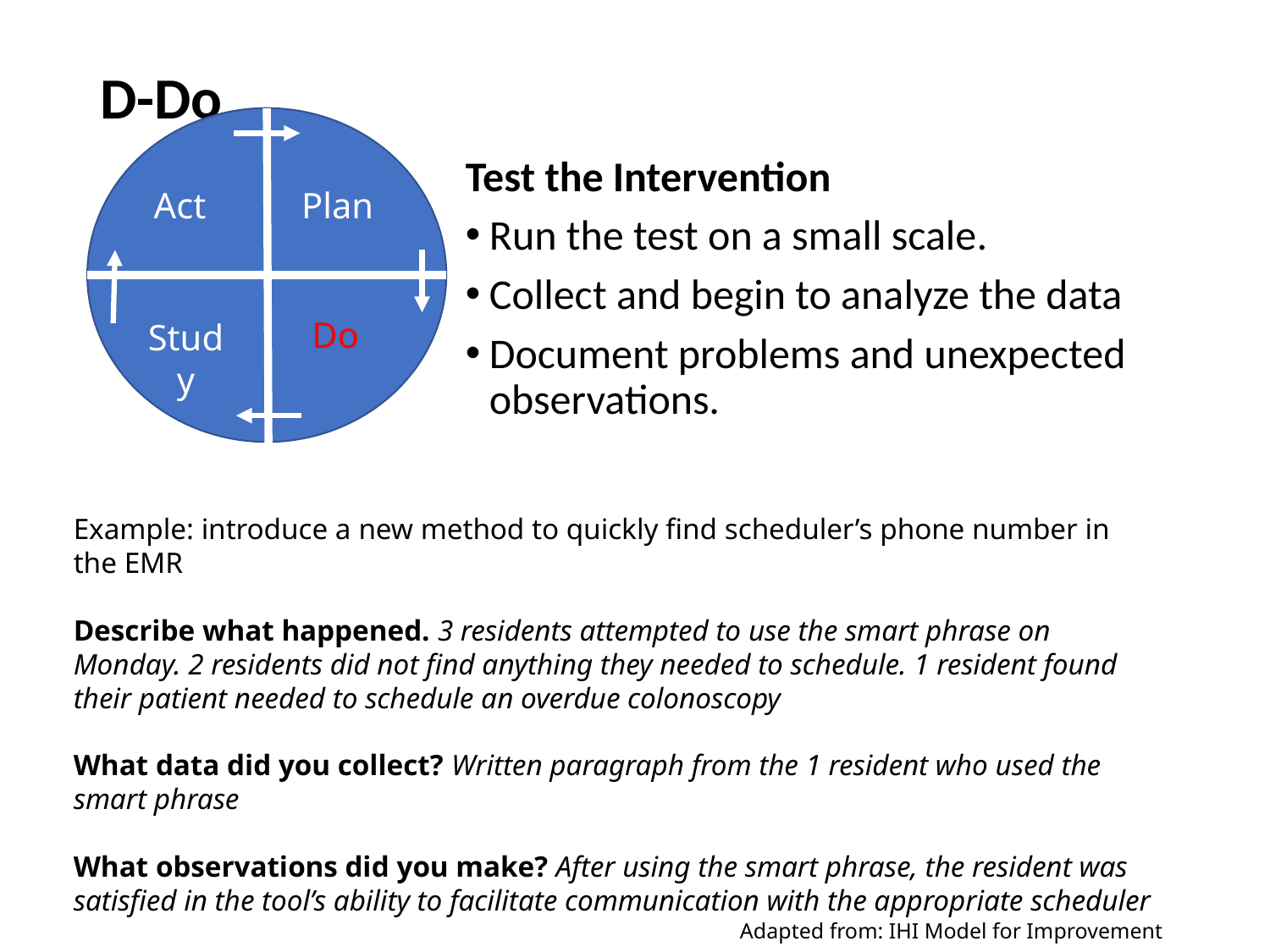

D-Do
Test the Intervention
Run the test on a small scale.
Collect and begin to analyze the data
Document problems and unexpected observations.
Act
Plan
Do
Study
Example: introduce a new method to quickly find scheduler’s phone number in the EMR
Describe what happened. 3 residents attempted to use the smart phrase on Monday. 2 residents did not find anything they needed to schedule. 1 resident found their patient needed to schedule an overdue colonoscopy
What data did you collect? Written paragraph from the 1 resident who used the smart phrase
What observations did you make? After using the smart phrase, the resident was satisfied in the tool’s ability to facilitate communication with the appropriate scheduler
Adapted from: IHI Model for Improvement

## Slide 8
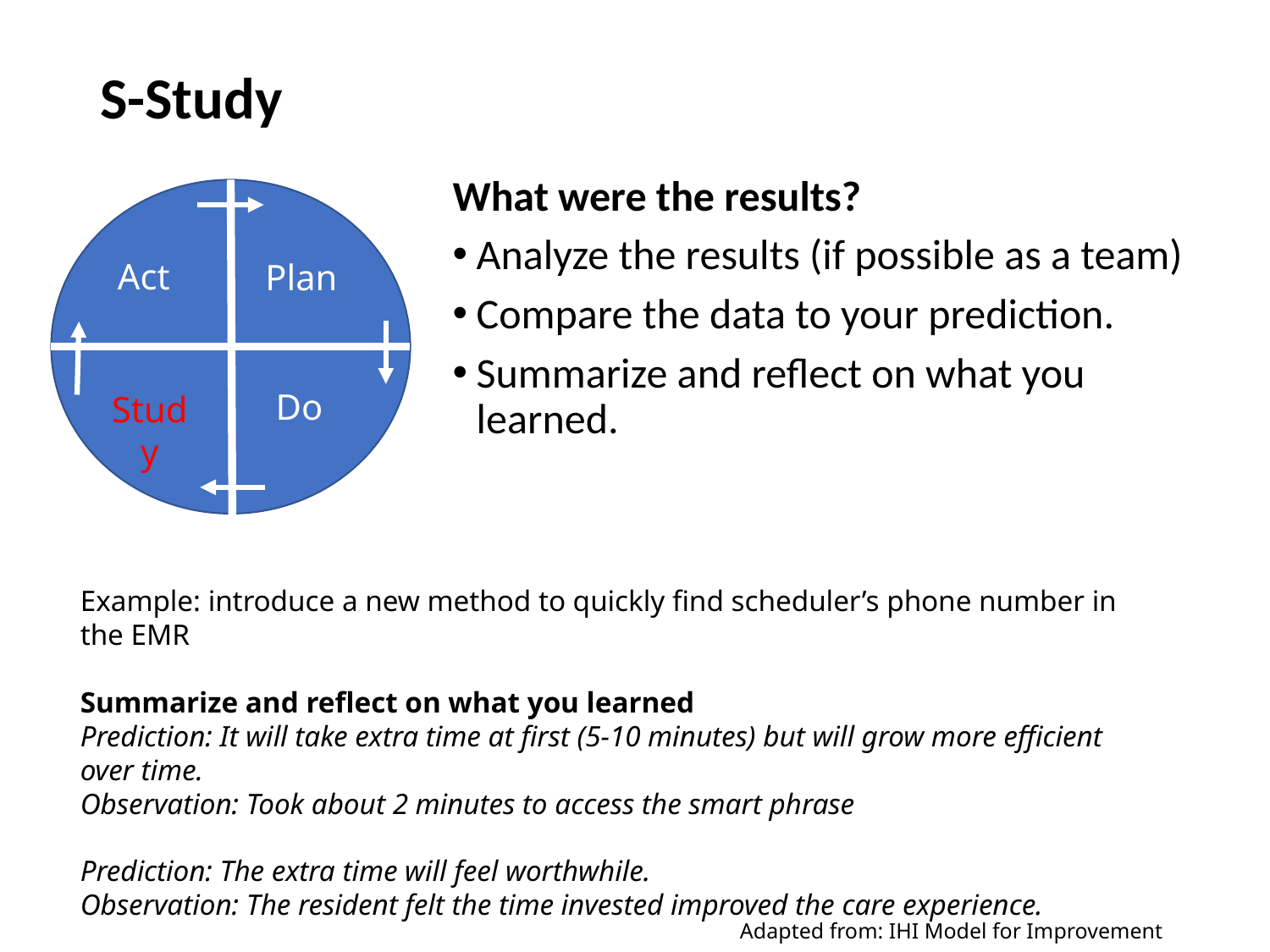

S-Study
What were the results?
Analyze the results (if possible as a team)
Compare the data to your prediction.
Summarize and reflect on what you learned.
Act
Plan
Do
Study
Example: introduce a new method to quickly find scheduler’s phone number in the EMR
Summarize and reflect on what you learned
Prediction: It will take extra time at first (5-10 minutes) but will grow more efficient over time.
Observation: Took about 2 minutes to access the smart phrase
Prediction: The extra time will feel worthwhile.
Observation: The resident felt the time invested improved the care experience.
Adapted from: IHI Model for Improvement

## Slide 9
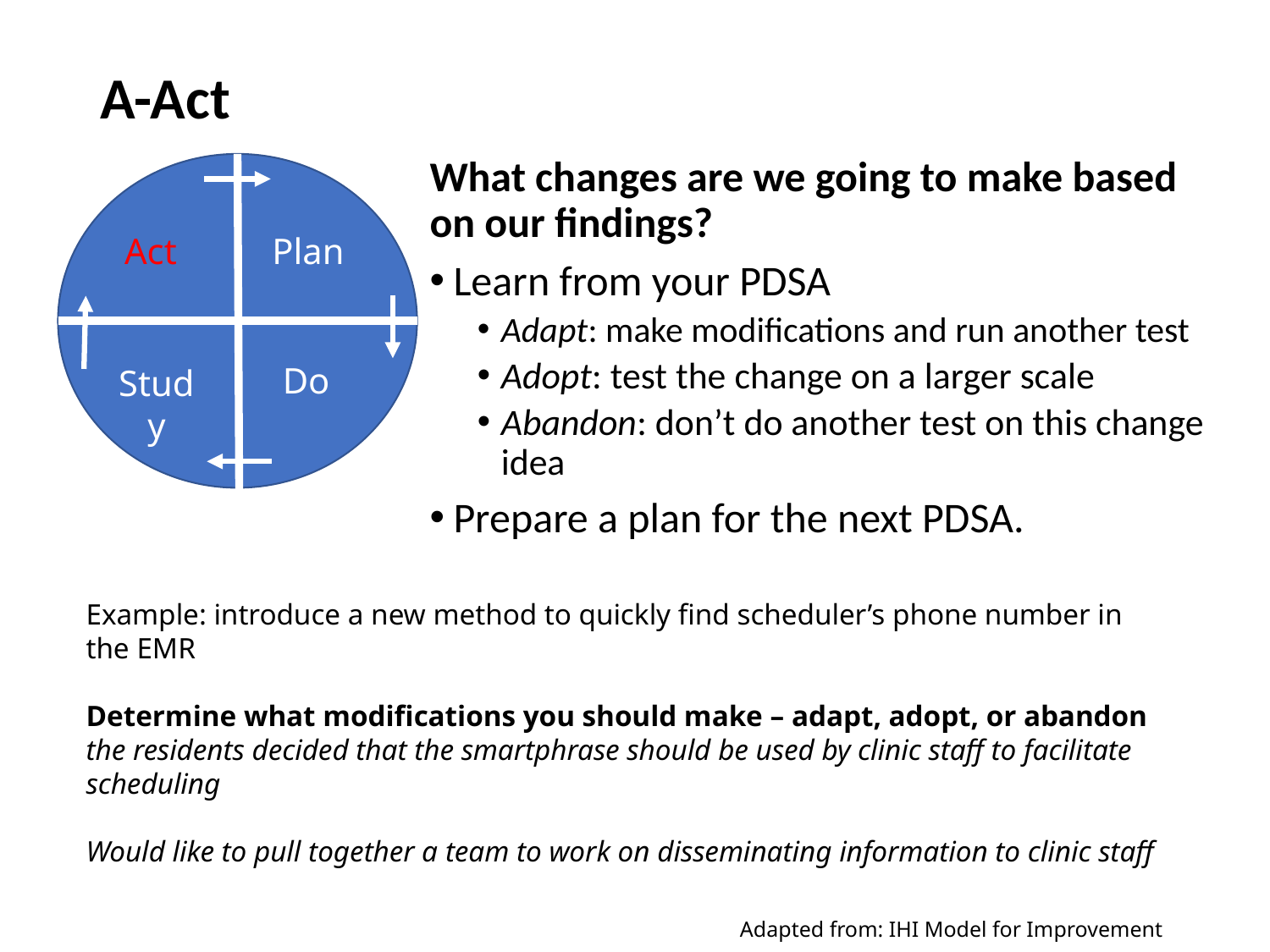

A-Act
What changes are we going to make based on our findings?
Learn from your PDSA
Adapt: make modifications and run another test
Adopt: test the change on a larger scale
Abandon: don’t do another test on this change idea
Prepare a plan for the next PDSA.
Act
Plan
Do
Study
Example: introduce a new method to quickly find scheduler’s phone number in the EMR
Determine what modifications you should make – adapt, adopt, or abandon
the residents decided that the smartphrase should be used by clinic staff to facilitate scheduling
Would like to pull together a team to work on disseminating information to clinic staff
Adapted from: IHI Model for Improvement

## Slide 10
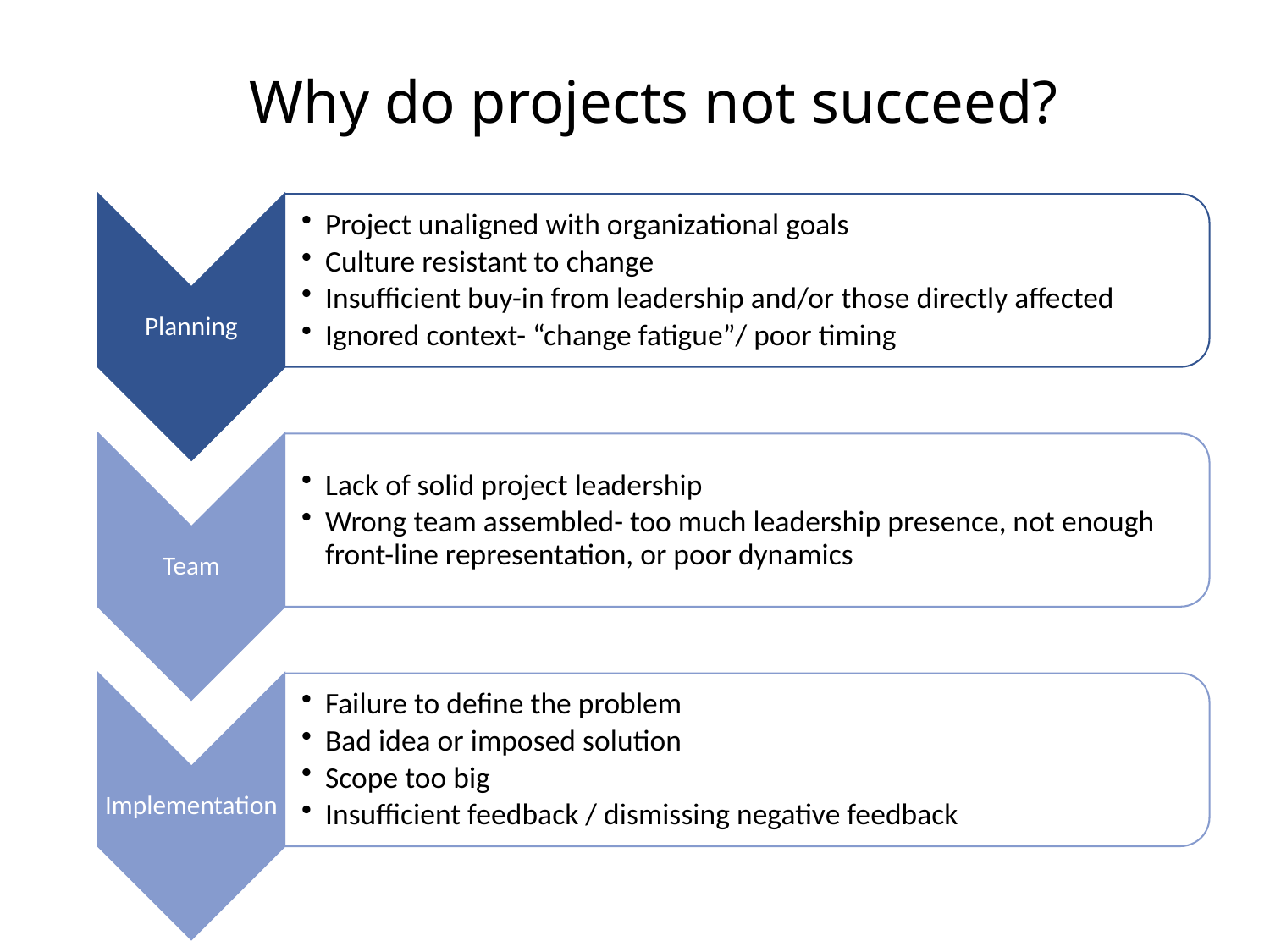

# Why do projects not succeed?

## Slide 11
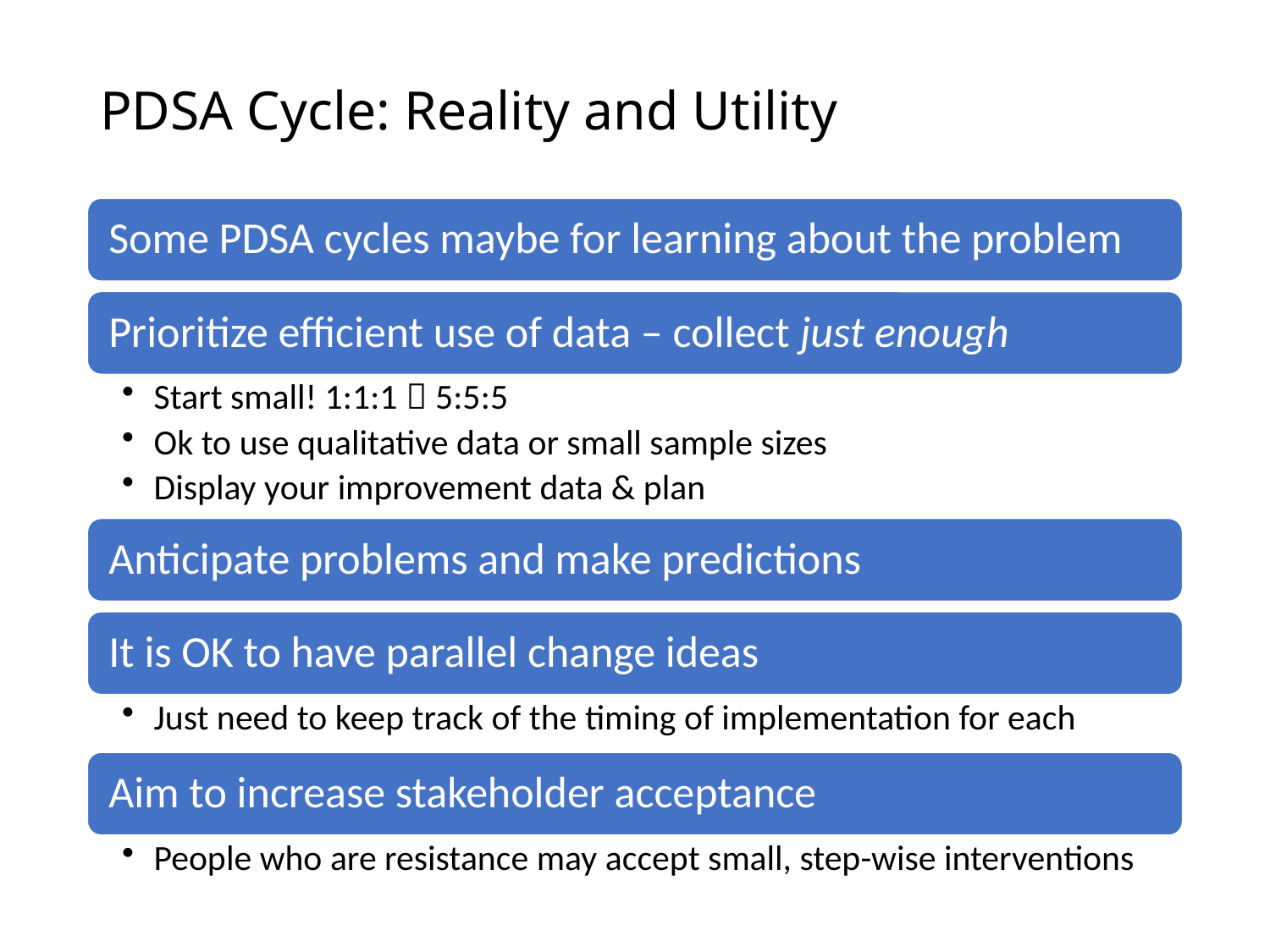

# PDSA Cycle: Reality and Utility

## Slide 12
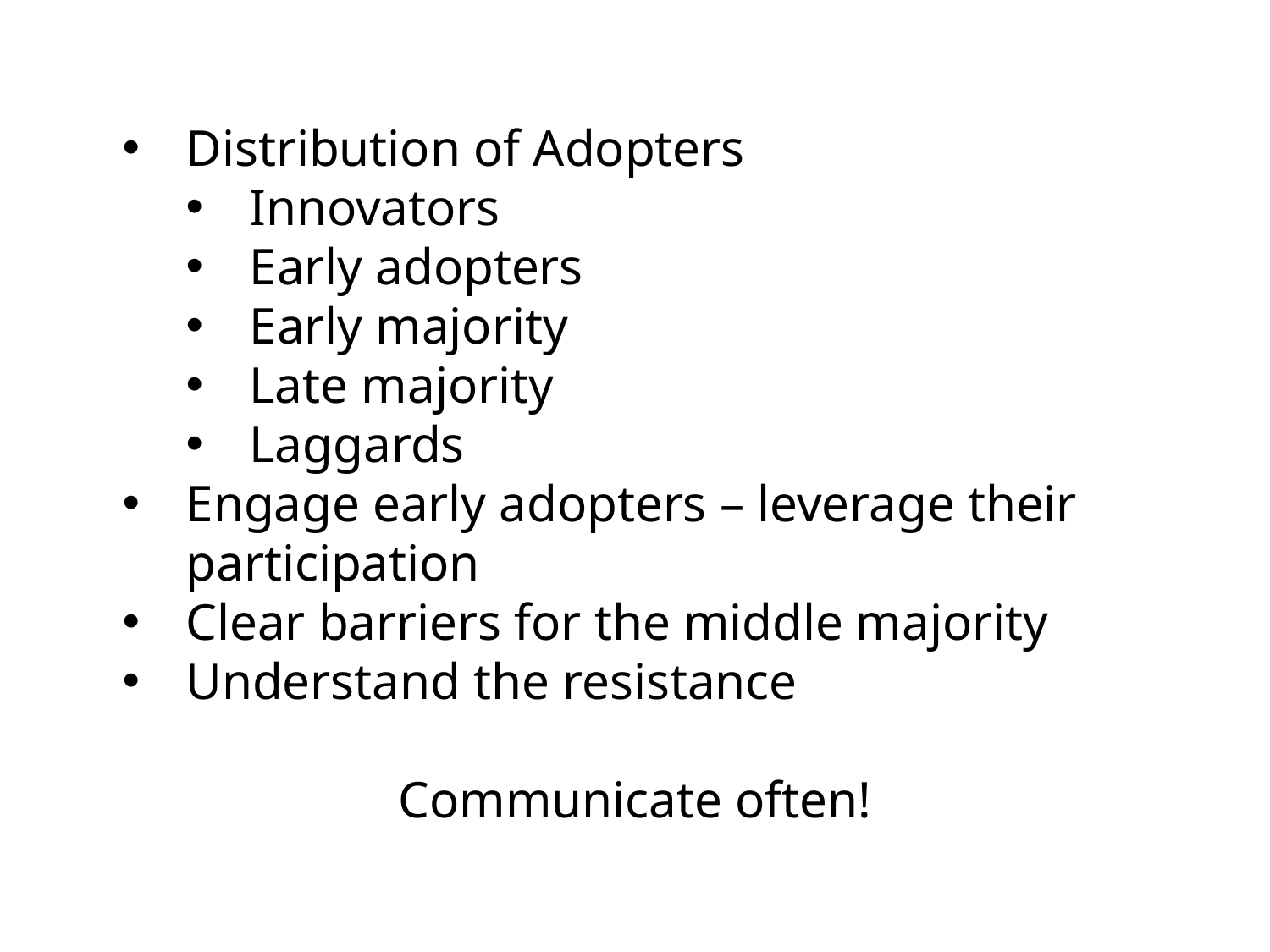

Distribution of Adopters
Innovators
Early adopters
Early majority
Late majority
Laggards
Engage early adopters – leverage their participation
Clear barriers for the middle majority
Understand the resistance
Communicate often!

## Slide 13
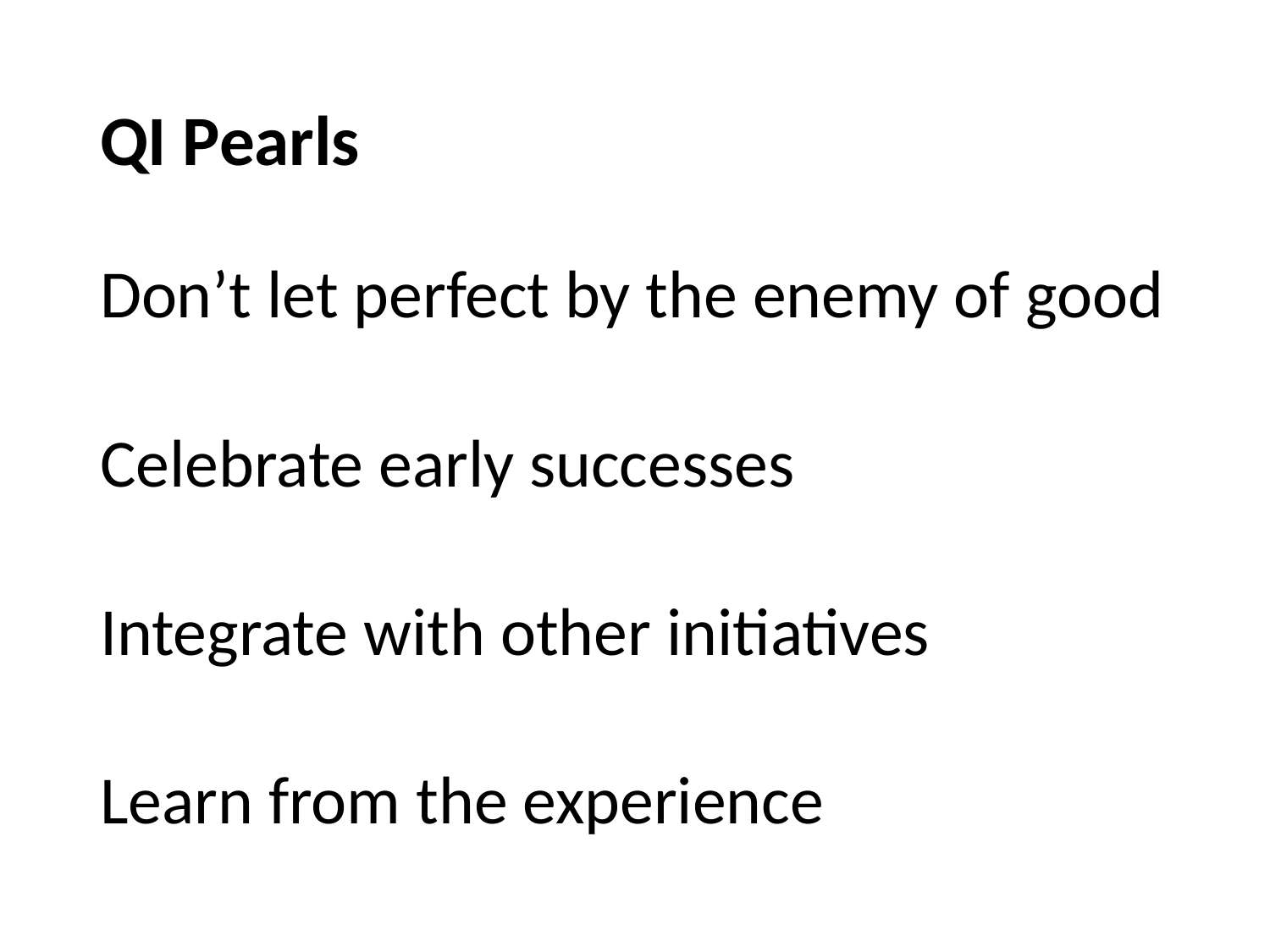

# QI Pearls
Don’t let perfect by the enemy of good
Celebrate early successes
Integrate with other initiatives
Learn from the experience

## Slide 14
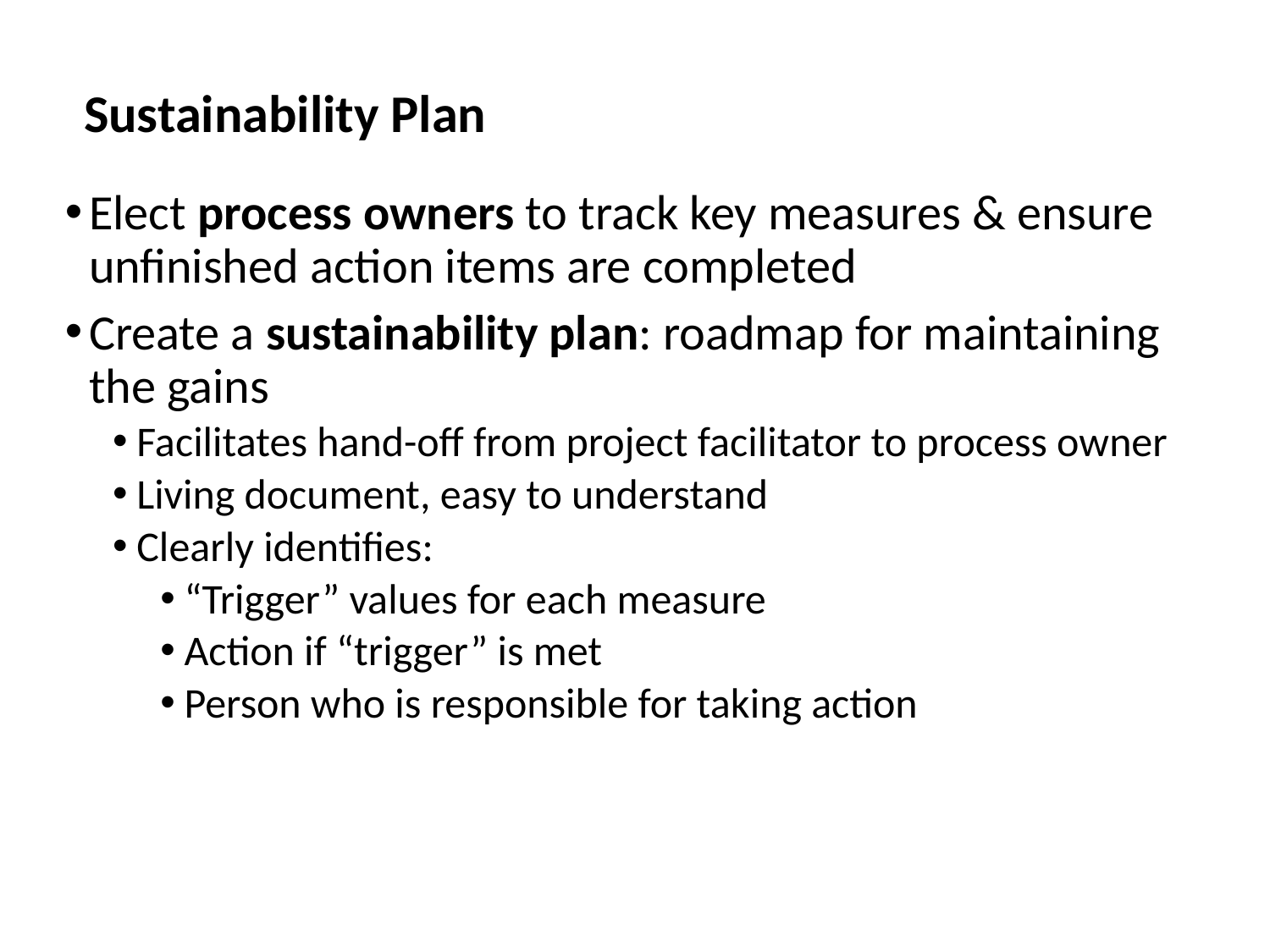

# Sustainability Plan
Elect process owners to track key measures & ensure unfinished action items are completed
Create a sustainability plan: roadmap for maintaining the gains
Facilitates hand-off from project facilitator to process owner
Living document, easy to understand
Clearly identifies:
“Trigger” values for each measure
Action if “trigger” is met
Person who is responsible for taking action

## Slide 15
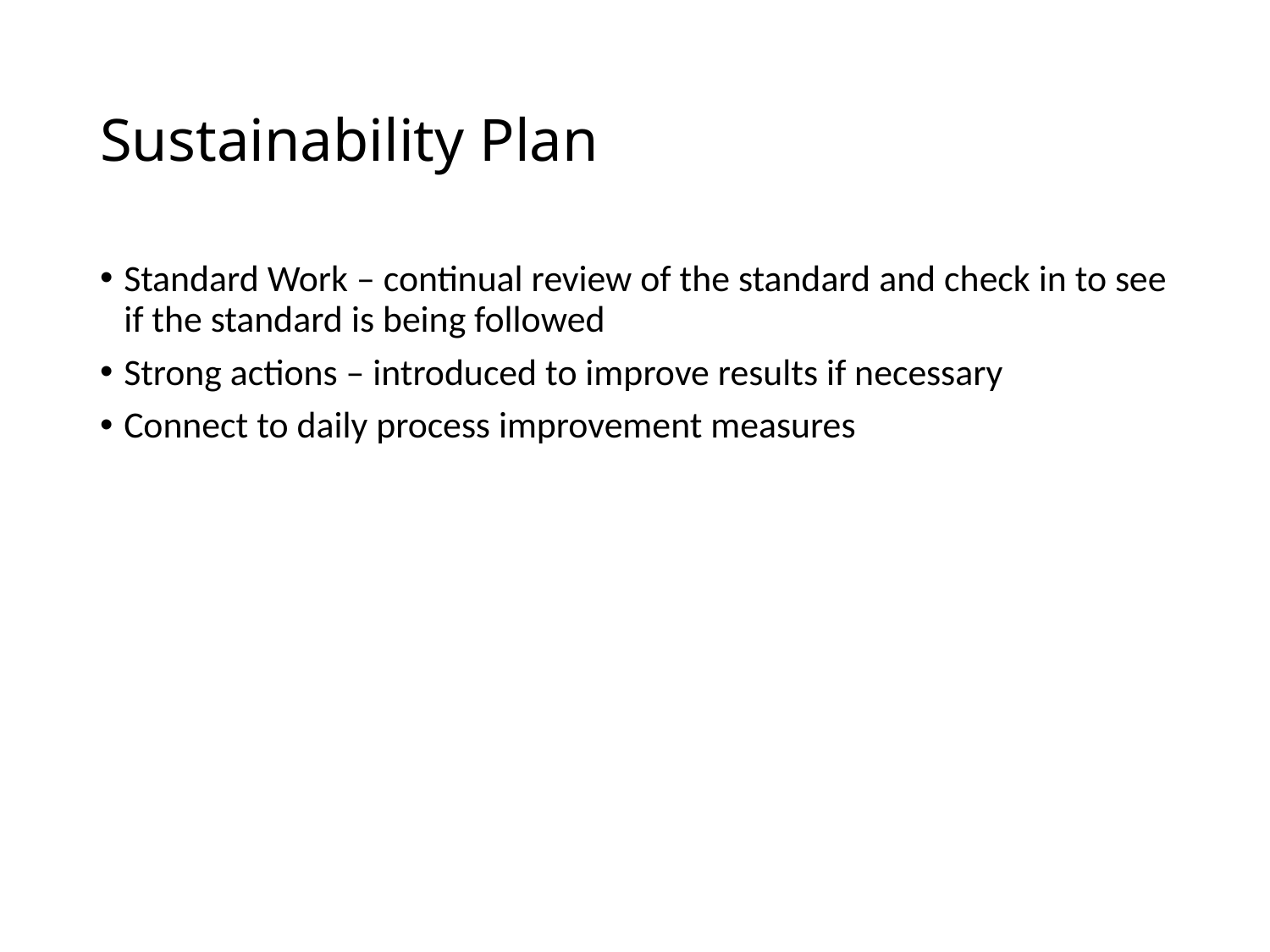

# Sustainability Plan
Standard Work – continual review of the standard and check in to see if the standard is being followed
Strong actions – introduced to improve results if necessary
Connect to daily process improvement measures

## Slide 16
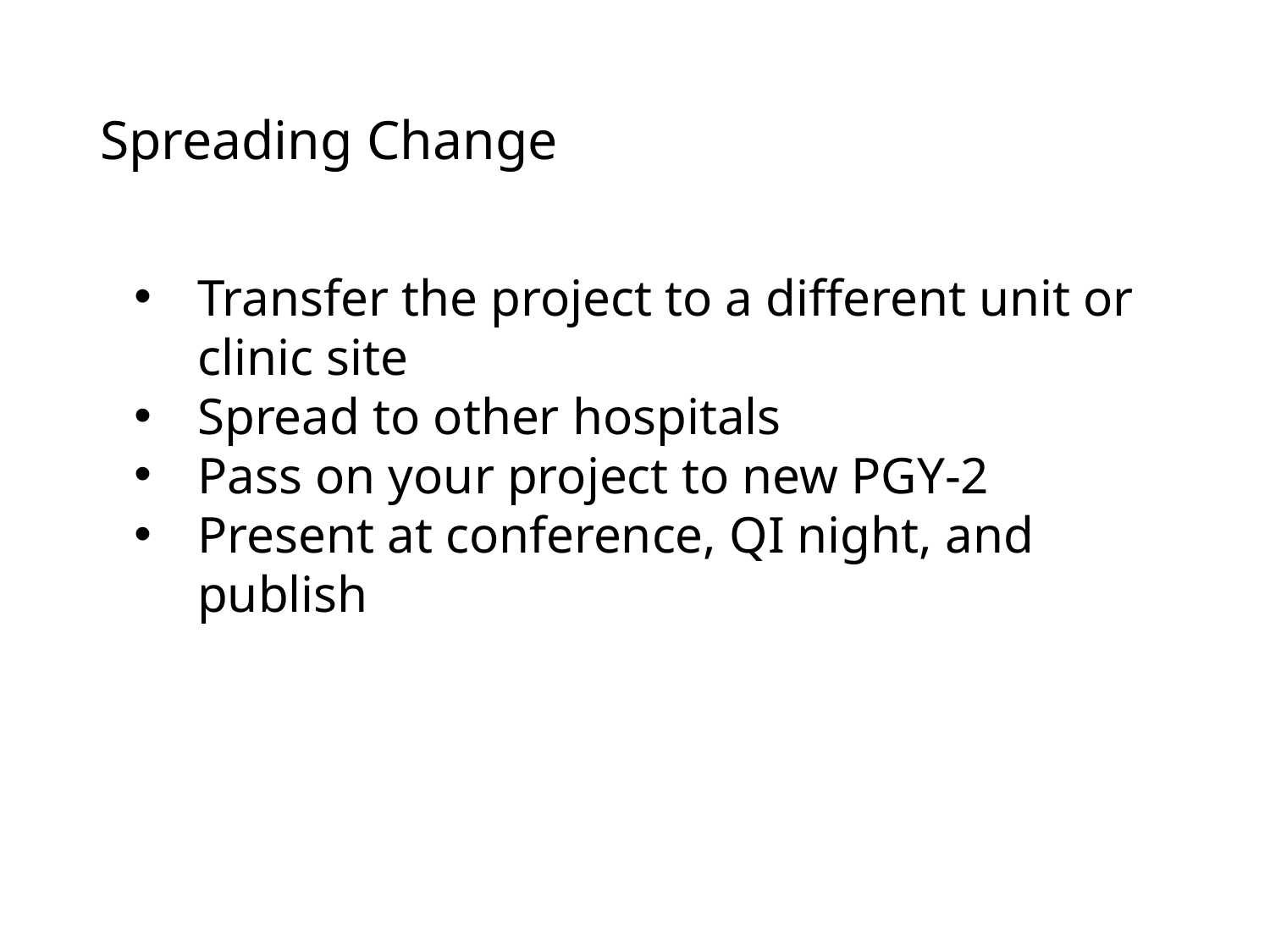

# Spreading Change
Transfer the project to a different unit or clinic site
Spread to other hospitals
Pass on your project to new PGY-2
Present at conference, QI night, and publish

## Slide 17
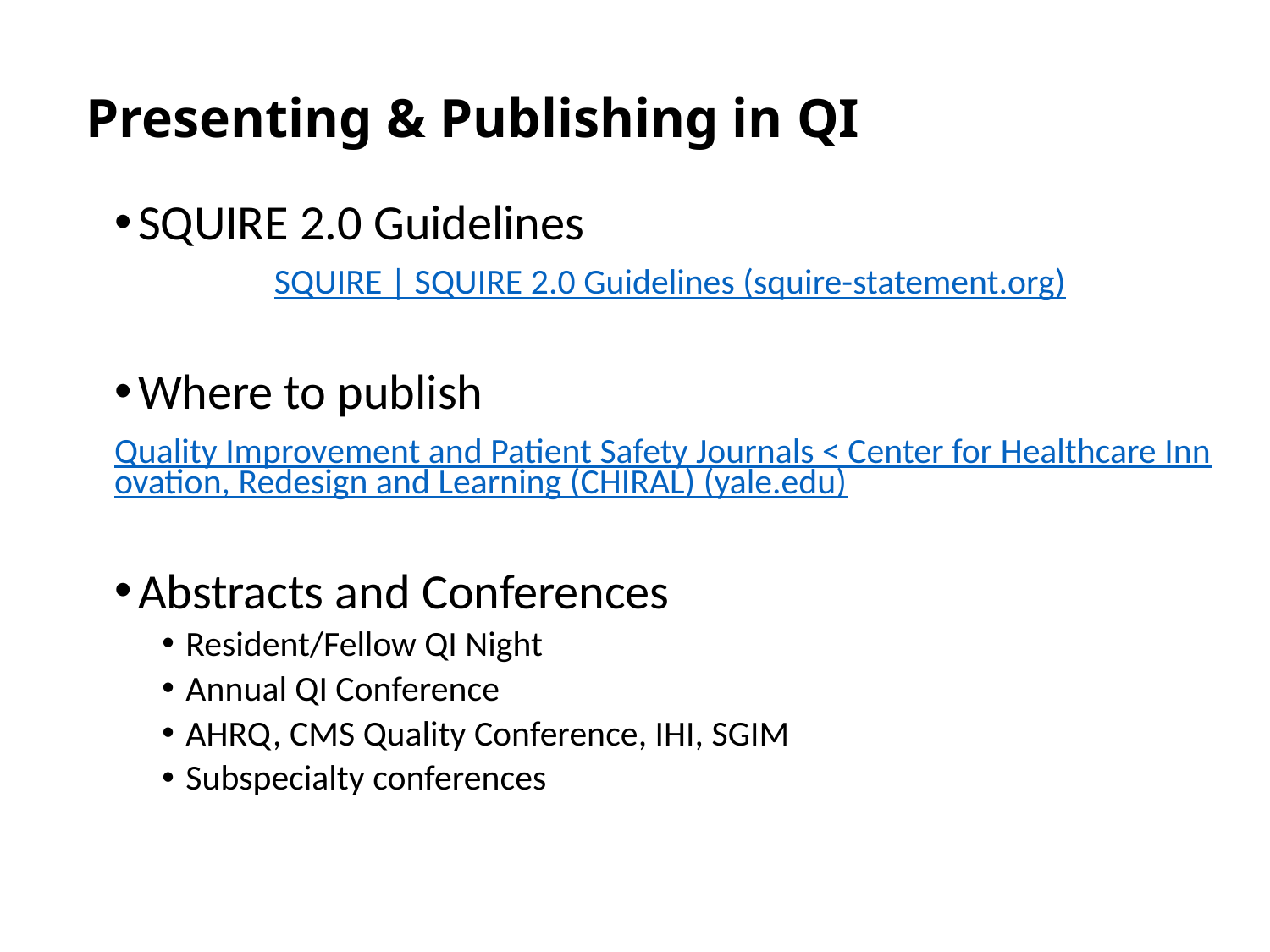

# Presenting & Publishing in QI
SQUIRE 2.0 Guidelines
SQUIRE | SQUIRE 2.0 Guidelines (squire-statement.org)
Where to publish
Quality Improvement and Patient Safety Journals < Center for Healthcare Innovation, Redesign and Learning (CHIRAL) (yale.edu)
Abstracts and Conferences
Resident/Fellow QI Night
Annual QI Conference
AHRQ, CMS Quality Conference, IHI, SGIM
Subspecialty conferences

## Slide 18
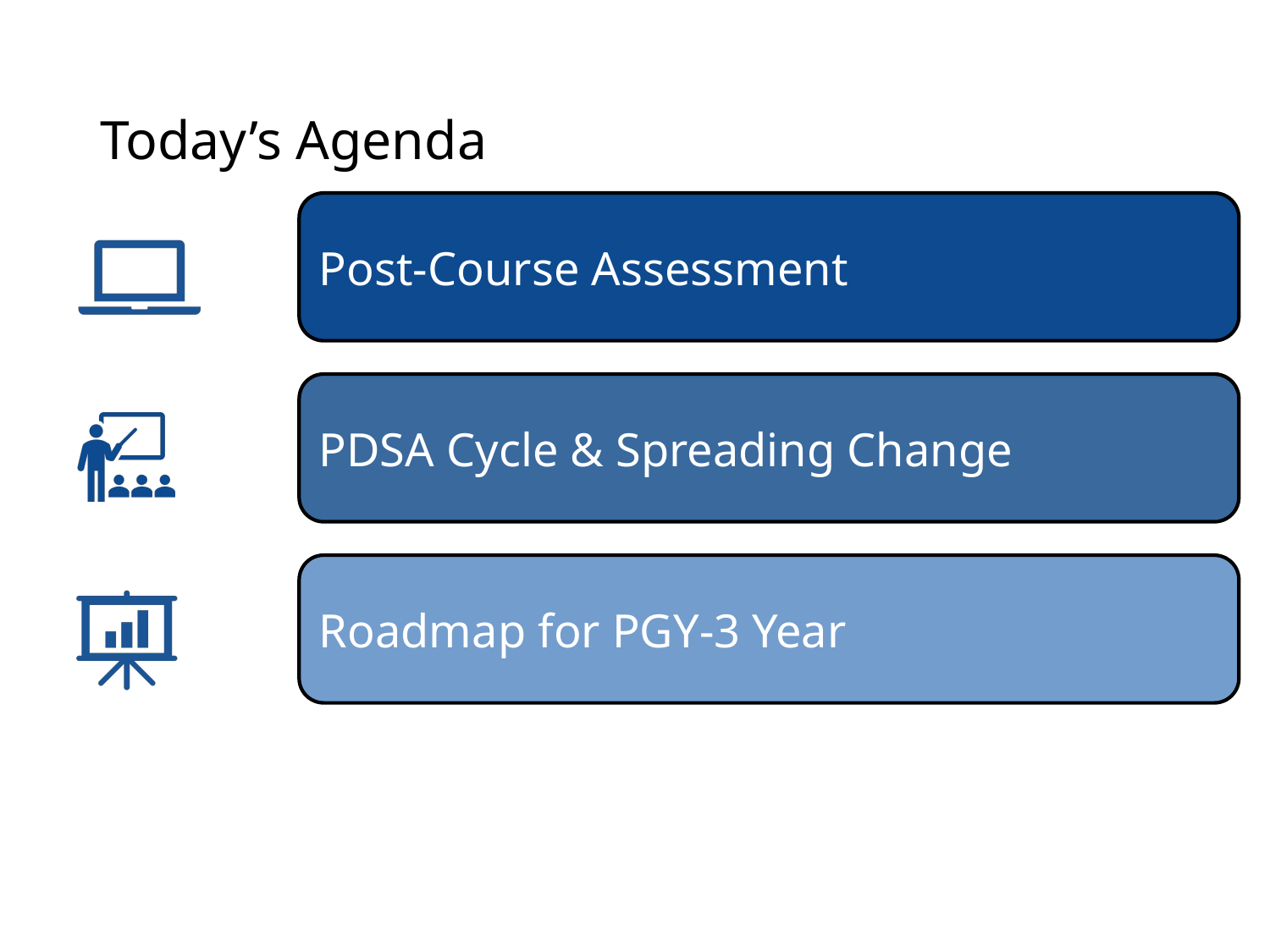

# Today’s Agenda
Post-Course Assessment
PDSA Cycle & Spreading Change
Roadmap for PGY-3 Year

## Slide 19
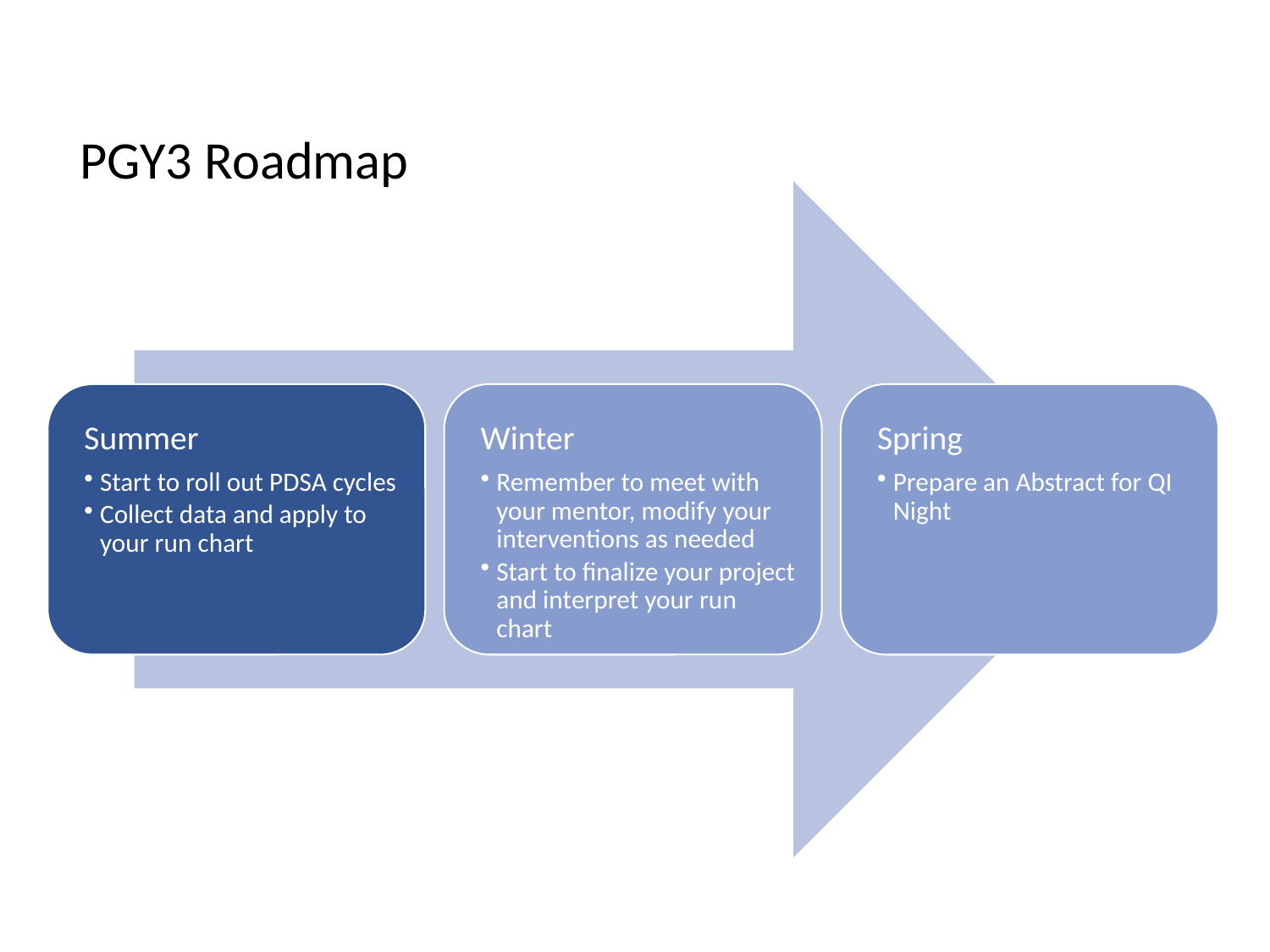

# PGY3 Roadmap

## Slide 20
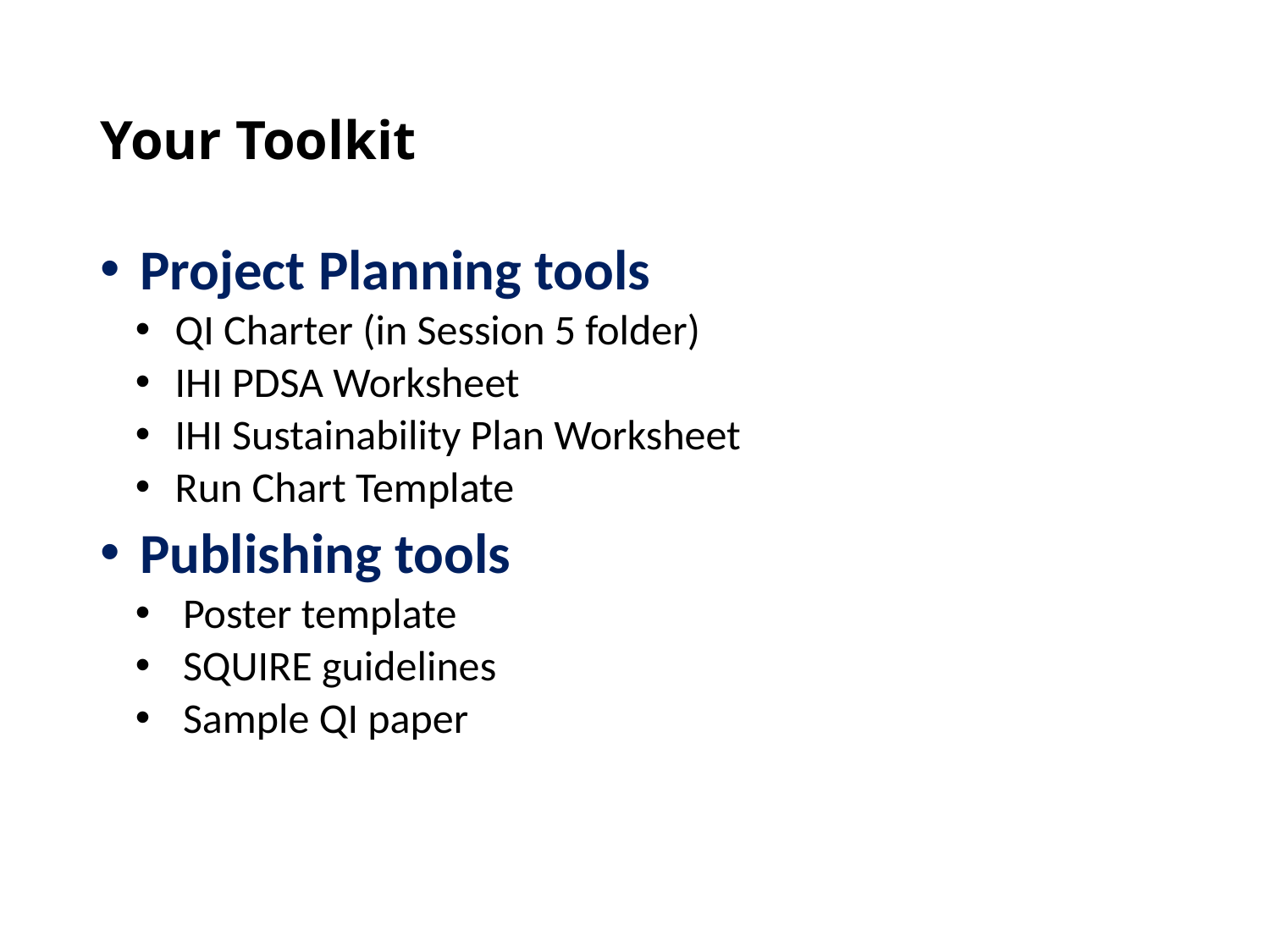

# Your Toolkit
Project Planning tools
QI Charter (in Session 5 folder)
IHI PDSA Worksheet
IHI Sustainability Plan Worksheet
Run Chart Template
Publishing tools
Poster template
SQUIRE guidelines
Sample QI paper

## Slide 21
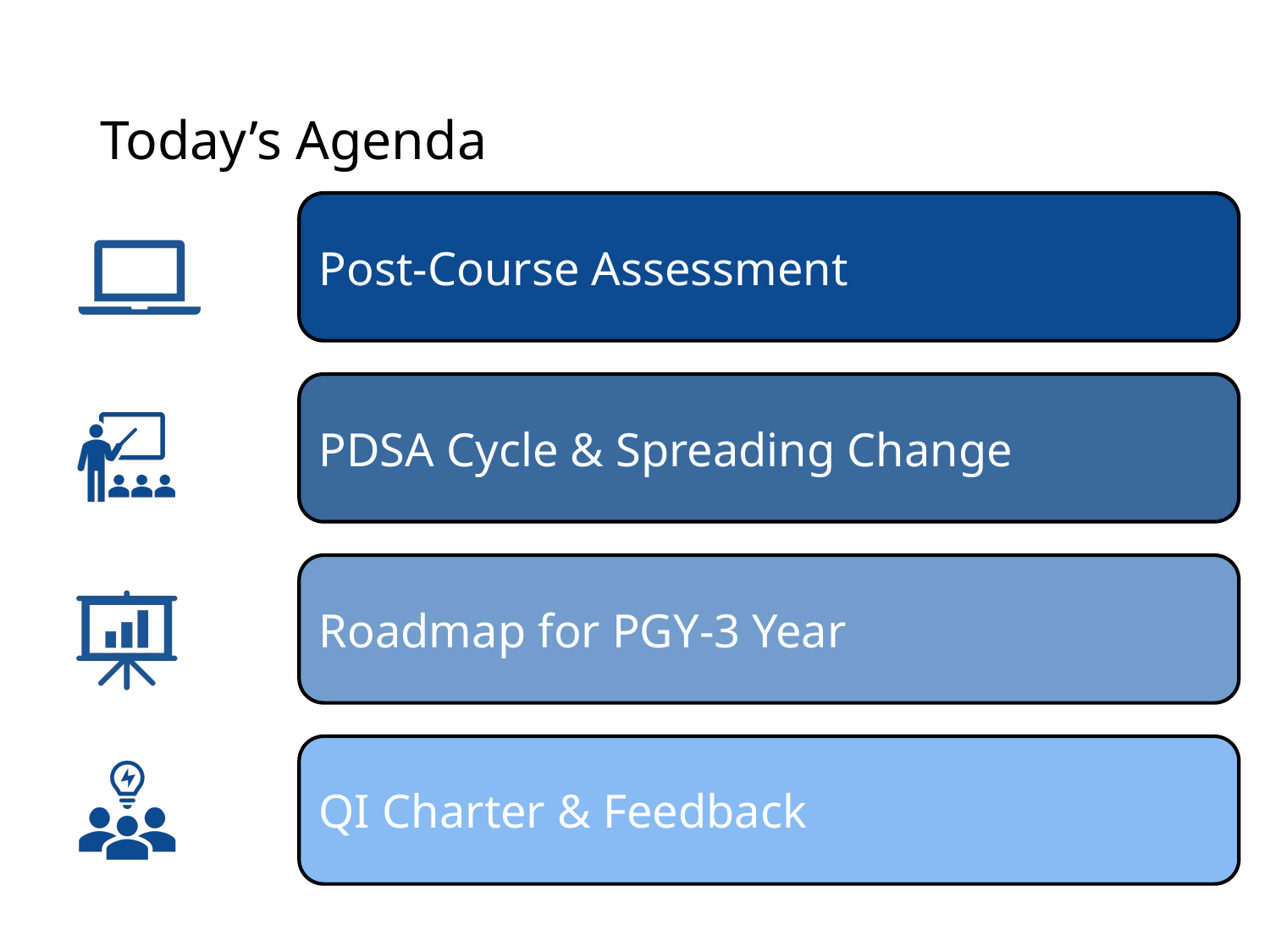

# Today’s Agenda
Post-Course Assessment
PDSA Cycle & Spreading Change
Roadmap for PGY-3 Year
QI Charter & Feedback
