# Supplementary material for: Building Resident Quality Improvement Knowledge and Engagement Through a Longitudinal, Mentored, and Experiential Learning-Based Quality Improvement Curriculum
Source: MedEdPORTAL. 2023 Apr 18;19:11310. doi: 10.15766/mep_2374-8265.11310 (PMC10110773; doi:10.15766/mep_2374-8265.11310)
Supplement: Supplementary file 1 — Session 1 Slides.pptxSession 1 Workbook.pptxSession 2 Slides.pptxSession 2 Workbook.pptxSession 3 Slides.pptxSession 4 Work-in-Progress Presentation Template.pptxSession 5 Slides.pptxQI Charter Template.docxFaculty Milestones.docxFaculty Guide.docxResident Survey.docx [file mep_2374-8265.11310-s001.zip › H. QI Charter Template.docx]

**QI Project Charter**

*Adapted from IHI Tool: Quality Improvement Project Charter 2018*

**Project Title**:

**Team members**:

**Mentor**:

**Date**:

1. **What are we trying to accomplish?**

*Document your current thinking about the activities of the project (e.g., design a new process, improve an existing product or service, etc.). Note the subsystem, pilot population, and/or demonstration unit where the work will take place. Consider including your long-term vision and short-term project goals.*

Problem Statement:

Insert your problem statement from your work-in-progress presentation here.

Rationale (defines why)

*Explain why the current process or system needs improvement. If possible, include baseline data and other benchmarks.*

Expected Outcomes and Benefits

*How will this improvement benefit the team, the organization, customers, and/or the community? Is there potential for a business impact, such as reduced costs or other financial benefits?*

Aim Statement

*What outcome are you hoping to achieve? Specify how good, for whom, and by when (a specific date).* Insert your aim statement from your work-in-progress presentation here.

1. **How will we know that a change is an improvement?**

*When defining your project-level measures, provide operational definitions, which specify unambiguously how to derive each measure, and be sure to define numerators and denominators in measures such as percent or rates.*

Outcome Measure(s) *List the measure(s) you ultimately want to affect as a result of this project.*

Process Measure(s) *List the measure(s) that will tell you if the system is performing as planned to affect the outcome measure.*

Balancing Measure(s) *List the measures that will tell you whether you are introducing problems elsewhere in the system.*

You could insert your data collection plan here.

1. **What changes can we make that will result in improvement?**

*Start by exploring the process or system you are trying to improve with tools such as interviews, direct observation, fishbone (cause and effect) diagrams, driver diagrams, and process maps/flowcharts.*

You could insert your process map and fishbone diagram here.

Intervention/Change Ideas

*What ideas do you have for initial tests of change (Plan-Do-Study-Act cycles)?*

*Who are the key Stakeholders whose input and support will this project require? How will you engage these key stakeholders?*

You could insert your Impact/Effort Grid here.

Barriers

*What barriers do you predict to your success? How will you overcome these barriers?*

Boundaries

*List any guidelines for the team, including project constraints, rules or procedures, technology considerations, what is out of scope, etc.*
