# Supplementary material for: Building Resident Quality Improvement Knowledge and Engagement Through a Longitudinal, Mentored, and Experiential Learning-Based Quality Improvement Curriculum
Source: MedEdPORTAL. 2023 Apr 18;19:11310. doi: 10.15766/mep_2374-8265.11310 (PMC10110773; doi:10.15766/mep_2374-8265.11310)
Supplement: Supplementary file 1 — Session 1 Slides.pptxSession 1 Workbook.pptxSession 2 Slides.pptxSession 2 Workbook.pptxSession 3 Slides.pptxSession 4 Work-in-Progress Presentation Template.pptxSession 5 Slides.pptxQI Charter Template.docxFaculty Milestones.docxFaculty Guide.docxResident Survey.docx [file mep_2374-8265.11310-s001.zip › I. Faculty Milestones.docx]

**Resident QI Project Expectations**

**Expectation: residents will check in with faculty mentor at least once every 6-8 weeks**

**Summer/Fall**

- **Resident goals**
  - Brainstorm project ideas
  - Draft problem statement
  - Draft process map and fishbone diagram
  - Draft aim statement
  - Meet with mentor
- **Mentor goals: guide project scope / feasibility**
  - *Please help residents select a project that is doable within the time constraints of residency (in particular inpatient responsibilities). They are expected to work on their QI projects mostly during their ambulatory blocks (2 out of every 8 weeks)*
  - Provide institutional knowledge / subject expertise on project idea
  - Identify and connect additional stakeholders
  - Review problem statement, process map, fishbone diagram, aim statement and provide guidance
  - +/- Provide institutional knowledge /subject expertise on suggested interventions and feasibility

**Winter**

- **Resident goals**
  - Understanding data from a QI perspective
  - Formalize data collection plan using template
  - Brainstorm intervention ideas / create impact/effort grid
  - Meet with mentor
- **Mentor goals: guide data collection and developing intervention ideas**
  - Provide knowledge of pre-existing databases or how to obtain relevant data
  - Provide institutional knowledge /subject expertise on suggested interventions and feasibility
  - Identify and support discussions with additional stakeholders re: interventions (“clear institutional roadblocks”)
  - Review data collection template and impact/effort grid and provide guidance

**Late Winter/Early Spring**

- **Resident goals**
  - Present project idea at Work-in-Progress presentation (residents will "pitch" QI project to QI experts
  - Meet with mentor
- **Mentor goals: preparing for Work-in-Progress presentations**
  - Review resident Work-in-Progress presentation and provide feedback
  - Attend Work-in-Progress *if time permits*

**Spring**

- **Resident goals**
  - Learn how to implement and spread change
  - Review and incorporate feedback from Work-in-Progress
  - Plan and implement PDSA cycle #1 (expectation: keeping scope as small as possible)
  - Complete QI charter
  - Meet with mentor
- **Mentor goals: implementing PDSA #1**
  - Review and incorporate feedback from Work-in-Progress
  - Provide institutional support of implementation PDSA cycle #1
  - Review QI charter and assess project launch readiness, provide guidance as needed
