# Supplementary material for: Building Resident Quality Improvement Knowledge and Engagement Through a Longitudinal, Mentored, and Experiential Learning-Based Quality Improvement Curriculum
Source: MedEdPORTAL. 2023 Apr 18;19:11310. doi: 10.15766/mep_2374-8265.11310 (PMC10110773; doi:10.15766/mep_2374-8265.11310)
Supplement: Supplementary file 1 — Session 1 Slides.pptxSession 1 Workbook.pptxSession 2 Slides.pptxSession 2 Workbook.pptxSession 3 Slides.pptxSession 4 Work-in-Progress Presentation Template.pptxSession 5 Slides.pptxQI Charter Template.docxFaculty Milestones.docxFaculty Guide.docxResident Survey.docx [file mep_2374-8265.11310-s001.zip › J. Faculty Guide.docx]

**Faculty Guide**

**QI Session 1: Introduction to QI**

Teaching Objectives:

- Provide a brief overview of the various QI methods, with focus on IHI model
- Discuss how to approach a problem
  - Identify stakeholders
  - Identify the problem/process: process map and fish bone diagram
  - Write a Problem statement
  - Fill out an impact/effort grid
- Review how to choose a QI project & set expectations

Team Goals:

- Create QI team
- Identify a pain point
- Identify possible mentors

Timeline

- **Pre-session**
  - ask residents to identify “pain points” in their clinical training
  - Complete QIKAT-R
- **9:00-9:15 AM**: Introduction into QI Curriculum and IHI Model of Improvement
- **9:15-11:00AM**: Practice using tools for understanding the problem
  - Interviewing Stakeholders
  - Creating a Process Map
  - Evaluating root causes using a Fishbone Diagram
  - Practice drafting a Problem Statement
  - Create an impact/effort grid
- **11:00AM-12:00PM**: time to choose your own QI project
  - Brainstorm project ideas and form your QI team
- **Post session**
  - Ask residents to form a QI team, brainstorm project ideas, and identify and meet with a potential mentor by the next session

**QI Session 2: Developing an Aim and Choosing Interventions**

Teaching Objectives:

- Teach the components of a comprehensive problem statement
- Discuss the components of an aim Statement
  - Teach various methods for choosing interventions (IHI Change Concepts, Action Hierarchy, Impact/Effort Grid, review process map or root cause diagram)
- Provide teams time to work on their projects, introduced to Resident Workbook
- Introduce concept of obtaining data &IRB exemptions

Team Goals: introduce to resident workbook

- draft problem statement
- draft process map and fishbone diagram
- draft aim statement
- think about how to obtain data targeting aim statement

Timeline

- **Pre-session**
  - Check in to see if residents met with their identified mentor
  - Ask the residents to start thinking about potential stakeholders, performing a process walk and other information necessary to complete a process map and fishbone diagram
- **9:00-9:15AM:** Recap of session 1 and intro to aim statements
- **9:15-10:15AM:** Divide into QI teams to complete the Resident Workbook. The workbook will guide QI teams to complete the following tasks:
  - Write a problem statement
  - Make a process map
  - Make a fishbone diagram
  - Write an aim statement
- **10:15-10:45AM:** Each group presents their workbook + feedback from other groups
- **10:45-11:15AM:** Intro to choosing an intervention
  - Brainstorm interventions, rank interventions via impact/effort grid, action hierarchy
  - Assign interventions to impact/effort grid
- **11:15AM-12PM**: project work time
- **Post-session**
  - Ask the residents to complete before the next session:
    - address all the questions on the orange slides in the workbook and revise/edit their workbook up to slide 16 (problem statement, process map, fishbone diagram, aim statement)
      - Identify if they need to talk to stakeholders to help with above
      - Meet with their mentor to review problem statement, process map, fishbone diagram, aim statement
    - Start brainstorm potential interventions and send the chief resident their ideas

**QI Session 3: Measuring Change**

Teaching Objectives:

- Review the differences between research and QI
- Discuss QI based data collection
- Teach the difference between outcome, process, and balancing measures
- Provide an overview on forming and interpreting run charts (understand variation, types of sampling) and pareto charts

Team Goals:

- introduce to work-in-progress presentation template
- Continue to work on resident workbook with intervention ideas, impact/effort grid, and action hierarchy
- Formalize data collection plan
- Brainstorm intervention ideas

Timeline

- **Pre-session**
  - Check in with residents regarding their progress on their workbook and potential intervention ideas
- **9:00-9:30AM:** Review Session 2 content
- **9:30-10:15AM**: Types of Measures
  - CMS data exercise
- **10:15-11:00AM**: Forming and Interpreting Run Charts
- **11:00-12:00PM:** prepare Work-in-Progress presentation & project work time
- **Post-session**
  - Ask residents to complete up to slide 22 in their workbook (intervention ideas, impact/effort grid, and action hierarchy)
  - Develop Work-in-Progress presentations and start thinking about data collection plan
  - Residents should check in with mentor at least once before the next session regarding project progress

**QI Session 4: Work-in-Progress presentations**

Teaching Objectives

- Present ongoing QI projects and receive feedback from clinical and QI experts

Team Goals

- Present project to clinical leadership for feedback

Timeline

- **Pre-session**
  - Reach out to potential clinical and QI leaders at the institution several months in advance for their availabilities
- **9:00-11:00AM**: Each group presents their Work-in-Progress project for 10-15 minutes and receive feedback
- **11:00-12:00PM:** project work time
- **Post-session**
  - Summarize and send feedback to each project from the clinical and QI leaders
  - Ask residents to plan or roll out their first intervention cycle
  - Residents should check in with mentor at least once before the next session regarding project progress

**QI Session 5: The PDSA Cycle and Spreading Change**

Teaching Objectives:

- Review the fundamentals of the PDSA cycles and the concept of starting small and scaling up
- Review principles of sustainability
- Discuss the principles and psychology of spreading change
- Provide summary for future steps: QI charter, publication forums, etc

Team Goals:

- Review and incorporate feedback from Work-in-Progress session
- Complete any necessary IRB proposals if necessary
- Plan and implement PDSA #1
- Complete QI Charter

Timeline

- **Pre-session**
  - Check in with residents regarding intervention ideas and progress for the first intervention cycle
- **9:00-9:15 AM:** complete QIKAT-R
- **9:15-10:00 AM:** Discuss PDSA cycles and spreading change
- **10:00-10:30AM:** Preparing for PGY-3 year
- **10:30-12:00PM**: project work time & complete QI charter
- **Post-session**
  - Implement PDSA cycle #1

Additional Q&A:

**Who are the learners?**

In this curriculum, our trainees were PGY2 internal medicine residents, however this curriculum is applicable to residents, fellows, and medical students. We recommend offering this curriculum early during their training for maximum project work time and application of QI concepts.

While the examples provided in this curriculum are geared towards internal medicine residents, the examples can be adapted to other specialties as well. For example, in session 1’s “critical results after hours” example, the critical result can be changed to critical issues for different specialties or any other situations where a clinician is balancing multiple critical responsibilities. For example, the scenario can be changed to a surgical resident addressing a critical imaging result for an outpatient post-op patient while in the operating room. Alternatively, the scenario can be adapted to a consulting fellow covering a busy inpatient service who receives a call about a critical result.

**When will the curriculum be delivered and when will the participants work on their QI projects?**

For our curriculum, our five sessions were delivered during one half day session every ambulatory clinic block, which occurred once every eight weeks. While the curriculum can be delivered in a shorter period of time, having the teaching spread over the time it typically takes to complete a QI project will ensure teaching, mentorship and oversight to run in parallel. We recommend that QI project work is structured at regular intervals within the existing education time, which will allow dedicated time for residents to be fully engaged in QI work.

The slides in appendix A, C, E, G contain the content that was used in our curriculum, with additional notes that provide more detail and talking points for the slide deck.

**Who will be teaching?**

Decide who will teach and who will be back up teachers. In our case, we found success in having a dedicated chief resident with protected teaching time. Curricular oversight was provided by a QI lead faculty while other faculty members supported sessions as needed. In the absence of a chief resident, a dedicated QI faculty member/members with protected time to deliver and oversee the curriculum can be considered.

**How are faculty mentors recruited?**

An email is sent out to the entire department at the beginning of the academic year prior to session 1 to solicit faculty who are interested in serving as QI mentors and/or have ongoing QI projects. Interested faculty, their projects, and prior QI and mentorship experiences are compiled in a list that is shared with the residents during session 1 and 2.

Develop a project mentor guide (see appendix I) and communicate with all the newly enrolled faculty mentors to ensure understanding and provide any clarifications as needed.

**How are resident QI projects developed?**

The residents should share their project ideas and faculty mentor with the chief resident (faculty QI education lead) by session 2. If a resident group has a project idea but no faculty mentor, the chief resident can provide suggestions and connect residents to faculty mentors in their area of interest based on their institutional experiences and relationships.

Develop QI project milestones (ie work-in-progress presentations, QI charter, workbooks) and share with residents. Communicate with the residents a recommended check-in plan with their project mentor and chief resident usually every 6-8 weeks.

**How will QI knowledge be measured?**

Administer a validated assessment tool such as QIKAT-R at the start and end of the curriculum to the residents. Other ways to measure curriculum success include tracking the number of QI projects adopted by the institution, ad hoc surveys of learner attitudes and understanding, number of QI projects presented at various venues, publications, and more.

**What are other tips for the curriculum?**

- Establish a clear communication plan with the residents in terms of curriculum and project expectations. Set clear timelines.
- Optional:
  - Plan a session where residents get to pitch their QI project ideas to a group of QI experts just before they roll out their first PDSA cycle so that the residents can receive feedback and institutional support
  - Celebrate the resident success by providing an end of the year platform to showcase their work to the department, leadership, etc
