# Supplementary material for: Building Resident Quality Improvement Knowledge and Engagement Through a Longitudinal, Mentored, and Experiential Learning-Based Quality Improvement Curriculum
Source: MedEdPORTAL. 2023 Apr 18;19:11310. doi: 10.15766/mep_2374-8265.11310 (PMC10110773; doi:10.15766/mep_2374-8265.11310)
Supplement: Supplementary file 1 — Session 1 Slides.pptxSession 1 Workbook.pptxSession 2 Slides.pptxSession 2 Workbook.pptxSession 3 Slides.pptxSession 4 Work-in-Progress Presentation Template.pptxSession 5 Slides.pptxQI Charter Template.docxFaculty Milestones.docxFaculty Guide.docxResident Survey.docx [file mep_2374-8265.11310-s001.zip › K. Resident Survey.docx]

**Resident Feedback Survey**

What is your overall rating of the QI curriculum?

- Poor
- Fair
- Good
- Very Good
- Excellent

I understand how to make an effective problem statement

- Strongly disagree
- Disagree
- Neither agree nor disagree
- Somewhat agree
- Strongly agree

I understand how to write an effective aim statement

- Strongly disagree
- Disagree
- Neither agree nor disagree
- Somewhat agree
- Strongly agree

I understand how to identify stakeholders

- Strongly disagree
- Disagree
- Neither agree nor disagree
- Somewhat agree
- Strongly agree

I can use a fishbone diagram to understand a problem

- Strongly disagree
- Disagree
- Neither agree nor disagree
- Somewhat agree
- Strongly agree

I can use a process map to understand a problem

- Strongly disagree
- Disagree
- Neither agree nor disagree
- Somewhat agree
- Strongly agree

I understand how to identify possible interventions

- Strongly disagree
- Disagree
- Neither agree nor disagree
- Somewhat agree
- Strongly agree
