# Supplementary material for: Building Resident Quality Improvement Knowledge and Engagement Through a Longitudinal, Mentored, and Experiential Learning-Based Quality Improvement Curriculum
Source: MedEdPORTAL. 2023 Apr 18;19:11310. doi: 10.15766/mep_2374-8265.11310 (PMC10110773; doi:10.15766/mep_2374-8265.11310)
Supplement: Supplementary file 1 — Session 1 Slides.pptxSession 1 Workbook.pptxSession 2 Slides.pptxSession 2 Workbook.pptxSession 3 Slides.pptxSession 4 Work-in-Progress Presentation Template.pptxSession 5 Slides.pptxQI Charter Template.docxFaculty Milestones.docxFaculty Guide.docxResident Survey.docx [file mep_2374-8265.11310-s001.zip › B. Session 1 Workbook.pptx]

## Slide 1
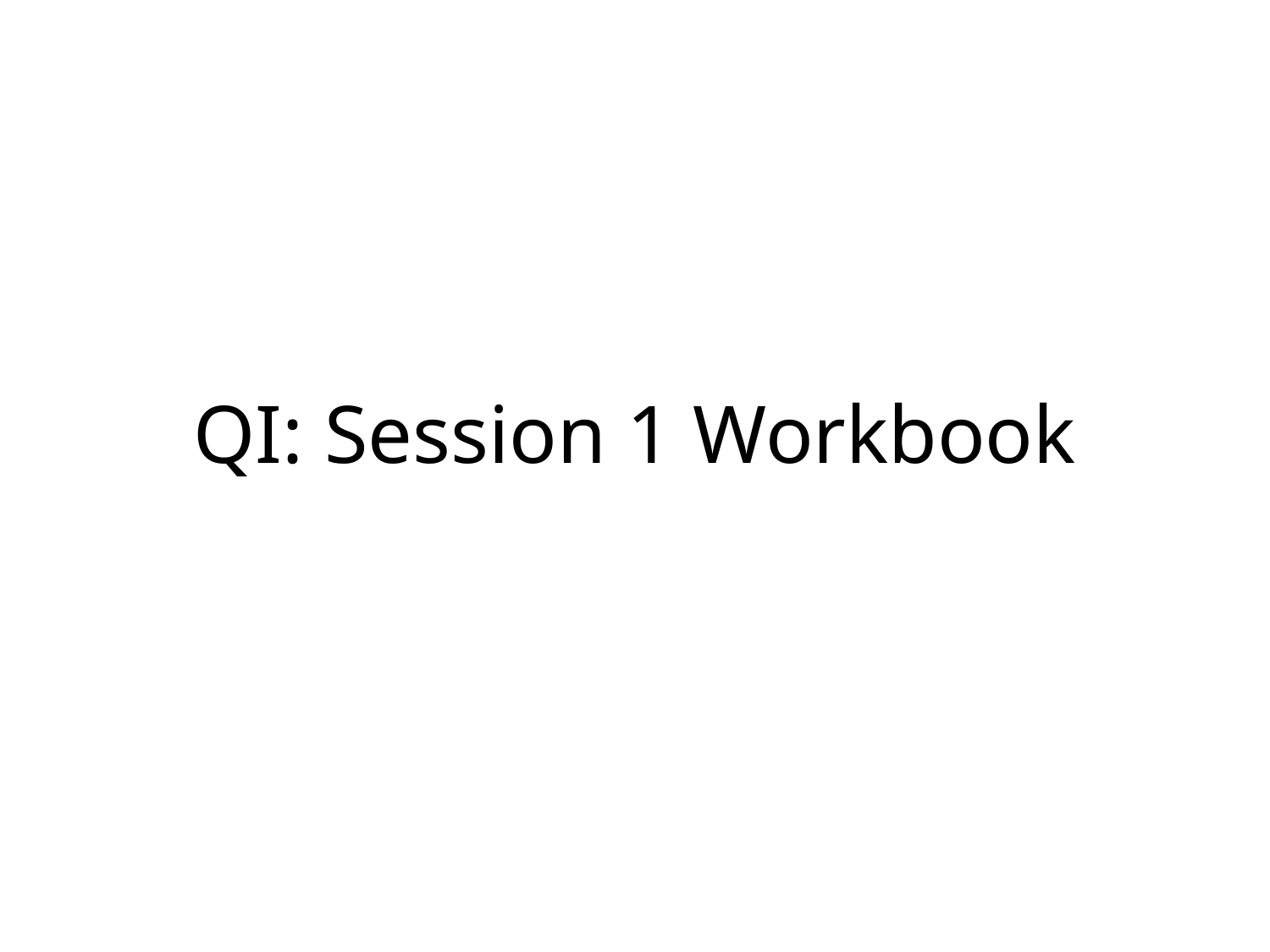

# QI: Session 1 Workbook

## Slide 2
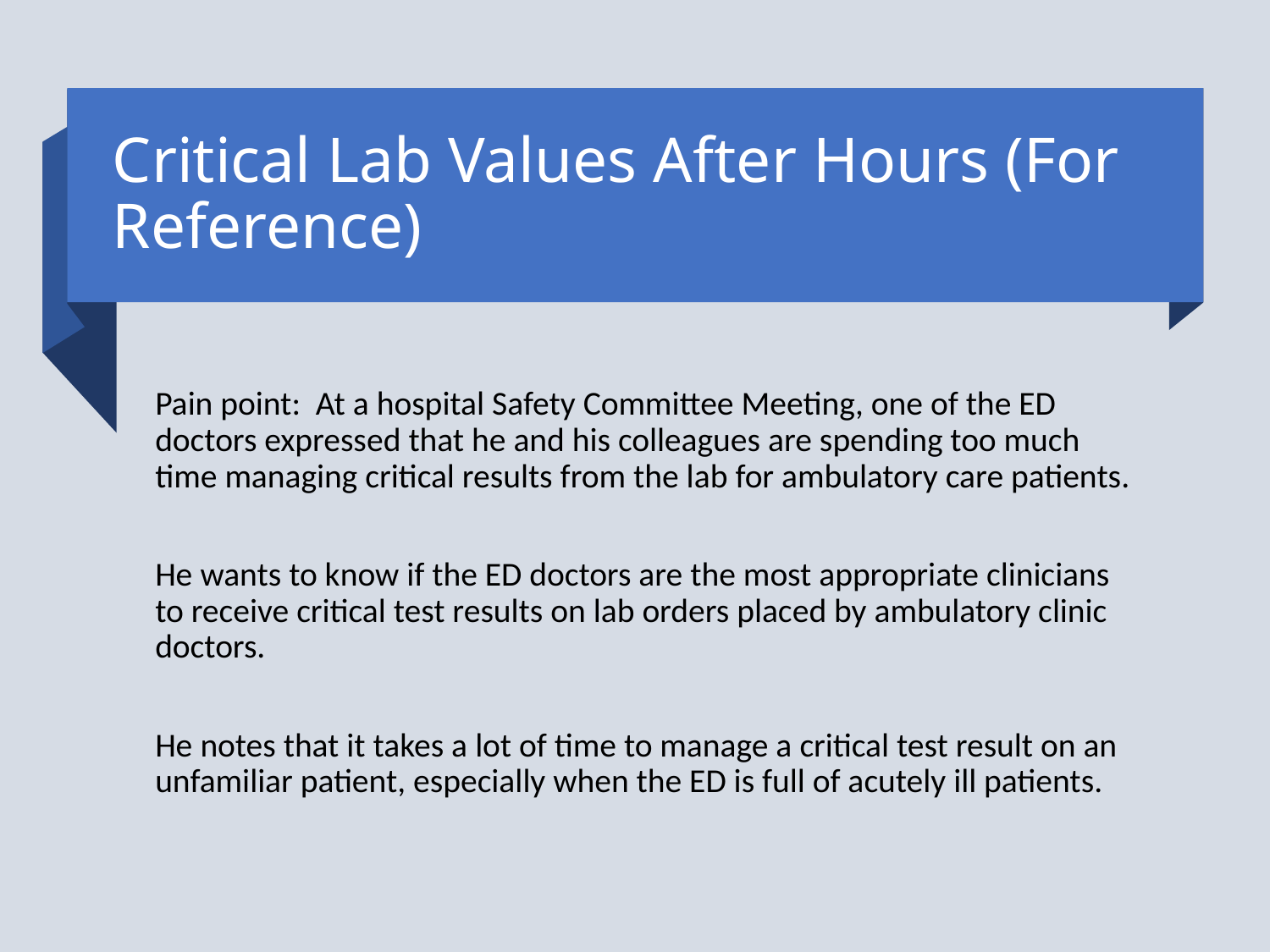

# Critical Lab Values After Hours (For Reference)
Pain point: At a hospital Safety Committee Meeting, one of the ED doctors expressed that he and his colleagues are spending too much time managing critical results from the lab for ambulatory care patients.
He wants to know if the ED doctors are the most appropriate clinicians to receive critical test results on lab orders placed by ambulatory clinic doctors.
He notes that it takes a lot of time to manage a critical test result on an unfamiliar patient, especially when the ED is full of acutely ill patients.

## Slide 3
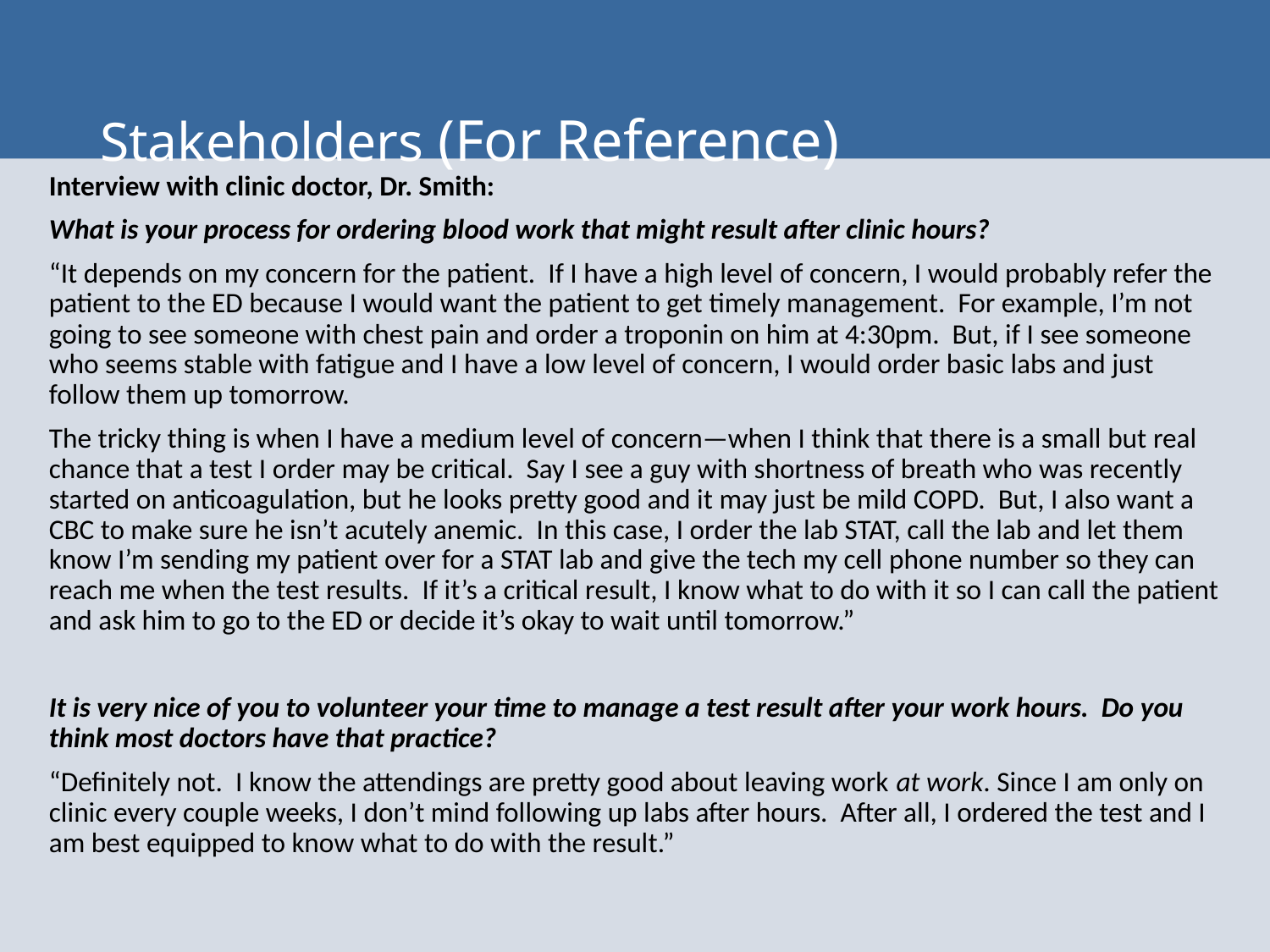

# Stakeholders (For Reference)
Interview with clinic doctor, Dr. Smith:
What is your process for ordering blood work that might result after clinic hours?
“It depends on my concern for the patient. If I have a high level of concern, I would probably refer the patient to the ED because I would want the patient to get timely management. For example, I’m not going to see someone with chest pain and order a troponin on him at 4:30pm. But, if I see someone who seems stable with fatigue and I have a low level of concern, I would order basic labs and just follow them up tomorrow.
The tricky thing is when I have a medium level of concern—when I think that there is a small but real chance that a test I order may be critical. Say I see a guy with shortness of breath who was recently started on anticoagulation, but he looks pretty good and it may just be mild COPD. But, I also want a CBC to make sure he isn’t acutely anemic. In this case, I order the lab STAT, call the lab and let them know I’m sending my patient over for a STAT lab and give the tech my cell phone number so they can reach me when the test results. If it’s a critical result, I know what to do with it so I can call the patient and ask him to go to the ED or decide it’s okay to wait until tomorrow.”
It is very nice of you to volunteer your time to manage a test result after your work hours. Do you think most doctors have that practice?
“Definitely not. I know the attendings are pretty good about leaving work at work. Since I am only on clinic every couple weeks, I don’t mind following up labs after hours. After all, I ordered the test and I am best equipped to know what to do with the result.”

## Slide 4
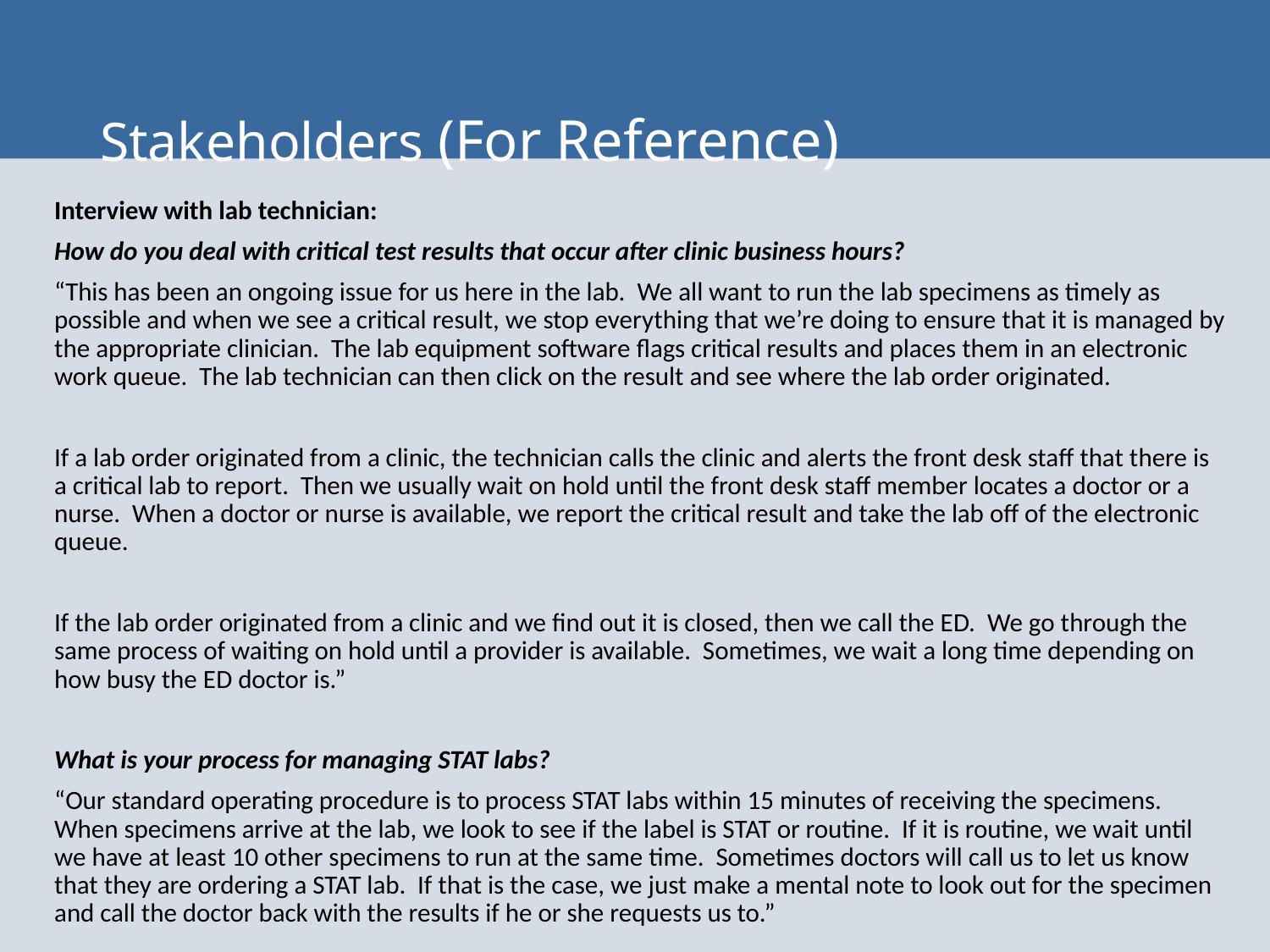

# Stakeholders (For Reference)
Interview with lab technician:
How do you deal with critical test results that occur after clinic business hours?
“This has been an ongoing issue for us here in the lab. We all want to run the lab specimens as timely as possible and when we see a critical result, we stop everything that we’re doing to ensure that it is managed by the appropriate clinician. The lab equipment software flags critical results and places them in an electronic work queue. The lab technician can then click on the result and see where the lab order originated.
If a lab order originated from a clinic, the technician calls the clinic and alerts the front desk staff that there is a critical lab to report. Then we usually wait on hold until the front desk staff member locates a doctor or a nurse. When a doctor or nurse is available, we report the critical result and take the lab off of the electronic queue.
If the lab order originated from a clinic and we find out it is closed, then we call the ED. We go through the same process of waiting on hold until a provider is available. Sometimes, we wait a long time depending on how busy the ED doctor is.”
What is your process for managing STAT labs?
“Our standard operating procedure is to process STAT labs within 15 minutes of receiving the specimens. When specimens arrive at the lab, we look to see if the label is STAT or routine. If it is routine, we wait until we have at least 10 other specimens to run at the same time. Sometimes doctors will call us to let us know that they are ordering a STAT lab. If that is the case, we just make a mental note to look out for the specimen and call the doctor back with the results if he or she requests us to.”

## Slide 5
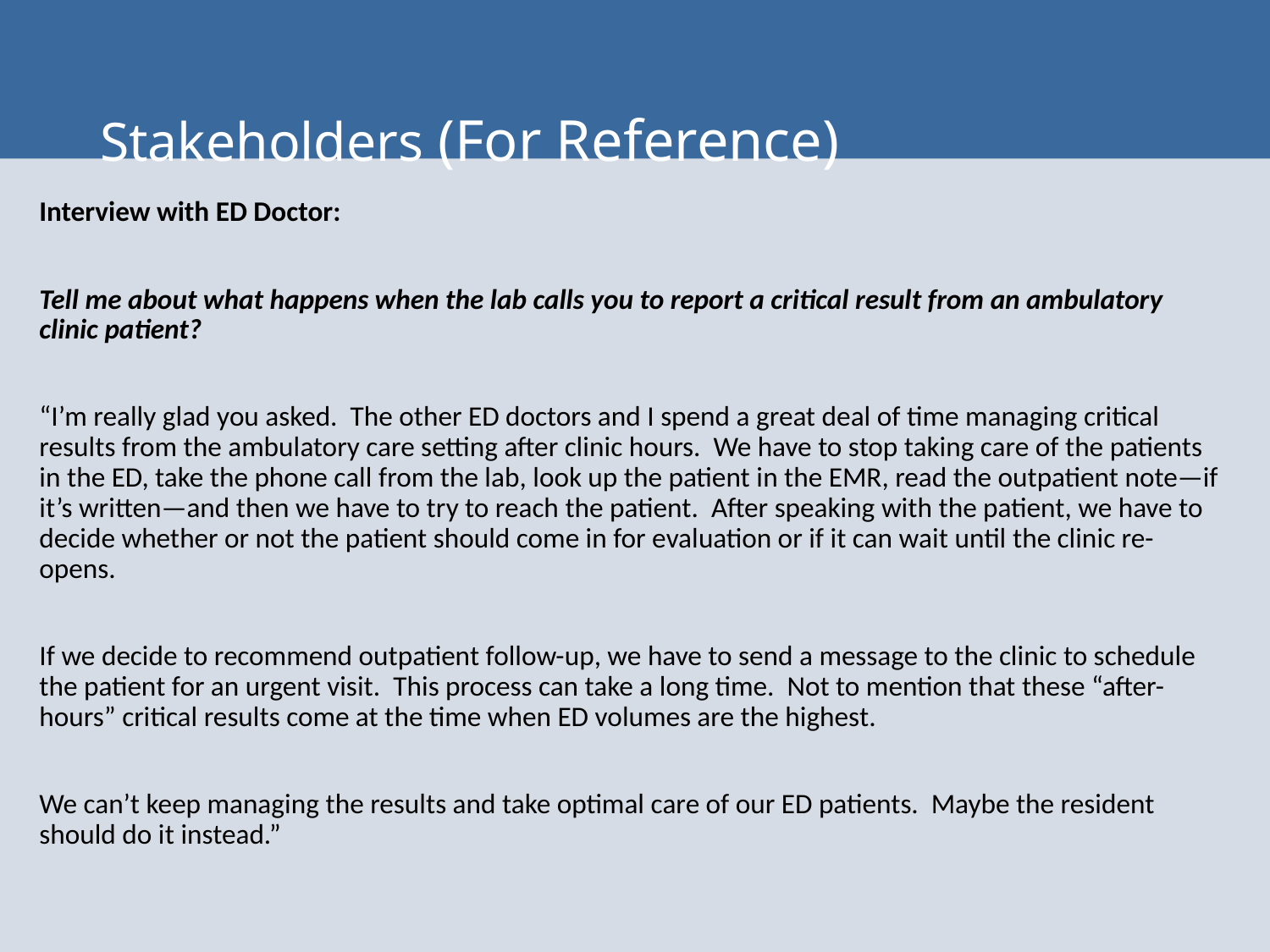

# Stakeholders (For Reference)
Interview with ED Doctor:
Tell me about what happens when the lab calls you to report a critical result from an ambulatory clinic patient?
“I’m really glad you asked. The other ED doctors and I spend a great deal of time managing critical results from the ambulatory care setting after clinic hours. We have to stop taking care of the patients in the ED, take the phone call from the lab, look up the patient in the EMR, read the outpatient note—if it’s written—and then we have to try to reach the patient. After speaking with the patient, we have to decide whether or not the patient should come in for evaluation or if it can wait until the clinic re-opens.
If we decide to recommend outpatient follow-up, we have to send a message to the clinic to schedule the patient for an urgent visit. This process can take a long time. Not to mention that these “after-hours” critical results come at the time when ED volumes are the highest.
We can’t keep managing the results and take optimal care of our ED patients. Maybe the resident should do it instead.”

## Slide 6
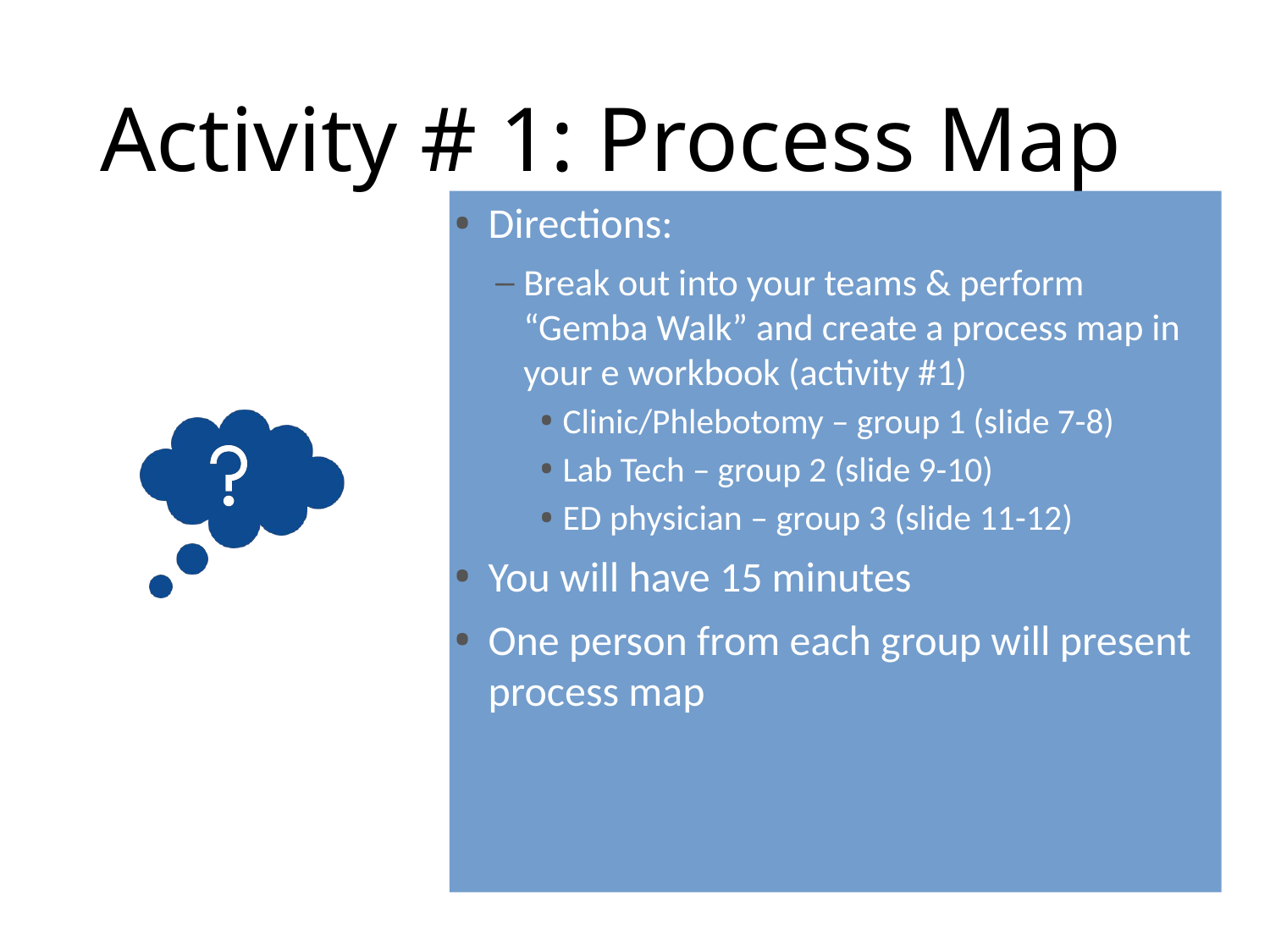

# Activity # 1: Process Map
Directions:
Break out into your teams & perform “Gemba Walk” and create a process map in your e workbook (activity #1)
Clinic/Phlebotomy – group 1 (slide 7-8)
Lab Tech – group 2 (slide 9-10)
ED physician – group 3 (slide 11-12)
You will have 15 minutes
One person from each group will present process map

## Slide 7
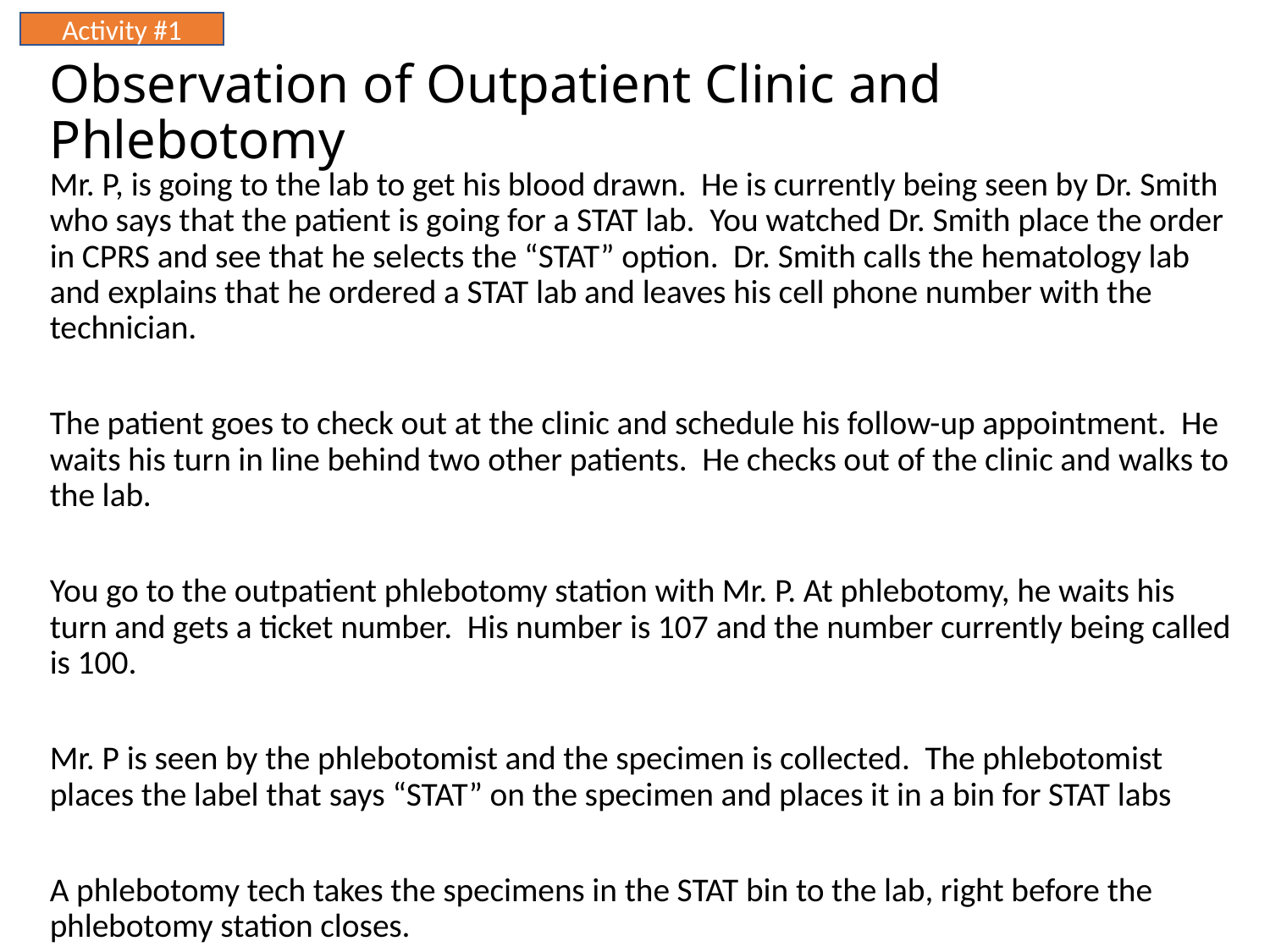

Activity #1
# Observation of Outpatient Clinic and Phlebotomy
Mr. P, is going to the lab to get his blood drawn. He is currently being seen by Dr. Smith who says that the patient is going for a STAT lab. You watched Dr. Smith place the order in CPRS and see that he selects the “STAT” option. Dr. Smith calls the hematology lab and explains that he ordered a STAT lab and leaves his cell phone number with the technician.
The patient goes to check out at the clinic and schedule his follow-up appointment. He waits his turn in line behind two other patients. He checks out of the clinic and walks to the lab.
You go to the outpatient phlebotomy station with Mr. P. At phlebotomy, he waits his turn and gets a ticket number. His number is 107 and the number currently being called is 100.
Mr. P is seen by the phlebotomist and the specimen is collected. The phlebotomist places the label that says “STAT” on the specimen and places it in a bin for STAT labs
A phlebotomy tech takes the specimens in the STAT bin to the lab, right before the phlebotomy station closes.

## Slide 8
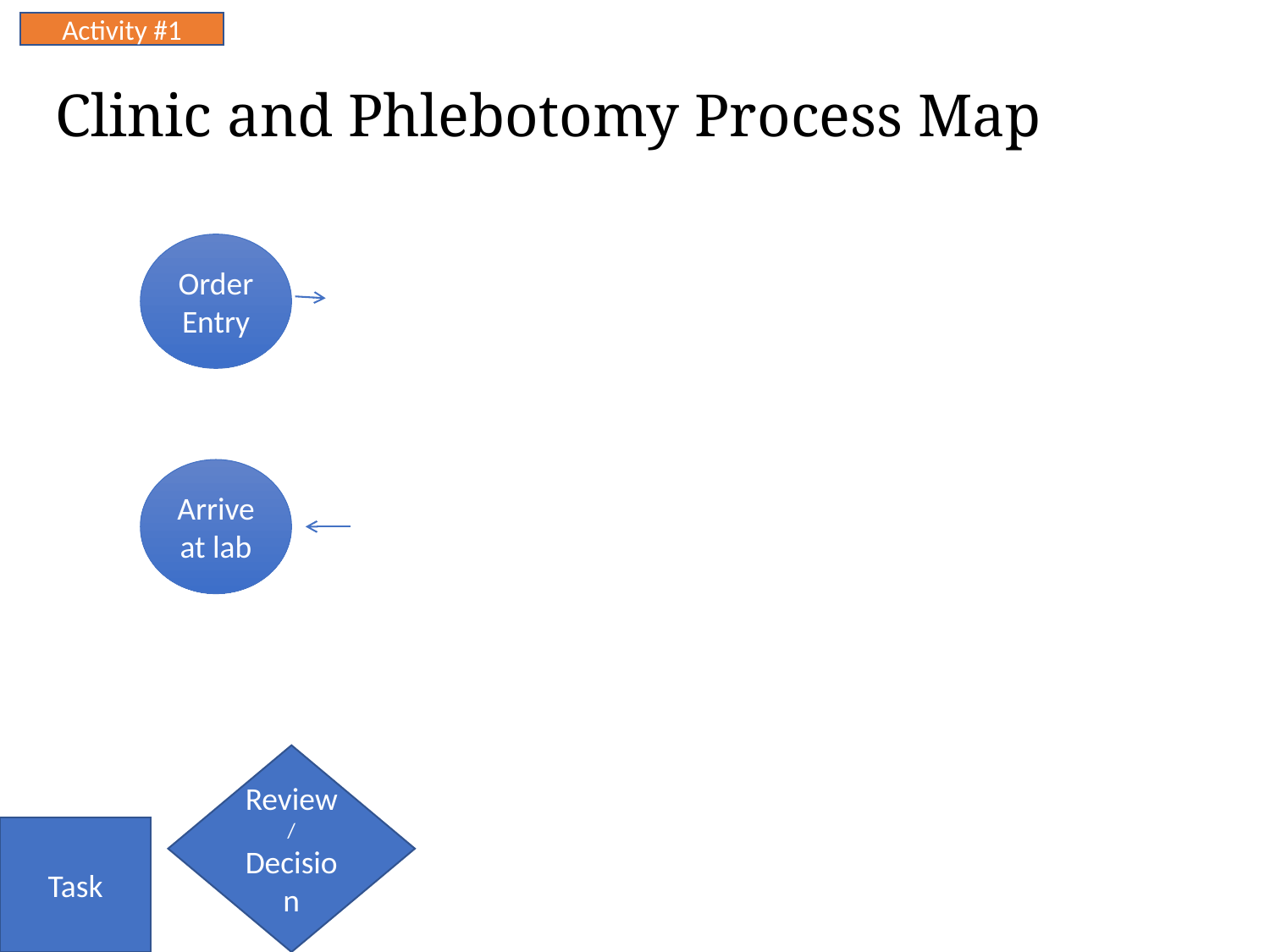

Activity #1
# Clinic and Phlebotomy Process Map
Order Entry
Arrive at lab
Review/Decision
Task

## Slide 9
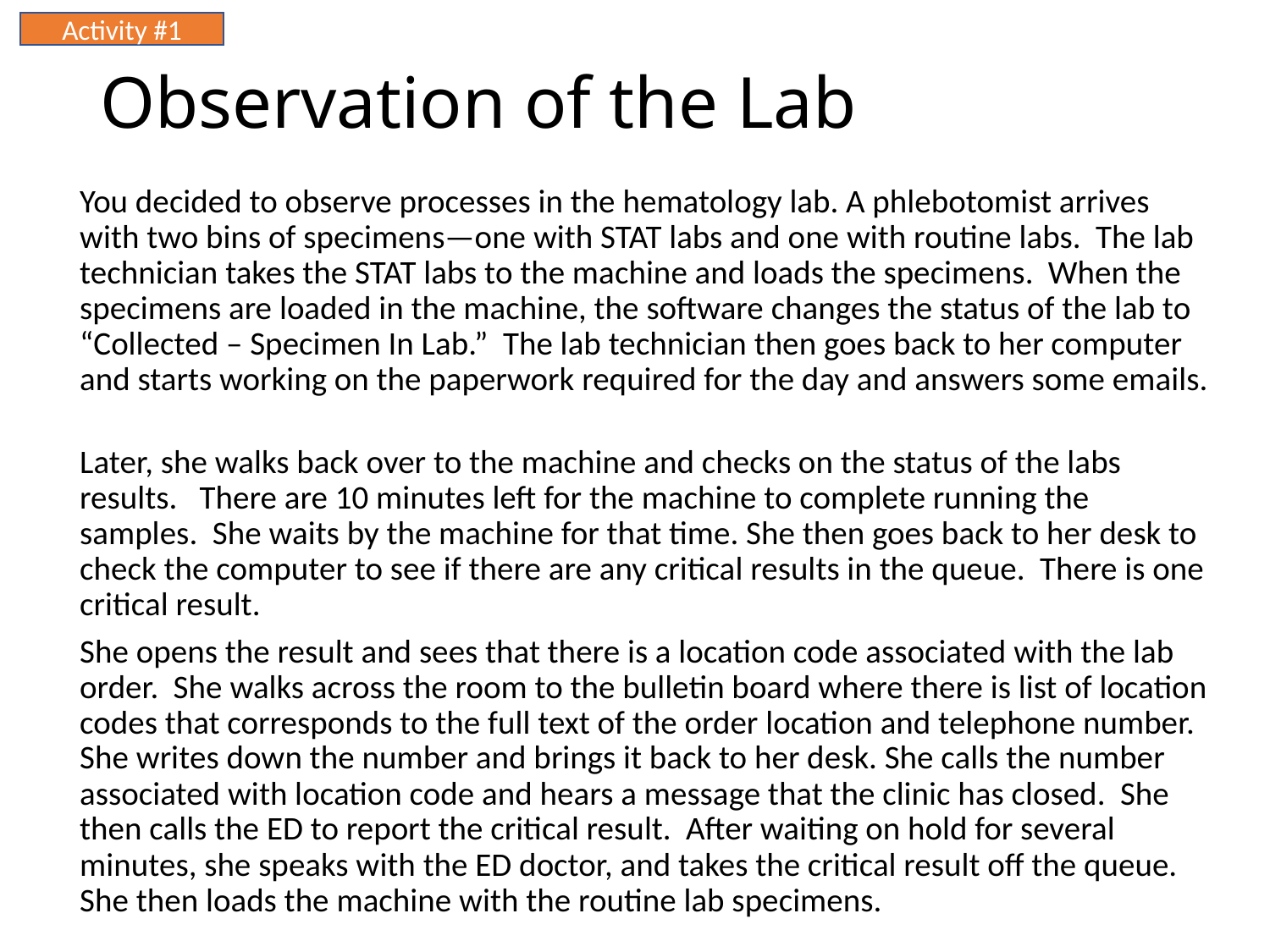

Activity #1
# Observation of the Lab
You decided to observe processes in the hematology lab. A phlebotomist arrives with two bins of specimens—one with STAT labs and one with routine labs. The lab technician takes the STAT labs to the machine and loads the specimens. When the specimens are loaded in the machine, the software changes the status of the lab to “Collected – Specimen In Lab.” The lab technician then goes back to her computer and starts working on the paperwork required for the day and answers some emails.
Later, she walks back over to the machine and checks on the status of the labs results. There are 10 minutes left for the machine to complete running the samples. She waits by the machine for that time. She then goes back to her desk to check the computer to see if there are any critical results in the queue. There is one critical result.
She opens the result and sees that there is a location code associated with the lab order. She walks across the room to the bulletin board where there is list of location codes that corresponds to the full text of the order location and telephone number. She writes down the number and brings it back to her desk. She calls the number associated with location code and hears a message that the clinic has closed. She then calls the ED to report the critical result. After waiting on hold for several minutes, she speaks with the ED doctor, and takes the critical result off the queue. She then loads the machine with the routine lab specimens.

## Slide 10
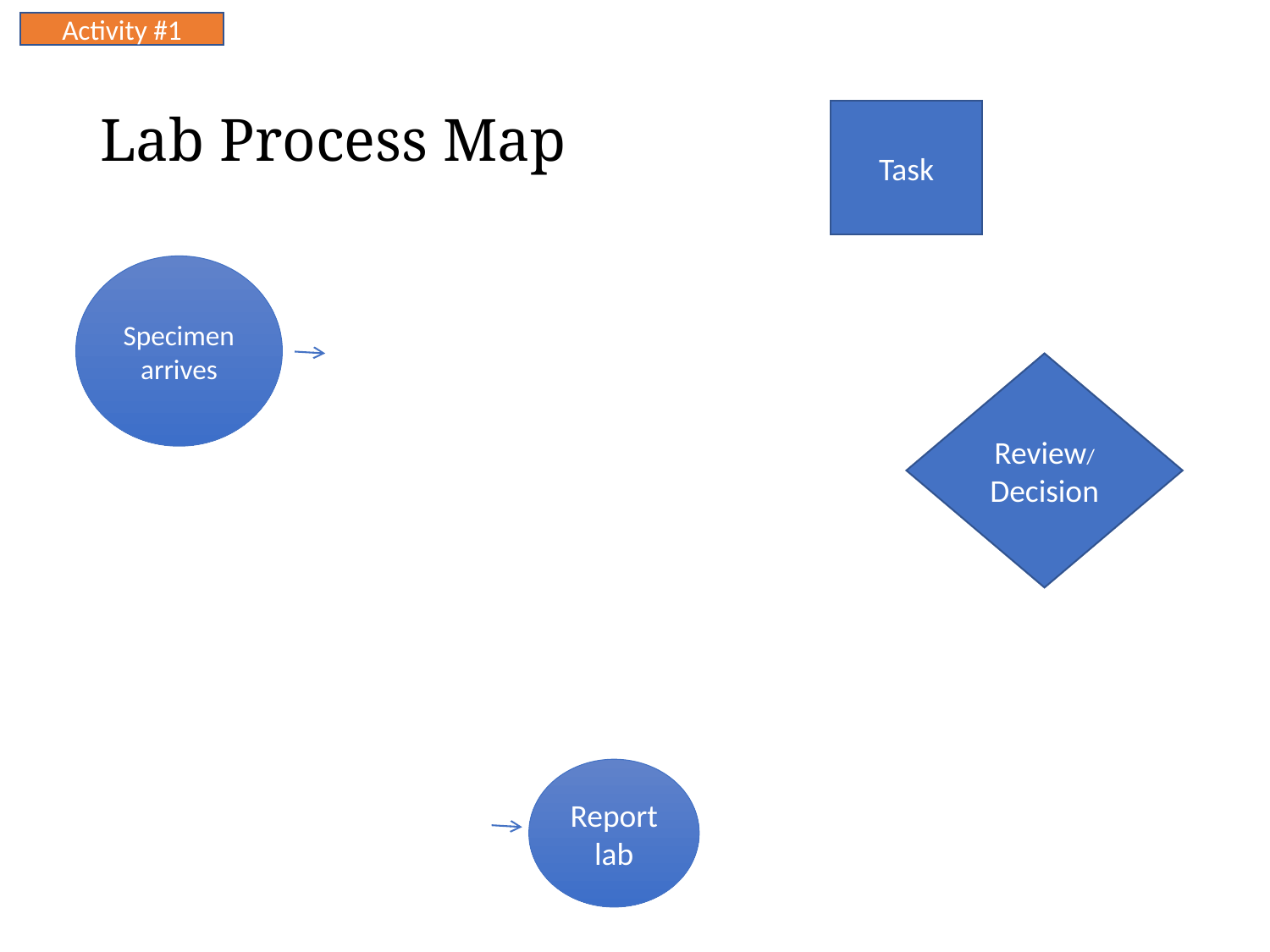

Activity #1
# Lab Process Map
Task
Specimen arrives
Review/Decision
Report lab

## Slide 11
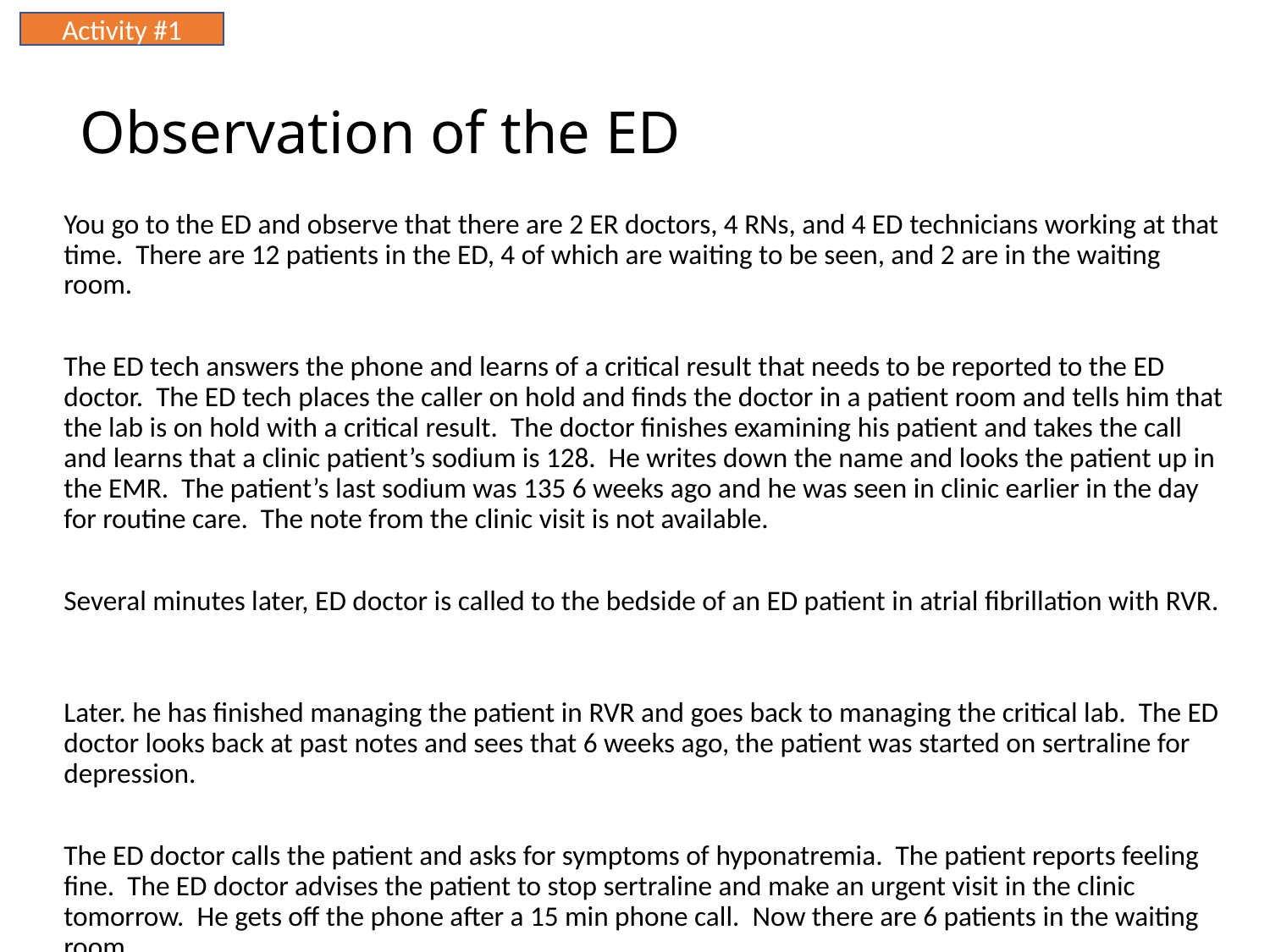

Activity #1
# Observation of the ED
You go to the ED and observe that there are 2 ER doctors, 4 RNs, and 4 ED technicians working at that time. There are 12 patients in the ED, 4 of which are waiting to be seen, and 2 are in the waiting room.
The ED tech answers the phone and learns of a critical result that needs to be reported to the ED doctor. The ED tech places the caller on hold and finds the doctor in a patient room and tells him that the lab is on hold with a critical result. The doctor finishes examining his patient and takes the call and learns that a clinic patient’s sodium is 128. He writes down the name and looks the patient up in the EMR. The patient’s last sodium was 135 6 weeks ago and he was seen in clinic earlier in the day for routine care. The note from the clinic visit is not available.
Several minutes later, ED doctor is called to the bedside of an ED patient in atrial fibrillation with RVR.
Later. he has finished managing the patient in RVR and goes back to managing the critical lab. The ED doctor looks back at past notes and sees that 6 weeks ago, the patient was started on sertraline for depression.
The ED doctor calls the patient and asks for symptoms of hyponatremia. The patient reports feeling fine. The ED doctor advises the patient to stop sertraline and make an urgent visit in the clinic tomorrow. He gets off the phone after a 15 min phone call. Now there are 6 patients in the waiting room.

## Slide 12
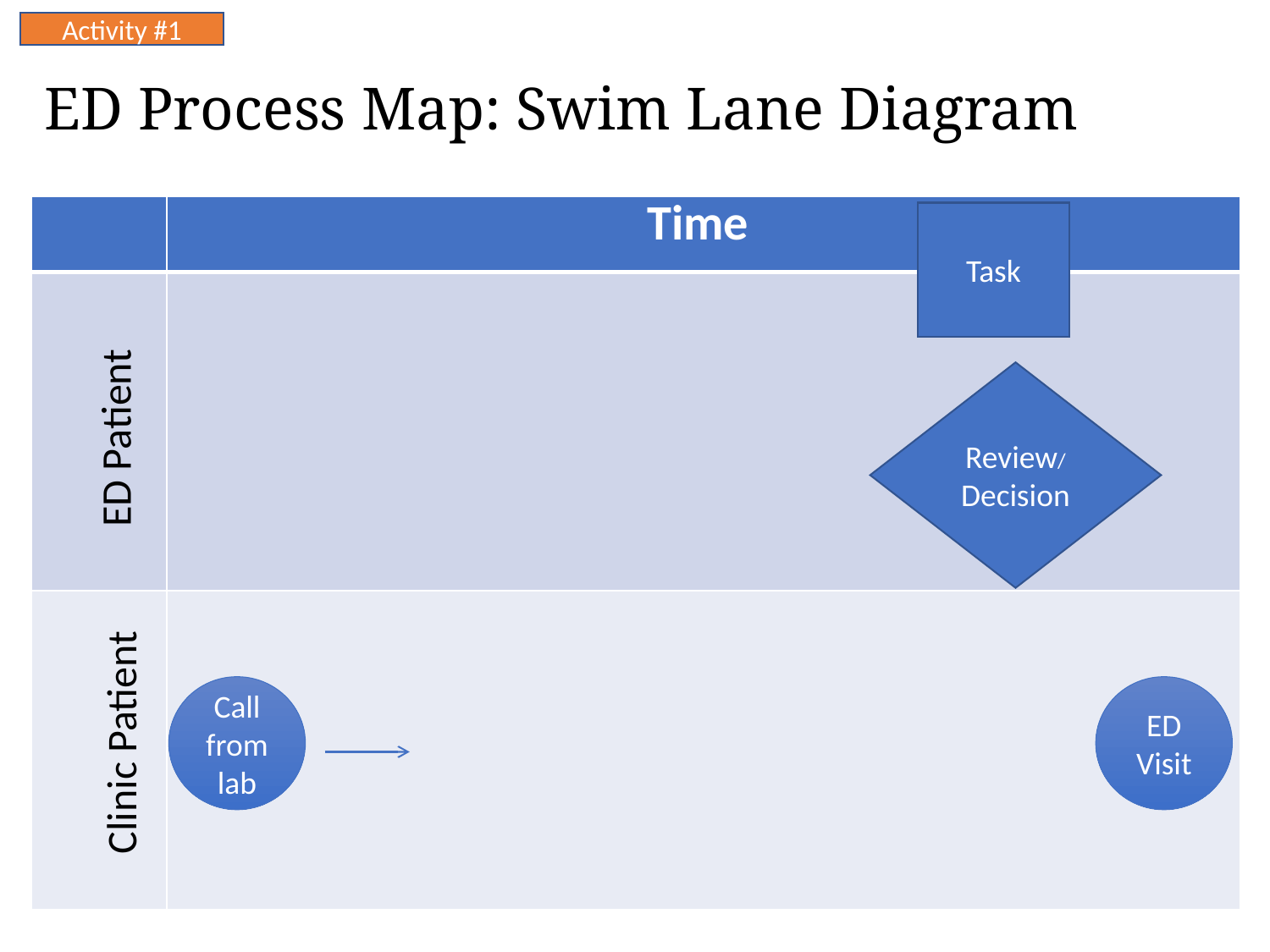

Activity #1
# ED Process Map: Swim Lane Diagram
| | Time |
| --- | --- |
| | |
| | |
Task
Review/Decision
ED Patient
Call from lab
ED Visit
Clinic Patient

## Slide 13
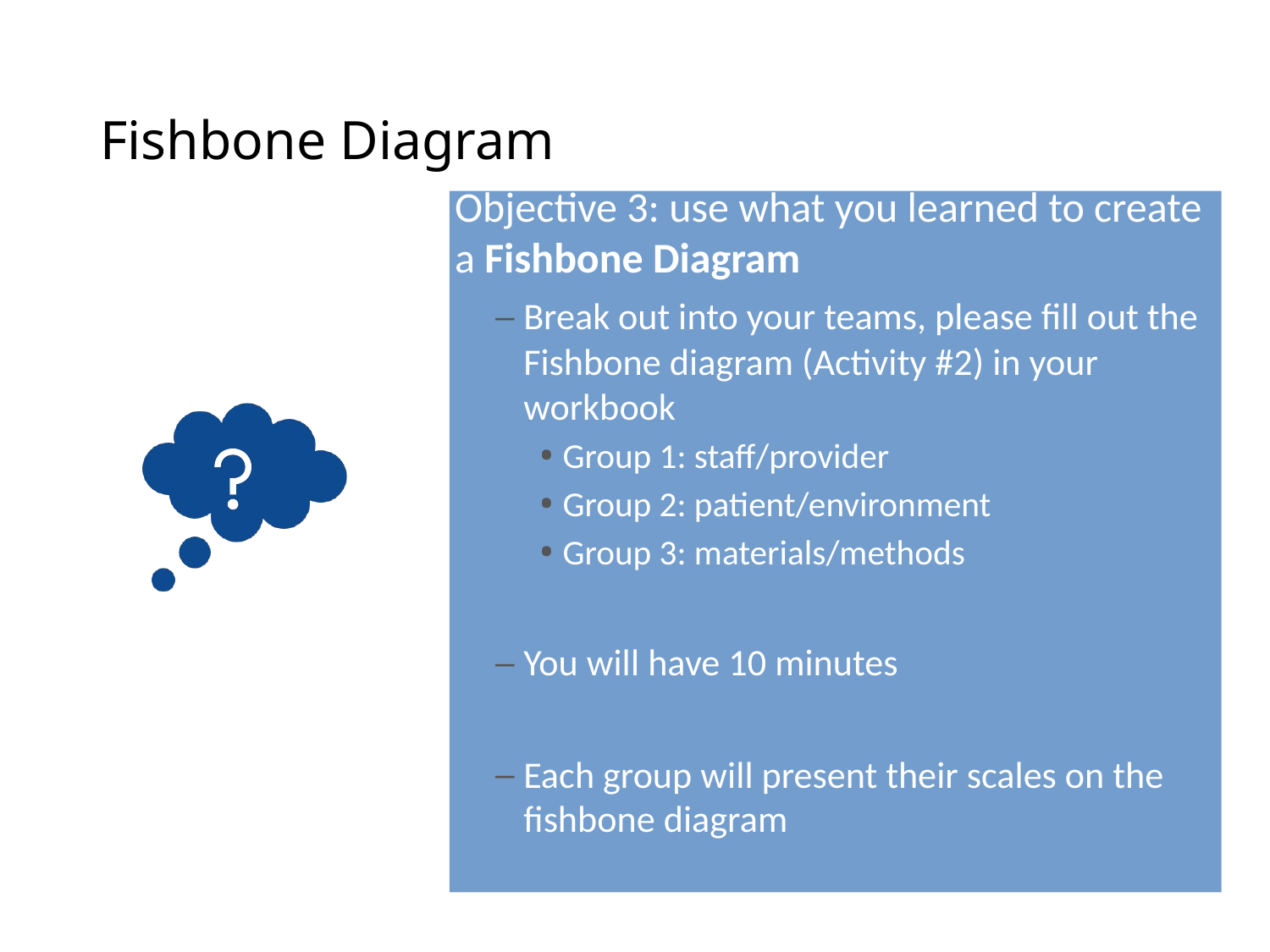

# Fishbone Diagram
Objective 3: use what you learned to create a Fishbone Diagram
Break out into your teams, please fill out the Fishbone diagram (Activity #2) in your workbook
Group 1: staff/provider
Group 2: patient/environment
Group 3: materials/methods
You will have 10 minutes
Each group will present their scales on the fishbone diagram

## Slide 14
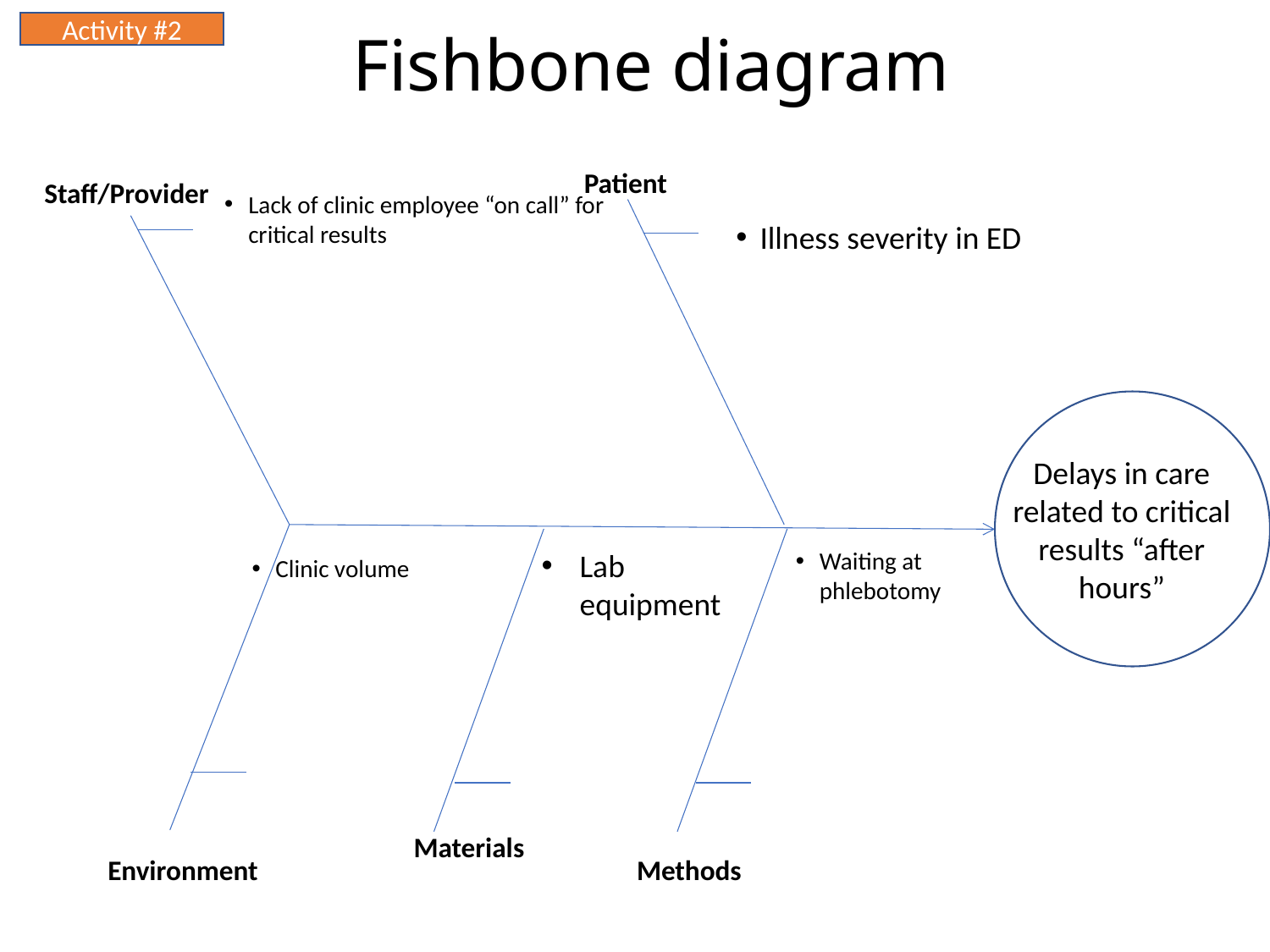

# Fishbone diagram
Activity #2
Patient
Staff/Provider
Lack of clinic employee “on call” for critical results
Illness severity in ED
Delays in care related to critical results “after hours”
Lab equipment
Waiting at phlebotomy
Clinic volume
Materials
Environment
Methods

## Slide 15
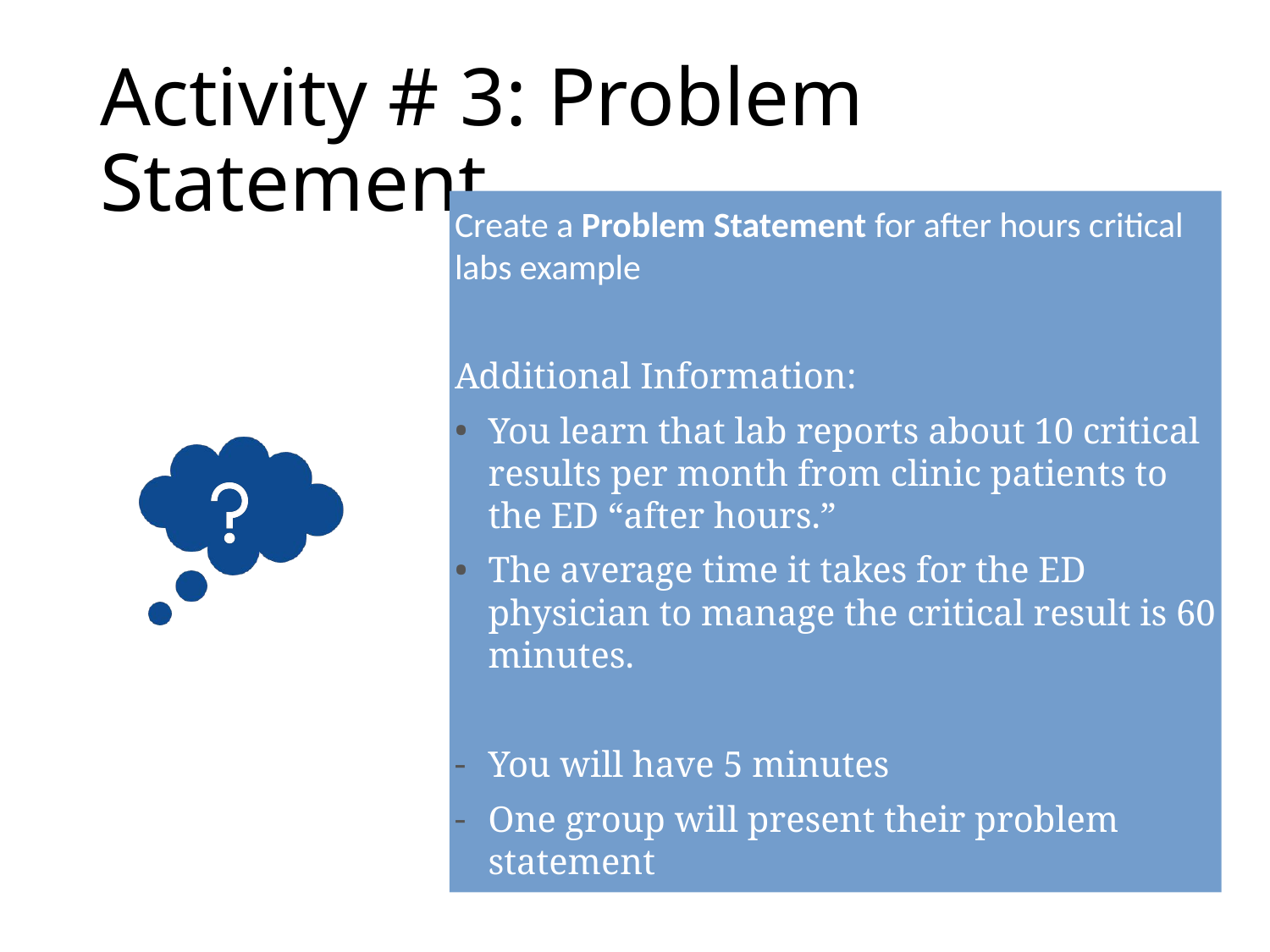

# Activity # 3: Problem Statement
Create a Problem Statement for after hours critical labs example
Additional Information:
You learn that lab reports about 10 critical results per month from clinic patients to the ED “after hours.”
The average time it takes for the ED physician to manage the critical result is 60 minutes.
You will have 5 minutes
One group will present their problem statement

## Slide 16
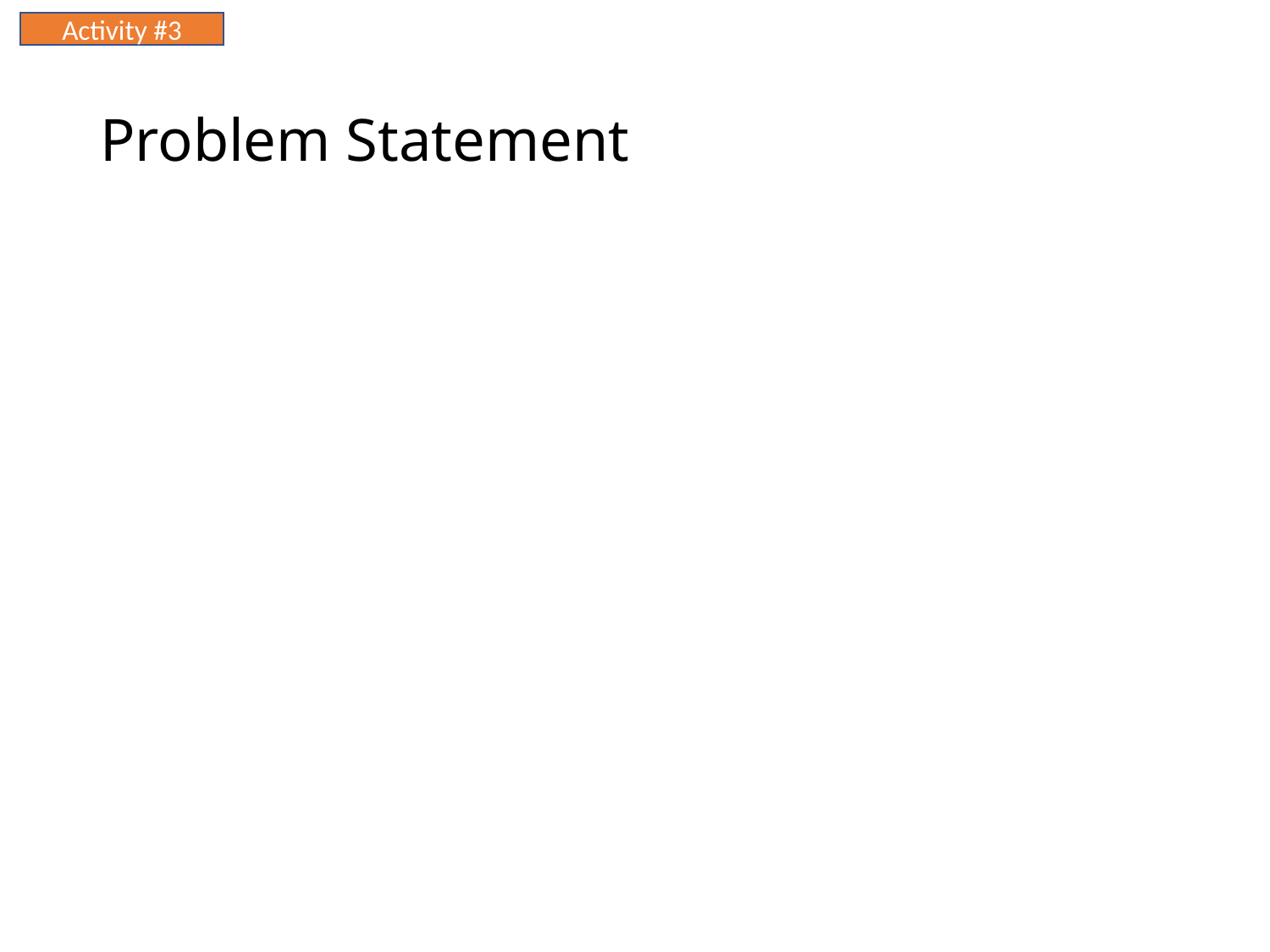

Activity #3
# Problem Statement

## Slide 17
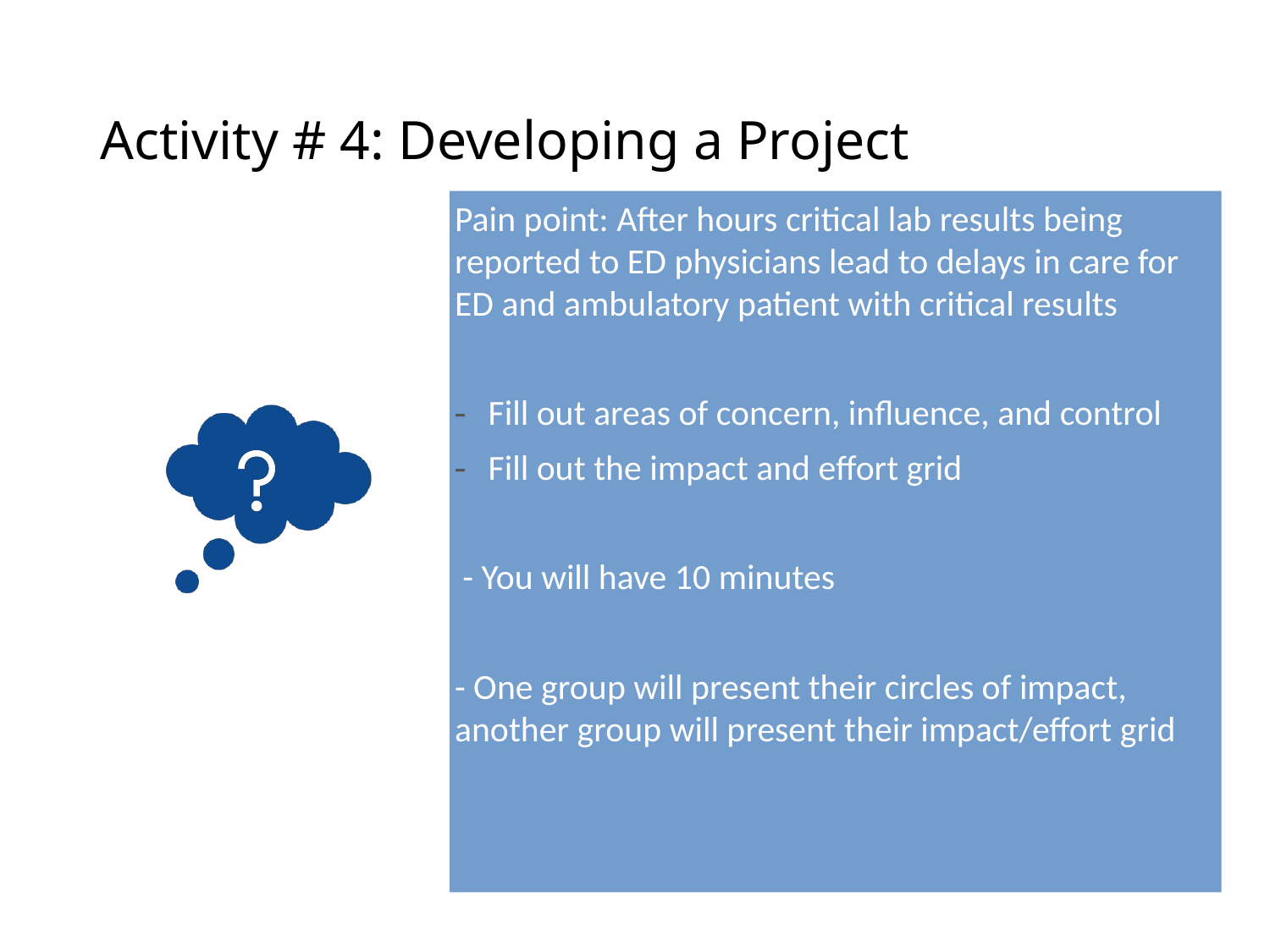

# Activity # 4: Developing a Project
Pain point: After hours critical lab results being reported to ED physicians lead to delays in care for ED and ambulatory patient with critical results
Fill out areas of concern, influence, and control
Fill out the impact and effort grid
 - You will have 10 minutes
- One group will present their circles of impact, another group will present their impact/effort grid

## Slide 18
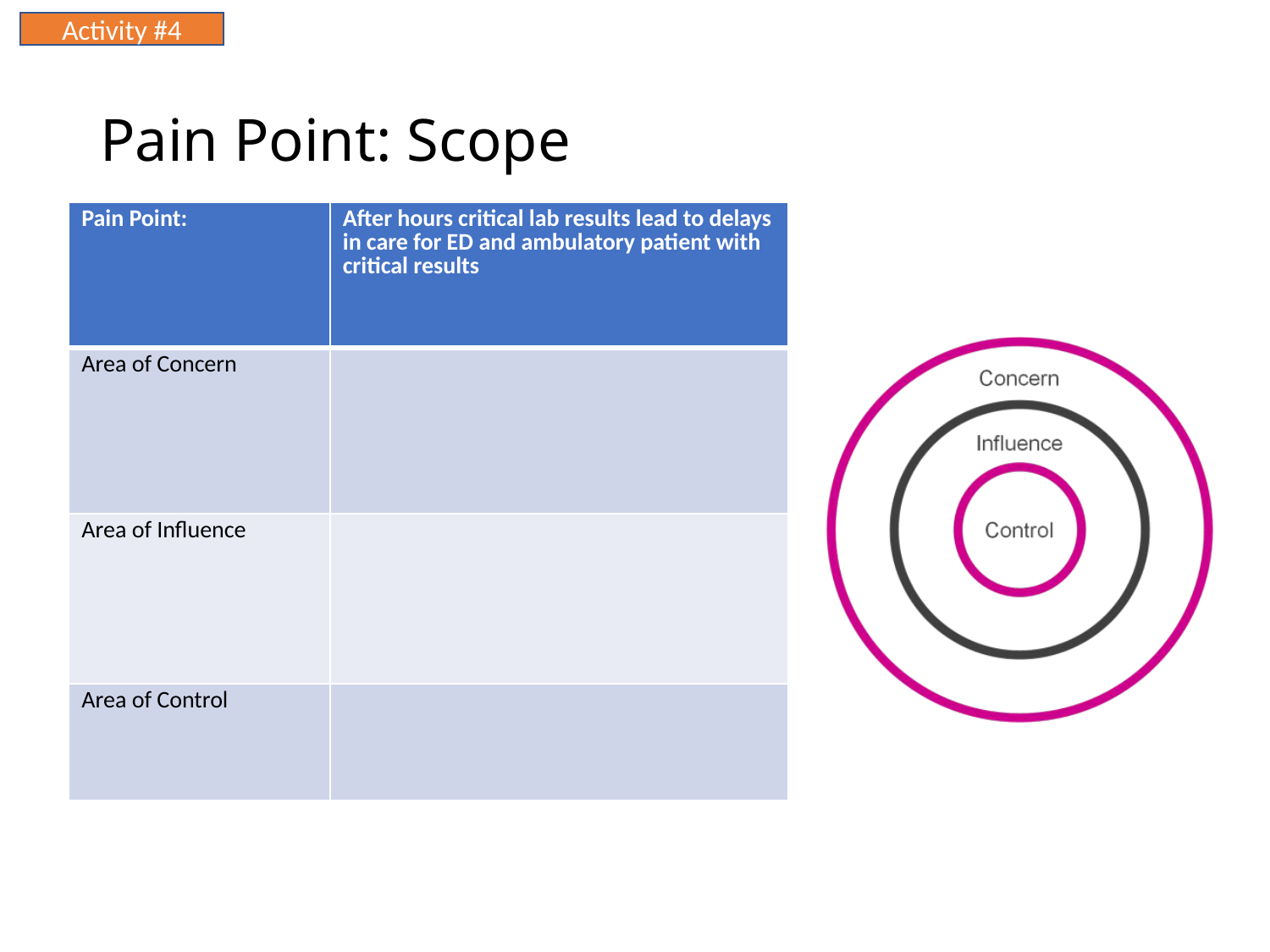

Activity #4
# Pain Point: Scope
| Pain Point: | After hours critical lab results lead to delays in care for ED and ambulatory patient with critical results |
| --- | --- |
| Area of Concern | |
| Area of Influence | |
| Area of Control | |

## Slide 19
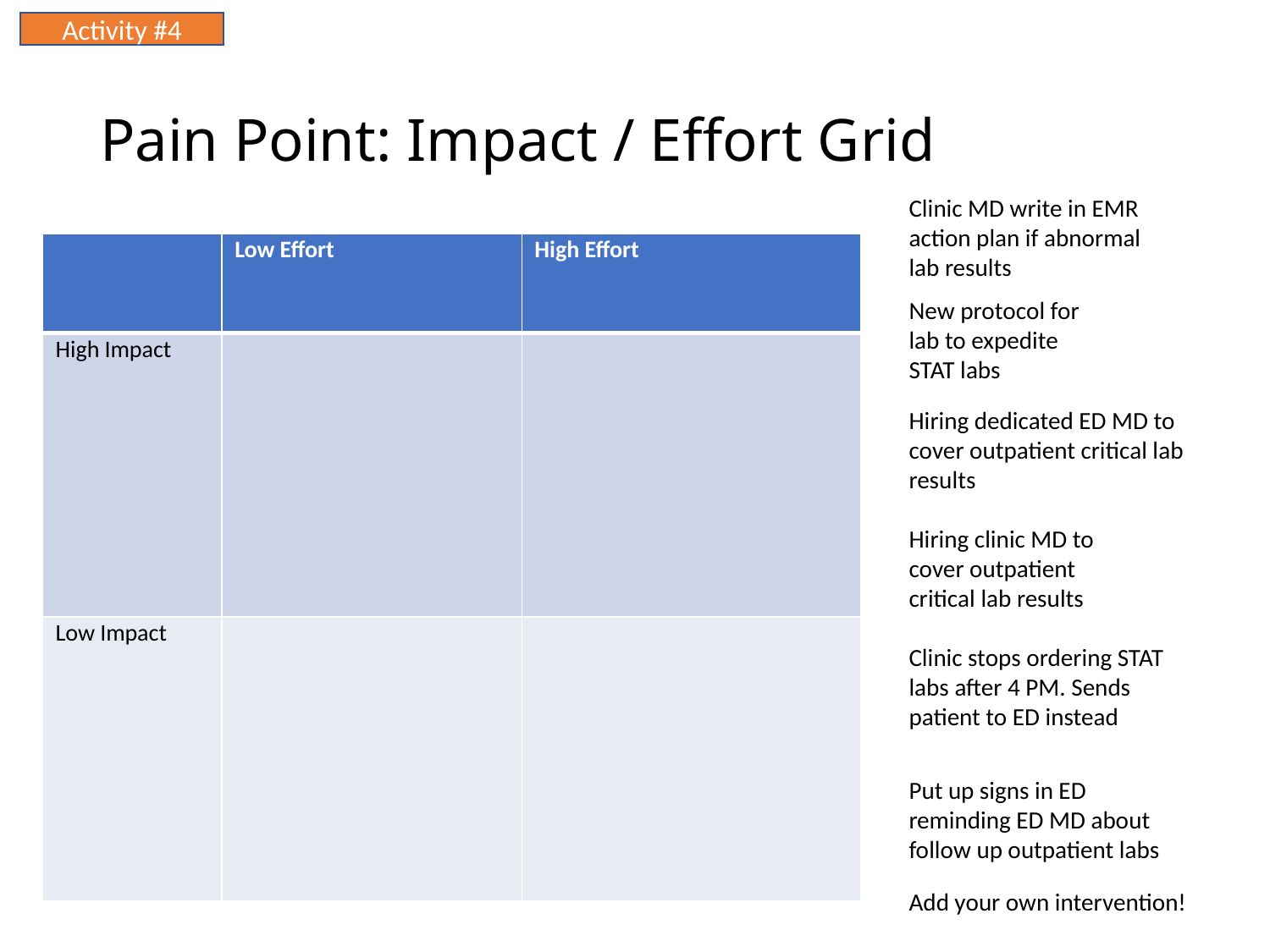

Activity #4
# Pain Point: Impact / Effort Grid
Clinic MD write in EMR action plan if abnormal lab results
| | Low Effort | High Effort |
| --- | --- | --- |
| High Impact | | |
| Low Impact | | |
New protocol for lab to expedite STAT labs
Hiring dedicated ED MD to cover outpatient critical lab results
Hiring clinic MD to cover outpatient critical lab results
Clinic stops ordering STAT labs after 4 PM. Sends patient to ED instead
Put up signs in ED reminding ED MD about follow up outpatient labs
Add your own intervention!
