# Supplementary material for: Building Resident Quality Improvement Knowledge and Engagement Through a Longitudinal, Mentored, and Experiential Learning-Based Quality Improvement Curriculum
Source: MedEdPORTAL. 2023 Apr 18;19:11310. doi: 10.15766/mep_2374-8265.11310 (PMC10110773; doi:10.15766/mep_2374-8265.11310)
Supplement: Supplementary file 1 — Session 1 Slides.pptxSession 1 Workbook.pptxSession 2 Slides.pptxSession 2 Workbook.pptxSession 3 Slides.pptxSession 4 Work-in-Progress Presentation Template.pptxSession 5 Slides.pptxQI Charter Template.docxFaculty Milestones.docxFaculty Guide.docxResident Survey.docx [file mep_2374-8265.11310-s001.zip › C. Session 2 Slides.pptx]

## Slide 1
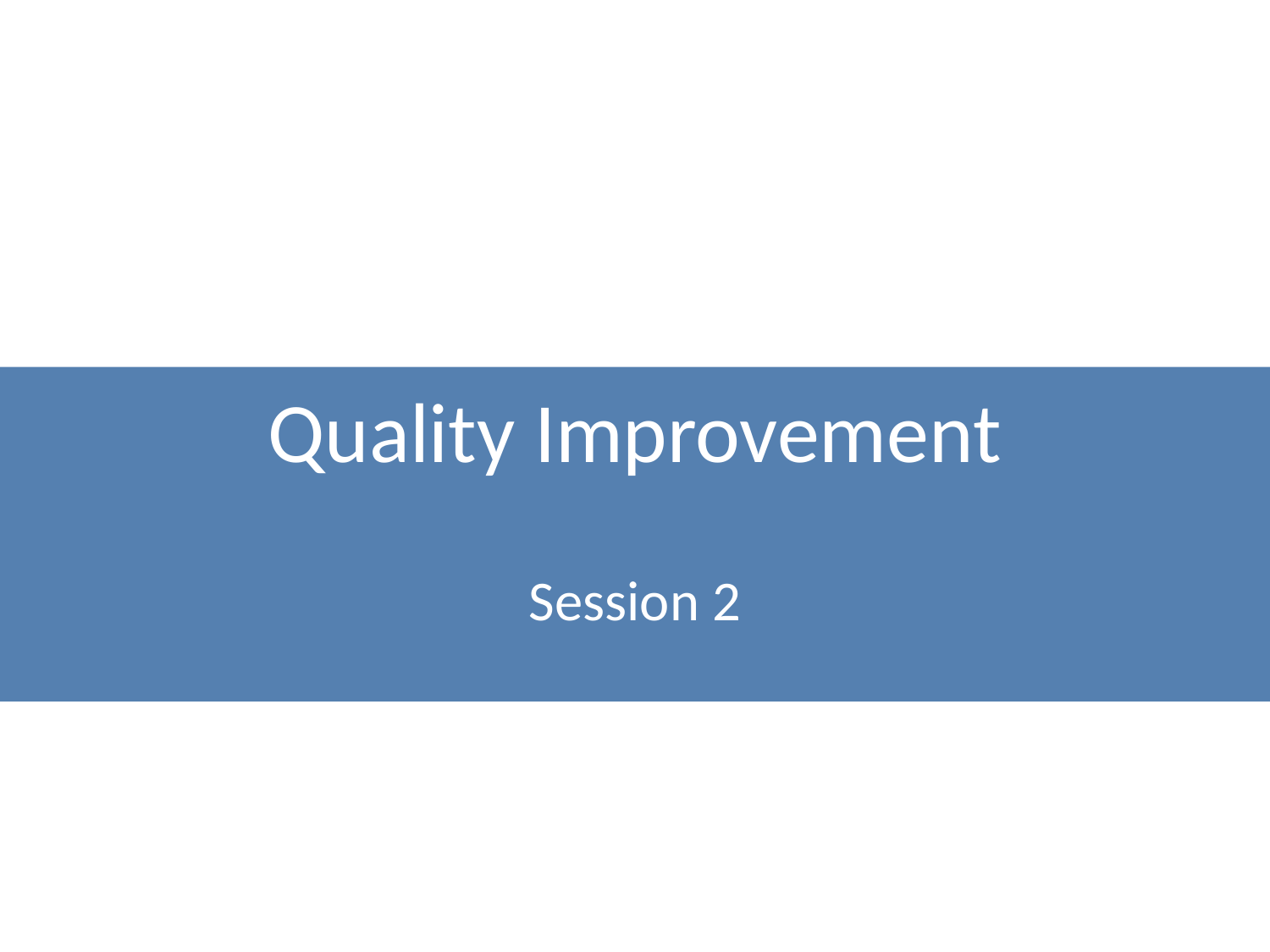

# Quality Improvement Session 2

## Slide 2
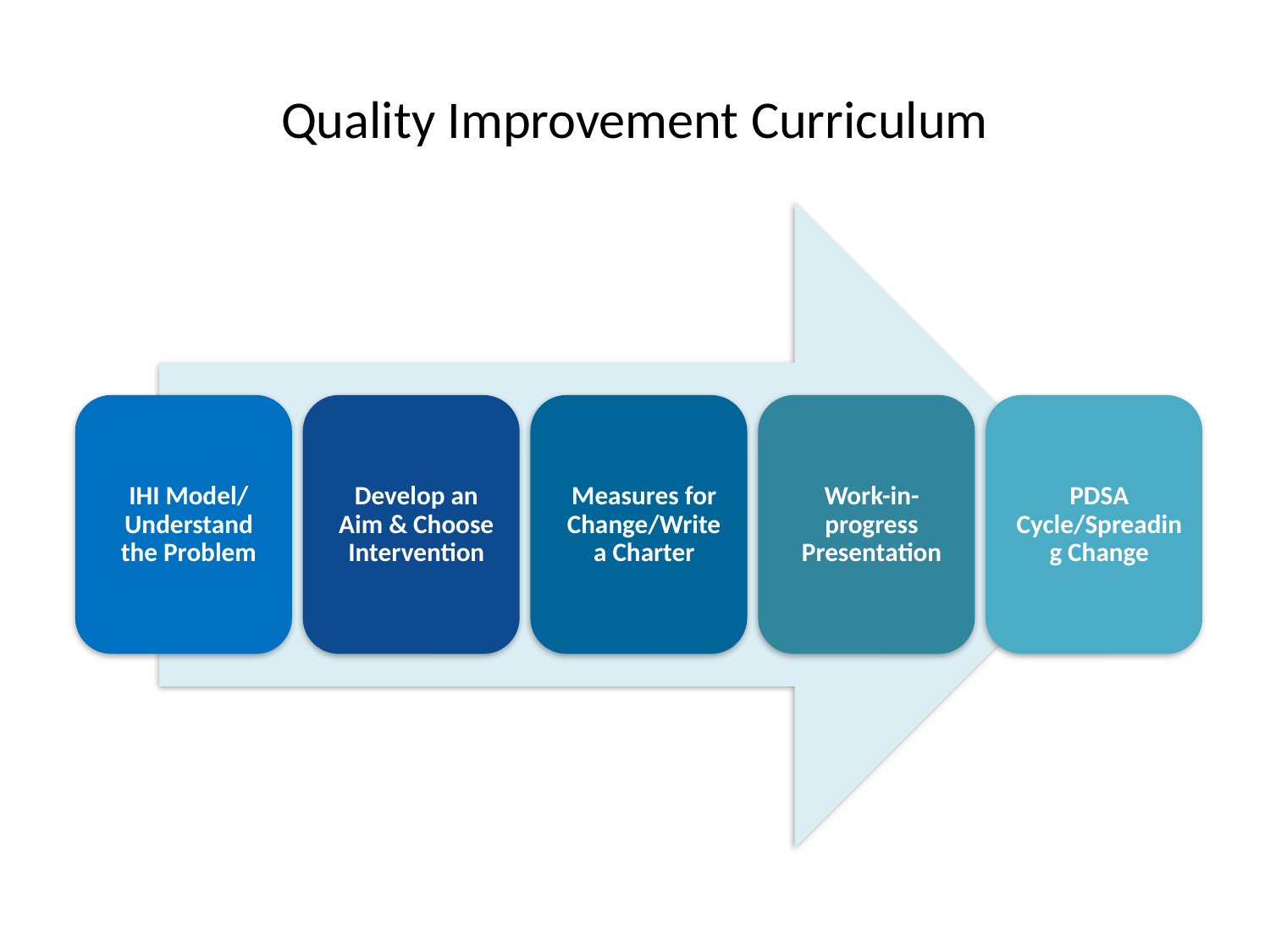

# Quality Improvement Curriculum

## Slide 3
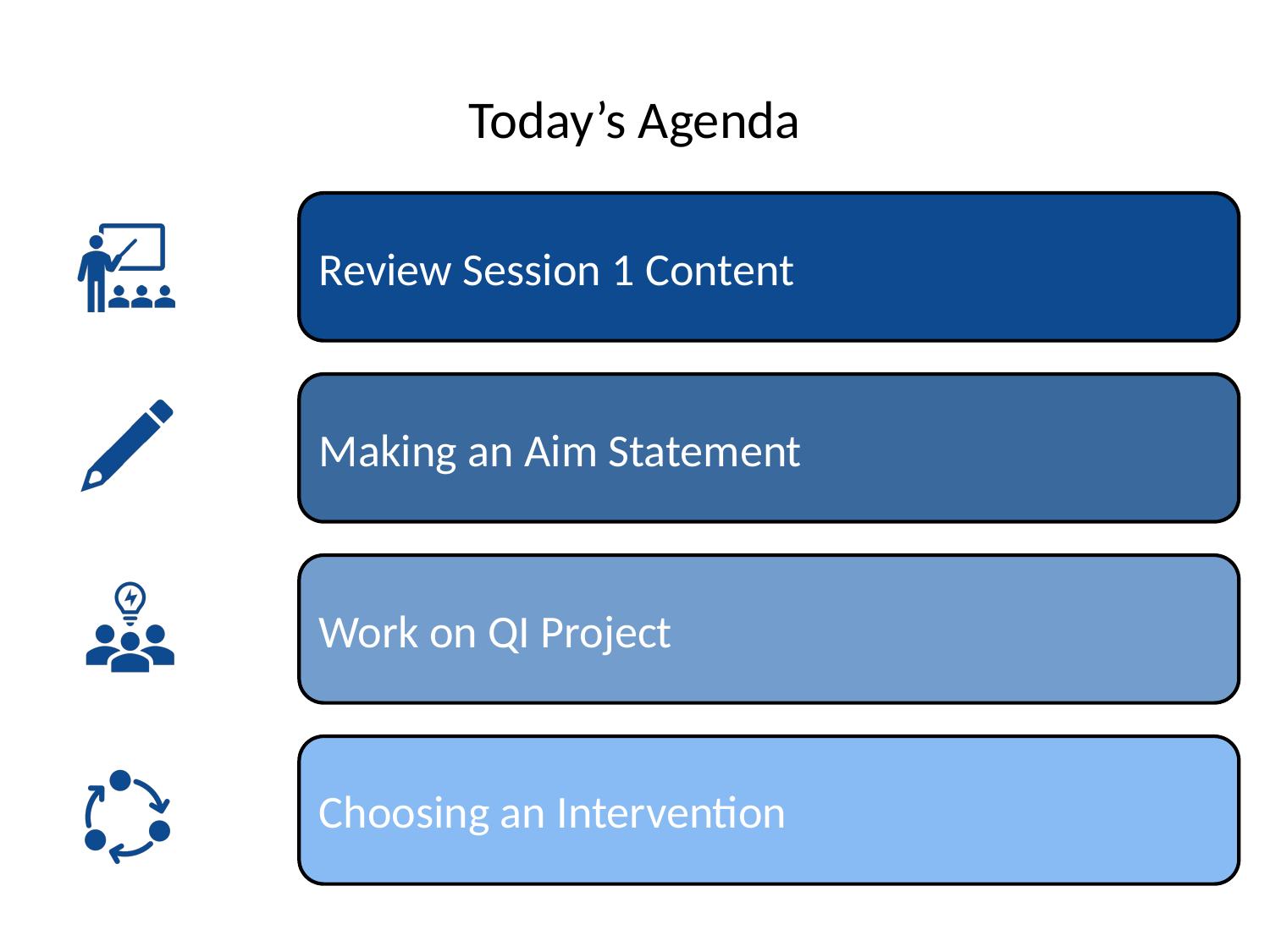

# Today’s Agenda
Review Session 1 Content
Making an Aim Statement
Work on QI Project
Choosing an Intervention

## Slide 4
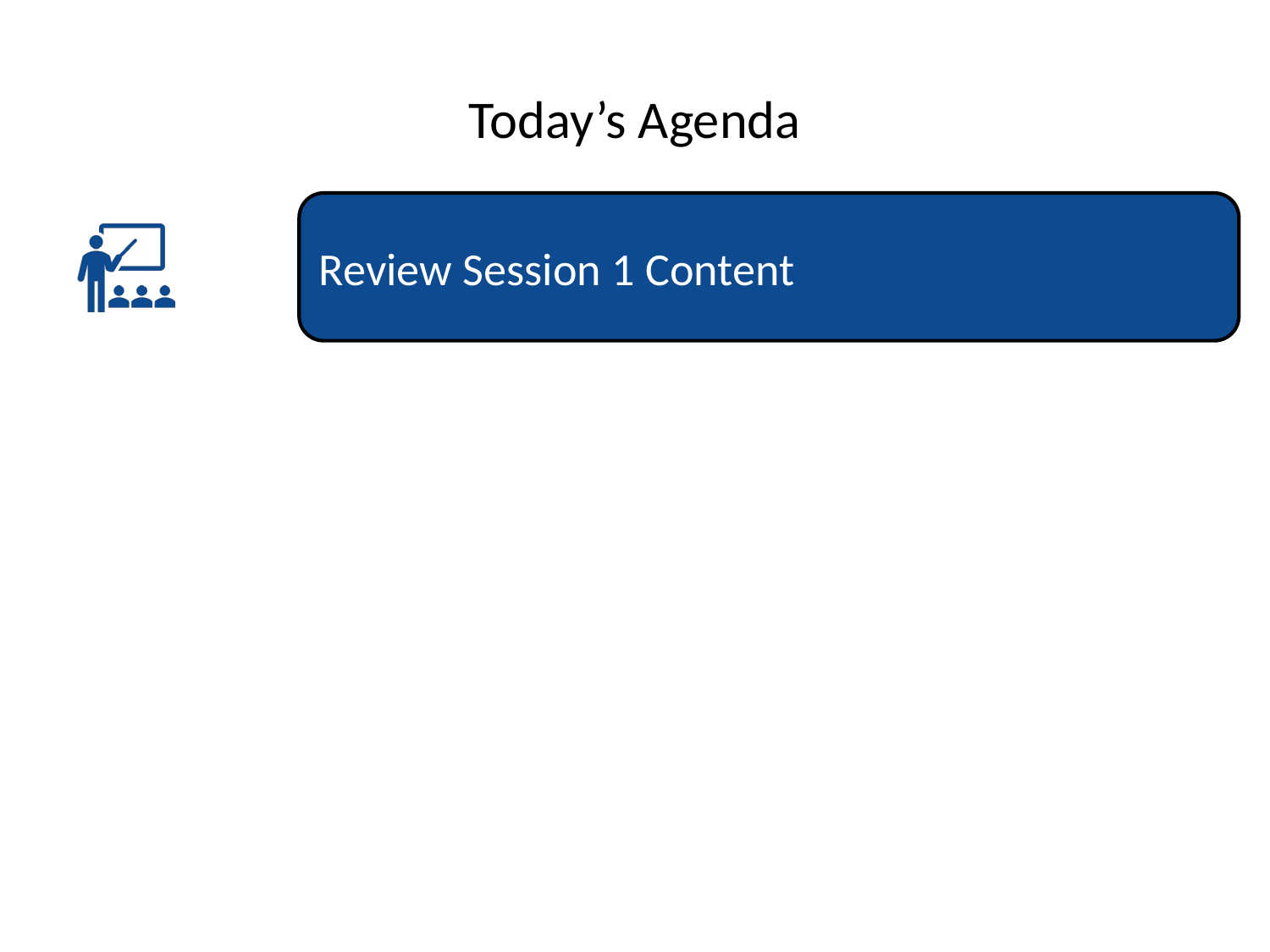

# Today’s Agenda
Review Session 1 Content

## Slide 5
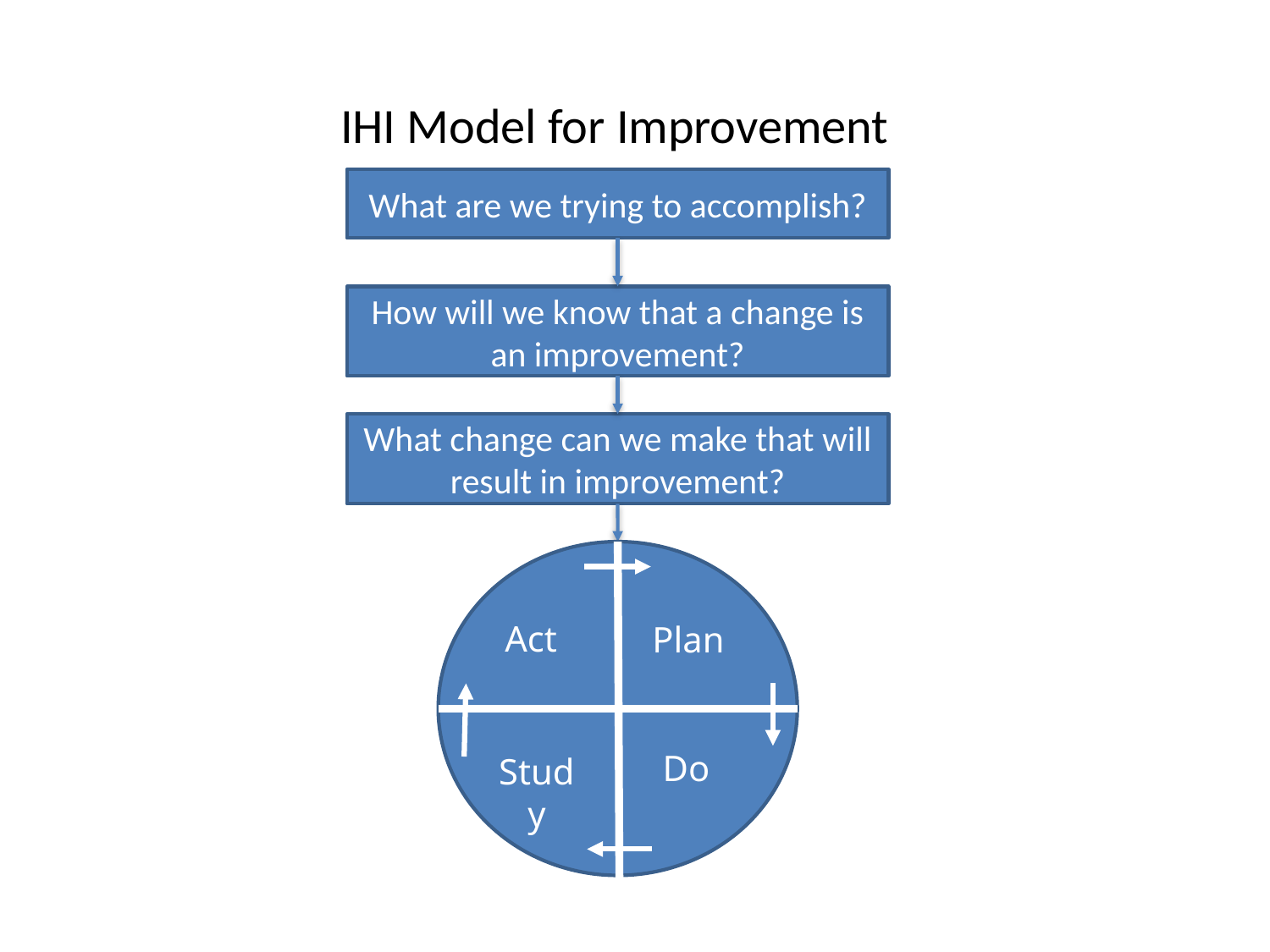

IHI Model for Improvement
What are we trying to accomplish?
How will we know that a change is an improvement?
What change can we make that will result in improvement?
Act
Plan
Do
Study

## Slide 6
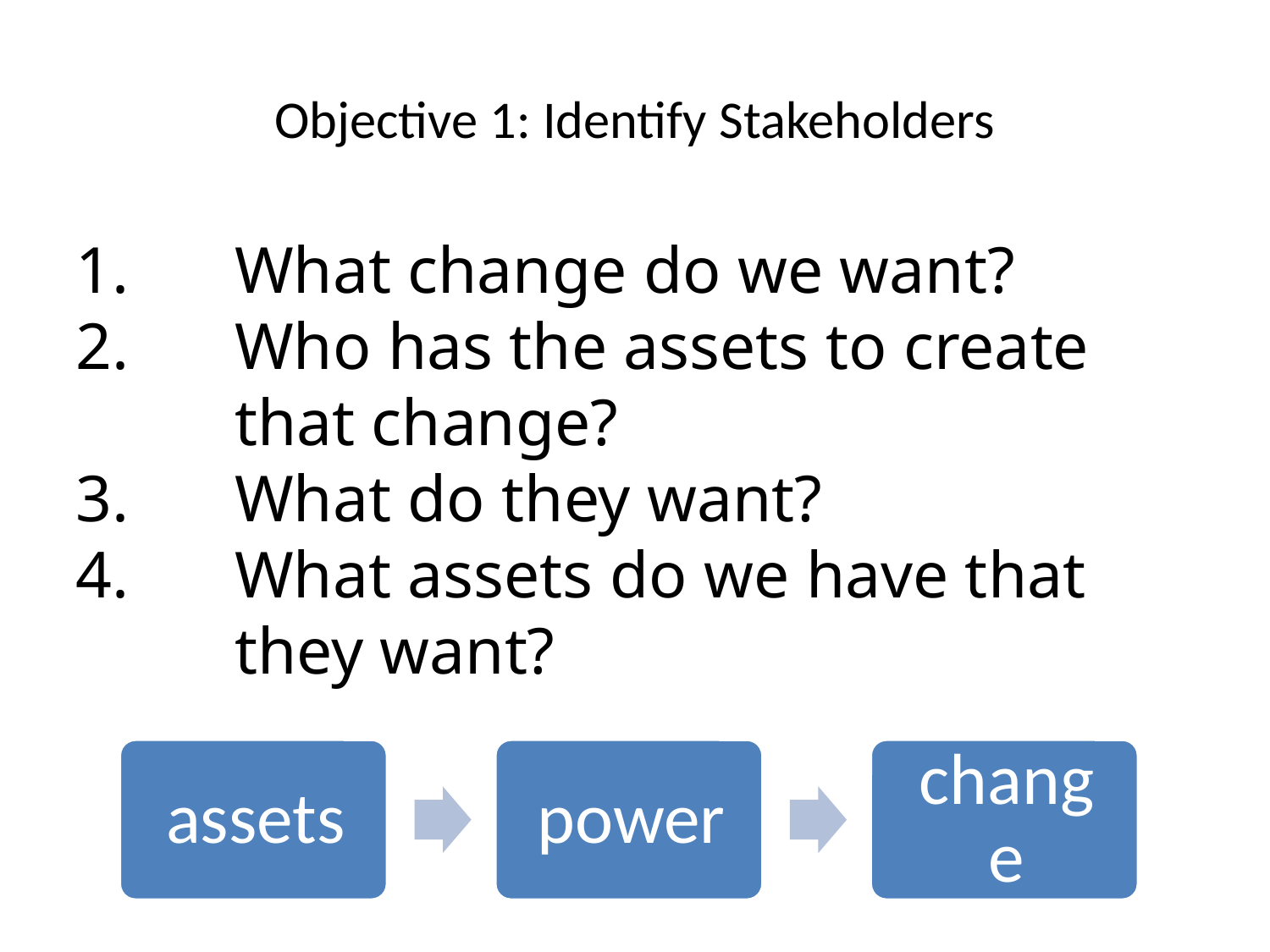

# Objective 1: Identify Stakeholders
What change do we want?
Who has the assets to create that change?
What do they want?
What assets do we have that they want?

## Slide 7
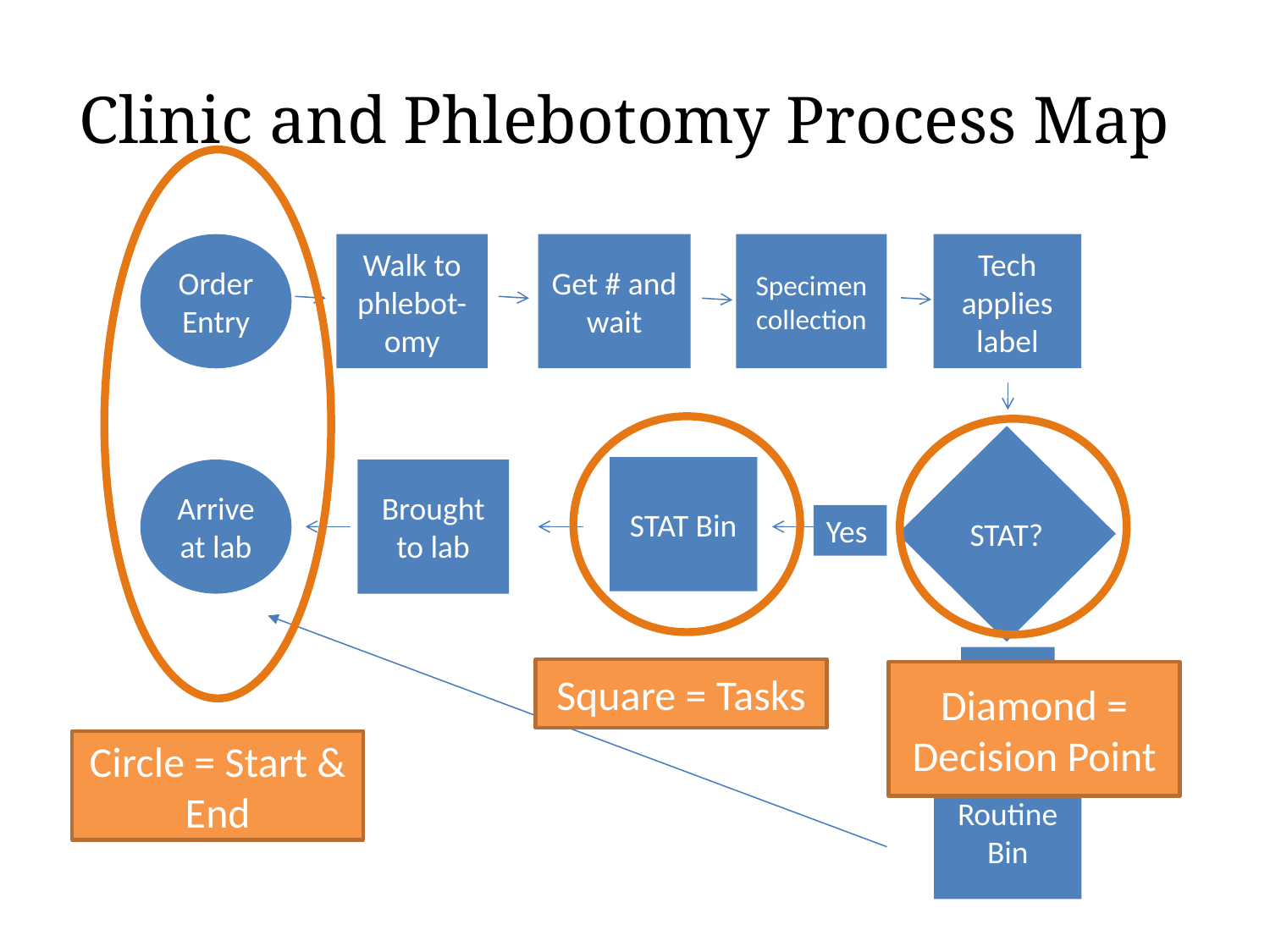

# Clinic and Phlebotomy Process Map
Order Entry
Walk to phlebot-omy
Get # and wait
Specimen collection
Tech applies label
STAT?
STAT Bin
Arrive at lab
Brought to lab
Yes
No
Routine Bin
Square = Tasks
Diamond = Decision Point
Circle = Start & End

## Slide 8
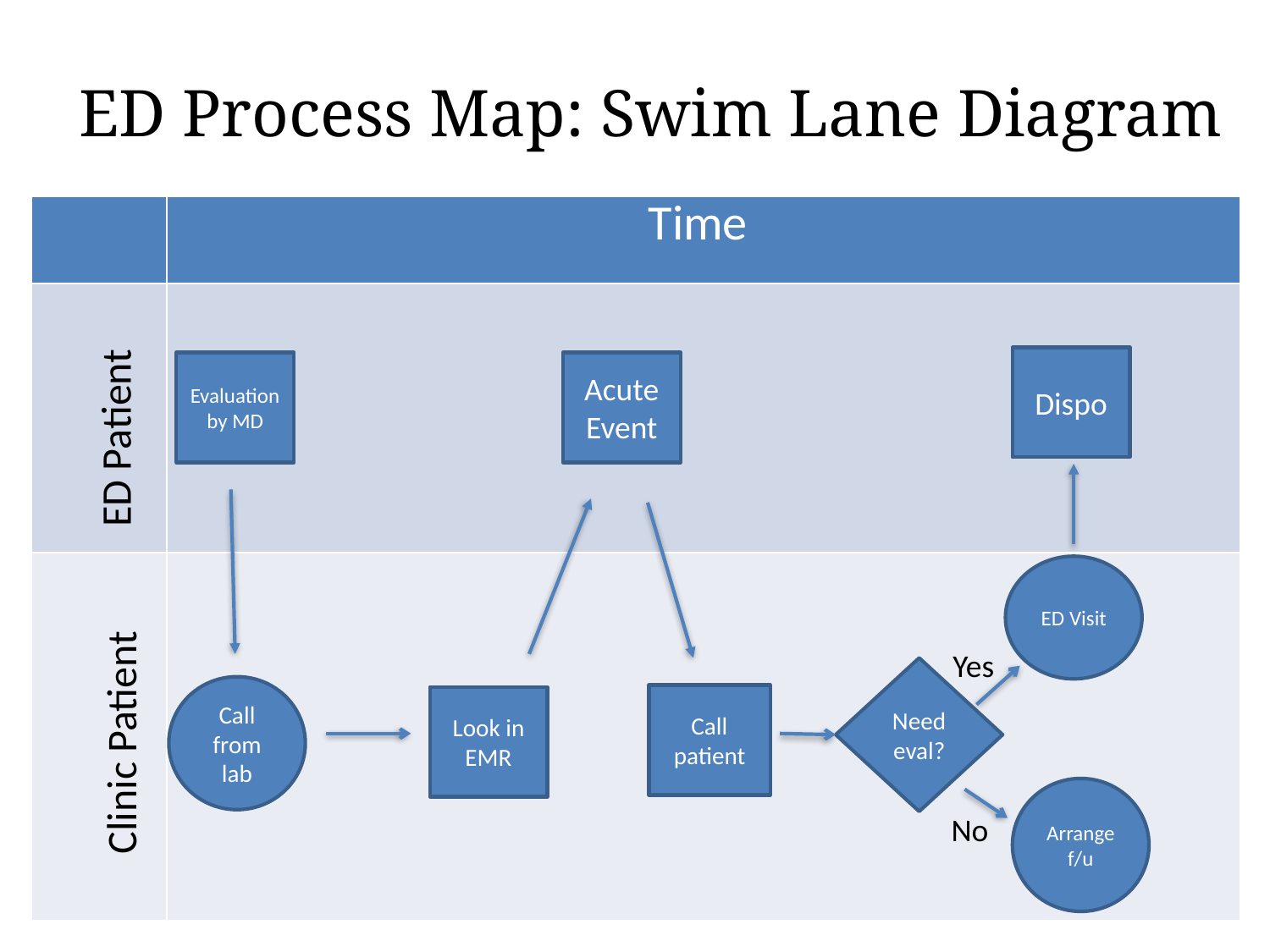

# ED Process Map: Swim Lane Diagram
| | Time |
| --- | --- |
| | |
| | |
Dispo
Acute Event
Evaluation by MD
ED Patient
ED Visit
Yes
Need eval?
Call from lab
Call patient
Look in EMR
Clinic Patient
Arrange f/u
No

## Slide 9
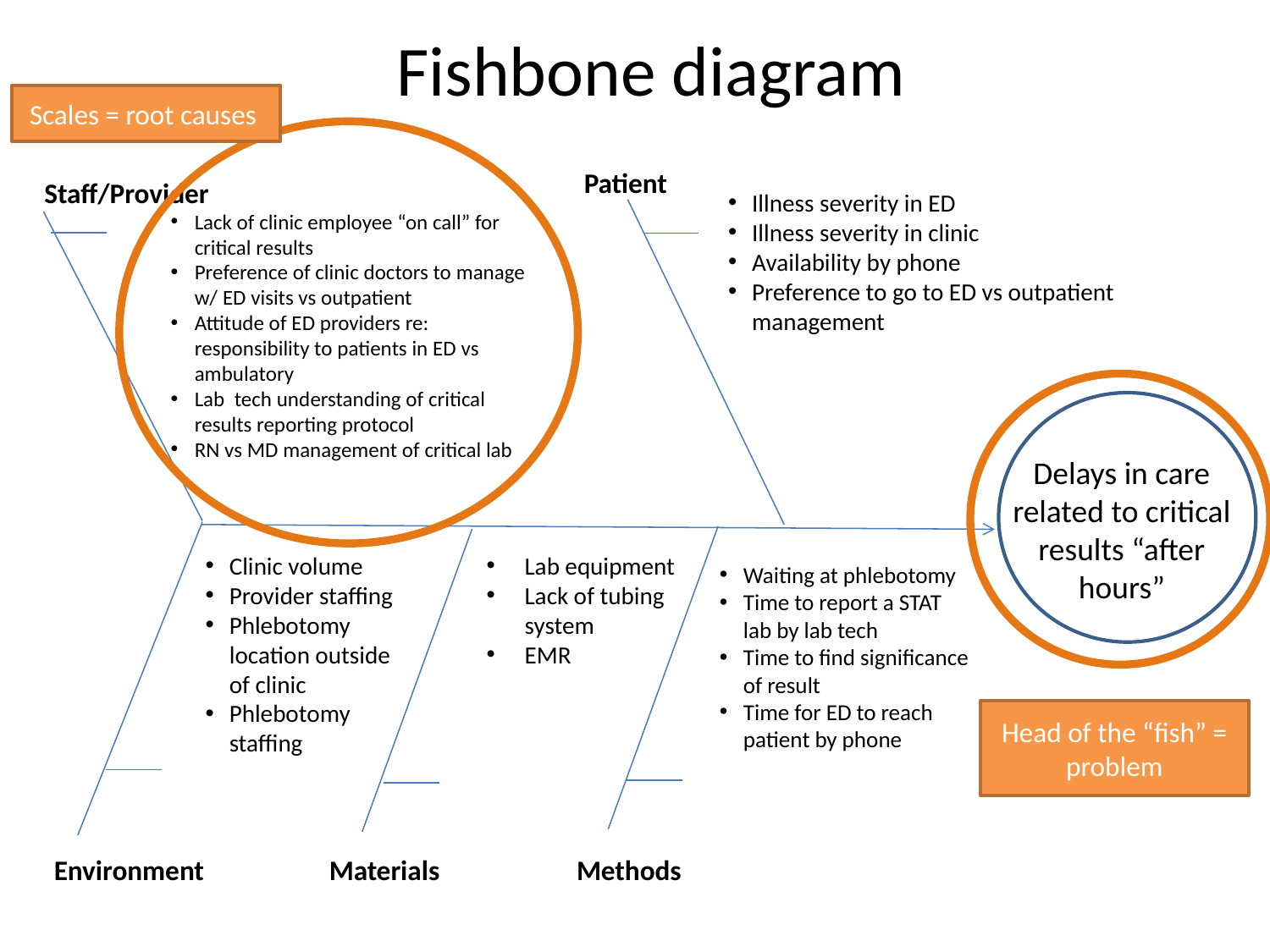

# Fishbone diagram
Scales = root causes
Patient
Staff/Provider
Illness severity in ED
Illness severity in clinic
Availability by phone
Preference to go to ED vs outpatient management
Lack of clinic employee “on call” for critical results
Preference of clinic doctors to manage w/ ED visits vs outpatient
Attitude of ED providers re: responsibility to patients in ED vs ambulatory
Lab tech understanding of critical results reporting protocol
RN vs MD management of critical lab
Delays in care related to critical results “after hours”
Clinic volume
Provider staffing
Phlebotomy location outside of clinic
Phlebotomy staffing
Lab equipment
Lack of tubing system
EMR
Waiting at phlebotomy
Time to report a STAT lab by lab tech
Time to find significance of result
Time for ED to reach patient by phone
Head of the “fish” = problem
Materials
Environment
Methods

## Slide 10
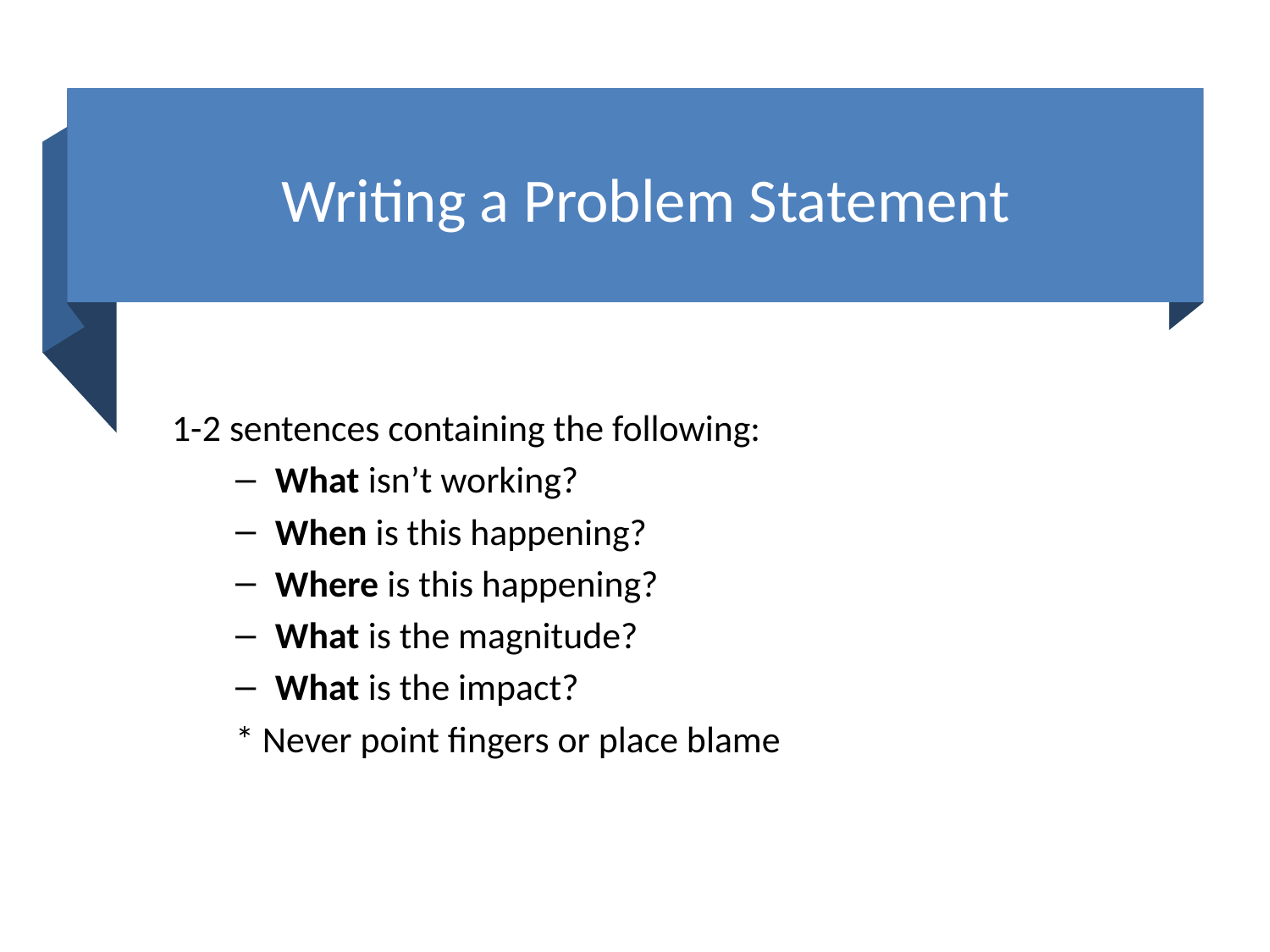

# Writing a Problem Statement
1-2 sentences containing the following:
What isn’t working?
When is this happening?
Where is this happening?
What is the magnitude?
What is the impact?
* Never point fingers or place blame

## Slide 11
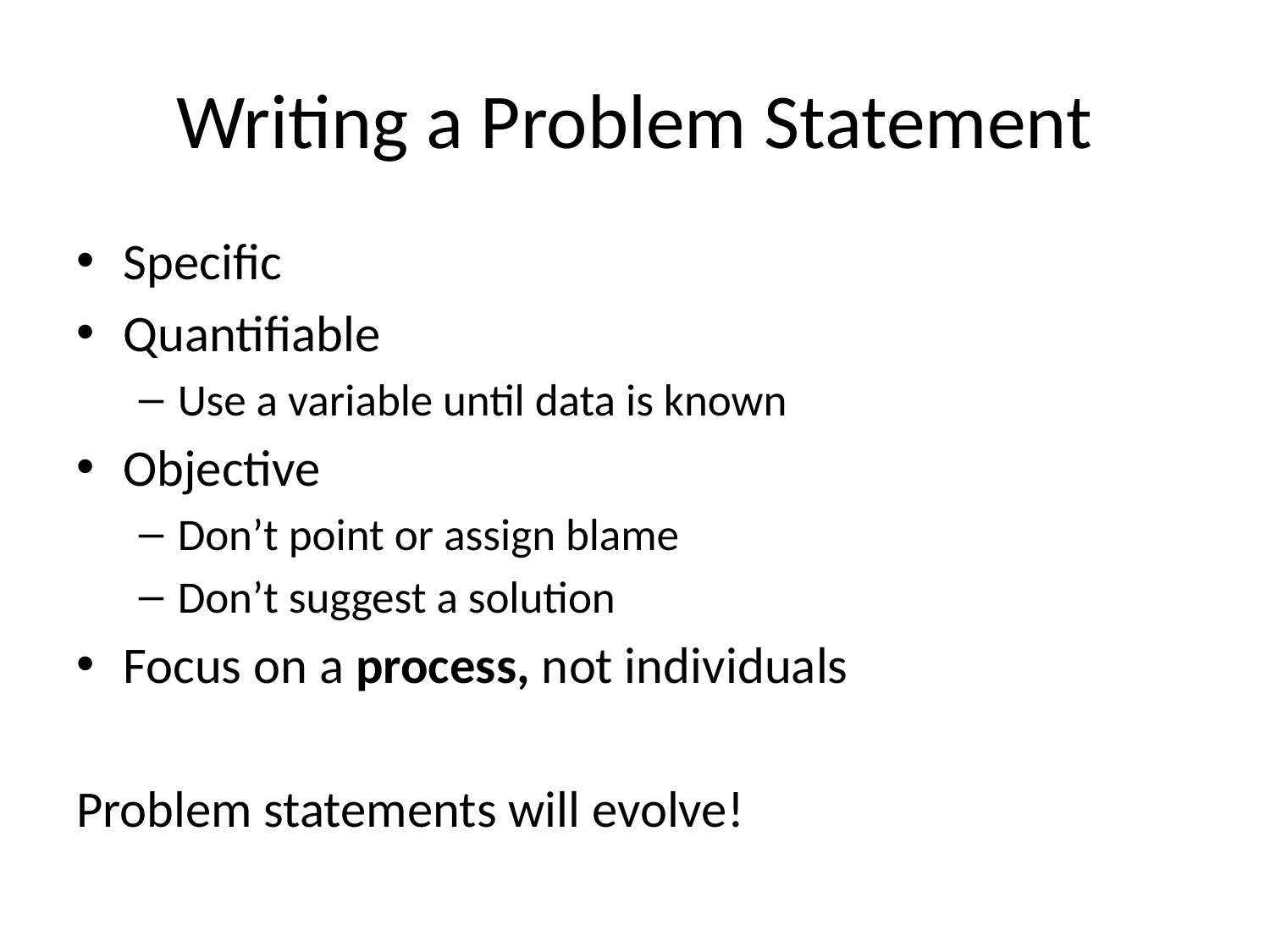

# Writing a Problem Statement
Specific
Quantifiable
Use a variable until data is known
Objective
Don’t point or assign blame
Don’t suggest a solution
Focus on a process, not individuals
Problem statements will evolve!

## Slide 12
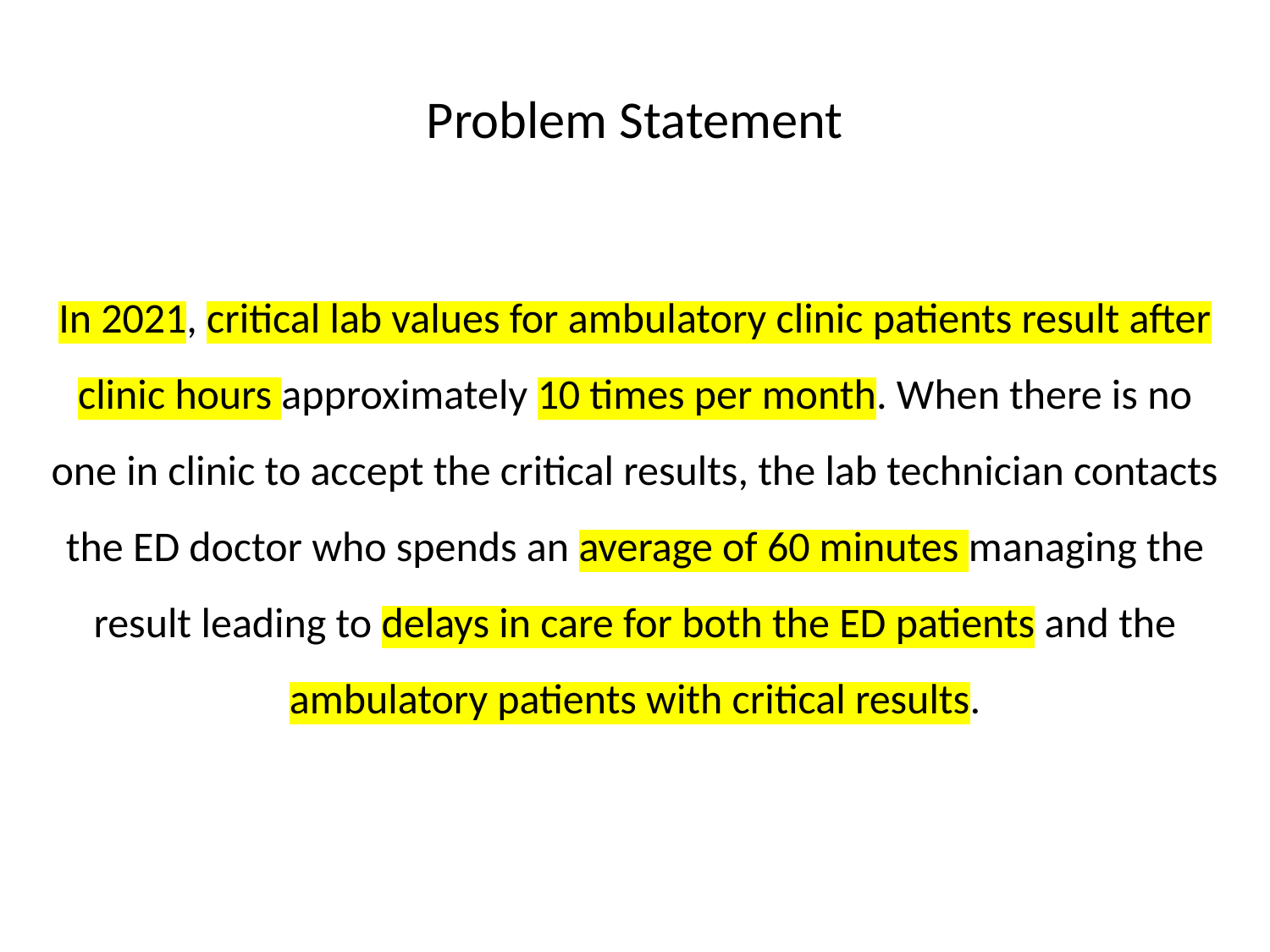

# Problem Statement
In 2021, critical lab values for ambulatory clinic patients result after clinic hours approximately 10 times per month. When there is no one in clinic to accept the critical results, the lab technician contacts the ED doctor who spends an average of 60 minutes managing the result leading to delays in care for both the ED patients and the ambulatory patients with critical results.

## Slide 13
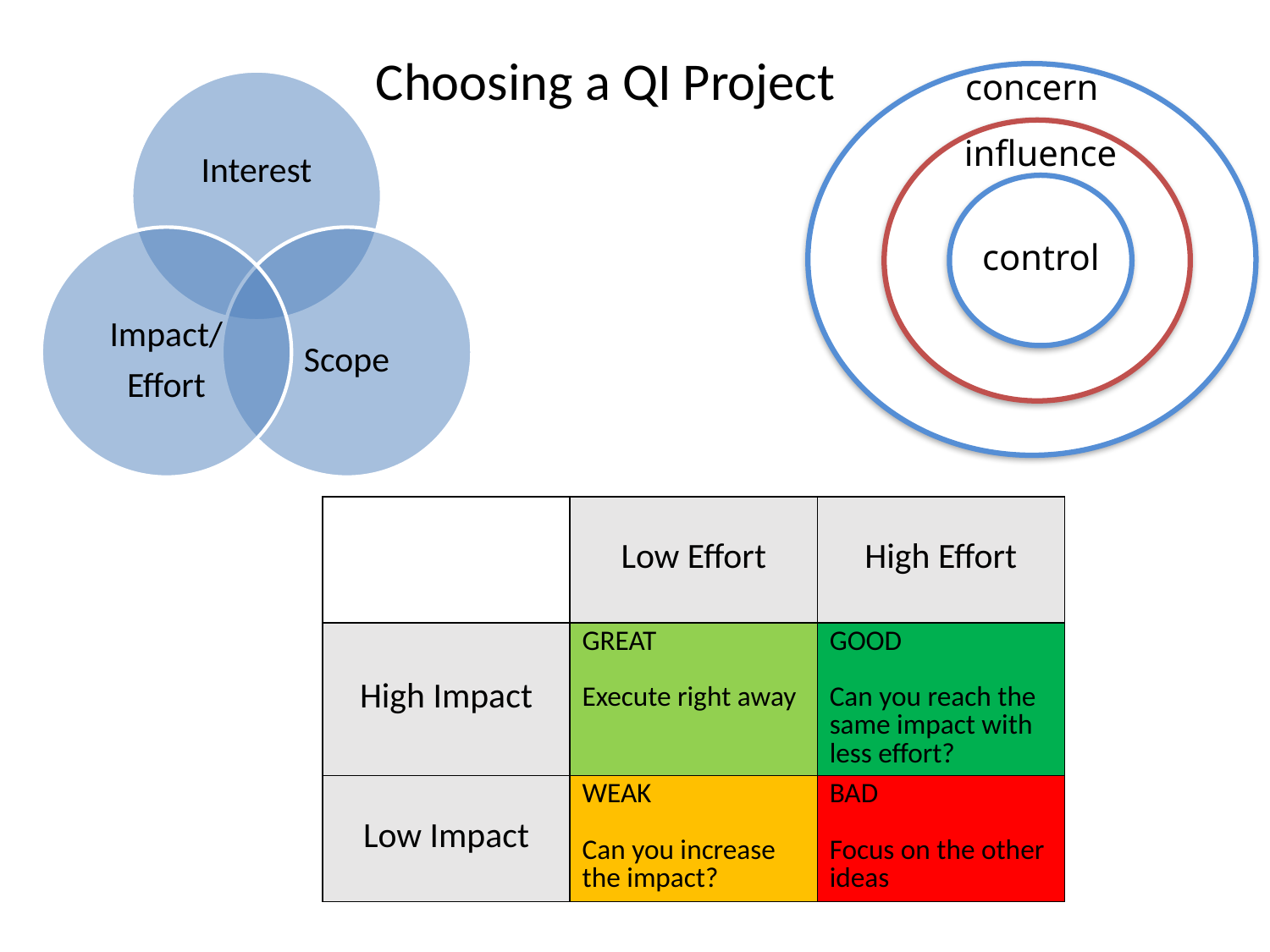

# Choosing a QI Project
concern
influence
control
| ​ | Low Effort​ | High Effort​ |
| --- | --- | --- |
| High Impact​ | ​GREAT Execute right away | ​GOOD Can you reach the same impact with less effort? |
| Low Impact​ | ​WEAK Can you increase the impact? | ​BAD Focus on the other ideas |

## Slide 14
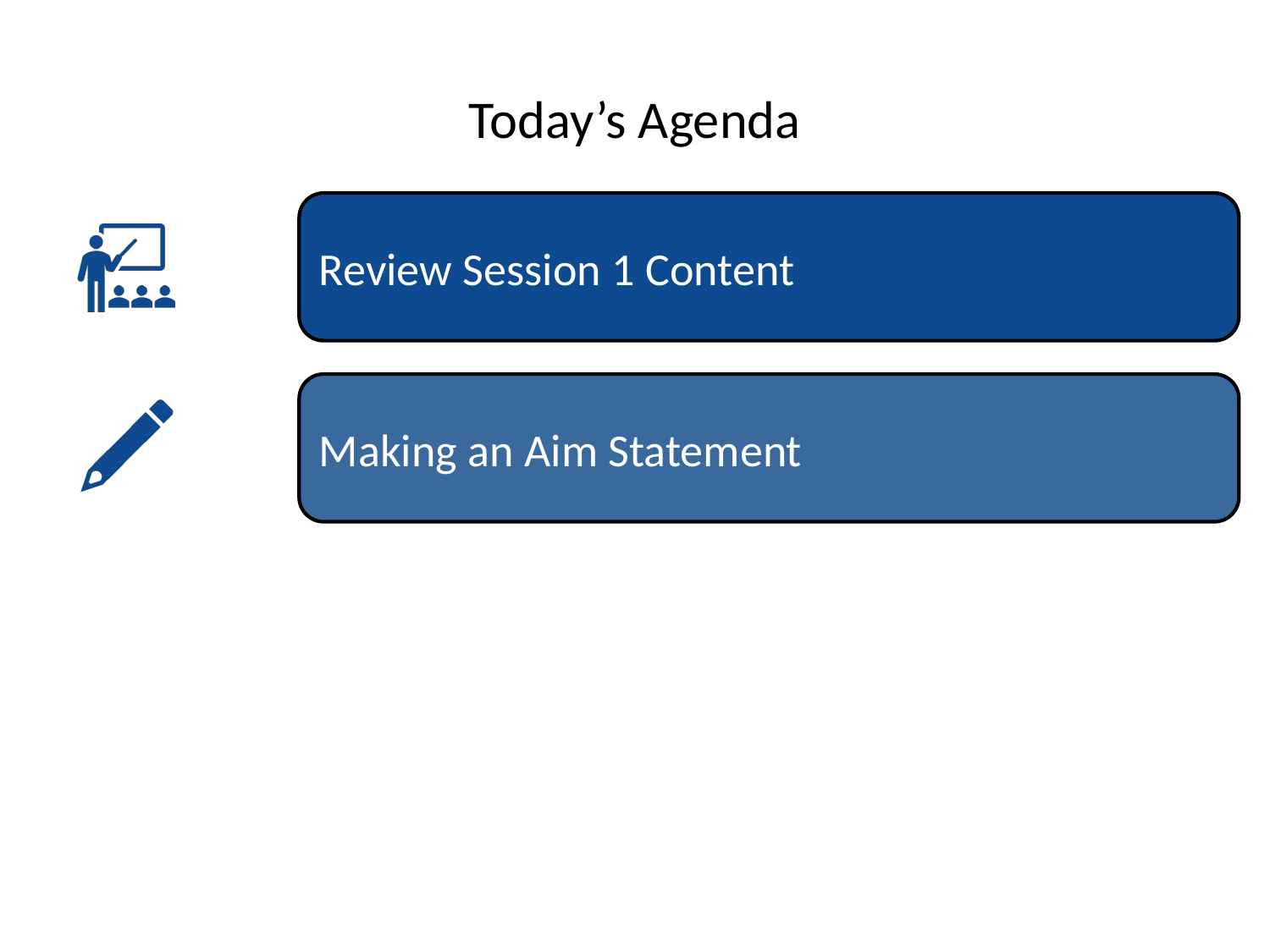

# Today’s Agenda
Review Session 1 Content
Making an Aim Statement

## Slide 15
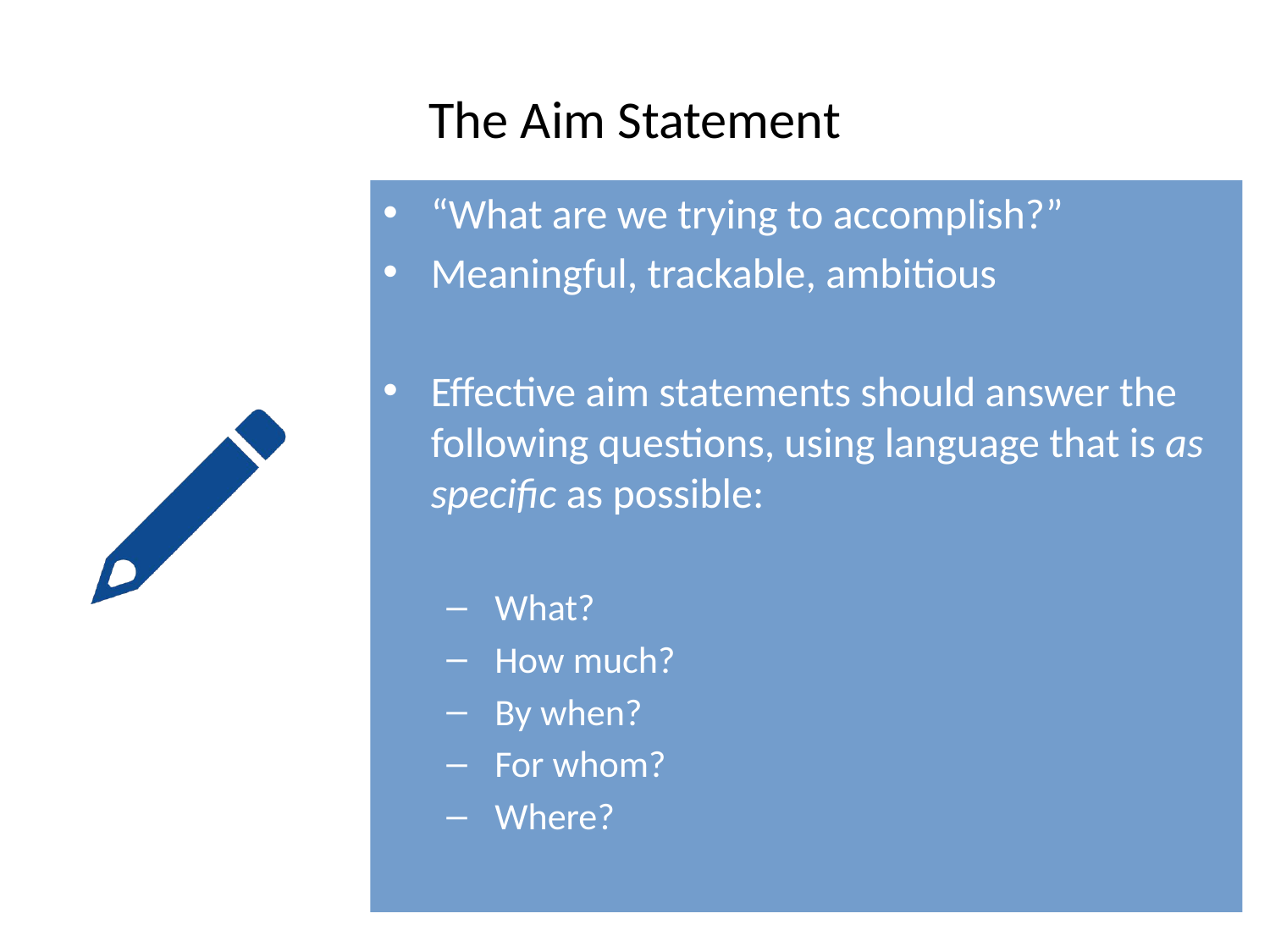

# The Aim Statement
“What are we trying to accomplish?”
Meaningful, trackable, ambitious
Effective aim statements should answer the following questions, using language that is as specific as possible:
 What?
 How much?
 By when?
 For whom?
 Where?

## Slide 16
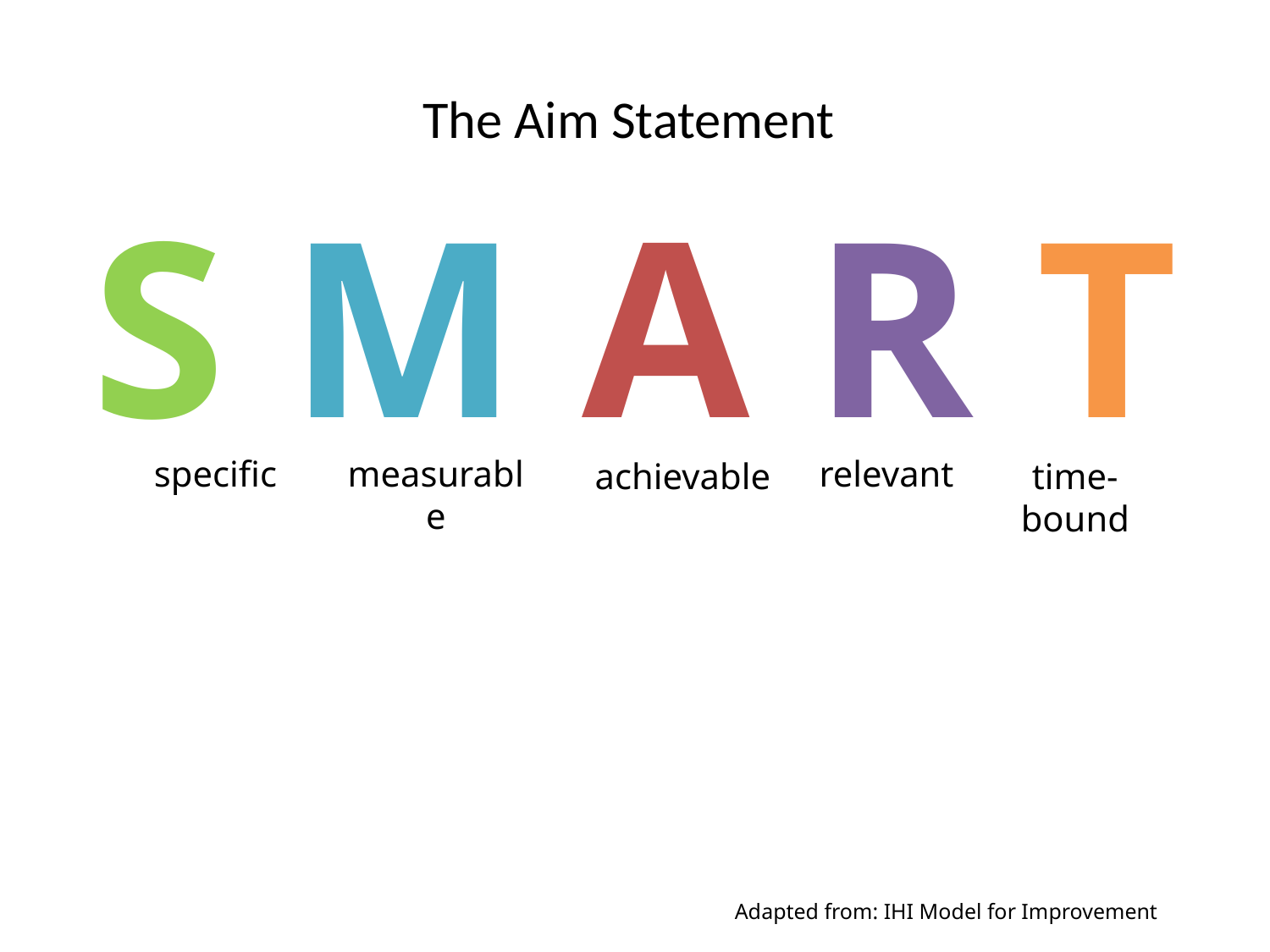

# The Aim Statement
S M A R T
specific
relevant
measurable
achievable
time-bound
Adapted from: IHI Model for Improvement

## Slide 17
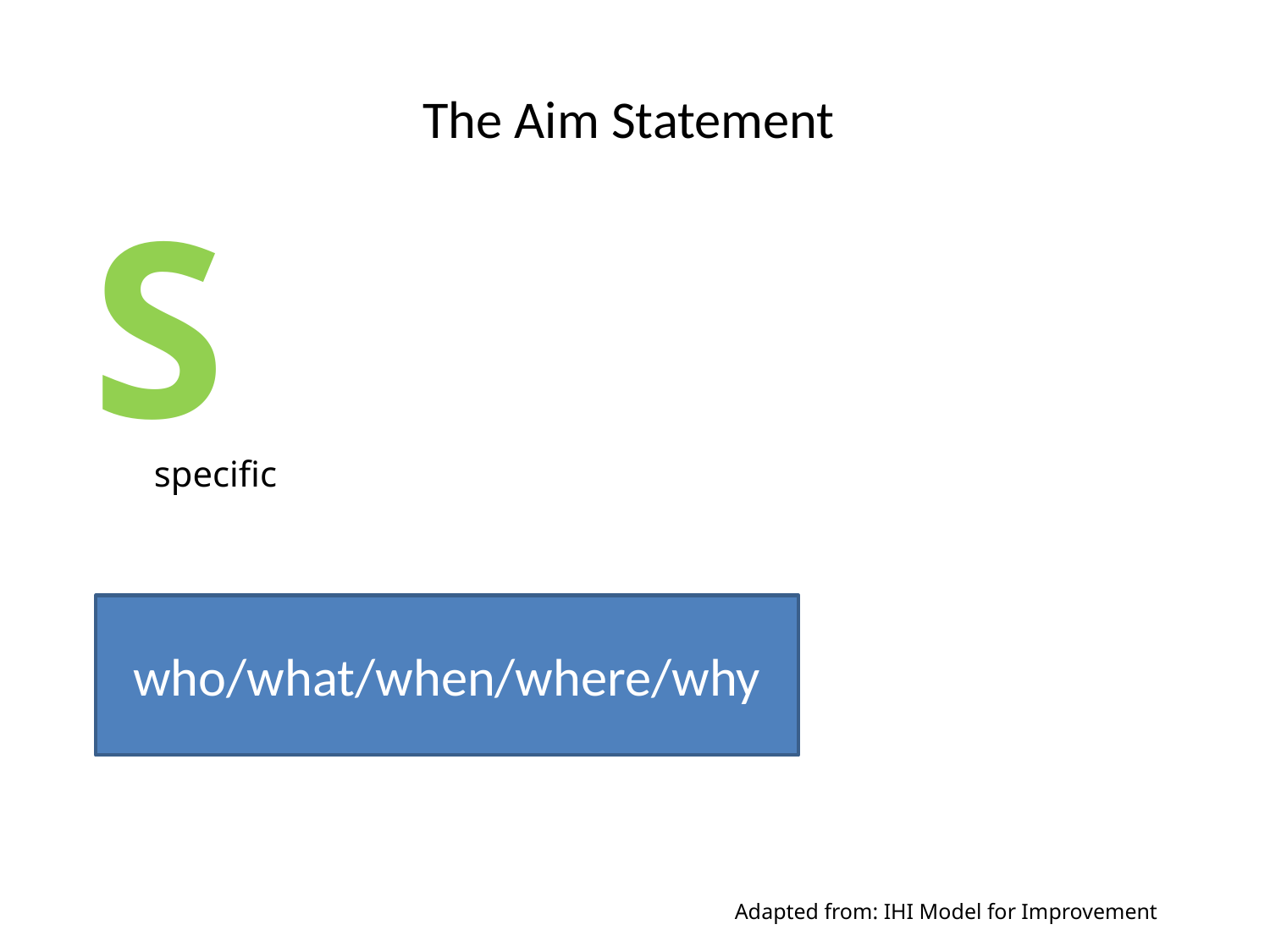

# The Aim Statement
S M A R T
specific
relevant
measurable
achievable
time-bound
who/what/when/where/why
Adapted from: IHI Model for Improvement

## Slide 18
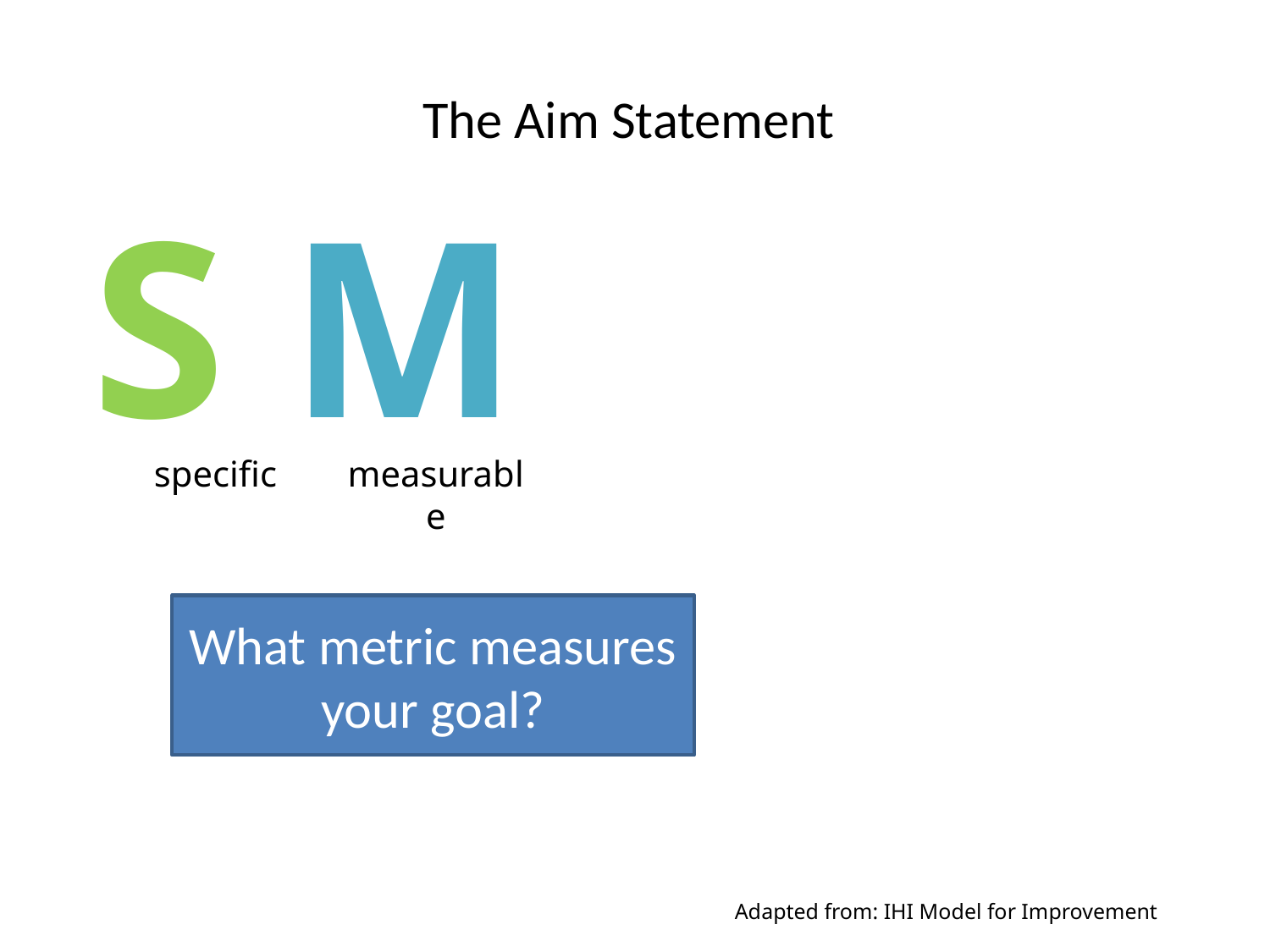

# The Aim Statement
S M A R T
specific
relevant
measurable
achievable
time-bound
What metric measures your goal?
Adapted from: IHI Model for Improvement

## Slide 19
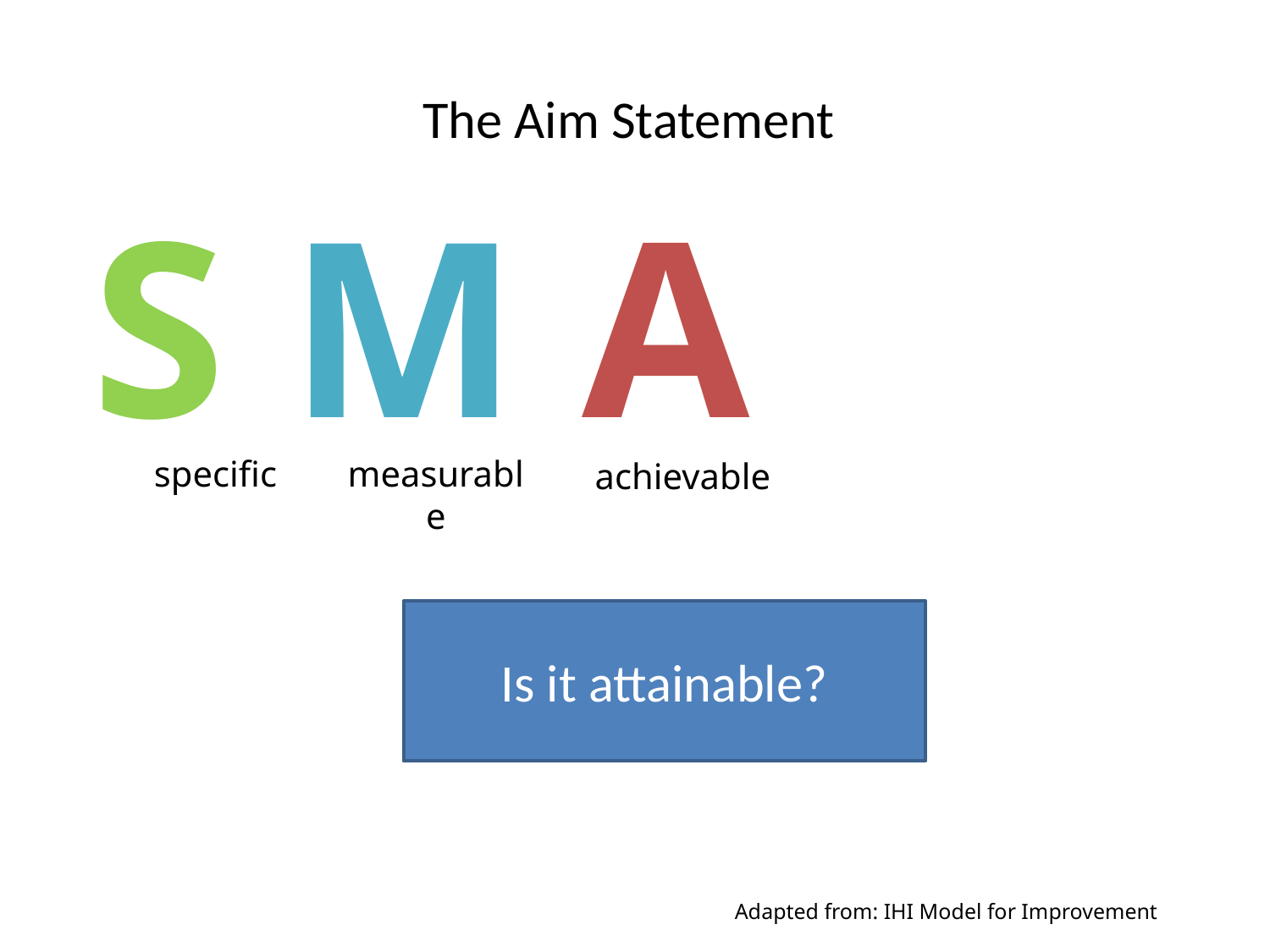

# The Aim Statement
S M A R T
specific
relevant
measurable
achievable
time-bound
Is it attainable?
Adapted from: IHI Model for Improvement

## Slide 20
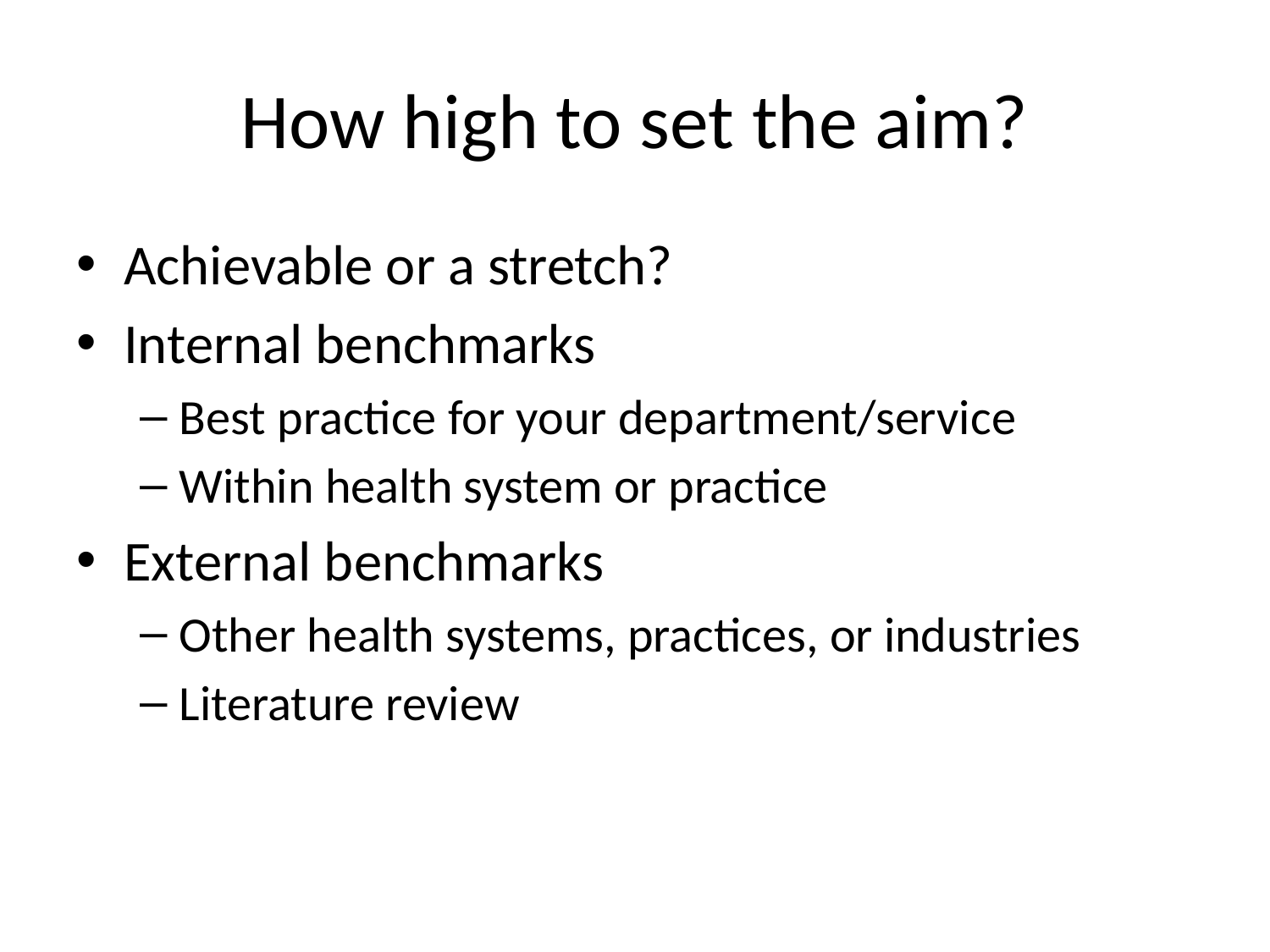

# How high to set the aim?
Achievable or a stretch?
Internal benchmarks
Best practice for your department/service
Within health system or practice
External benchmarks
Other health systems, practices, or industries
Literature review

## Slide 21
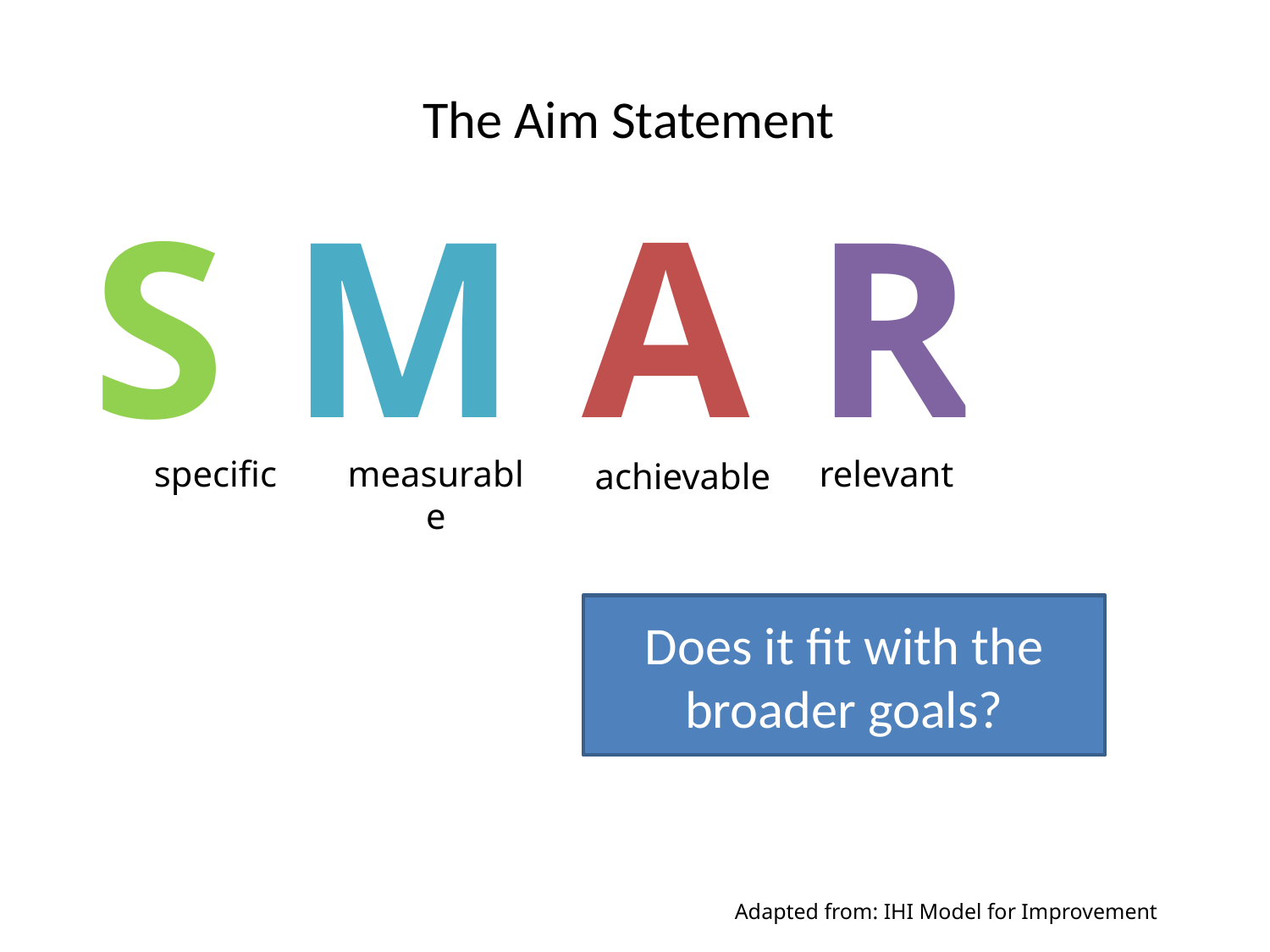

# The Aim Statement
S M A R T
specific
relevant
measurable
achievable
time-bound
Does it fit with the broader goals?
Adapted from: IHI Model for Improvement

## Slide 22
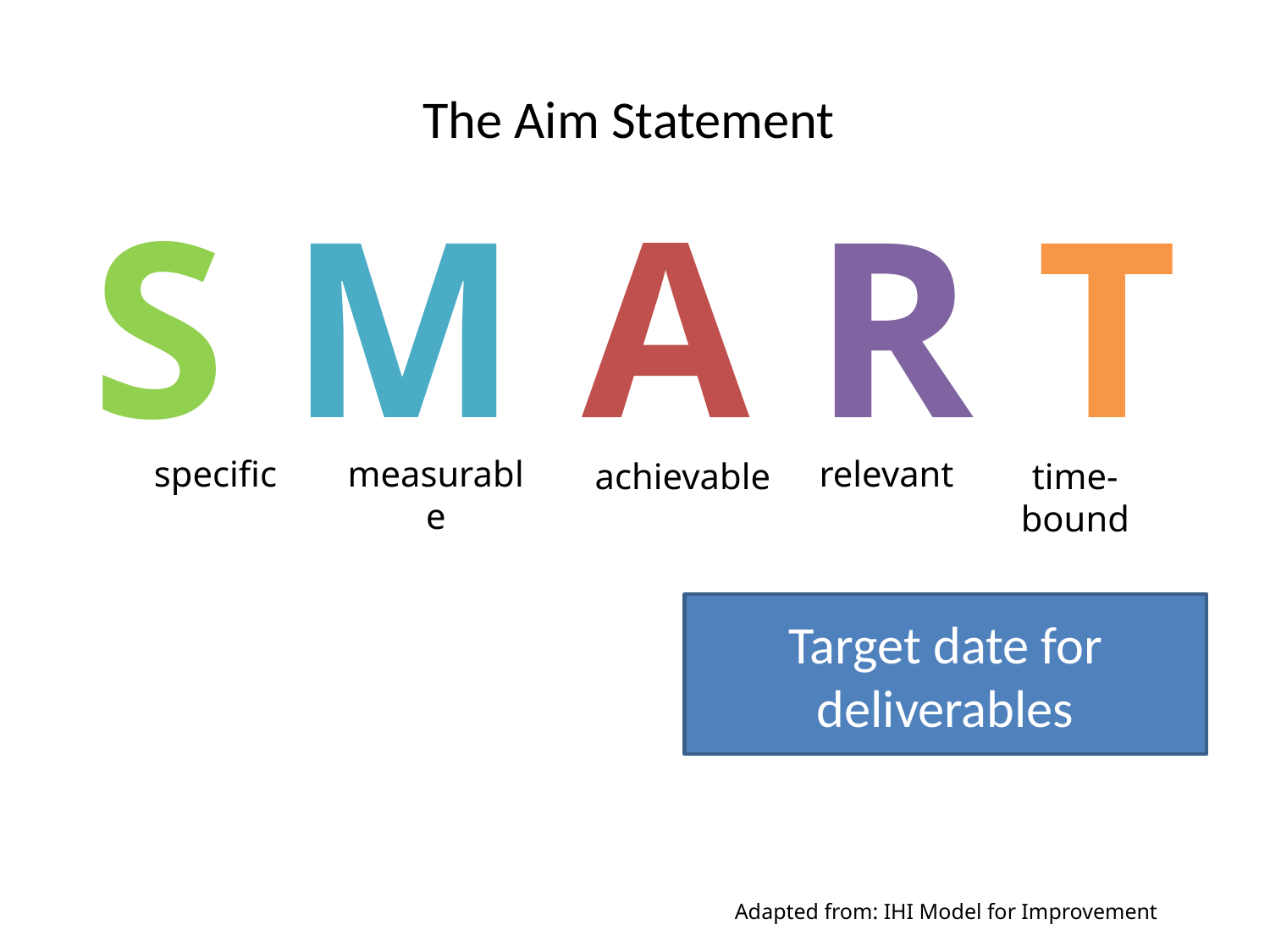

# The Aim Statement
S M A R T
specific
relevant
measurable
achievable
time-bound
Target date for deliverables
Adapted from: IHI Model for Improvement

## Slide 23
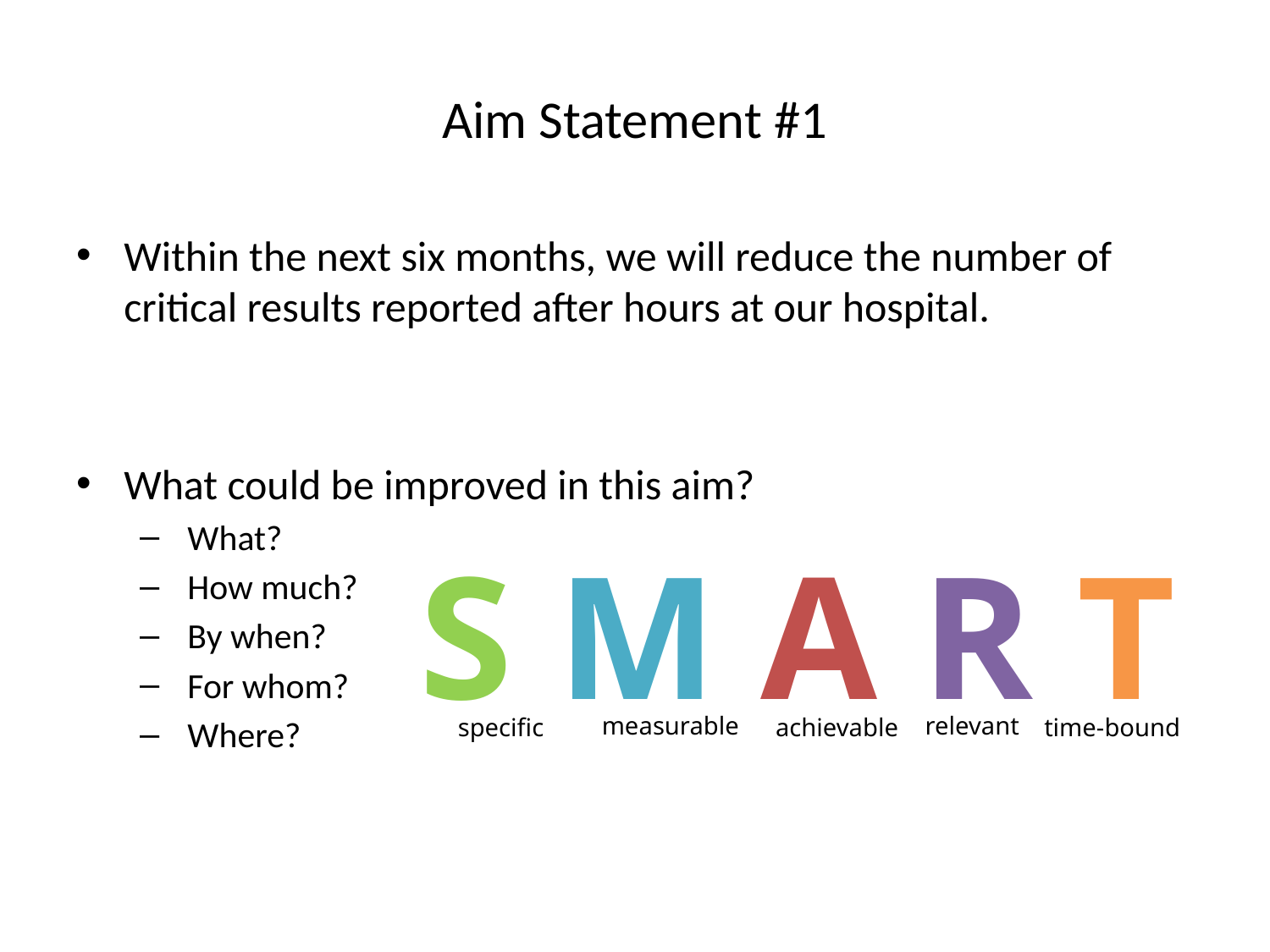

# Aim Statement #1
Within the next six months, we will reduce the number of critical results reported after hours at our hospital.
What could be improved in this aim?
 What?
 How much?
 By when?
 For whom?
 Where?
S M A R T
measurable
relevant
achievable
time-bound
specific

## Slide 24
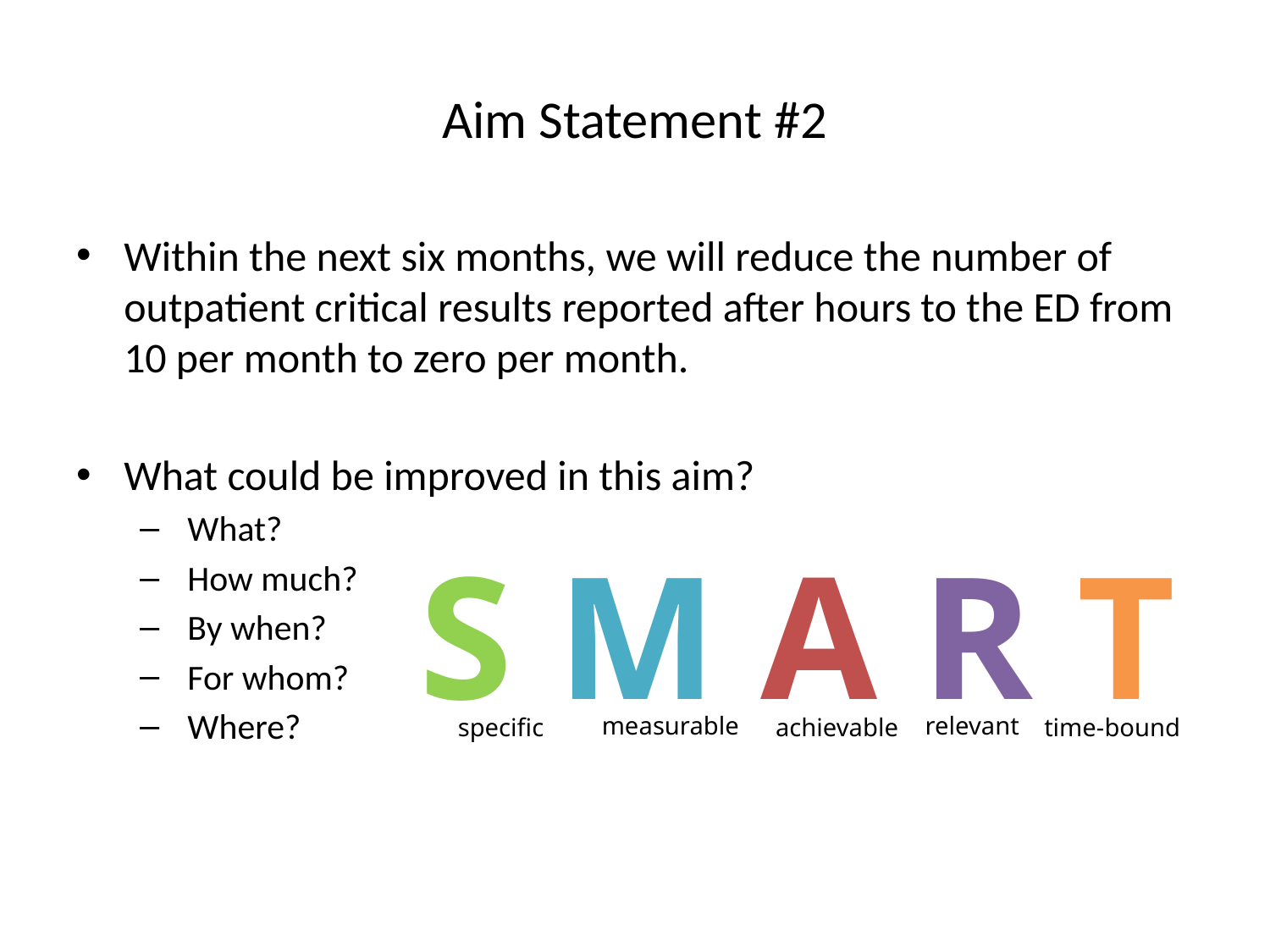

# Aim Statement #2
Within the next six months, we will reduce the number of outpatient critical results reported after hours to the ED from 10 per month to zero per month.
What could be improved in this aim?
 What?
 How much?
 By when?
 For whom?
 Where?
S M A R T
measurable
relevant
achievable
time-bound
specific

## Slide 25
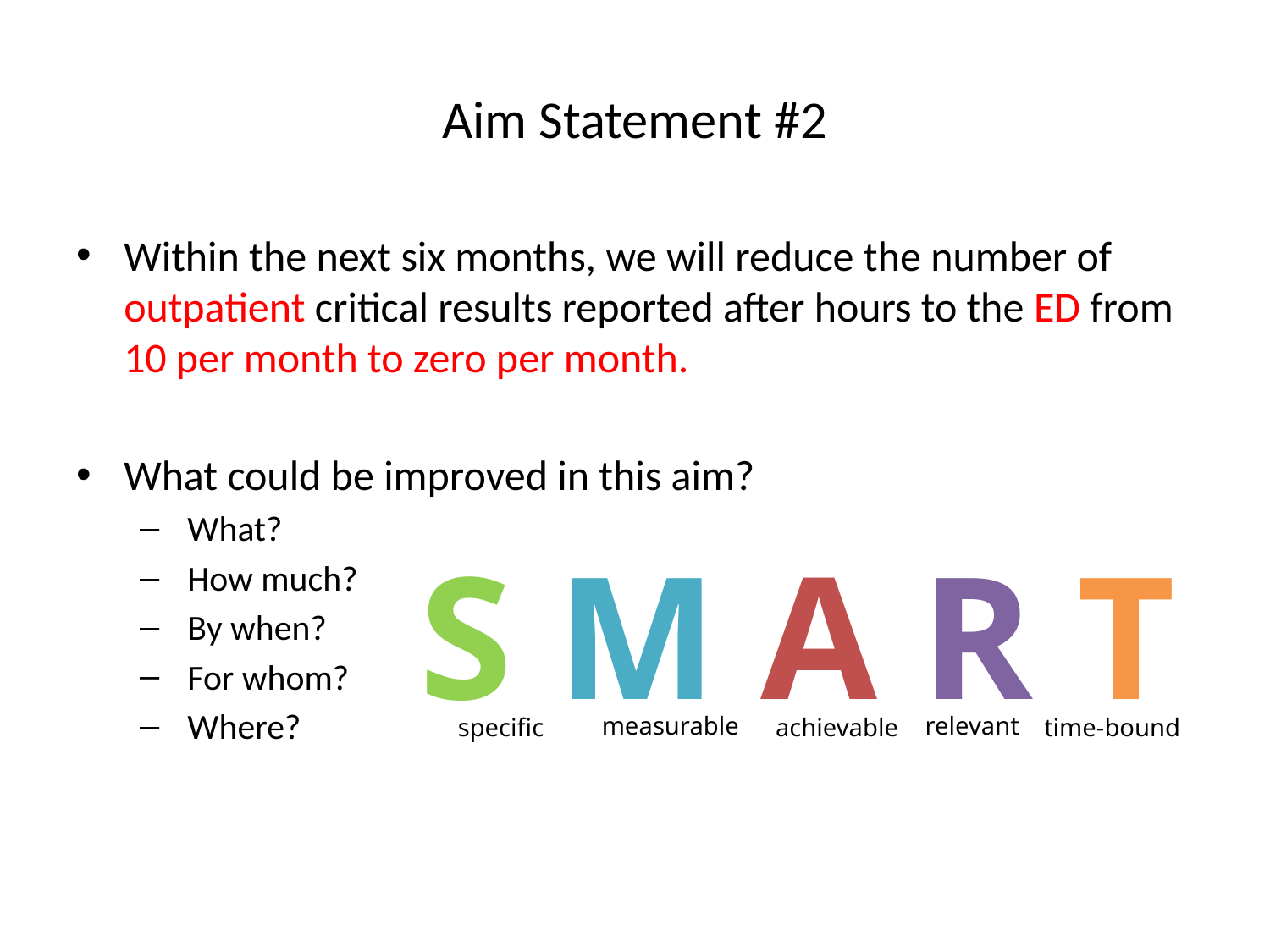

# Aim Statement #2
Within the next six months, we will reduce the number of outpatient critical results reported after hours to the ED from 10 per month to zero per month.
What could be improved in this aim?
 What?
 How much?
 By when?
 For whom?
 Where?
S M A R T
measurable
relevant
achievable
time-bound
specific

## Slide 26
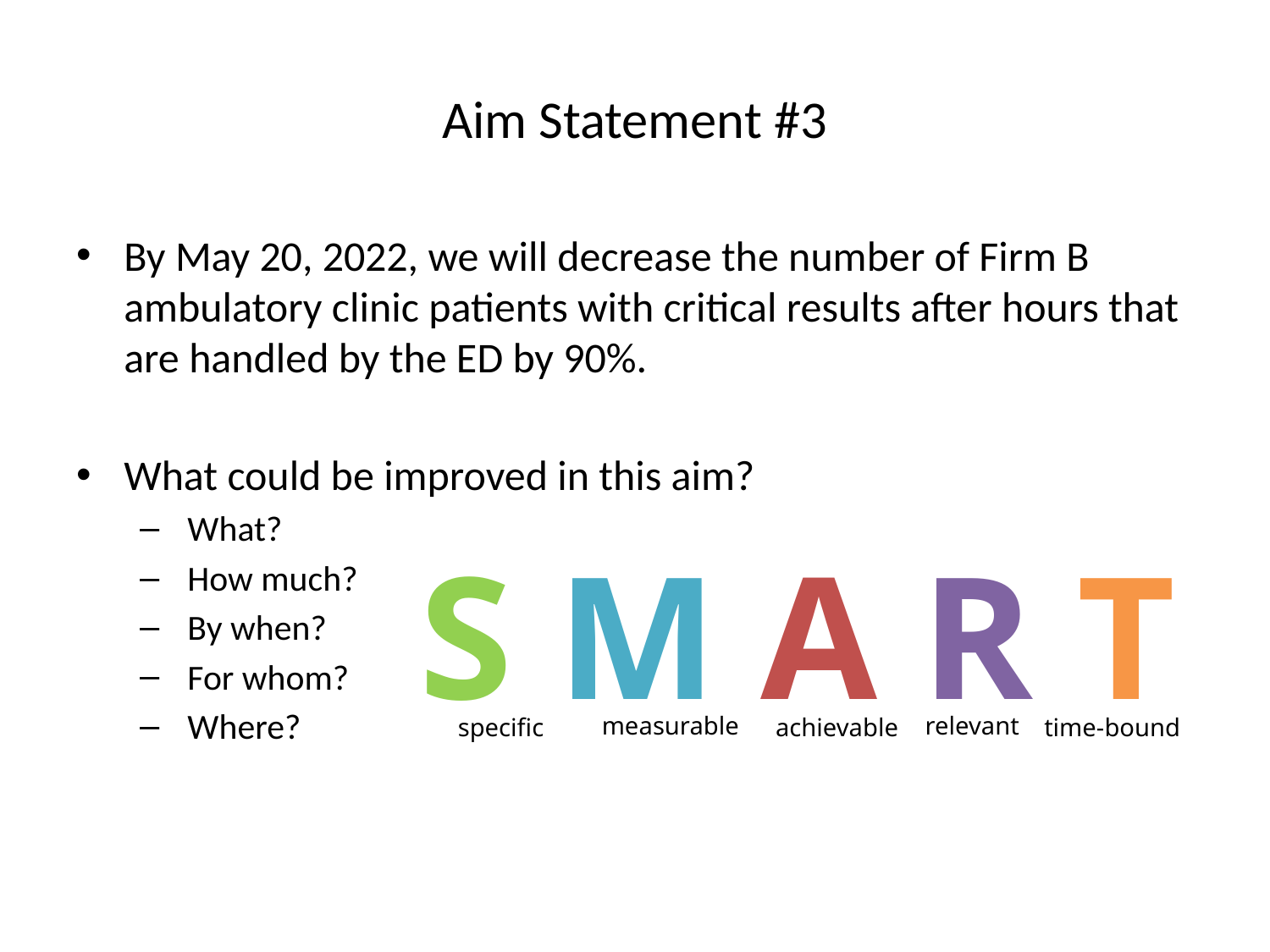

# Aim Statement #3
By May 20, 2022, we will decrease the number of Firm B ambulatory clinic patients with critical results after hours that are handled by the ED by 90%.
What could be improved in this aim?
 What?
 How much?
 By when?
 For whom?
 Where?
S M A R T
measurable
relevant
achievable
time-bound
specific

## Slide 27
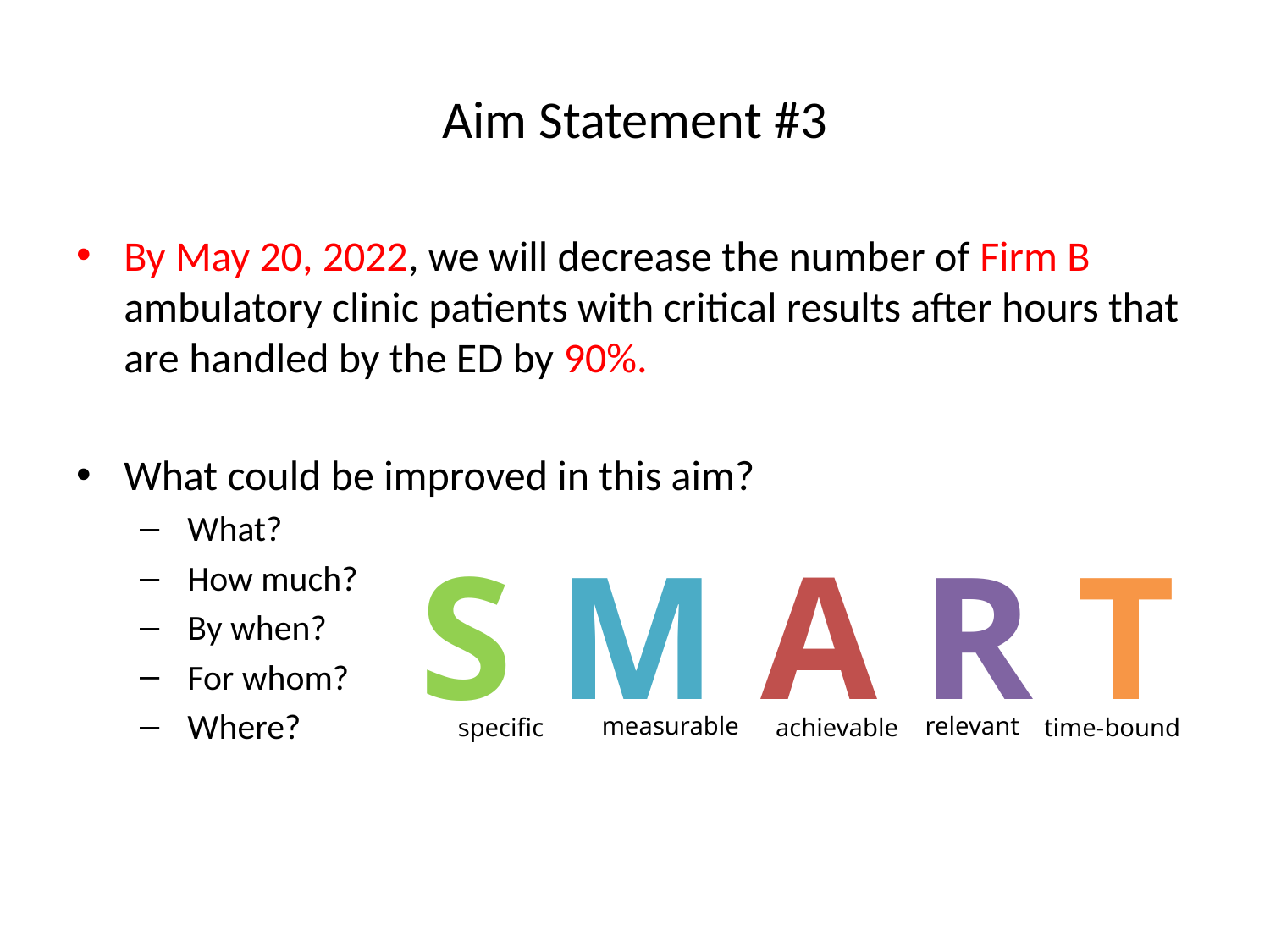

# Aim Statement #3
By May 20, 2022, we will decrease the number of Firm B ambulatory clinic patients with critical results after hours that are handled by the ED by 90%.
What could be improved in this aim?
 What?
 How much?
 By when?
 For whom?
 Where?
S M A R T
measurable
relevant
achievable
time-bound
specific

## Slide 28
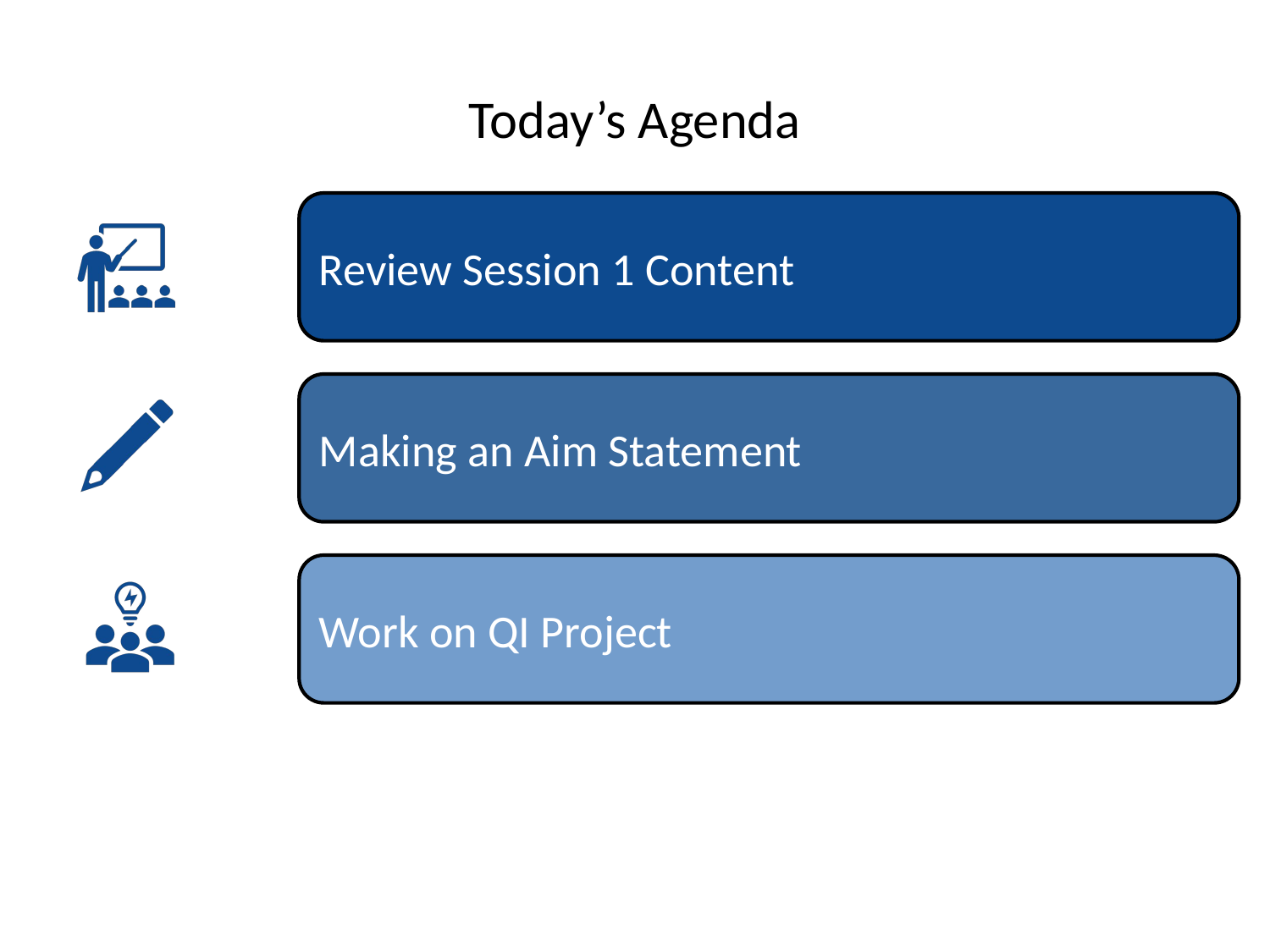

# Today’s Agenda
Review Session 1 Content
Making an Aim Statement
Work on QI Project

## Slide 29
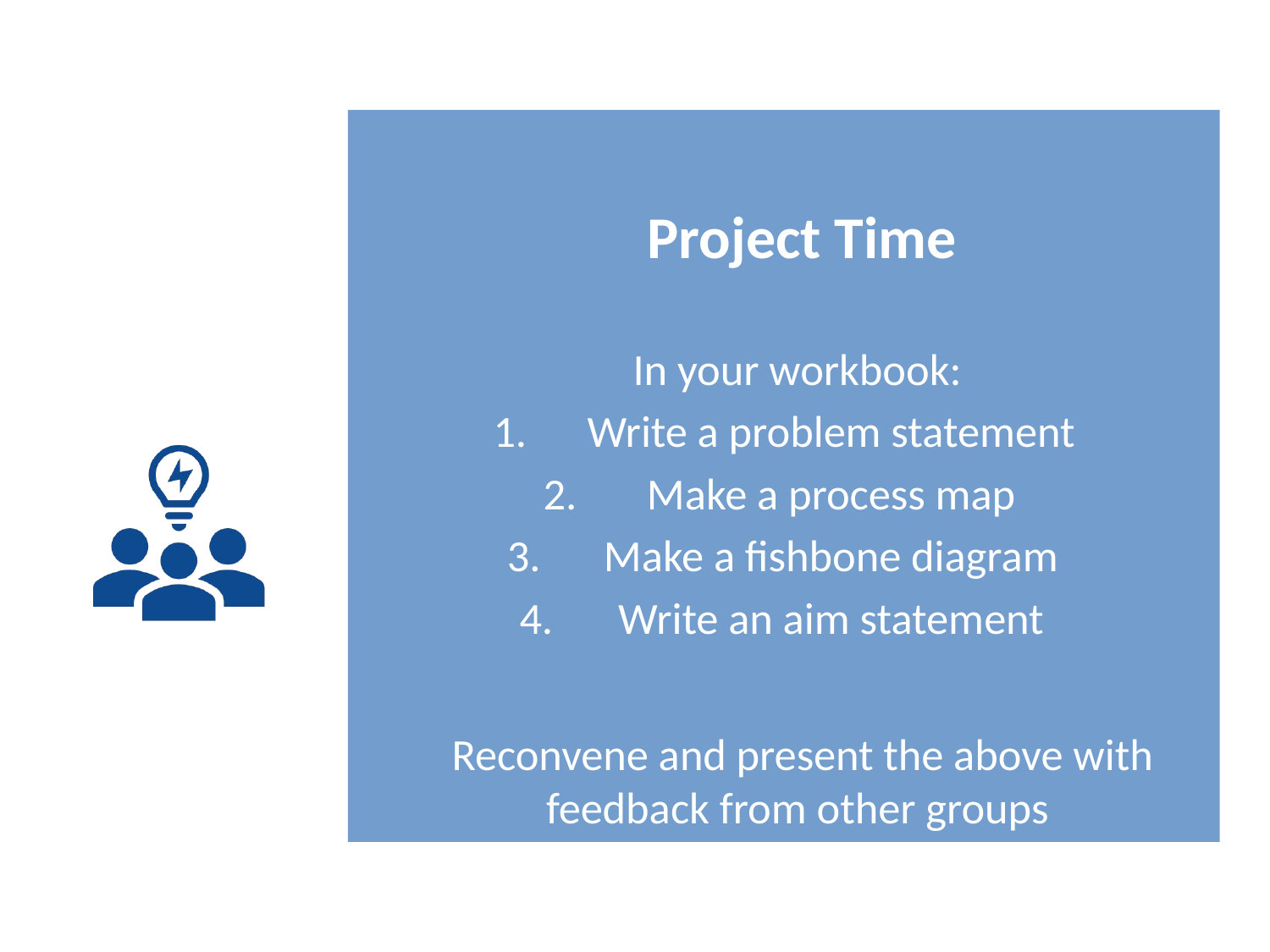

Project Time
In your workbook:
Write a problem statement
Make a process map
Make a fishbone diagram
Write an aim statement
Reconvene and present the above with feedback from other groups

## Slide 30
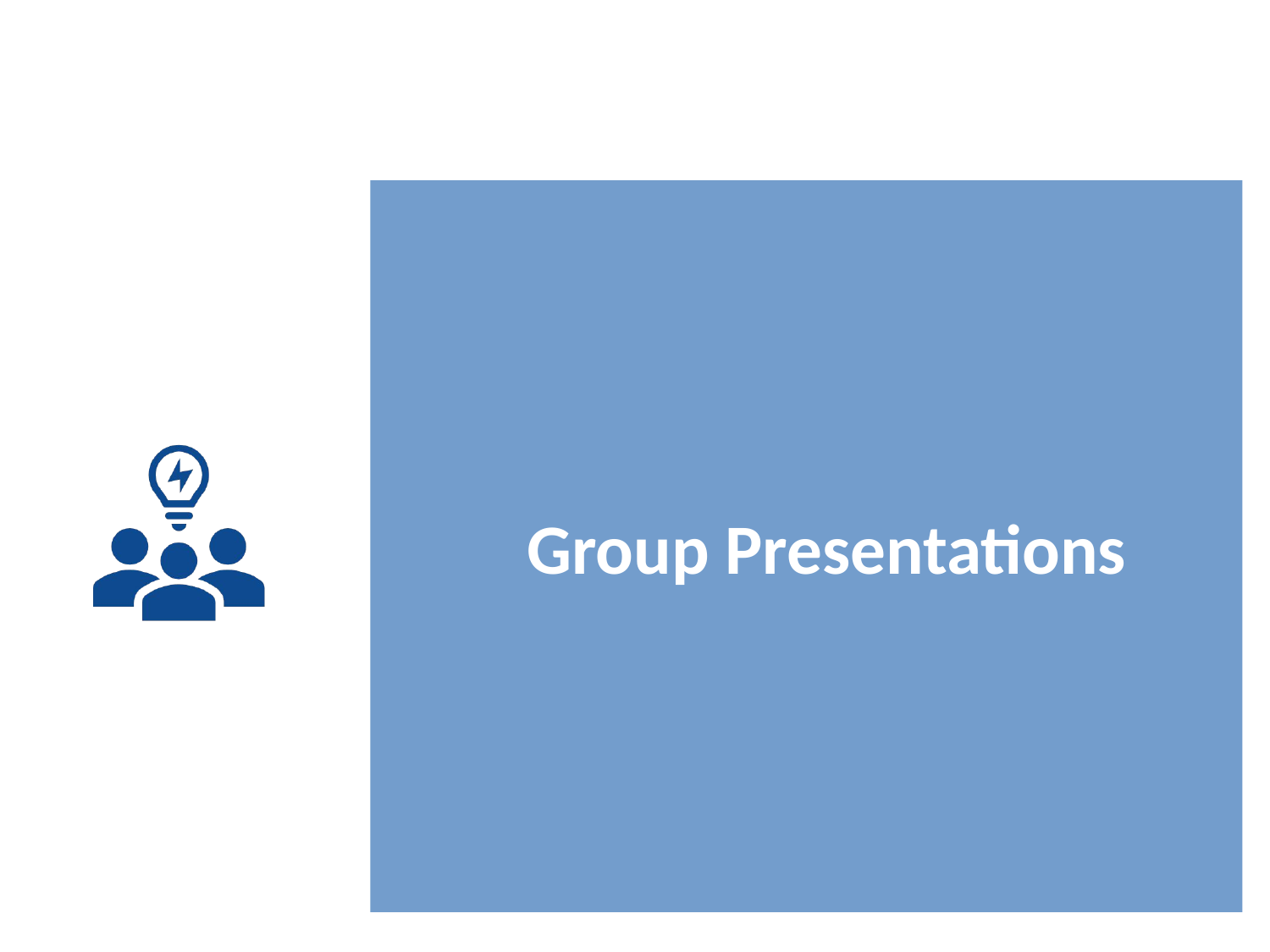

#
Group Presentations

## Slide 31
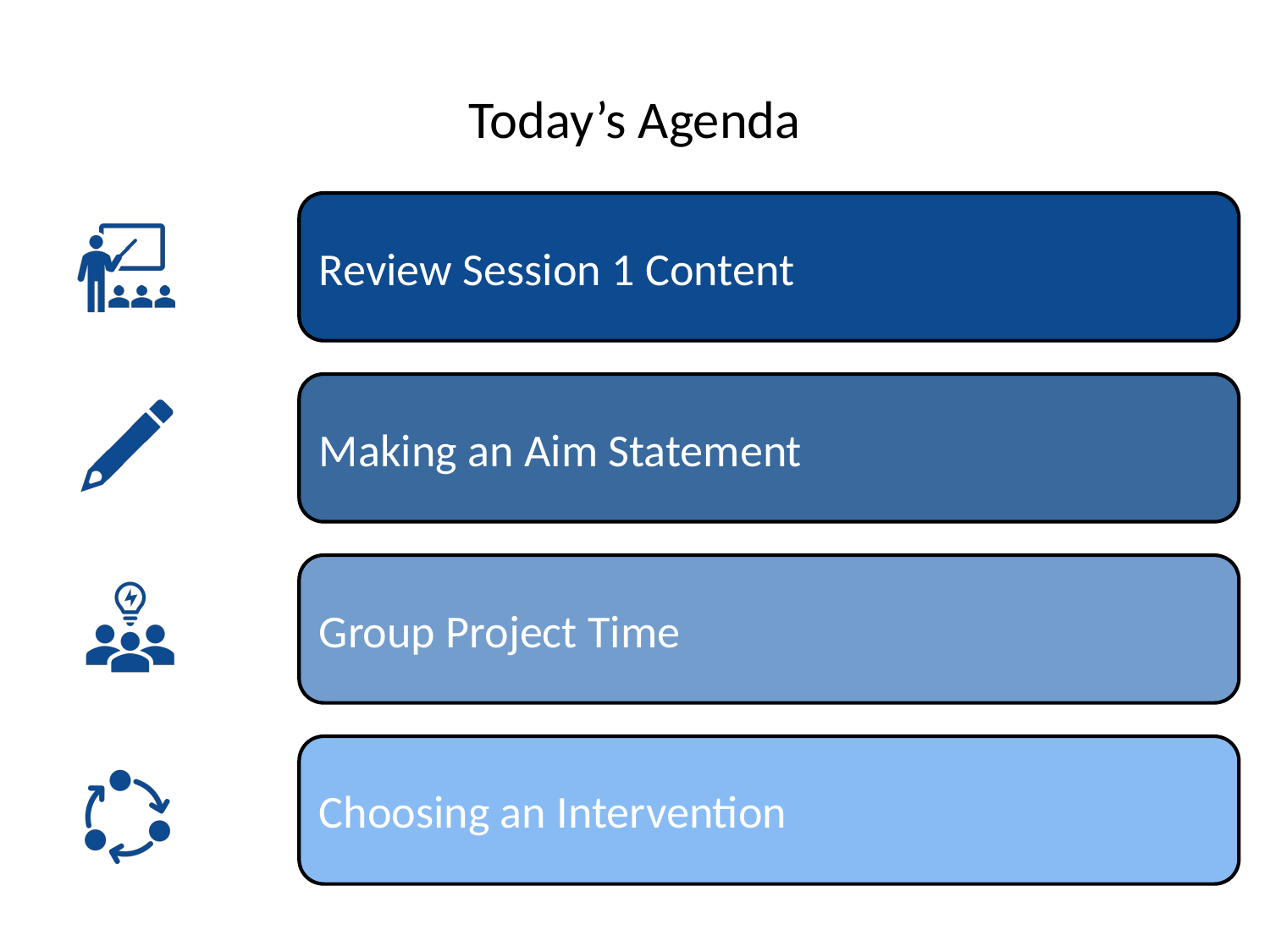

# Today’s Agenda
Review Session 1 Content
Making an Aim Statement
Group Project Time
Choosing an Intervention

## Slide 32
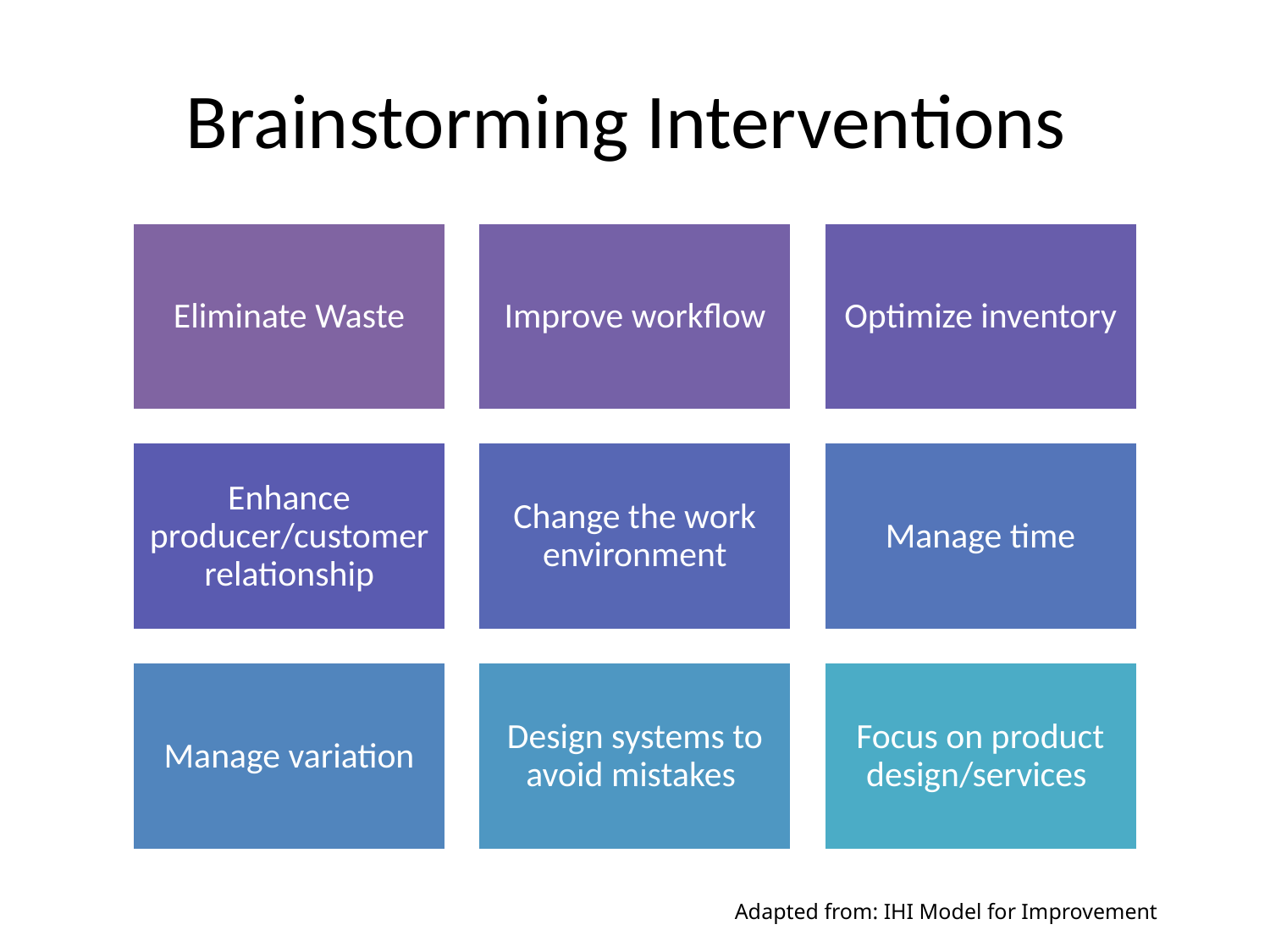

# Brainstorming Interventions
Adapted from: IHI Model for Improvement

## Slide 33
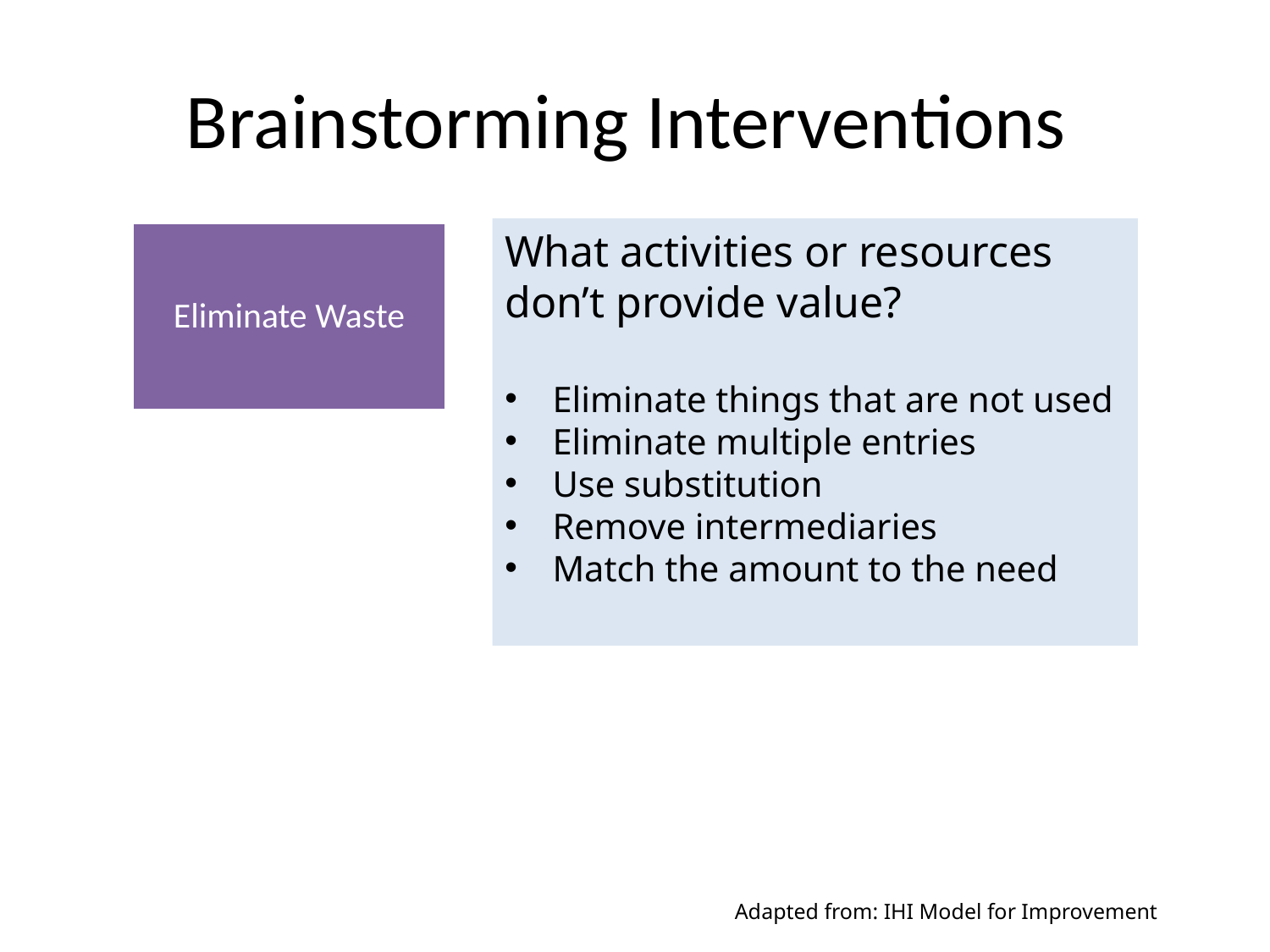

# Brainstorming Interventions
What activities or resources don’t provide value?
Eliminate things that are not used
Eliminate multiple entries
Use substitution
Remove intermediaries
Match the amount to the need
Adapted from: IHI Model for Improvement

## Slide 34
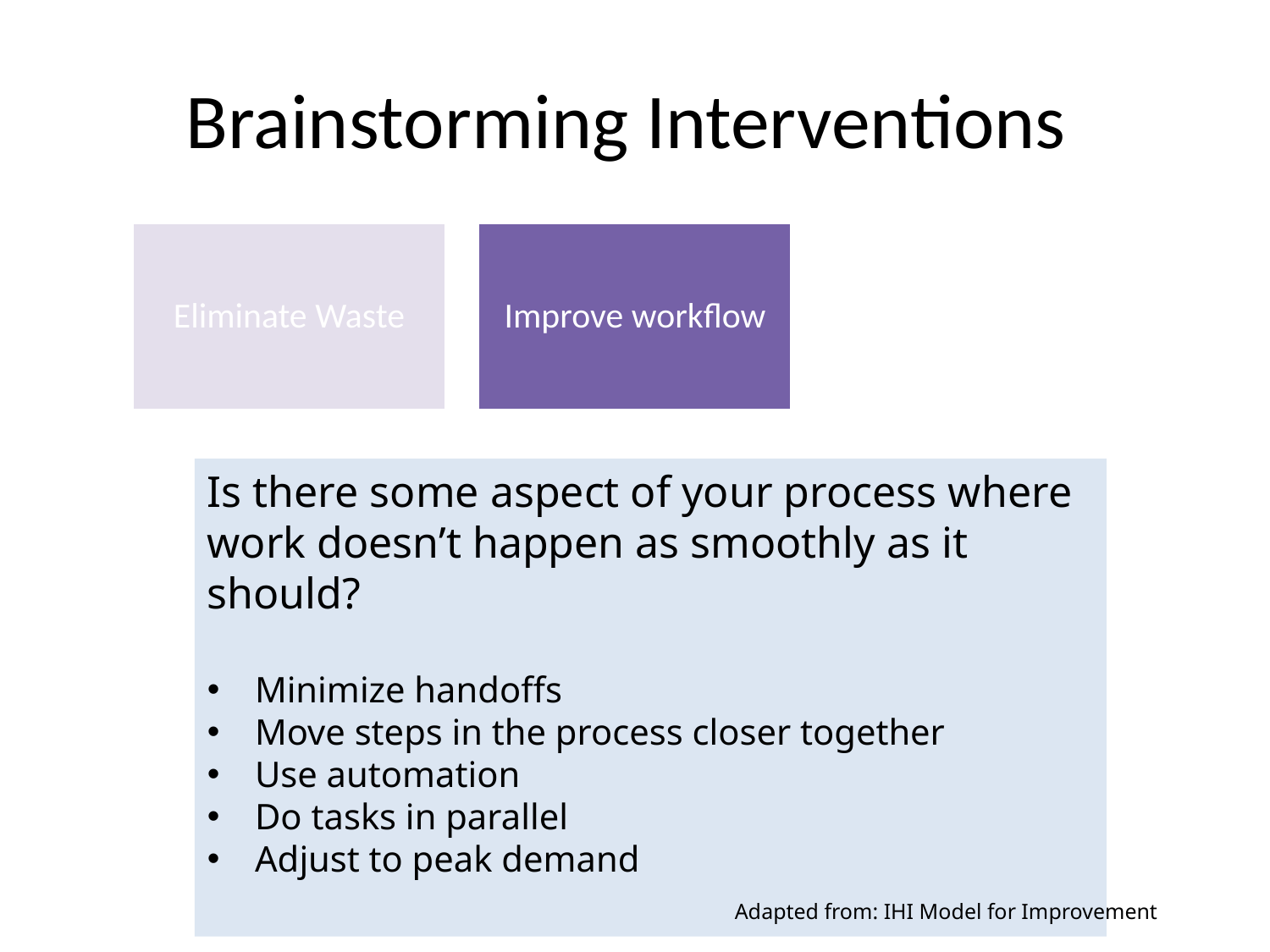

# Brainstorming Interventions
Is there some aspect of your process where work doesn’t happen as smoothly as it should?
Minimize handoffs
Move steps in the process closer together
Use automation
Do tasks in parallel
Adjust to peak demand
Adapted from: IHI Model for Improvement

## Slide 35
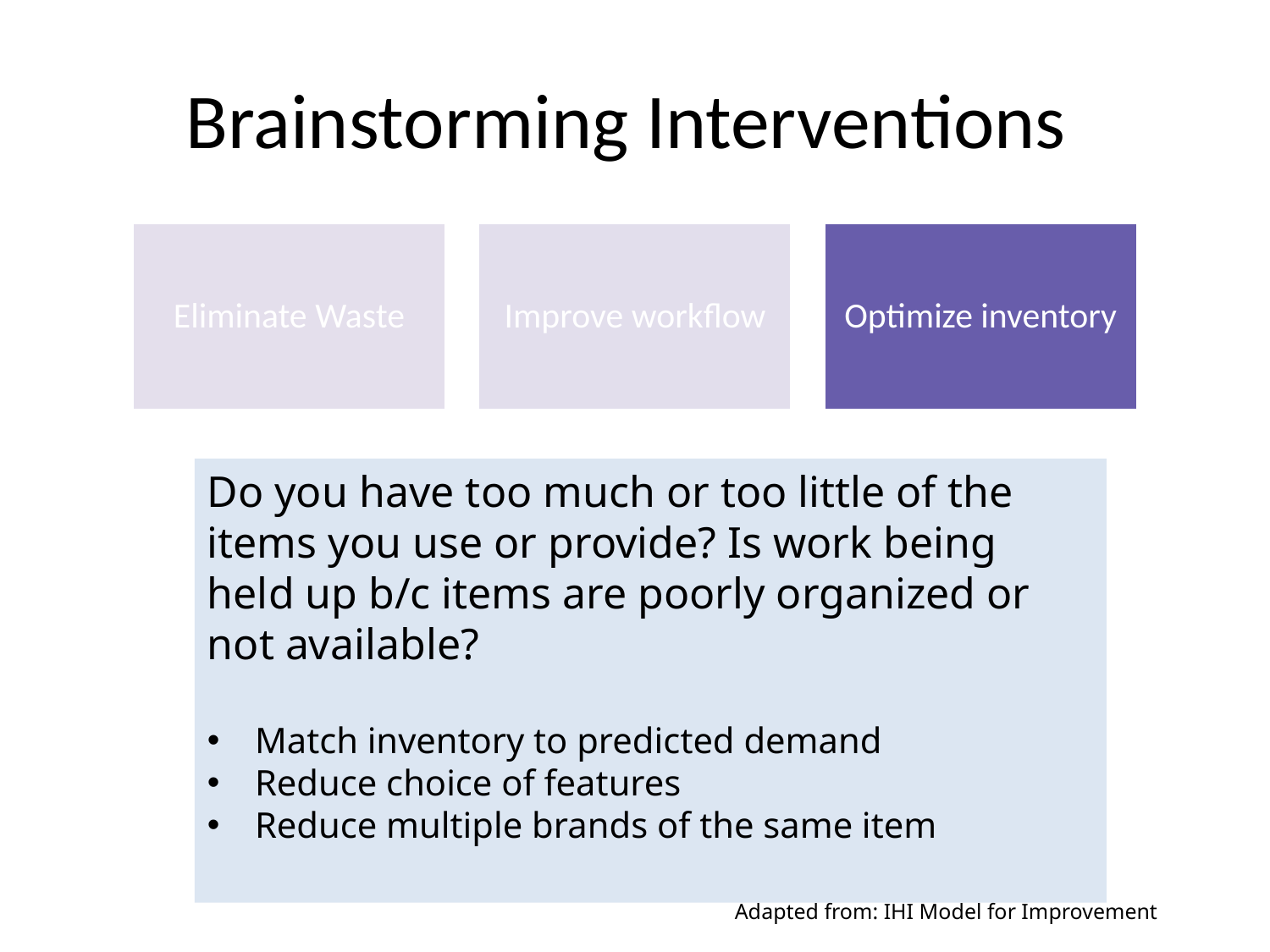

# Brainstorming Interventions
Do you have too much or too little of the items you use or provide? Is work being held up b/c items are poorly organized or not available?
Match inventory to predicted demand
Reduce choice of features
Reduce multiple brands of the same item
Adapted from: IHI Model for Improvement

## Slide 36
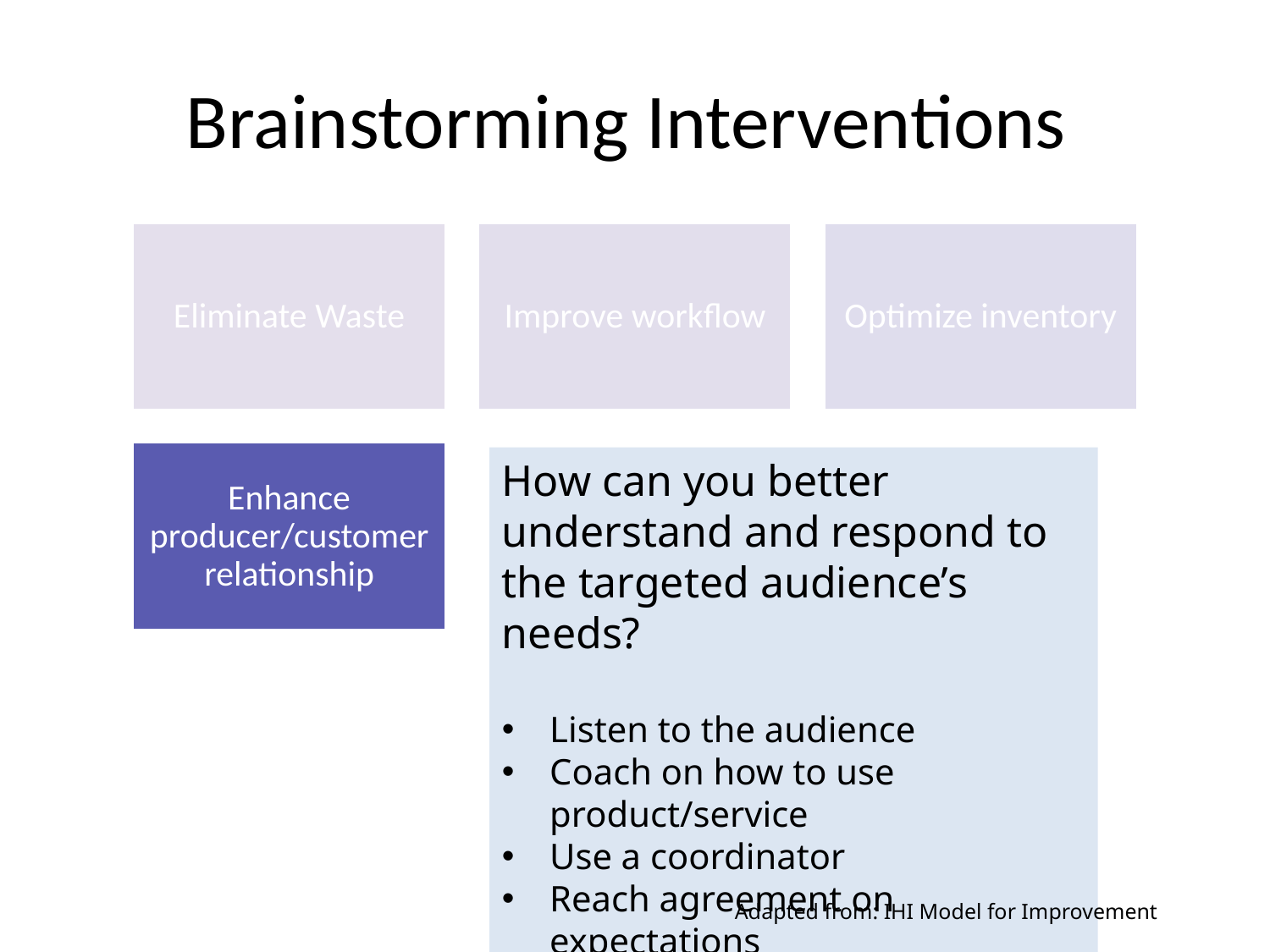

# Brainstorming Interventions
How can you better understand and respond to the targeted audience’s needs?
Listen to the audience
Coach on how to use product/service
Use a coordinator
Reach agreement on expectations
Adapted from: IHI Model for Improvement

## Slide 37
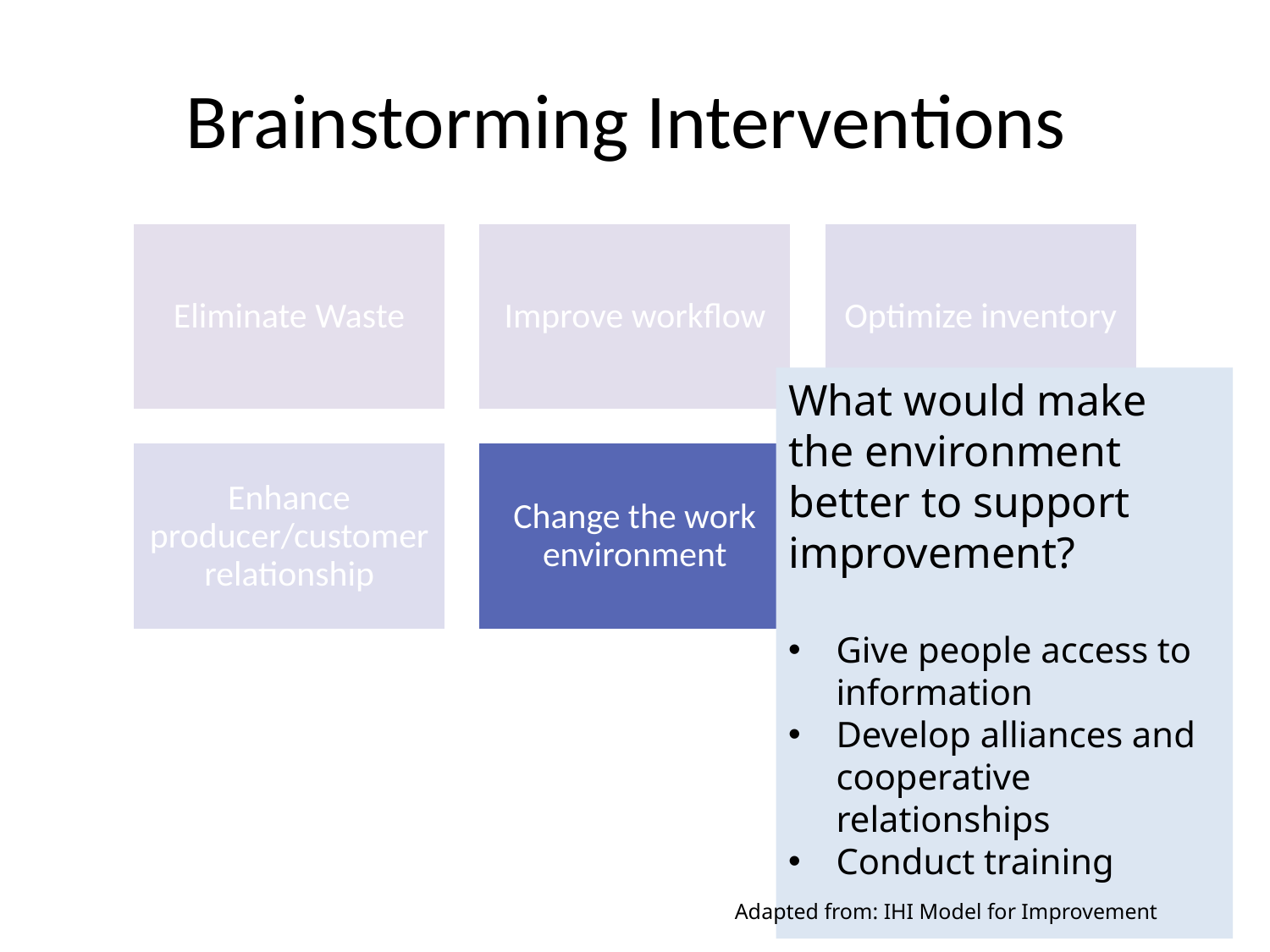

# Brainstorming Interventions
What would make the environment better to support improvement?
Give people access to information
Develop alliances and cooperative relationships
Conduct training
Adapted from: IHI Model for Improvement

## Slide 38
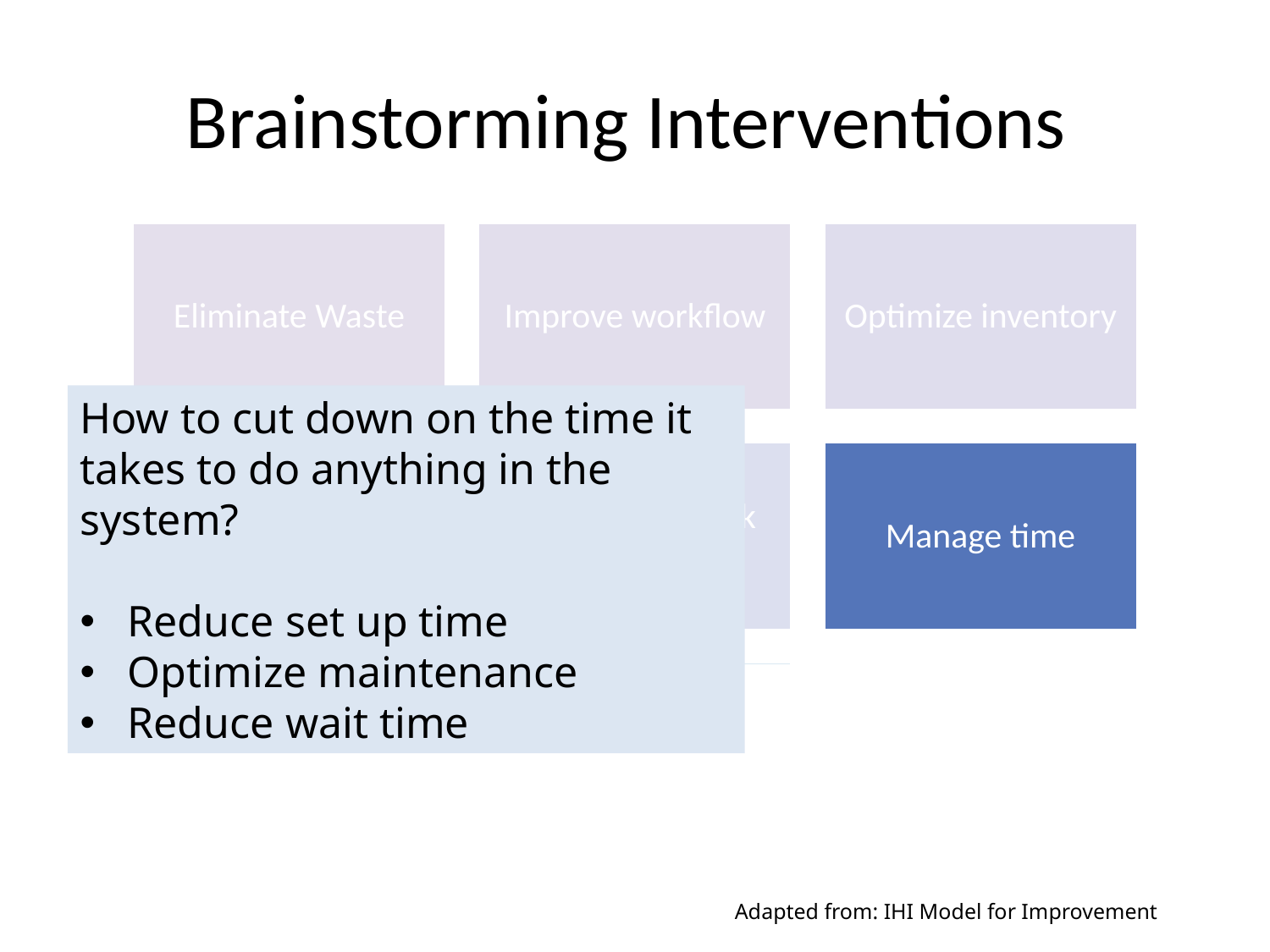

# Brainstorming Interventions
How to cut down on the time it takes to do anything in the system?
Reduce set up time
Optimize maintenance
Reduce wait time
Adapted from: IHI Model for Improvement

## Slide 39
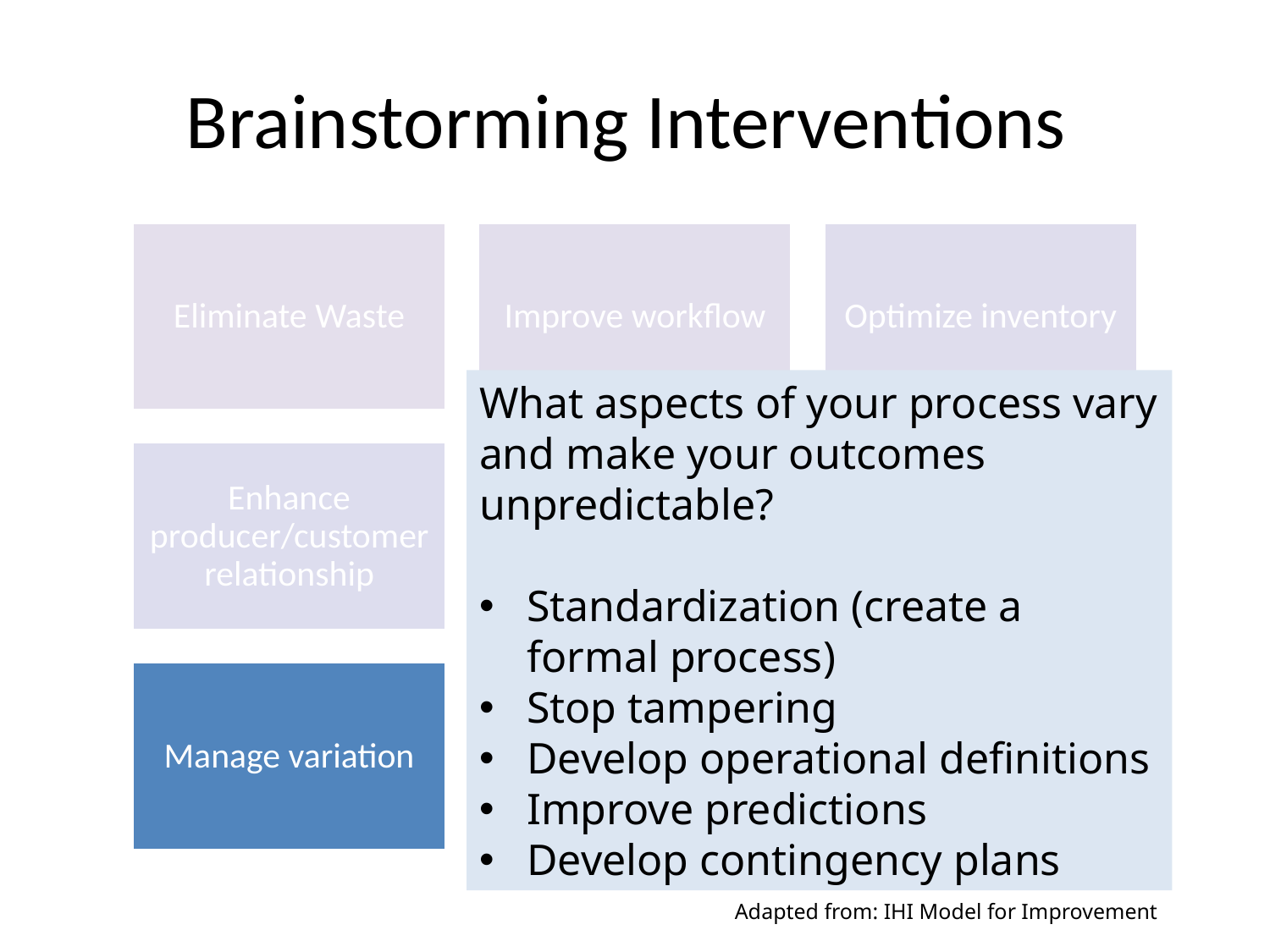

# Brainstorming Interventions
What aspects of your process vary and make your outcomes unpredictable?
Standardization (create a formal process)
Stop tampering
Develop operational definitions
Improve predictions
Develop contingency plans
Adapted from: IHI Model for Improvement

## Slide 40
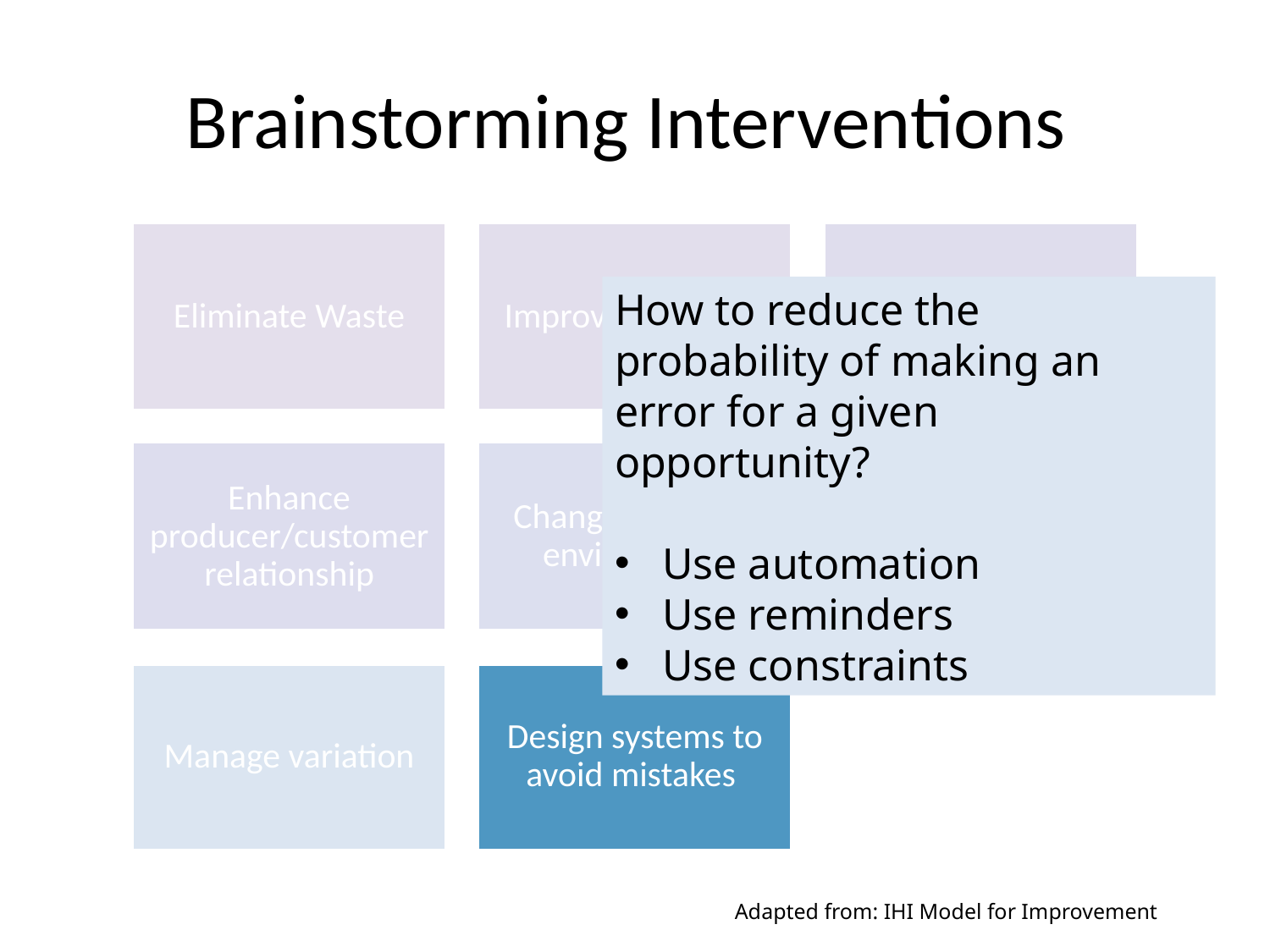

# Brainstorming Interventions
How to reduce the probability of making an error for a given opportunity?
Use automation
Use reminders
Use constraints
Adapted from: IHI Model for Improvement

## Slide 41
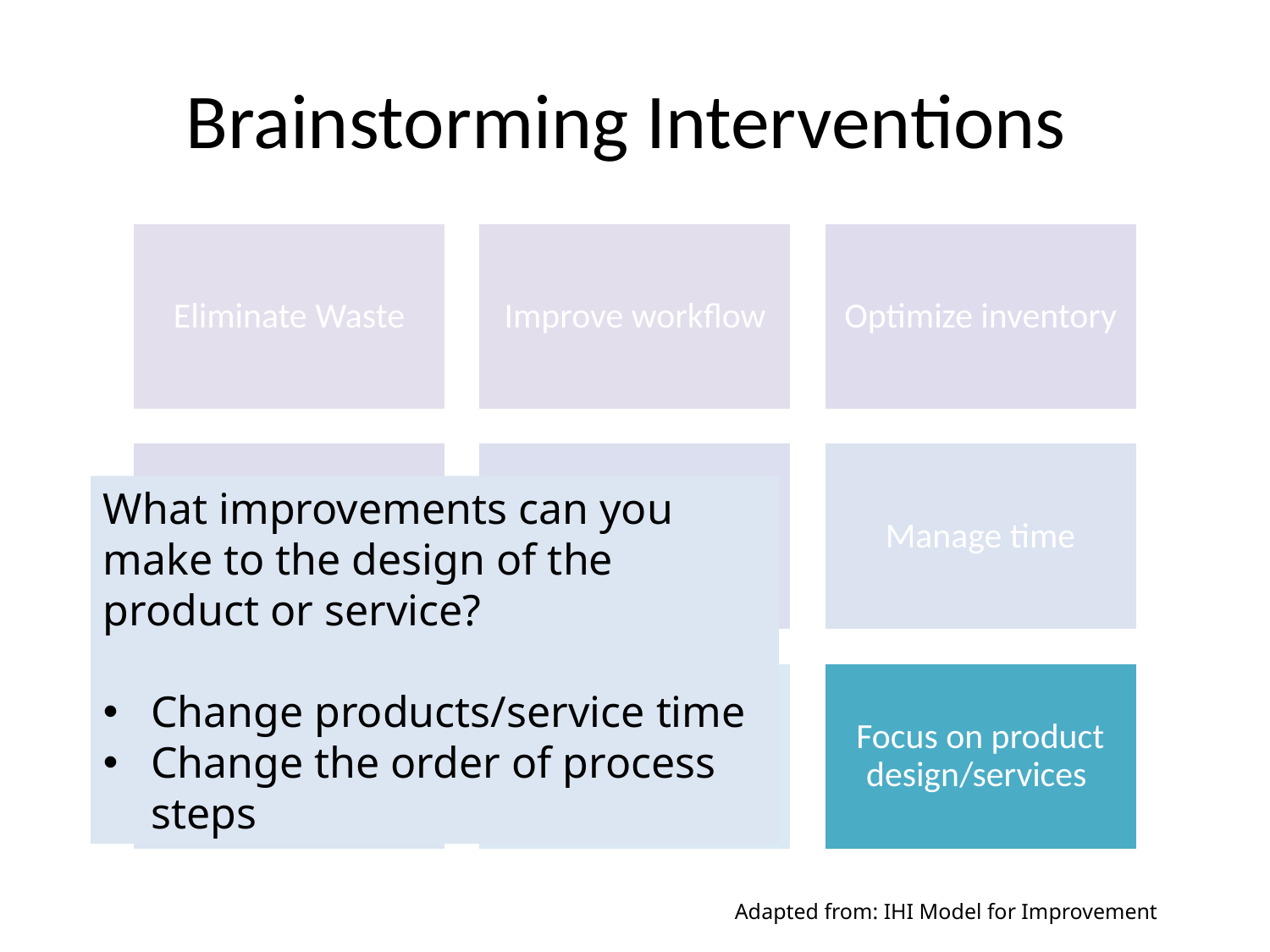

# Brainstorming Interventions
What improvements can you make to the design of the product or service?
Change products/service time
Change the order of process steps
Adapted from: IHI Model for Improvement

## Slide 42
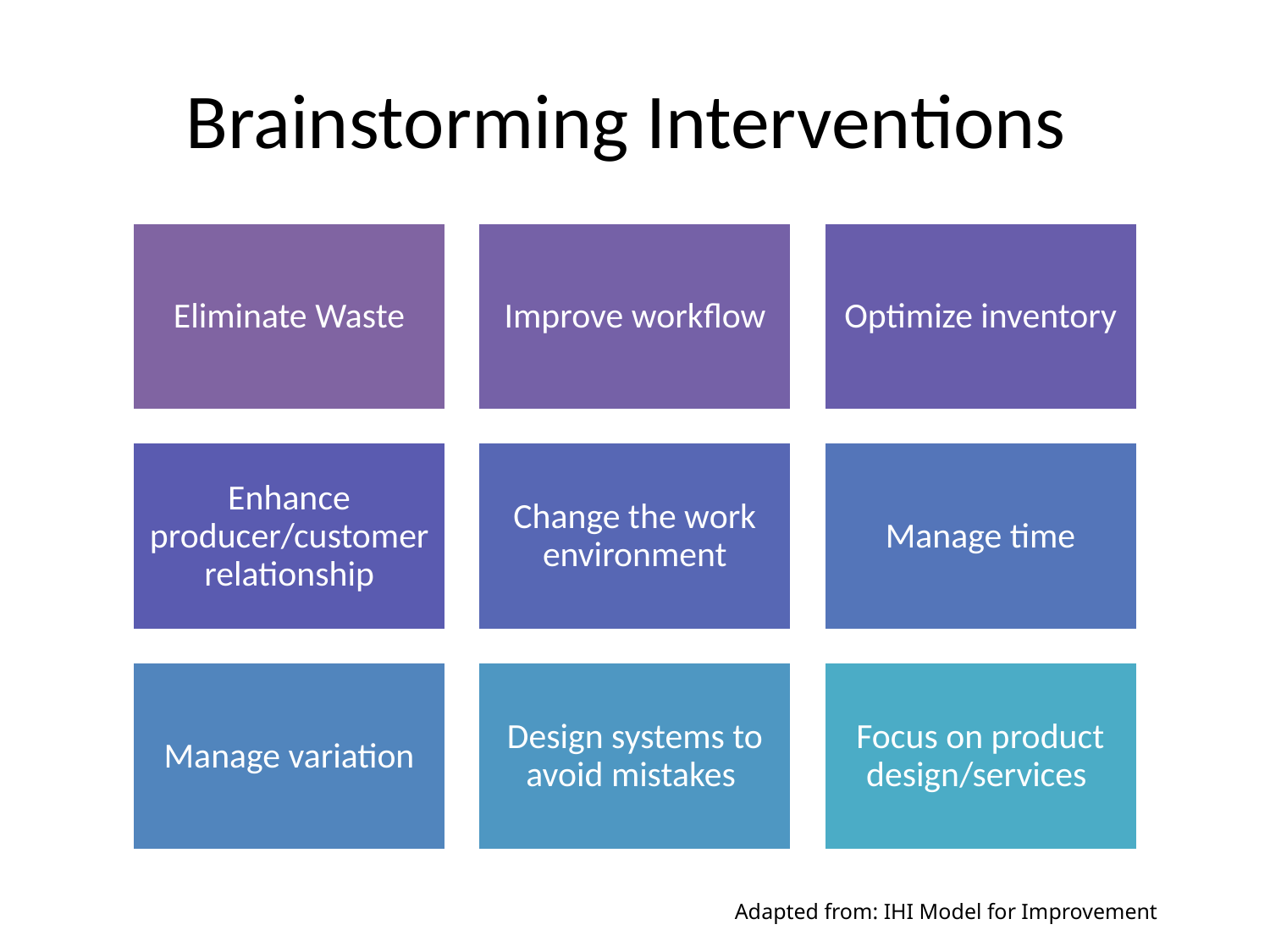

# Brainstorming Interventions
Adapted from: IHI Model for Improvement

## Slide 43
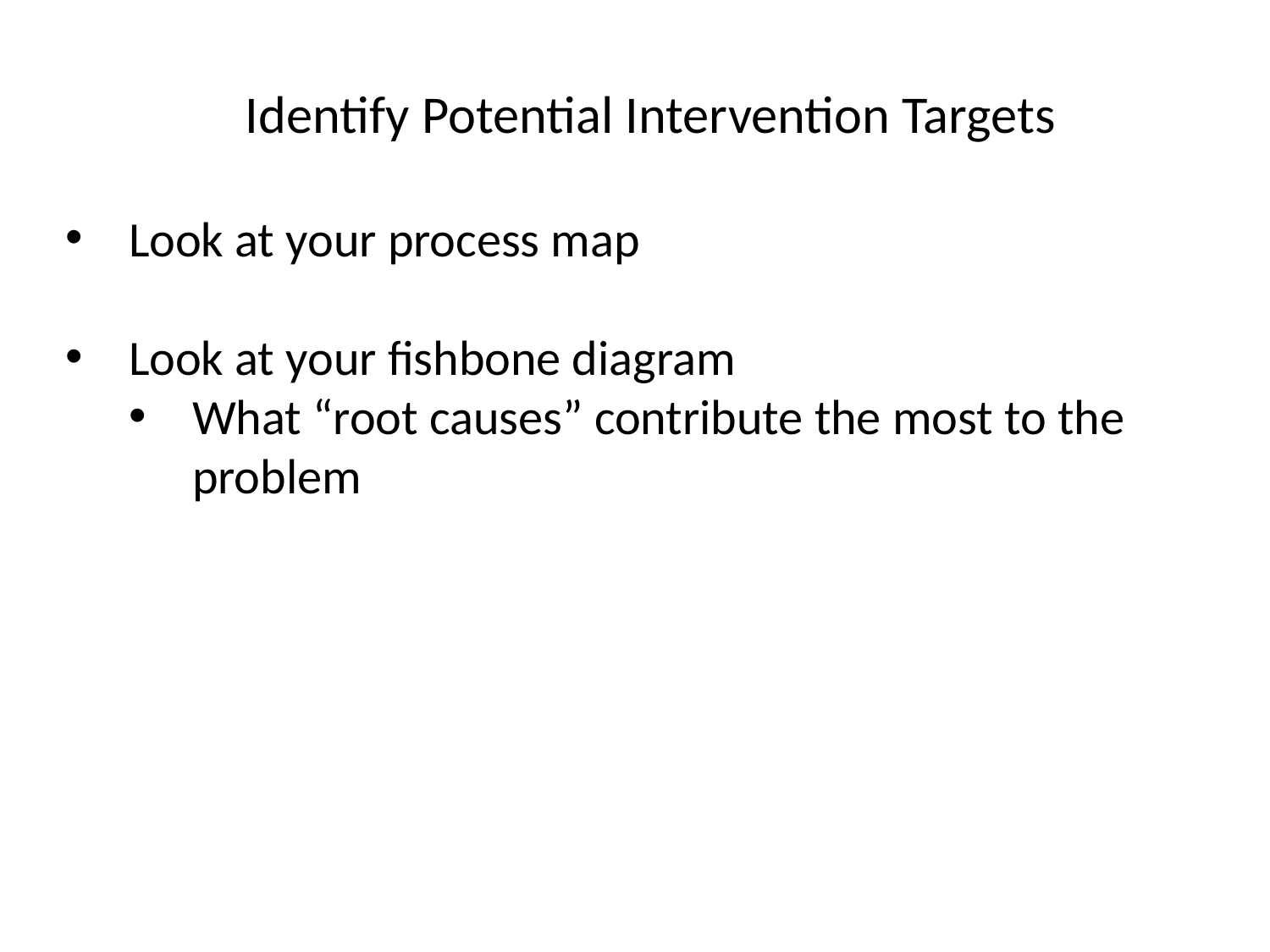

# Identify Potential Intervention Targets
Look at your process map
Look at your fishbone diagram
What “root causes” contribute the most to the problem

## Slide 44
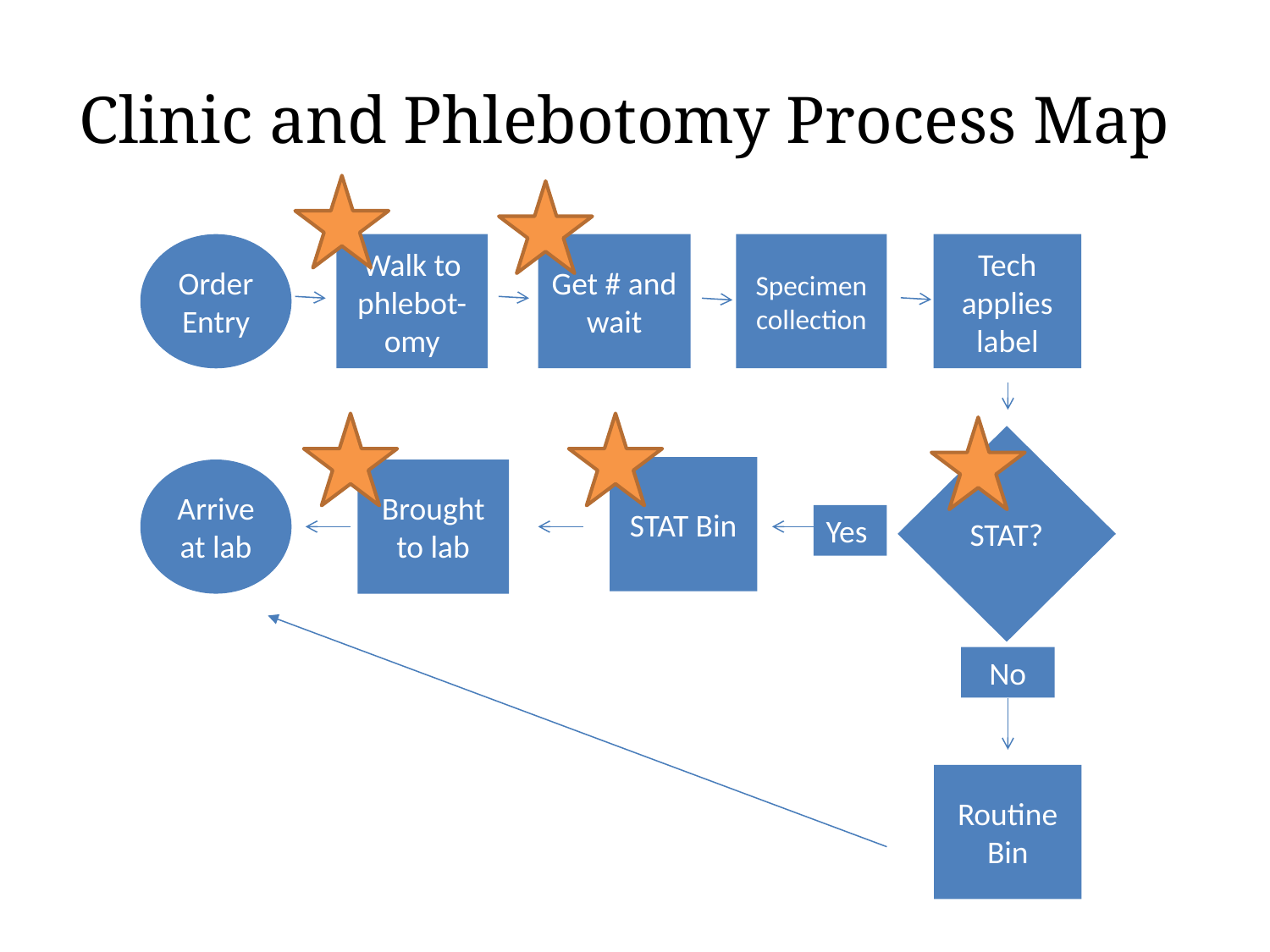

# Clinic and Phlebotomy Process Map
Order Entry
Walk to phlebot-omy
Get # and wait
Specimen collection
Tech applies label
STAT?
STAT Bin
Arrive at lab
Brought to lab
Yes
No
Routine Bin

## Slide 45
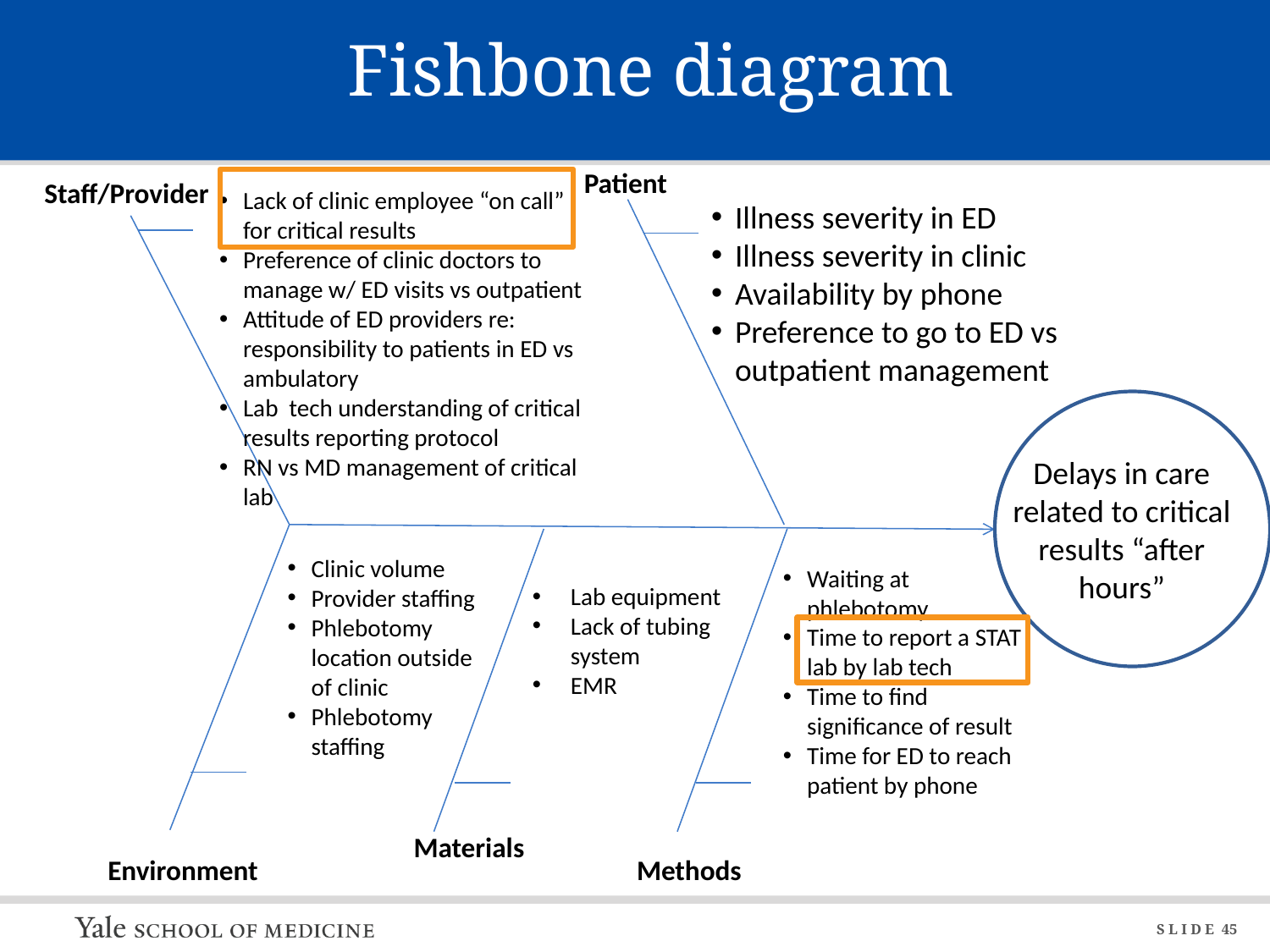

# Fishbone diagram
Patient
Staff/Provider
Lack of clinic employee “on call” for critical results
Preference of clinic doctors to manage w/ ED visits vs outpatient
Attitude of ED providers re: responsibility to patients in ED vs ambulatory
Lab tech understanding of critical results reporting protocol
RN vs MD management of critical lab
Illness severity in ED
Illness severity in clinic
Availability by phone
Preference to go to ED vs outpatient management
Delays in care related to critical results “after hours”
Clinic volume
Provider staffing
Phlebotomy location outside of clinic
Phlebotomy staffing
Waiting at phlebotomy
Time to report a STAT lab by lab tech
Time to find significance of result
Time for ED to reach patient by phone
Lab equipment
Lack of tubing system
EMR
Materials
Environment
Methods

## Slide 46
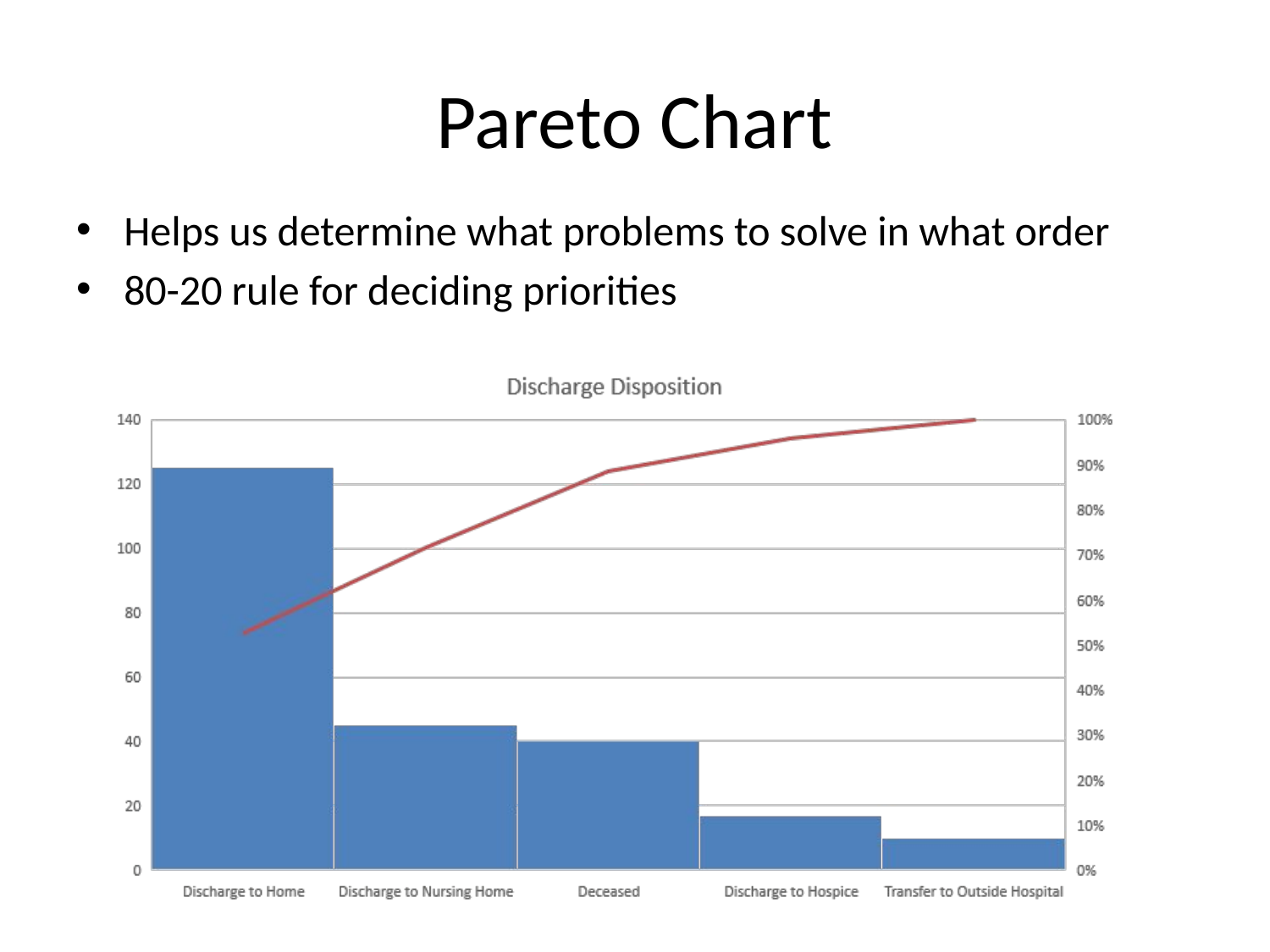

# Pareto Chart
Helps us determine what problems to solve in what order
80-20 rule for deciding priorities

## Slide 47
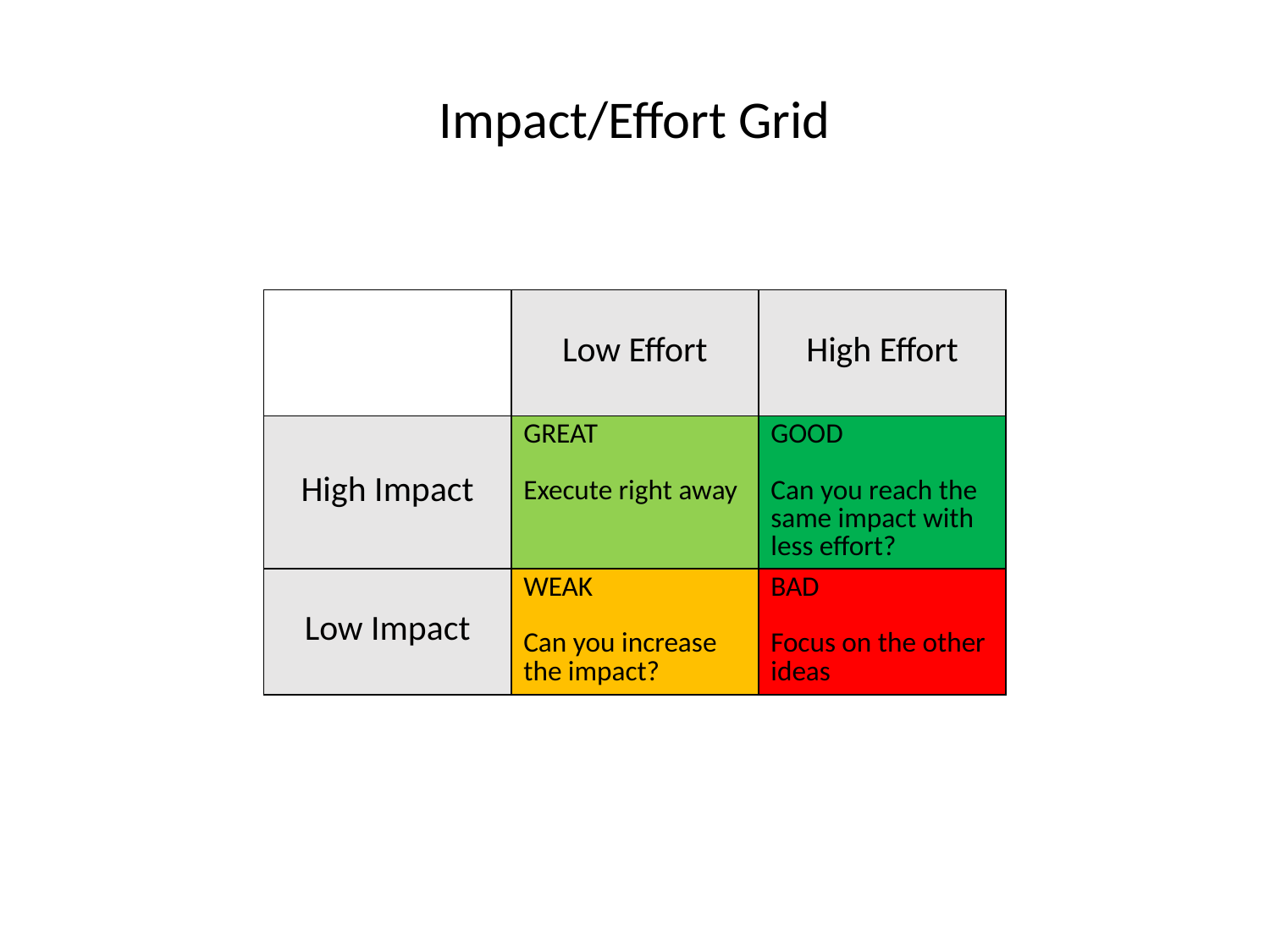

# Impact/Effort Grid
| ​ | Low Effort​ | High Effort​ |
| --- | --- | --- |
| High Impact​ | ​GREAT Execute right away | ​GOOD Can you reach the same impact with less effort? |
| Low Impact​ | ​WEAK Can you increase the impact? | ​BAD Focus on the other ideas |

## Slide 48
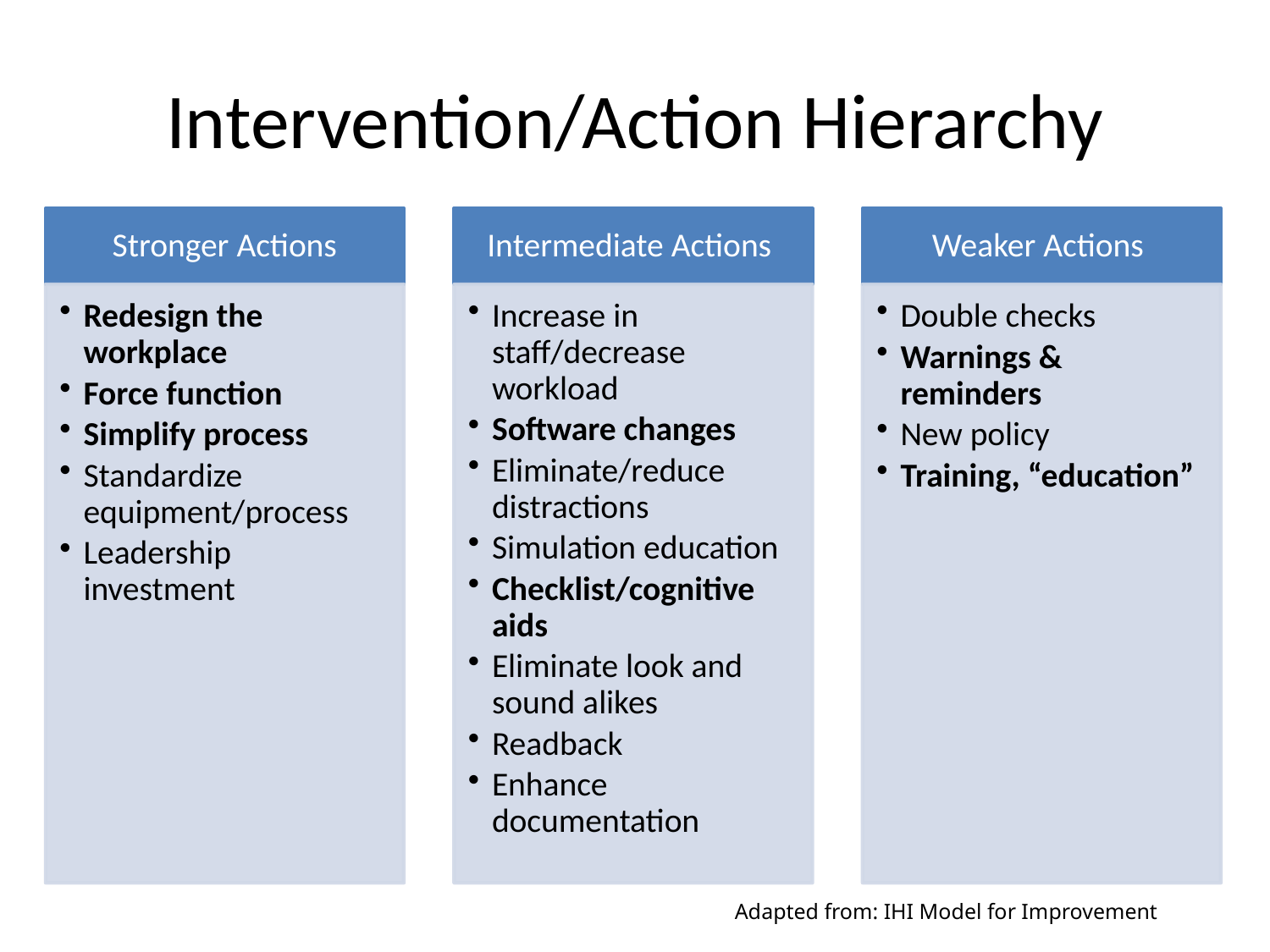

# Intervention/Action Hierarchy
Adapted from: IHI Model for Improvement

## Slide 49
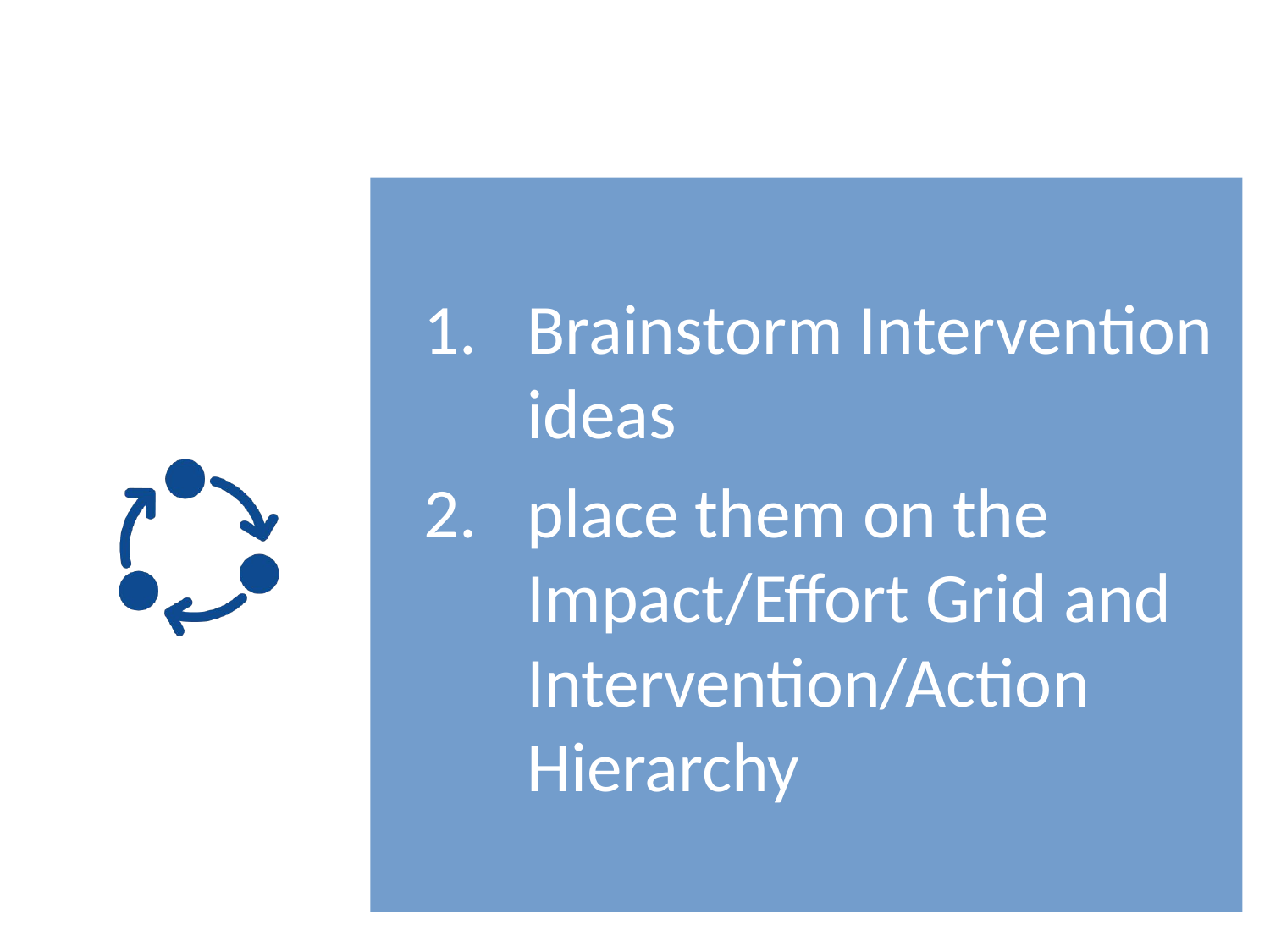

#
Brainstorm Intervention ideas
place them on the Impact/Effort Grid and Intervention/Action Hierarchy

## Slide 50
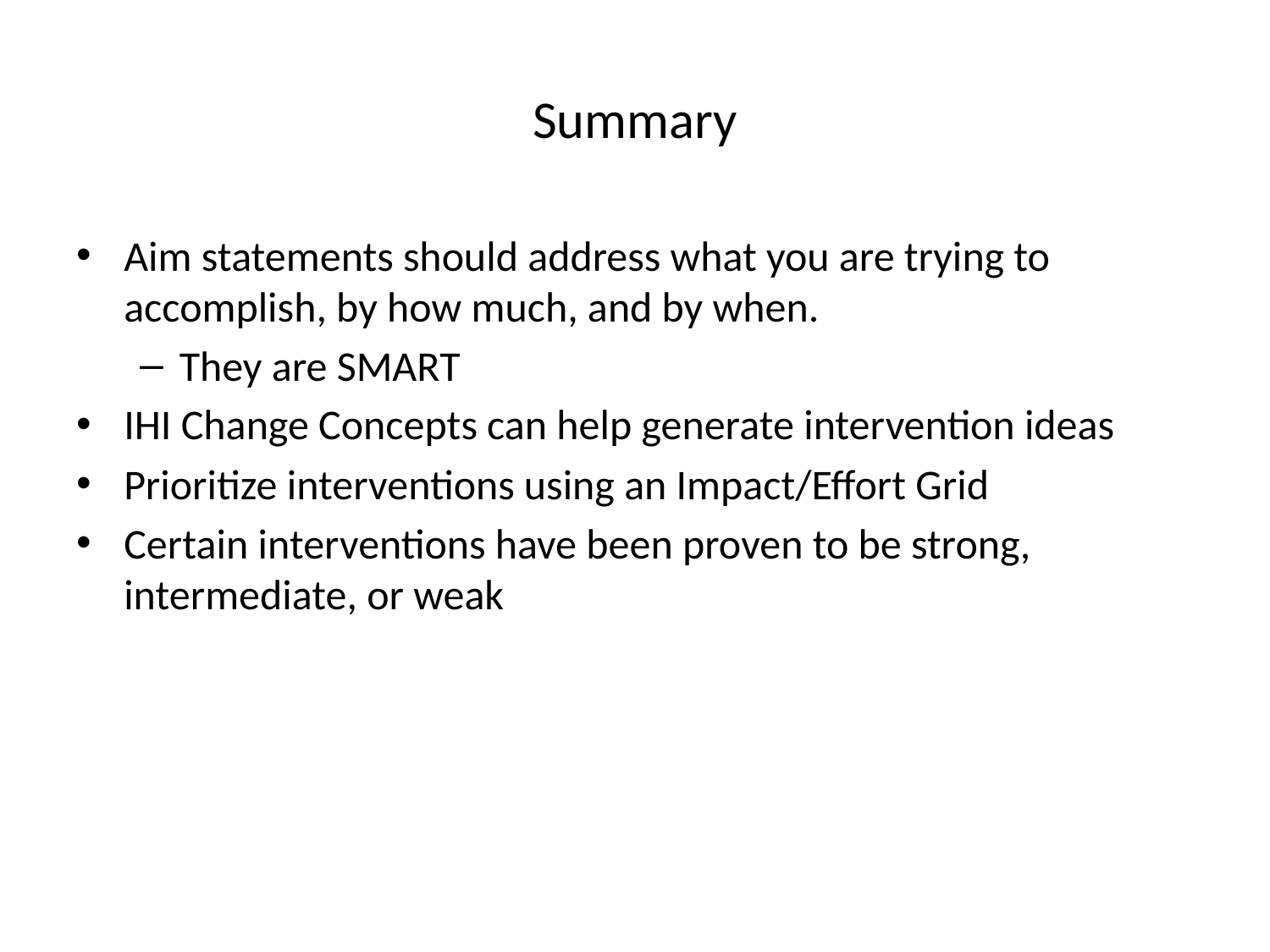

# Summary
Aim statements should address what you are trying to accomplish, by how much, and by when.
They are SMART
IHI Change Concepts can help generate intervention ideas
Prioritize interventions using an Impact/Effort Grid
Certain interventions have been proven to be strong, intermediate, or weak

## Slide 51
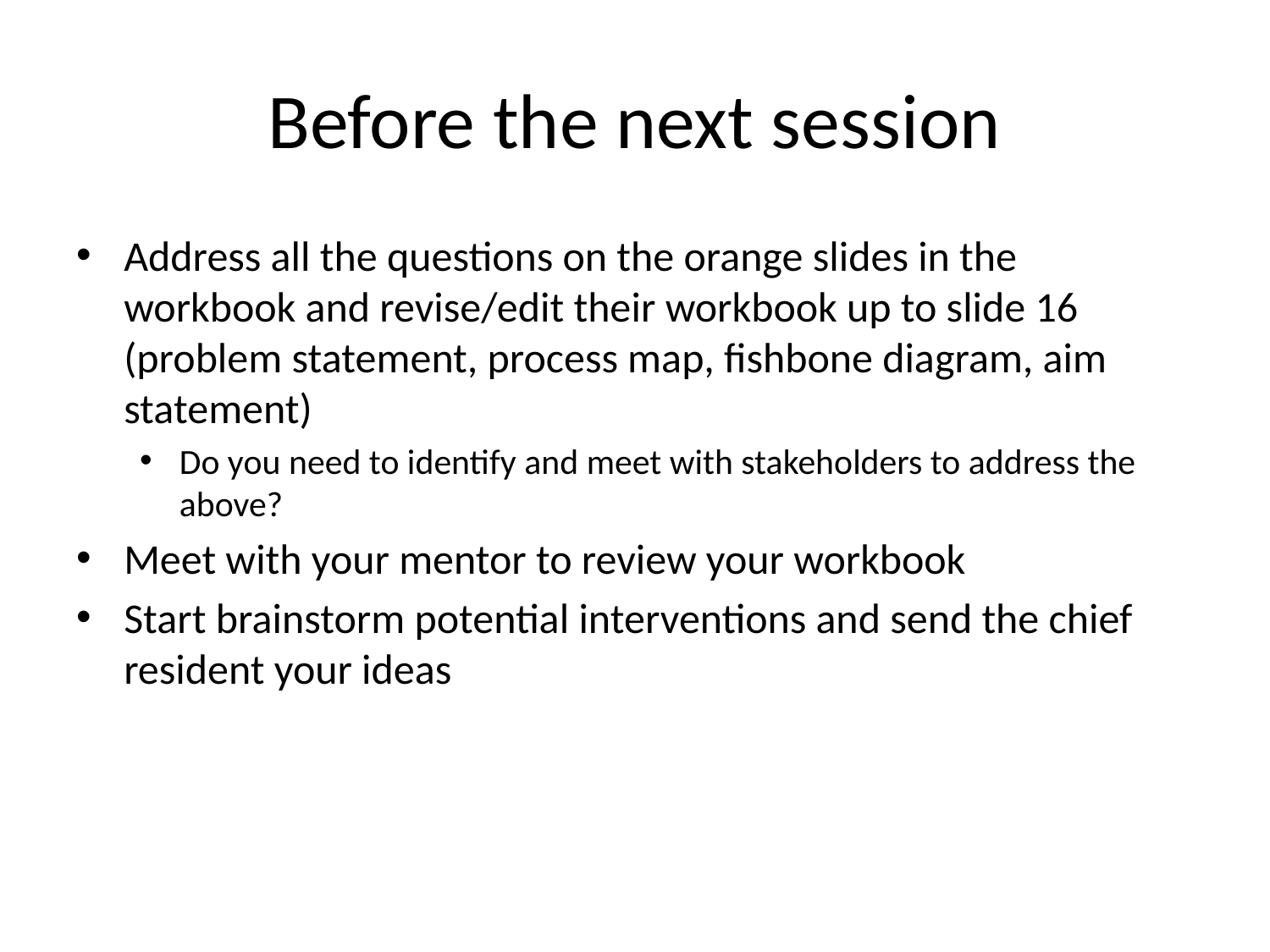

# Before the next session
Address all the questions on the orange slides in the workbook and revise/edit their workbook up to slide 16 (problem statement, process map, fishbone diagram, aim statement)
Do you need to identify and meet with stakeholders to address the above?
Meet with your mentor to review your workbook
Start brainstorm potential interventions and send the chief resident your ideas
