# Supplementary material for: Building Resident Quality Improvement Knowledge and Engagement Through a Longitudinal, Mentored, and Experiential Learning-Based Quality Improvement Curriculum
Source: MedEdPORTAL. 2023 Apr 18;19:11310. doi: 10.15766/mep_2374-8265.11310 (PMC10110773; doi:10.15766/mep_2374-8265.11310)
Supplement: Supplementary file 1 — Session 1 Slides.pptxSession 1 Workbook.pptxSession 2 Slides.pptxSession 2 Workbook.pptxSession 3 Slides.pptxSession 4 Work-in-Progress Presentation Template.pptxSession 5 Slides.pptxQI Charter Template.docxFaculty Milestones.docxFaculty Guide.docxResident Survey.docx [file mep_2374-8265.11310-s001.zip › D. Session 2 Workbook.pptx]

## Slide 1
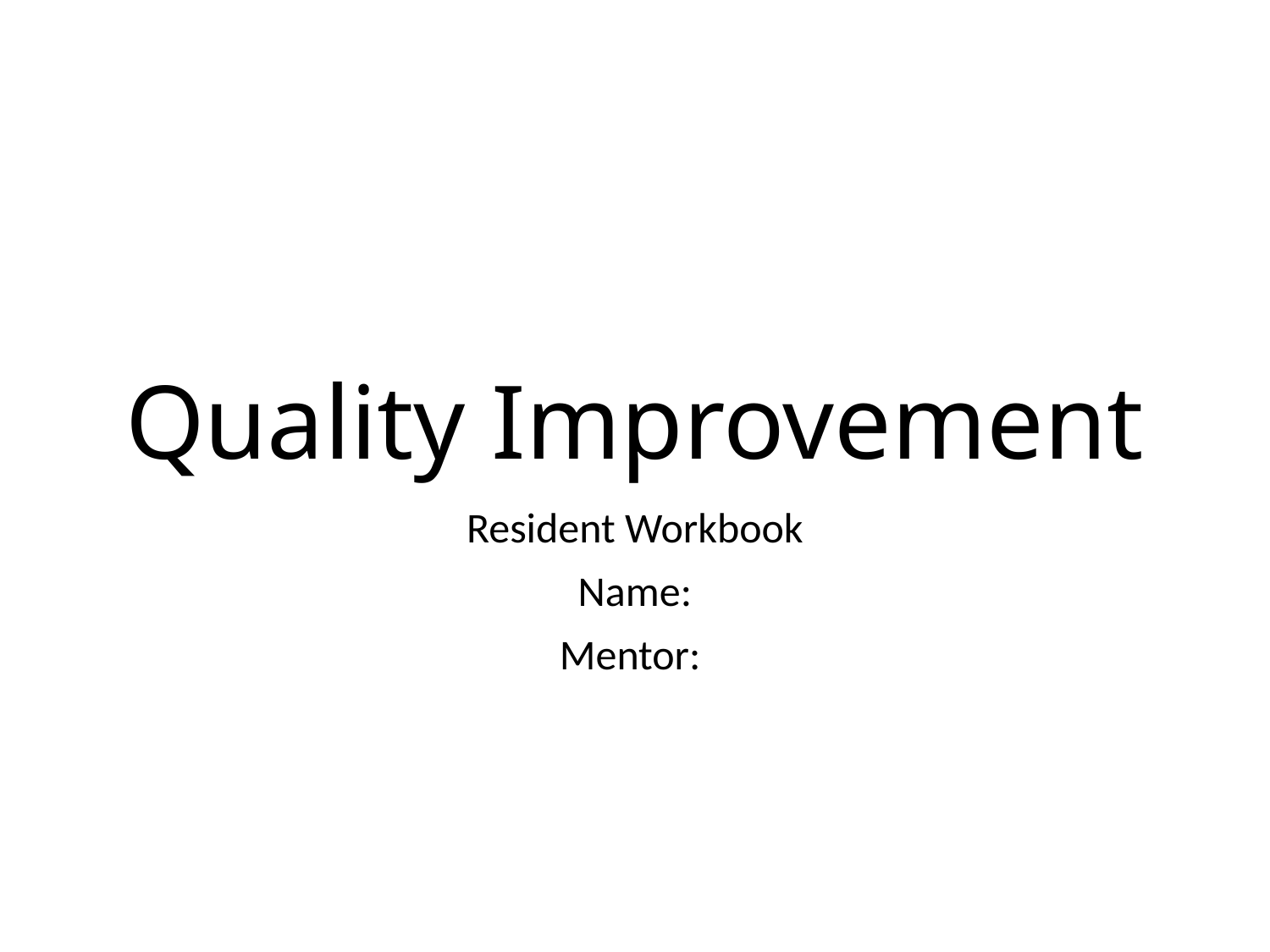

# Quality Improvement
Resident Workbook
Name:
Mentor:

## Slide 2
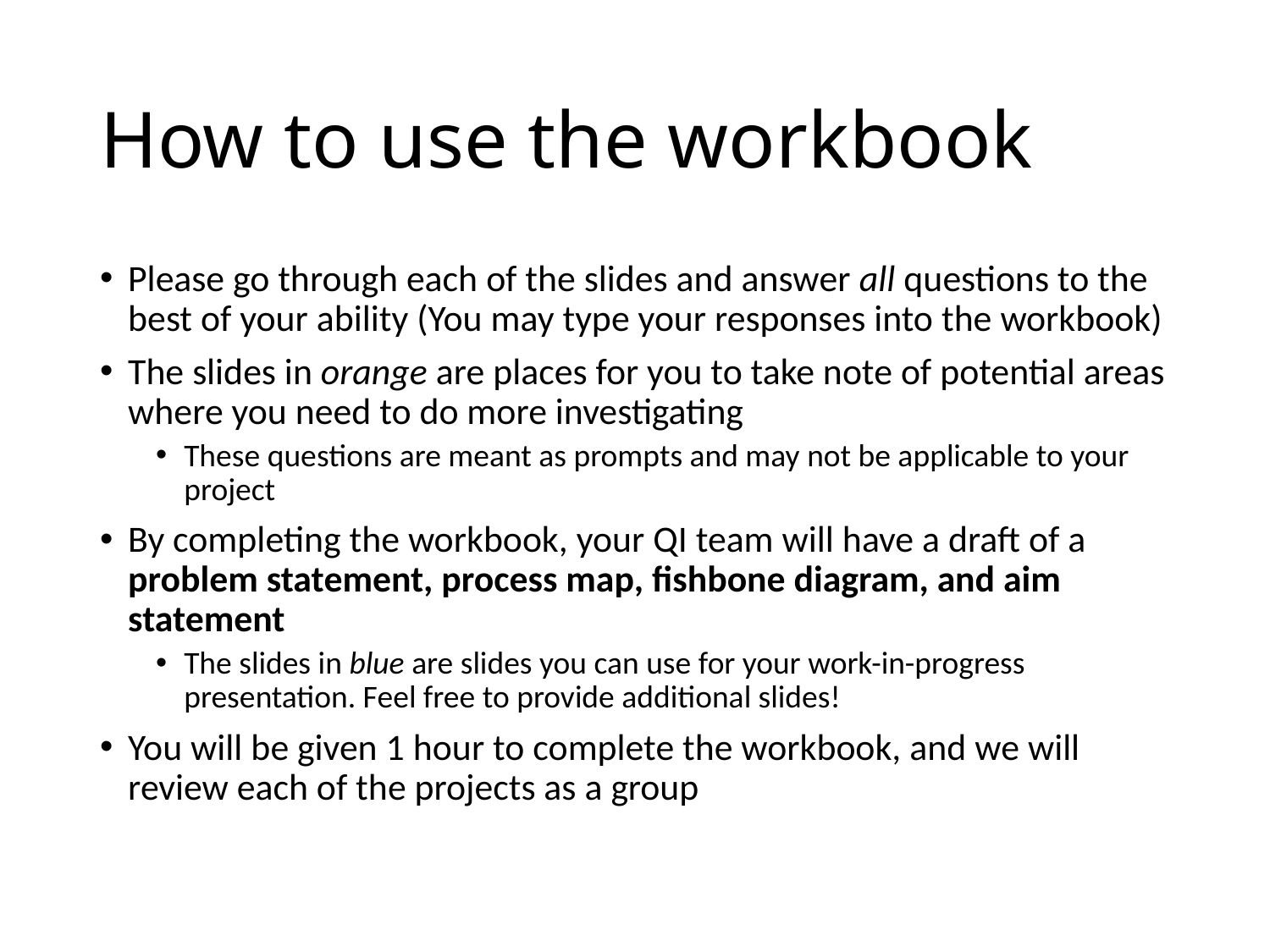

# How to use the workbook
Please go through each of the slides and answer all questions to the best of your ability (You may type your responses into the workbook)
The slides in orange are places for you to take note of potential areas where you need to do more investigating
These questions are meant as prompts and may not be applicable to your project
By completing the workbook, your QI team will have a draft of a problem statement, process map, fishbone diagram, and aim statement
The slides in blue are slides you can use for your work-in-progress presentation. Feel free to provide additional slides!
You will be given 1 hour to complete the workbook, and we will review each of the projects as a group

## Slide 3
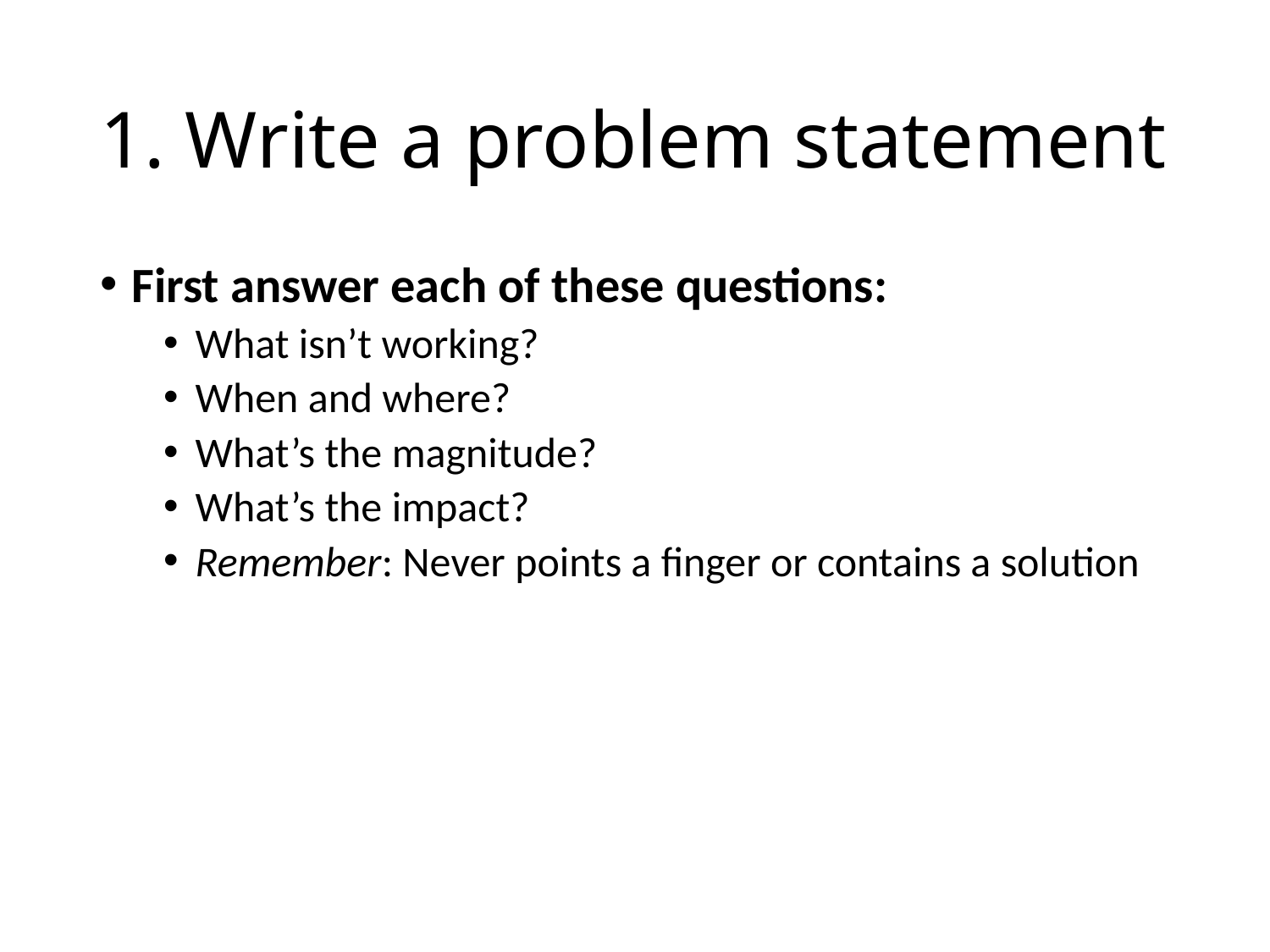

# 1. Write a problem statement
First answer each of these questions:
What isn’t working?
When and where?
What’s the magnitude?
What’s the impact?
Remember: Never points a finger or contains a solution

## Slide 4
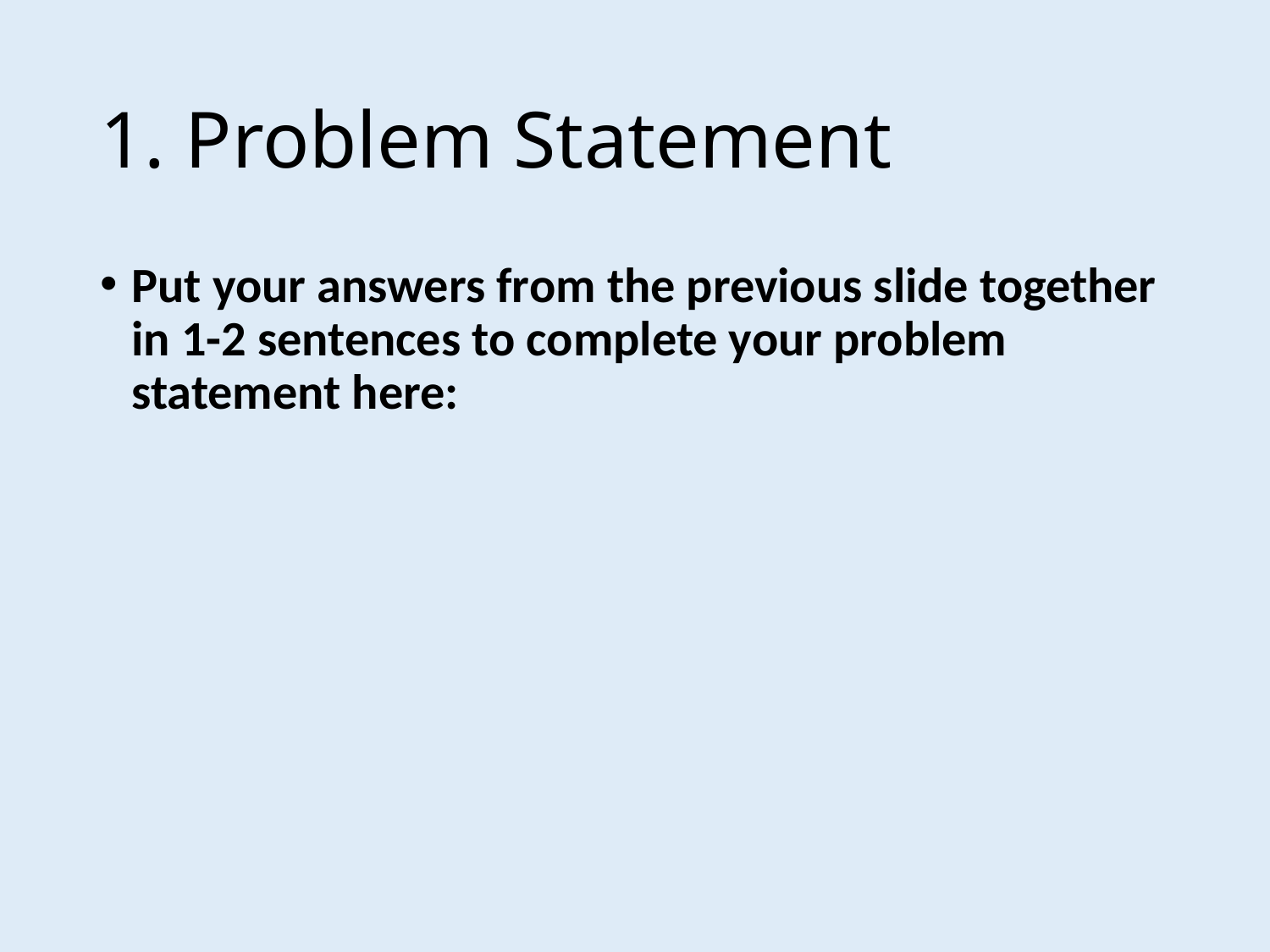

# 1. Problem Statement
Put your answers from the previous slide together in 1-2 sentences to complete your problem statement here:

## Slide 5
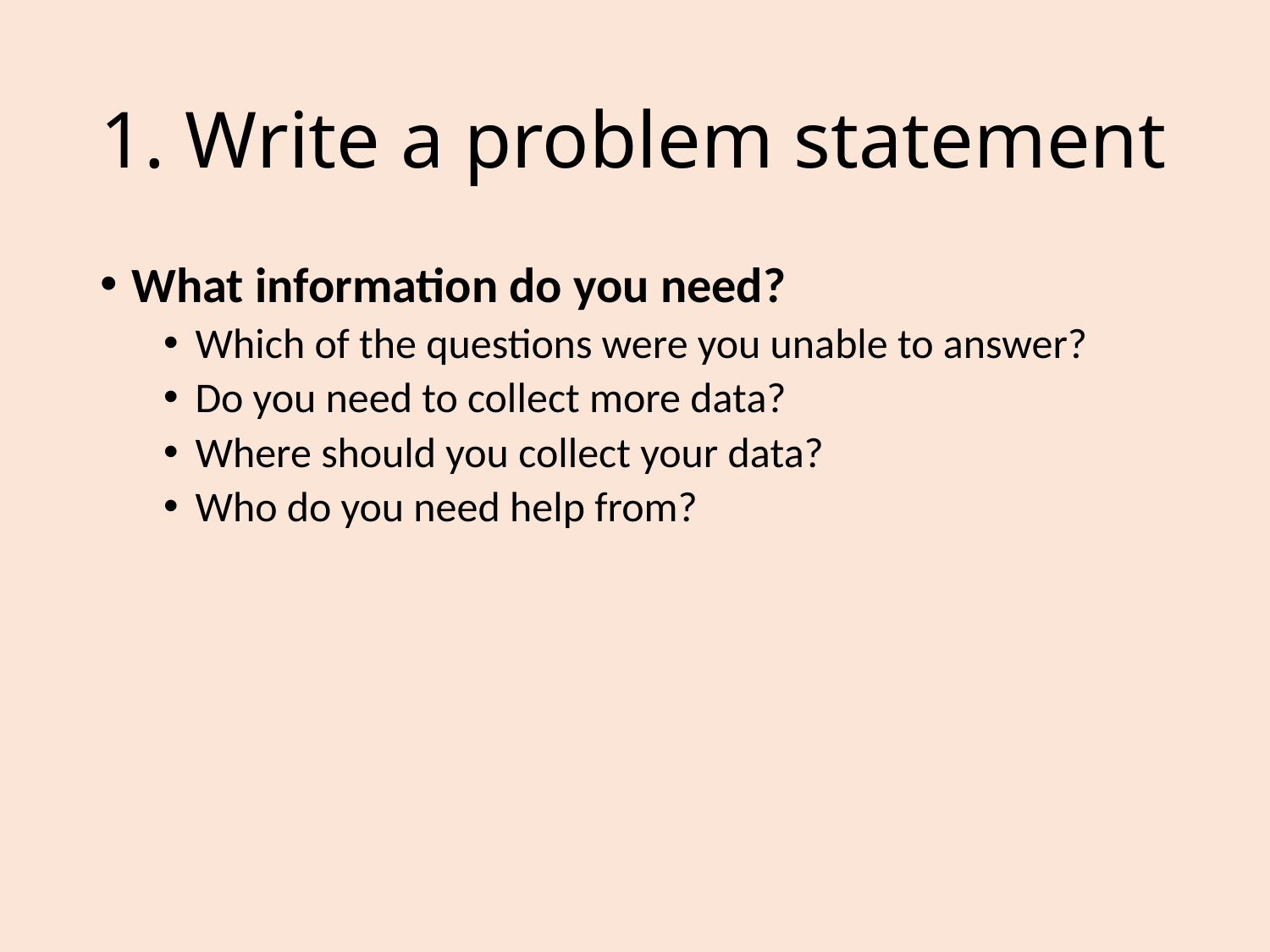

# 1. Write a problem statement
What information do you need?
Which of the questions were you unable to answer?
Do you need to collect more data?
Where should you collect your data?
Who do you need help from?

## Slide 6
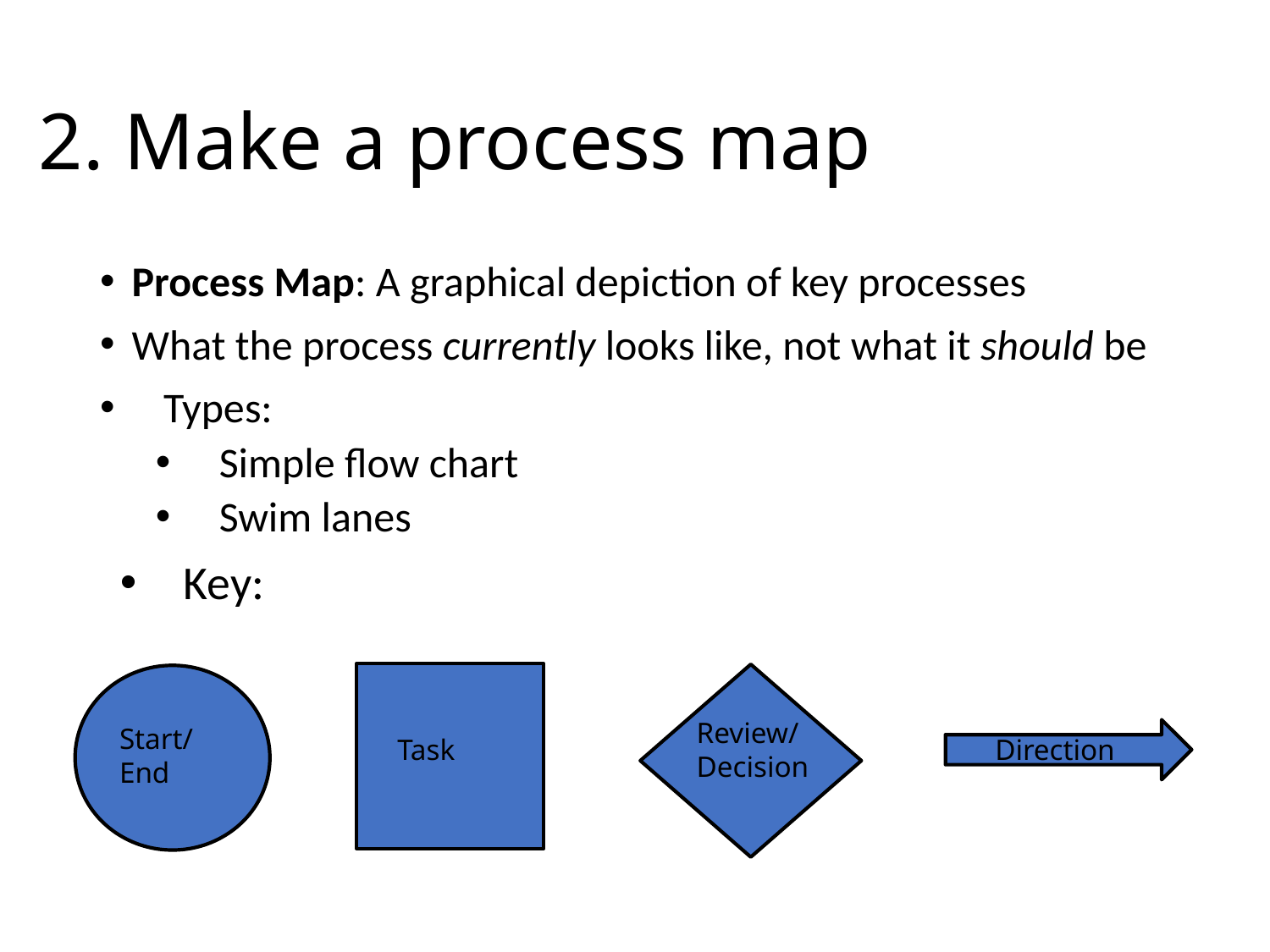

# 2. Make a process map
Process Map: A graphical depiction of key processes
What the process currently looks like, not what it should be
Types:
Simple flow chart
Swim lanes
Key:
Review/
Decision
Start/
End
Task
Direction

## Slide 7
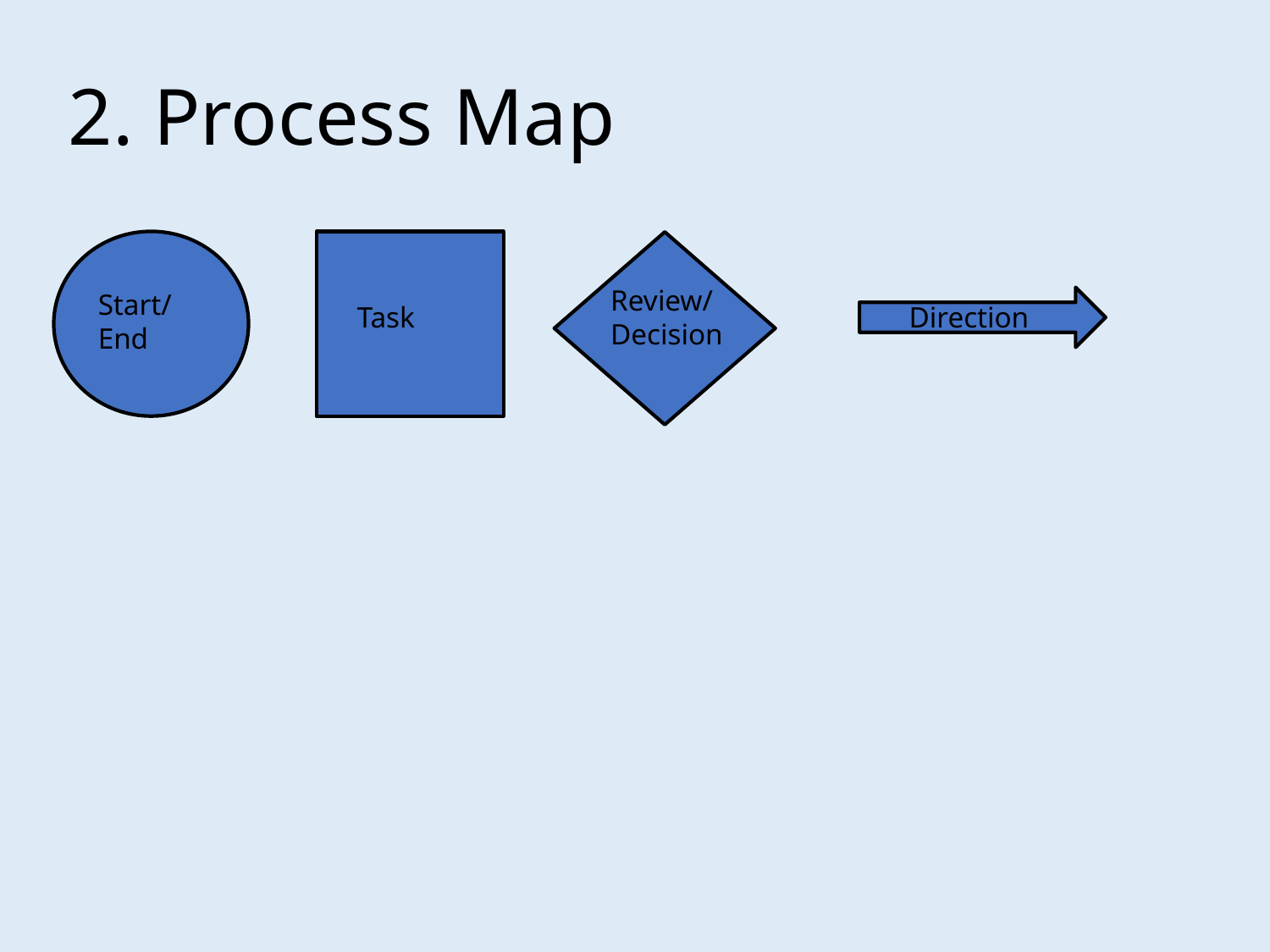

# 2. Process Map
Review/
Decision
Start/
End
Task
Direction

## Slide 8
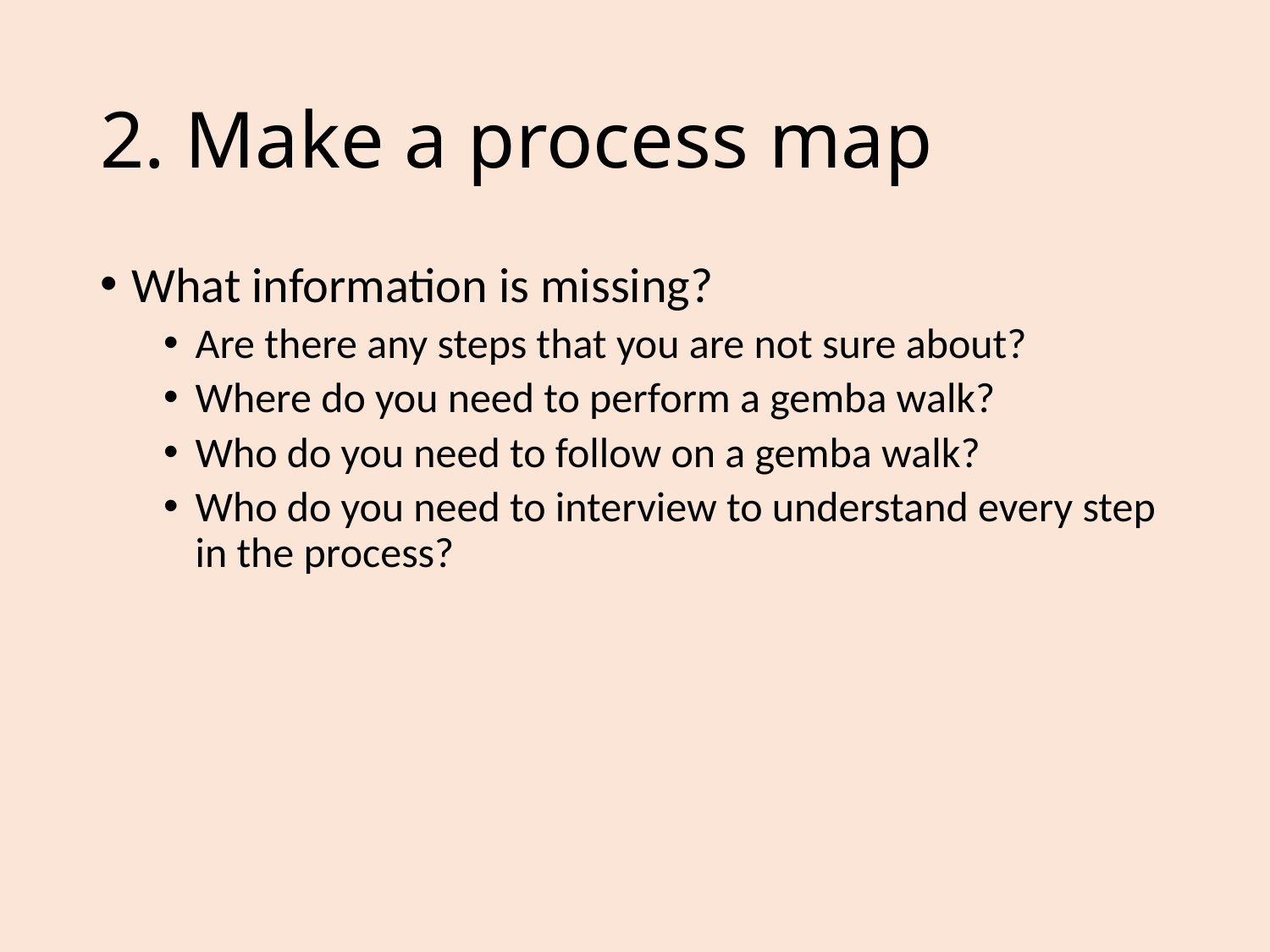

# 2. Make a process map
What information is missing?
Are there any steps that you are not sure about?
Where do you need to perform a gemba walk?
Who do you need to follow on a gemba walk?
Who do you need to interview to understand every step in the process?

## Slide 9
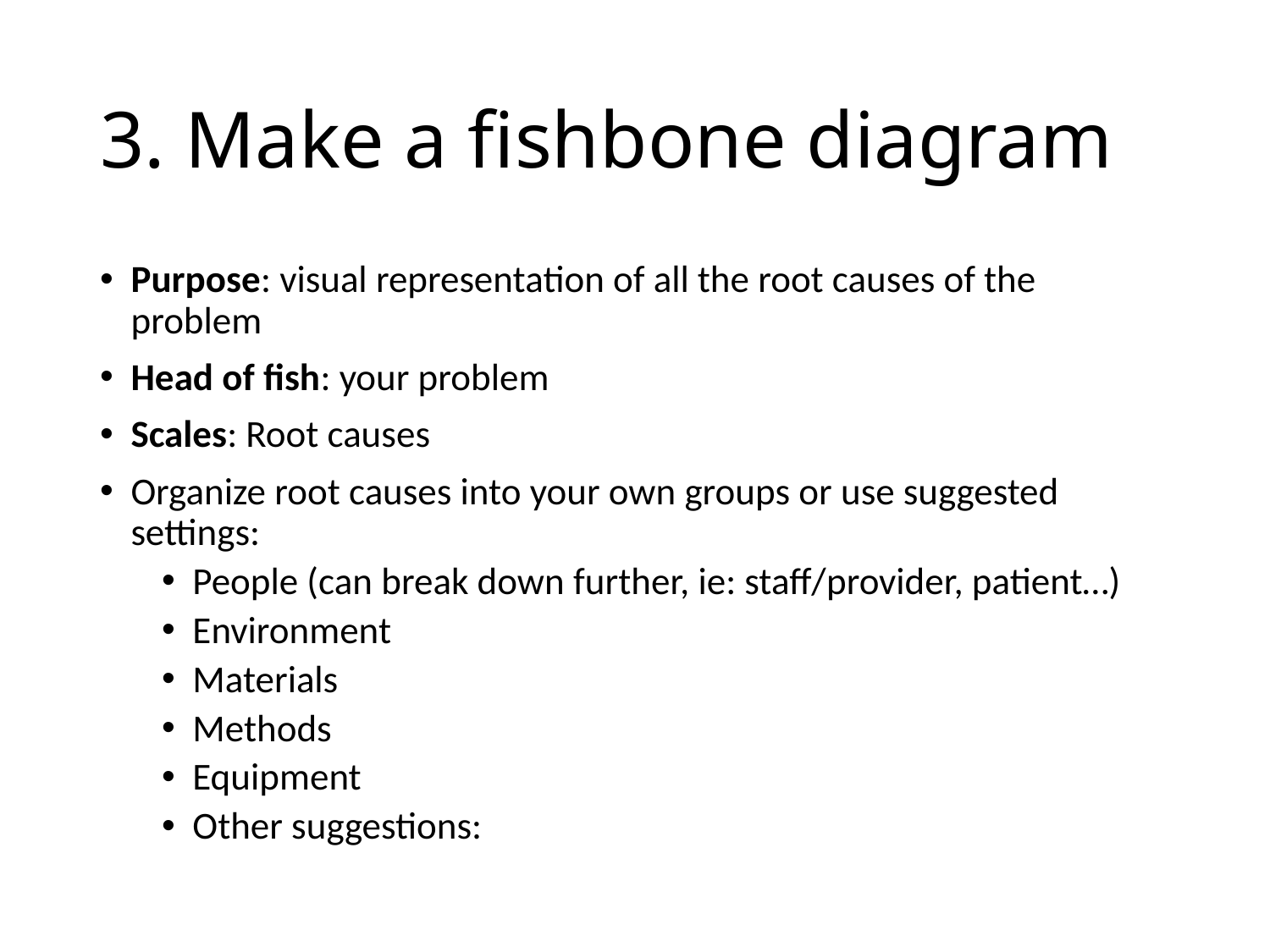

# 3. Make a fishbone diagram
Purpose: visual representation of all the root causes of the problem
Head of fish: your problem
Scales: Root causes
Organize root causes into your own groups or use suggested settings:
People (can break down further, ie: staff/provider, patient…)
Environment
Materials
Methods
Equipment
Other suggestions:

## Slide 10
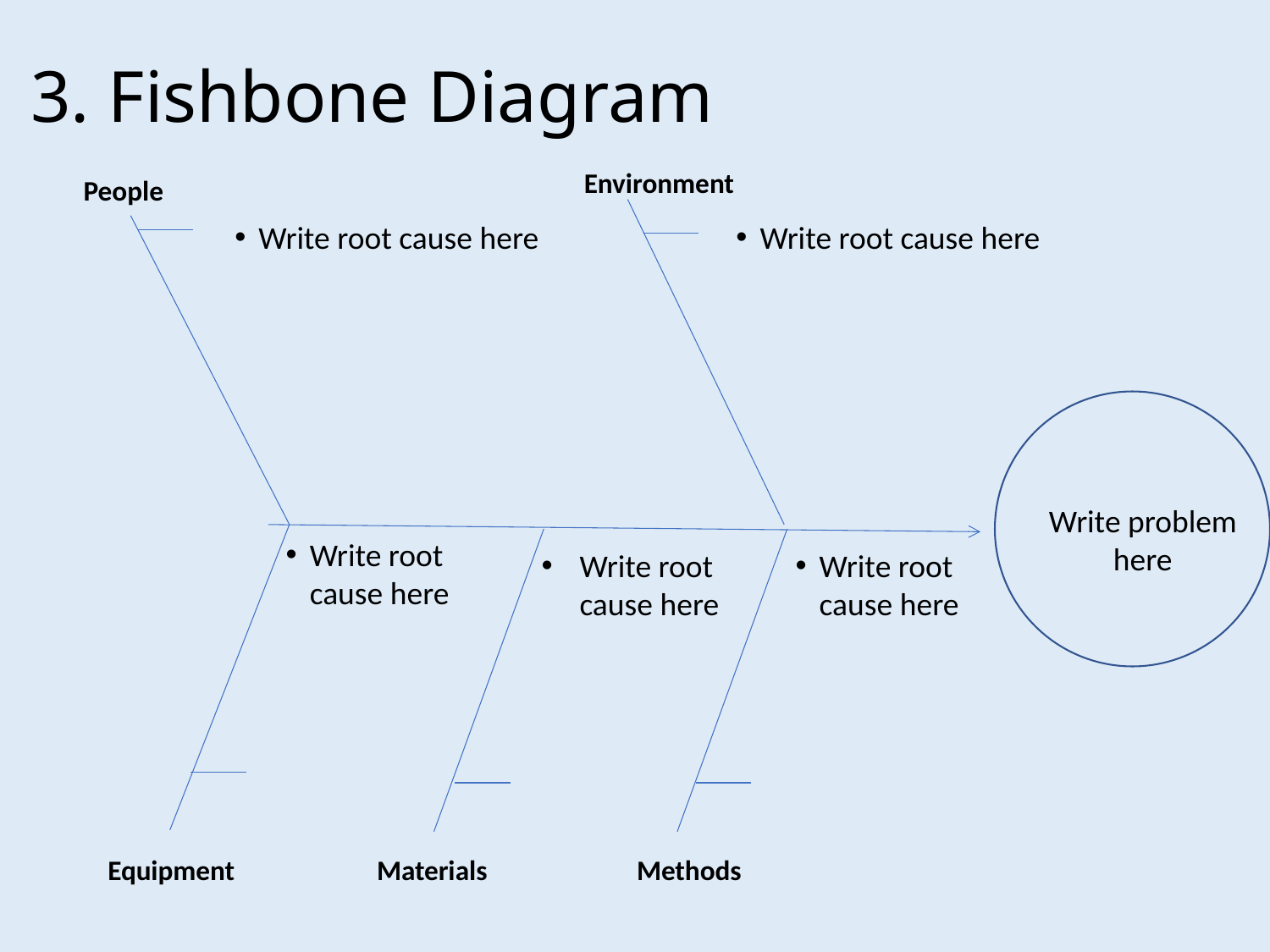

# 3. Fishbone Diagram
Environment
People
Write root cause here
Write root cause here
Write problem here
Write root cause here
Write root cause here
Write root cause here
Materials
Methods
Equipment

## Slide 11
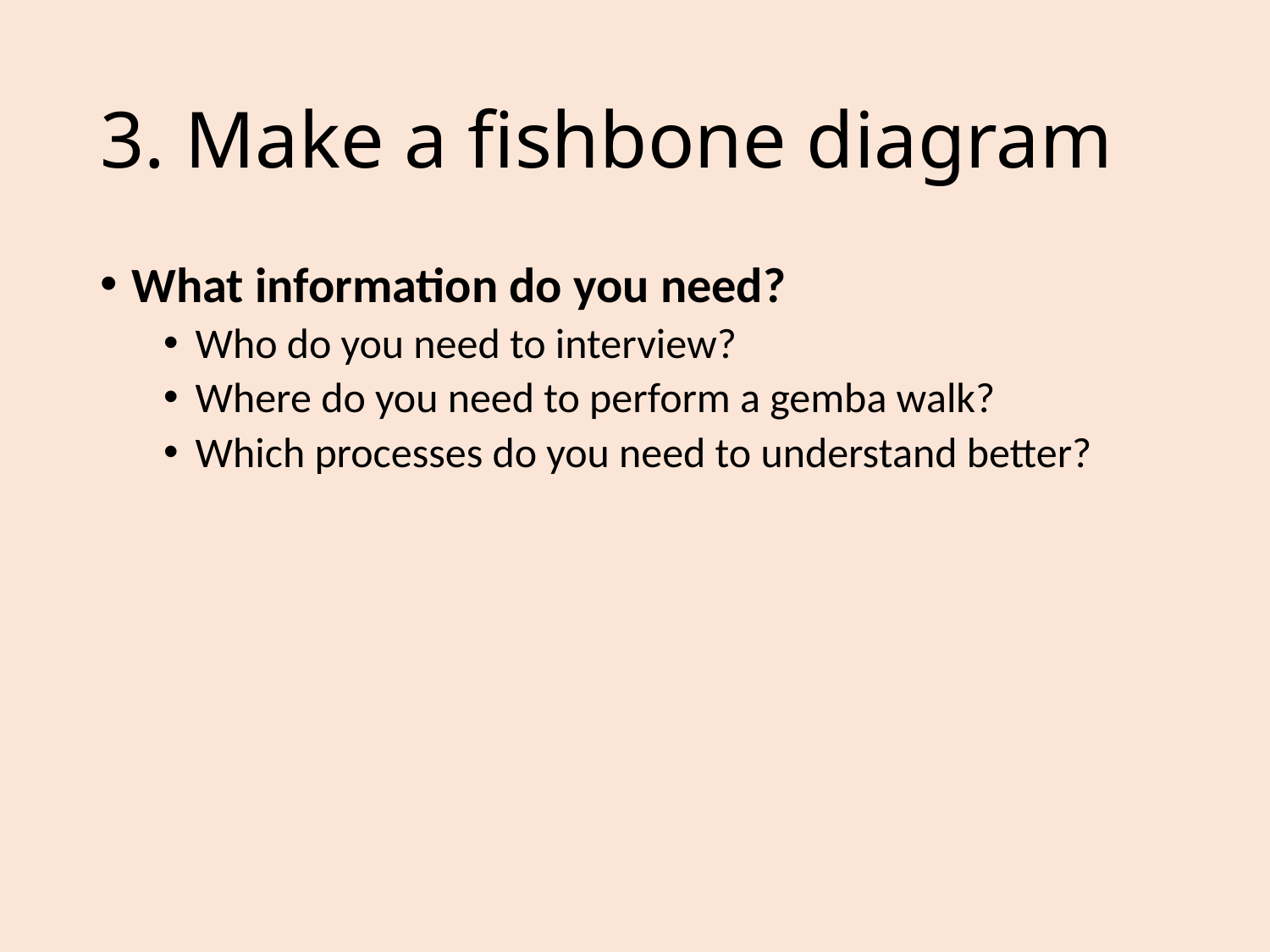

# 3. Make a fishbone diagram
What information do you need?
Who do you need to interview?
Where do you need to perform a gemba walk?
Which processes do you need to understand better?

## Slide 12
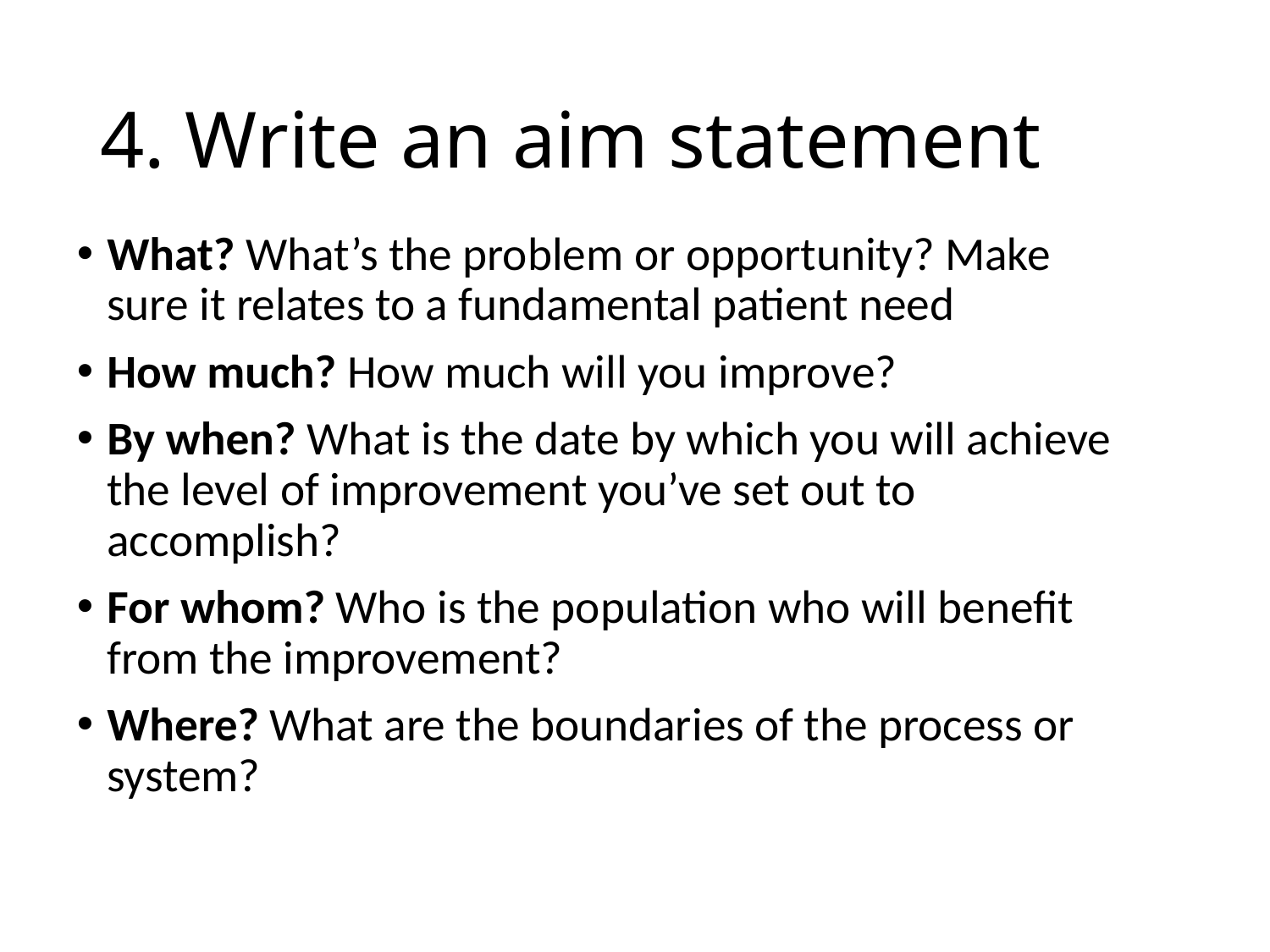

# 4. Write an aim statement
What? What’s the problem or opportunity? Make sure it relates to a fundamental patient need
How much? How much will you improve?
By when? What is the date by which you will achieve the level of improvement you’ve set out to accomplish?
For whom? Who is the population who will benefit from the improvement?
Where? What are the boundaries of the process or system?

## Slide 13
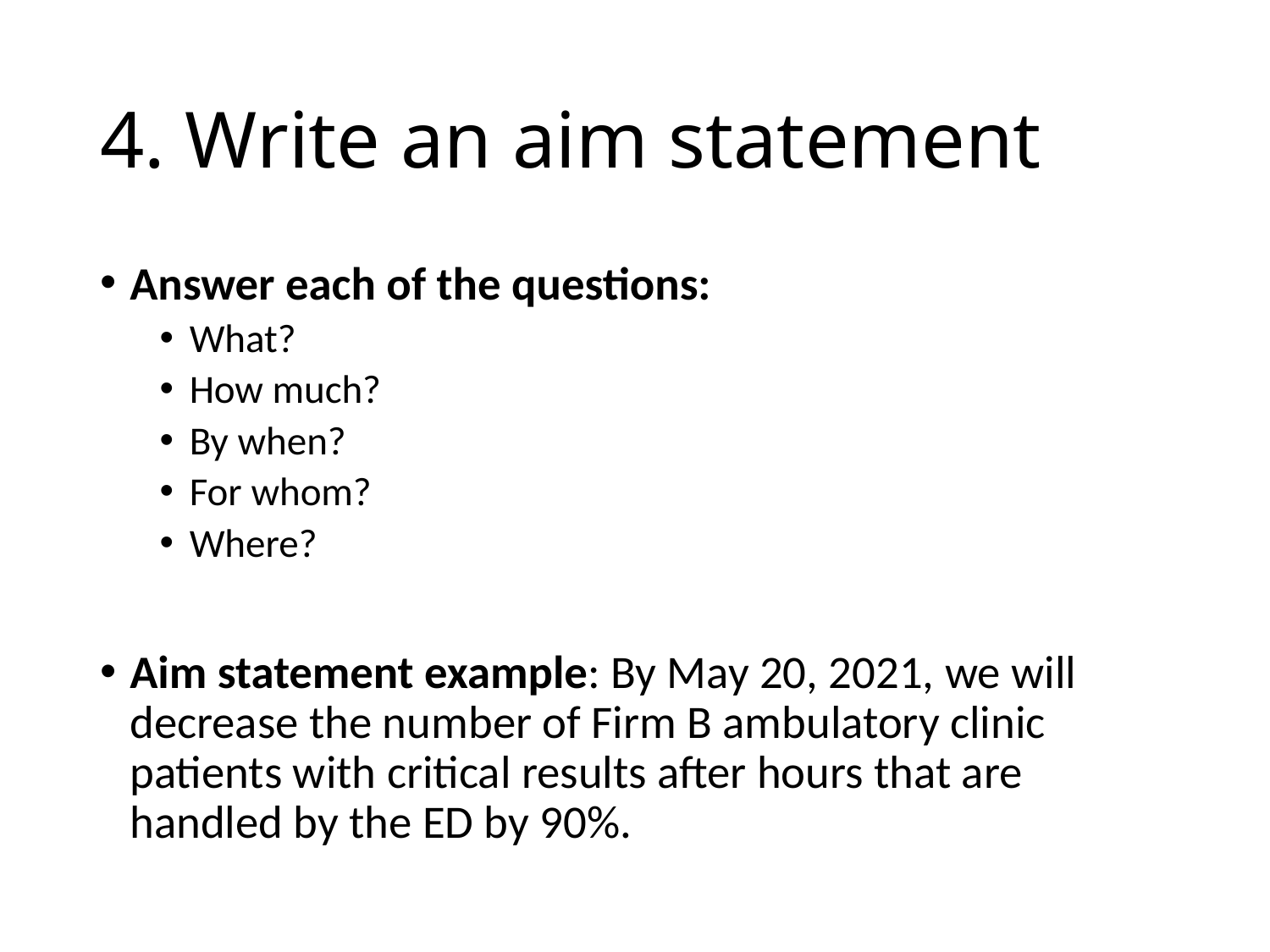

# 4. Write an aim statement
Answer each of the questions:
What?
How much?
By when?
For whom?
Where?
Aim statement example: By May 20, 2021, we will decrease the number of Firm B ambulatory clinic patients with critical results after hours that are handled by the ED by 90%.

## Slide 14
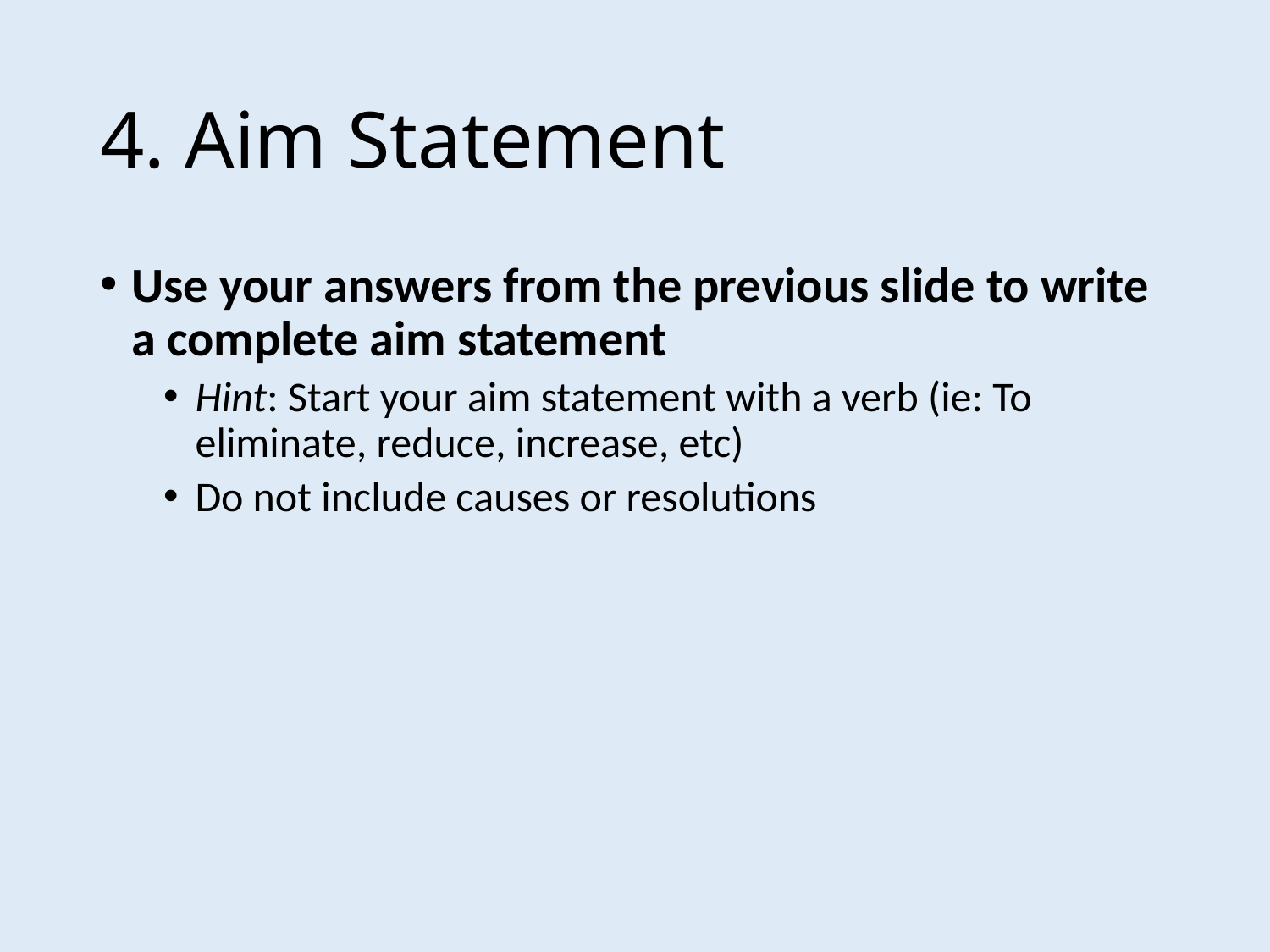

# 4. Aim Statement
Use your answers from the previous slide to write a complete aim statement
Hint: Start your aim statement with a verb (ie: To eliminate, reduce, increase, etc)
Do not include causes or resolutions

## Slide 15
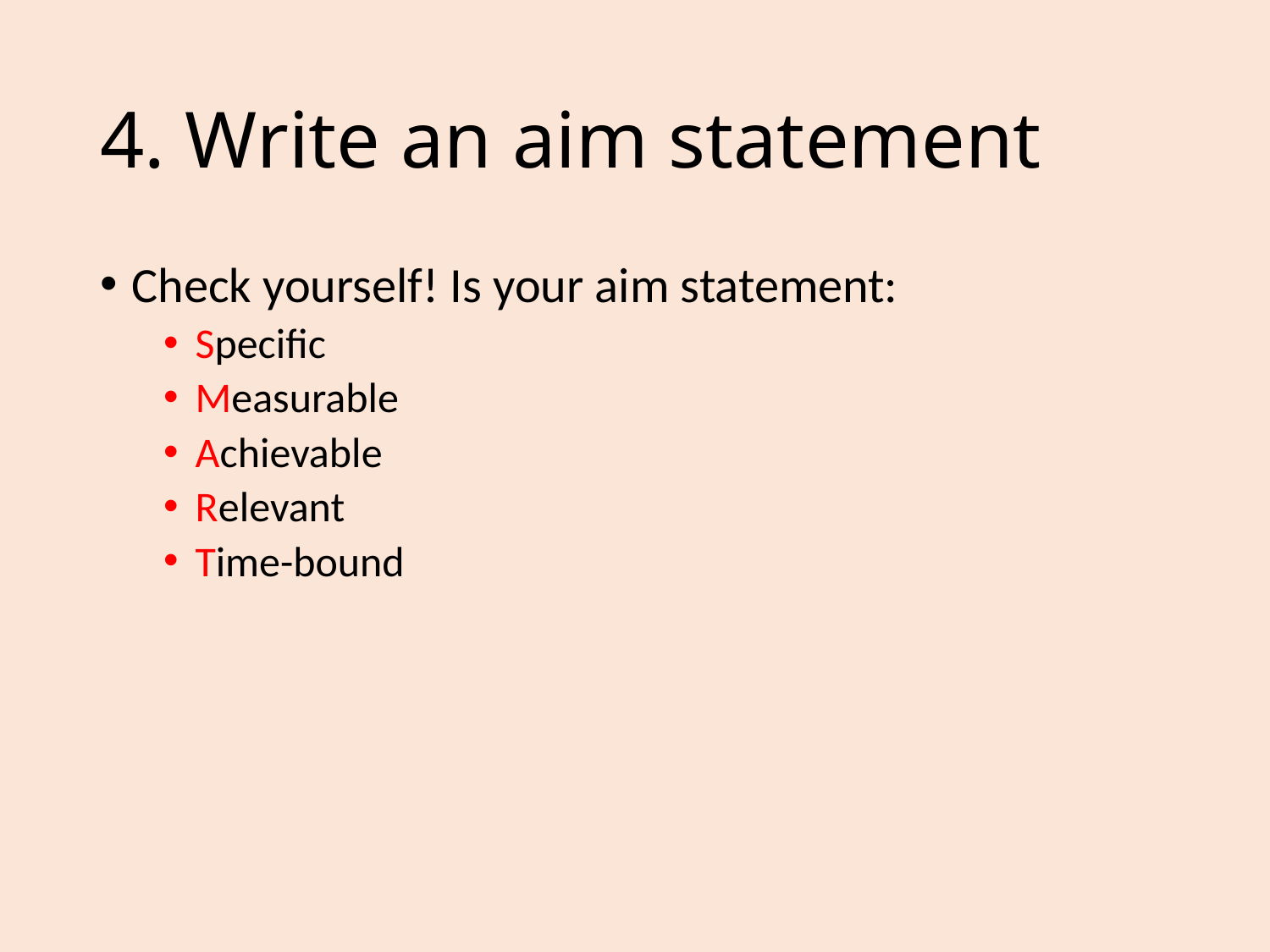

# 4. Write an aim statement
Check yourself! Is your aim statement:
Specific
Measurable
Achievable
Relevant
Time-bound

## Slide 16
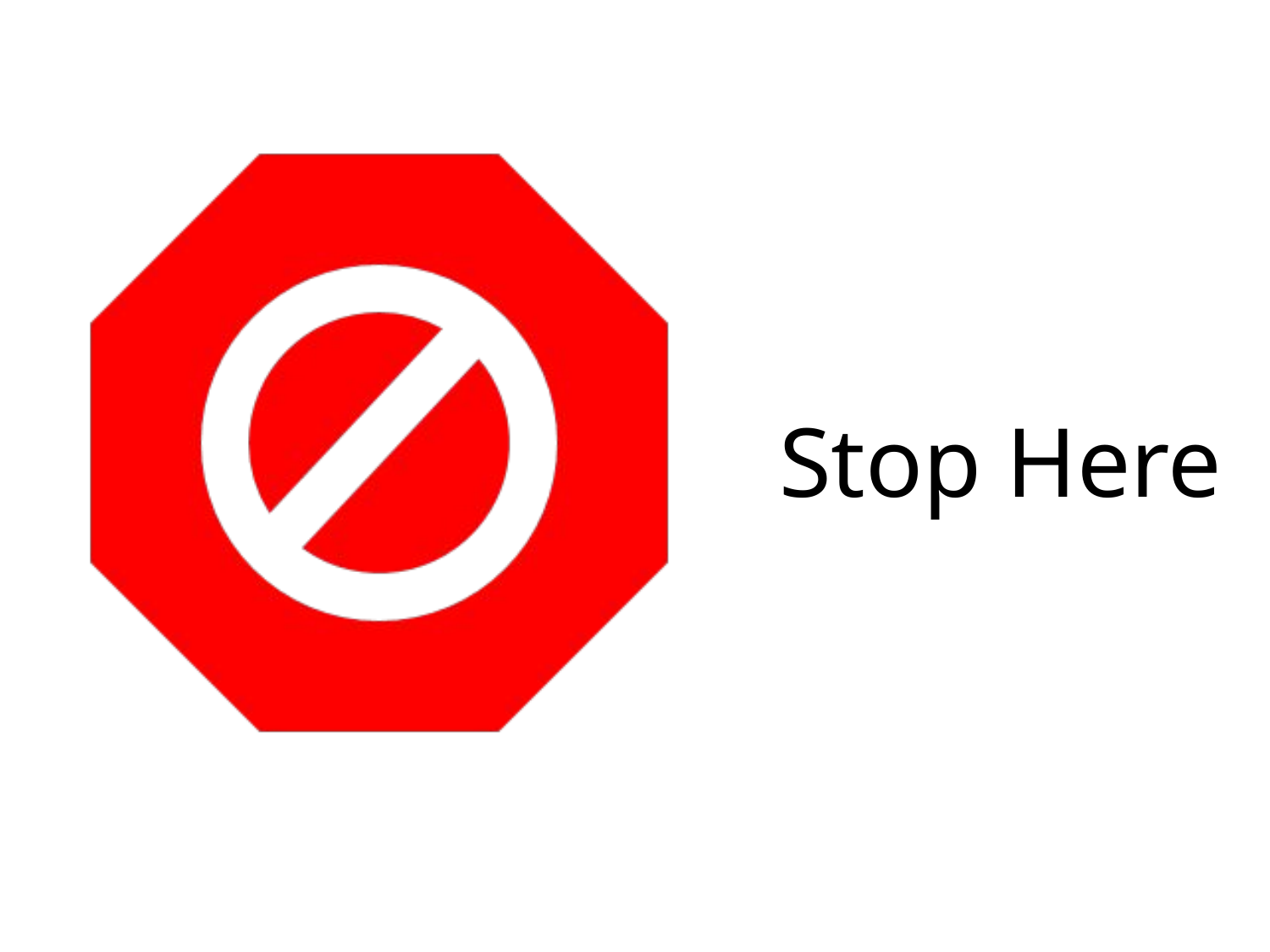

# Stop Here

## Slide 17
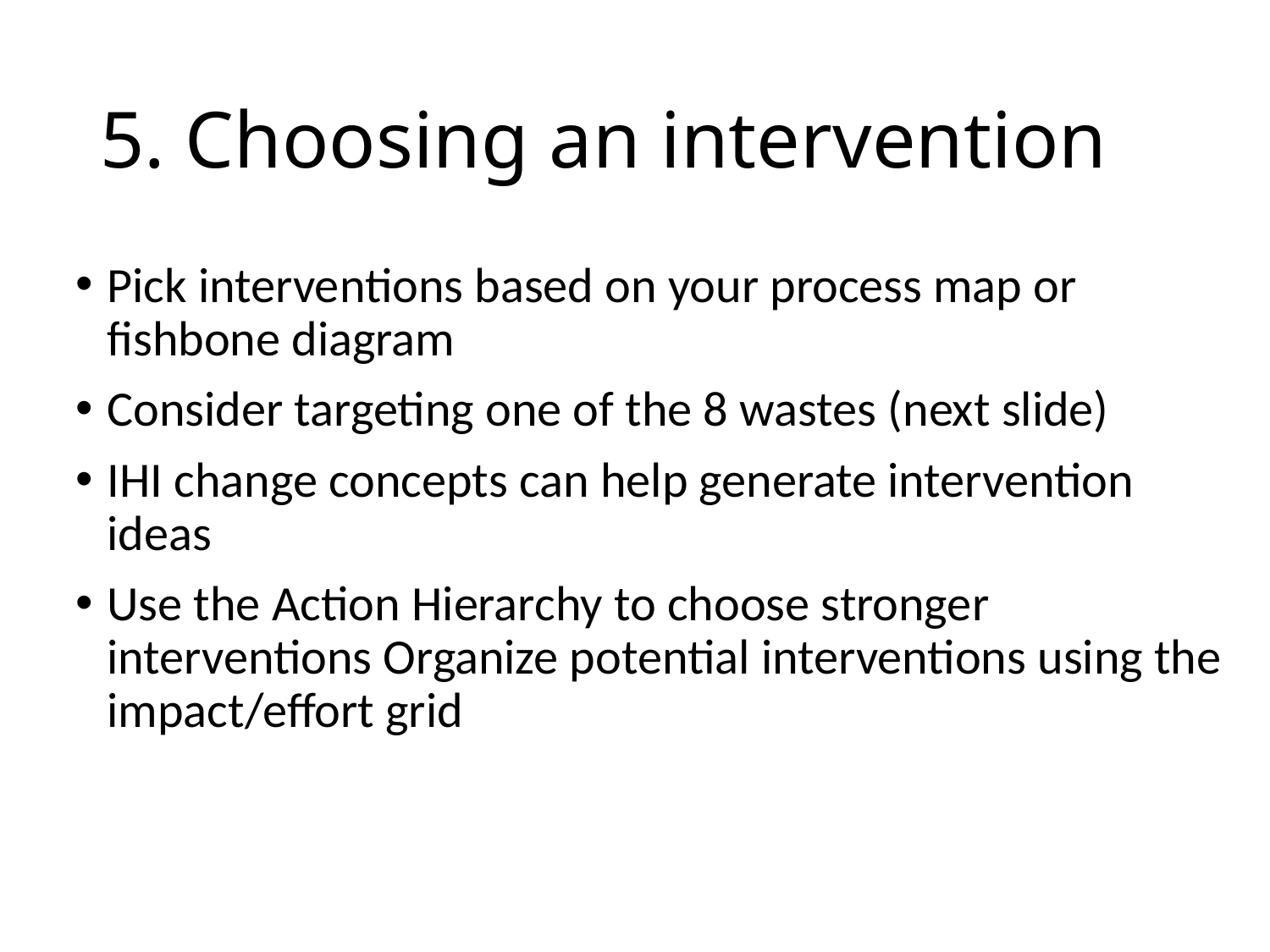

# 5. Choosing an intervention
Pick interventions based on your process map or fishbone diagram
Consider targeting one of the 8 wastes (next slide)
IHI change concepts can help generate intervention ideas
Use the Action Hierarchy to choose stronger interventions Organize potential interventions using the impact/effort grid

## Slide 18
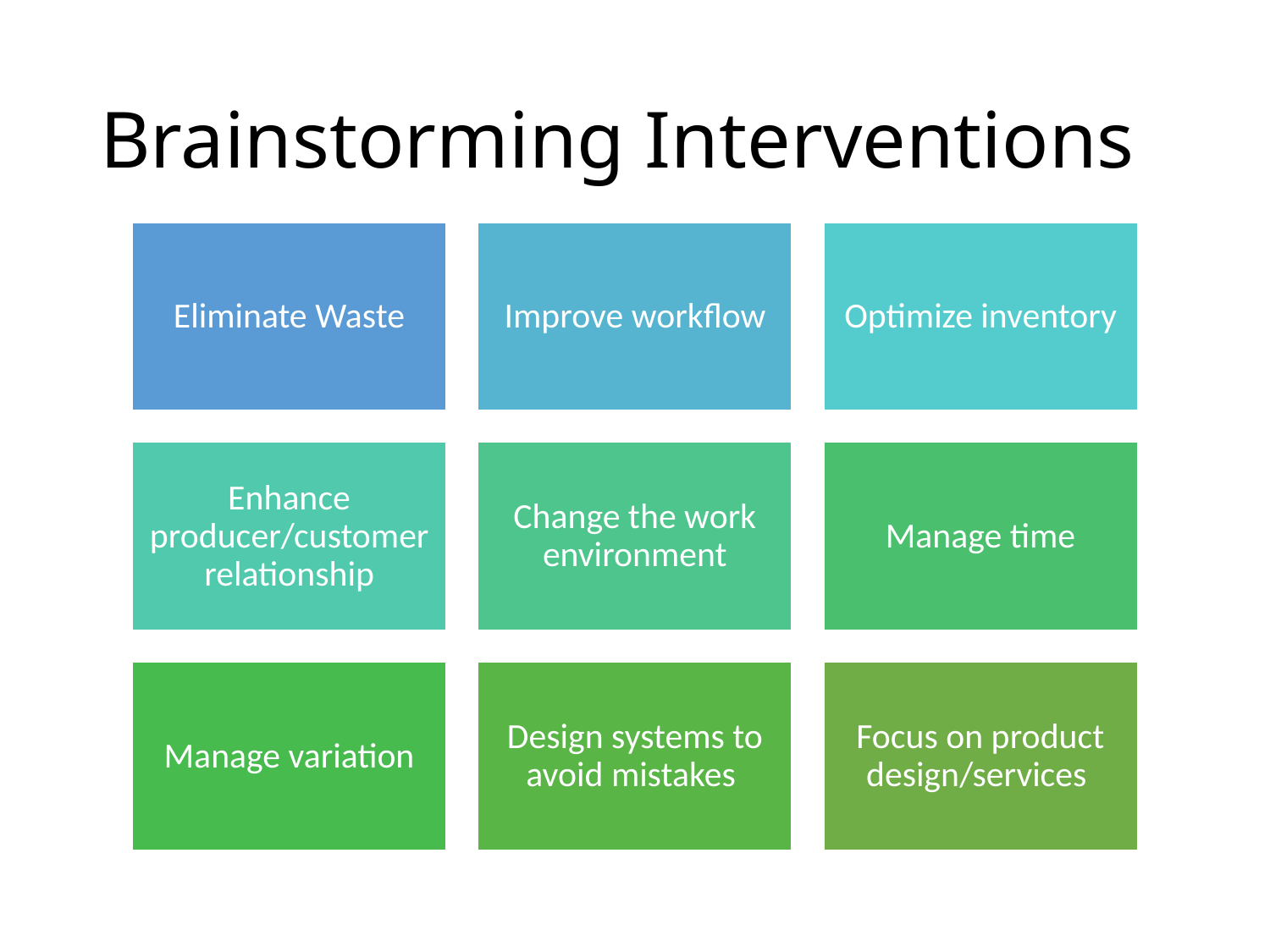

# Brainstorming Interventions

## Slide 19
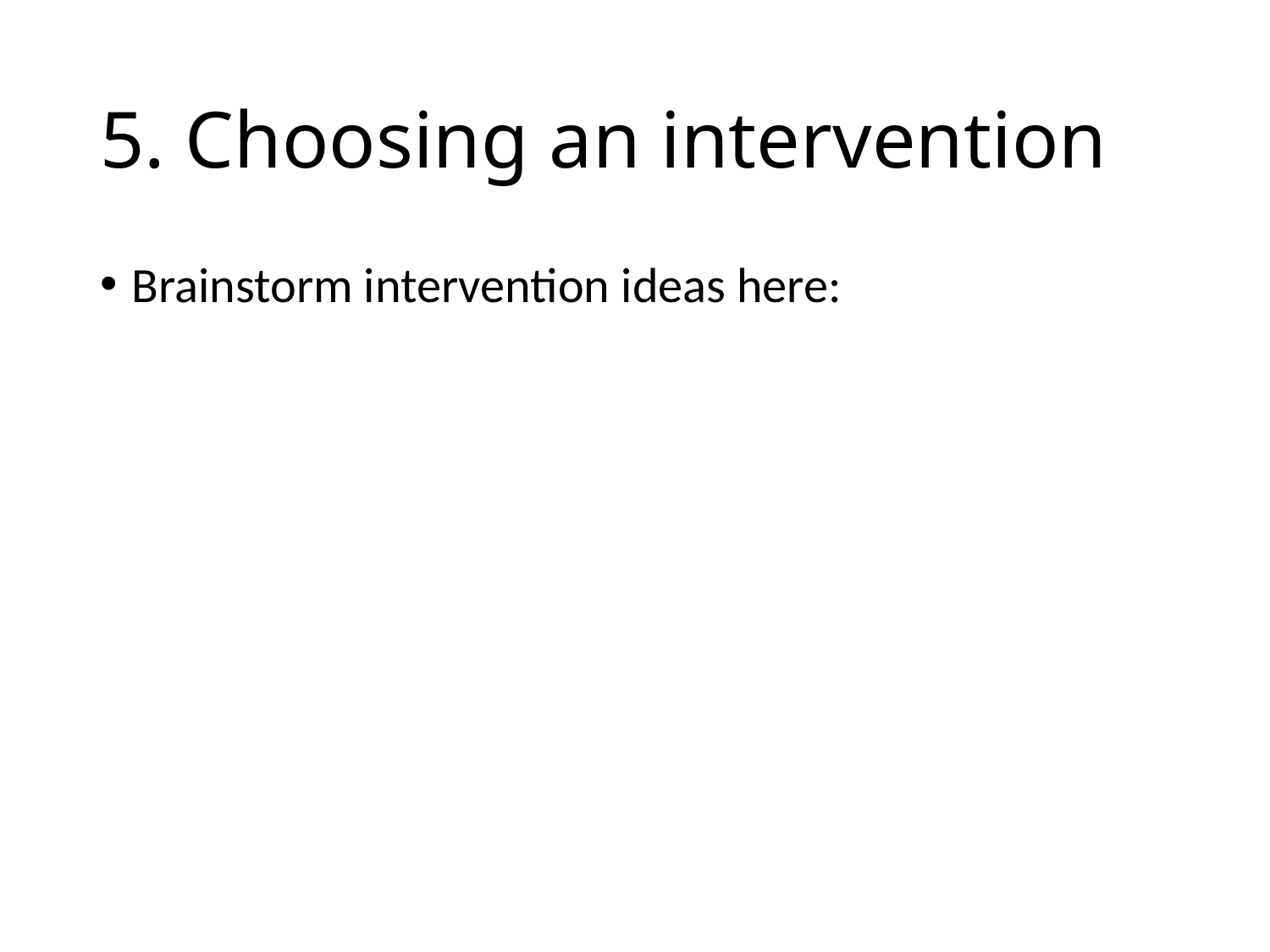

# 5. Choosing an intervention
Brainstorm intervention ideas here:

## Slide 20
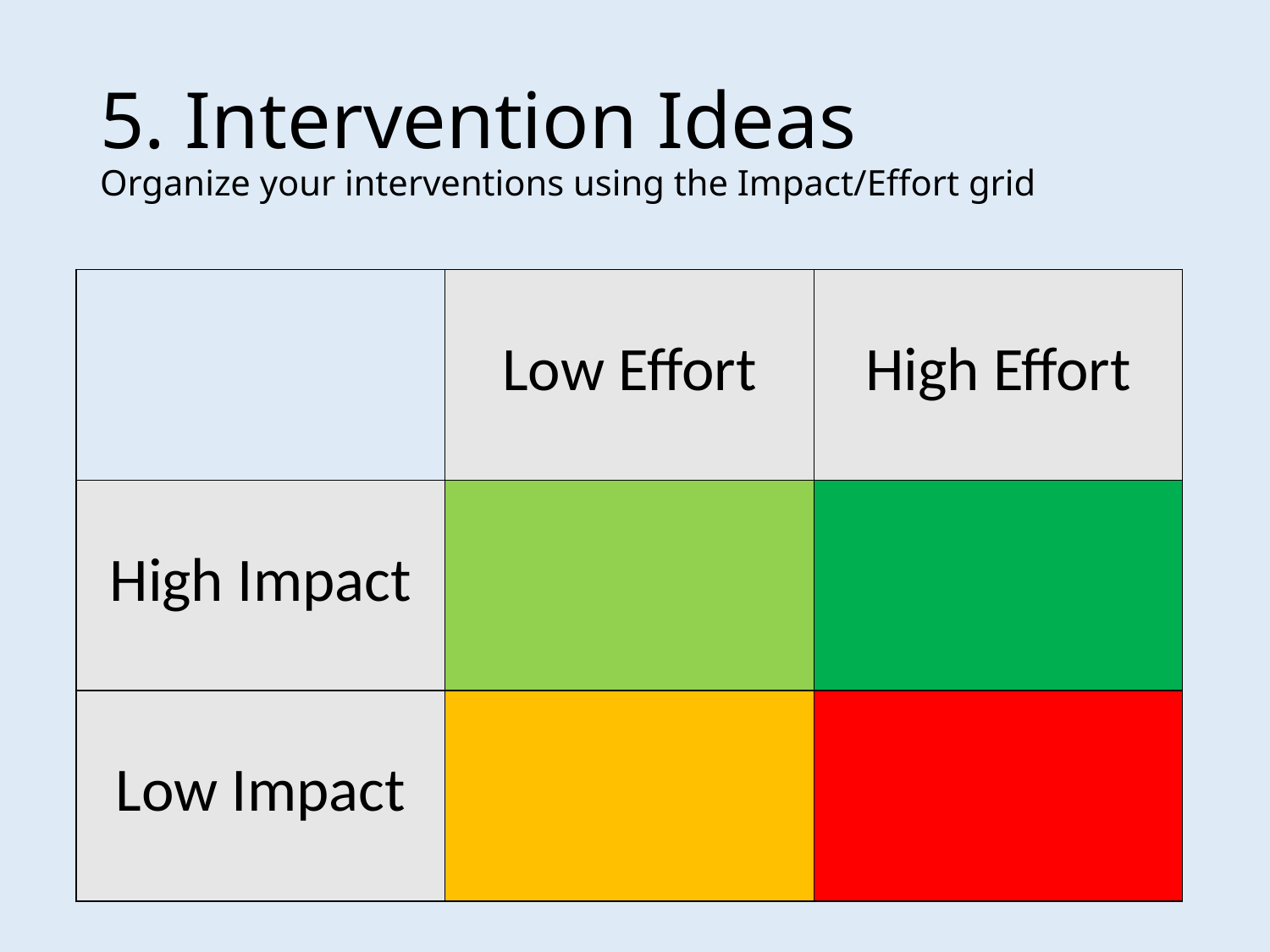

# 5. Intervention Ideas Organize your interventions using the Impact/Effort grid
| ​ | Low Effort​ | High Effort​ |
| --- | --- | --- |
| High Impact​ | ​ | ​ |
| Low Impact​ | ​ | ​ |

## Slide 21
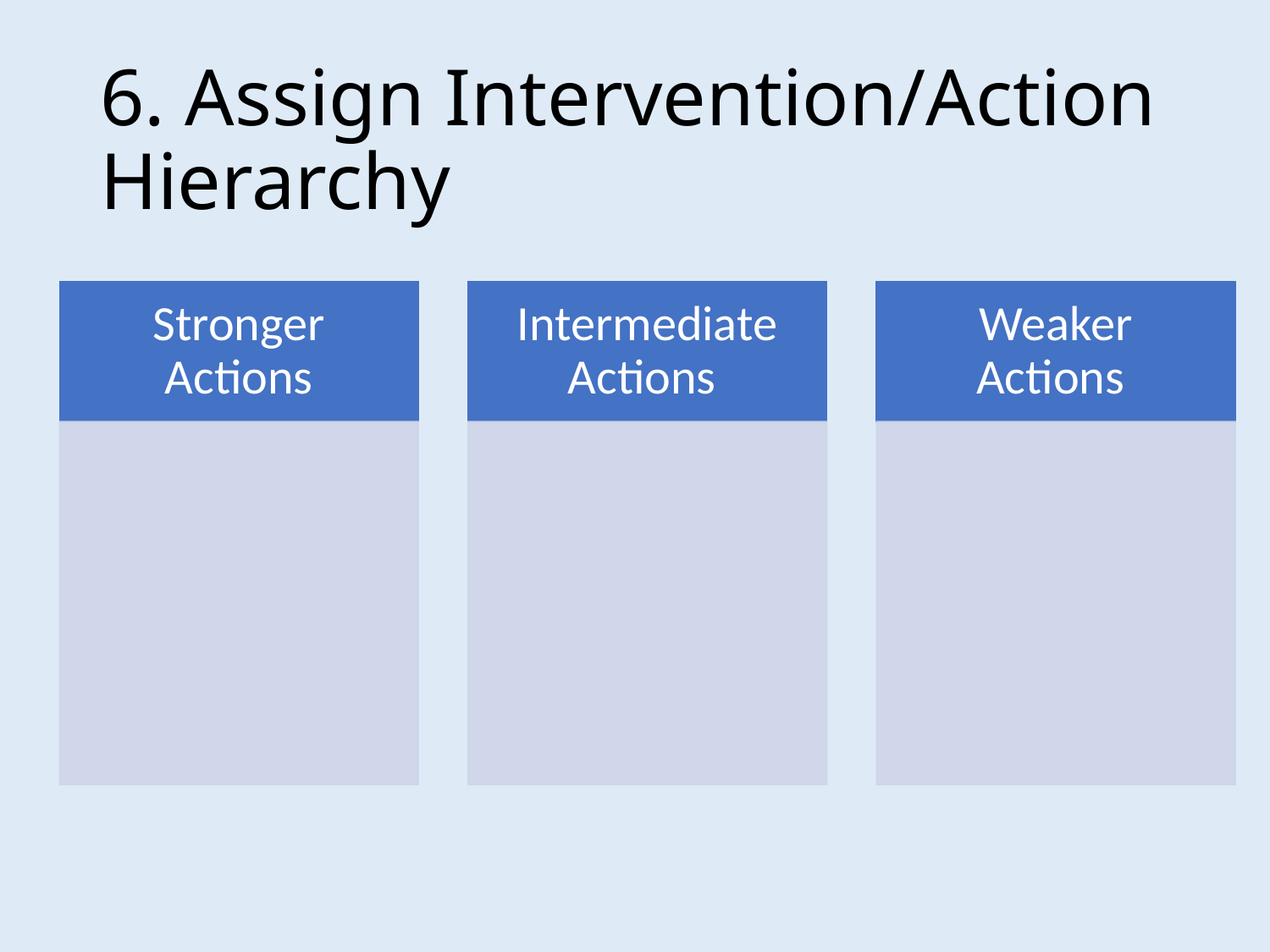

# 6. Assign Intervention/Action Hierarchy

## Slide 22
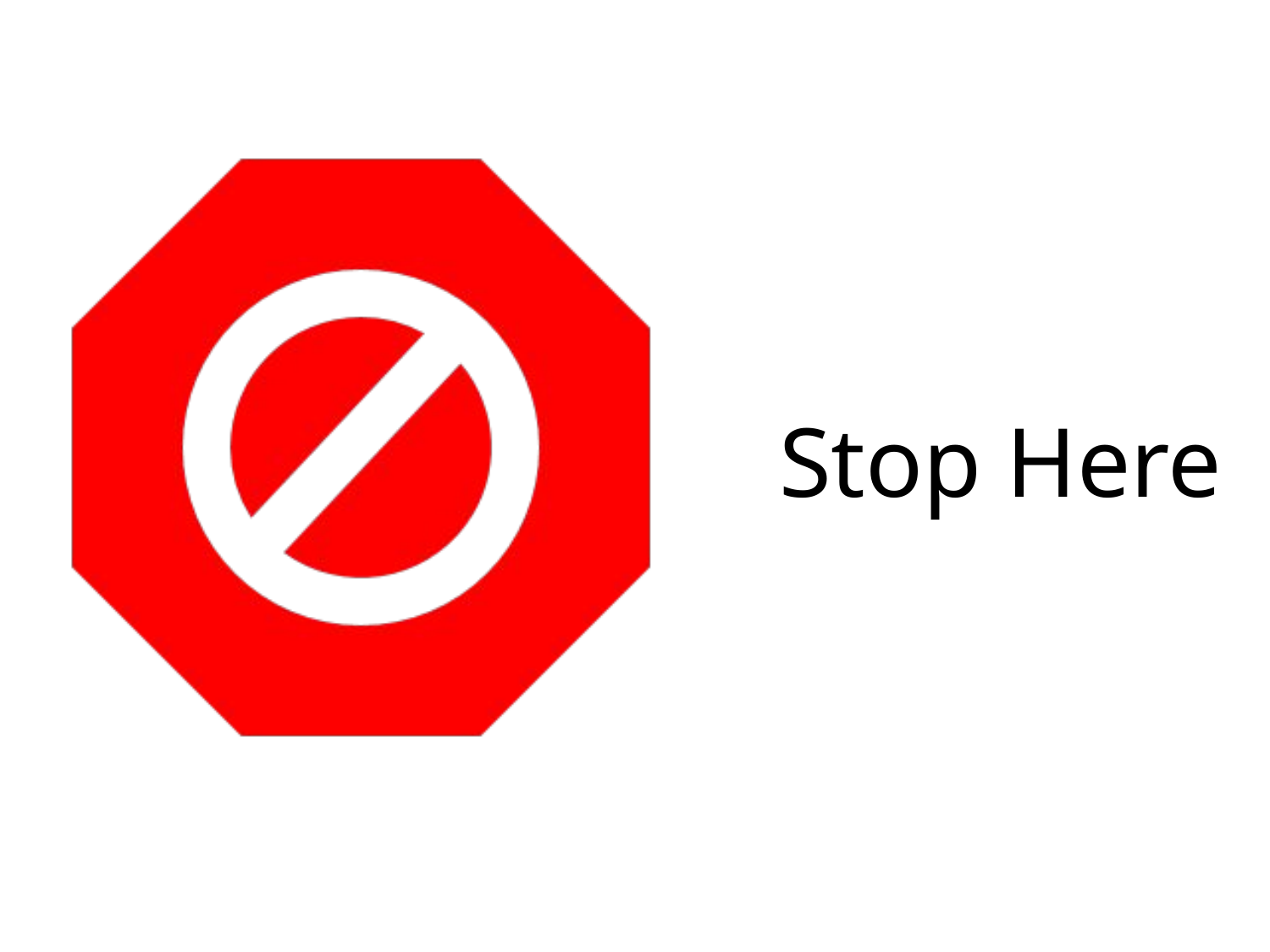

# Stop Here

## Slide 23
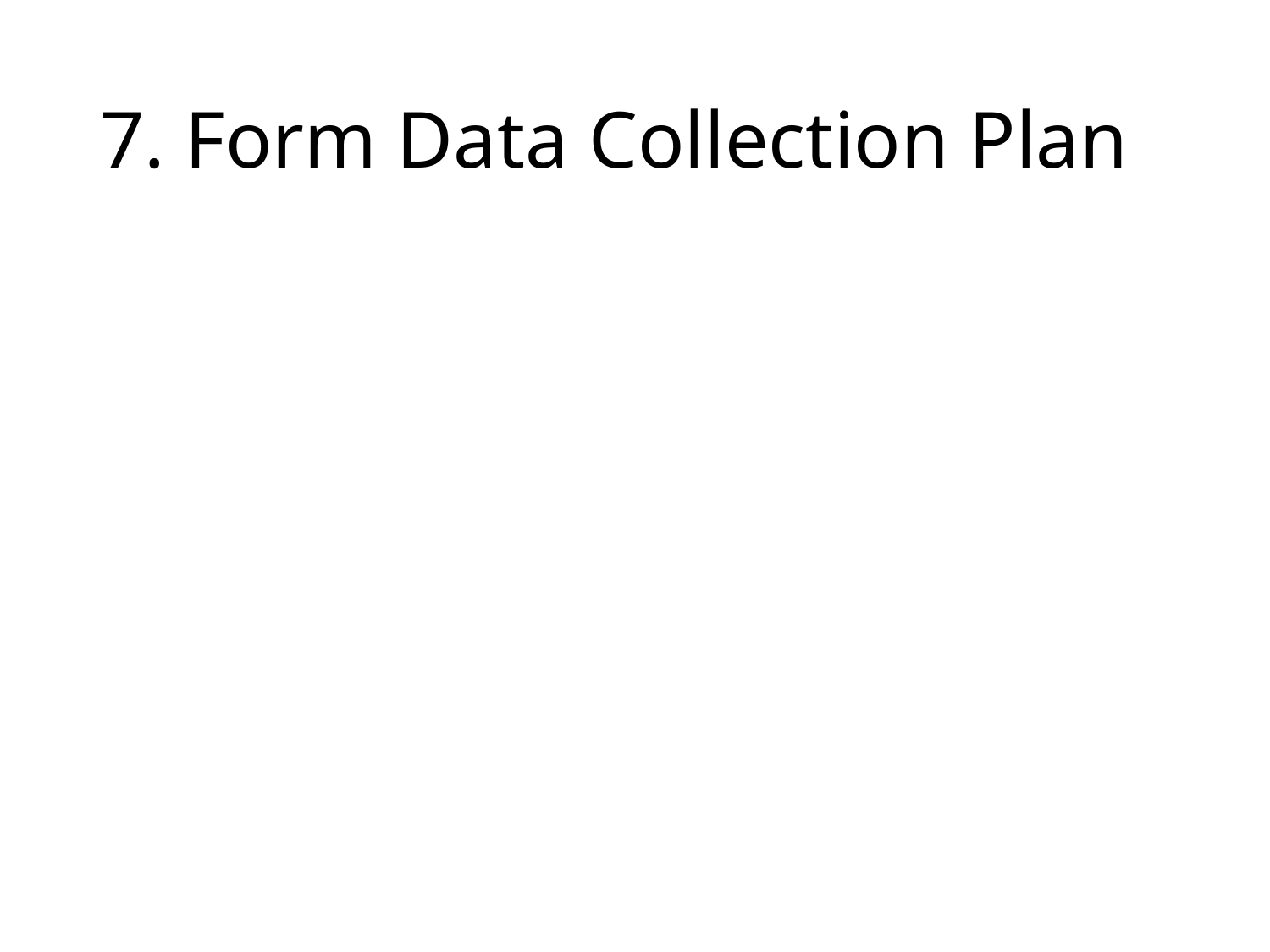

# 7. Form Data Collection Plan

## Slide 24
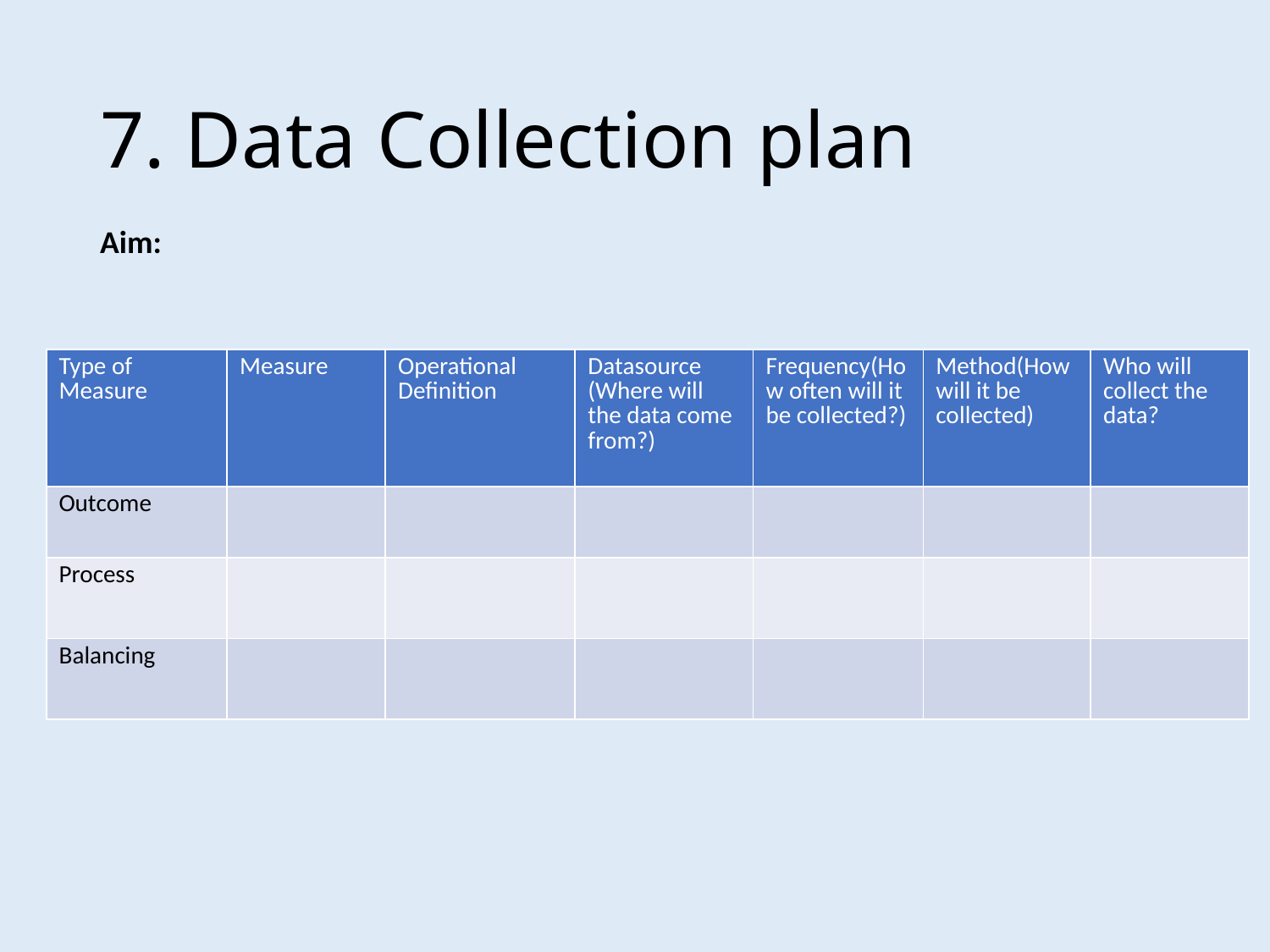

# 7. Data Collection plan
Aim:
| Type of Measure​ | Measure​ | Operational Definition​ | Datasource​ (Where will the data come from?)​ | Frequency(How often will it be collected?)​ ​ | Method(How will it be collected)​ ​ | Who will collect the data?​ |
| --- | --- | --- | --- | --- | --- | --- |
| ​Outcome | ​ | ​ | ​ | ​ | ​ | ​ |
| ​Process | ​ | ​ | ​ | ​ | ​ | ​ |
| ​Balancing | ​ | ​ | ​ | ​ | ​ | ​ |
