# Supplementary material for: Building Resident Quality Improvement Knowledge and Engagement Through a Longitudinal, Mentored, and Experiential Learning-Based Quality Improvement Curriculum
Source: MedEdPORTAL. 2023 Apr 18;19:11310. doi: 10.15766/mep_2374-8265.11310 (PMC10110773; doi:10.15766/mep_2374-8265.11310)
Supplement: Supplementary file 1 — Session 1 Slides.pptxSession 1 Workbook.pptxSession 2 Slides.pptxSession 2 Workbook.pptxSession 3 Slides.pptxSession 4 Work-in-Progress Presentation Template.pptxSession 5 Slides.pptxQI Charter Template.docxFaculty Milestones.docxFaculty Guide.docxResident Survey.docx [file mep_2374-8265.11310-s001.zip › E. Session 3 Slides.pptx]

## Slide 1
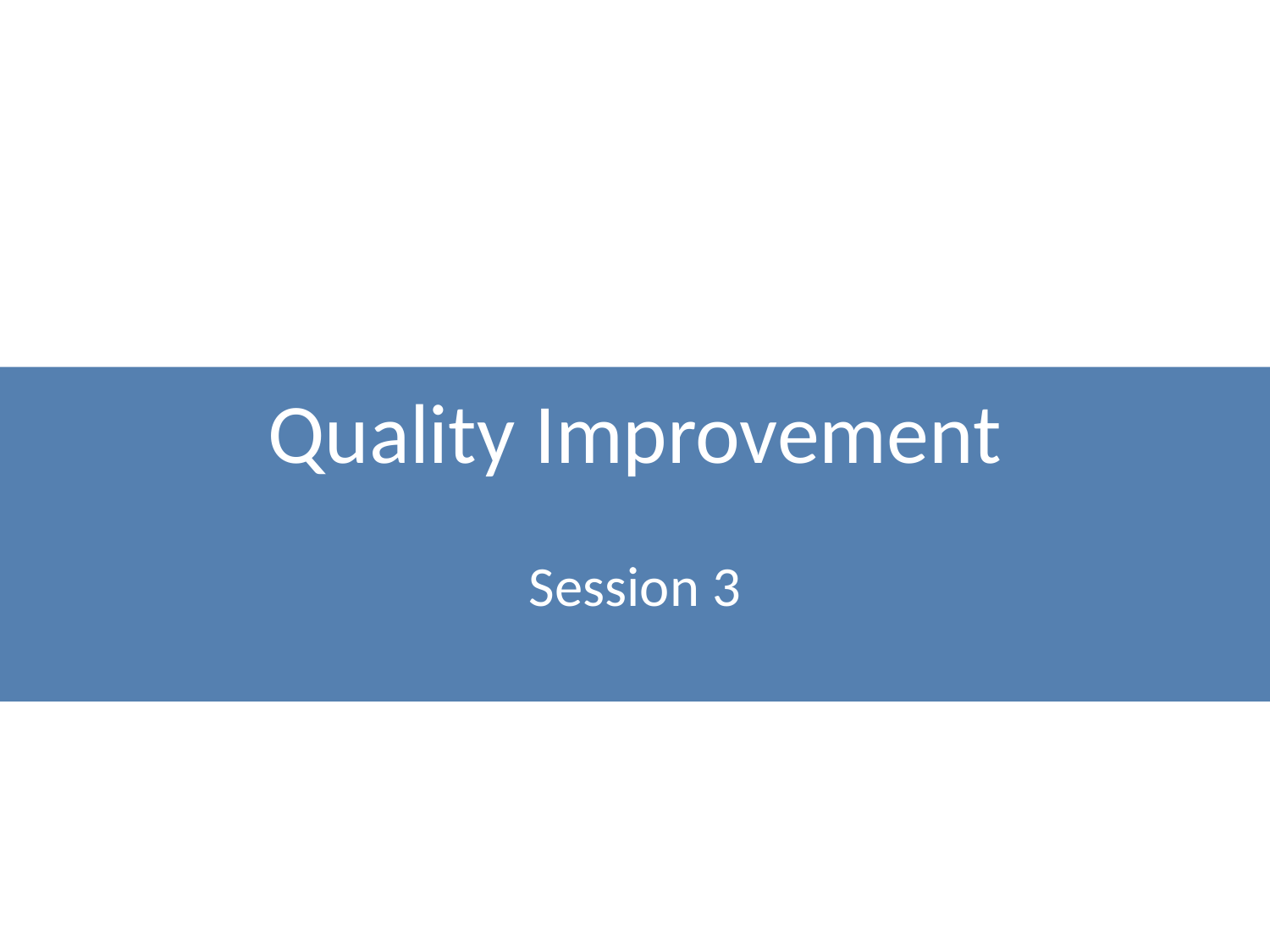

# Quality Improvement Session 3

## Slide 2
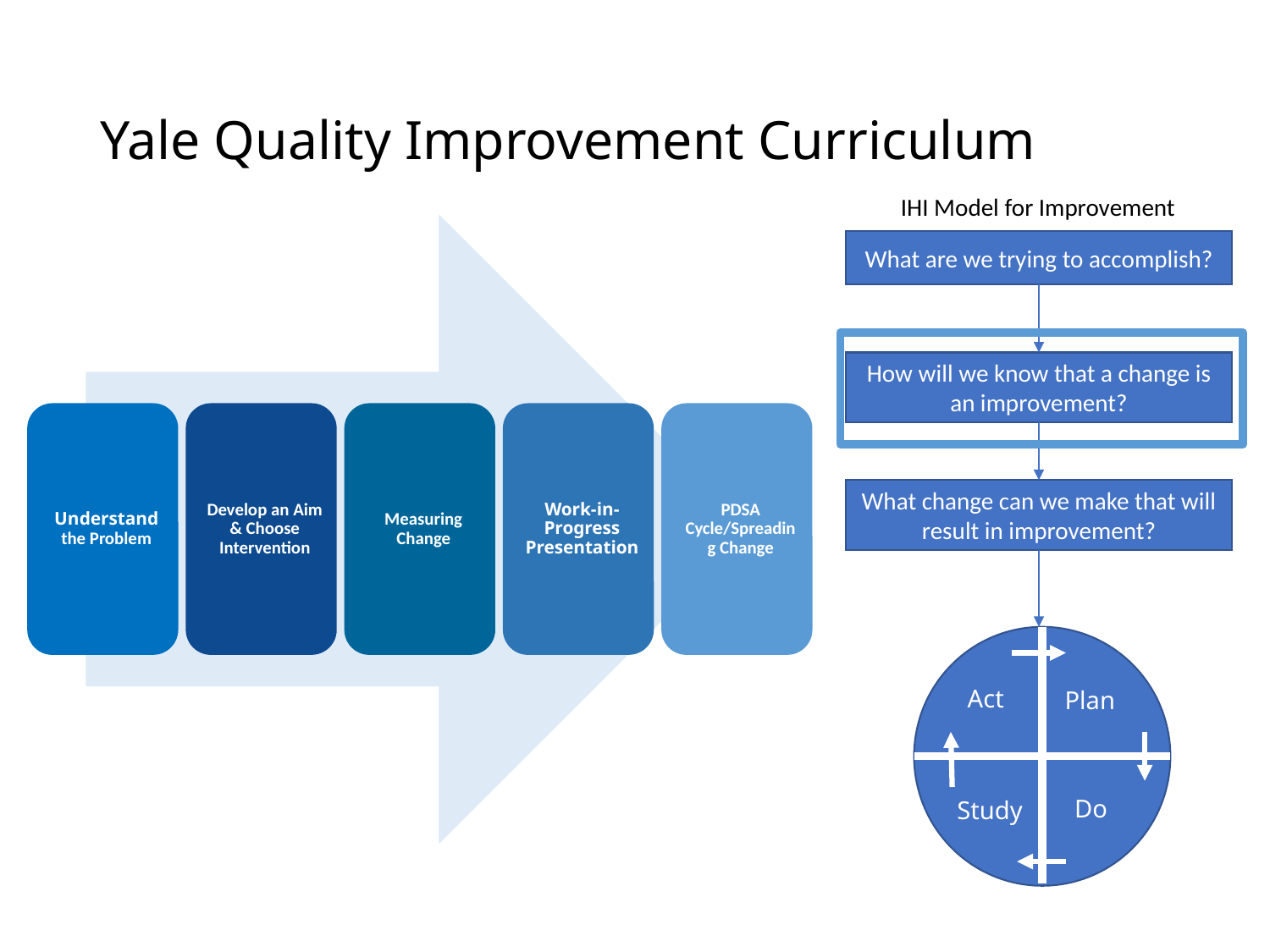

# Yale Quality Improvement Curriculum
IHI Model for Improvement
What are we trying to accomplish?
How will we know that a change is an improvement?
What change can we make that will result in improvement?
Act
Plan
Do
Study

## Slide 3
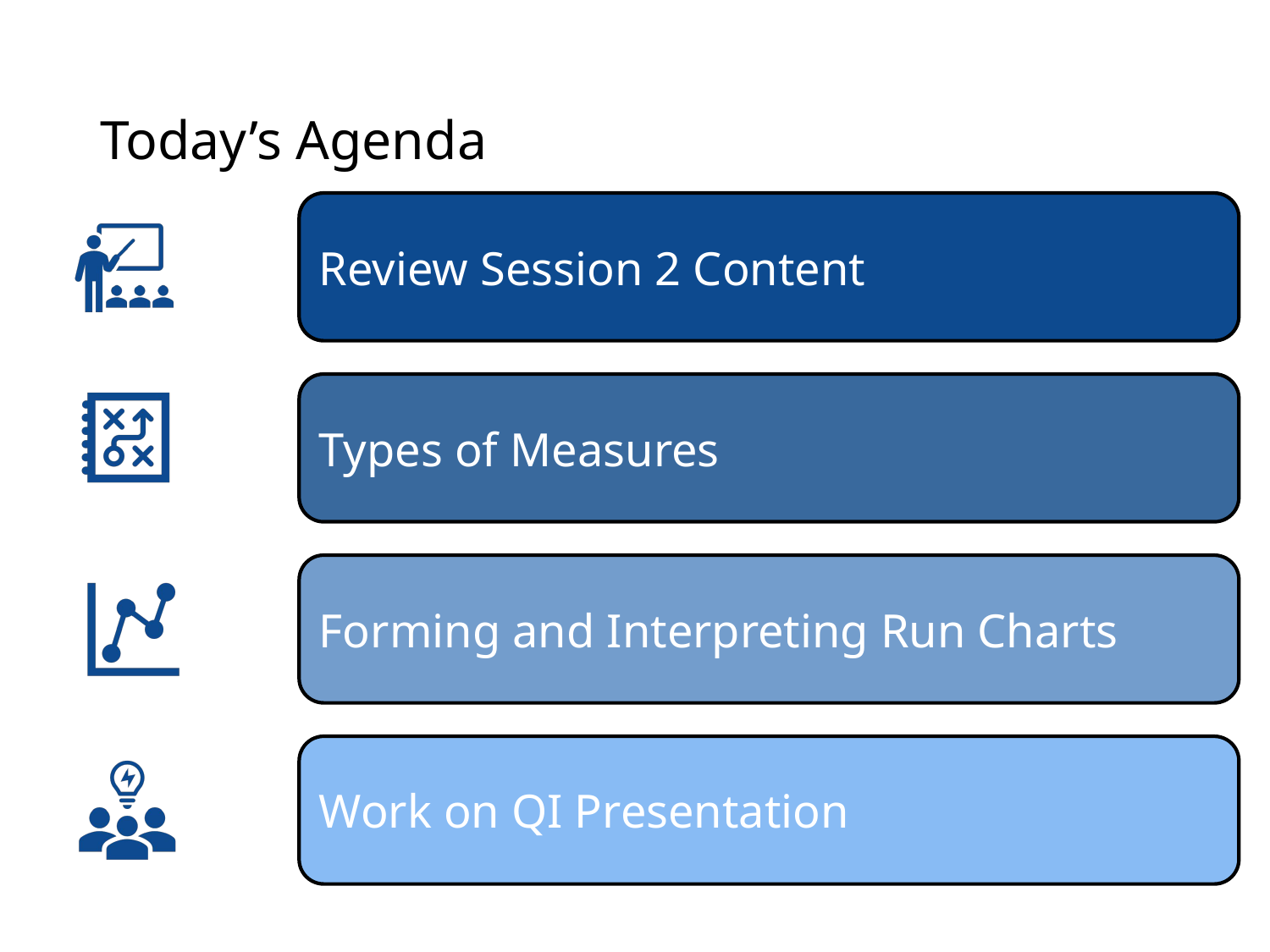

# Today’s Agenda
Review Session 2 Content
Types of Measures
Forming and Interpreting Run Charts
Work on QI Presentation

## Slide 4
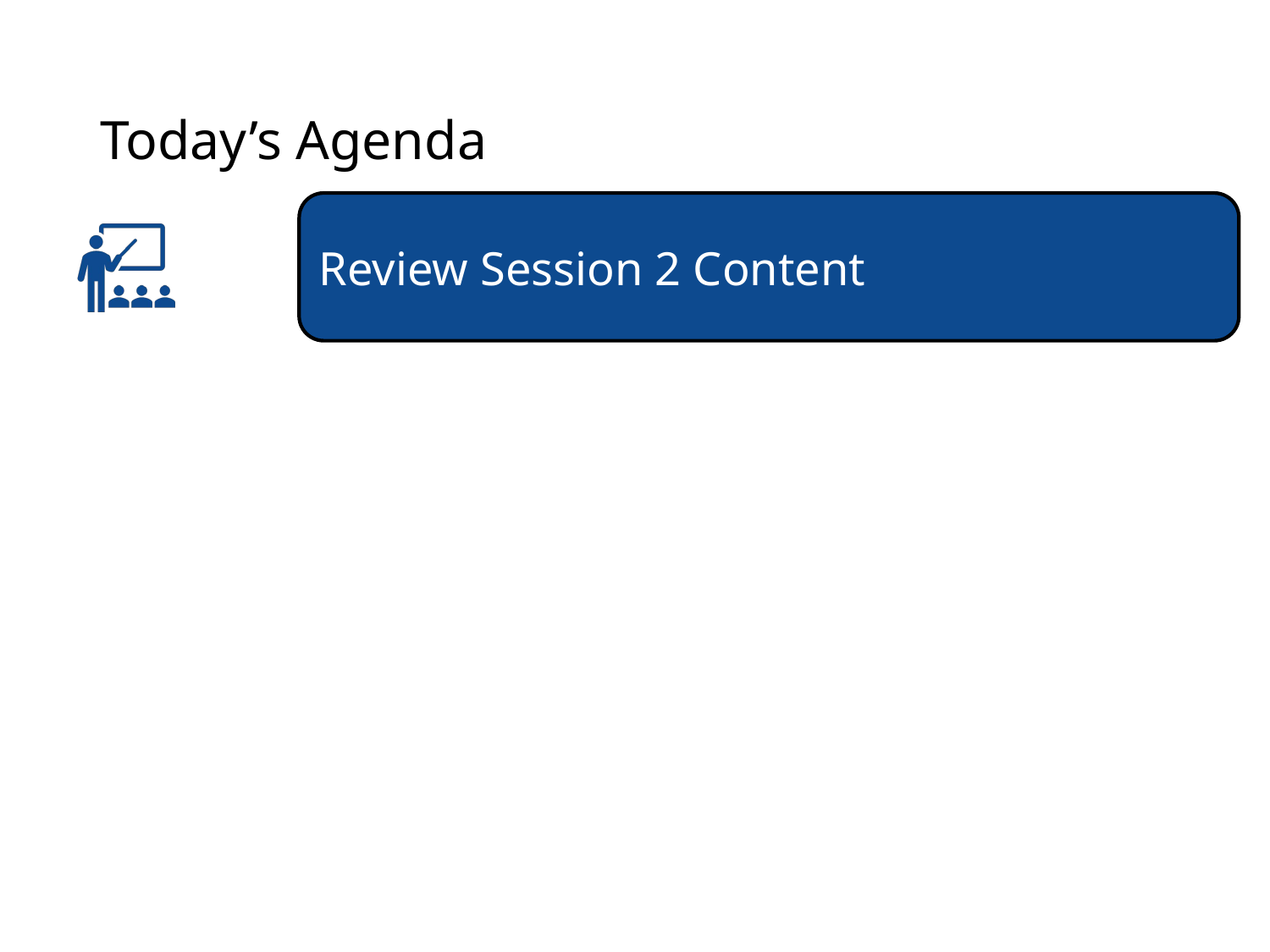

# Today’s Agenda
Review Session 2 Content

## Slide 5
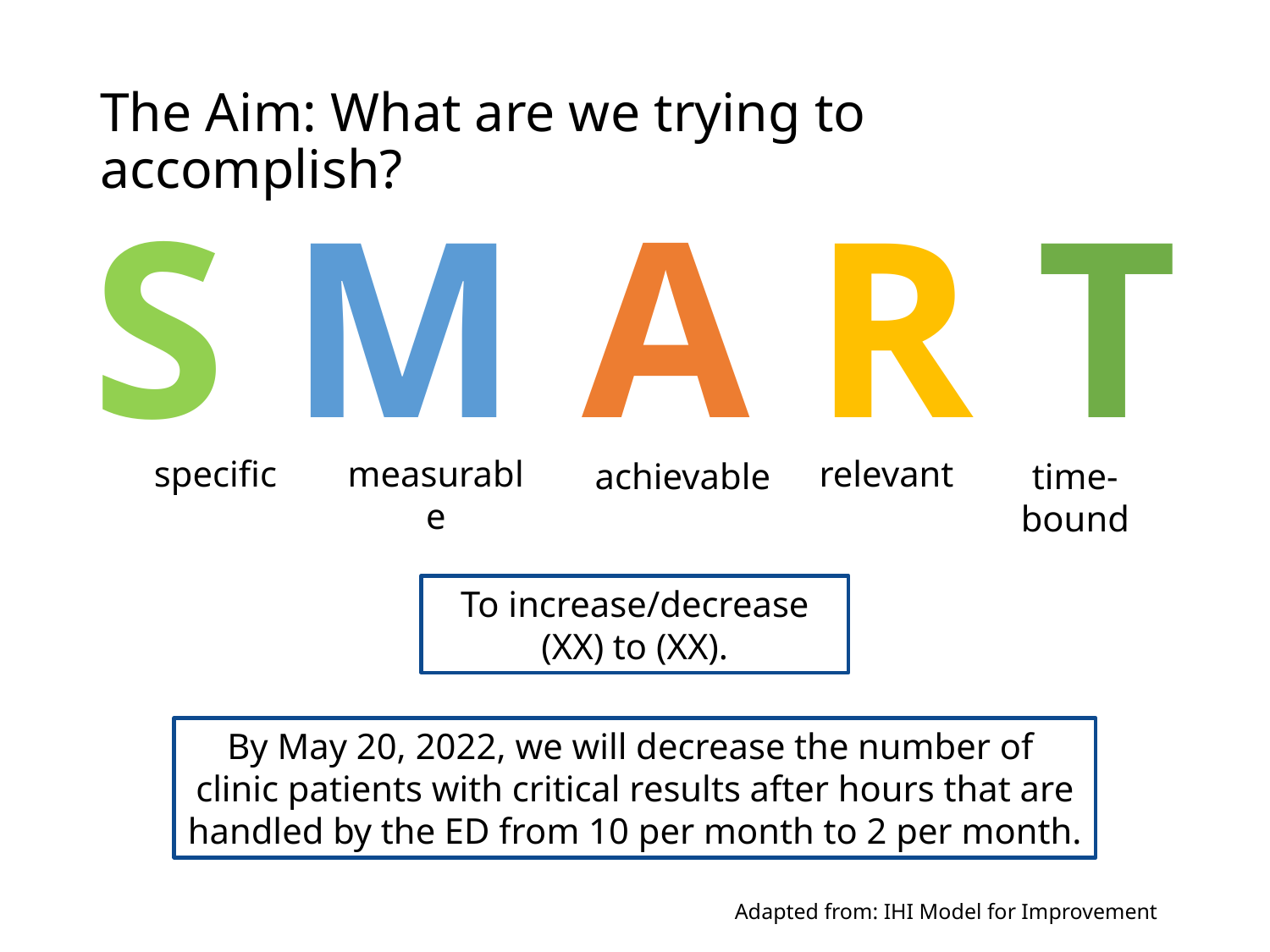

# The Aim: What are we trying to accomplish?
S M A R T
specific
relevant
measurable
achievable
time-bound
To increase/decrease
(XX) to (XX).
By May 20, 2022, we will decrease the number of
 clinic patients with critical results after hours that are
handled by the ED from 10 per month to 2 per month.
Adapted from: IHI Model for Improvement

## Slide 6
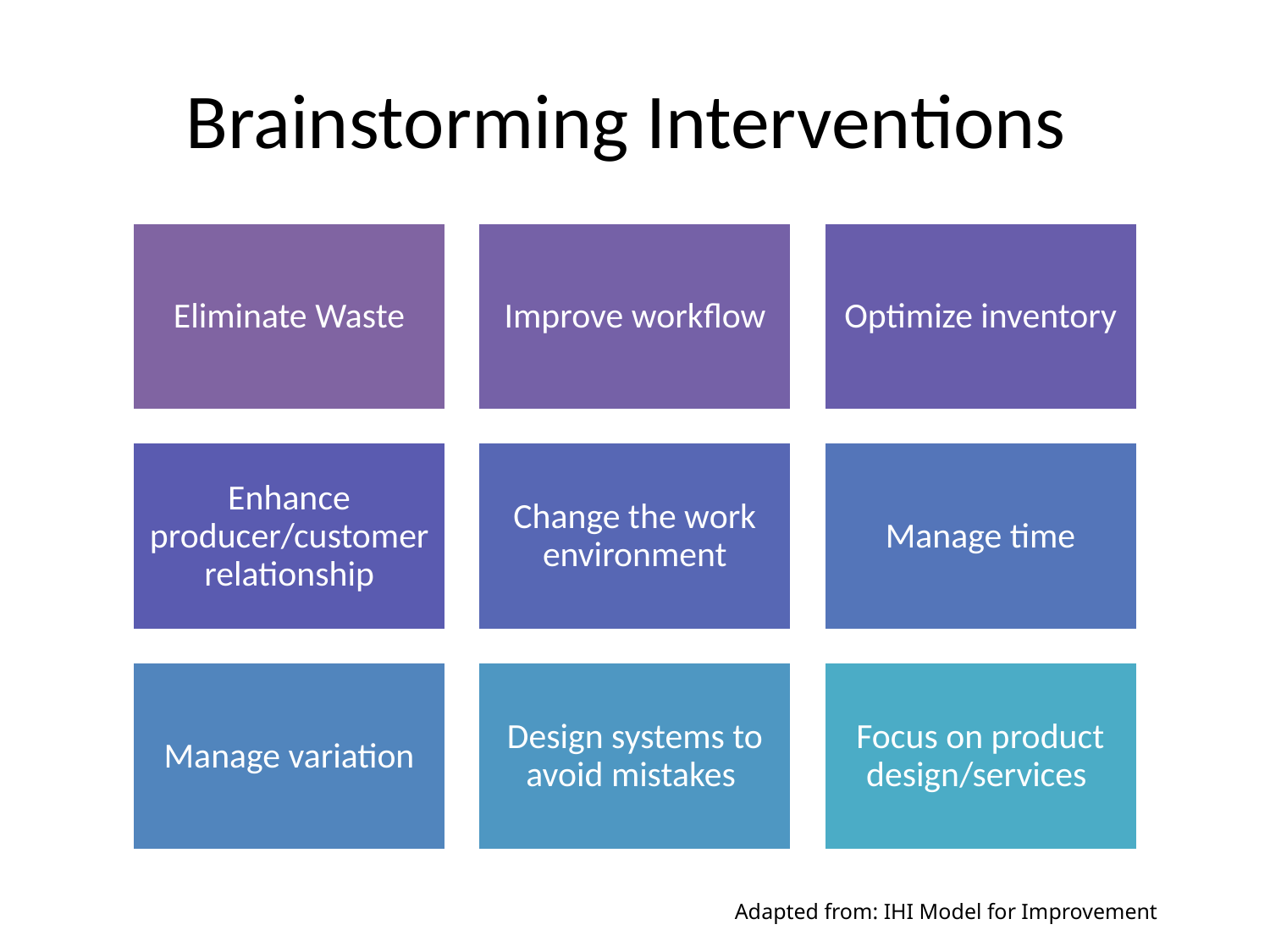

# Brainstorming Interventions
Adapted from: IHI Model for Improvement

## Slide 7
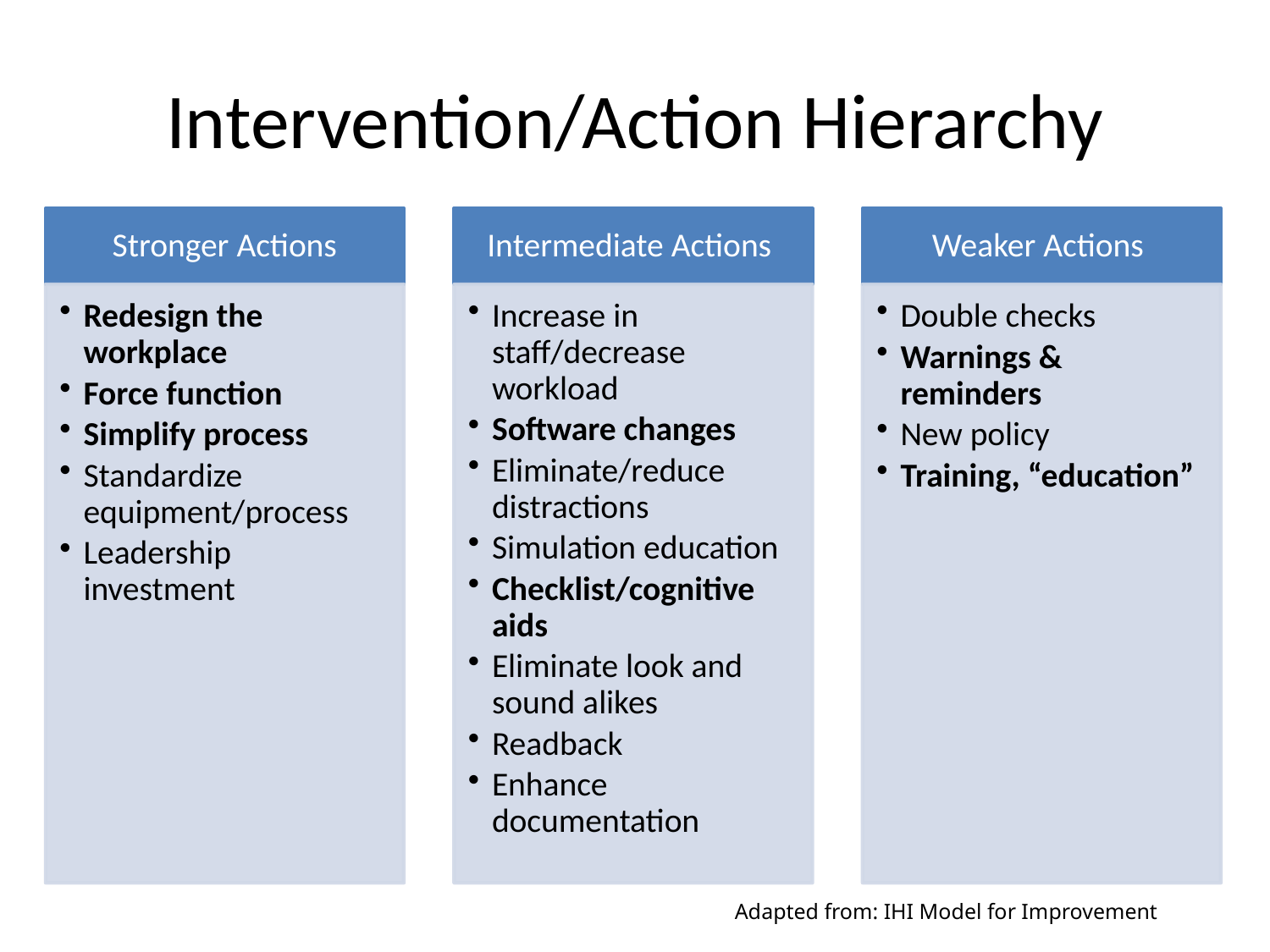

# Intervention/Action Hierarchy
Adapted from: IHI Model for Improvement

## Slide 8
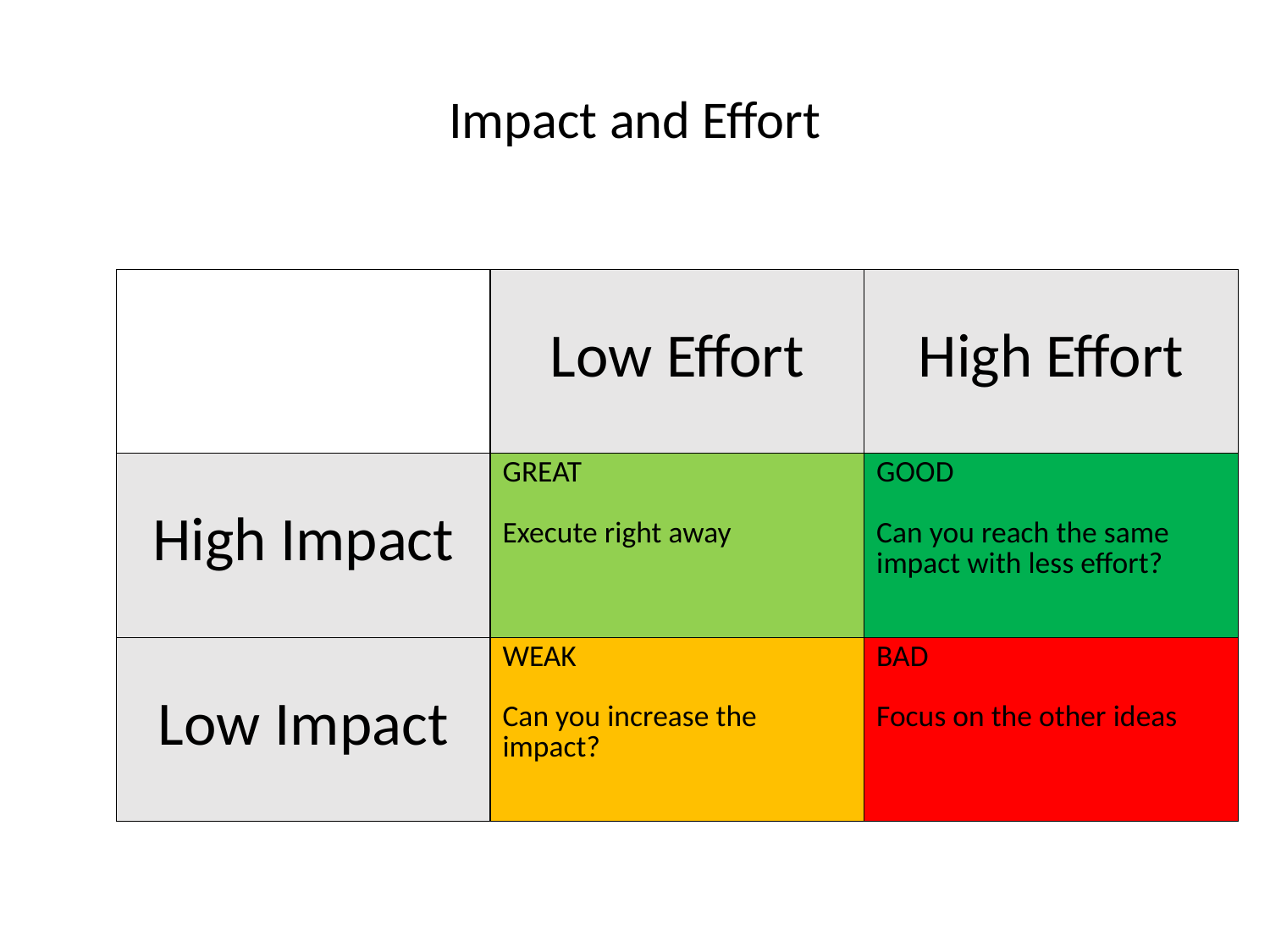

# Impact and Effort
| ​ | Low Effort​ | High Effort​ |
| --- | --- | --- |
| High Impact​ | ​GREAT Execute right away | ​GOOD Can you reach the same impact with less effort? |
| Low Impact​ | ​WEAK Can you increase the impact? | ​BAD Focus on the other ideas |

## Slide 9
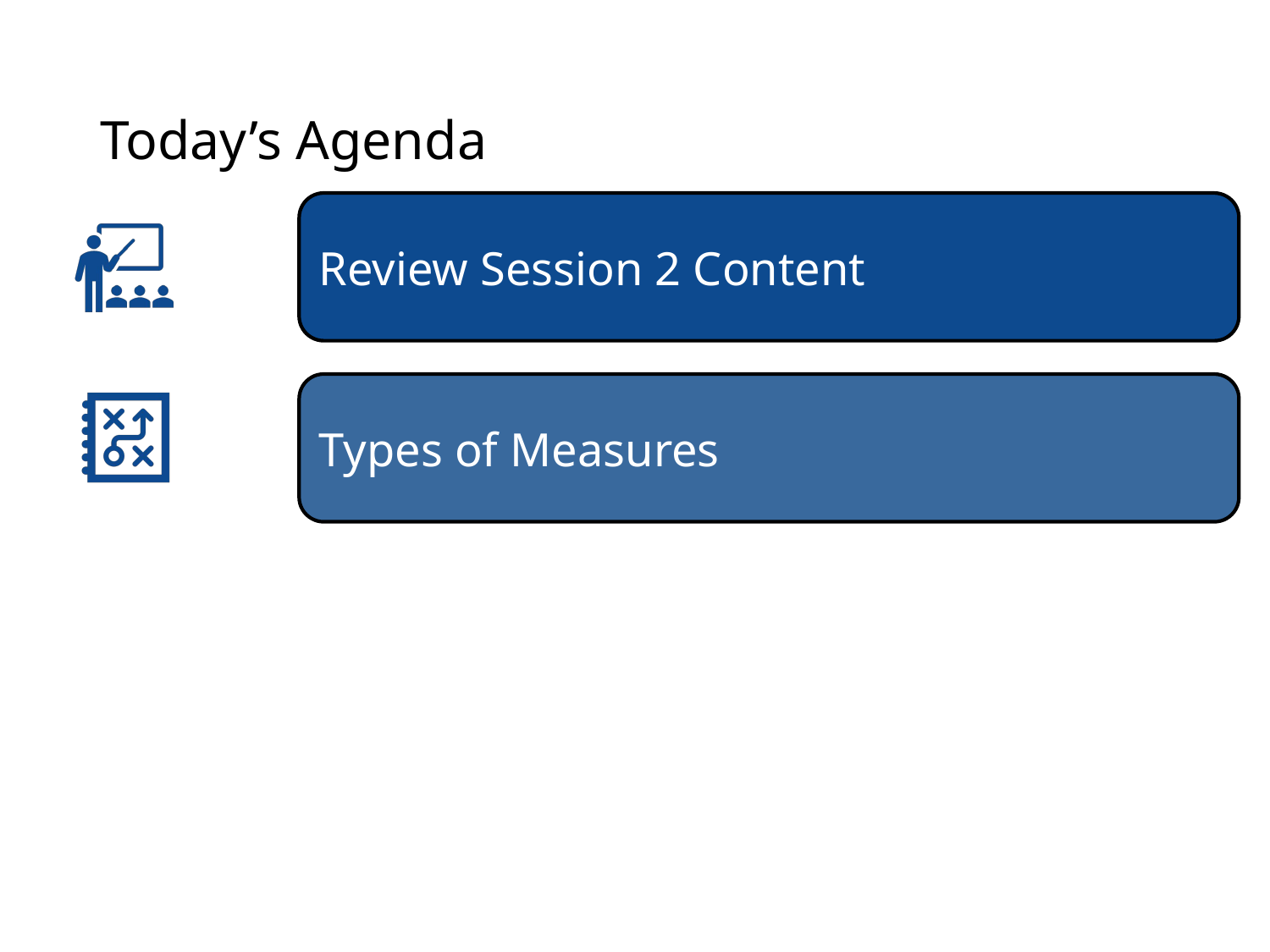

# Today’s Agenda
Review Session 2 Content
Types of Measures

## Slide 10
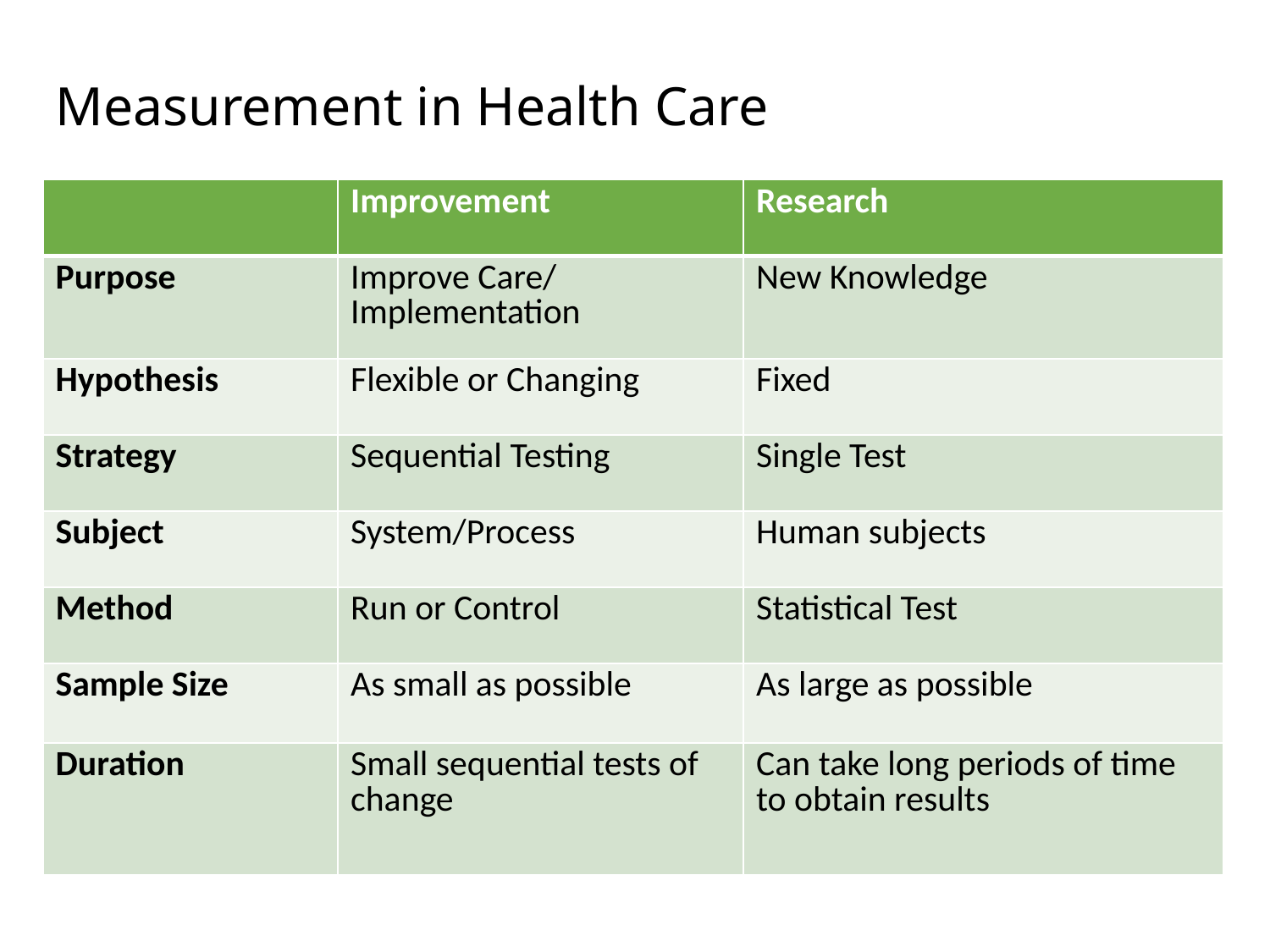

# Measurement in Health Care
| | Improvement | Research |
| --- | --- | --- |
| Purpose | Improve Care/ Implementation | New Knowledge |
| Hypothesis | Flexible or Changing | Fixed |
| Strategy | Sequential Testing | Single Test |
| Subject | System/Process | Human subjects |
| Method | Run or Control | Statistical Test |
| Sample Size | As small as possible | As large as possible |
| Duration | Small sequential tests of change | Can take long periods of time to obtain results |

## Slide 11
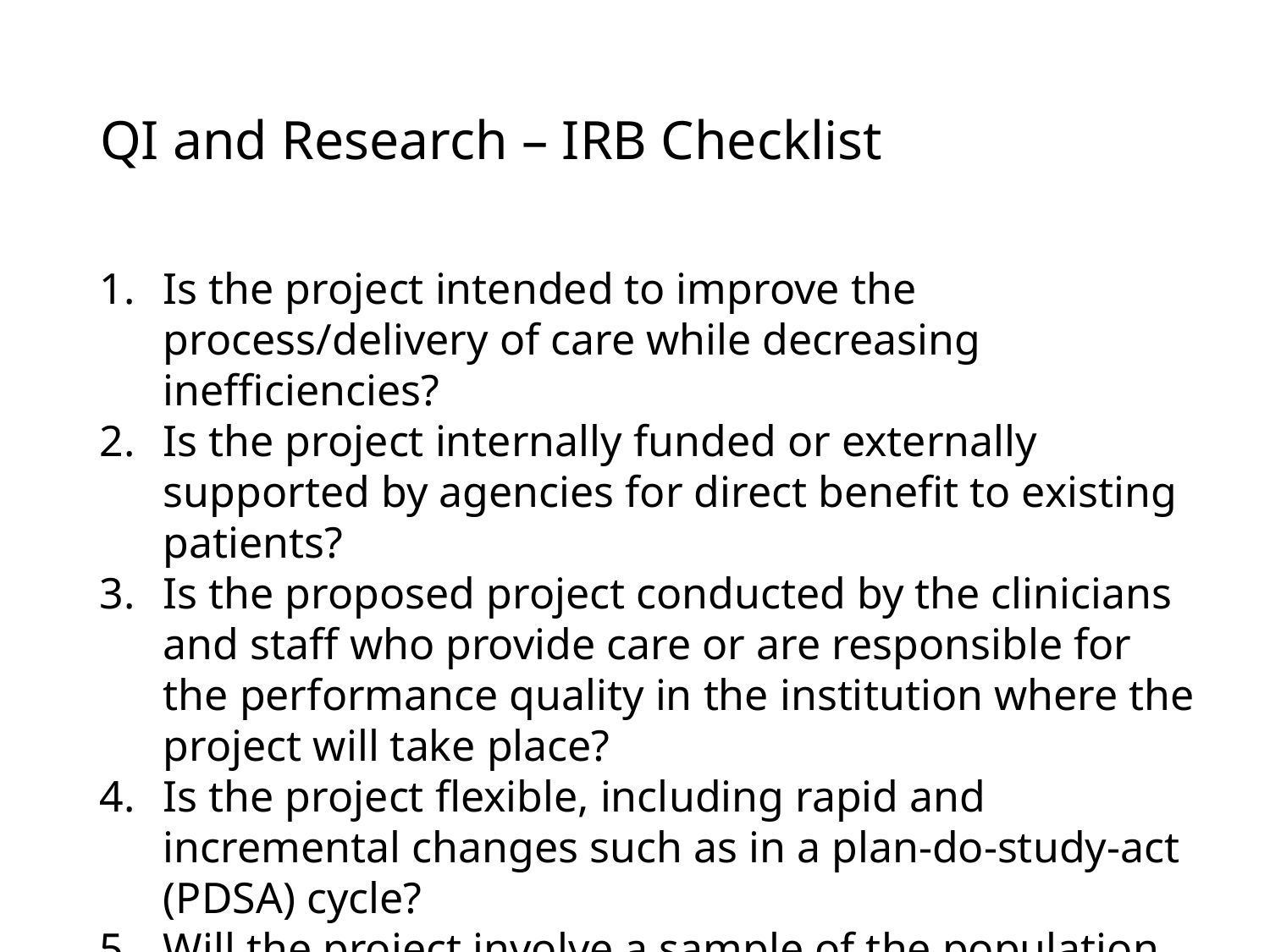

# QI and Research – IRB Checklist
Is the project intended to improve the process/delivery of care while decreasing inefficiencies?
Is the project internally funded or externally supported by agencies for direct benefit to existing patients?
Is the proposed project conducted by the clinicians and staff who provide care or are responsible for the performance quality in the institution where the project will take place?
Is the project flexible, including rapid and incremental changes such as in a plan-do-study-act (PDSA) cycle?
Will the project involve a sample of the population ordinarily seen at the institution?

## Slide 12
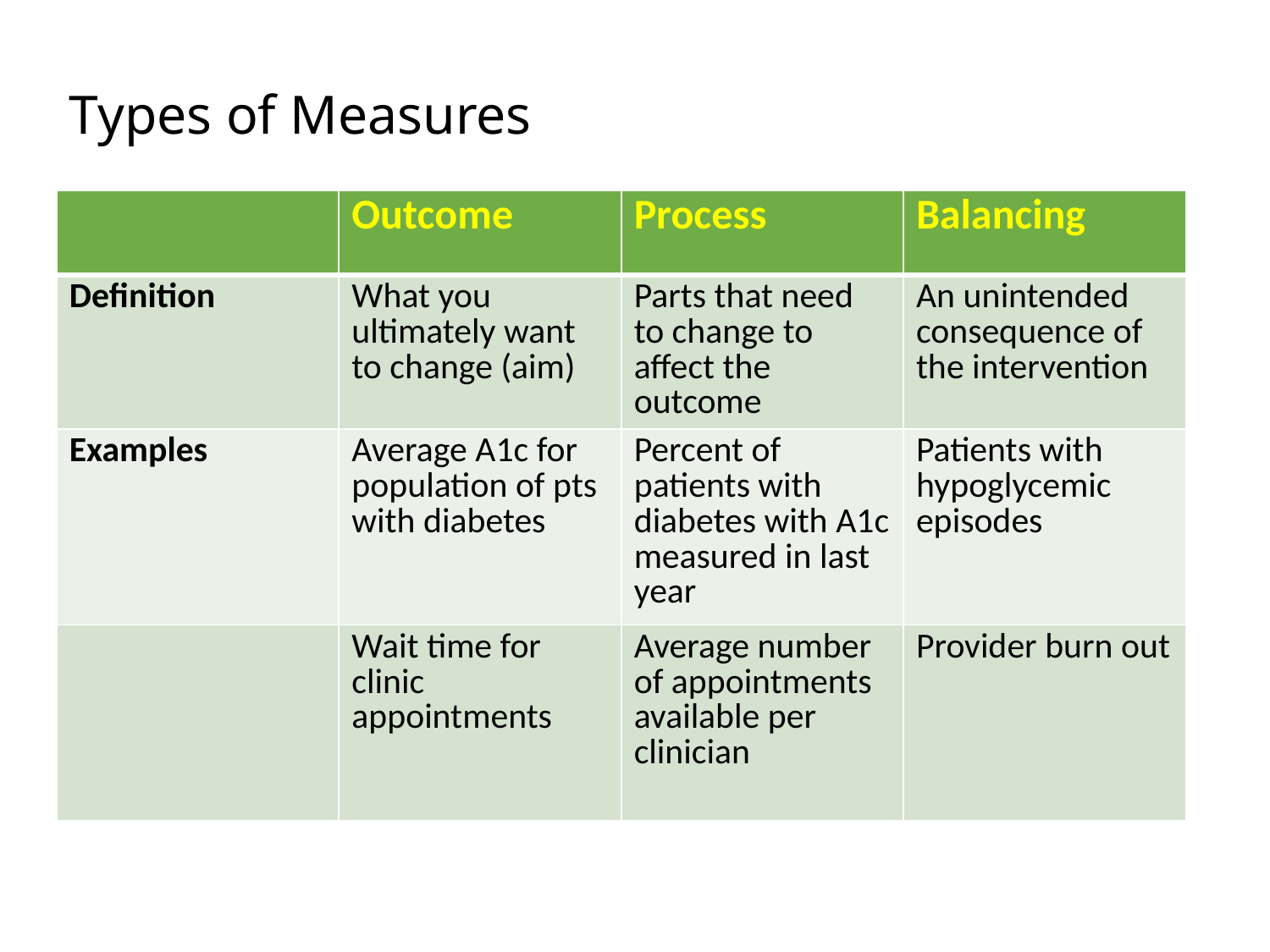

# Types of Measures
| | Outcome | Process | Balancing |
| --- | --- | --- | --- |
| Definition | What you ultimately want to change (aim) | Parts that need to change to affect the outcome | An unintended consequence of the intervention |
| Examples | Average A1c for population of pts with diabetes | Percent of patients with diabetes with A1c measured in last year | Patients with hypoglycemic episodes |
| | Wait time for clinic appointments | Average number of appointments available per clinician | Provider burn out |

## Slide 13
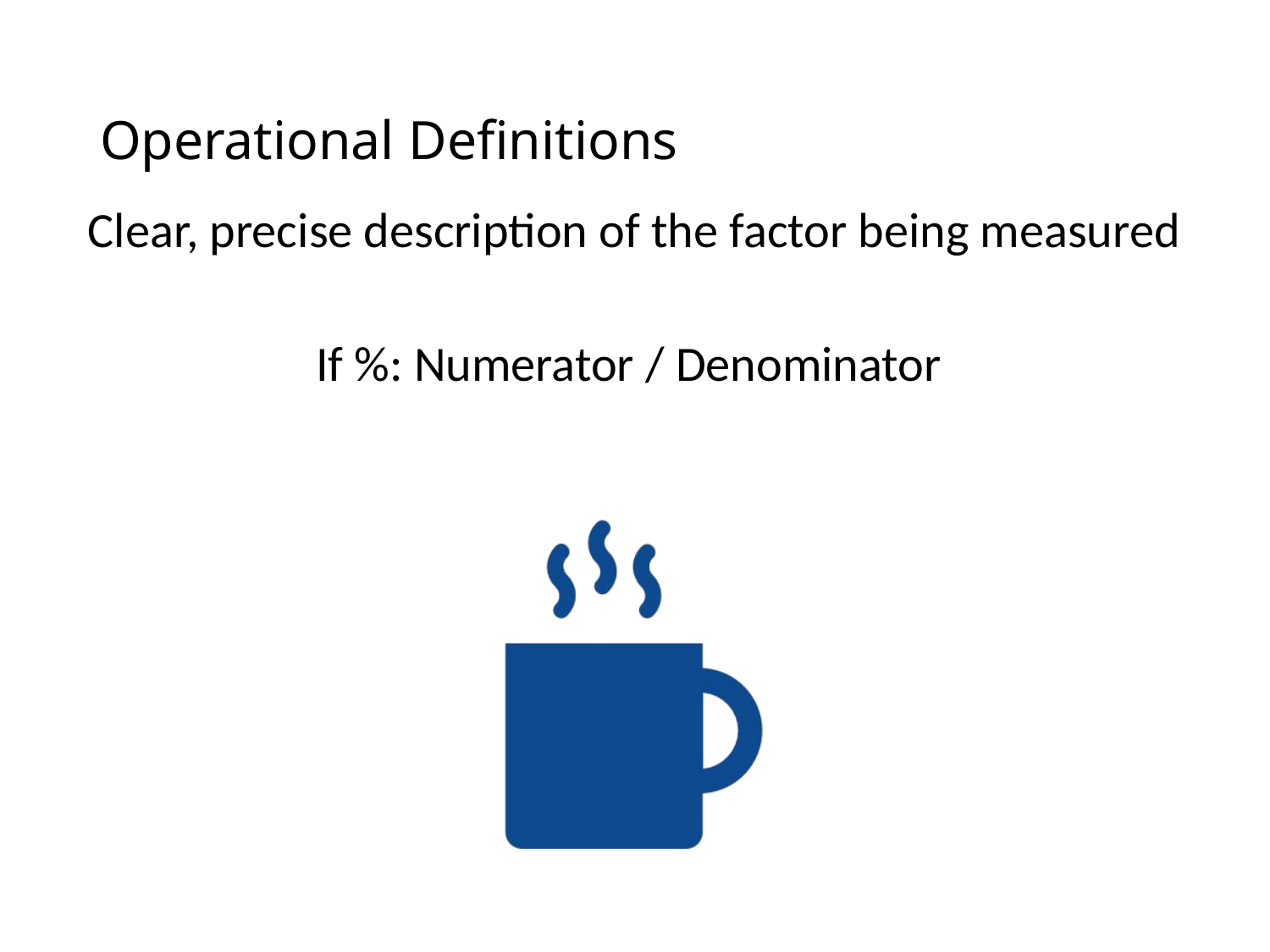

# Operational Definitions
Clear, precise description of the factor being measured
If %: Numerator / Denominator

## Slide 14
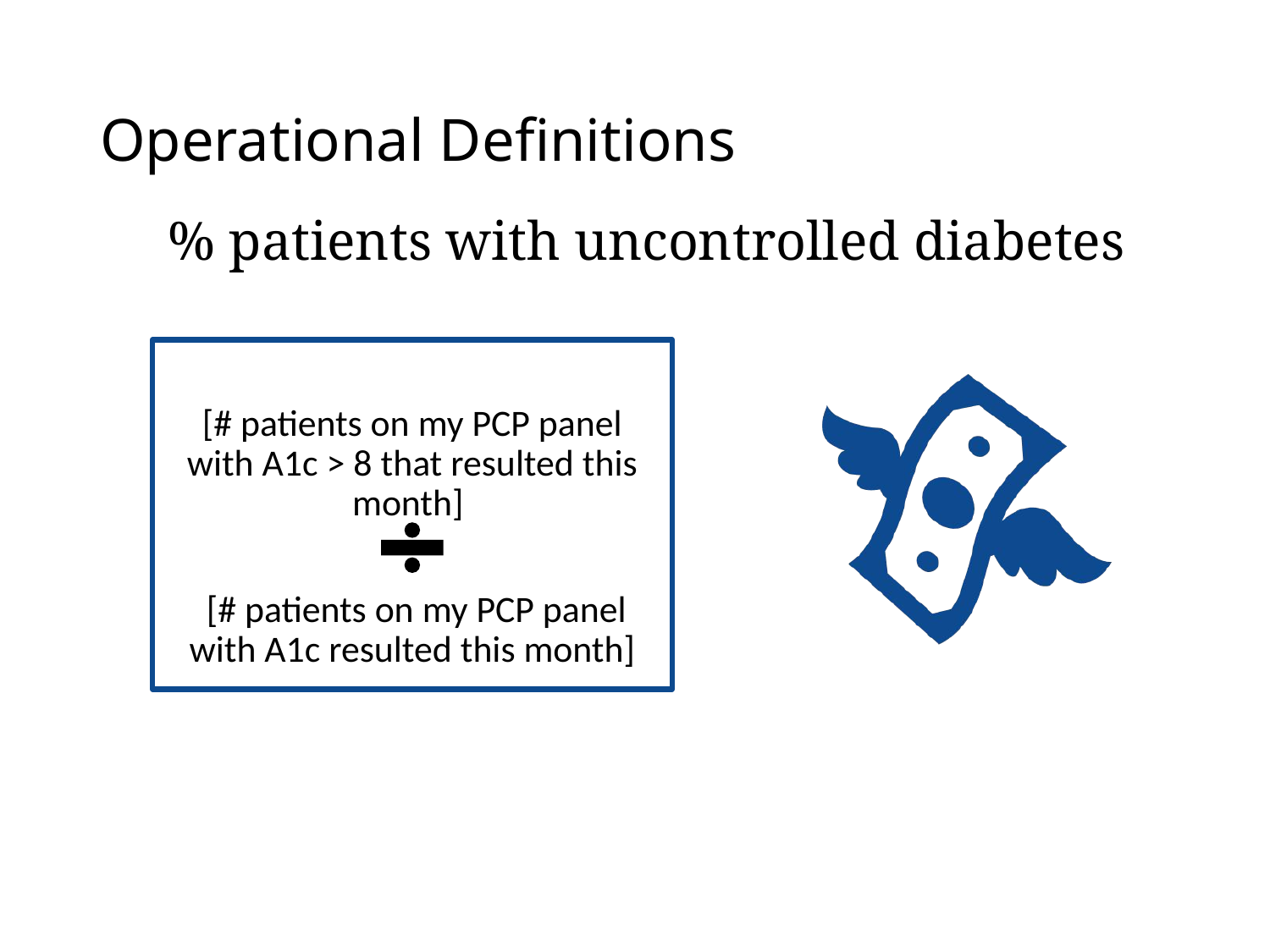

# Operational Definitions
% patients with uncontrolled diabetes
[# patients on my PCP panel with A1c > 8 that resulted this month]
 [# patients on my PCP panel with A1c resulted this month]

## Slide 15
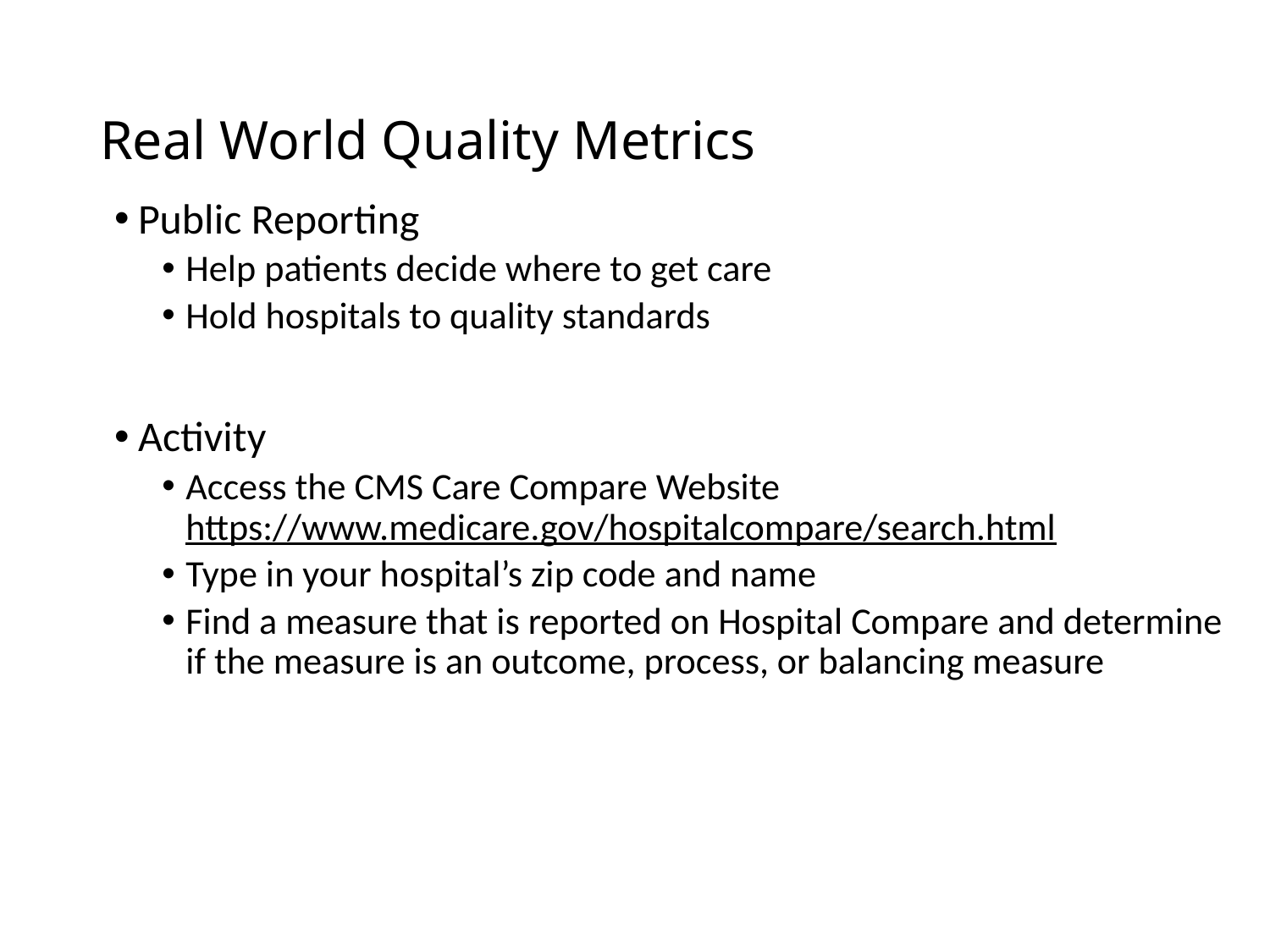

# Real World Quality Metrics
Public Reporting
Help patients decide where to get care
Hold hospitals to quality standards
Activity
Access the CMS Care Compare Website https://www.medicare.gov/hospitalcompare/search.html
Type in your hospital’s zip code and name
Find a measure that is reported on Hospital Compare and determine if the measure is an outcome, process, or balancing measure

## Slide 16
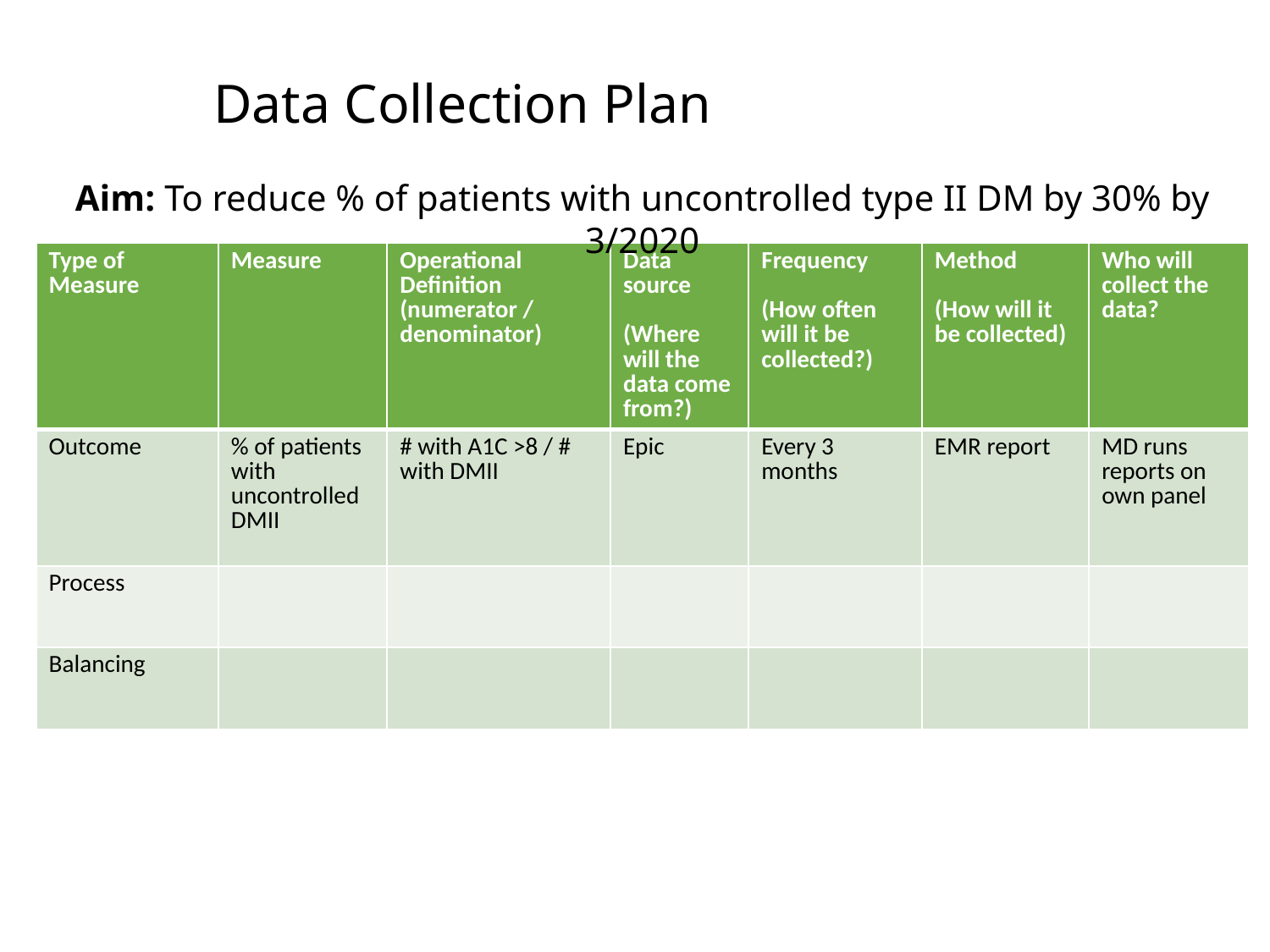

# Data Collection Plan
Aim: To reduce % of patients with uncontrolled type II DM by 30% by 3/2020
| Type of Measure | Measure | Operational Definition (numerator / denominator) | Data source (Where will the data come from?) | Frequency (How often will it be collected?) | Method (How will it be collected) | Who will collect the data? |
| --- | --- | --- | --- | --- | --- | --- |
| Outcome | % of patients with uncontrolled DMII | # with A1C >8 / # with DMII | Epic | Every 3 months | EMR report | MD runs reports on own panel |
| Process | | | | | | |
| Balancing | | | | | | |

## Slide 17
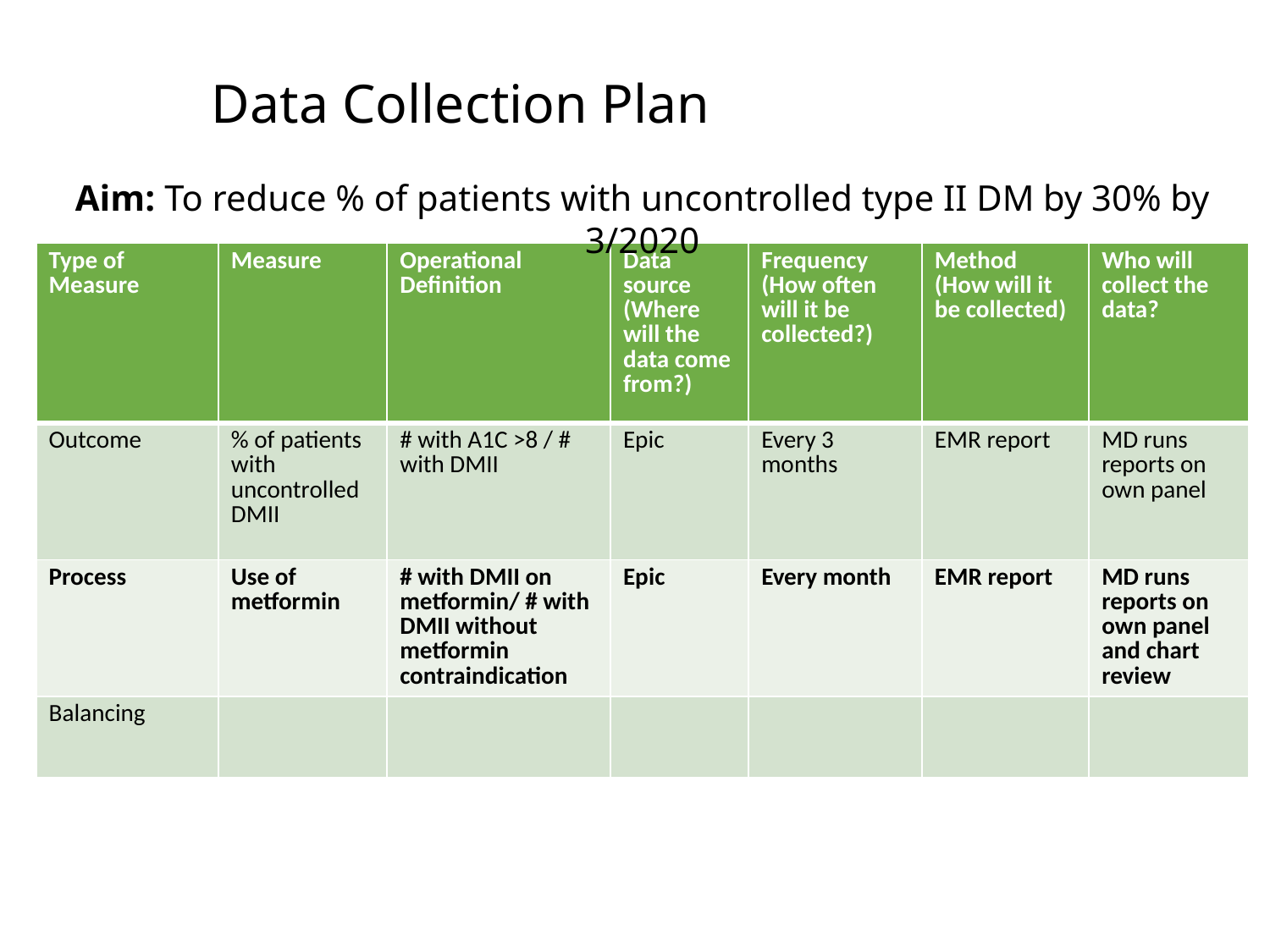

# Data Collection Plan
Aim: To reduce % of patients with uncontrolled type II DM by 30% by 3/2020
| Type of Measure | Measure | Operational Definition | Data source (Where will the data come from?) | Frequency (How often will it be collected?) | Method (How will it be collected) | Who will collect the data? |
| --- | --- | --- | --- | --- | --- | --- |
| Outcome | % of patients with uncontrolled DMII | # with A1C >8 / # with DMII | Epic | Every 3 months | EMR report | MD runs reports on own panel |
| Process | Use of metformin | # with DMII on metformin/ # with DMII without metformin contraindication | Epic | Every month | EMR report | MD runs reports on own panel and chart review |
| Balancing | | | | | | |

## Slide 18
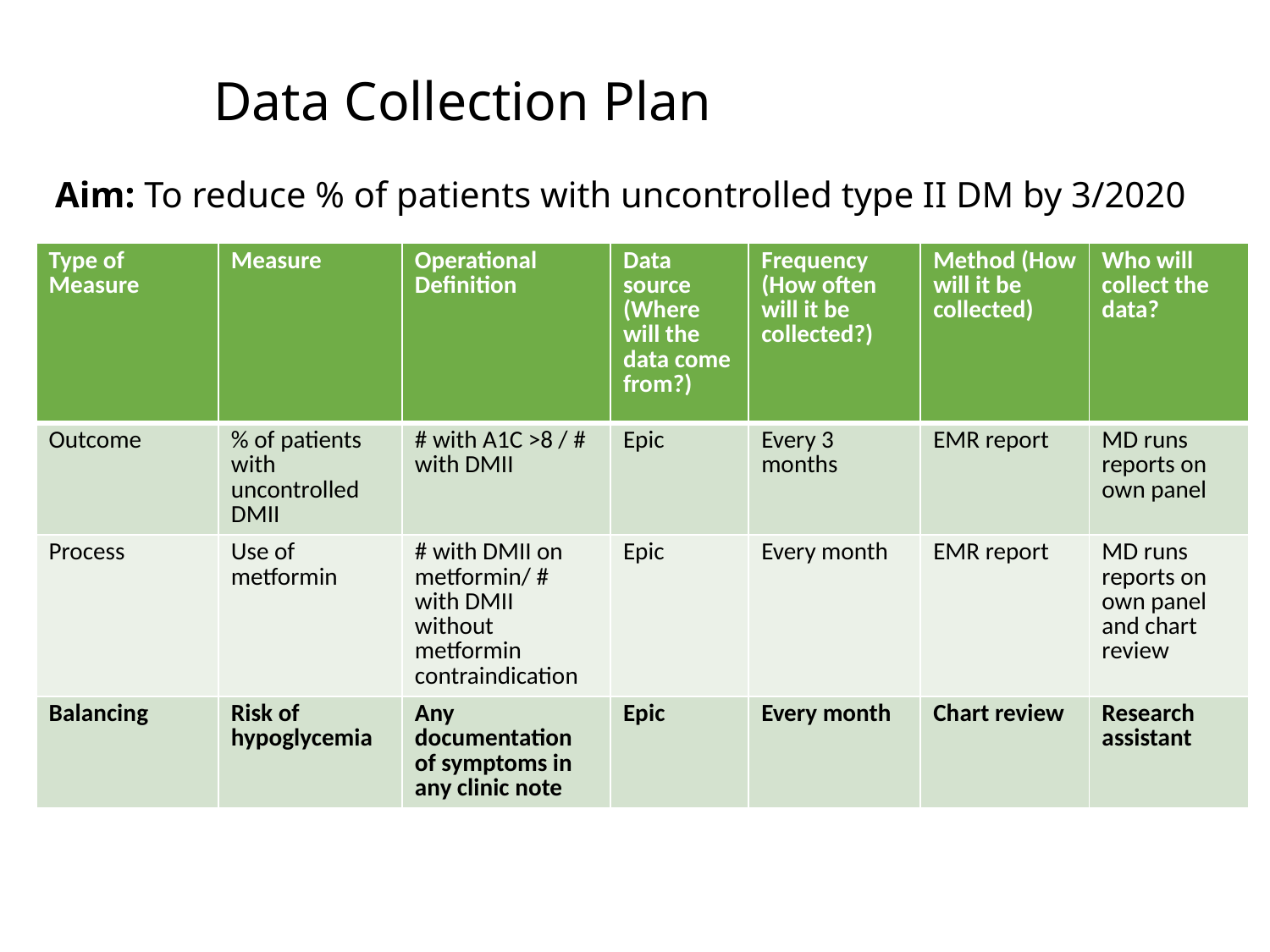

# Data Collection Plan
Aim: To reduce % of patients with uncontrolled type II DM by 3/2020
| Type of Measure | Measure | Operational Definition | Data source (Where will the data come from?) | Frequency (How often will it be collected?) | Method (How will it be collected) | Who will collect the data? |
| --- | --- | --- | --- | --- | --- | --- |
| Outcome | % of patients with uncontrolled DMII | # with A1C >8 / # with DMII | Epic | Every 3 months | EMR report | MD runs reports on own panel |
| Process | Use of metformin | # with DMII on metformin/ # with DMII without metformin contraindication | Epic | Every month | EMR report | MD runs reports on own panel and chart review |
| Balancing | Risk of hypoglycemia | Any documentation of symptoms in any clinic note | Epic | Every month | Chart review | Research assistant |

## Slide 19
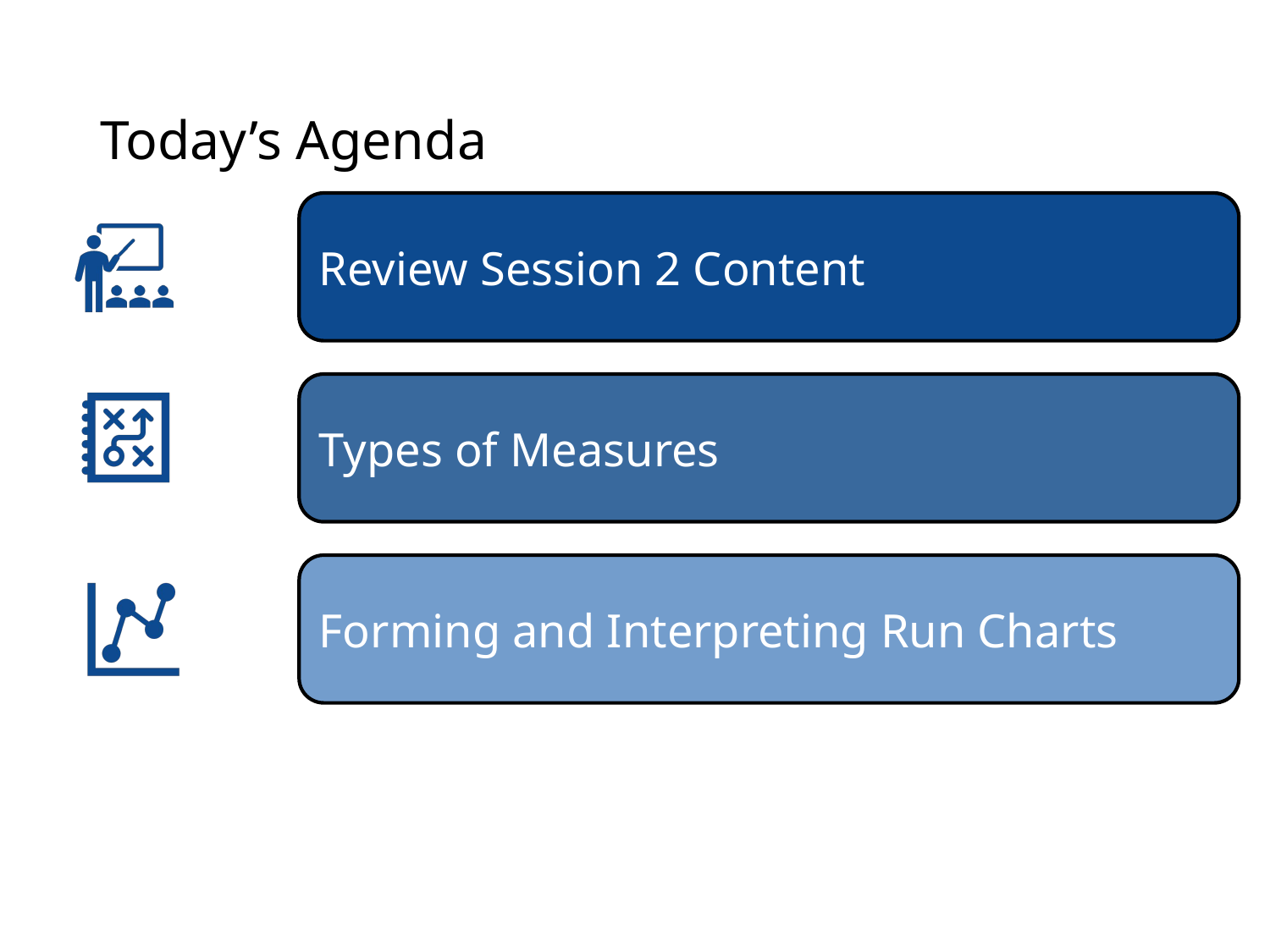

# Today’s Agenda
Review Session 2 Content
Types of Measures
Forming and Interpreting Run Charts

## Slide 20
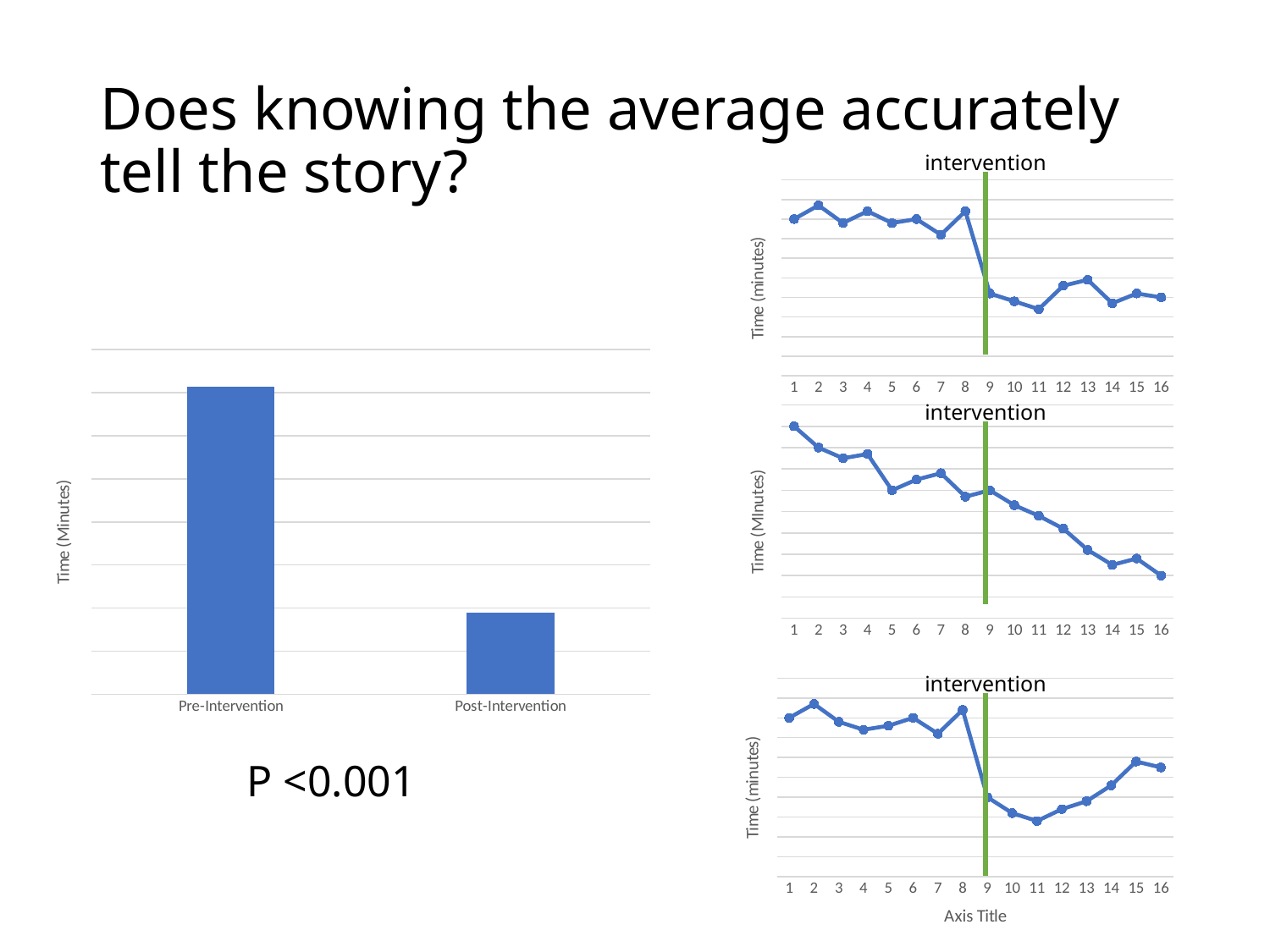

# Does knowing the average accurately tell the story?
intervention
### Chart
| Category | |
|---|---|
### Chart
| Category | |
|---|---|
| Pre-Intervention | 71.36363636363636 |
| Post-Intervention | 18.90909090909091 |intervention
### Chart
| Category | Time |
|---|---|intervention
### Chart
| Category | |
|---|---|P <0.001

## Slide 21
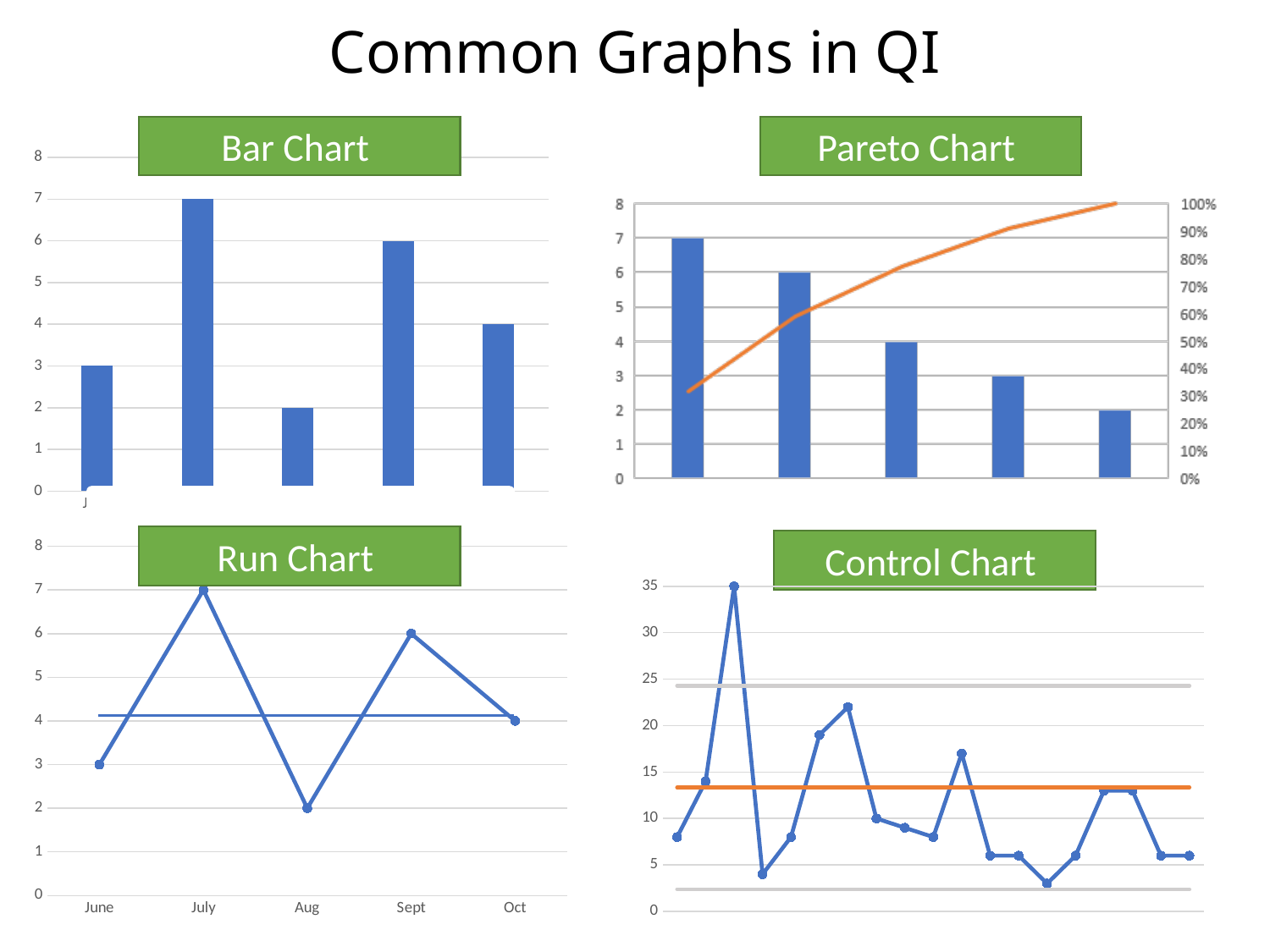

# Common Graphs in QI
Bar Chart
Pareto Chart
### Chart:
| Category | Count |
|---|---|
| June | 3.0 |
| July | 7.0 |
| Aug | 2.0 |
| Sept | 6.0 |
| Oct | 4.0 |
Run Chart
Control Chart
### Chart:
| Category | Count |
|---|---|
| June | 3.0 |
| July | 7.0 |
| Aug | 2.0 |
| Sept | 6.0 |
| Oct | 4.0 |
### Chart
| Category | Number of Monthly Tests | Central Line | Upper Control Limit | Lower Control Limit |
|---|---|---|---|---|
| Jan | 8.0 | 13.333333333333334 | 24.287784483436656 | 2.3788821832300115 |
| Feb | 14.0 | 13.333333333333334 | 24.287784483436656 | 2.3788821832300115 |
| Mar | 35.0 | 13.333333333333334 | 24.287784483436656 | 2.3788821832300115 |
| Apr | 4.0 | 13.333333333333334 | 24.287784483436656 | 2.3788821832300115 |
| May | 8.0 | 13.333333333333334 | 24.287784483436656 | 2.3788821832300115 |
| Jun | 19.0 | 13.333333333333334 | 24.287784483436656 | 2.3788821832300115 |
| Jul | 22.0 | 13.333333333333334 | 24.287784483436656 | 2.3788821832300115 |
| Aug | 10.0 | 13.333333333333334 | 24.287784483436656 | 2.3788821832300115 |
| Sep | 9.0 | 13.333333333333334 | 24.287784483436656 | 2.3788821832300115 |
| Oct | 8.0 | 13.333333333333334 | 24.287784483436656 | 2.3788821832300115 |
| Nov | 17.0 | 13.333333333333334 | 24.287784483436656 | 2.3788821832300115 |
| Dec | 6.0 | 13.333333333333334 | 24.287784483436656 | 2.3788821832300115 |
| Jan | 6.0 | 13.333333333333334 | 24.287784483436656 | 2.3788821832300115 |
| Feb | 3.0 | 13.333333333333334 | 24.287784483436656 | 2.3788821832300115 |
| Mar | 6.0 | 13.333333333333334 | 24.287784483436656 | 2.3788821832300115 |
| April | 13.0 | 13.333333333333334 | 24.287784483436656 | 2.3788821832300115 |
| May | 13.0 | 13.333333333333334 | 24.287784483436656 | 2.3788821832300115 |
| Jun | 6.0 | 13.333333333333334 | 24.287784483436656 | 2.3788821832300115 |
| Jul | 6.0 | 13.333333333333334 | 24.287784483436656 | 2.3788821832300115 |

## Slide 22
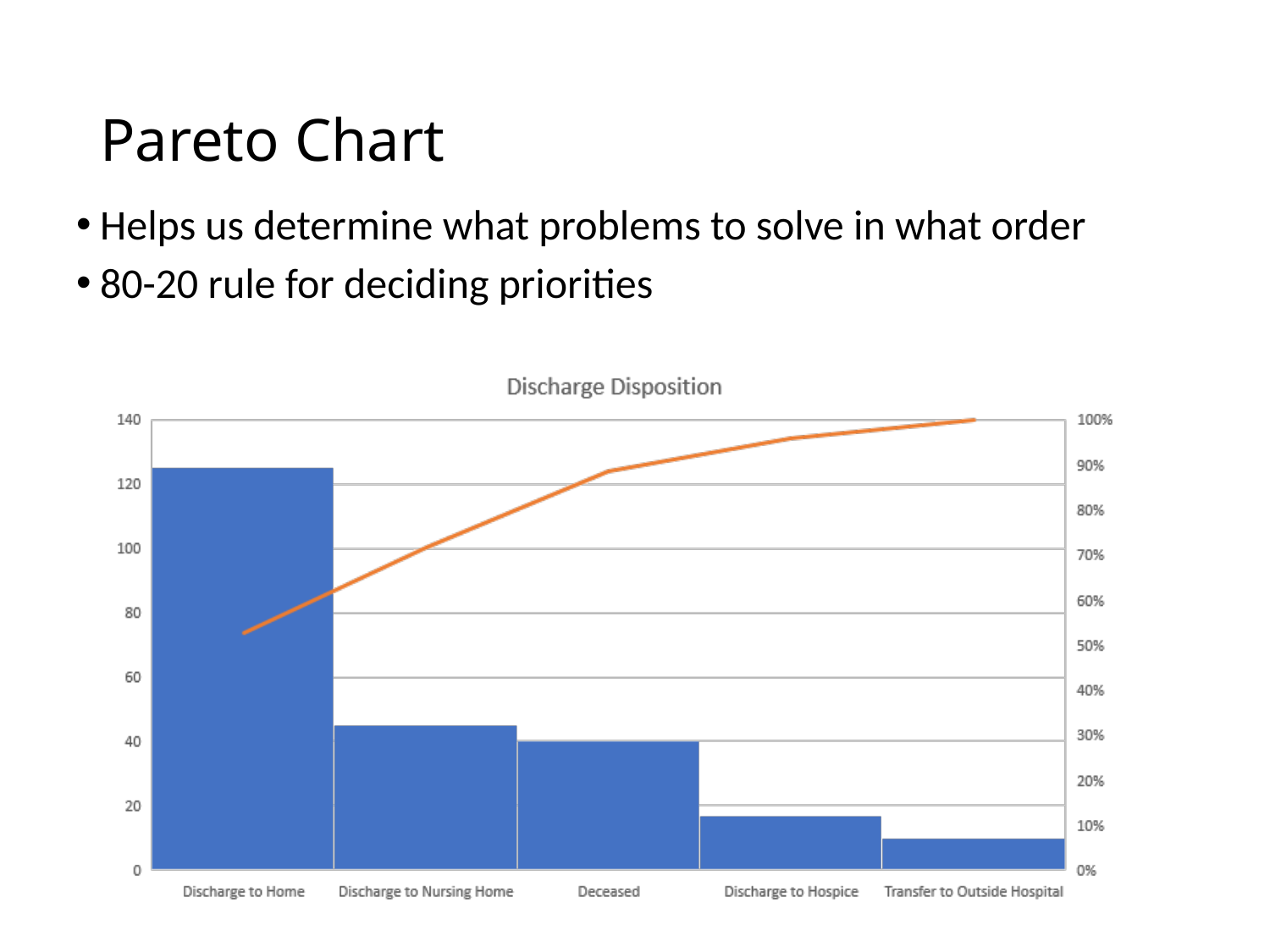

# Pareto Chart
Helps us determine what problems to solve in what order
80-20 rule for deciding priorities

## Slide 23
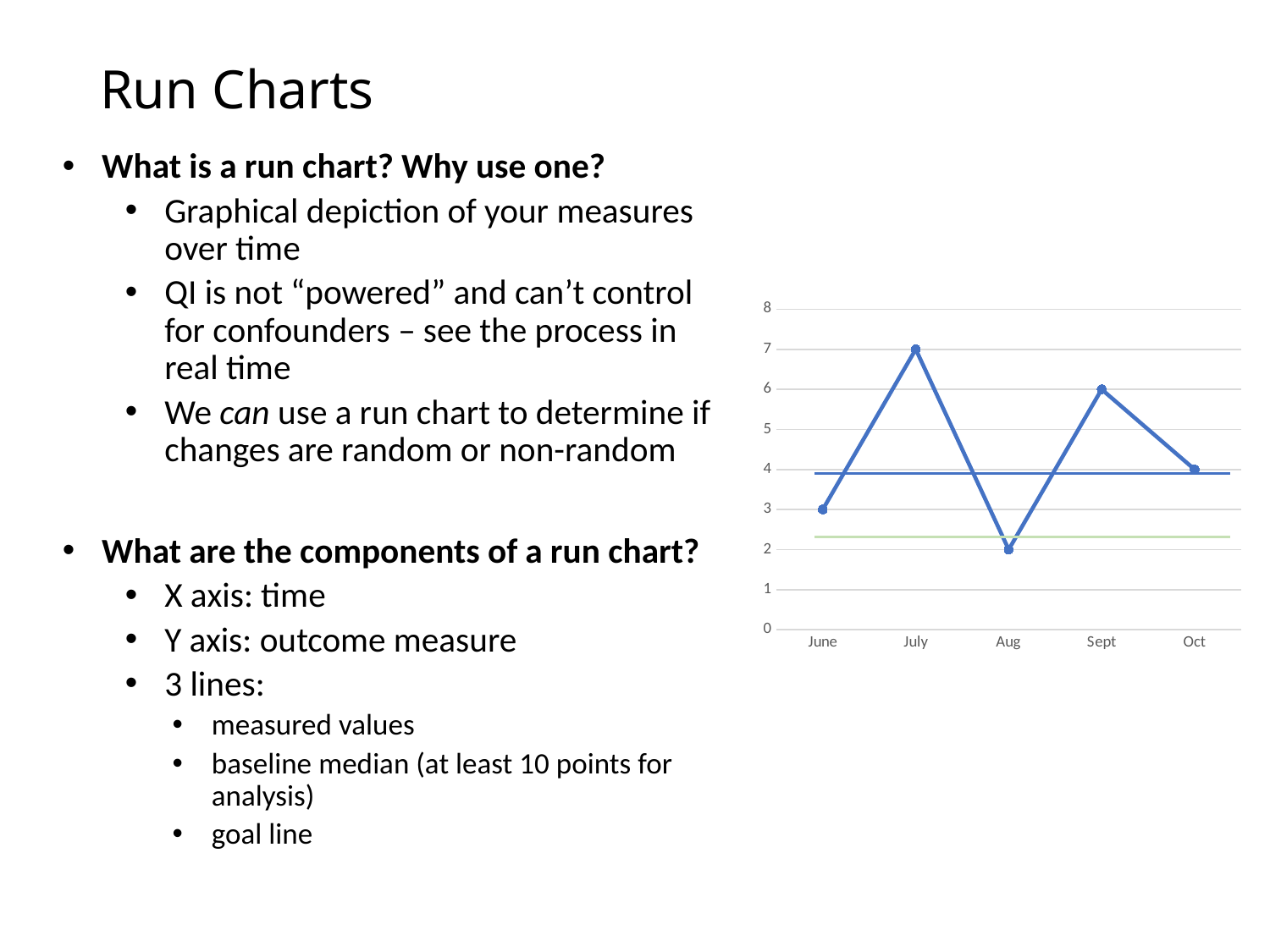

# Run Charts
What is a run chart? Why use one?
Graphical depiction of your measures over time
QI is not “powered” and can’t control for confounders – see the process in real time
We can use a run chart to determine if changes are random or non-random
What are the components of a run chart?
X axis: time
Y axis: outcome measure
3 lines:
measured values
baseline median (at least 10 points for analysis)
goal line
### Chart:
| Category | Count |
|---|---|
| June | 3.0 |
| July | 7.0 |
| Aug | 2.0 |
| Sept | 6.0 |
| Oct | 4.0 |

## Slide 24
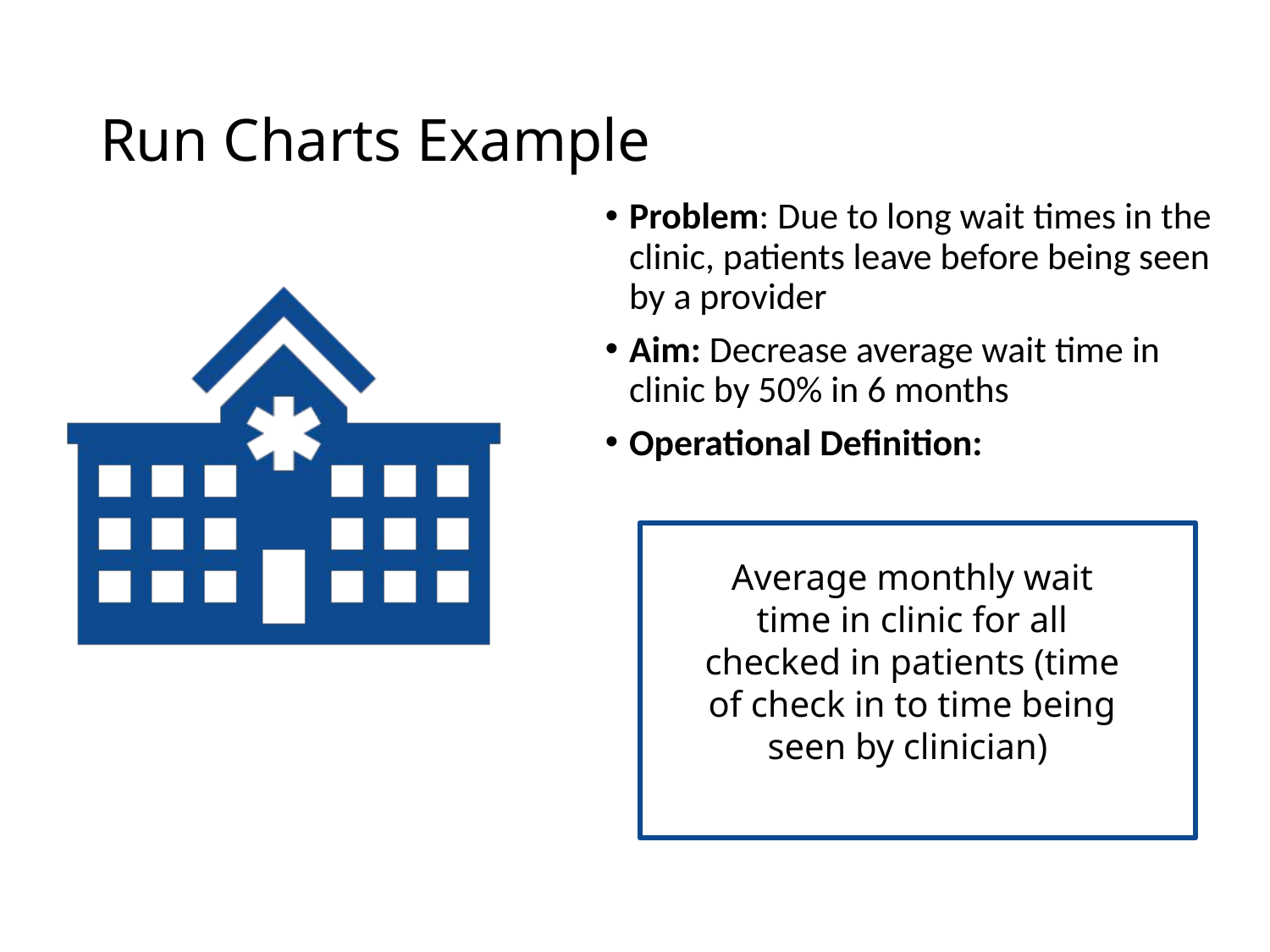

# Run Charts Example
Problem: Due to long wait times in the clinic, patients leave before being seen by a provider
Aim: Decrease average wait time in clinic by 50% in 6 months
Operational Definition:
Average monthly wait time in clinic for all checked in patients (time of check in to time being seen by clinician)

## Slide 25
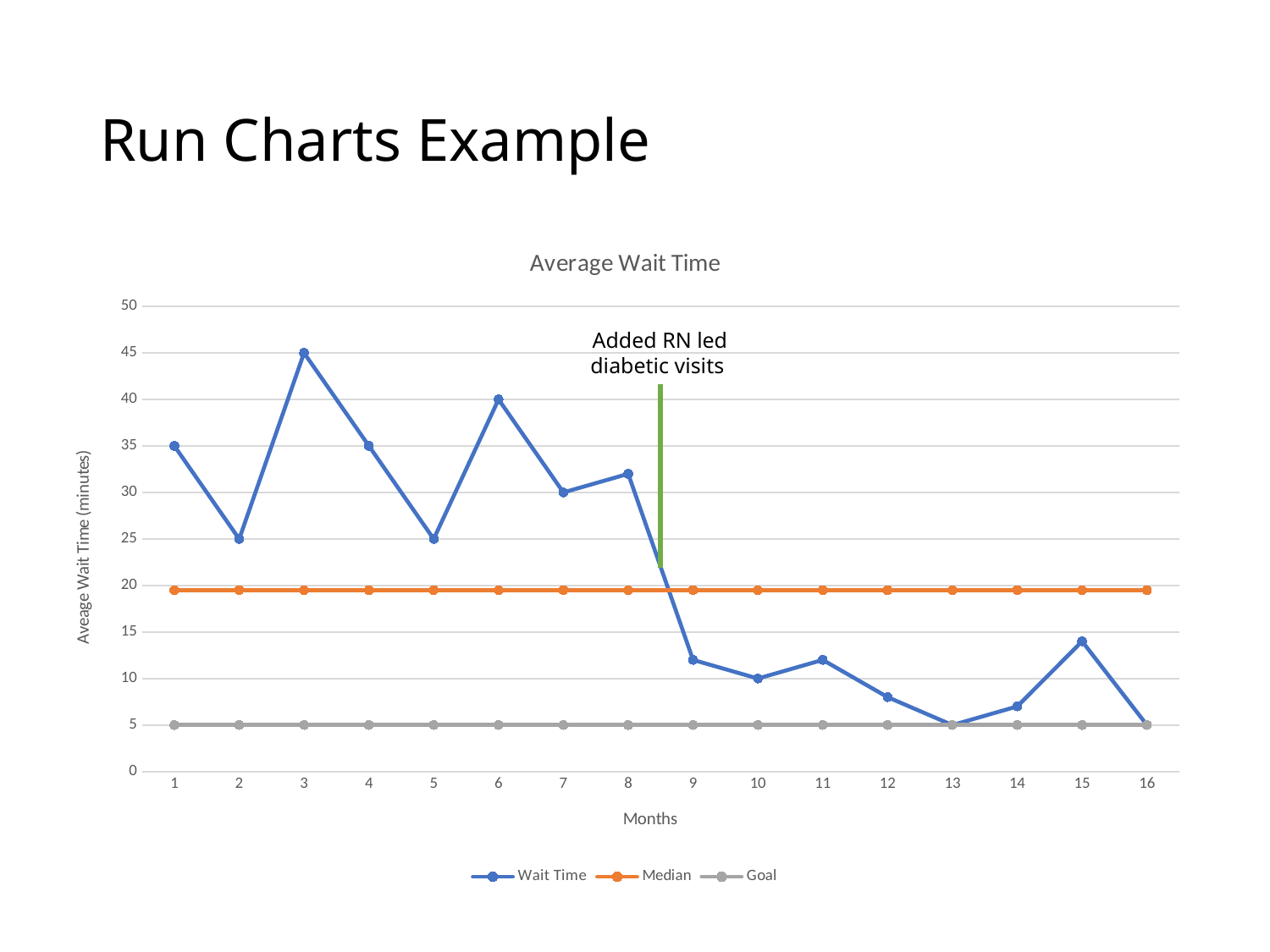

# Run Charts Example
### Chart: Average Wait Time
| Category | Wait Time | Median | Goal |
|---|---|---|---|Added RN led diabetic visits

## Slide 26
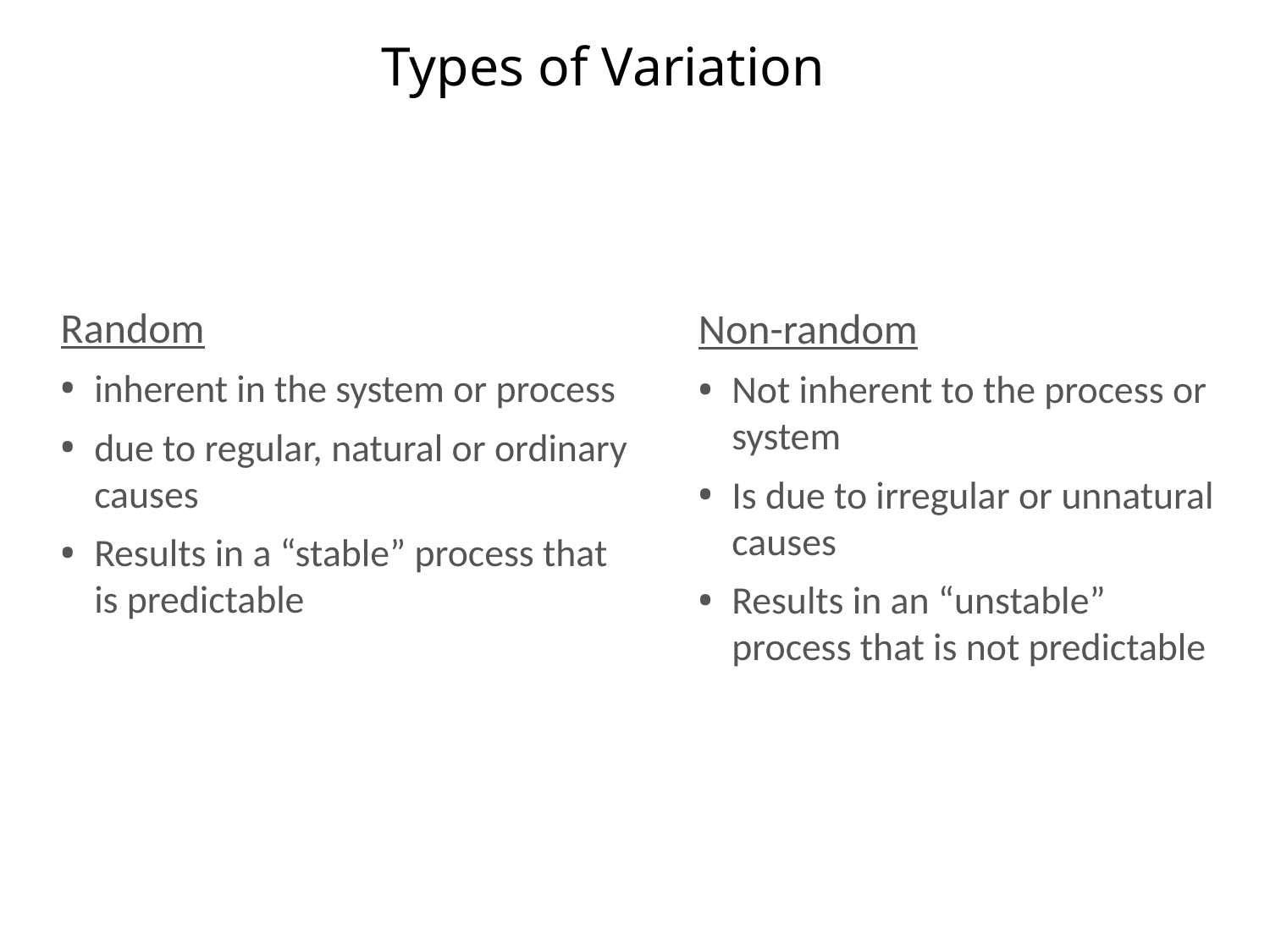

# Types of Variation
Random
inherent in the system or process
due to regular, natural or ordinary causes
Results in a “stable” process that is predictable
Non-random
Not inherent to the process or system
Is due to irregular or unnatural causes
Results in an “unstable” process that is not predictable

## Slide 27
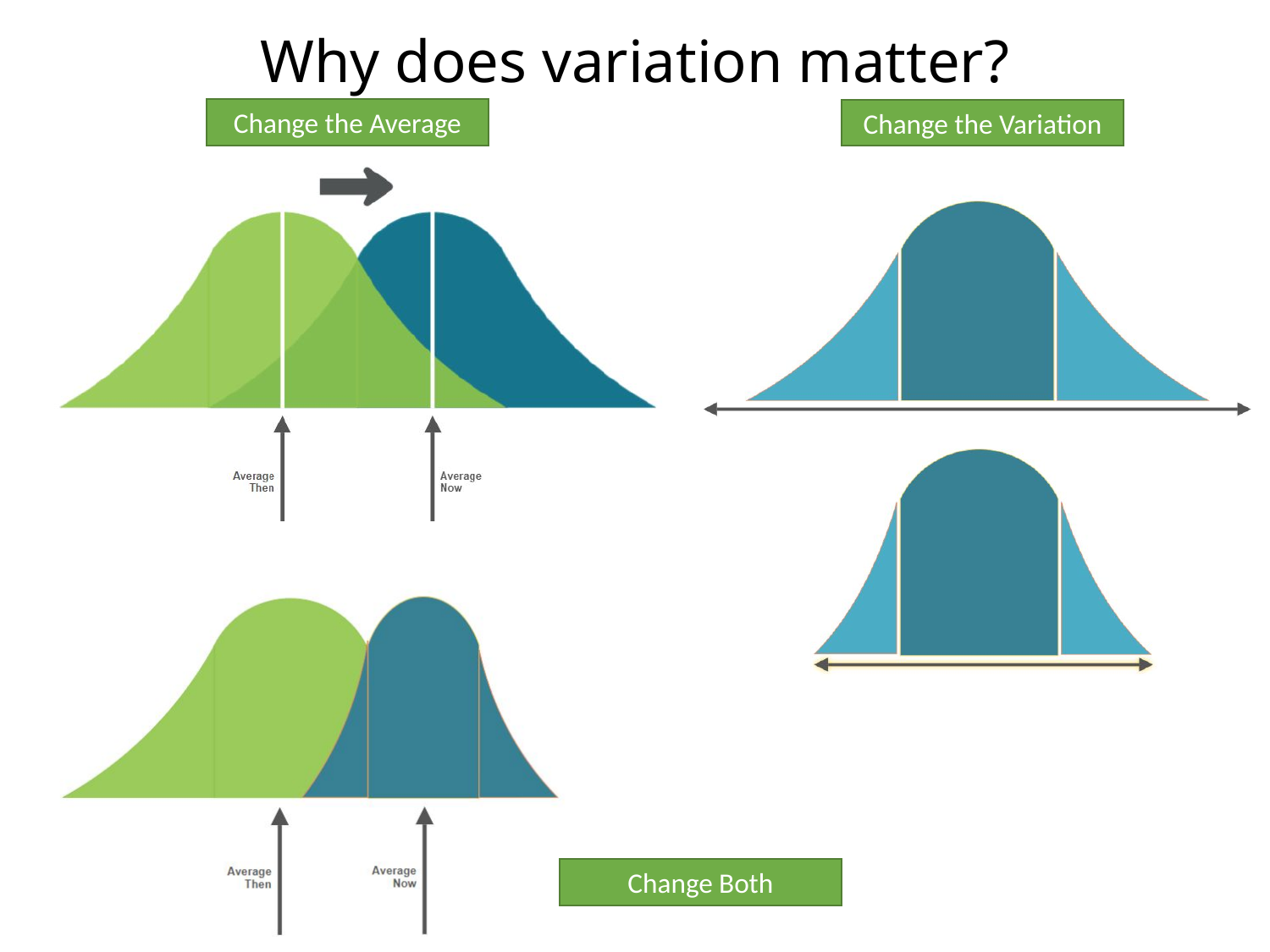

# Why does variation matter?
Change the Average
Change the Variation
Change Both

## Slide 28
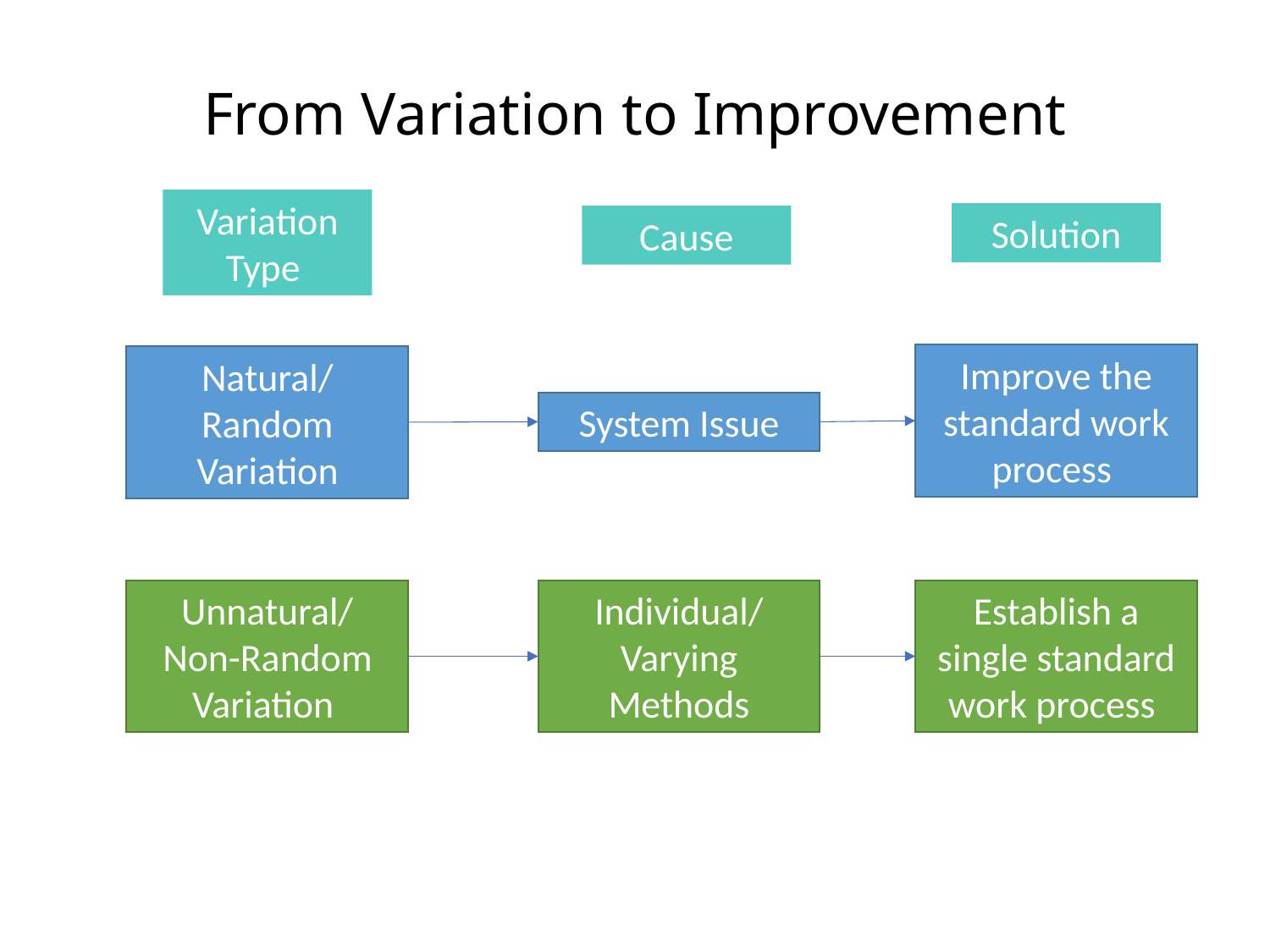

# From Variation to Improvement
Variation Type
Solution
Cause
Improve the standard work process
Natural/ Random Variation
System Issue
Unnatural/ Non-Random Variation
Individual/
Varying Methods
Establish a single standard work process

## Slide 29
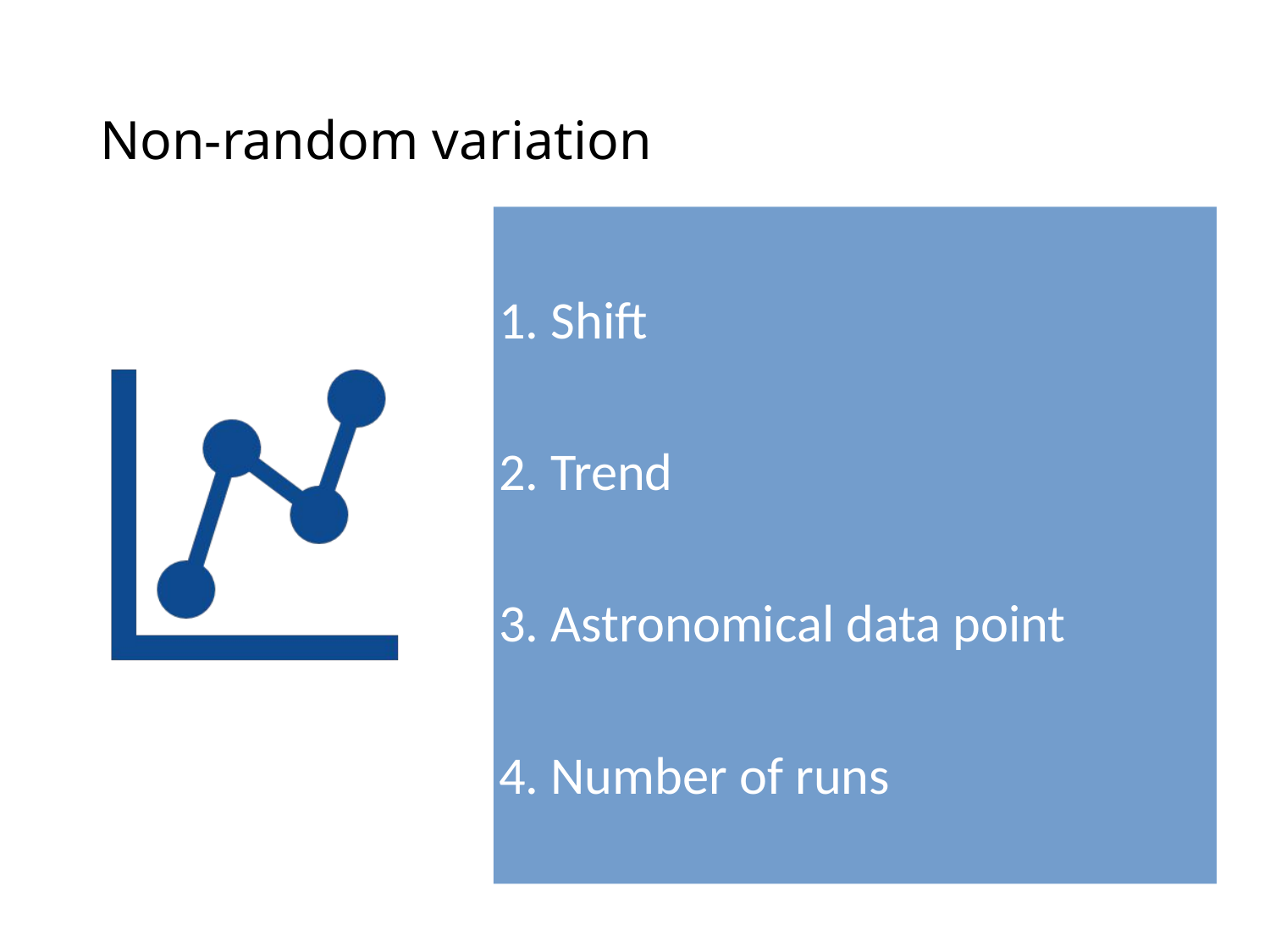

# Non-random variation
1. Shift
2. Trend
3. Astronomical data point
4. Number of runs

## Slide 30
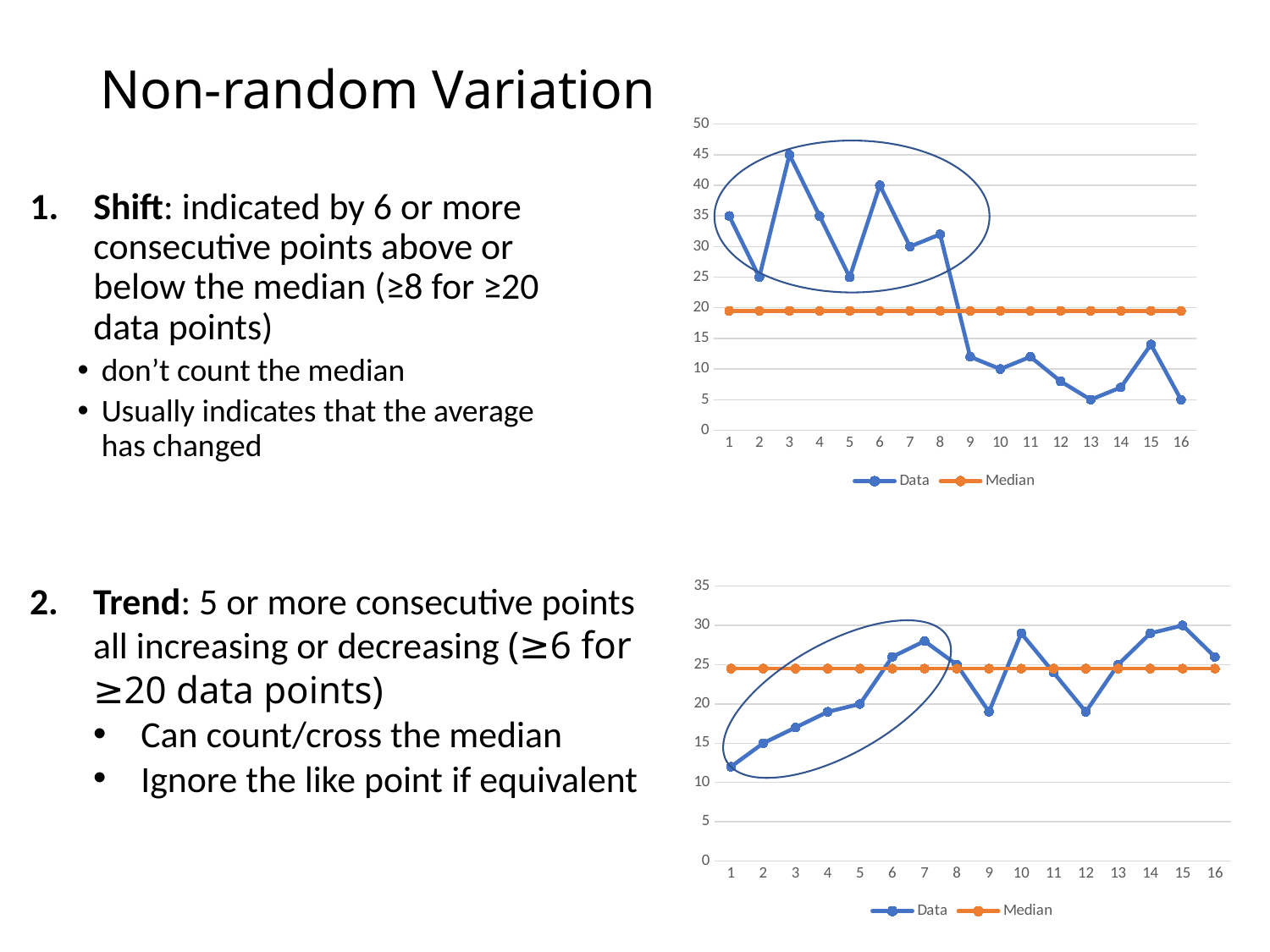

# Non-random Variation
### Chart
| Category | Data | Median |
|---|---|---|
Shift: indicated by 6 or more consecutive points above or below the median (≥8 for ≥20 data points)
don’t count the median
Usually indicates that the average has changed
Trend: 5 or more consecutive points all increasing or decreasing (≥6 for ≥20 data points)
Can count/cross the median
Ignore the like point if equivalent
### Chart
| Category | Data | Median |
|---|---|---|

## Slide 31
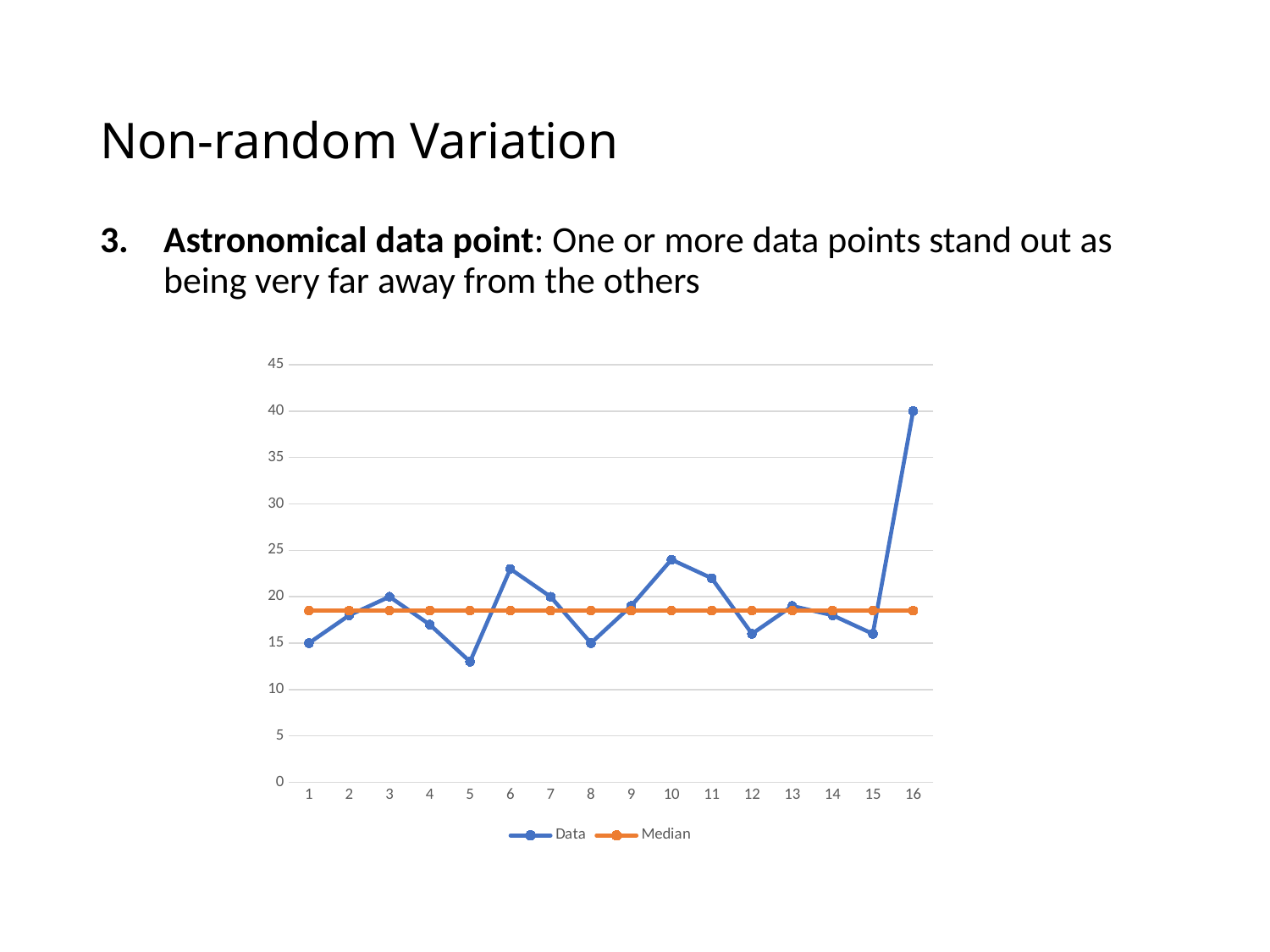

# Non-random Variation
Astronomical data point: One or more data points stand out as being very far away from the others
### Chart
| Category | Data | Median |
|---|---|---|

## Slide 32
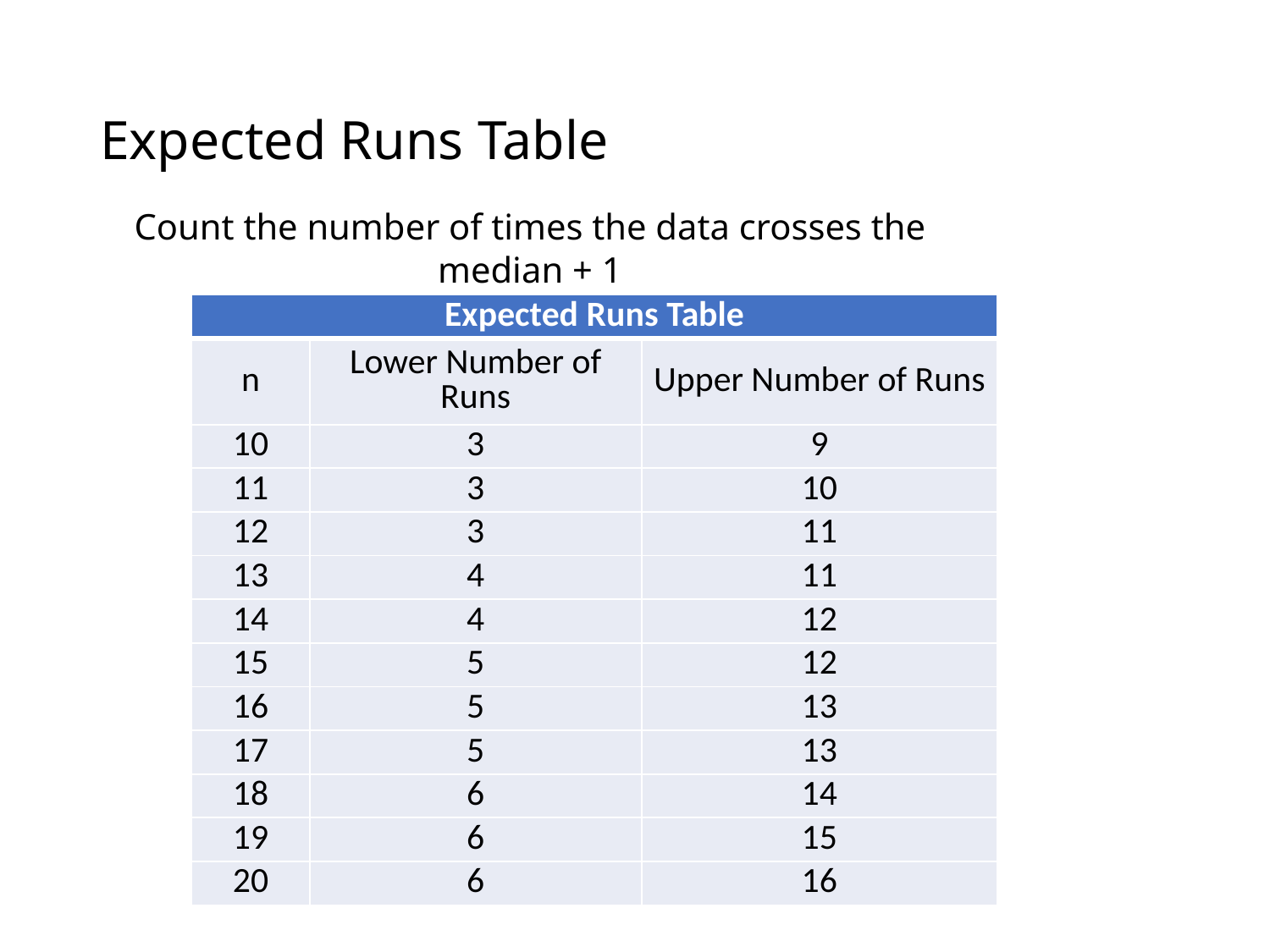

# Expected Runs Table
Count the number of times the data crosses the median + 1
| Expected Runs Table | | |
| --- | --- | --- |
| n | Lower Number of Runs | Upper Number of Runs |
| 10 | 3 | 9 |
| 11 | 3 | 10 |
| 12 | 3 | 11 |
| 13 | 4 | 11 |
| 14 | 4 | 12 |
| 15 | 5 | 12 |
| 16 | 5 | 13 |
| 17 | 5 | 13 |
| 18 | 6 | 14 |
| 19 | 6 | 15 |
| 20 | 6 | 16 |

## Slide 33
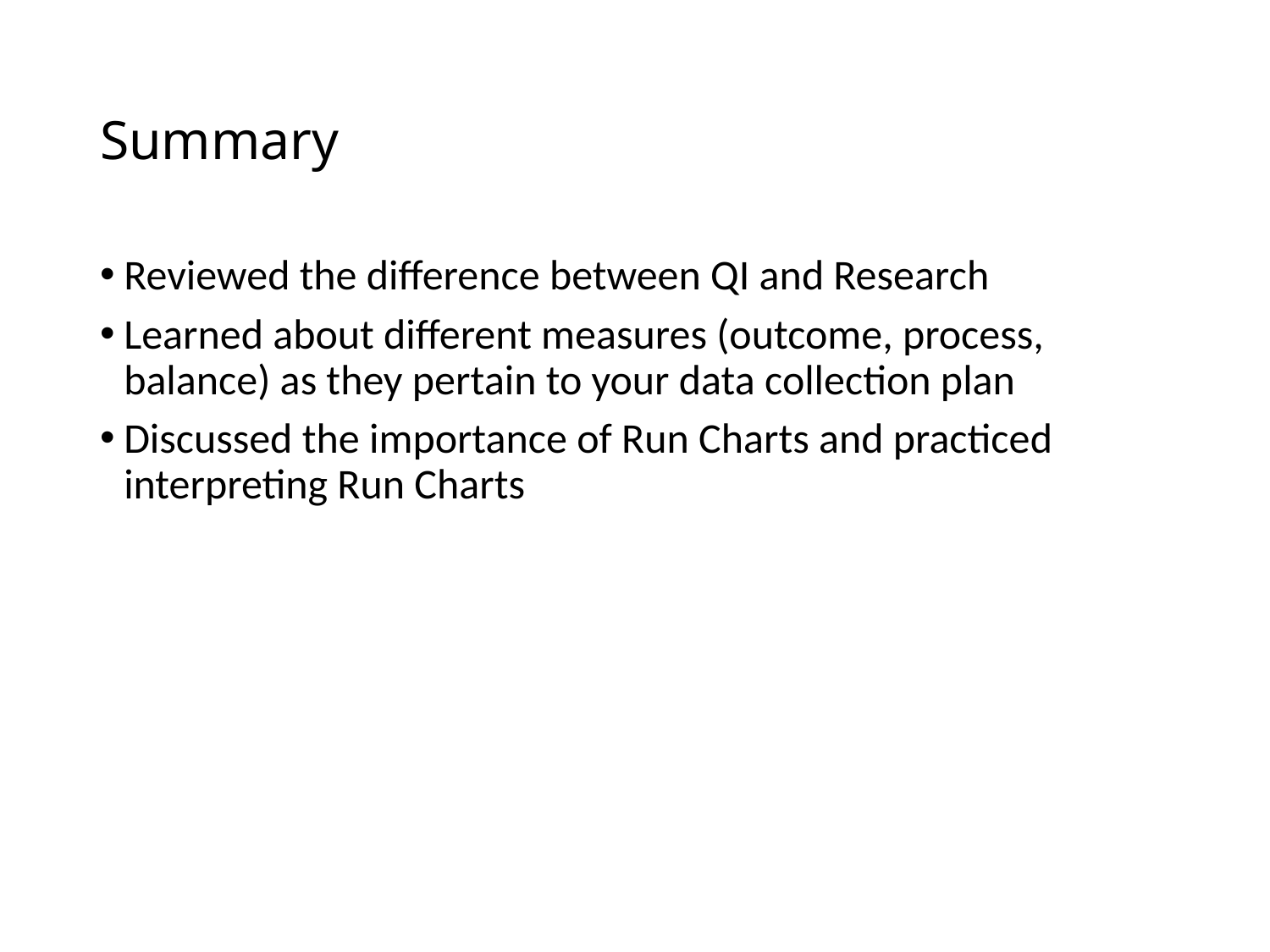

# Summary
Reviewed the difference between QI and Research
Learned about different measures (outcome, process, balance) as they pertain to your data collection plan
Discussed the importance of Run Charts and practiced interpreting Run Charts

## Slide 34
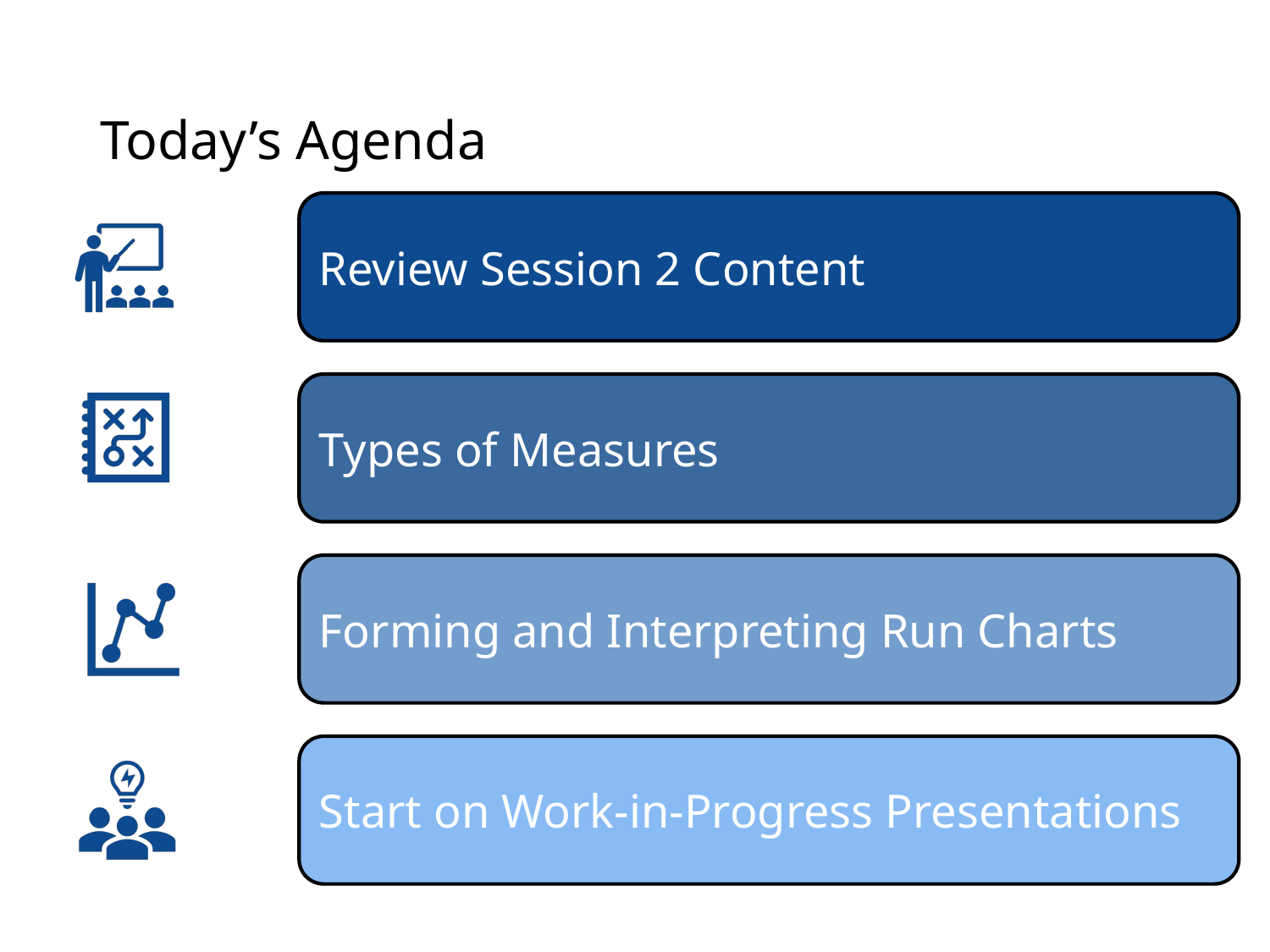

# Today’s Agenda
Review Session 2 Content
Types of Measures
Forming and Interpreting Run Charts
Start on Work-in-Progress Presentations
