# Supplementary material for: Building Resident Quality Improvement Knowledge and Engagement Through a Longitudinal, Mentored, and Experiential Learning-Based Quality Improvement Curriculum
Source: MedEdPORTAL. 2023 Apr 18;19:11310. doi: 10.15766/mep_2374-8265.11310 (PMC10110773; doi:10.15766/mep_2374-8265.11310)

## Slide 1
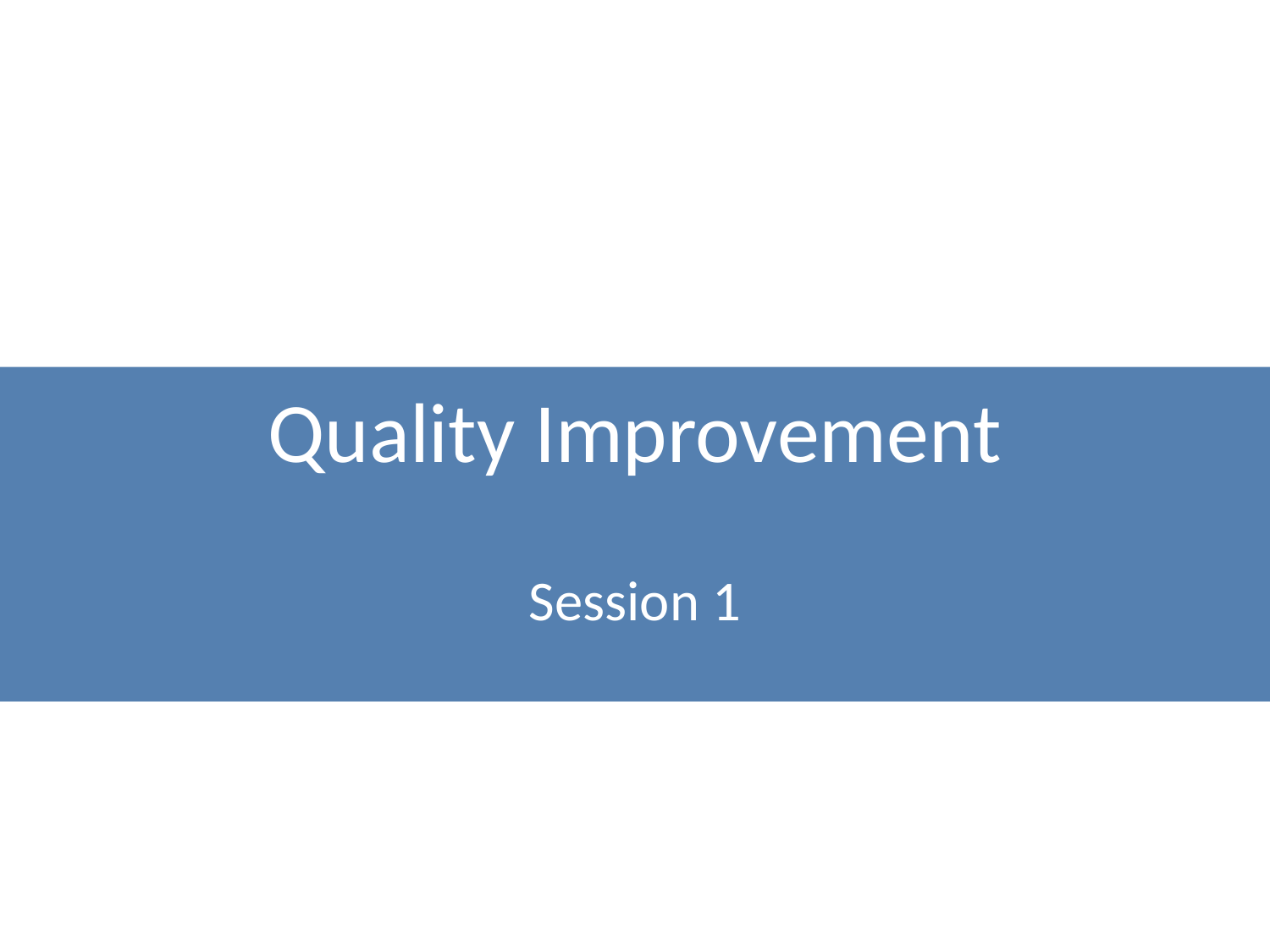

# Quality Improvement Session 1

## Slide 2
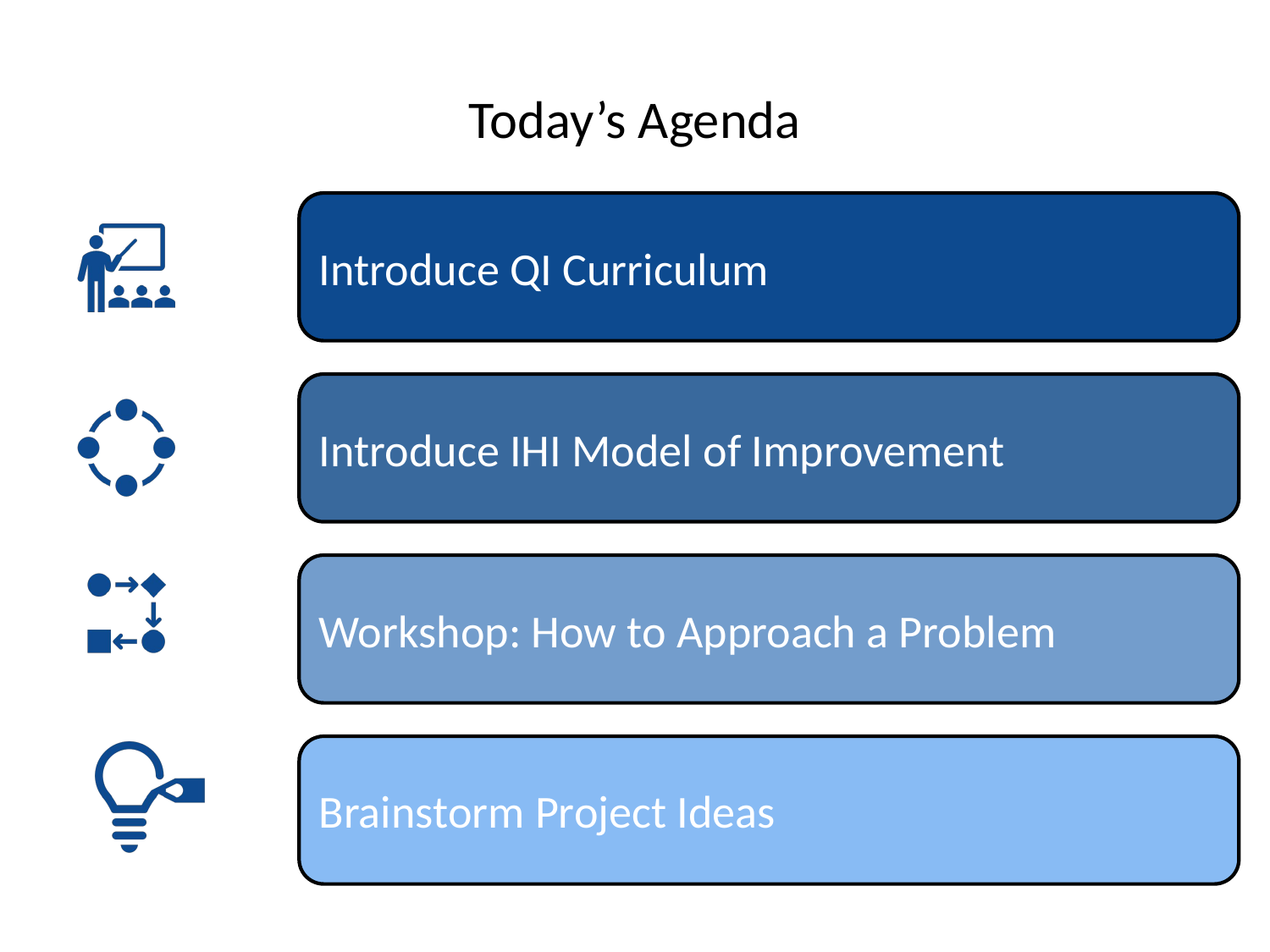

# Today’s Agenda
Introduce QI Curriculum
Introduce IHI Model of Improvement
Workshop: How to Approach a Problem
Brainstorm Project Ideas

## Slide 3
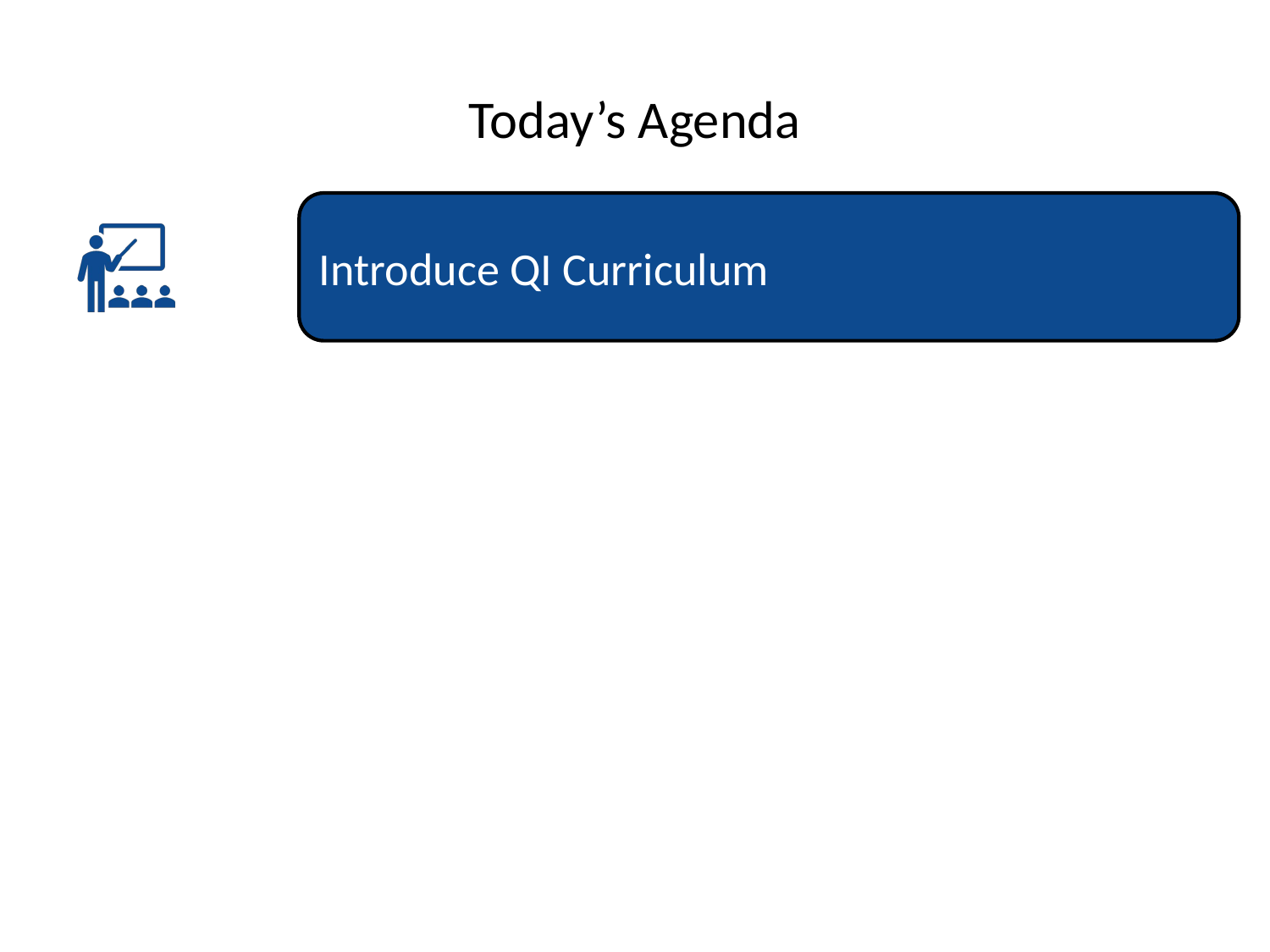

# Today’s Agenda
Introduce QI Curriculum

## Slide 4
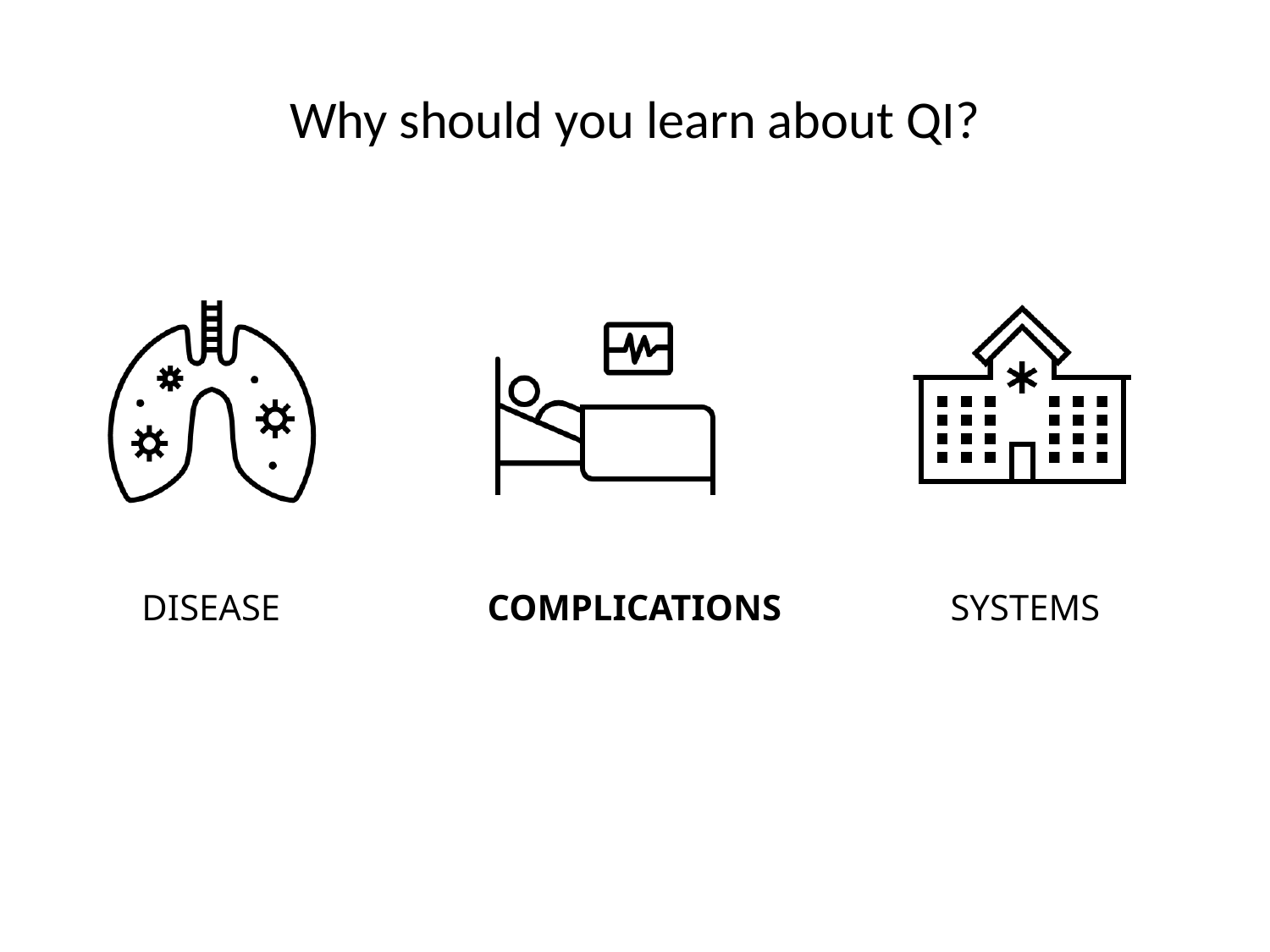

# Why should you learn about QI?
DISEASE
COMPLICATIONS
SYSTEMS

## Slide 5
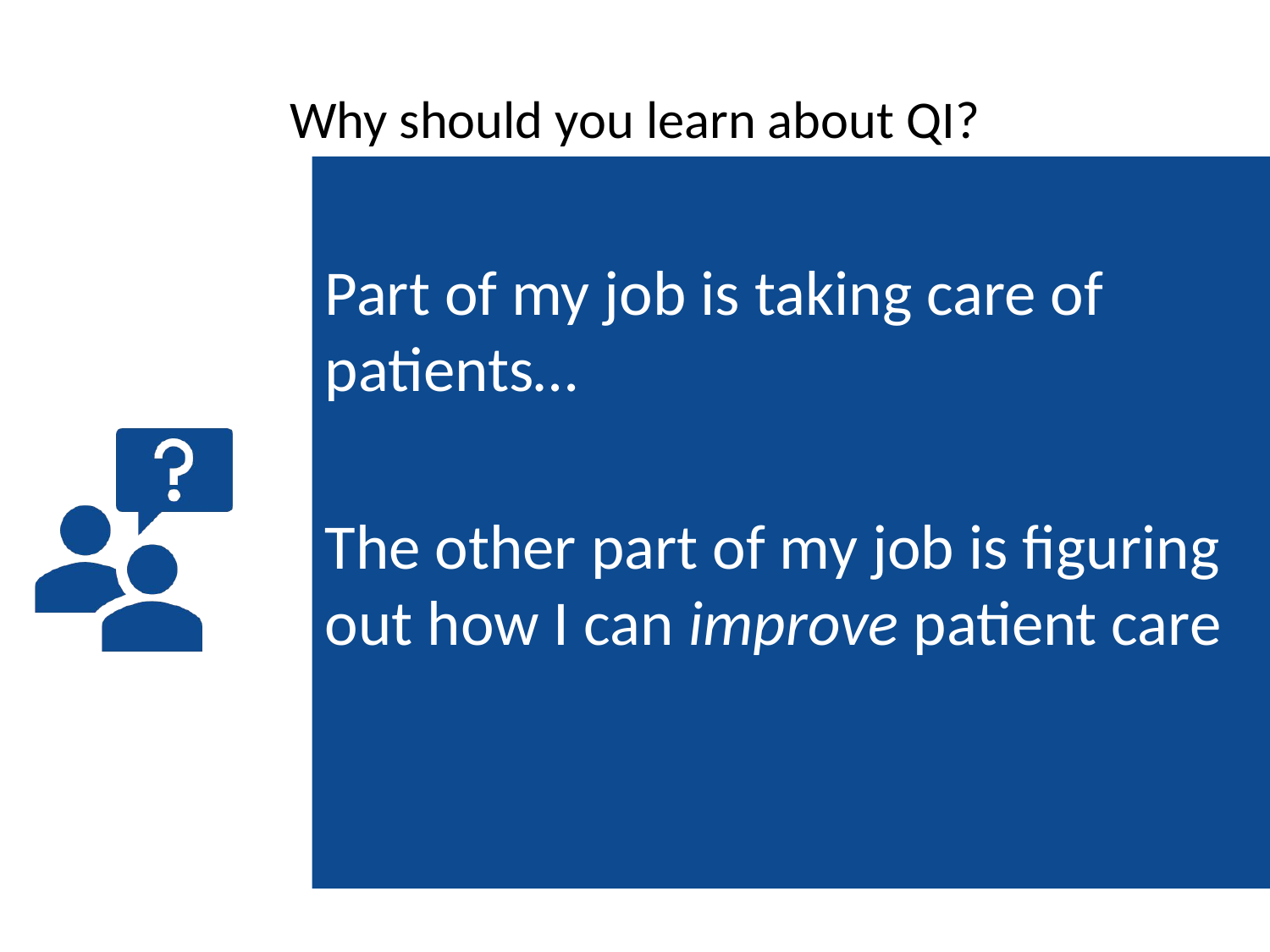

# Why should you learn about QI?
Part of my job is taking care of patients…
The other part of my job is figuring out how I can improve patient care

## Slide 6
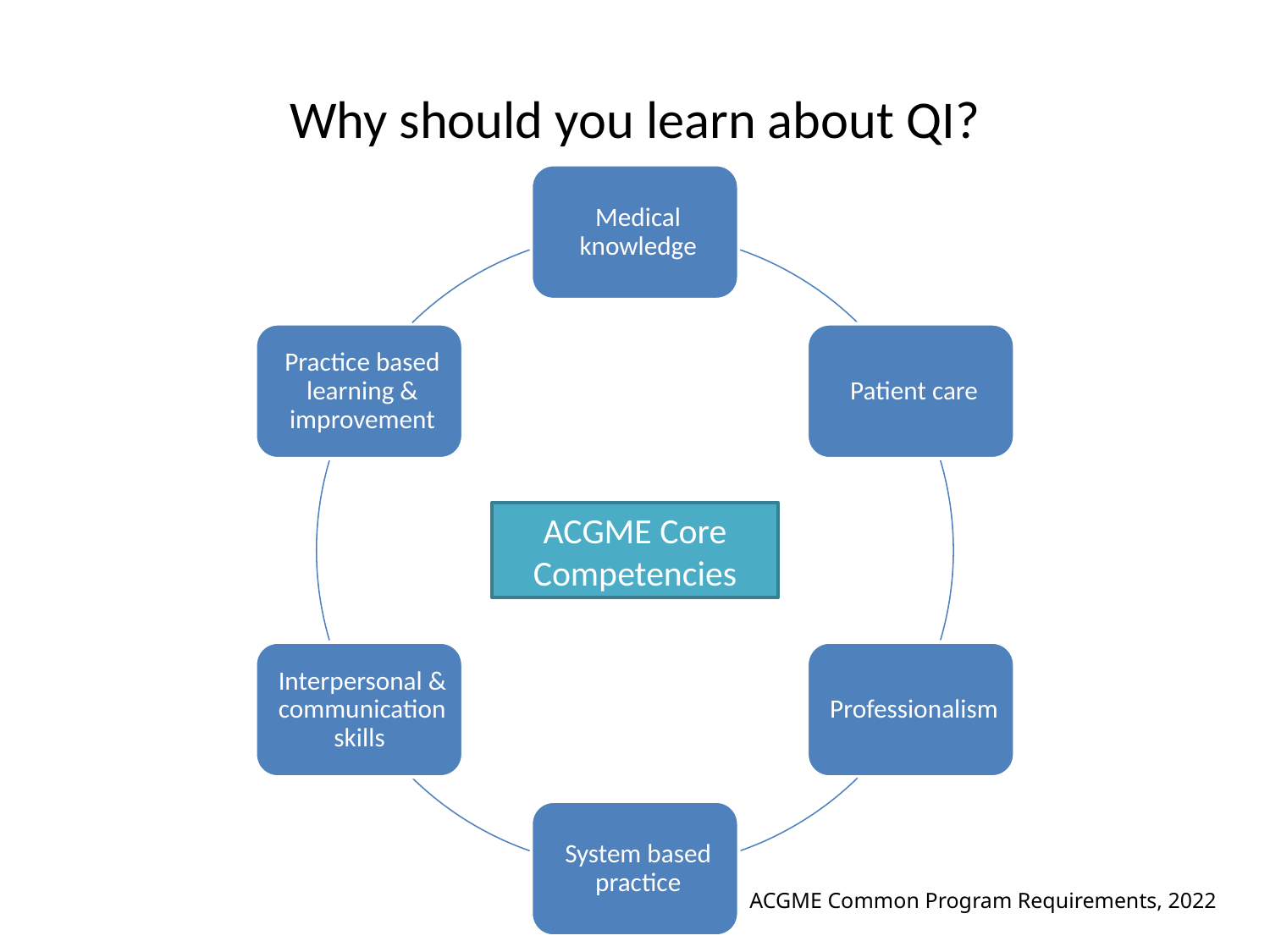

# Why should you learn about QI?
ACGME Core Competencies
ACGME Common Program Requirements, 2022

## Slide 7
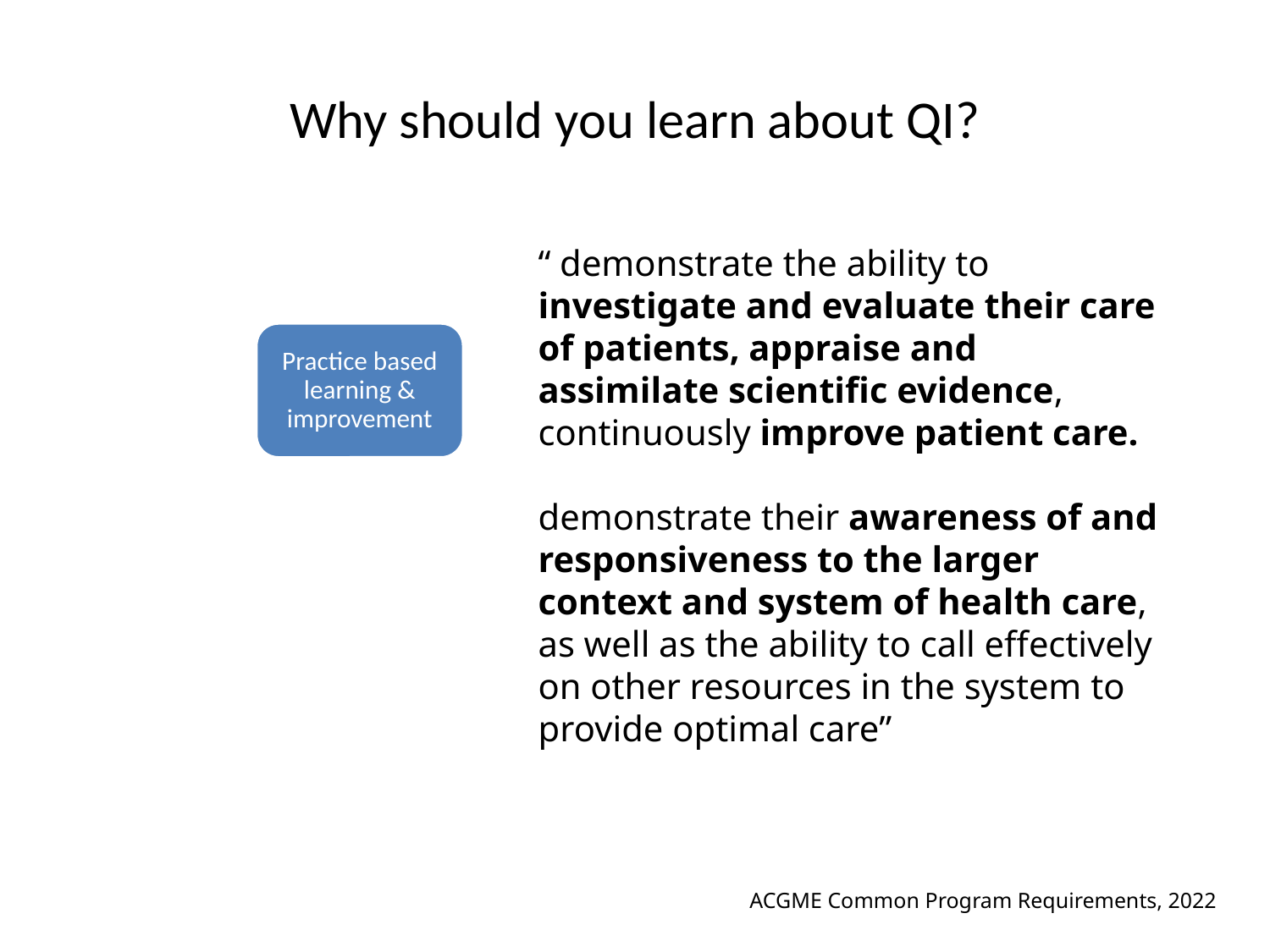

# Why should you learn about QI?
“ demonstrate the ability to investigate and evaluate their care of patients, appraise and assimilate scientific evidence, continuously improve patient care.
demonstrate their awareness of and responsiveness to the larger context and system of health care, as well as the ability to call effectively on other resources in the system to provide optimal care”
Practice based learning & improvement
ACGME Common Program Requirements, 2022

## Slide 8
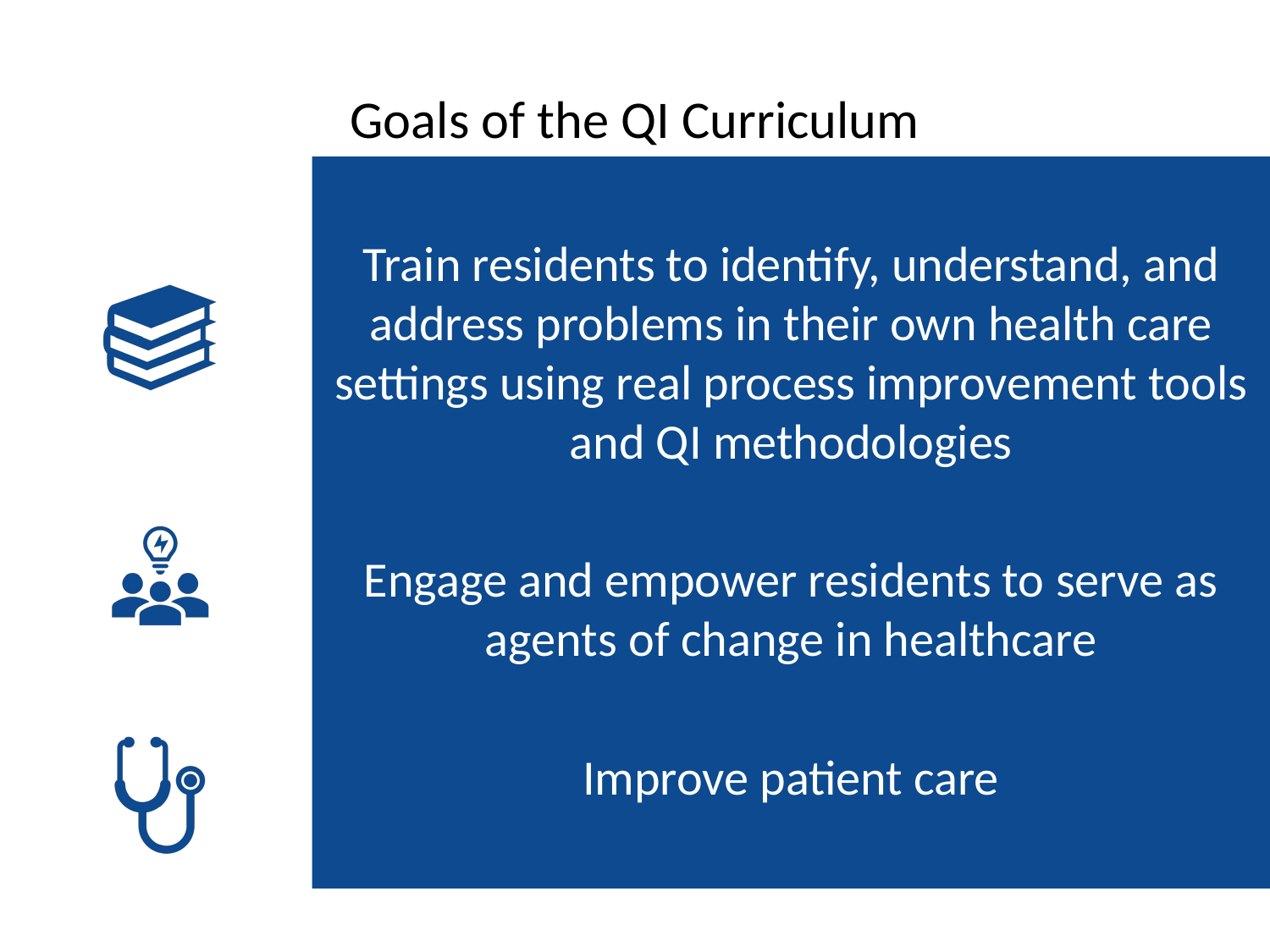

# Goals of the QI Curriculum
Train residents to identify, understand, and address problems in their own health care settings using real process improvement tools and QI methodologies
Engage and empower residents to serve as agents of change in healthcare
Improve patient care

## Slide 9
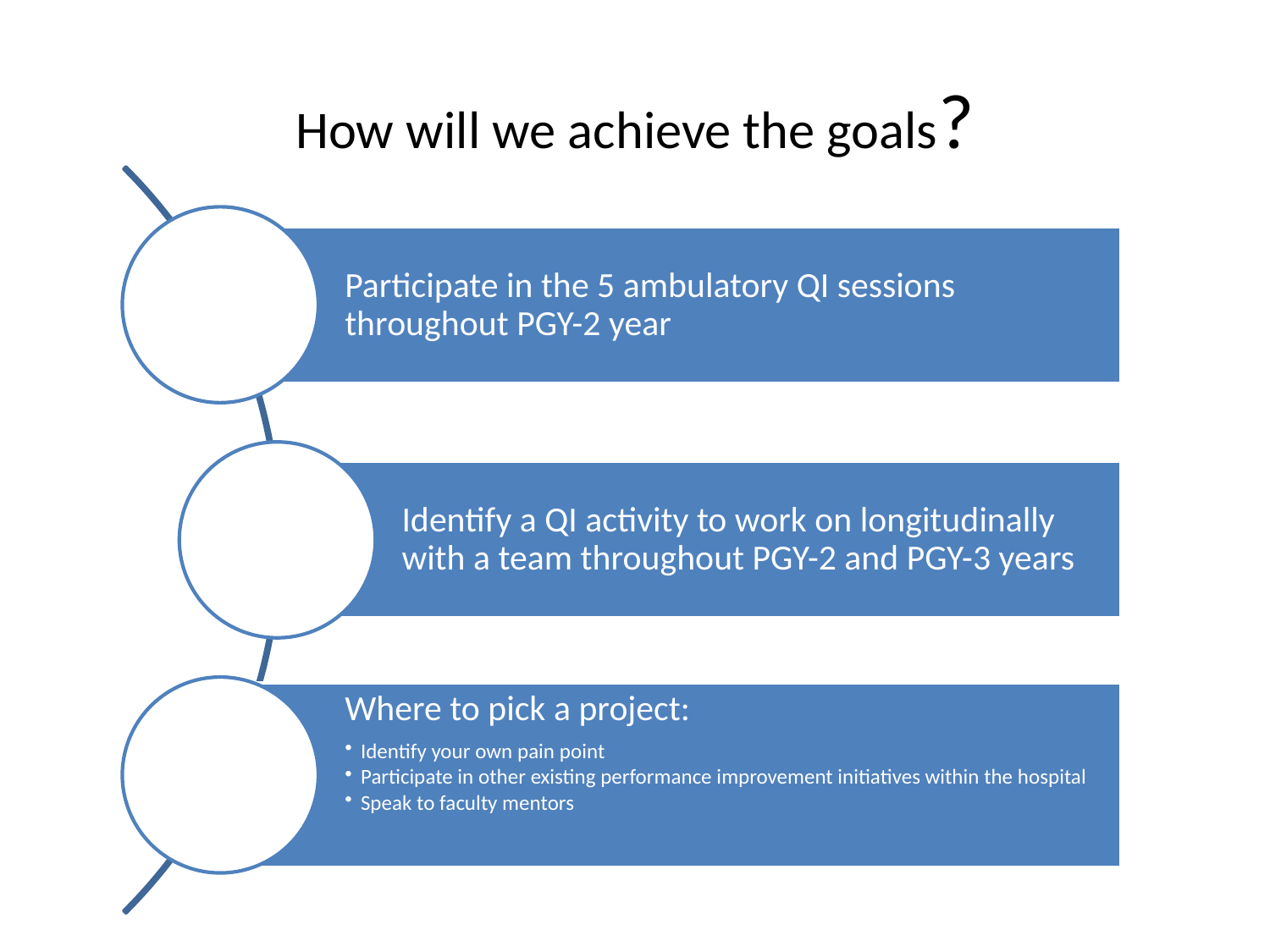

# How will we achieve the goals?

## Slide 10
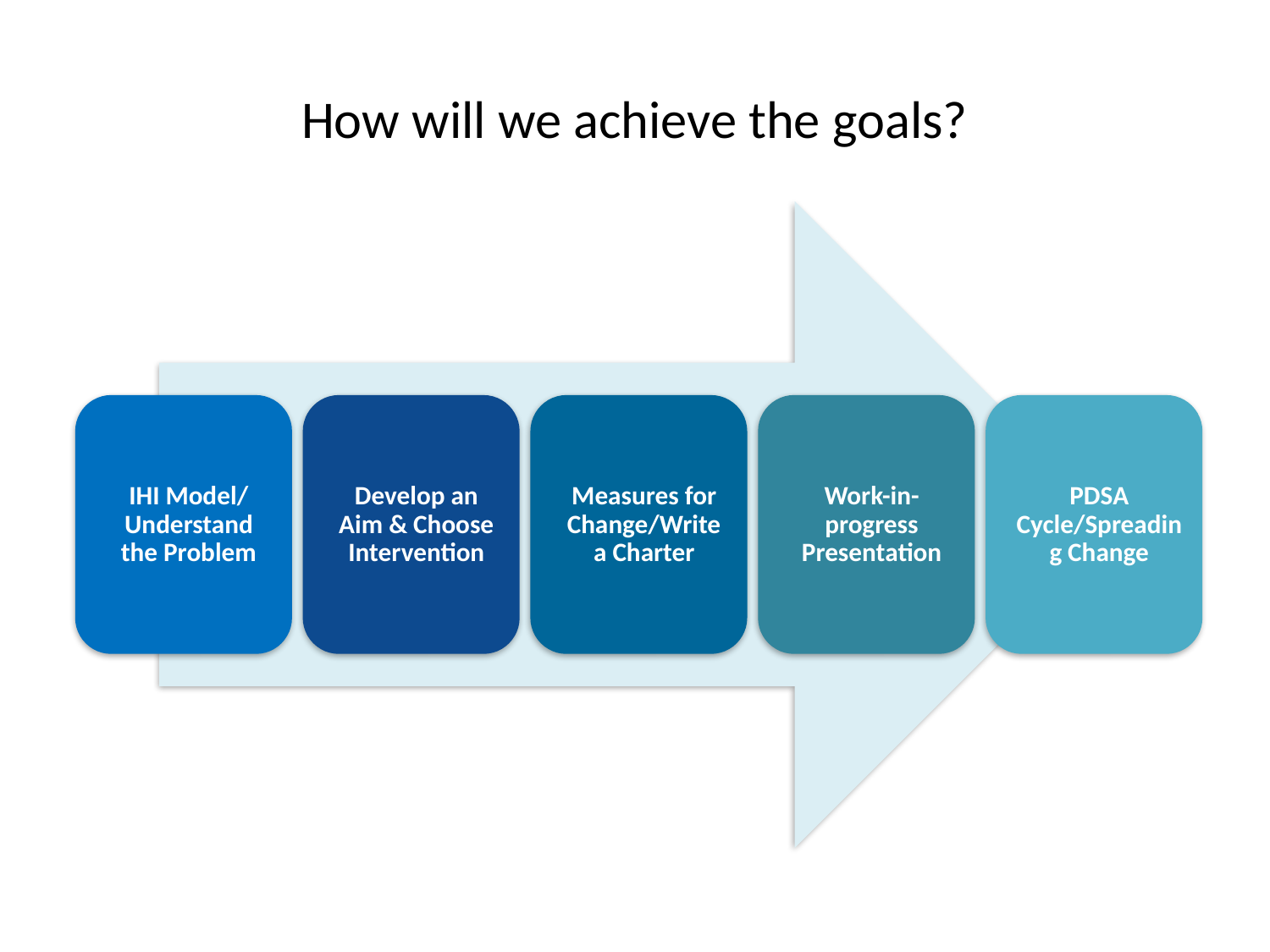

# How will we achieve the goals?

## Slide 11
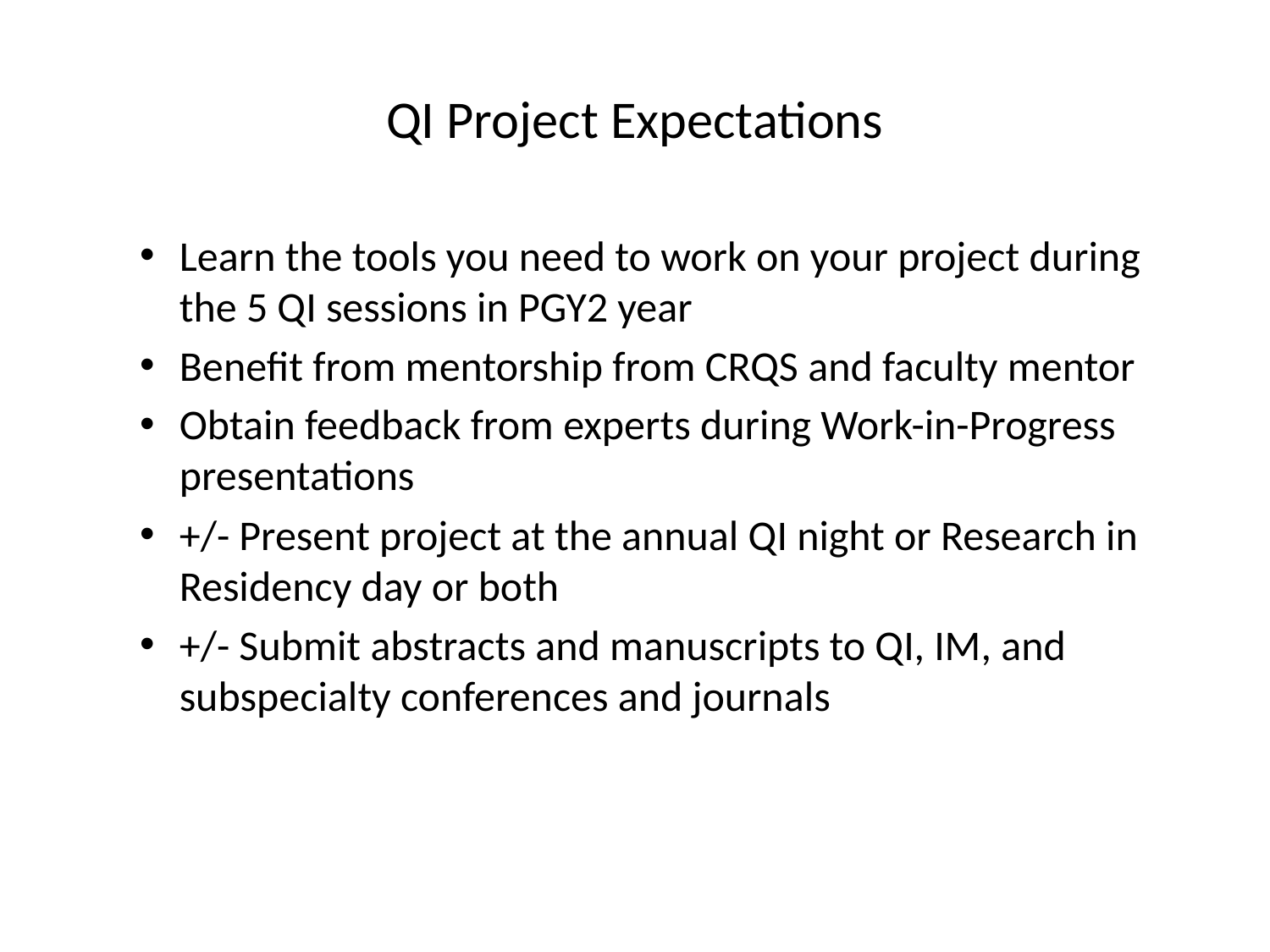

# QI Project Expectations
Learn the tools you need to work on your project during the 5 QI sessions in PGY2 year
Benefit from mentorship from CRQS and faculty mentor
Obtain feedback from experts during Work-in-Progress presentations
+/- Present project at the annual QI night or Research in Residency day or both
+/- Submit abstracts and manuscripts to QI, IM, and subspecialty conferences and journals

## Slide 12
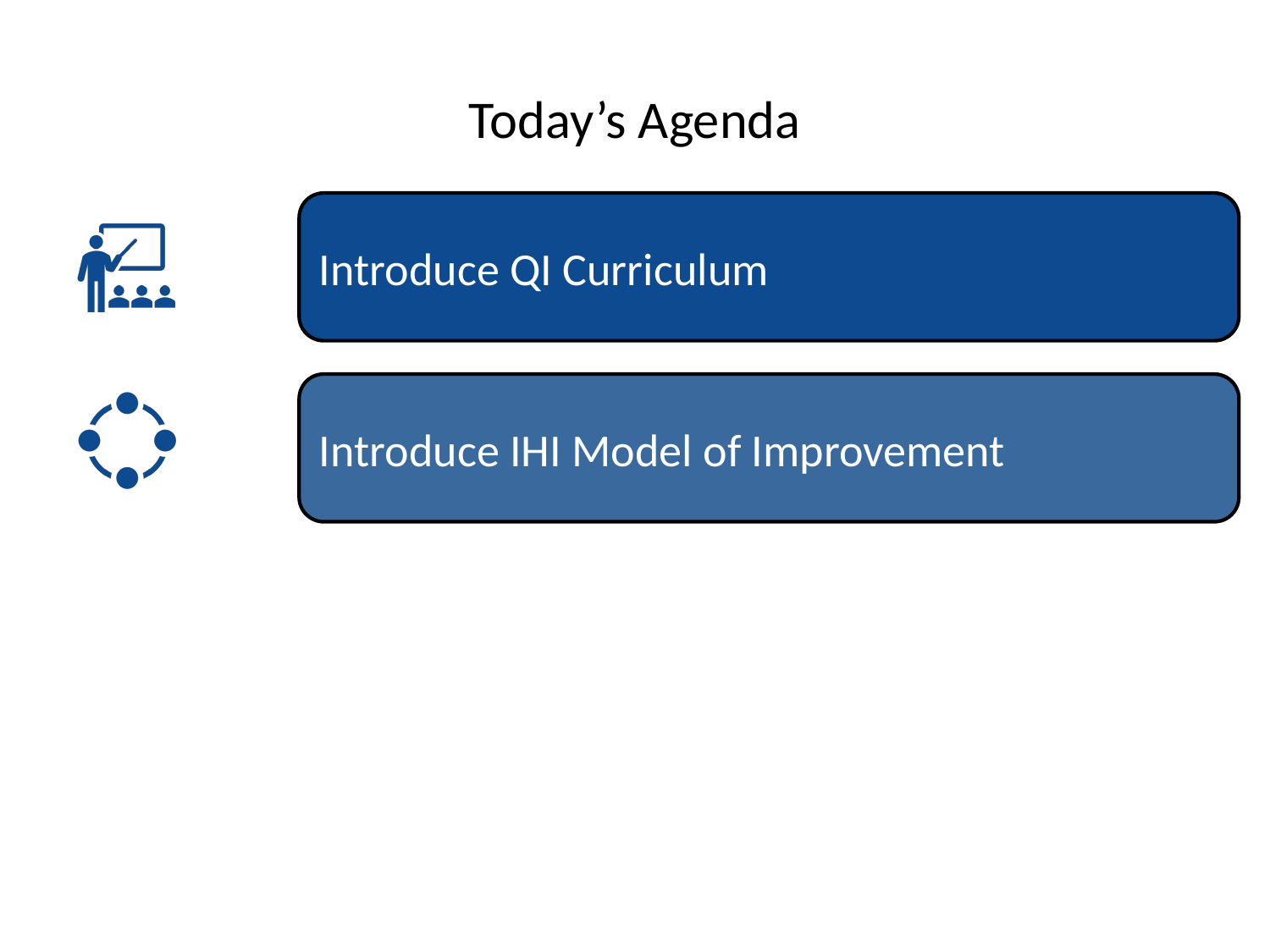

# Today’s Agenda
Introduce QI Curriculum
Introduce IHI Model of Improvement

## Slide 13
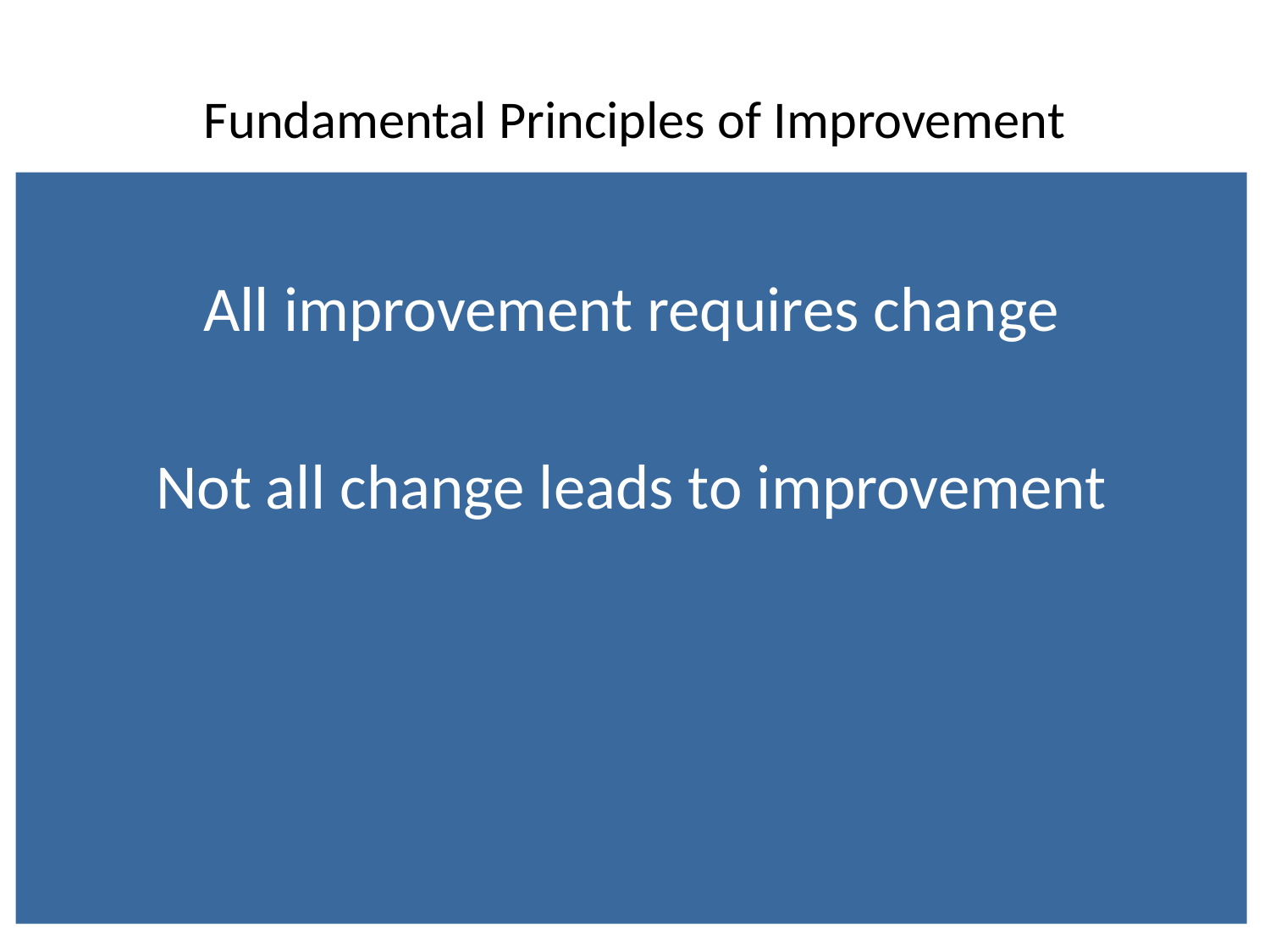

# Fundamental Principles of Improvement
All improvement requires change
Not all change leads to improvement

## Slide 14
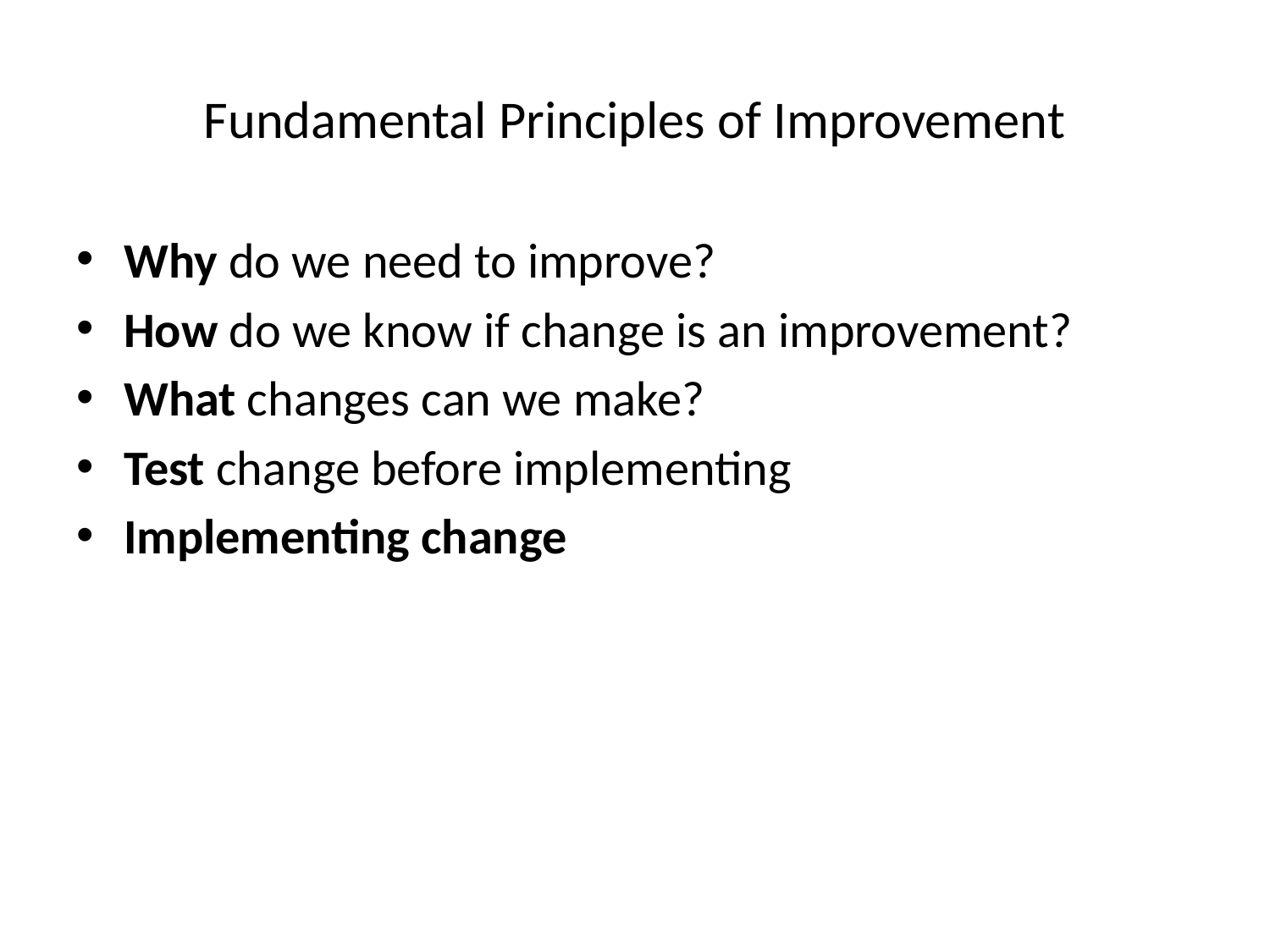

# Fundamental Principles of Improvement
Why do we need to improve?
How do we know if change is an improvement?
What changes can we make?
Test change before implementing
Implementing change

## Slide 15
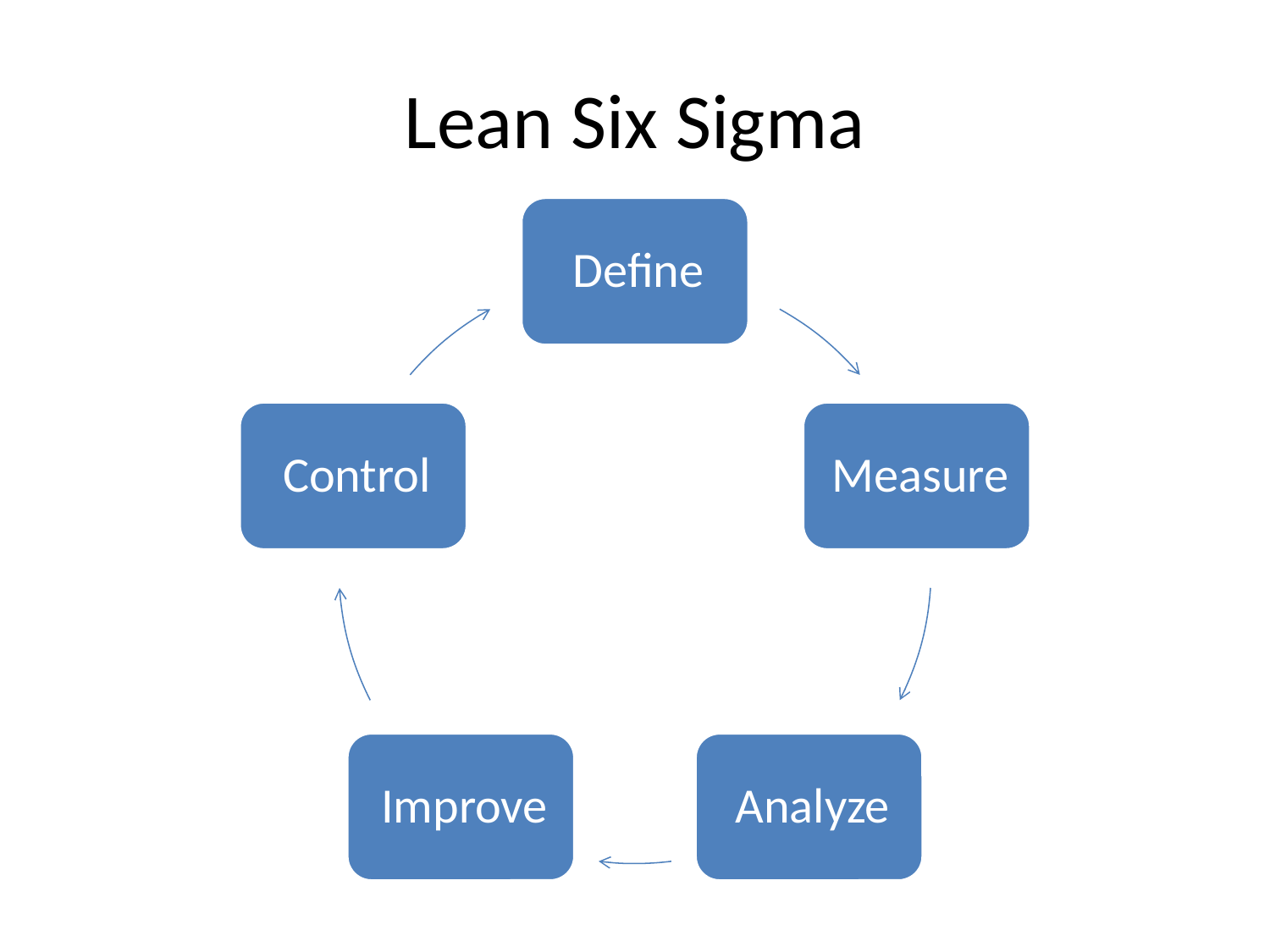

# Lean Six Sigma

## Slide 16
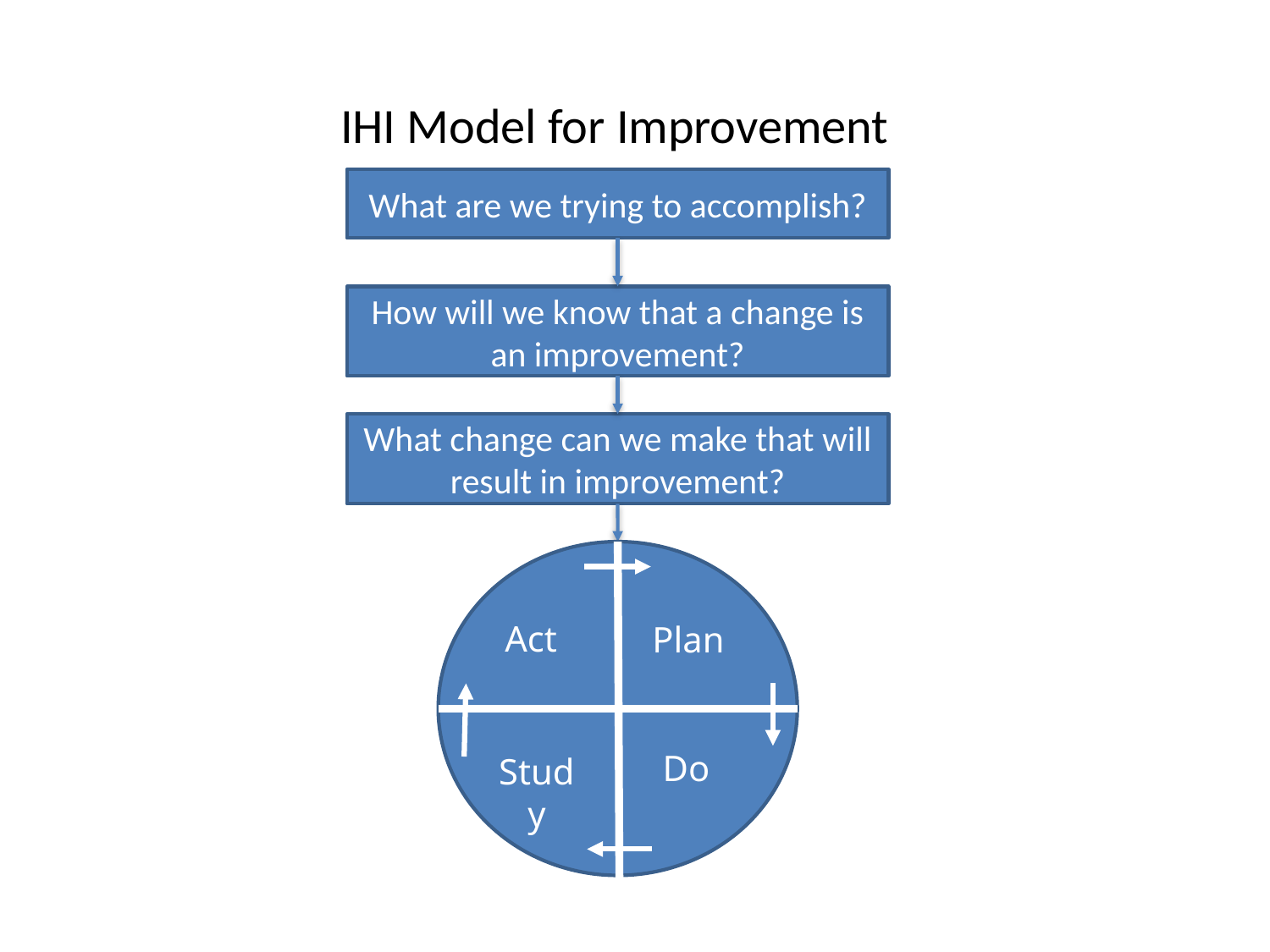

IHI Model for Improvement
What are we trying to accomplish?
How will we know that a change is an improvement?
What change can we make that will result in improvement?
Act
Plan
Do
Study

## Slide 17
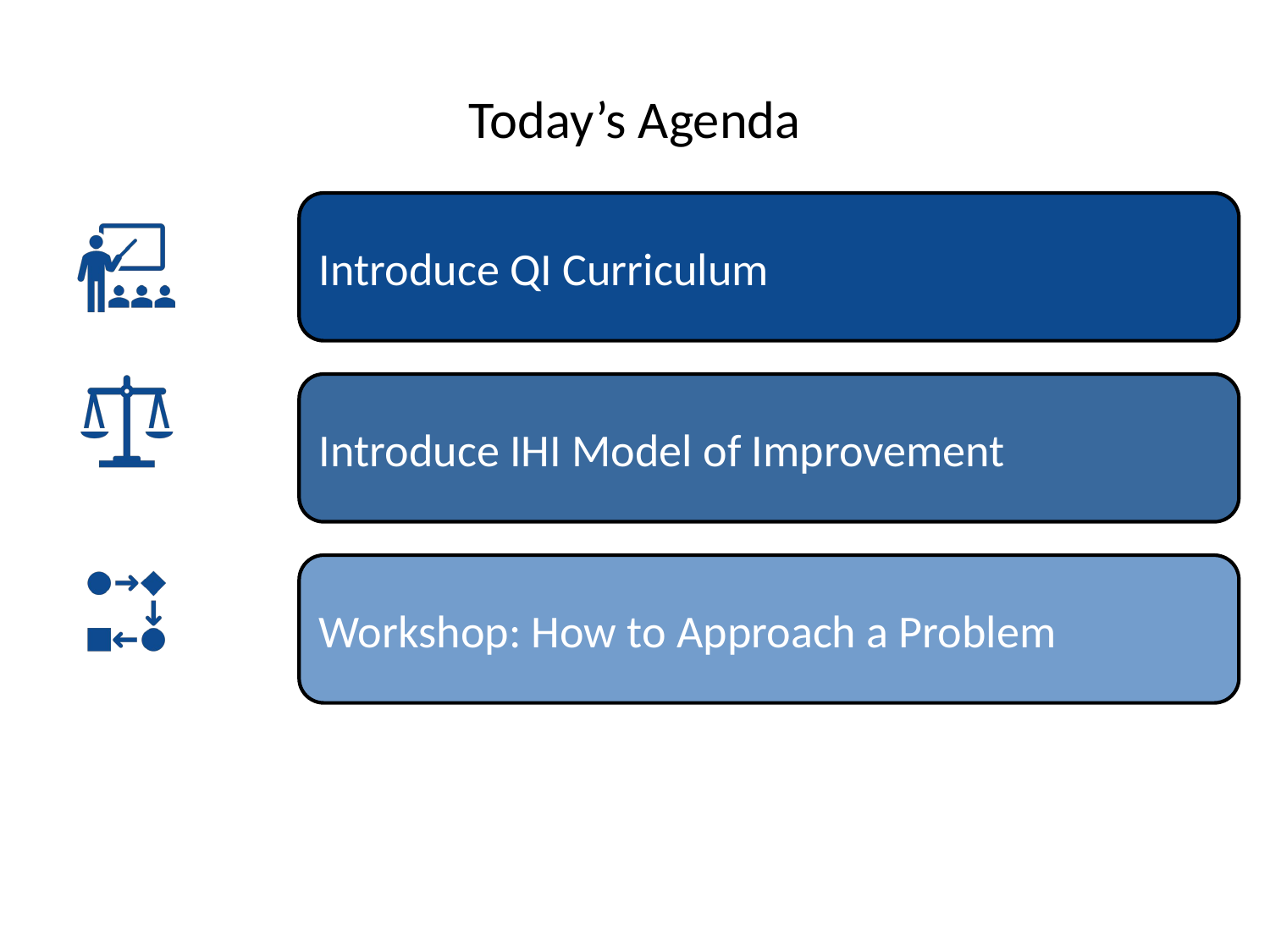

# Today’s Agenda
Introduce QI Curriculum
Introduce IHI Model of Improvement
Workshop: How to Approach a Problem

## Slide 18
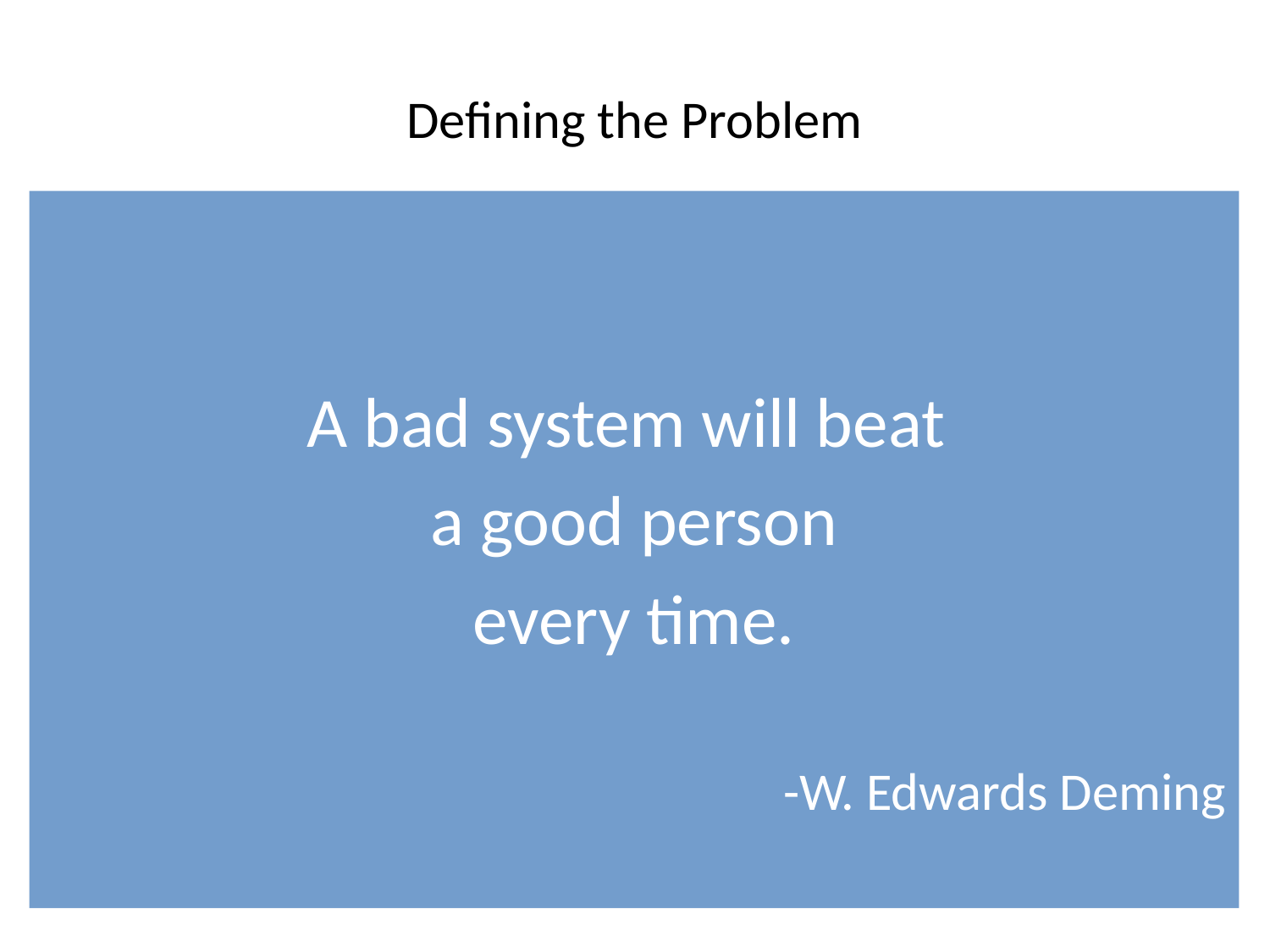

# Defining the Problem
A bad system will beat
a good person
every time.
-W. Edwards Deming

## Slide 19
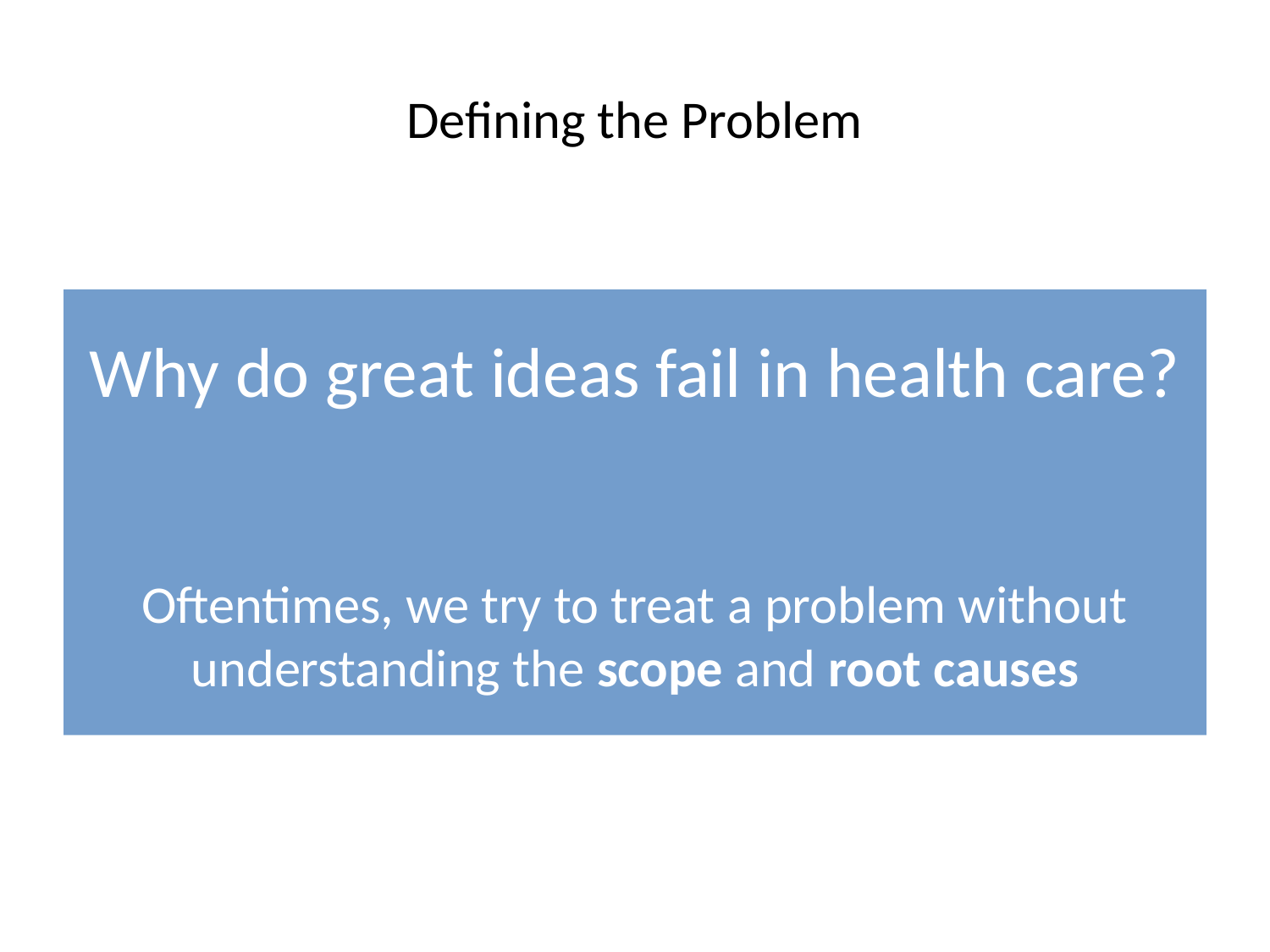

# Defining the Problem
Why do great ideas fail in health care?
Oftentimes, we try to treat a problem without understanding the scope and root causes

## Slide 20
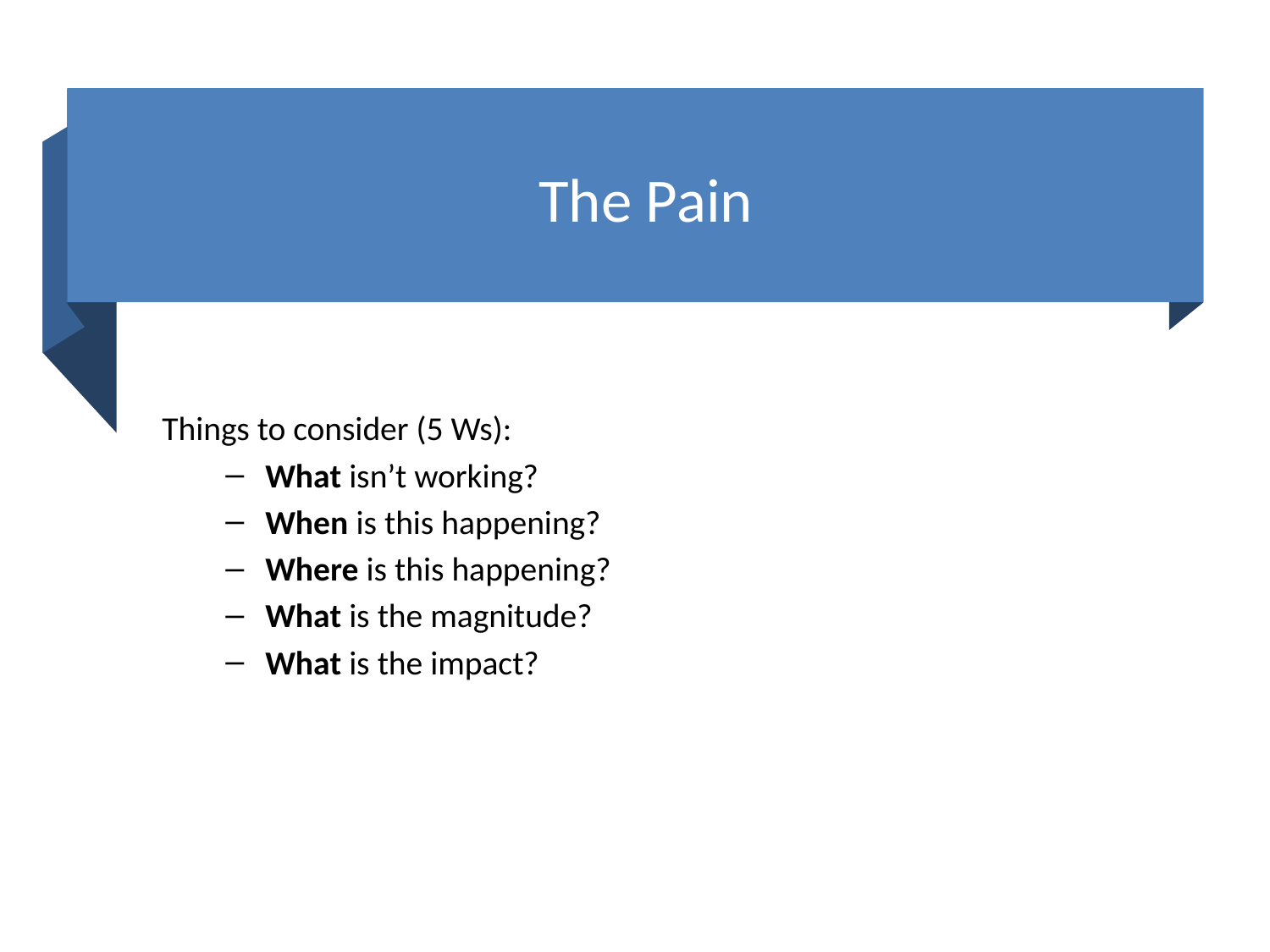

# The Pain
Things to consider (5 Ws):
What isn’t working?
When is this happening?
Where is this happening?
What is the magnitude?
What is the impact?

## Slide 21
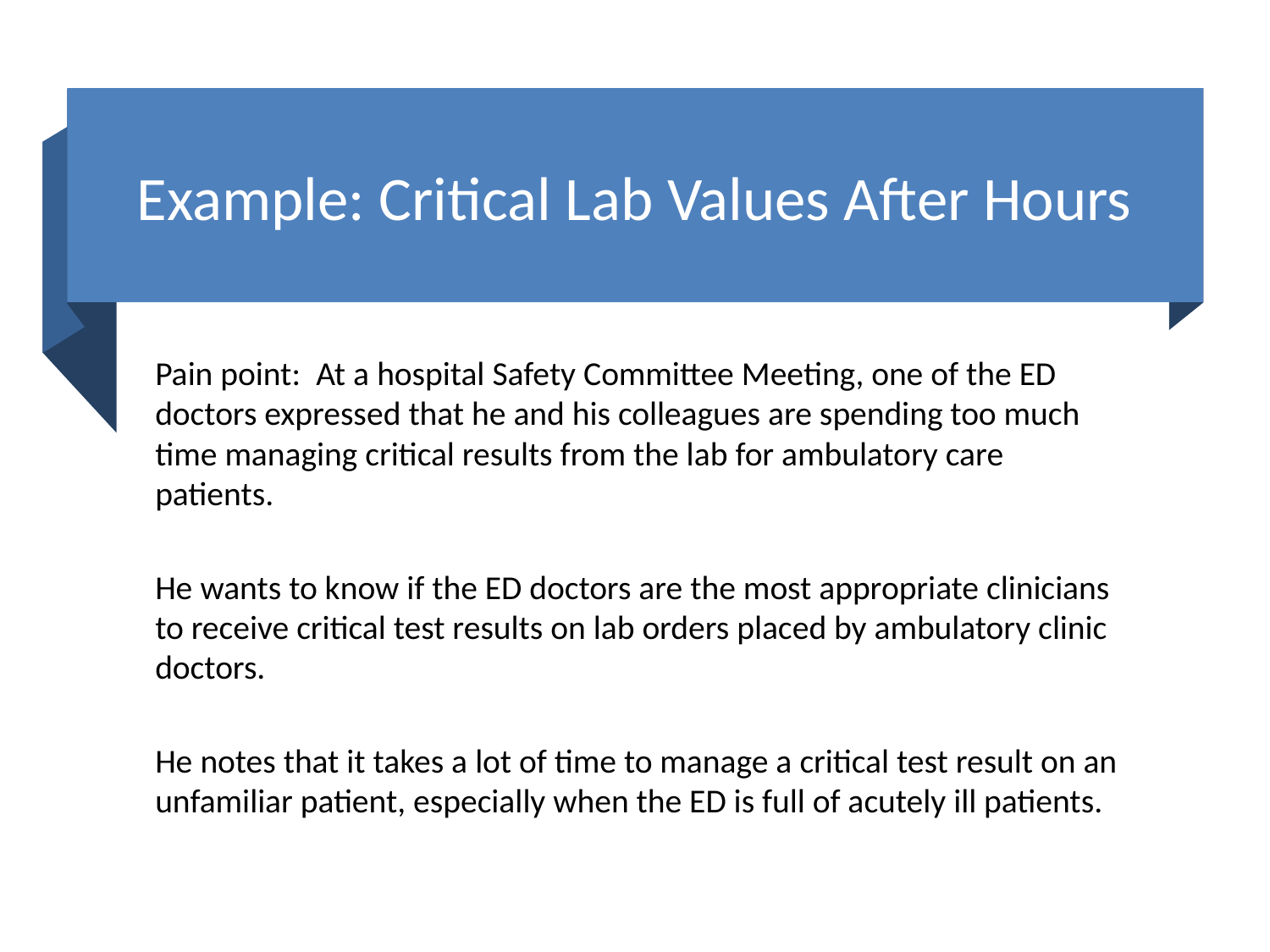

# Example: Critical Lab Values After Hours
Pain point:  At a hospital Safety Committee Meeting, one of the ED doctors expressed that he and his colleagues are spending too much time managing critical results from the lab for ambulatory care patients.
He wants to know if the ED doctors are the most appropriate clinicians to receive critical test results on lab orders placed by ambulatory clinic doctors.
He notes that it takes a lot of time to manage a critical test result on an unfamiliar patient, especially when the ED is full of acutely ill patients.

## Slide 22
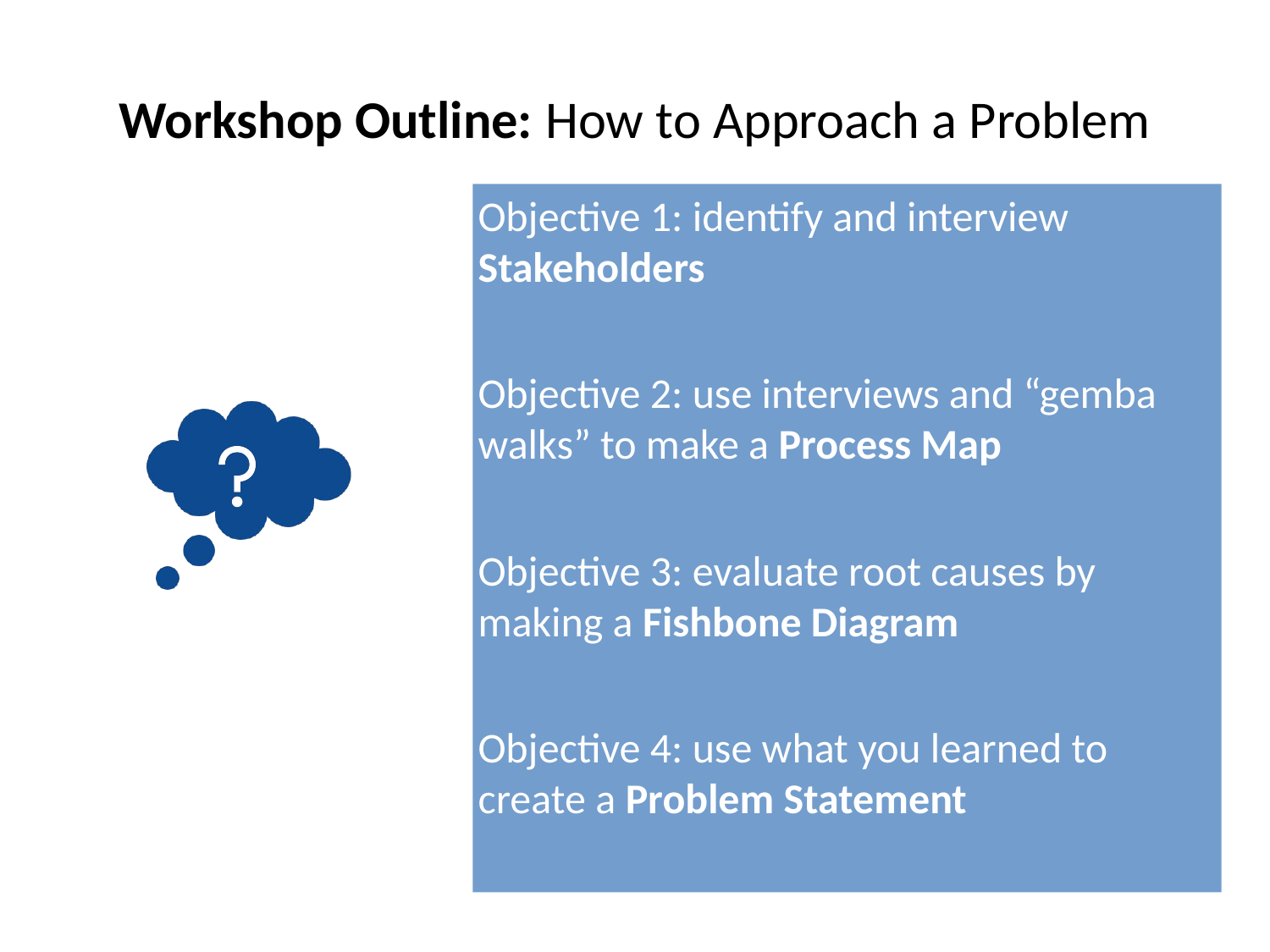

# Workshop Outline: How to Approach a Problem
Objective 1: identify and interview Stakeholders
Objective 2: use interviews and “gemba walks” to make a Process Map
Objective 3: evaluate root causes by making a Fishbone Diagram
Objective 4: use what you learned to create a Problem Statement

## Slide 23
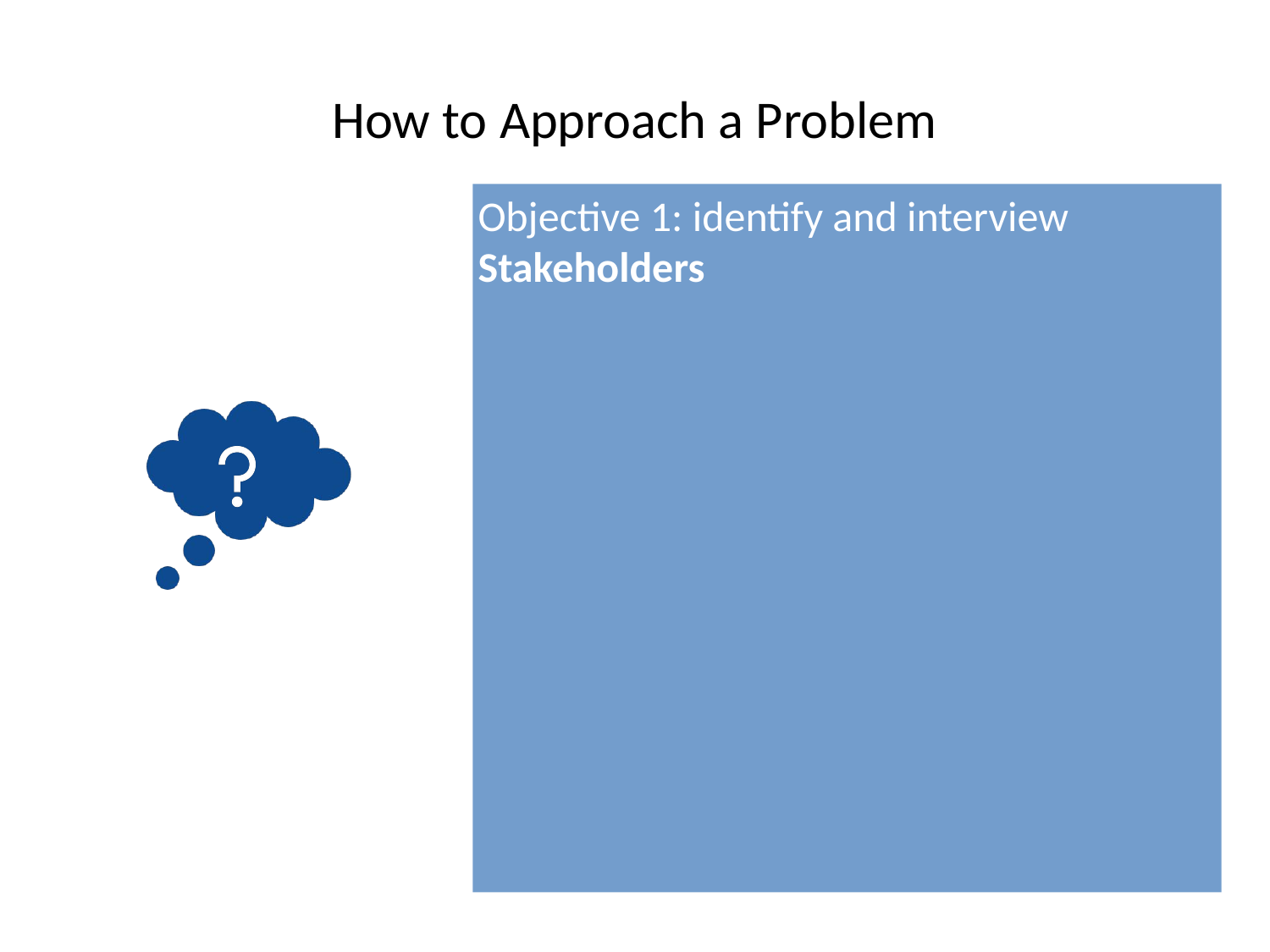

# How to Approach a Problem
Objective 1: identify and interview Stakeholders

## Slide 24
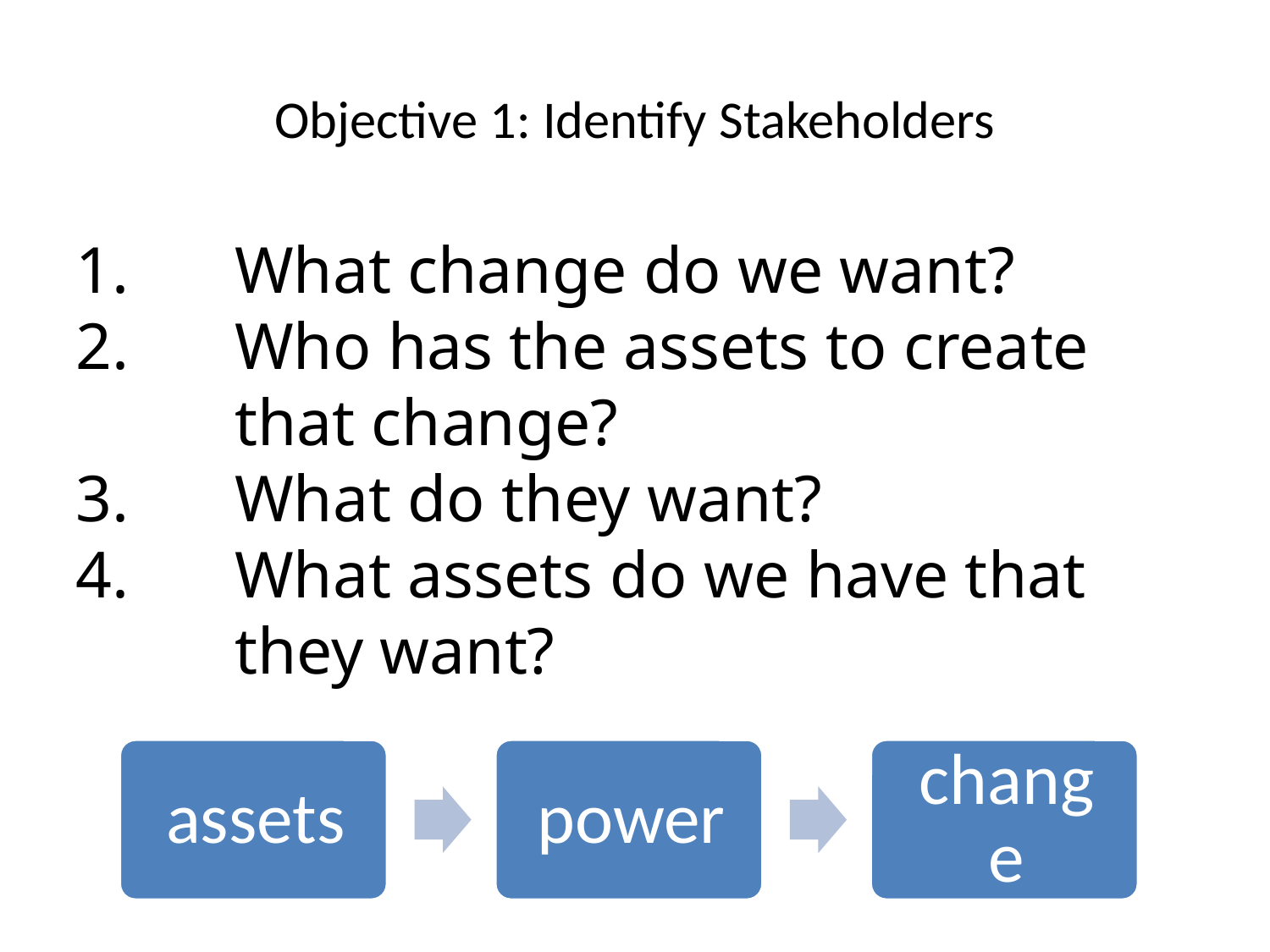

# Objective 1: Identify Stakeholders
What change do we want?
Who has the assets to create that change?
What do they want?
What assets do we have that they want?

## Slide 25
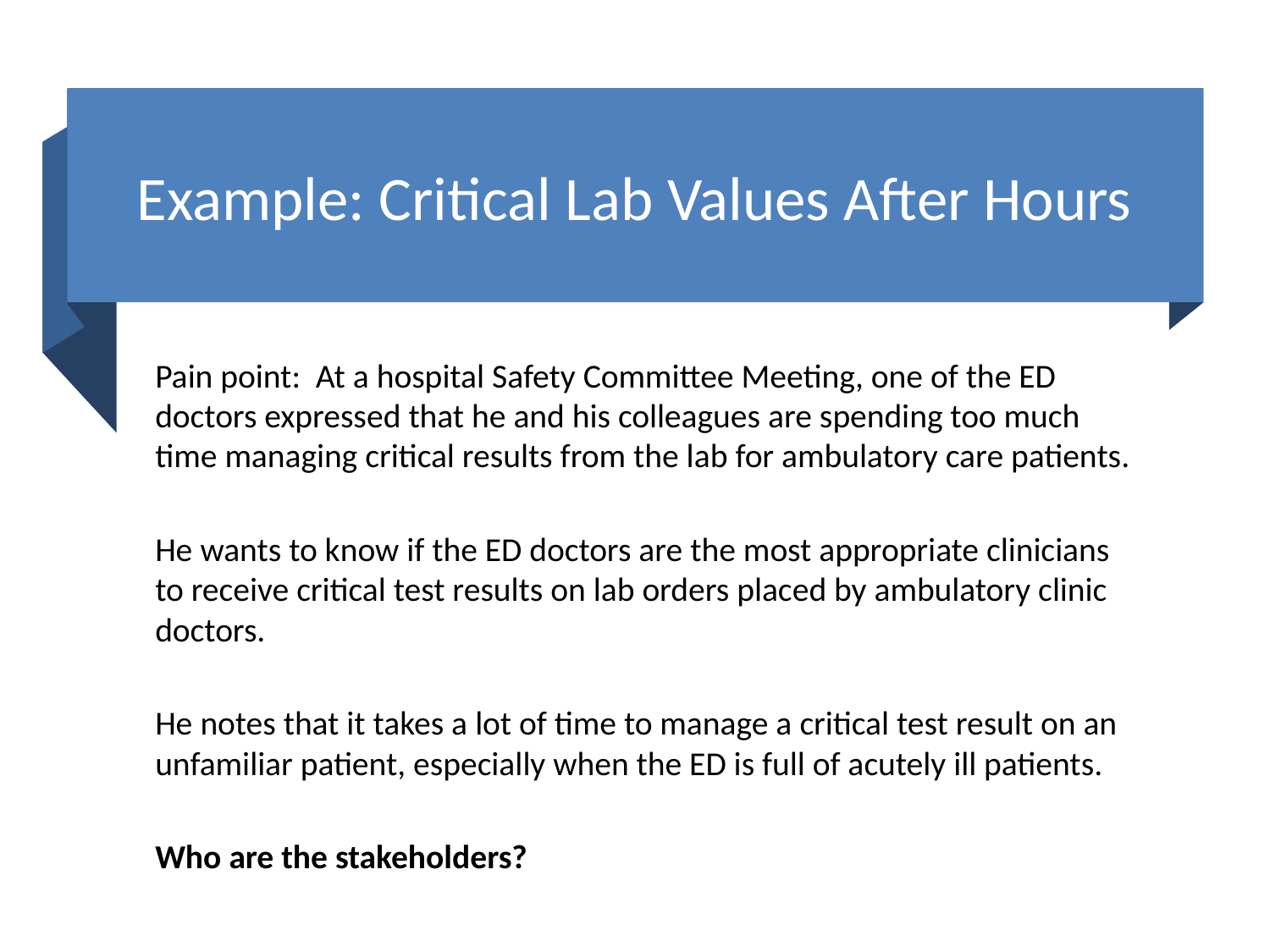

# Example: Critical Lab Values After Hours
Pain point: At a hospital Safety Committee Meeting, one of the ED doctors expressed that he and his colleagues are spending too much time managing critical results from the lab for ambulatory care patients.
He wants to know if the ED doctors are the most appropriate clinicians to receive critical test results on lab orders placed by ambulatory clinic doctors.
He notes that it takes a lot of time to manage a critical test result on an unfamiliar patient, especially when the ED is full of acutely ill patients.
Who are the stakeholders?

## Slide 26
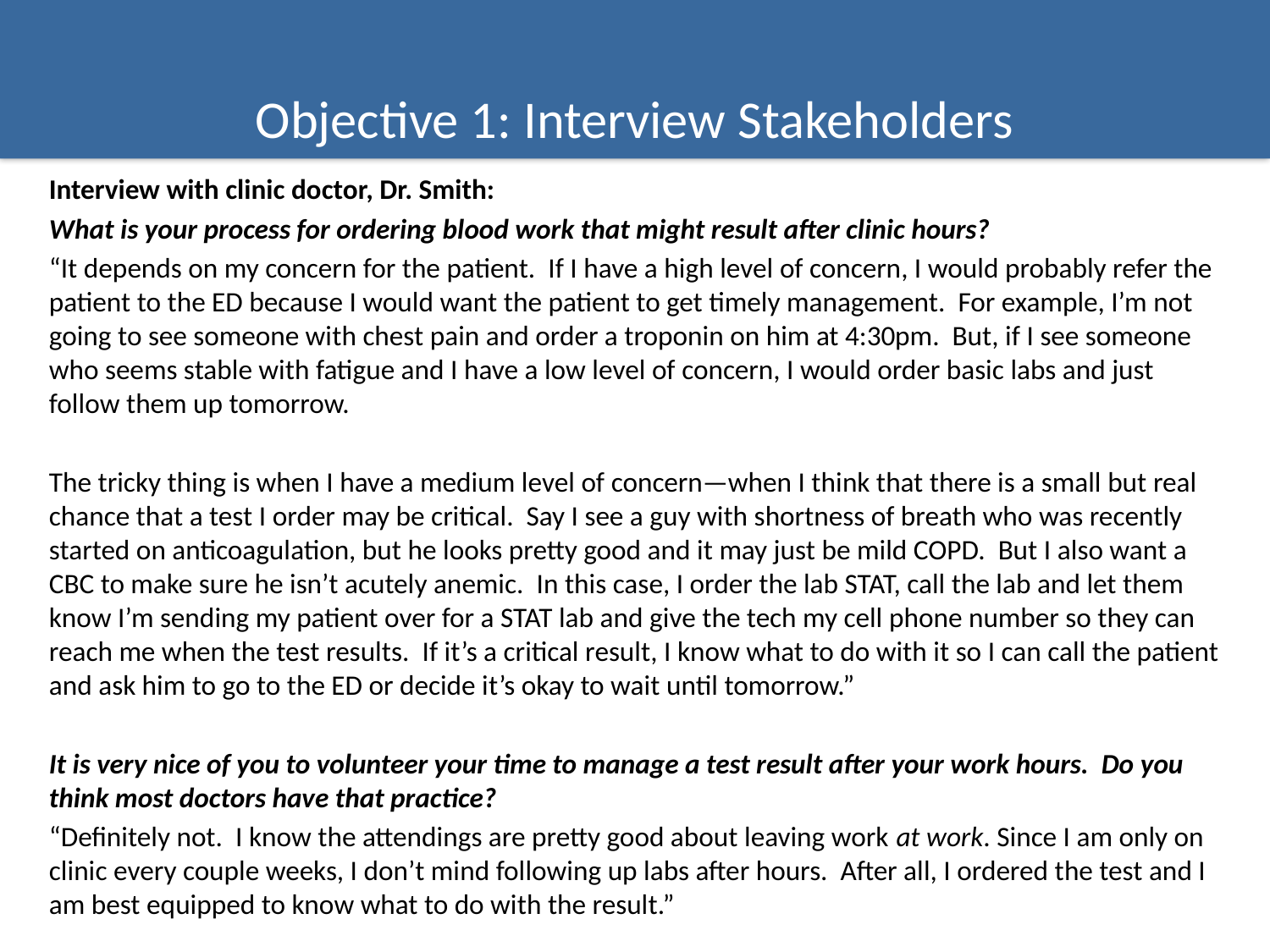

# Objective 1: Interview Stakeholders
Interview with clinic doctor, Dr. Smith:
What is your process for ordering blood work that might result after clinic hours?
“It depends on my concern for the patient. If I have a high level of concern, I would probably refer the patient to the ED because I would want the patient to get timely management. For example, I’m not going to see someone with chest pain and order a troponin on him at 4:30pm. But, if I see someone who seems stable with fatigue and I have a low level of concern, I would order basic labs and just follow them up tomorrow.
The tricky thing is when I have a medium level of concern—when I think that there is a small but real chance that a test I order may be critical. Say I see a guy with shortness of breath who was recently started on anticoagulation, but he looks pretty good and it may just be mild COPD. But I also want a CBC to make sure he isn’t acutely anemic. In this case, I order the lab STAT, call the lab and let them know I’m sending my patient over for a STAT lab and give the tech my cell phone number so they can reach me when the test results. If it’s a critical result, I know what to do with it so I can call the patient and ask him to go to the ED or decide it’s okay to wait until tomorrow.”
It is very nice of you to volunteer your time to manage a test result after your work hours. Do you think most doctors have that practice?
“Definitely not. I know the attendings are pretty good about leaving work at work. Since I am only on clinic every couple weeks, I don’t mind following up labs after hours. After all, I ordered the test and I am best equipped to know what to do with the result.”

## Slide 27
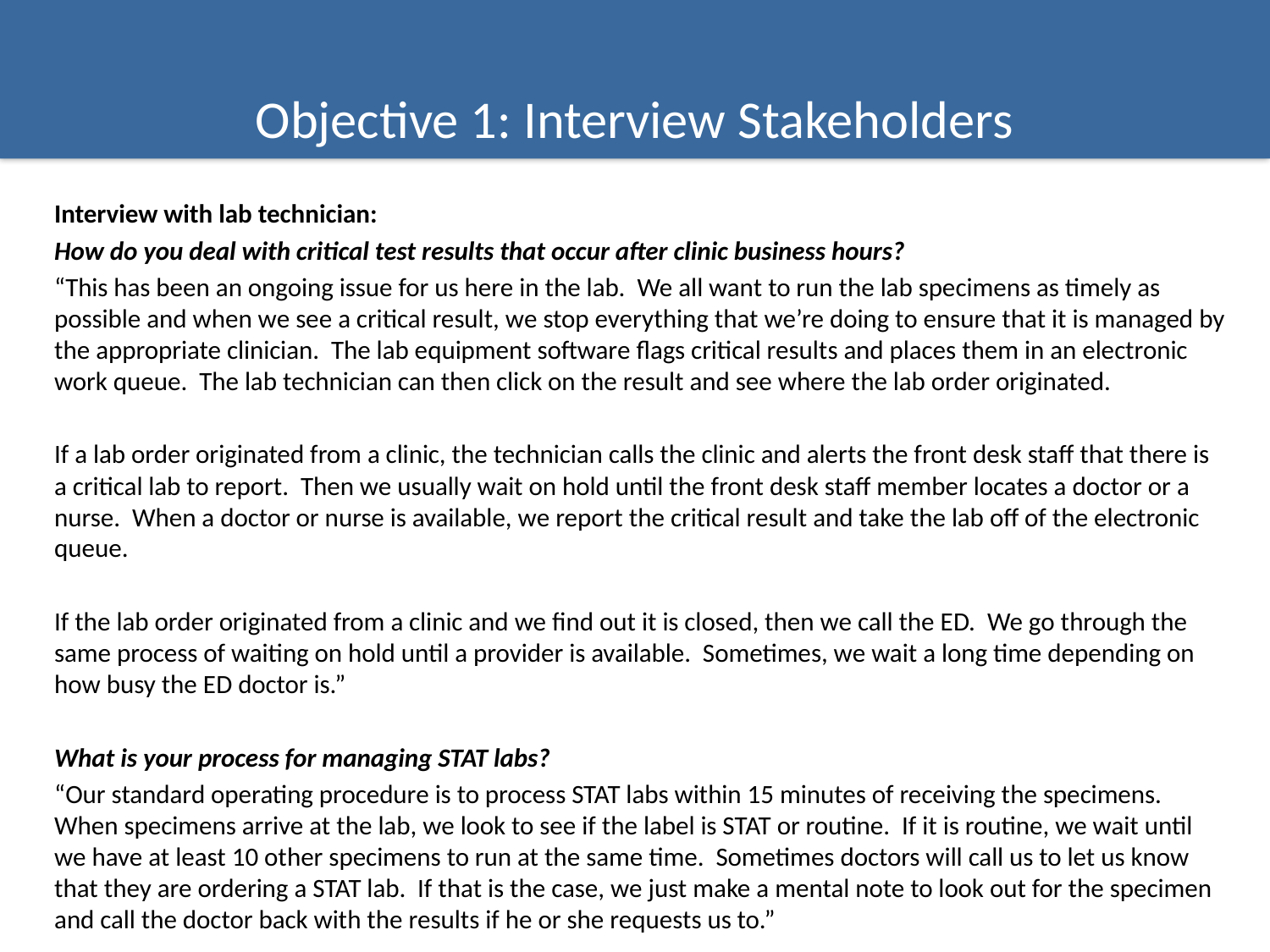

# Objective 1: Interview Stakeholders
Interview with lab technician:
How do you deal with critical test results that occur after clinic business hours?
“This has been an ongoing issue for us here in the lab. We all want to run the lab specimens as timely as possible and when we see a critical result, we stop everything that we’re doing to ensure that it is managed by the appropriate clinician. The lab equipment software flags critical results and places them in an electronic work queue. The lab technician can then click on the result and see where the lab order originated.
If a lab order originated from a clinic, the technician calls the clinic and alerts the front desk staff that there is a critical lab to report. Then we usually wait on hold until the front desk staff member locates a doctor or a nurse. When a doctor or nurse is available, we report the critical result and take the lab off of the electronic queue.
If the lab order originated from a clinic and we find out it is closed, then we call the ED. We go through the same process of waiting on hold until a provider is available. Sometimes, we wait a long time depending on how busy the ED doctor is.”
What is your process for managing STAT labs?
“Our standard operating procedure is to process STAT labs within 15 minutes of receiving the specimens. When specimens arrive at the lab, we look to see if the label is STAT or routine. If it is routine, we wait until we have at least 10 other specimens to run at the same time. Sometimes doctors will call us to let us know that they are ordering a STAT lab. If that is the case, we just make a mental note to look out for the specimen and call the doctor back with the results if he or she requests us to.”

## Slide 28
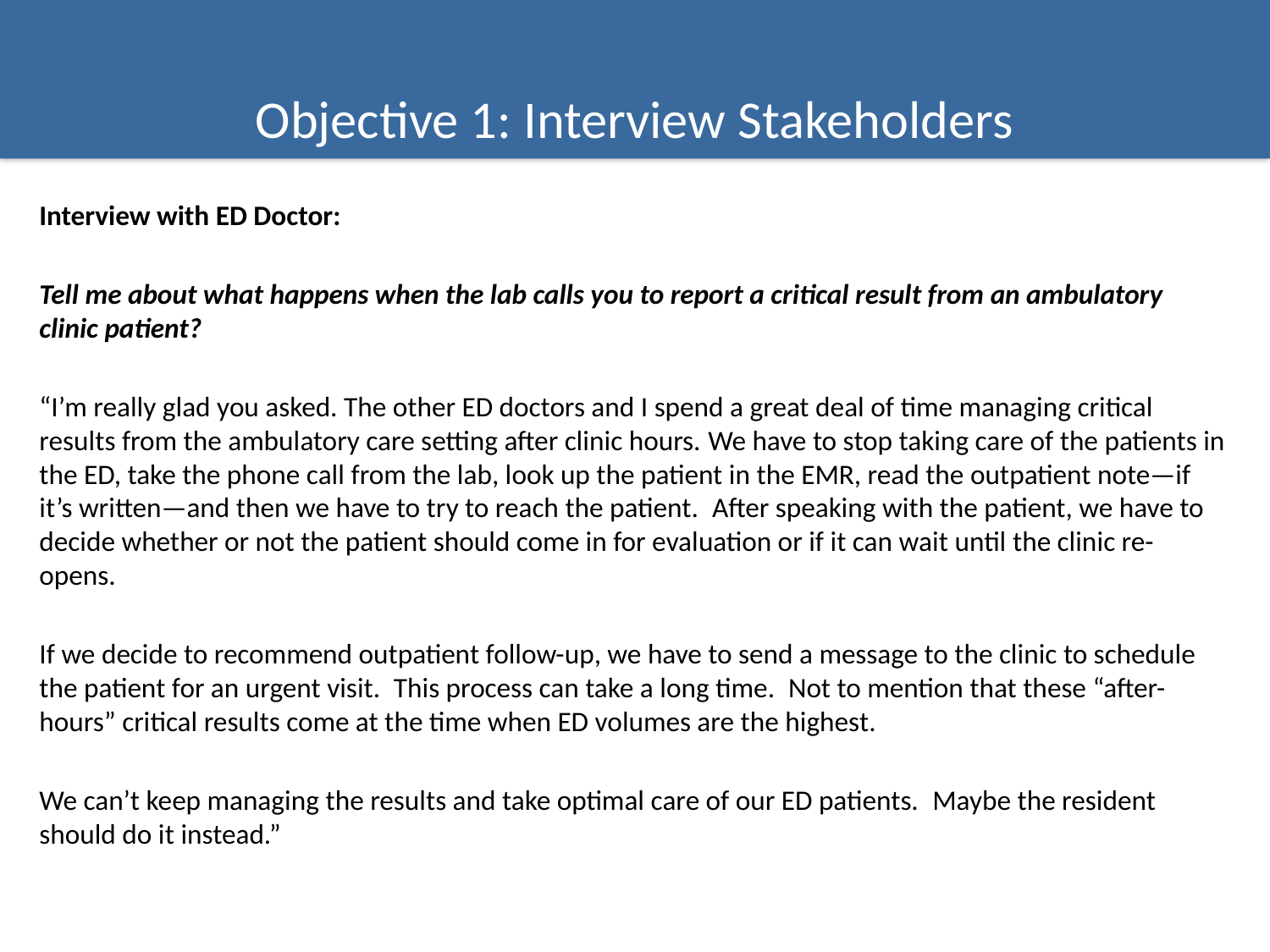

# Objective 1: Interview Stakeholders
Interview with ED Doctor:
Tell me about what happens when the lab calls you to report a critical result from an ambulatory clinic patient?
“I’m really glad you asked. The other ED doctors and I spend a great deal of time managing critical results from the ambulatory care setting after clinic hours. We have to stop taking care of the patients in the ED, take the phone call from the lab, look up the patient in the EMR, read the outpatient note—if it’s written—and then we have to try to reach the patient.  After speaking with the patient, we have to decide whether or not the patient should come in for evaluation or if it can wait until the clinic re-opens.
If we decide to recommend outpatient follow-up, we have to send a message to the clinic to schedule the patient for an urgent visit.  This process can take a long time.  Not to mention that these “after-hours” critical results come at the time when ED volumes are the highest.
We can’t keep managing the results and take optimal care of our ED patients.  Maybe the resident should do it instead.”

## Slide 29
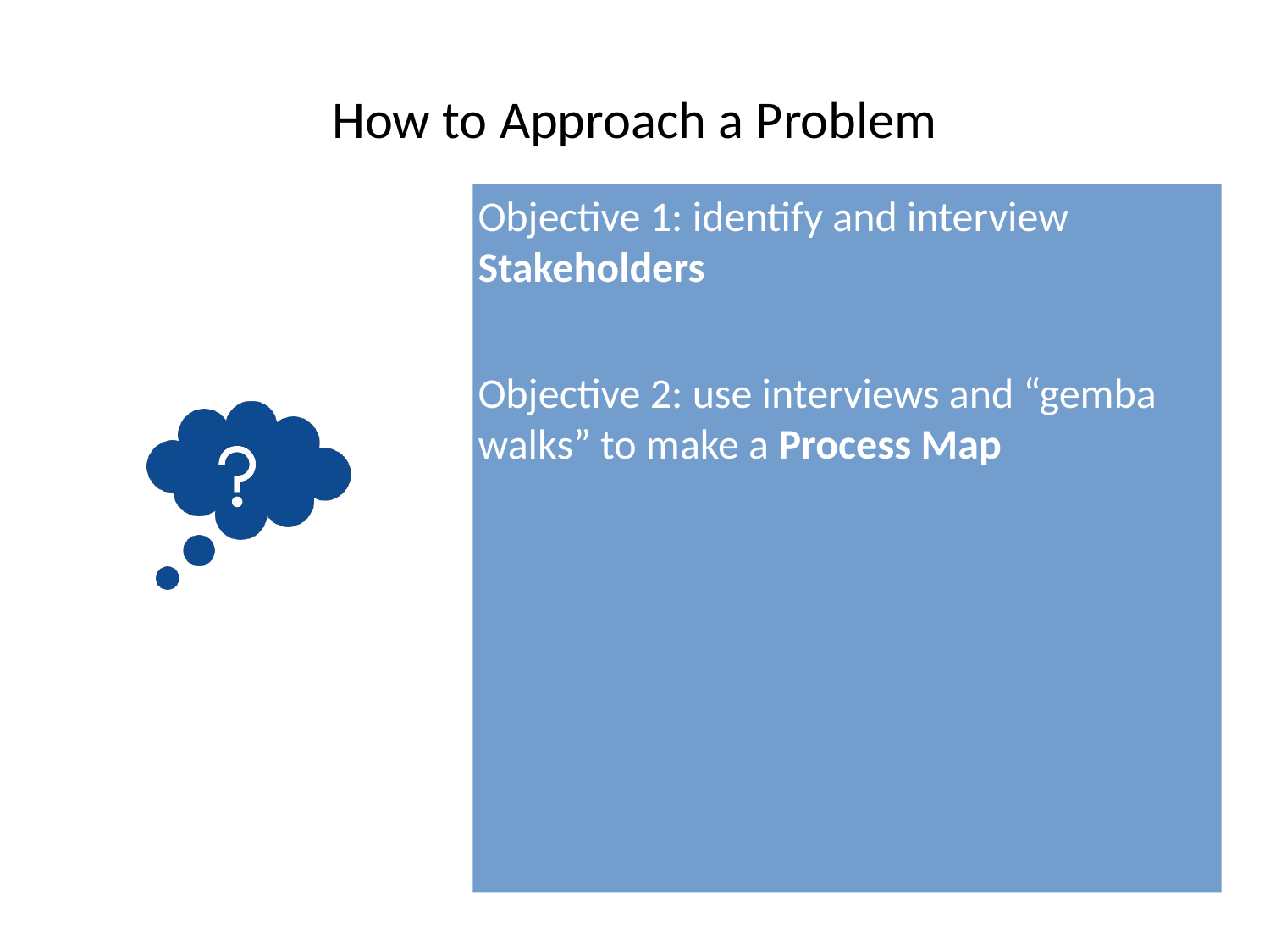

# How to Approach a Problem
Objective 1: identify and interview Stakeholders
Objective 2: use interviews and “gemba walks” to make a Process Map

## Slide 30
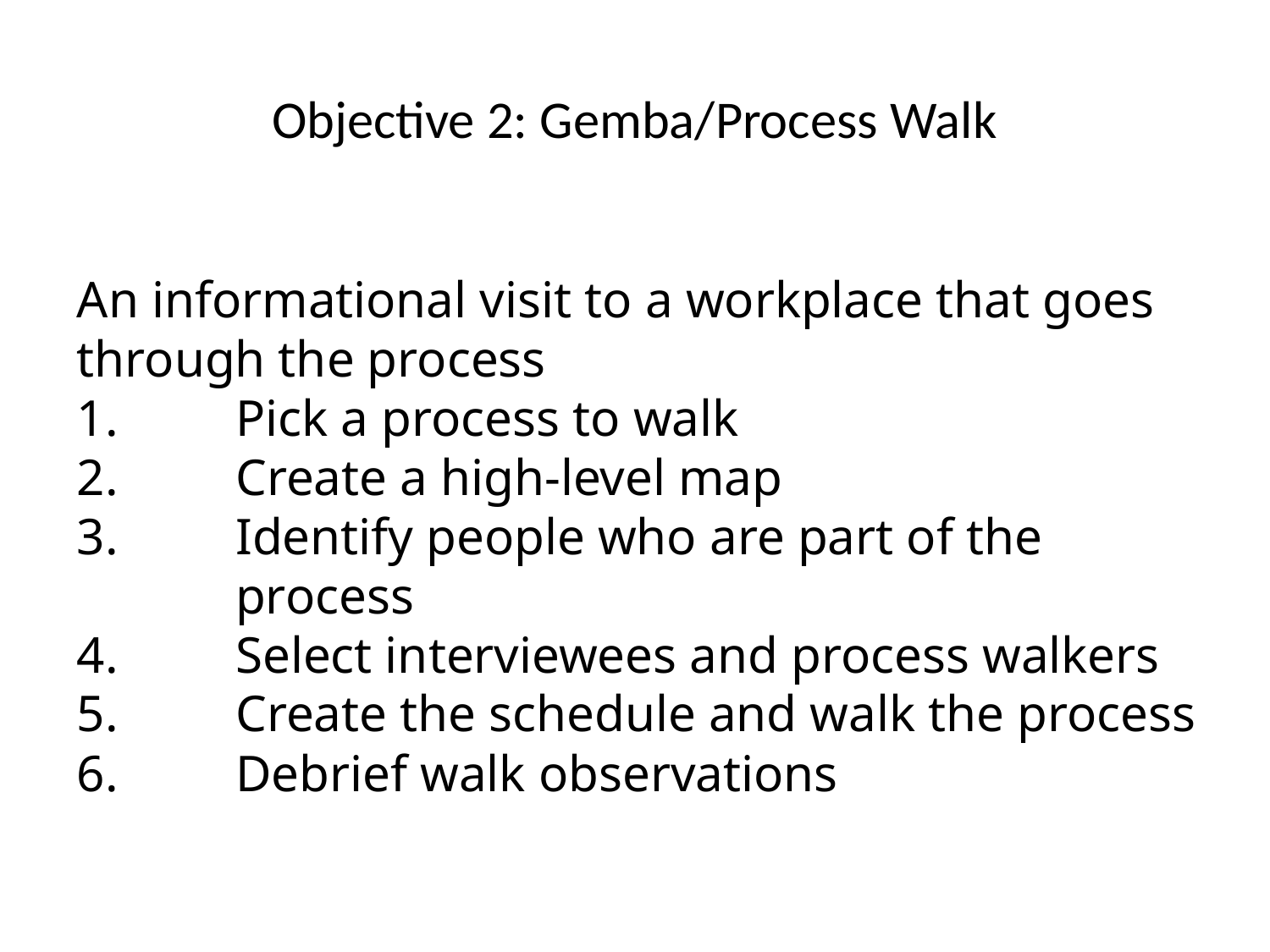

# Objective 2: Gemba/Process Walk
An informational visit to a workplace that goes through the process
Pick a process to walk
Create a high-level map
Identify people who are part of the process
Select interviewees and process walkers
Create the schedule and walk the process
Debrief walk observations

## Slide 31
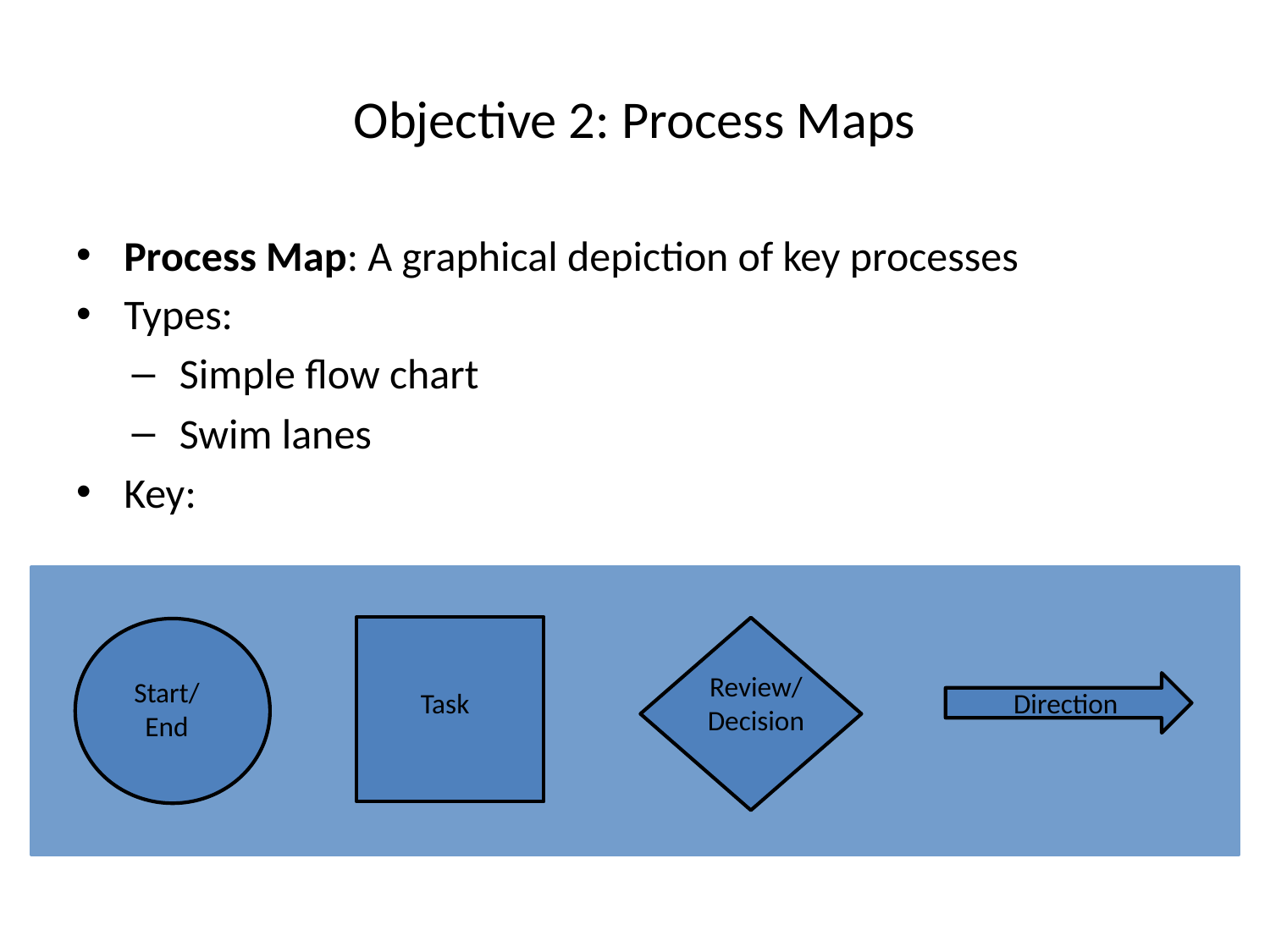

# Objective 2: Process Maps
Process Map: A graphical depiction of key processes
Types:
Simple flow chart
Swim lanes
Key:
Review/
Decision
Start/
End
Task
Direction

## Slide 32
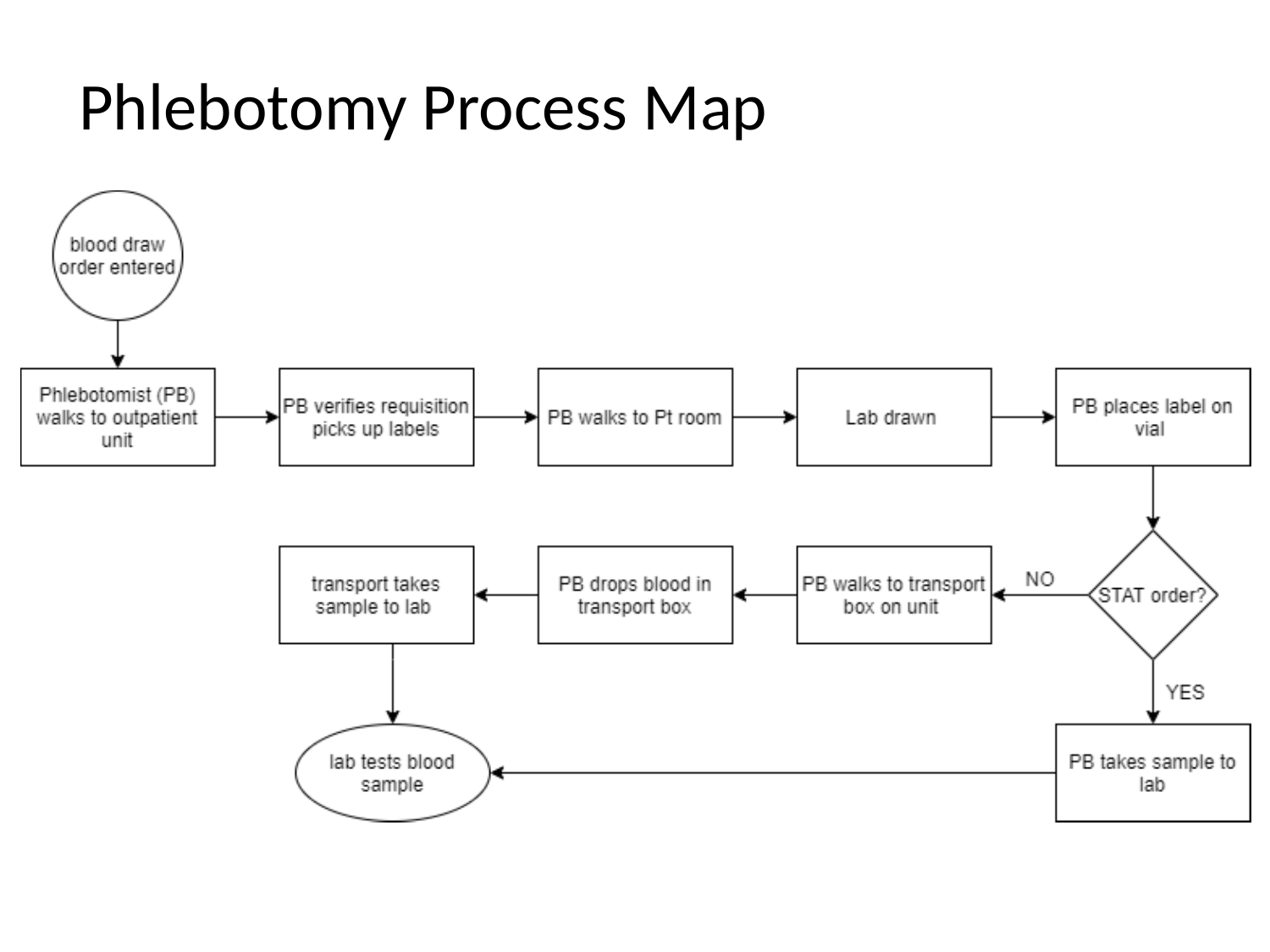

# Phlebotomy Process Map

## Slide 33
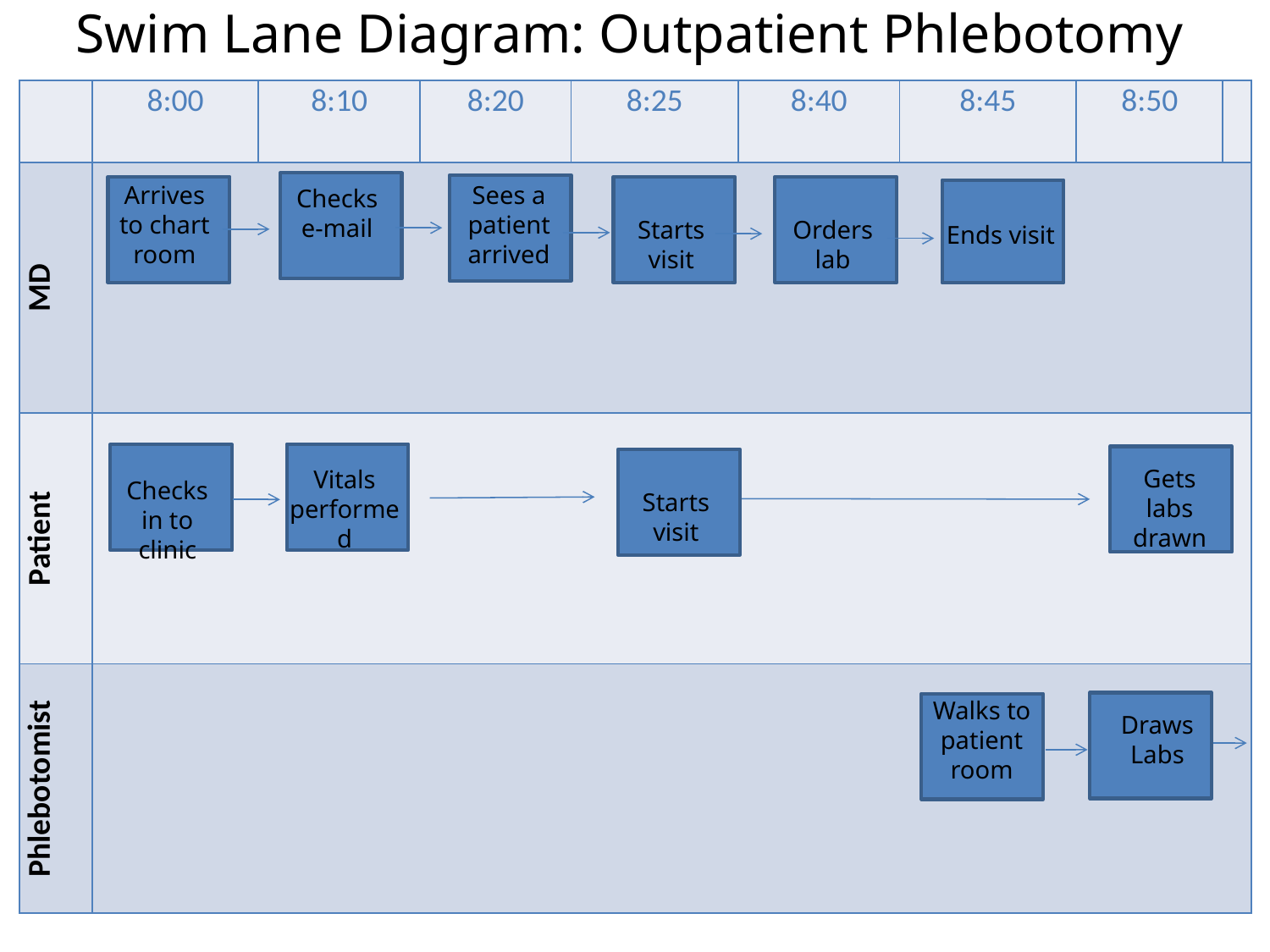

Swim Lane Diagram: Outpatient Phlebotomy
| | 8:00 | 8:10 | 8:20 | 8:25 | 8:40 | 8:45 | 8:50 | |
| --- | --- | --- | --- | --- | --- | --- | --- | --- |
| MD | | | | | | | | |
| Patient | | | | | | | | |
| Phlebotomist | | | | | | | | |
Sees a patient arrived
Arrives to chart room
Checks e-mail
Orders lab
Starts visit
Ends visit
Gets labs drawn
Vitals performed
Checks in to clinic
Starts visit
Walks to patient room
Draws Labs

## Slide 34
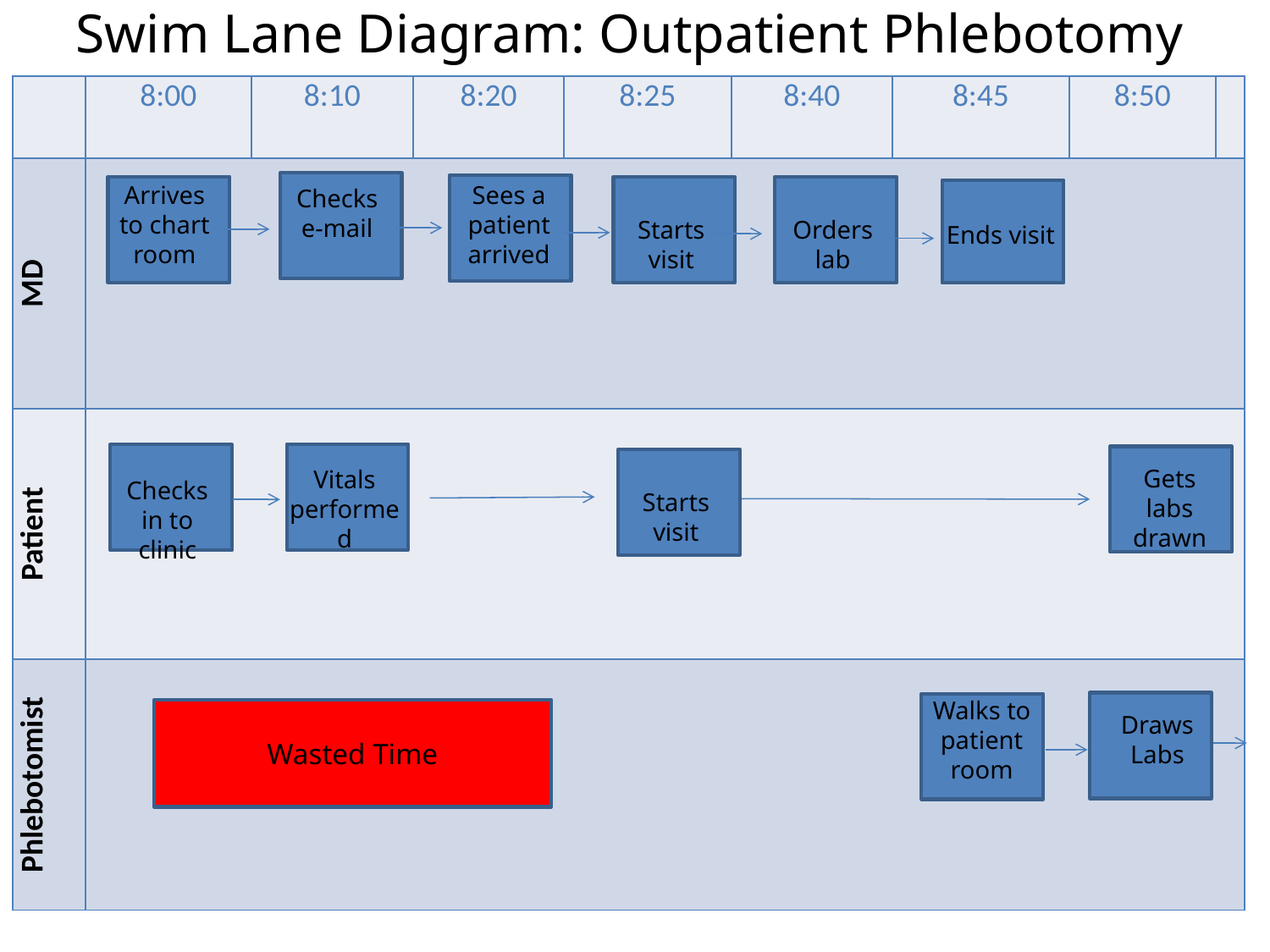

Swim Lane Diagram: Outpatient Phlebotomy
| | 8:00 | 8:10 | 8:20 | 8:25 | 8:40 | 8:45 | 8:50 | |
| --- | --- | --- | --- | --- | --- | --- | --- | --- |
| MD | | | | | | | | |
| Patient | | | | | | | | |
| Phlebotomist | | | | | | | | |
Sees a patient arrived
Arrives to chart room
Checks e-mail
Orders lab
Starts visit
Ends visit
Gets labs drawn
Vitals performed
Checks in to clinic
Starts visit
Walks to patient room
Draws Labs
Wasted Time

## Slide 35
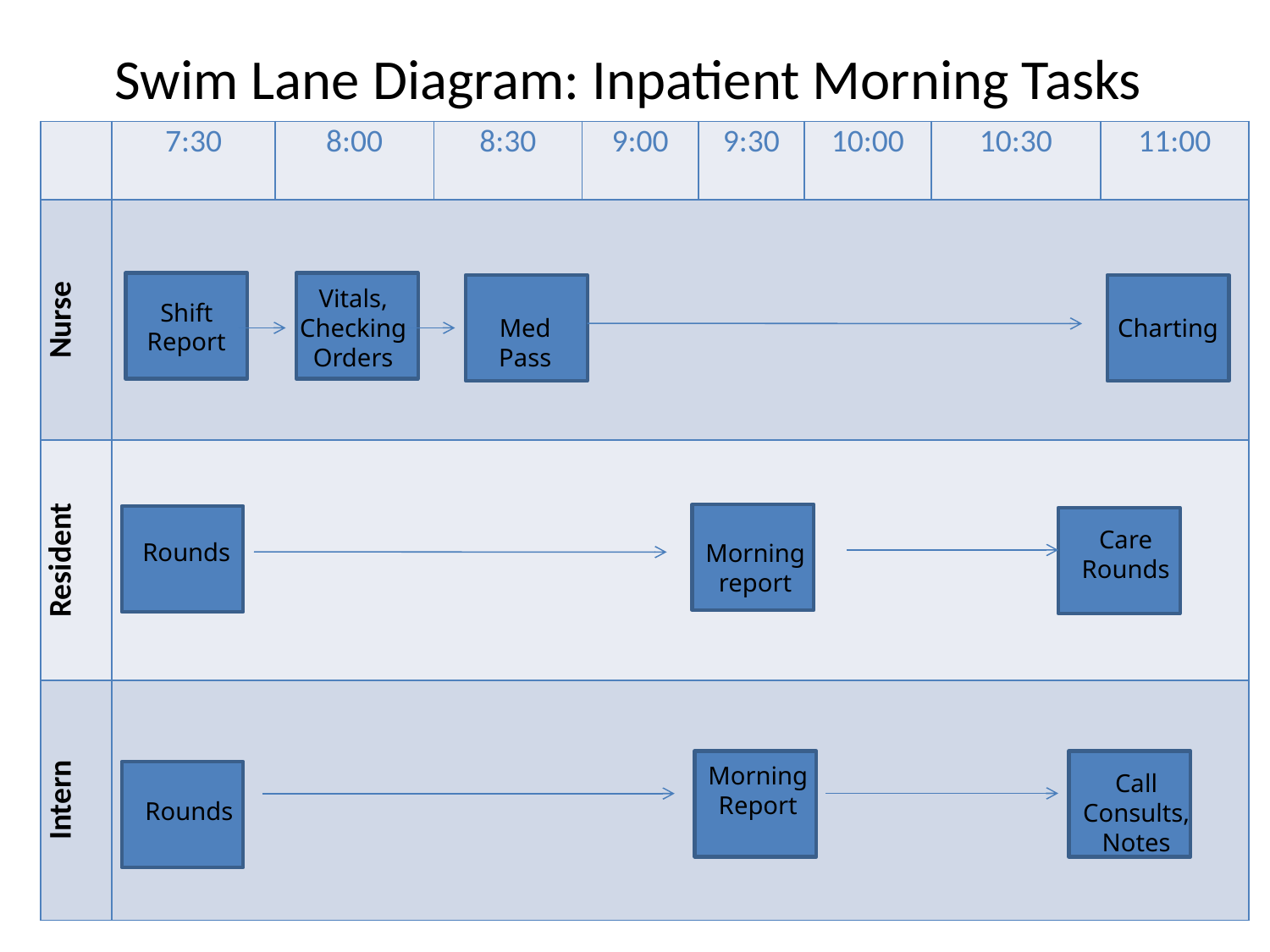

# Swim Lane Diagram: Inpatient Morning Tasks
| | 7:30 | 8:00 | 8:30 | 9:00 | 9:30 | 10:00 | 10:30 | 11:00 |
| --- | --- | --- | --- | --- | --- | --- | --- | --- |
| Nurse | | | | | | | | |
| Resident | | | | | | | | |
| Intern | | | | | | | | |
Vitals, Checking Orders
Shift Report
Med Pass
Charting
Care Rounds
Rounds
Morning report
Morning Report
Call Consults, Notes
Rounds

## Slide 36
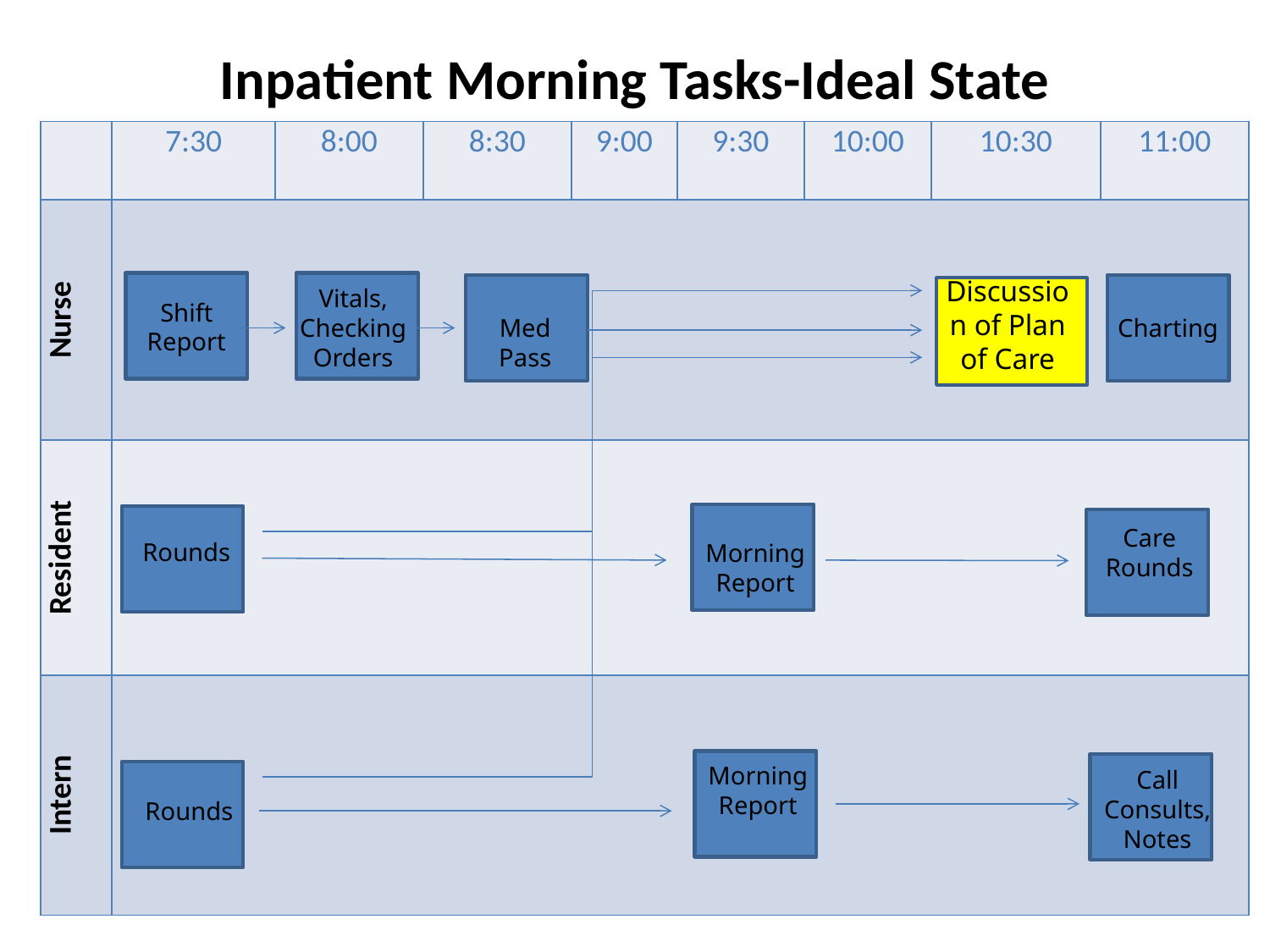

# Inpatient Morning Tasks-Ideal State
| | 7:30 | 8:00 | 8:30 | 9:00 | 9:30 | 10:00 | 10:30 | 11:00 |
| --- | --- | --- | --- | --- | --- | --- | --- | --- |
| Nurse | | | | | | | | |
| Resident | | | | | | | | |
| Intern | | | | | | | | |
Discussion of Plan of Care
Vitals, Checking Orders
Shift Report
Med Pass
Charting
Care Rounds
Rounds
Morning Report
Morning Report
Call Consults, Notes
Rounds

## Slide 37
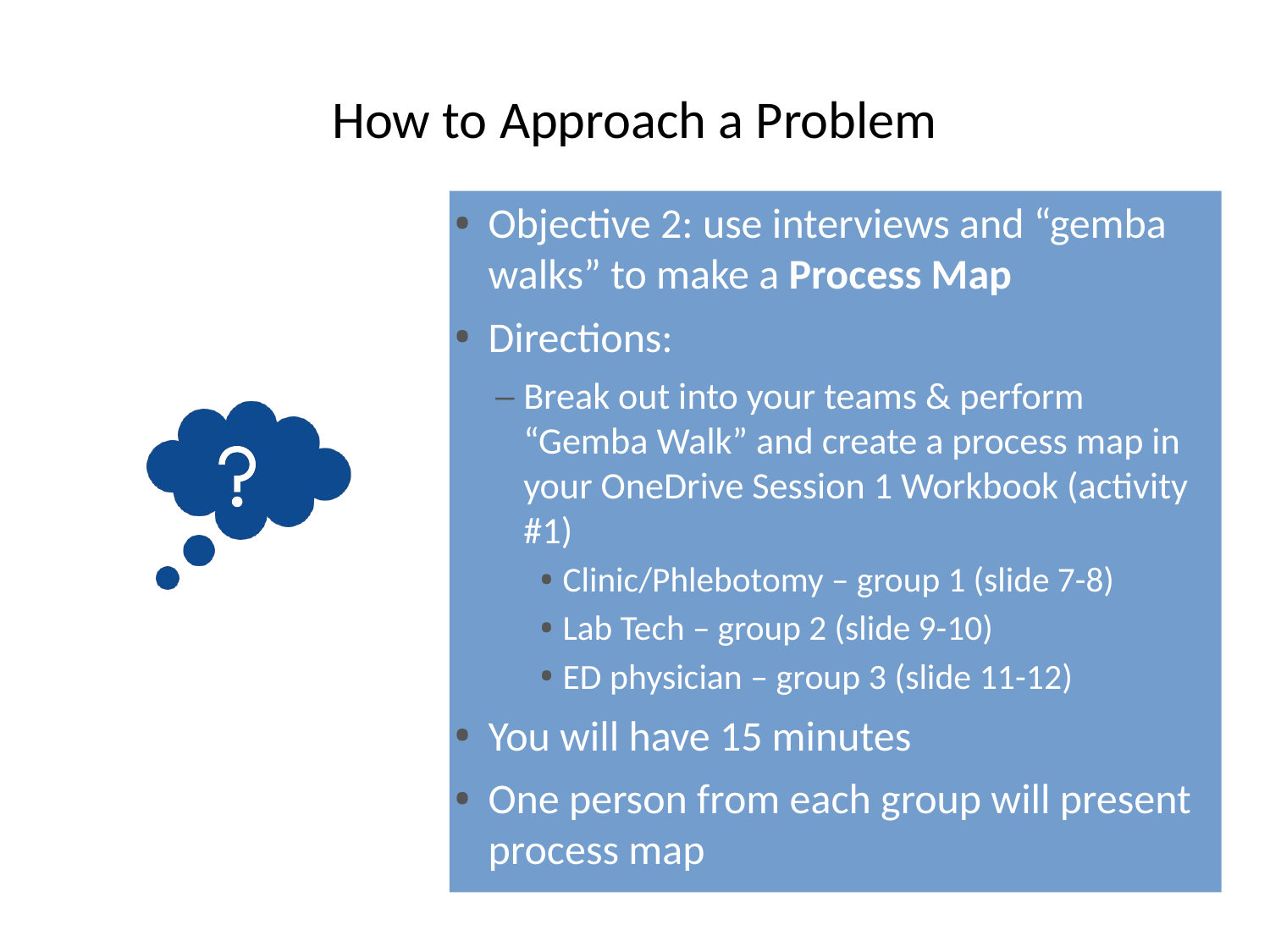

# How to Approach a Problem
Objective 2: use interviews and “gemba walks” to make a Process Map
Directions:
Break out into your teams & perform “Gemba Walk” and create a process map in your OneDrive Session 1 Workbook (activity #1)
Clinic/Phlebotomy – group 1 (slide 7-8)
Lab Tech – group 2 (slide 9-10)
ED physician – group 3 (slide 11-12)
You will have 15 minutes
One person from each group will present process map

## Slide 38
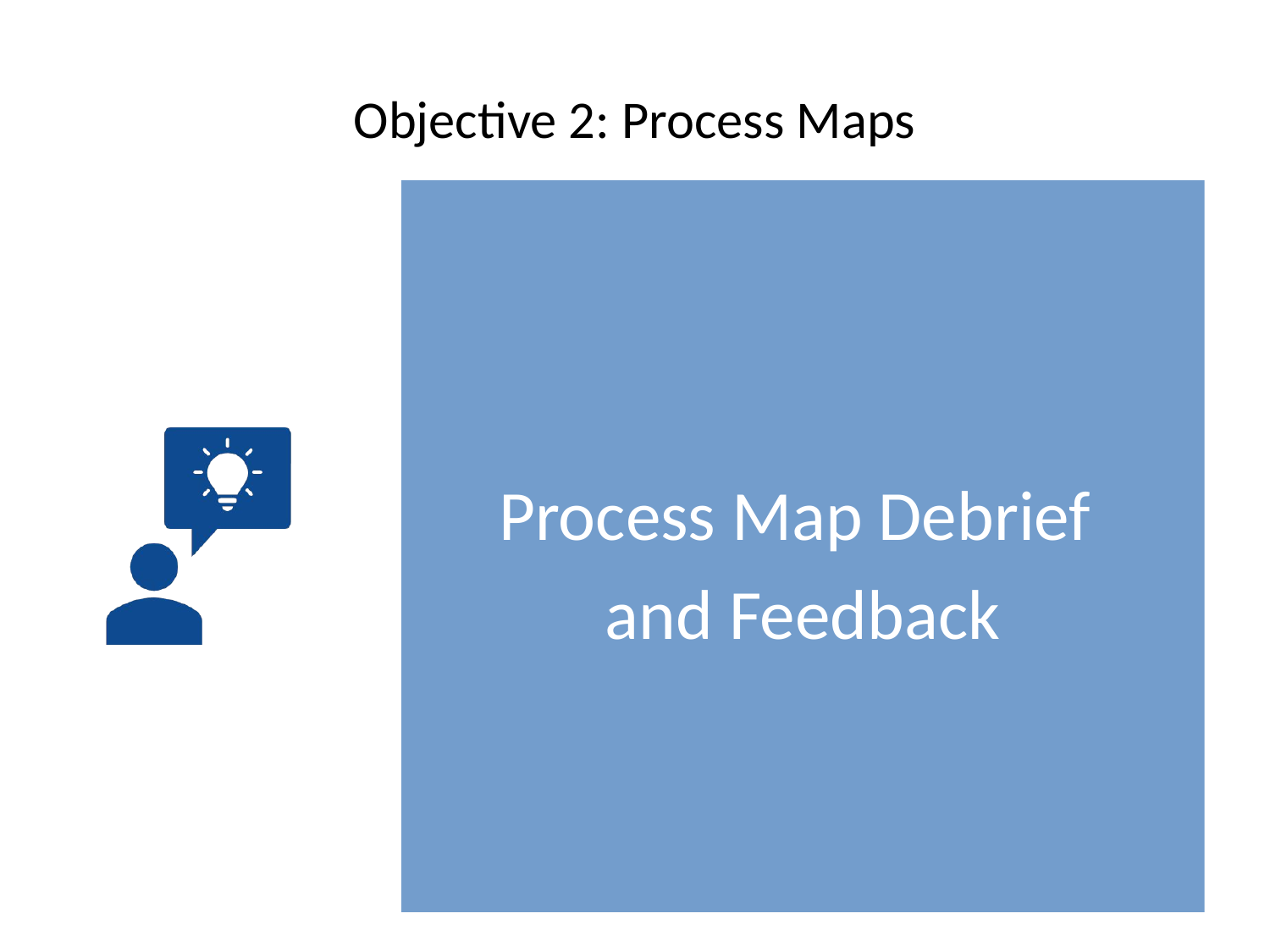

# Objective 2: Process Maps
Process Map Debrief
and Feedback

## Slide 39
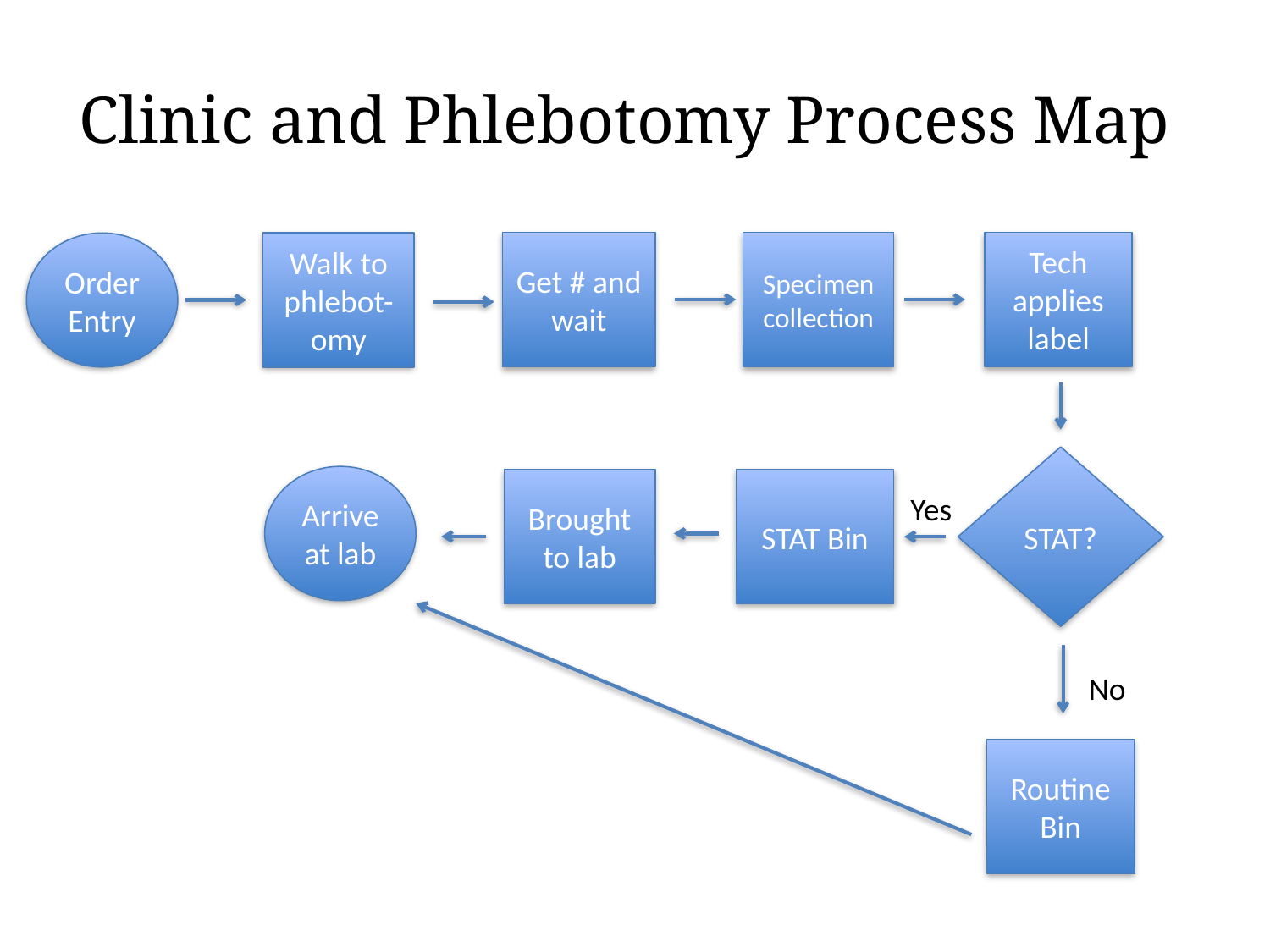

# Clinic and Phlebotomy Process Map
Get # and wait
Specimen collection
Tech applies label
Walk to phlebot-omy
Order Entry
STAT?
Arrive at lab
Brought to lab
STAT Bin
Yes
No
Routine Bin

## Slide 40
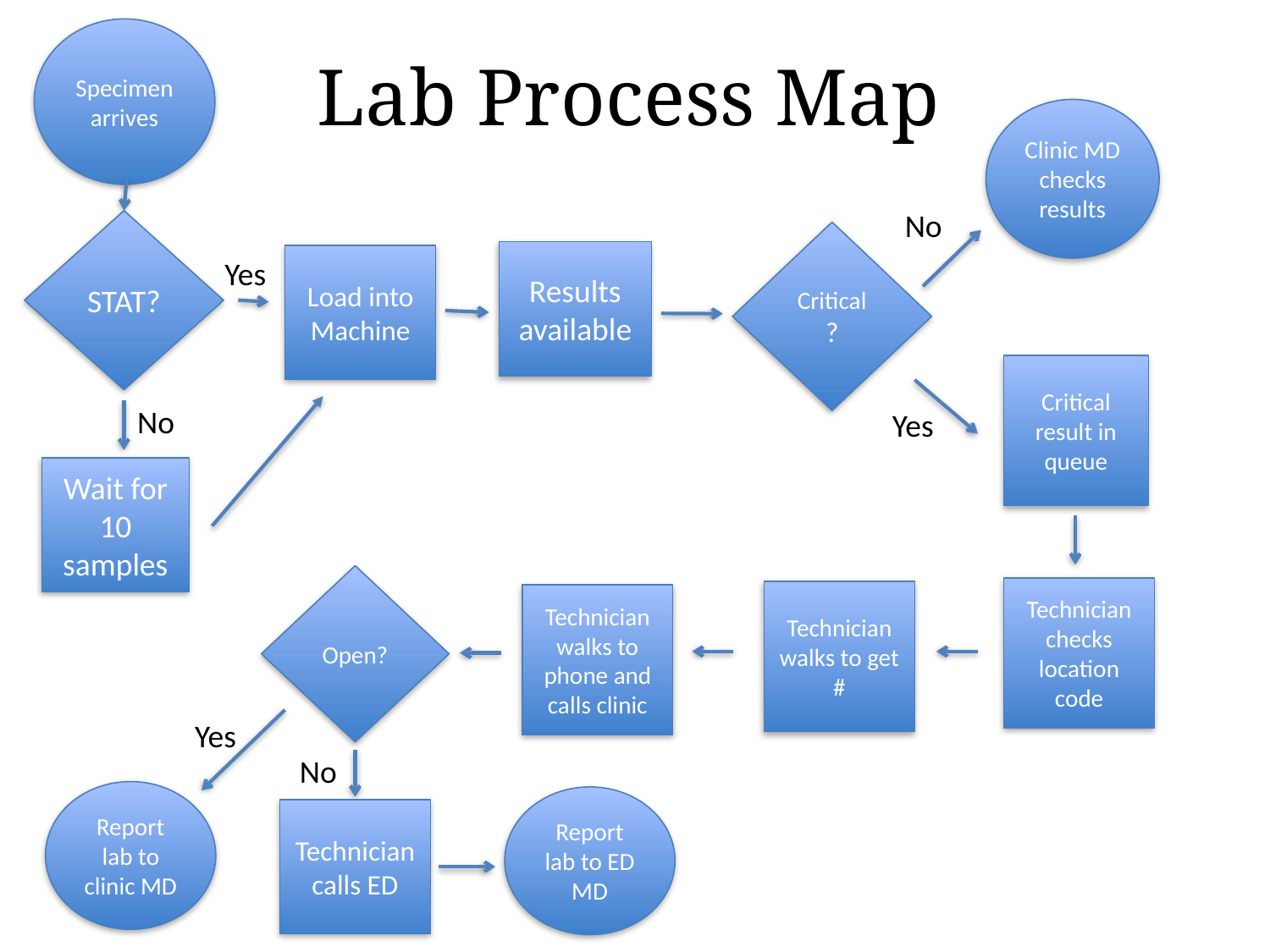

# Lab Process Map
Specimen arrives
Clinic MD checks results
No
STAT?
Critical?
Results available
Load into Machine
Yes
Critical result in queue
No
Yes
Wait for 10 samples
Open?
Technician checks location code
Technician walks to get #
Technician walks to phone and calls clinic
Yes
No
Report lab to clinic MD
Report lab to ED MD
Technician calls ED

## Slide 41
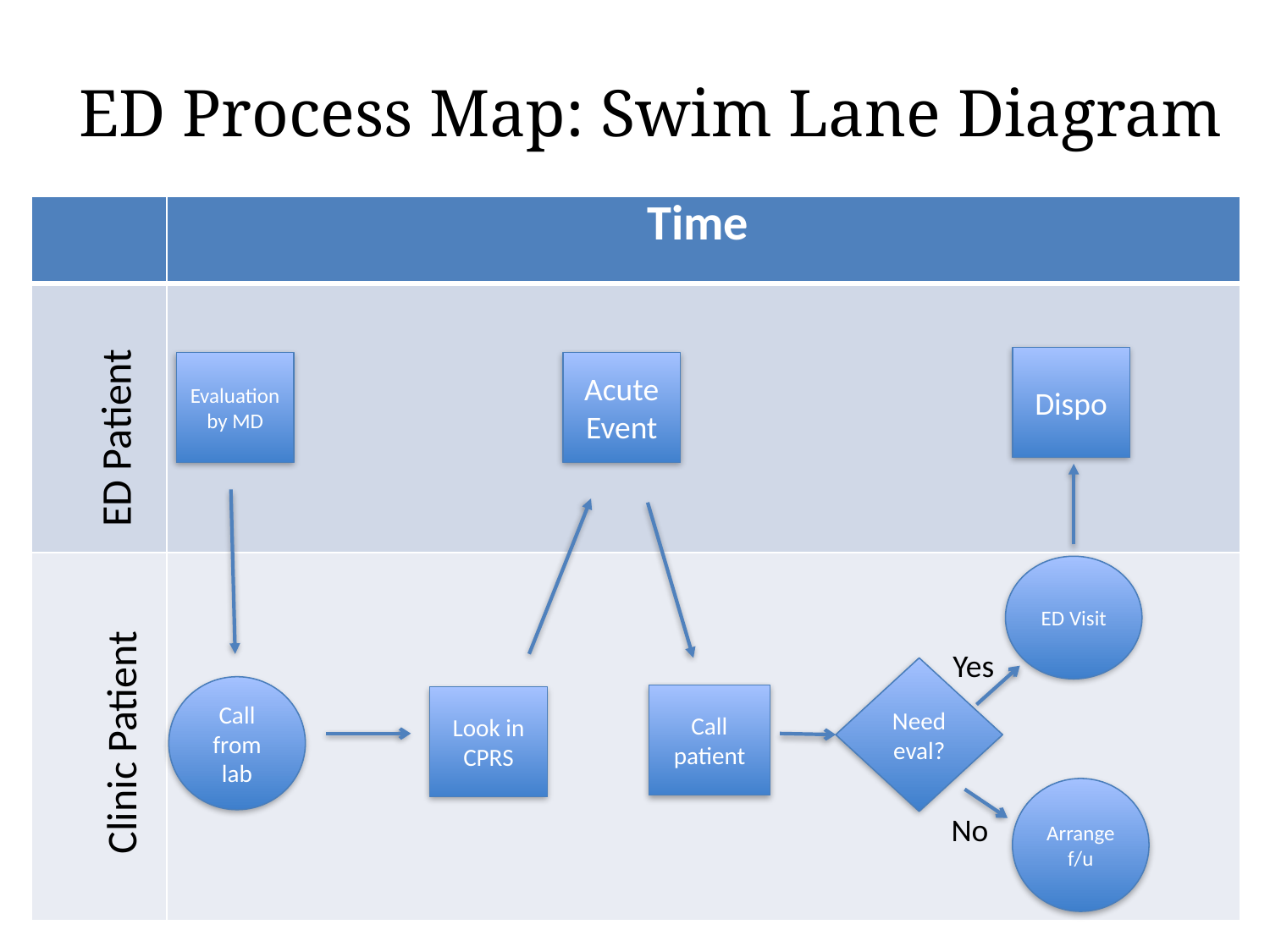

# ED Process Map: Swim Lane Diagram
| | Time |
| --- | --- |
| | |
| | |
Dispo
Acute Event
Evaluation by MD
ED Patient
ED Visit
Yes
Need eval?
Call from lab
Call patient
Look in CPRS
Clinic Patient
Arrange f/u
No

## Slide 42
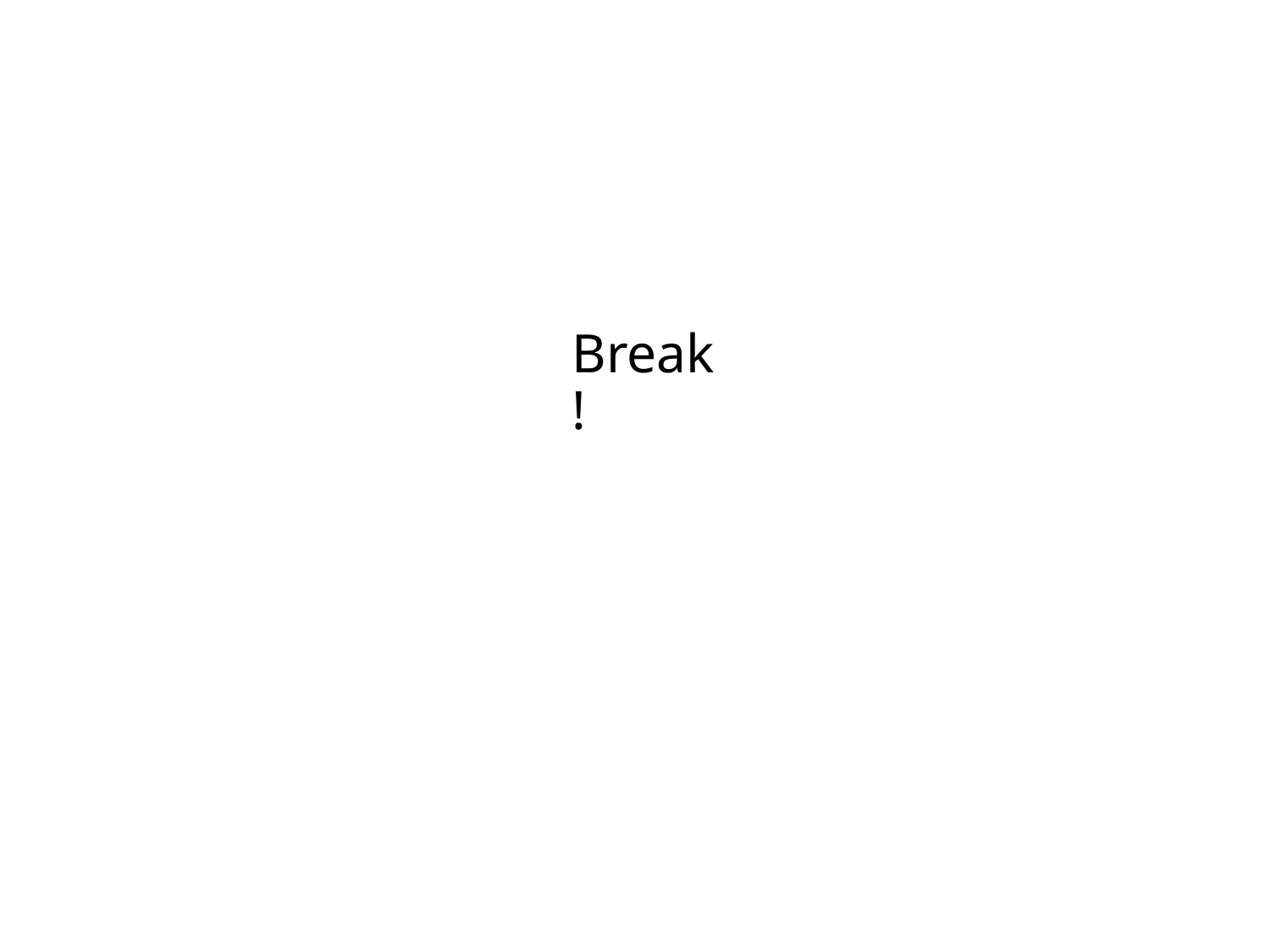

# Break!

## Slide 43
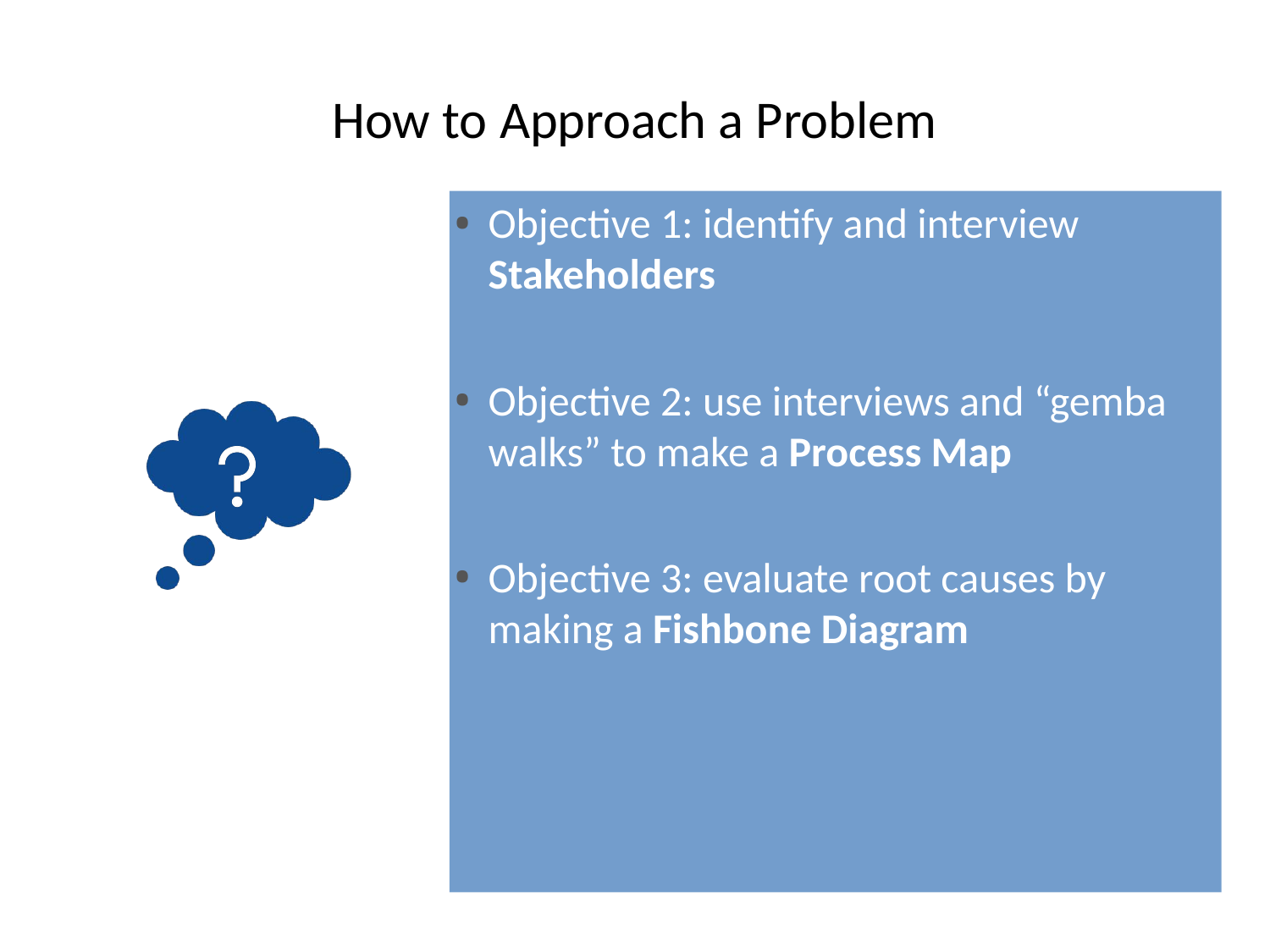

# How to Approach a Problem
Objective 1: identify and interview Stakeholders
Objective 2: use interviews and “gemba walks” to make a Process Map
Objective 3: evaluate root causes by making a Fishbone Diagram

## Slide 44
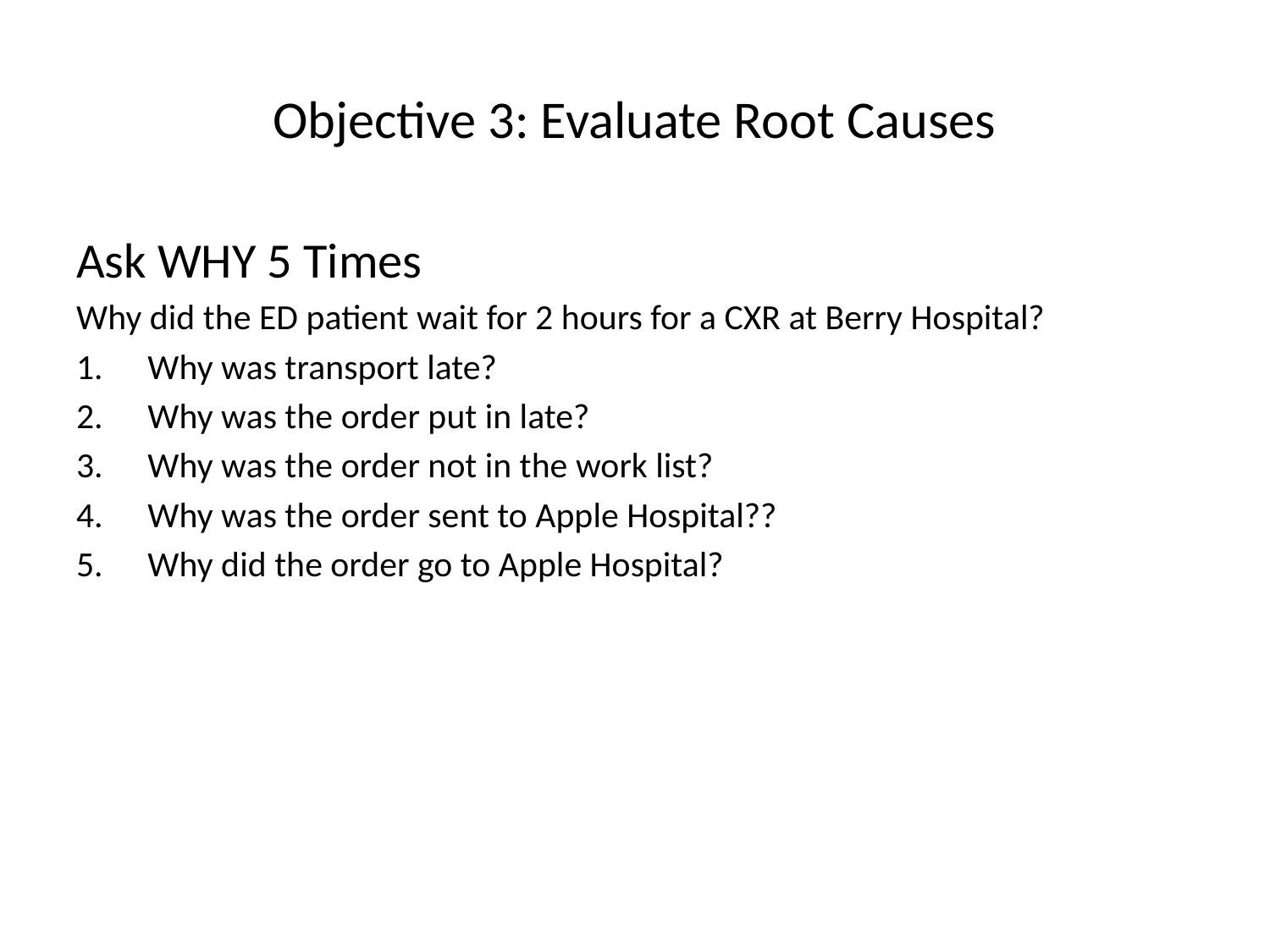

# Objective 3: Evaluate Root Causes
Ask WHY 5 Times
Why did the ED patient wait for 2 hours for a CXR at Berry Hospital?
Why was transport late?
Why was the order put in late?
Why was the order not in the work list?
Why was the order sent to Apple Hospital??
Why did the order go to Apple Hospital?

## Slide 45
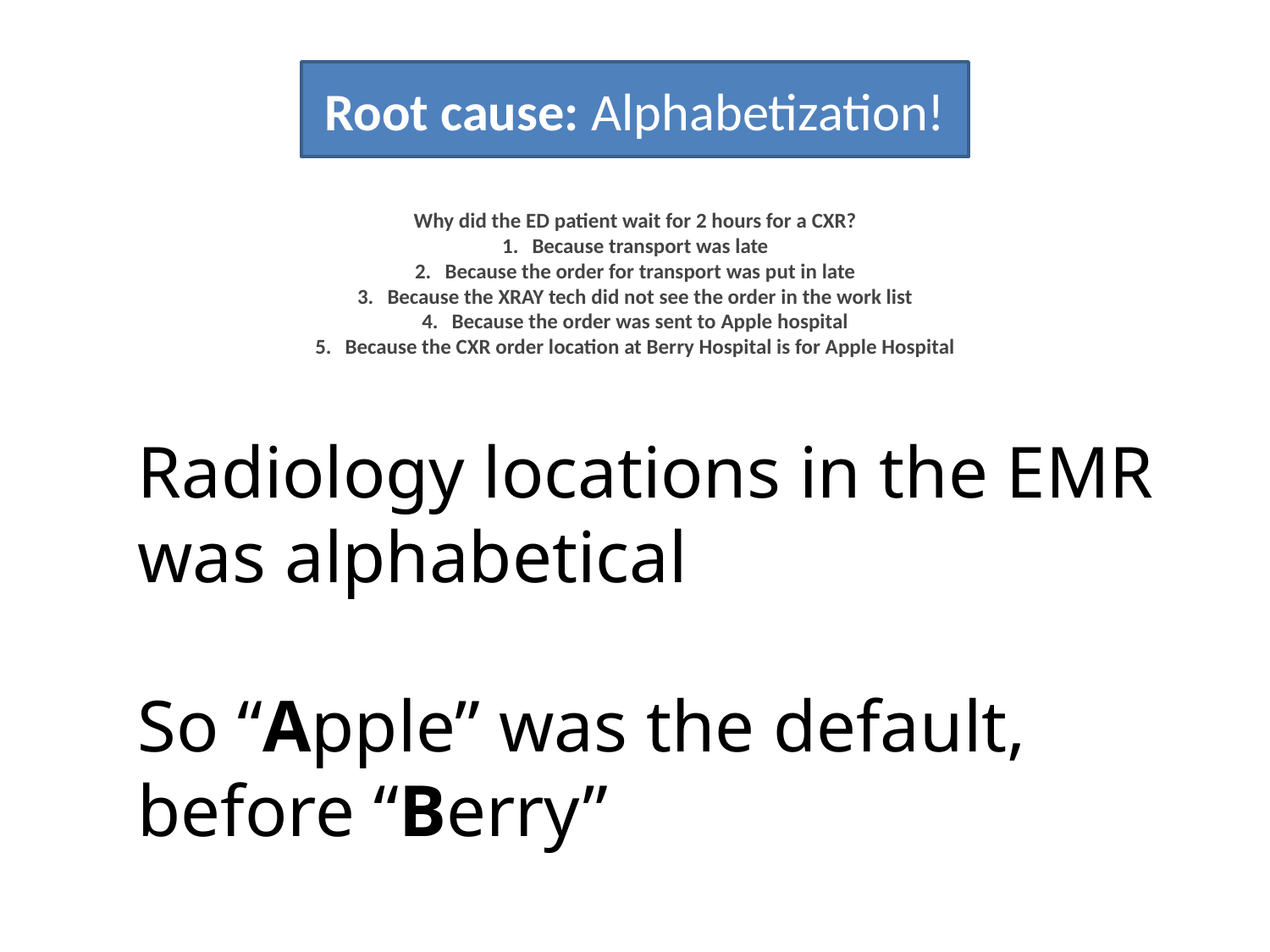

Root cause: Alphabetization!
Why did the ED patient wait for 2 hours for a CXR?​
Because transport was late​
Because the order for transport was put in late​
Because the XRAY tech did not see the order in the work list​
Because the order was sent to Apple hospital
Because the CXR order location at Berry Hospital is for Apple Hospital​
Radiology locations in the EMR was alphabetical
So “Apple” was the default, before “Berry”

## Slide 46
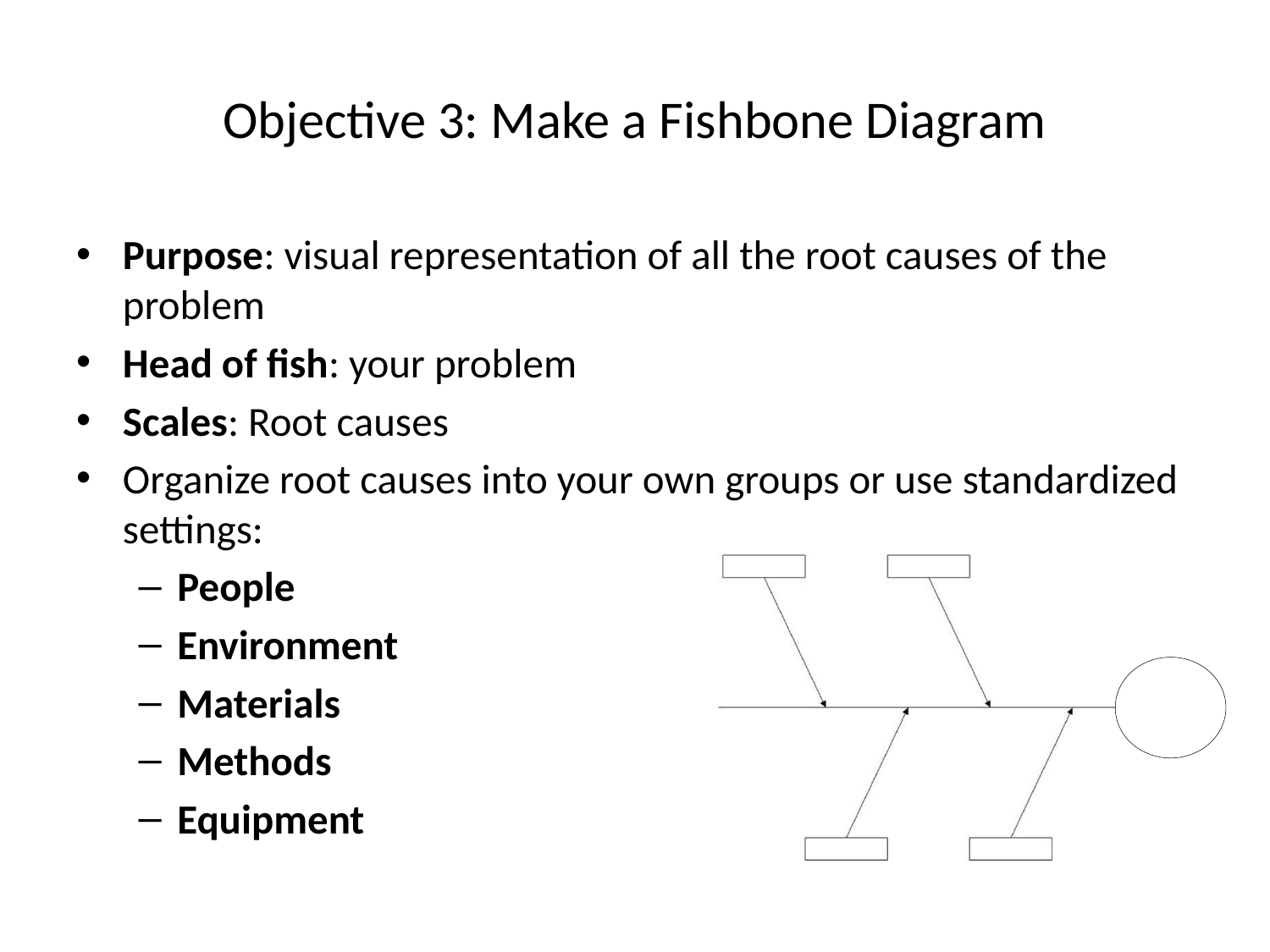

# Objective 3: Make a Fishbone Diagram
Purpose: visual representation of all the root causes of the problem
Head of fish: your problem
Scales: Root causes
Organize root causes into your own groups or use standardized settings:
People
Environment
Materials
Methods
Equipment

## Slide 47
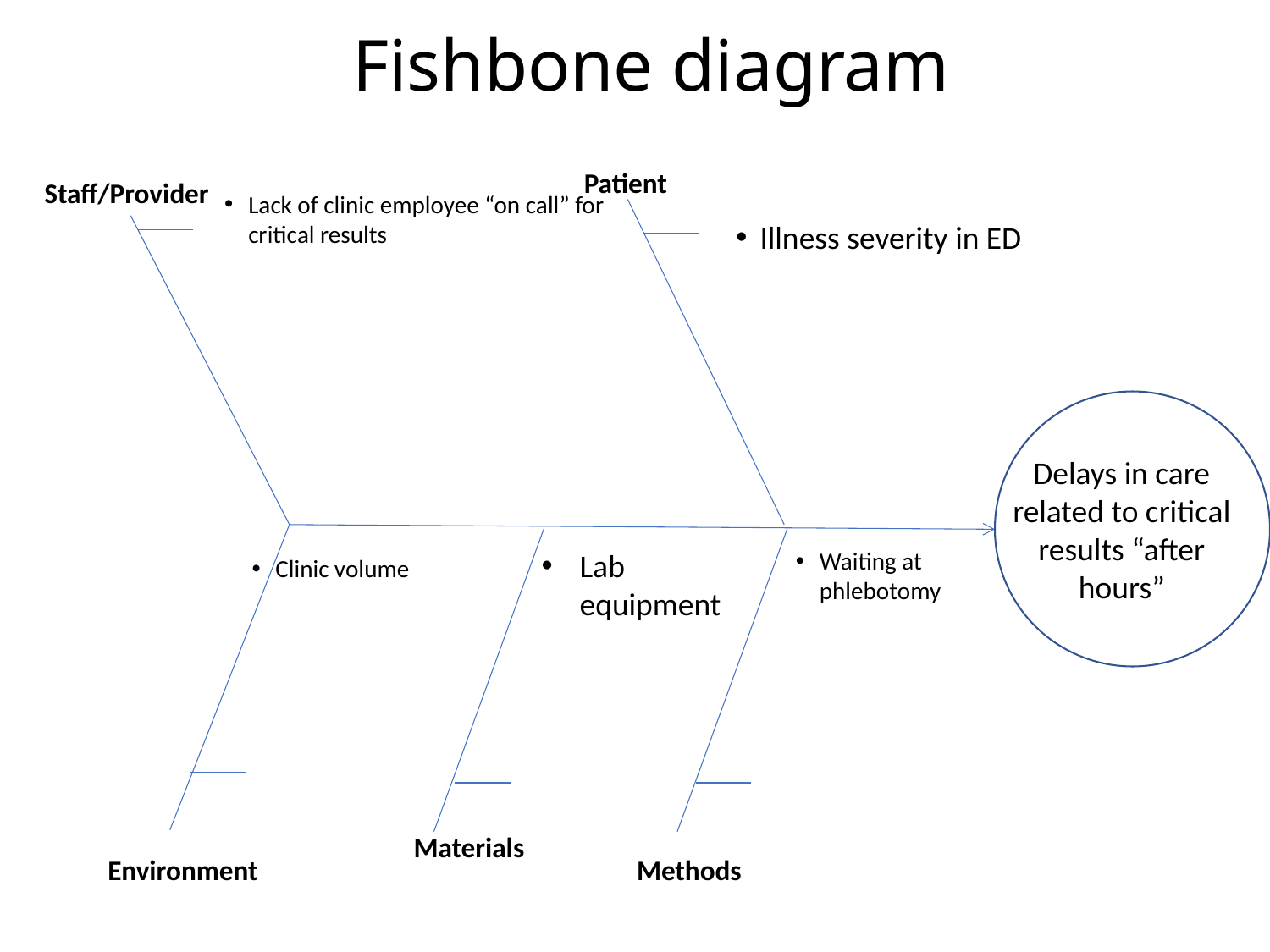

# Fishbone diagram
Patient
Staff/Provider
Lack of clinic employee “on call” for critical results
Illness severity in ED
Delays in care related to critical results “after hours”
Lab equipment
Waiting at phlebotomy
Clinic volume
Materials
Environment
Methods

## Slide 48
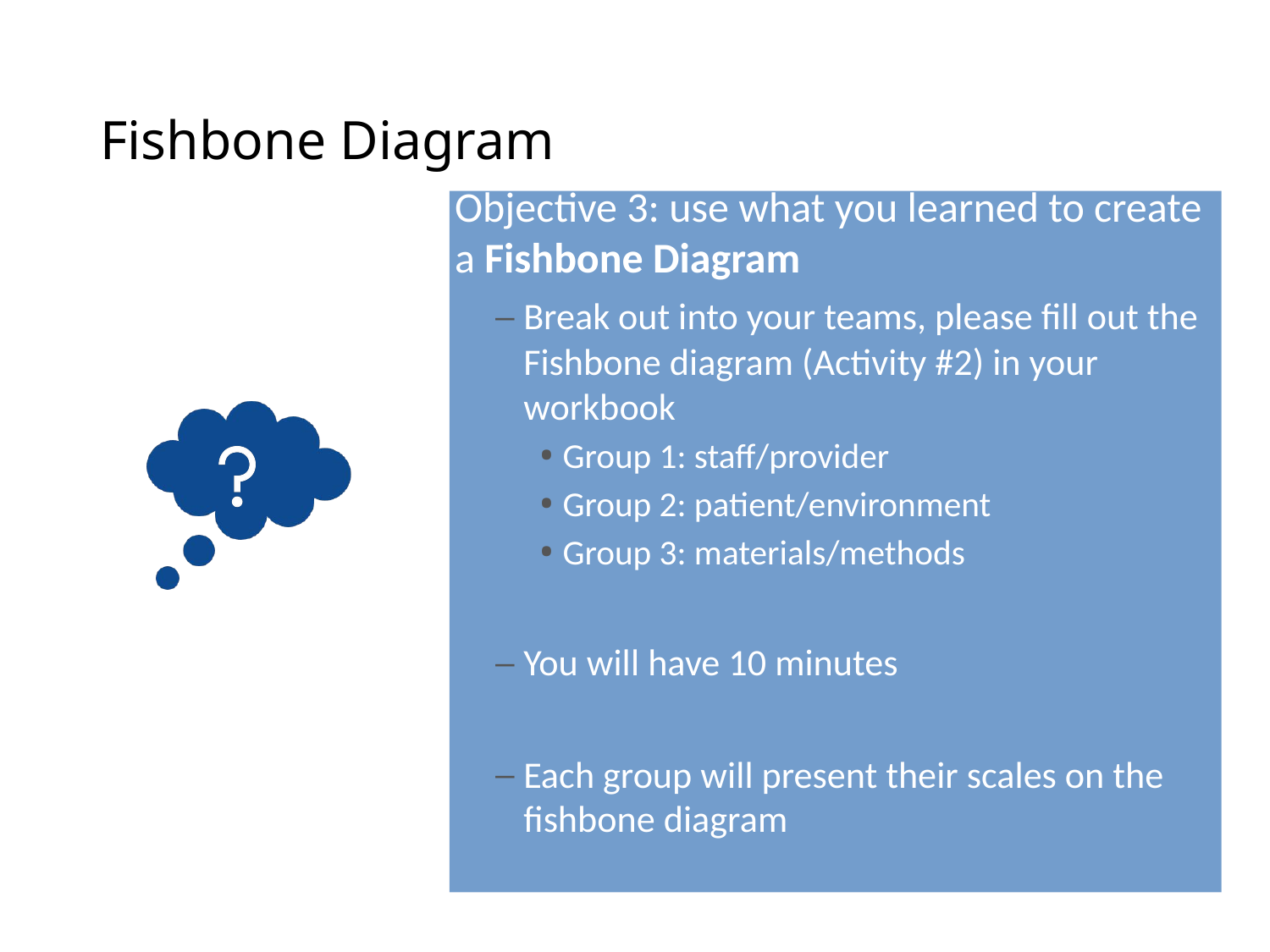

# Fishbone Diagram
Objective 3: use what you learned to create a Fishbone Diagram
Break out into your teams, please fill out the Fishbone diagram (Activity #2) in your workbook
Group 1: staff/provider
Group 2: patient/environment
Group 3: materials/methods
You will have 10 minutes
Each group will present their scales on the fishbone diagram

## Slide 49
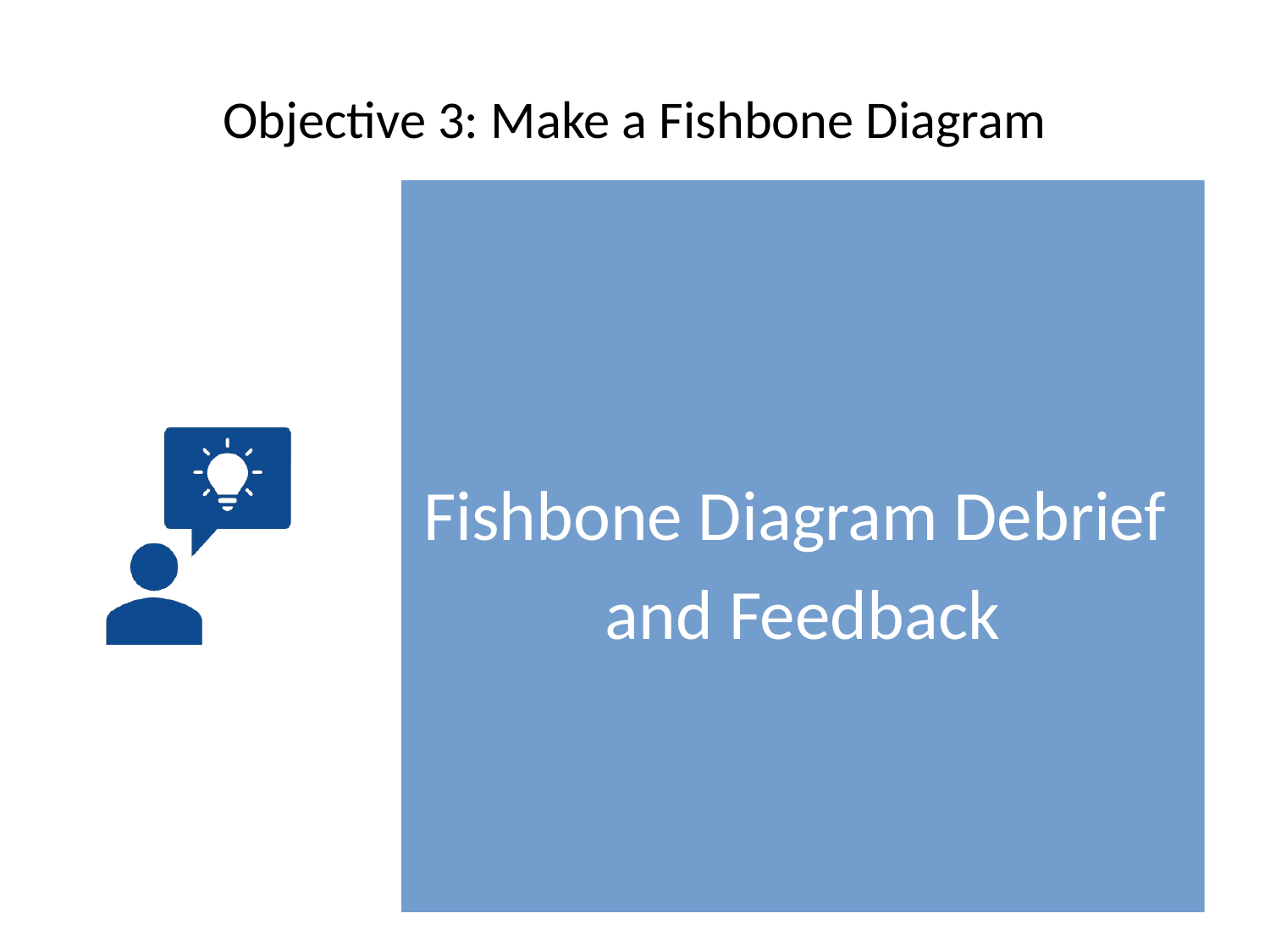

# Objective 3: Make a Fishbone Diagram
Fishbone Diagram Debrief
and Feedback

## Slide 50
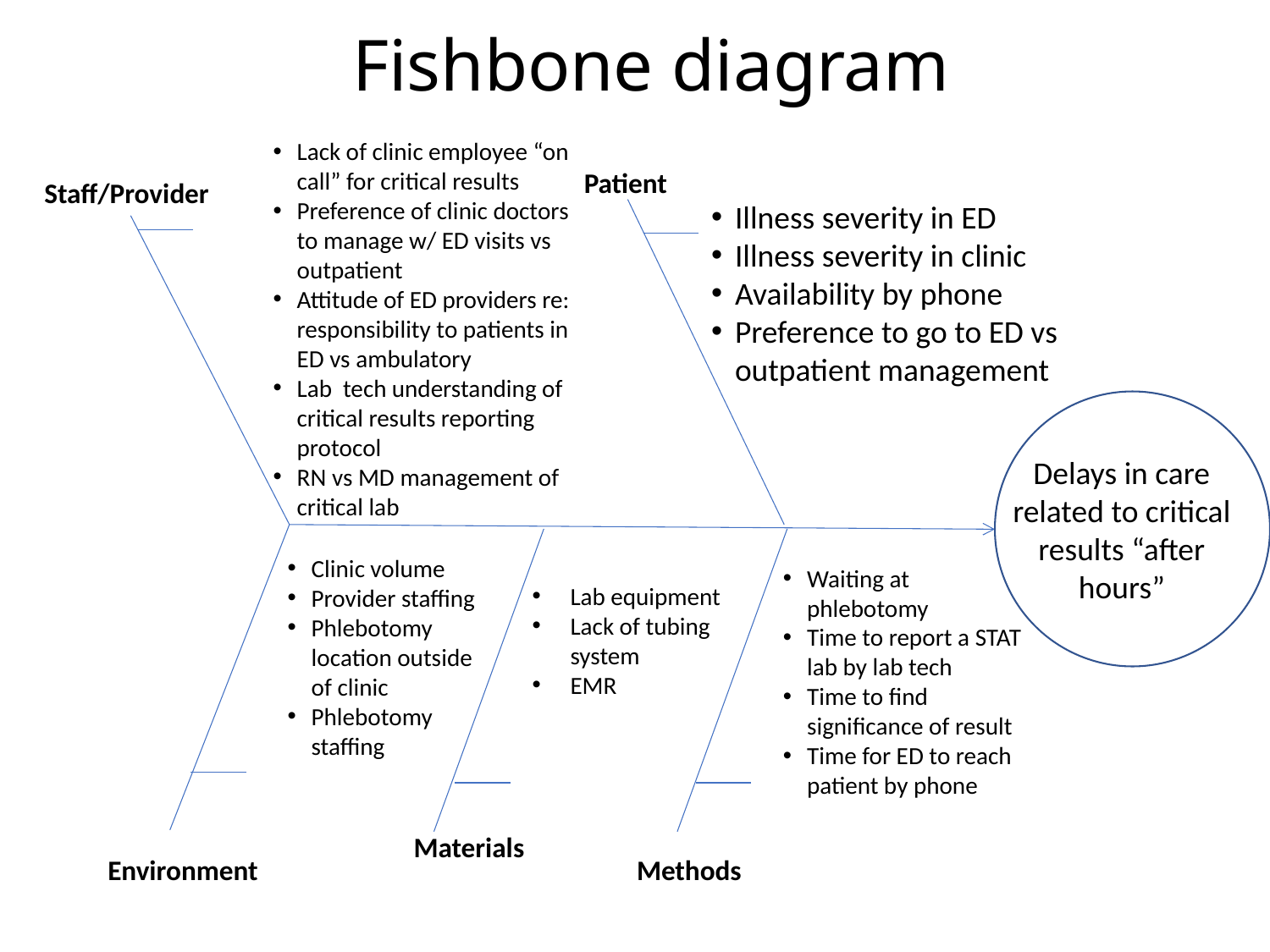

# Fishbone diagram
Lack of clinic employee “on call” for critical results
Preference of clinic doctors to manage w/ ED visits vs outpatient
Attitude of ED providers re: responsibility to patients in ED vs ambulatory
Lab tech understanding of critical results reporting protocol
RN vs MD management of critical lab
Patient
Staff/Provider
Illness severity in ED
Illness severity in clinic
Availability by phone
Preference to go to ED vs outpatient management
Delays in care related to critical results “after hours”
Clinic volume
Provider staffing
Phlebotomy location outside of clinic
Phlebotomy staffing
Waiting at phlebotomy
Time to report a STAT lab by lab tech
Time to find significance of result
Time for ED to reach patient by phone
Lab equipment
Lack of tubing system
EMR
Materials
Environment
Methods

## Slide 51
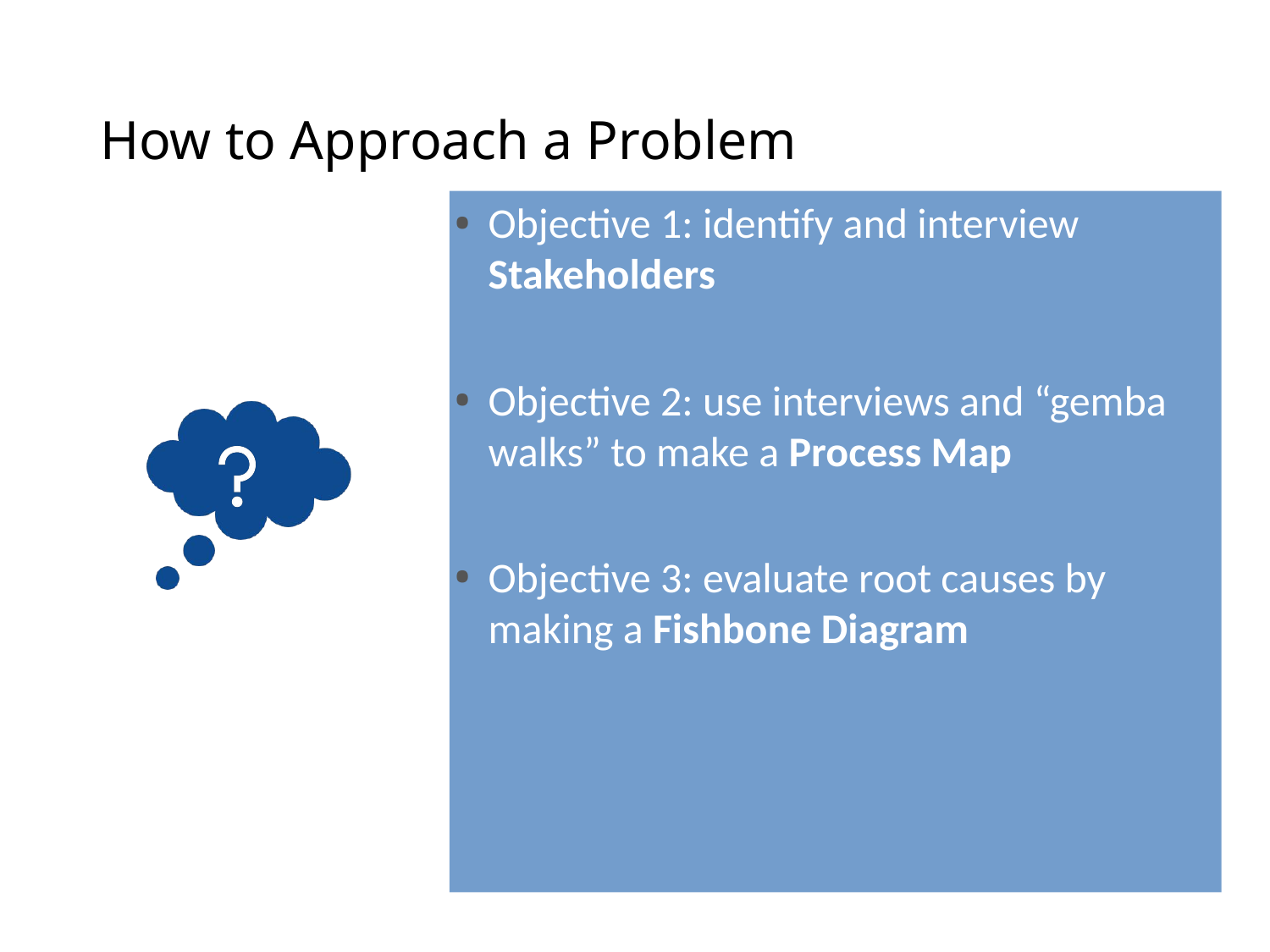

# How to Approach a Problem
Objective 1: identify and interview Stakeholders
Objective 2: use interviews and “gemba walks” to make a Process Map
Objective 3: evaluate root causes by making a Fishbone Diagram

## Slide 52
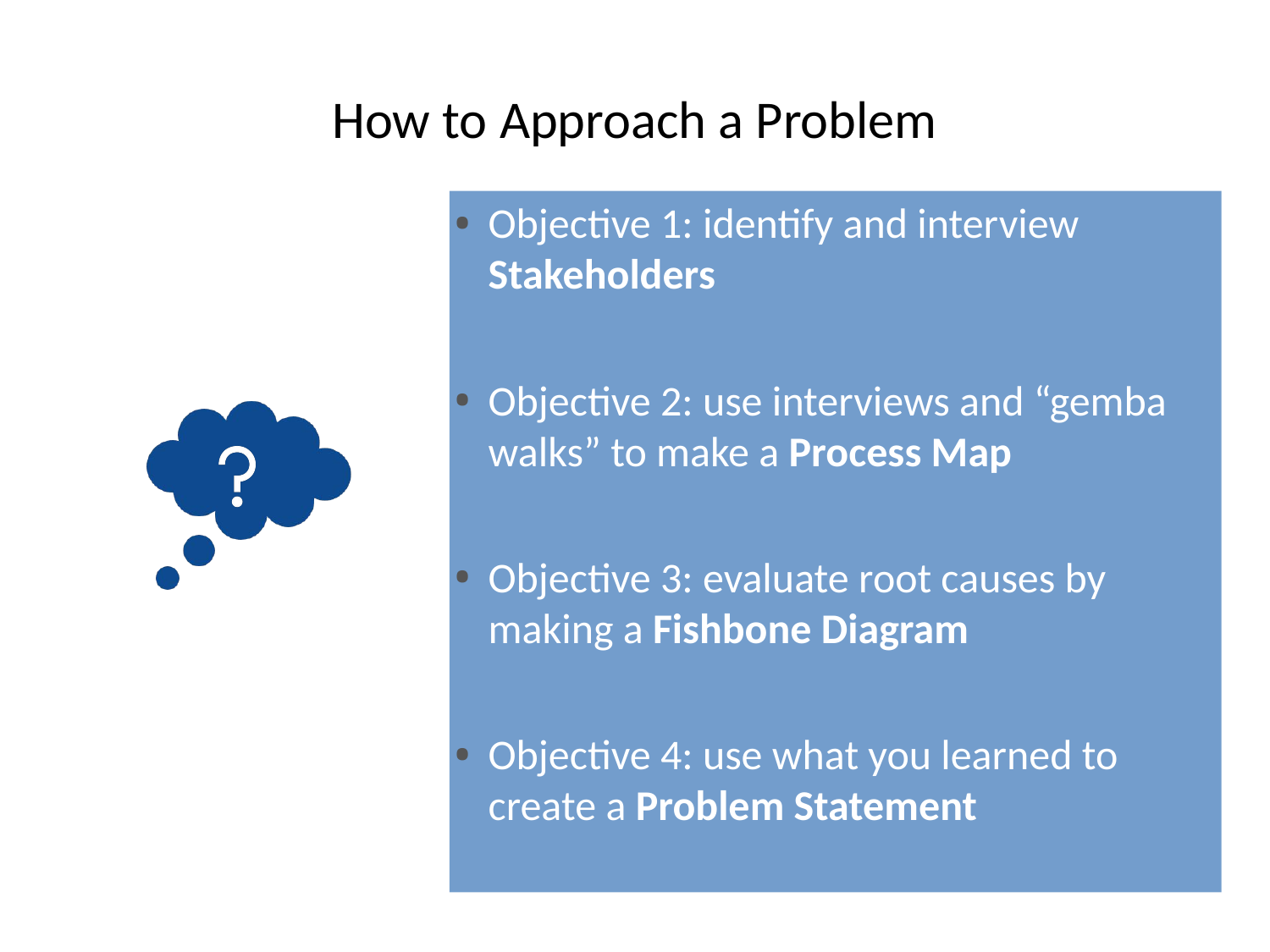

# How to Approach a Problem
Objective 1: identify and interview Stakeholders
Objective 2: use interviews and “gemba walks” to make a Process Map
Objective 3: evaluate root causes by making a Fishbone Diagram
Objective 4: use what you learned to create a Problem Statement

## Slide 53
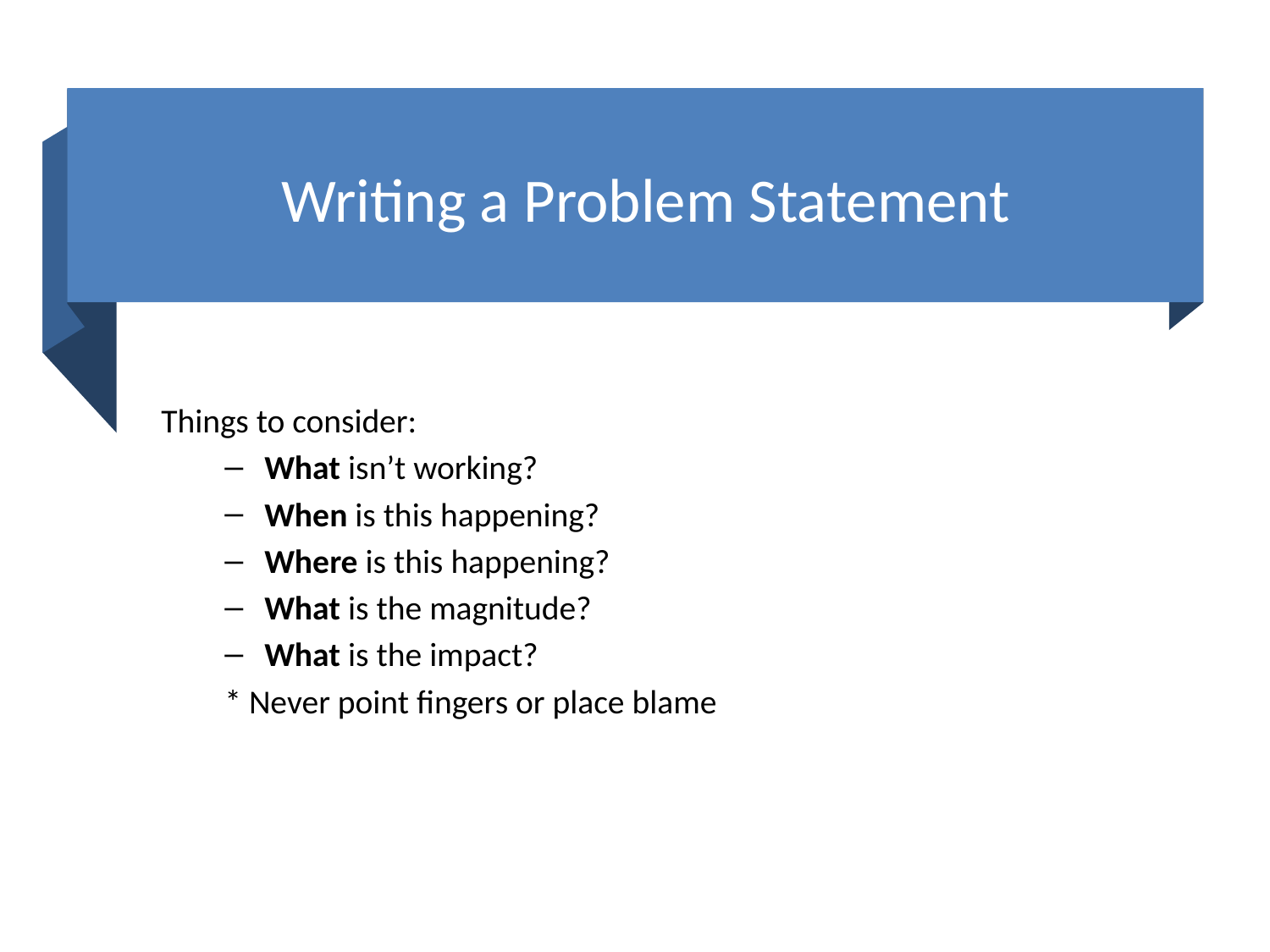

# Writing a Problem Statement
Things to consider:
What isn’t working?
When is this happening?
Where is this happening?
What is the magnitude?
What is the impact?
* Never point fingers or place blame

## Slide 54
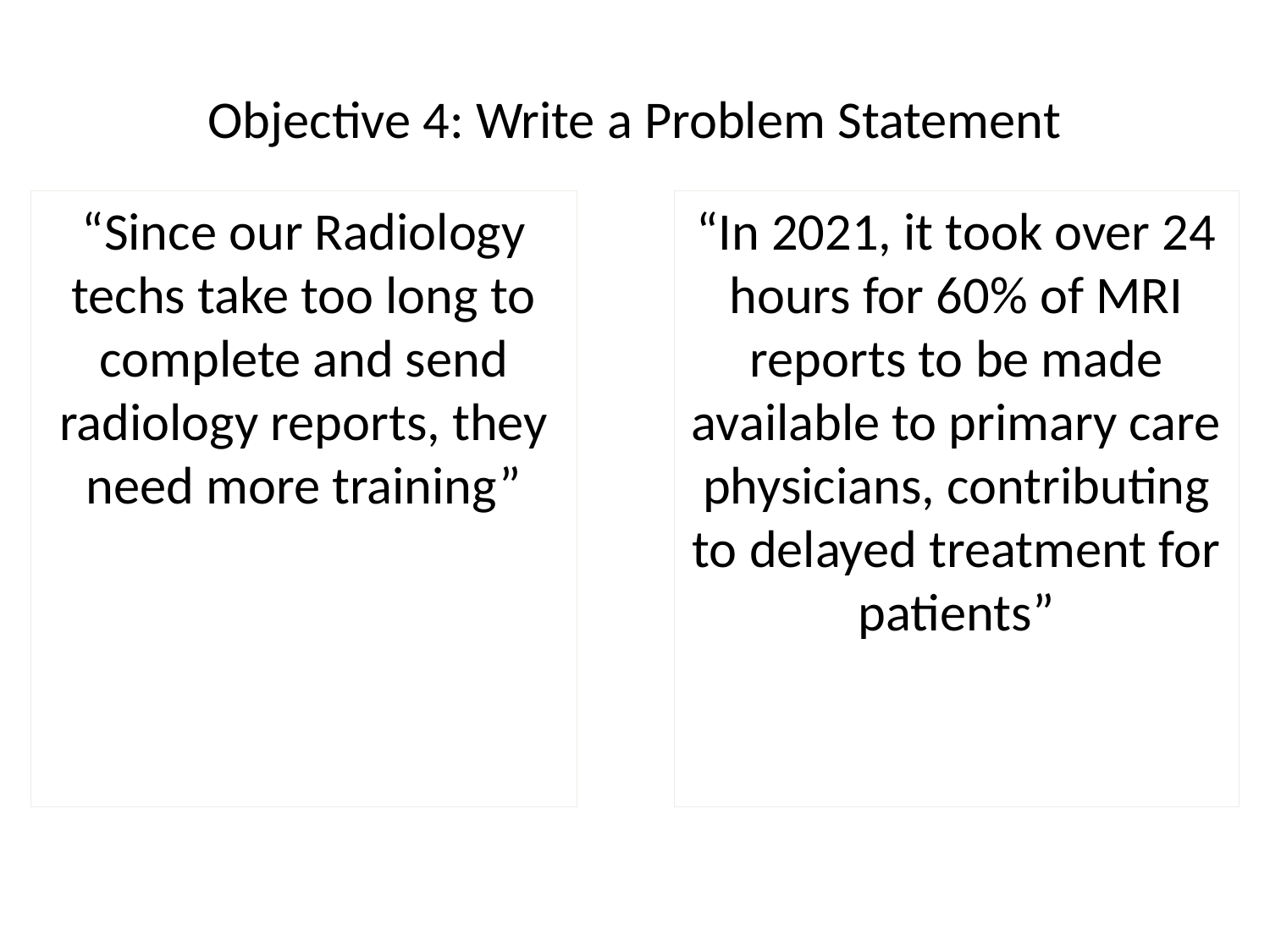

# Objective 4: Write a Problem Statement
“Since our Radiology techs take too long to complete and send radiology reports, they need more training”
“In 2021, it took over 24 hours for 60% of MRI reports to be made available to primary care physicians, contributing to delayed treatment for patients”

## Slide 55
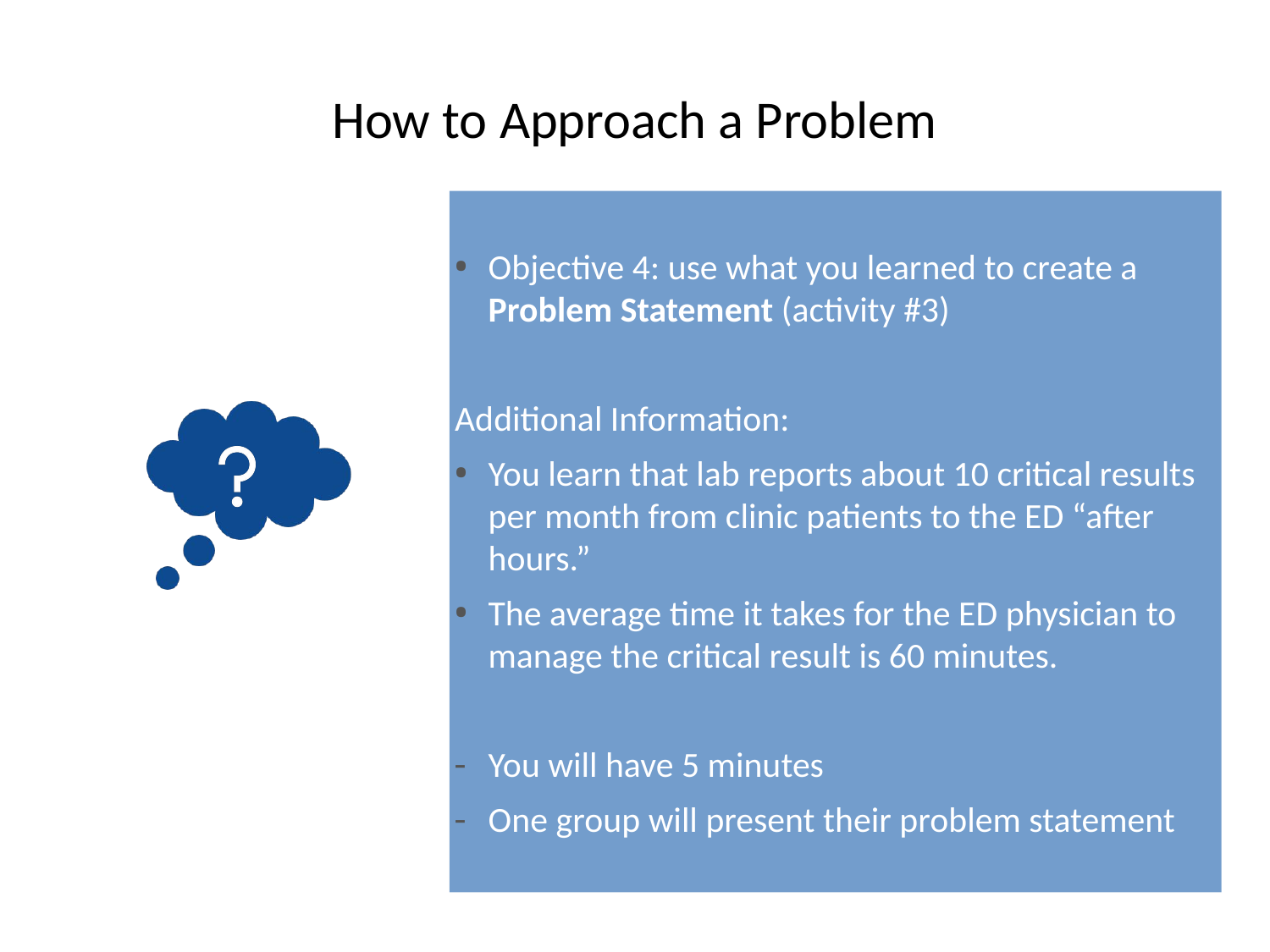

# How to Approach a Problem
Objective 4: use what you learned to create a Problem Statement (activity #3)
Additional Information:
You learn that lab reports about 10 critical results per month from clinic patients to the ED “after hours.”
The average time it takes for the ED physician to manage the critical result is 60 minutes.
You will have 5 minutes
One group will present their problem statement

## Slide 56
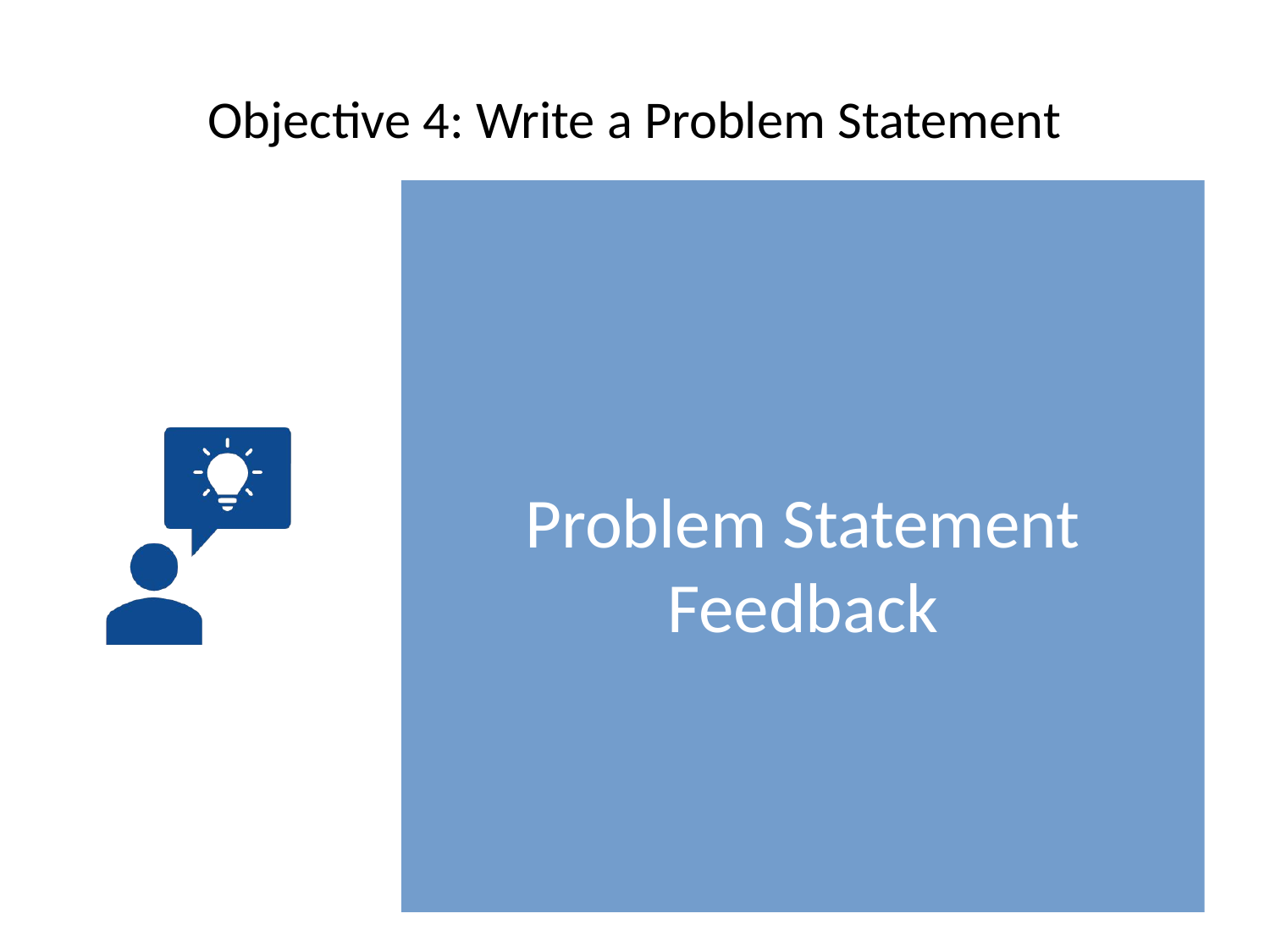

# Objective 4: Write a Problem Statement
Problem Statement Feedback

## Slide 57
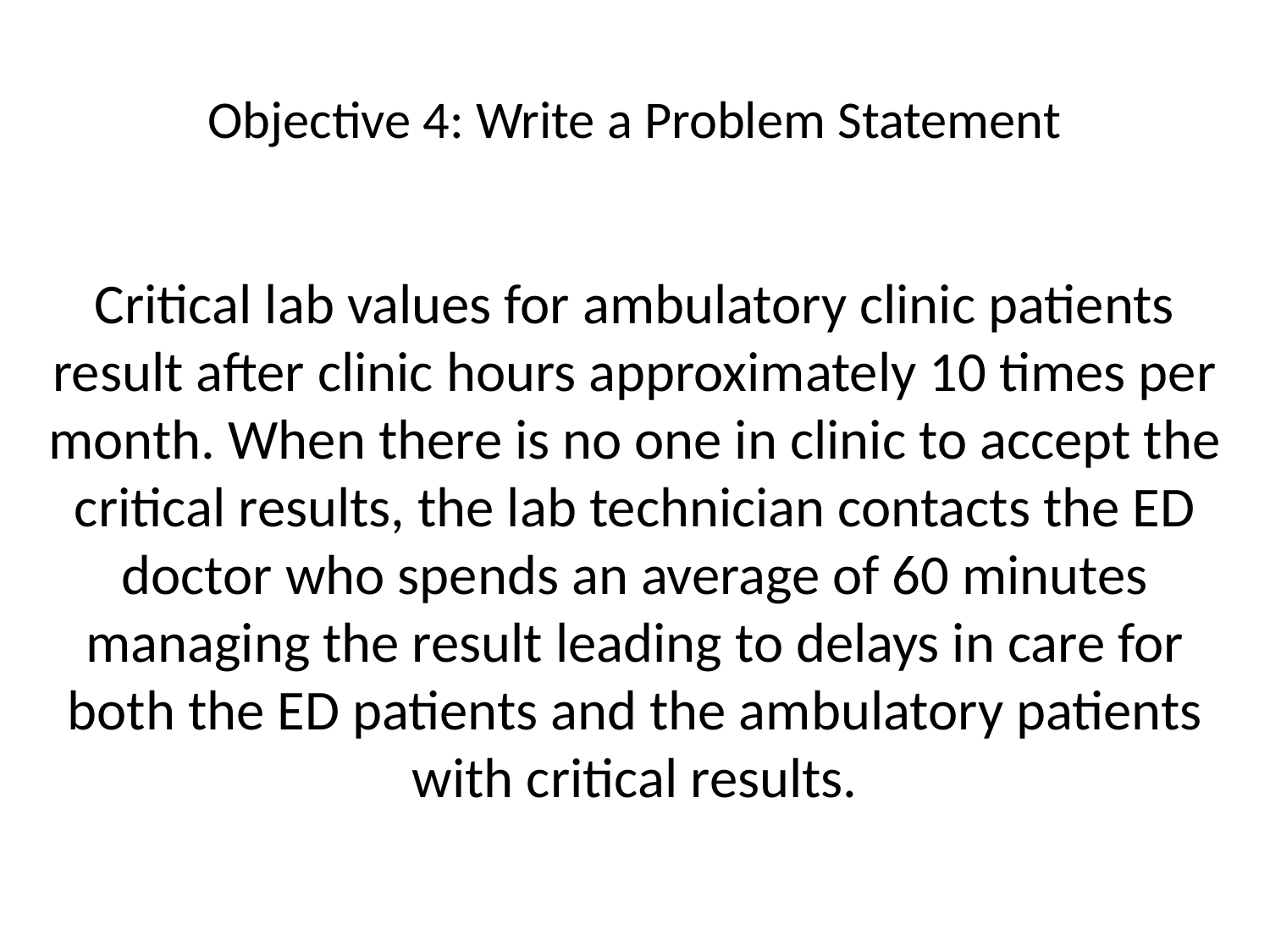

# Objective 4: Write a Problem Statement
Critical lab values for ambulatory clinic patients result after clinic hours approximately 10 times per month. When there is no one in clinic to accept the critical results, the lab technician contacts the ED doctor who spends an average of 60 minutes managing the result leading to delays in care for both the ED patients and the ambulatory patients with critical results.

## Slide 58
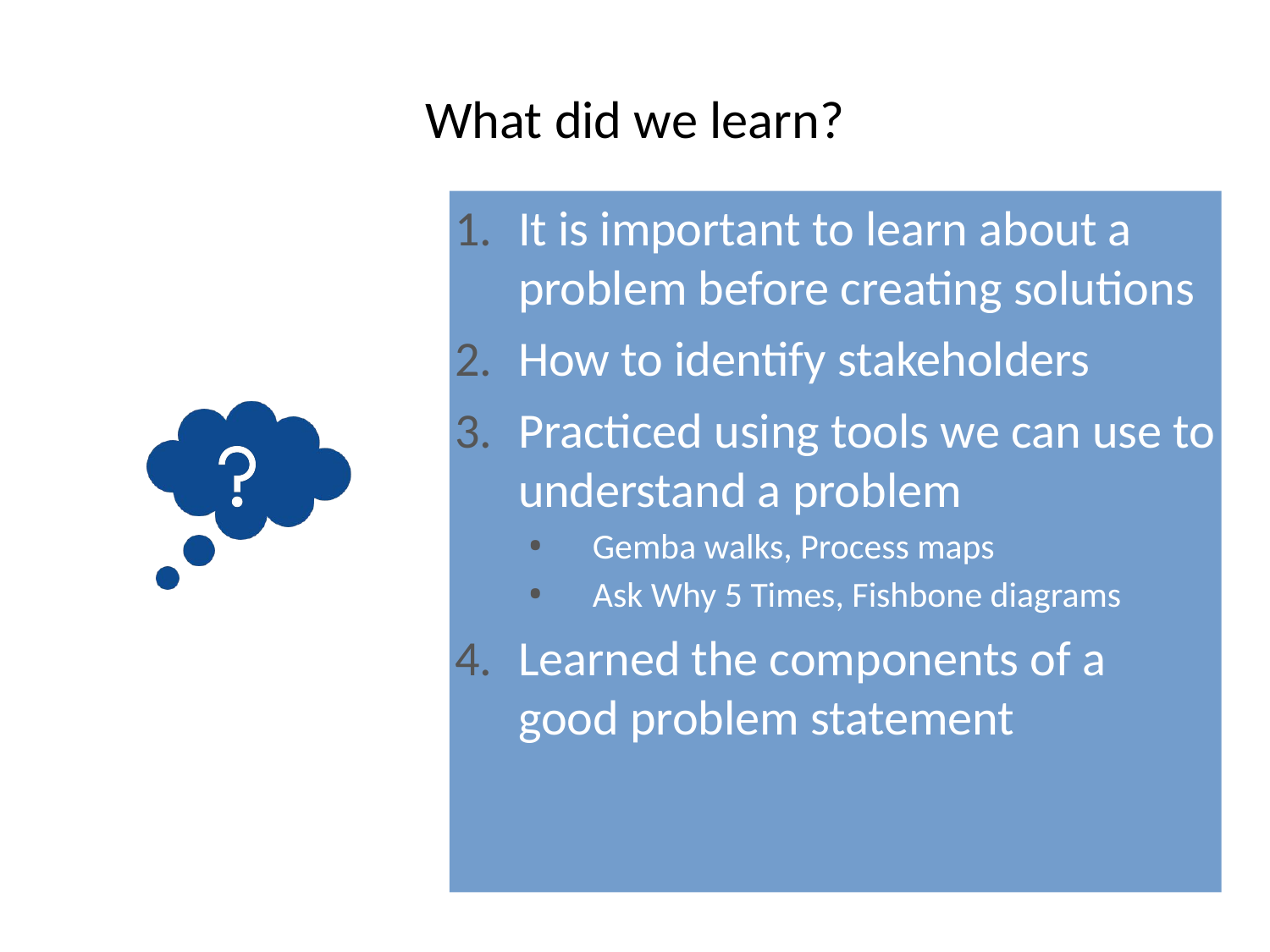

# What did we learn?
It is important to learn about a problem before creating solutions
How to identify stakeholders
Practiced using tools we can use to understand a problem
Gemba walks, Process maps
Ask Why 5 Times, Fishbone diagrams
Learned the components of a good problem statement

## Slide 59
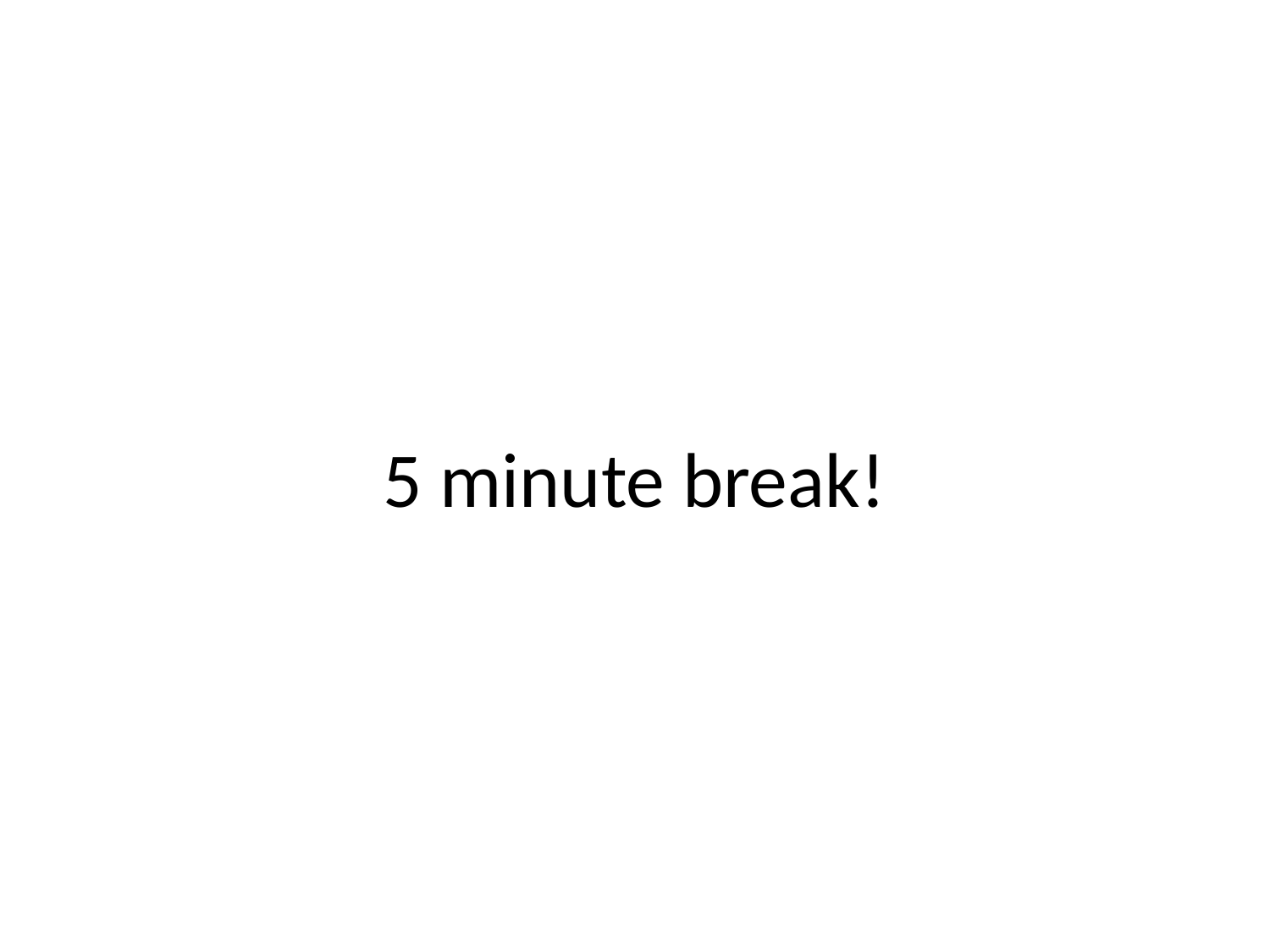

# 5 minute break!

## Slide 60
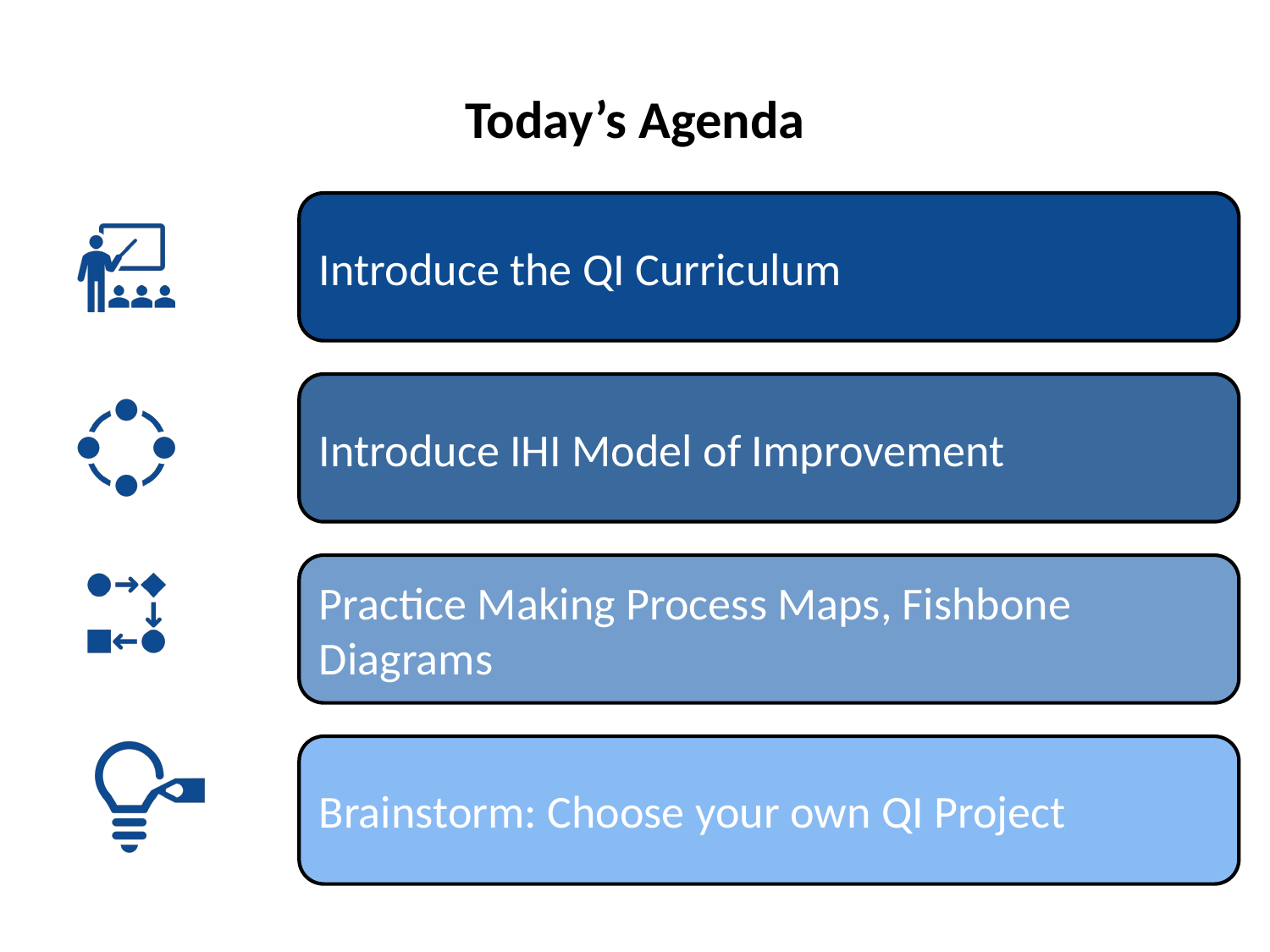

# Today’s Agenda
Introduce the QI Curriculum
Introduce IHI Model of Improvement
Practice Making Process Maps, Fishbone Diagrams
Brainstorm: Choose your own QI Project

## Slide 61
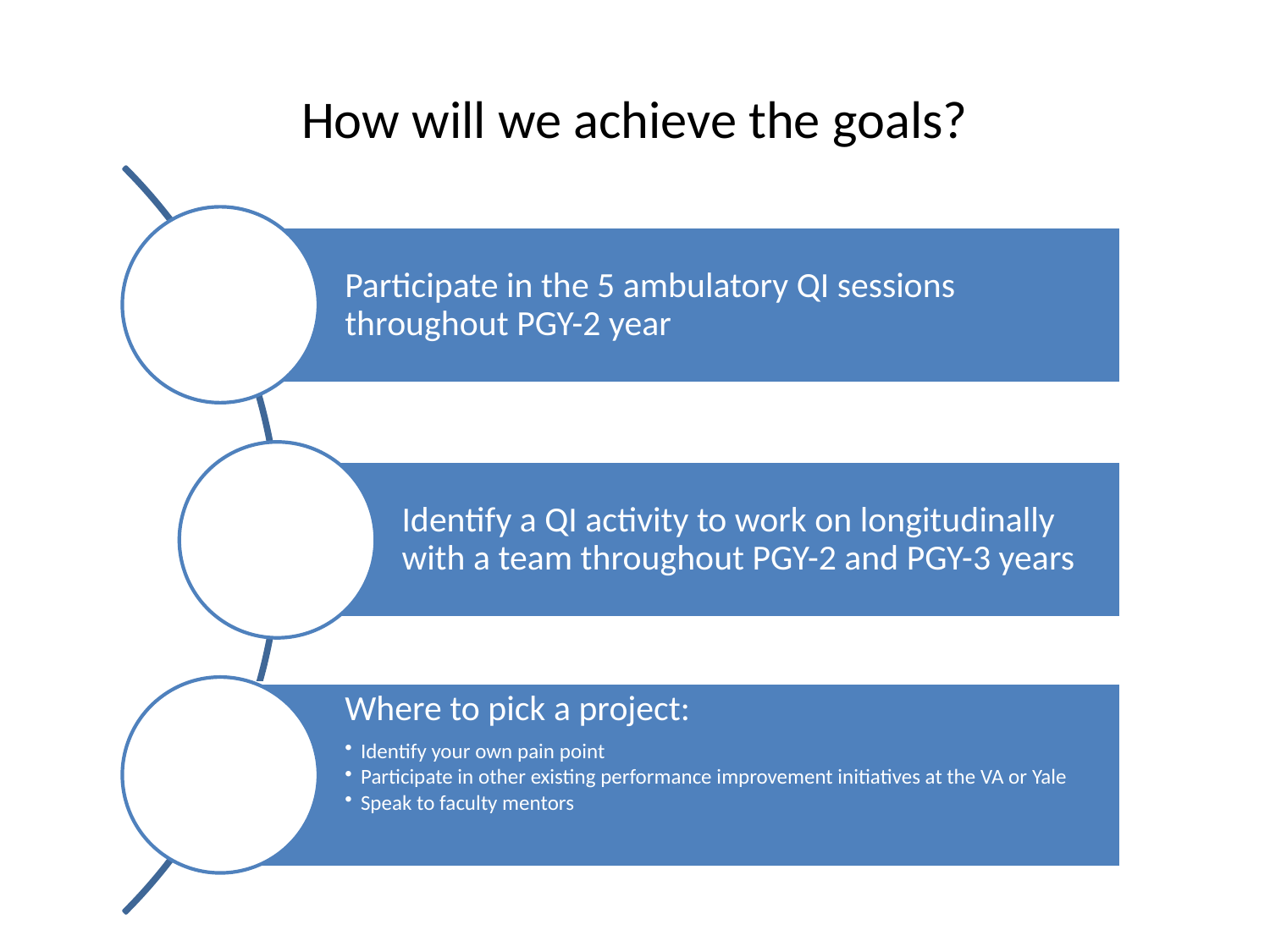

# How will we achieve the goals?

## Slide 62
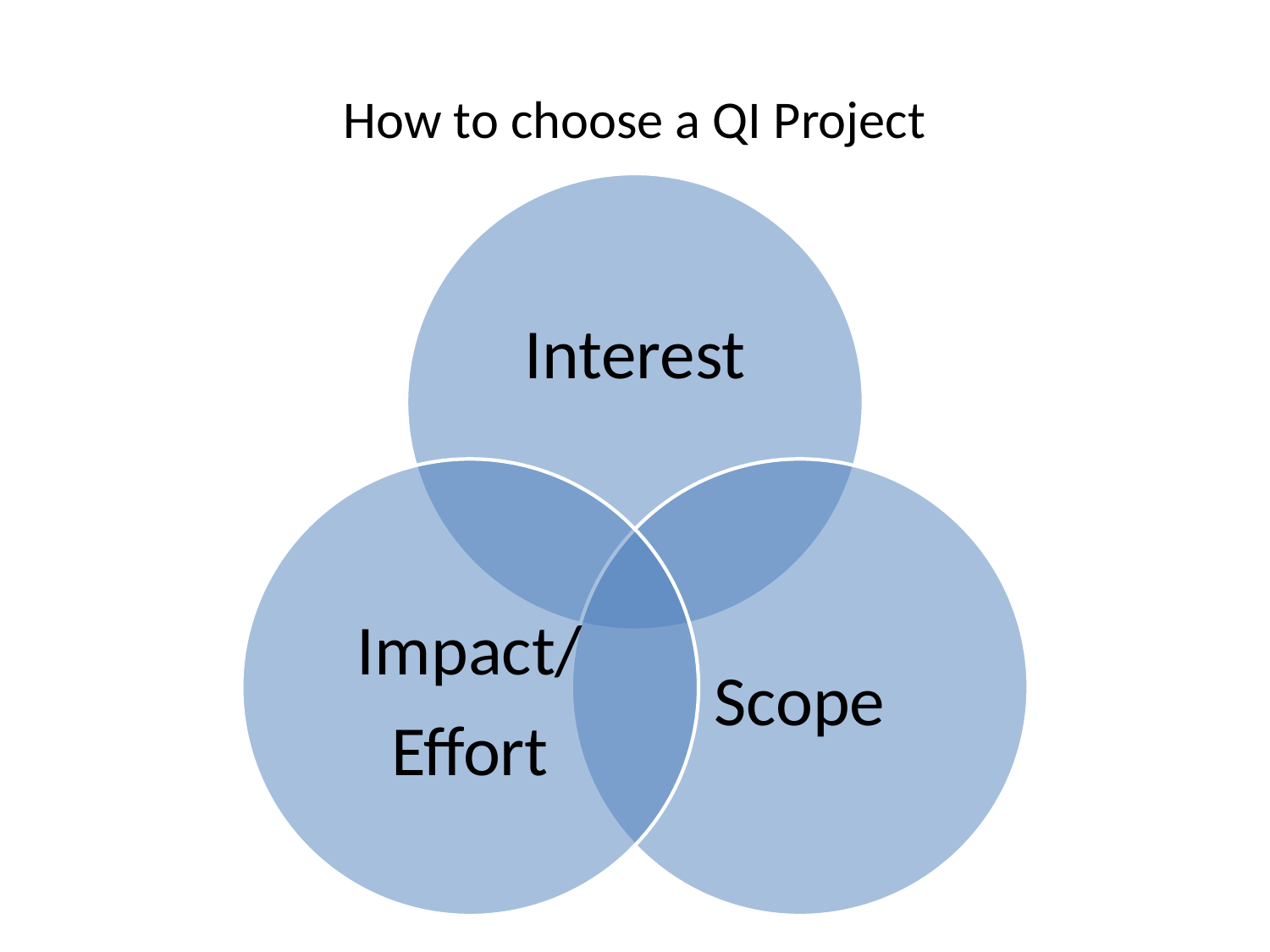

# How to choose a QI Project

## Slide 63
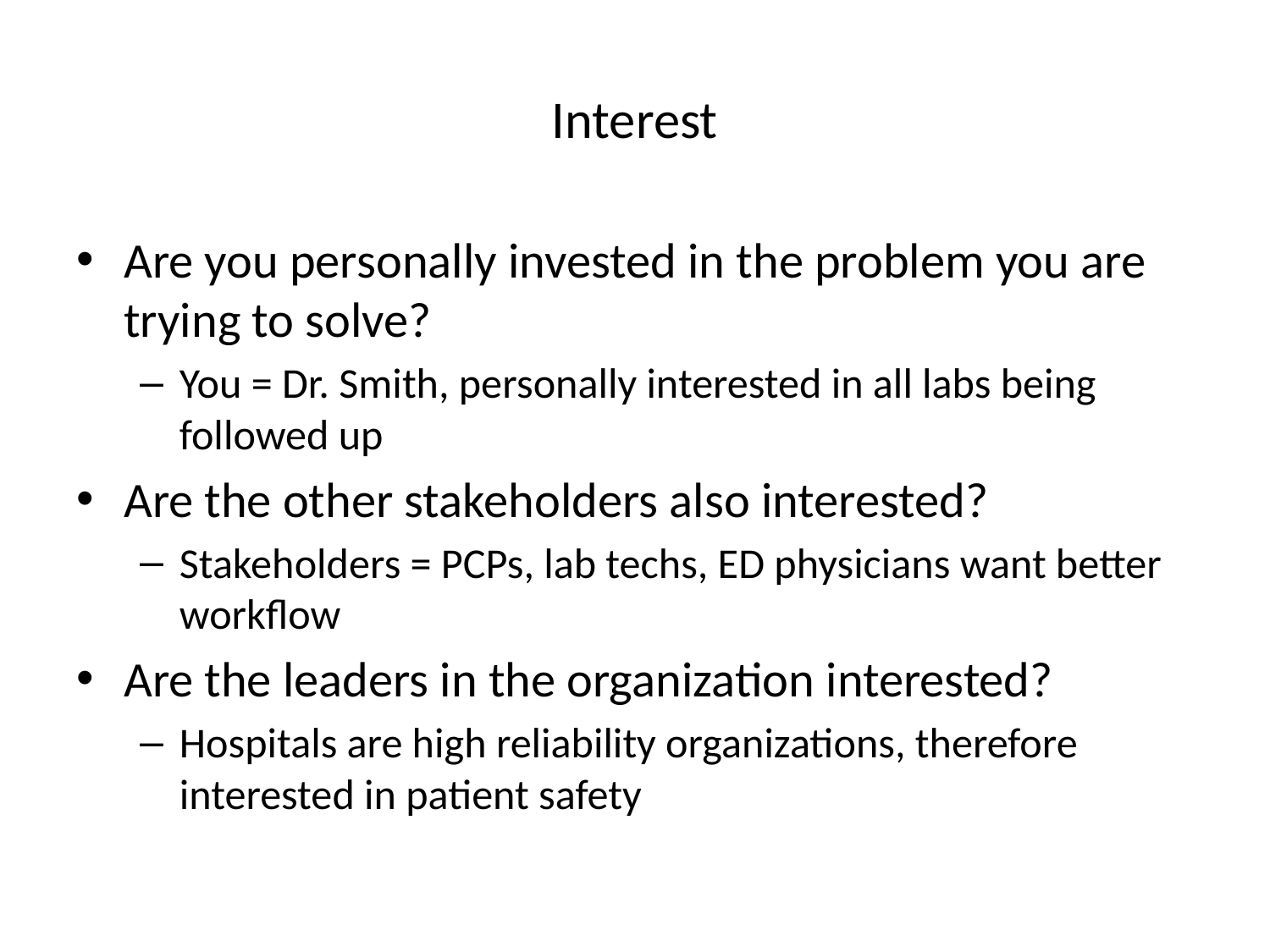

# Interest
Are you personally invested in the problem you are trying to solve?
You = Dr. Smith, personally interested in all labs being followed up
Are the other stakeholders also interested?
Stakeholders = PCPs, lab techs, ED physicians want better workflow
Are the leaders in the organization interested?
Hospitals are high reliability organizations, therefore interested in patient safety

## Slide 64
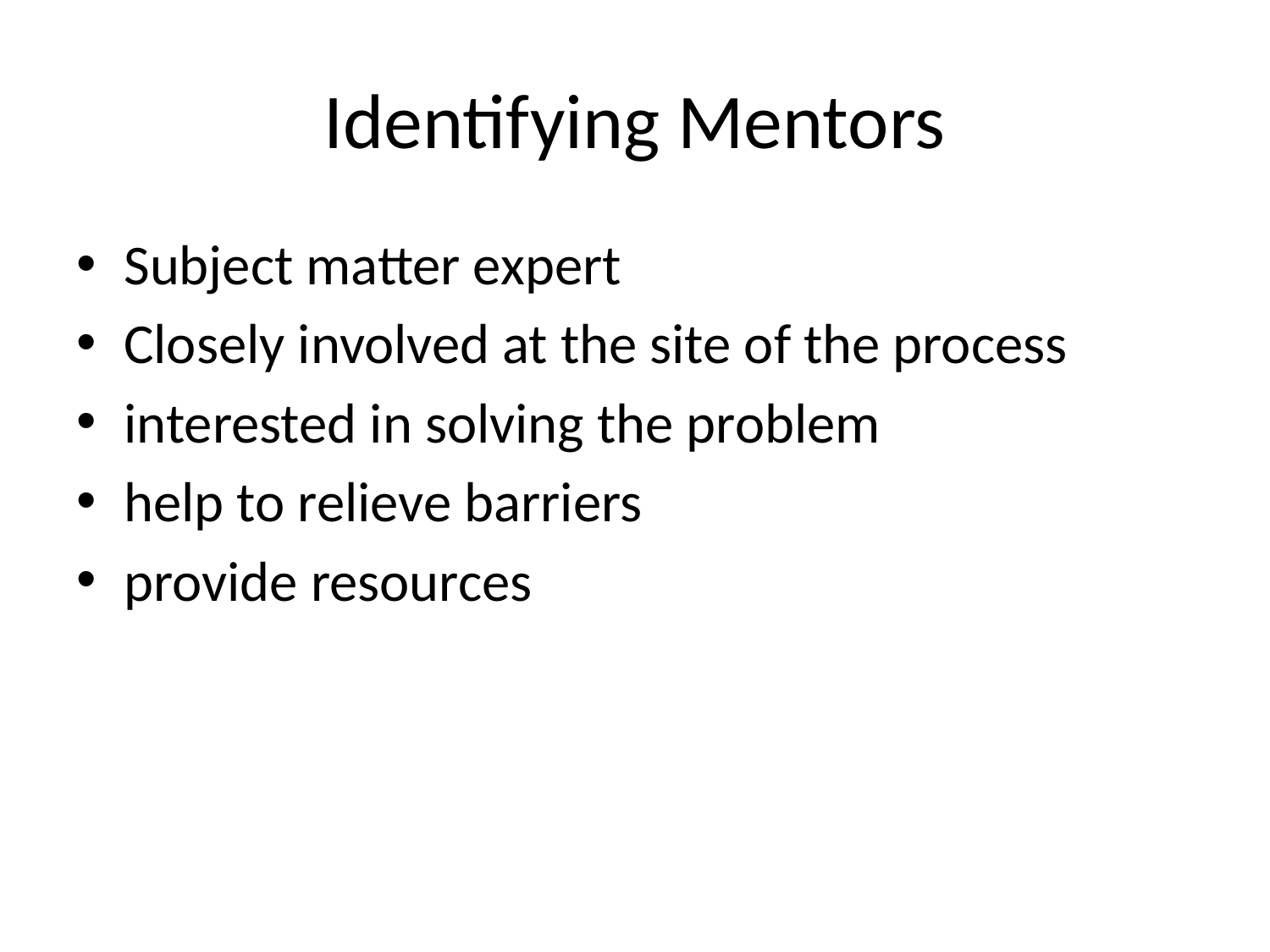

# Identifying Mentors
Subject matter expert
Closely involved at the site of the process
interested in solving the problem
help to relieve barriers
provide resources

## Slide 65
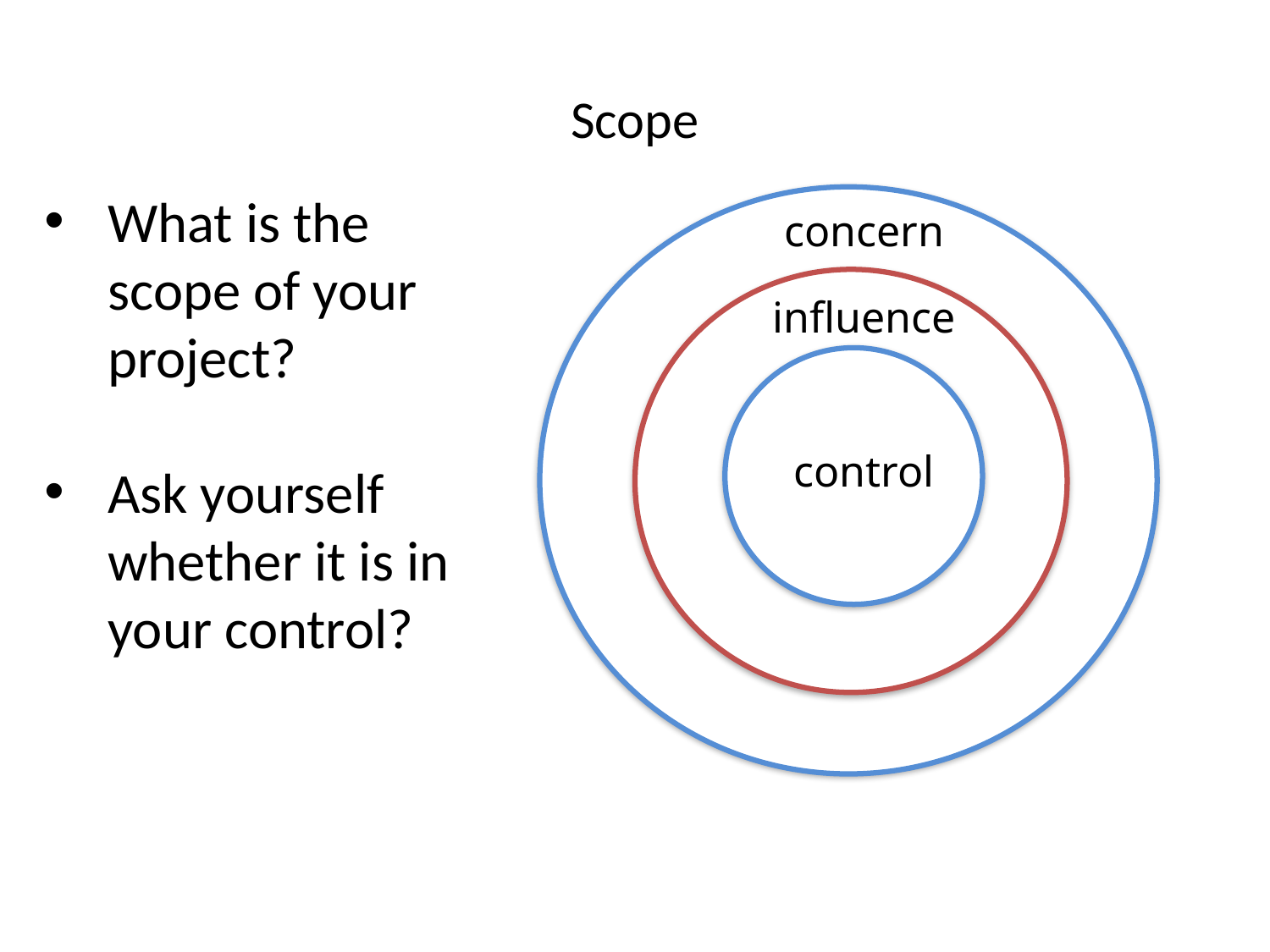

# Scope
What is the scope of your project?
Ask yourself whether it is in your control?
concern
influence
control

## Slide 66
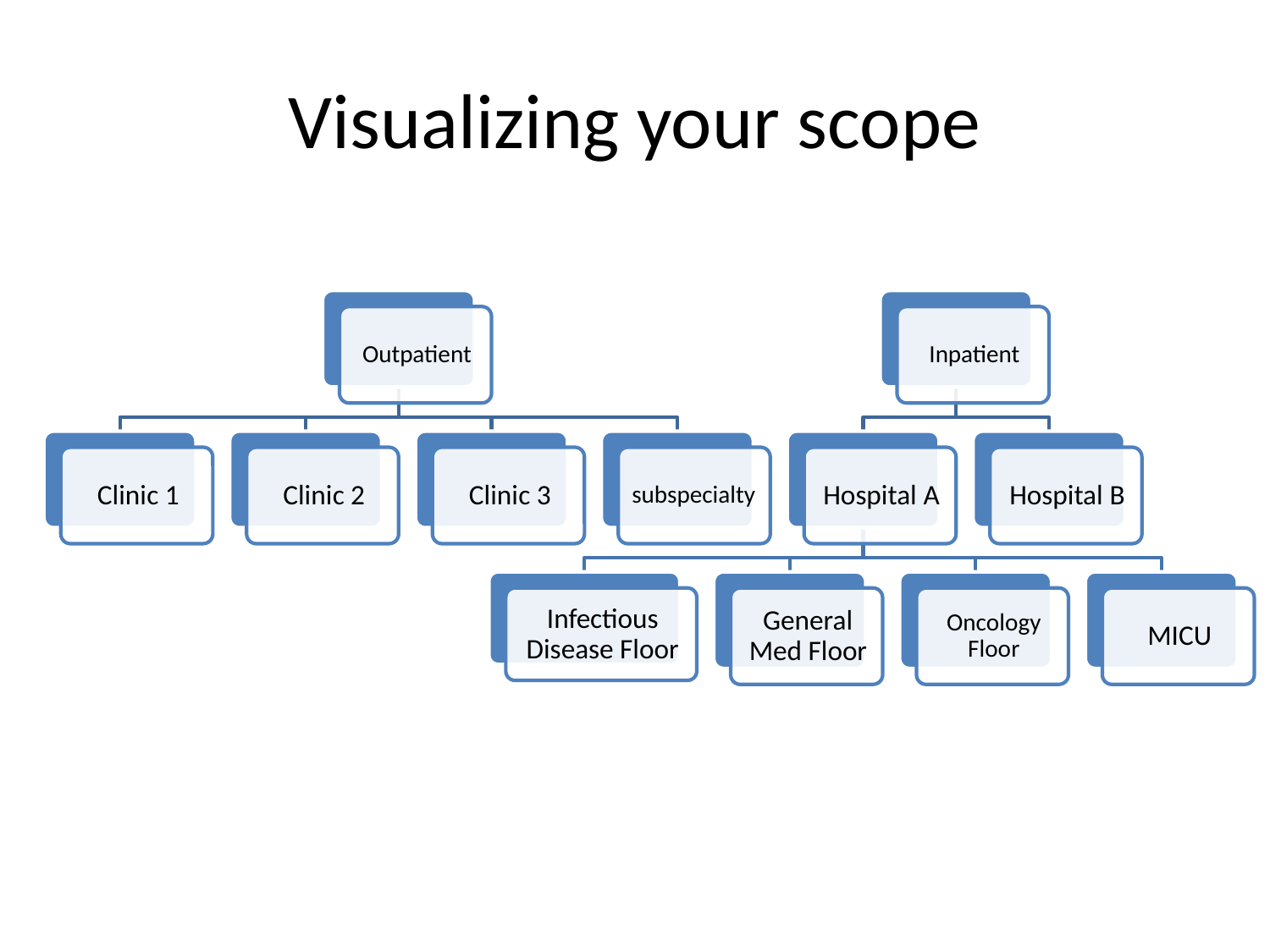

# Visualizing your scope

## Slide 67
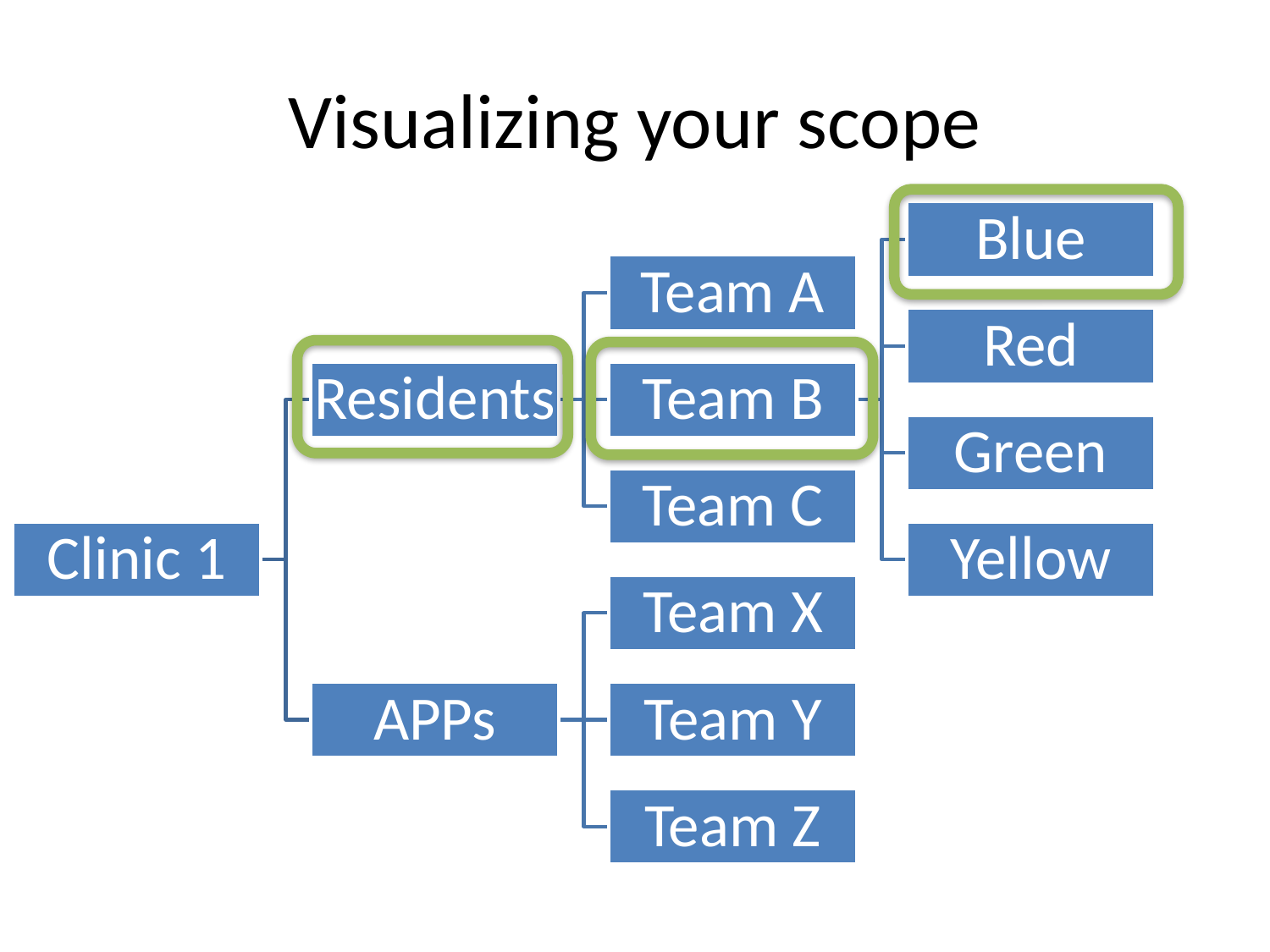

# Visualizing your scope

## Slide 68
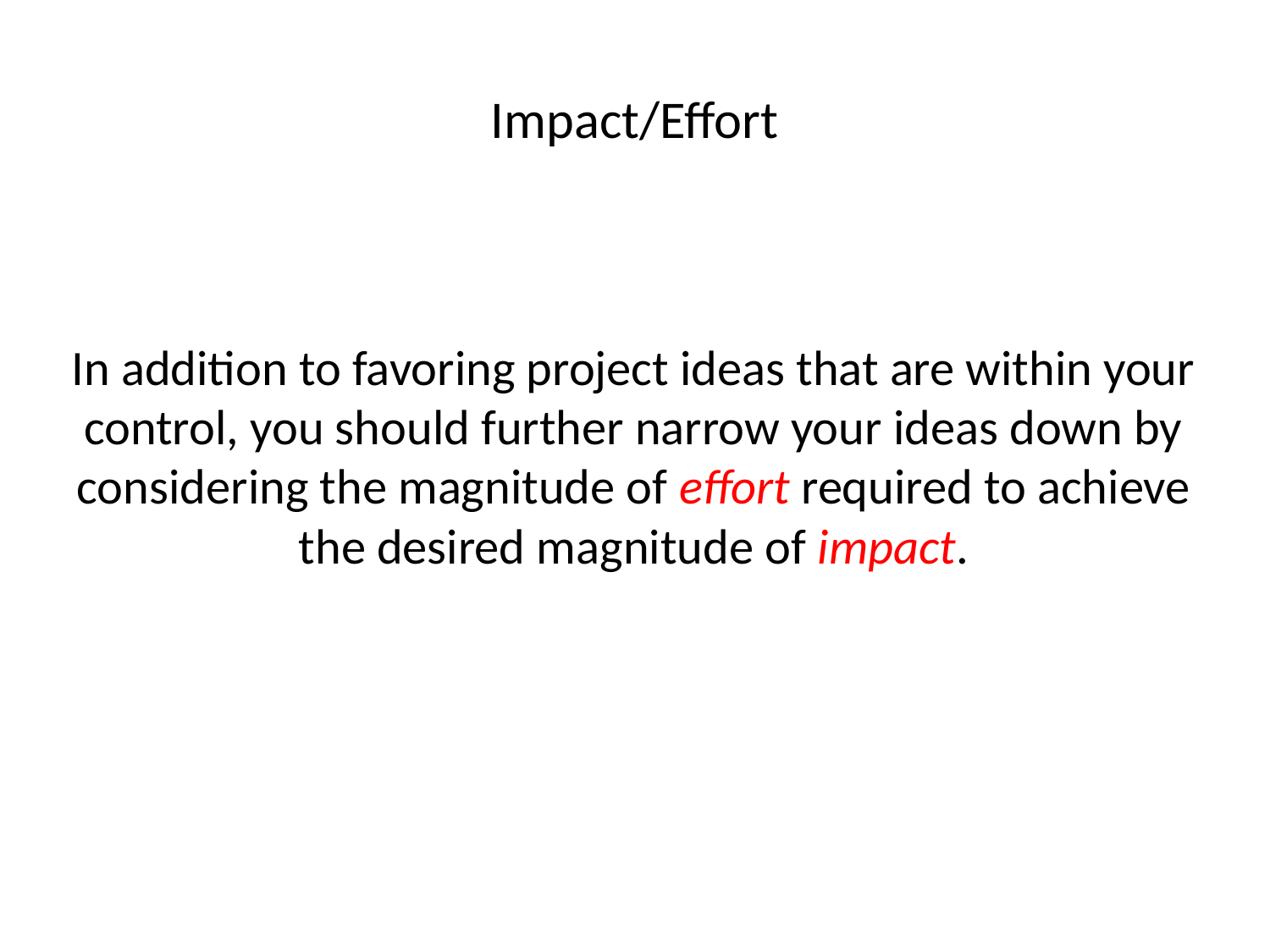

# Impact/Effort
In addition to favoring project ideas that are within your control, you should further narrow your ideas down by considering the magnitude of effort required to achieve the desired magnitude of impact.

## Slide 69
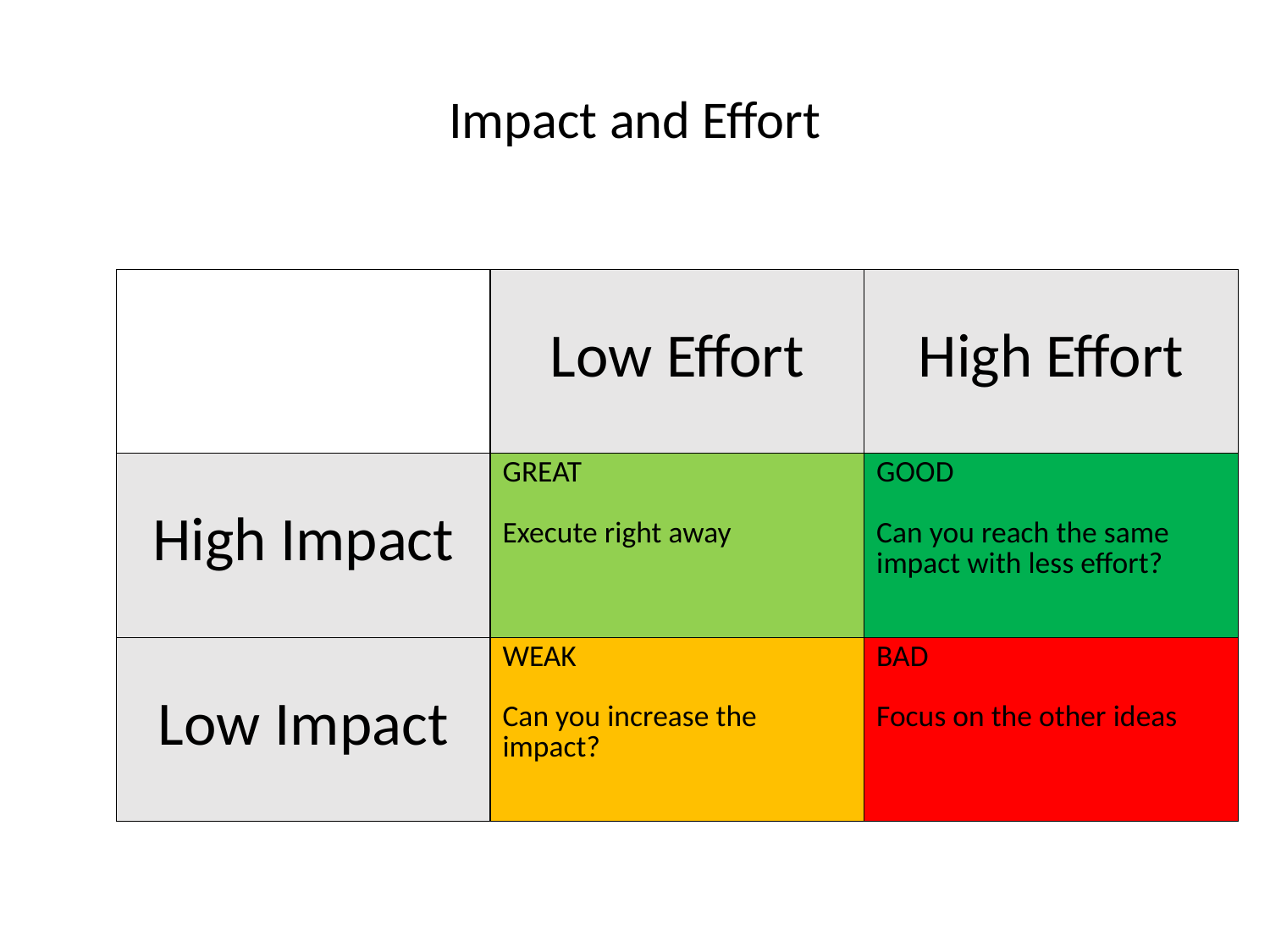

# Impact and Effort
| ​ | Low Effort​ | High Effort​ |
| --- | --- | --- |
| High Impact​ | ​GREAT Execute right away | ​GOOD Can you reach the same impact with less effort? |
| Low Impact​ | ​WEAK Can you increase the impact? | ​BAD Focus on the other ideas |

## Slide 70
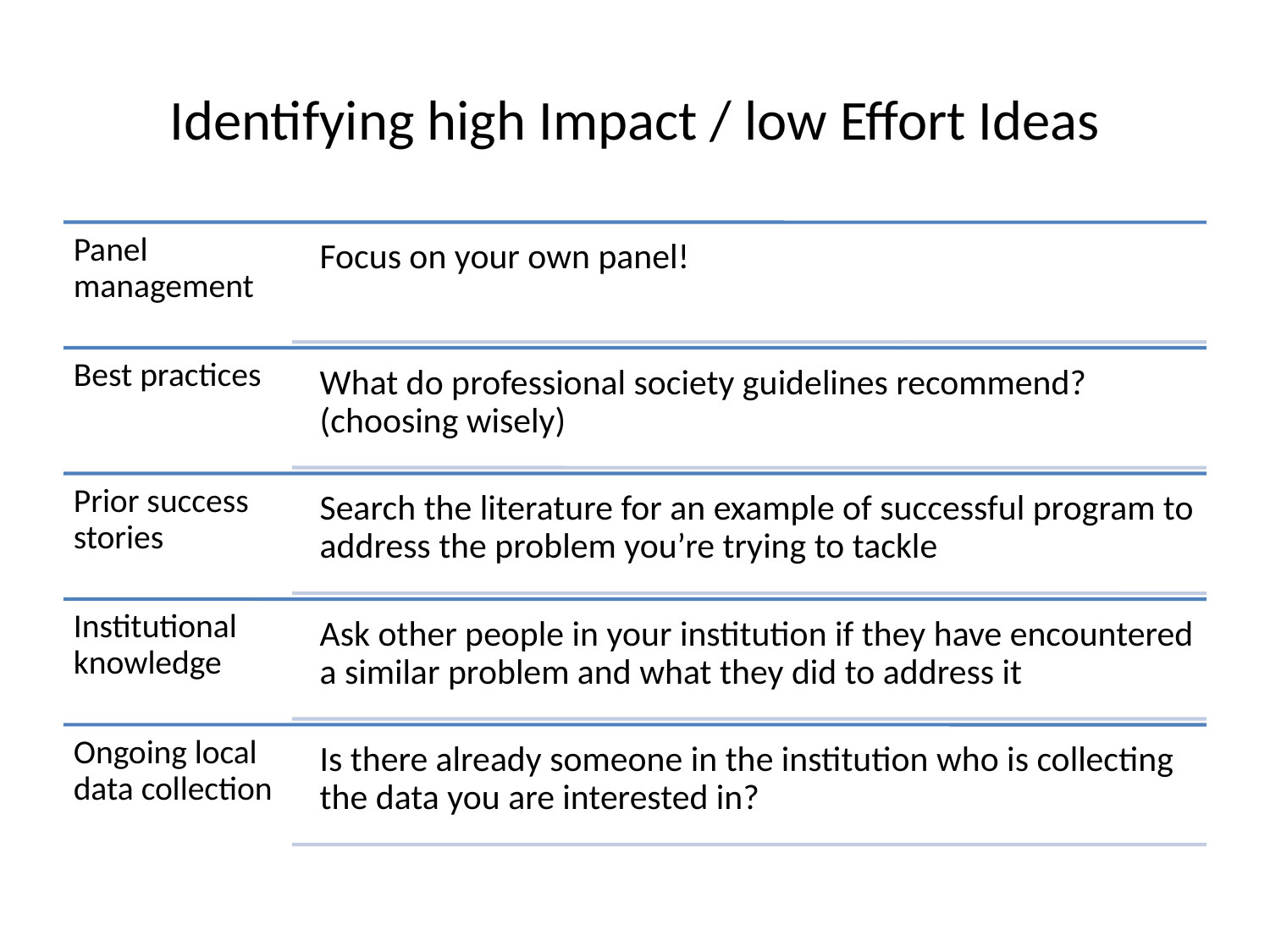

# Identifying high Impact / low Effort Ideas

## Slide 71
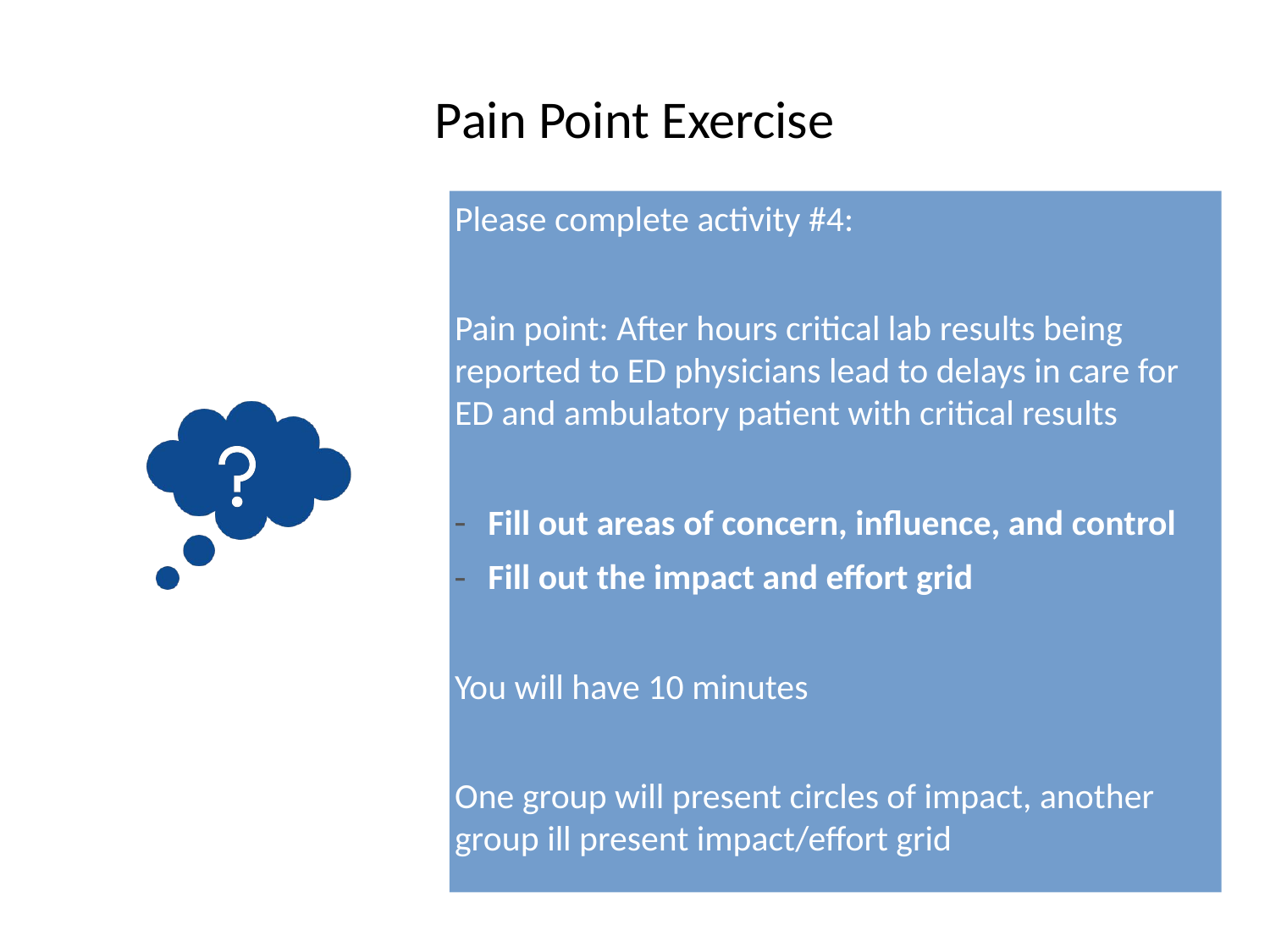

# Pain Point Exercise
Please complete activity #4:
Pain point: After hours critical lab results being reported to ED physicians lead to delays in care for ED and ambulatory patient with critical results
Fill out areas of concern, influence, and control
Fill out the impact and effort grid
You will have 10 minutes
One group will present circles of impact, another group ill present impact/effort grid

## Slide 72
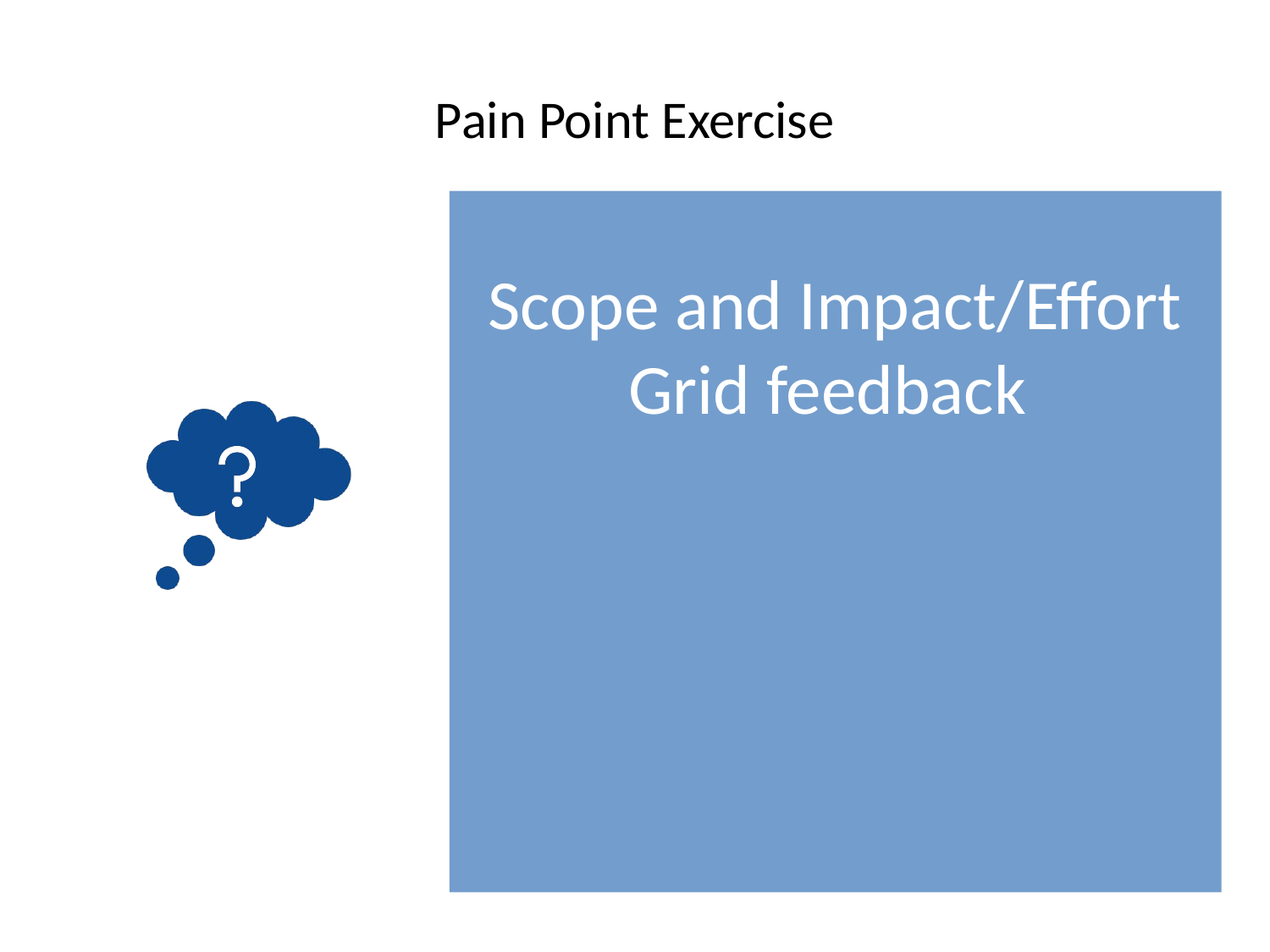

# Pain Point Exercise
Scope and Impact/Effort Grid feedback

## Slide 73
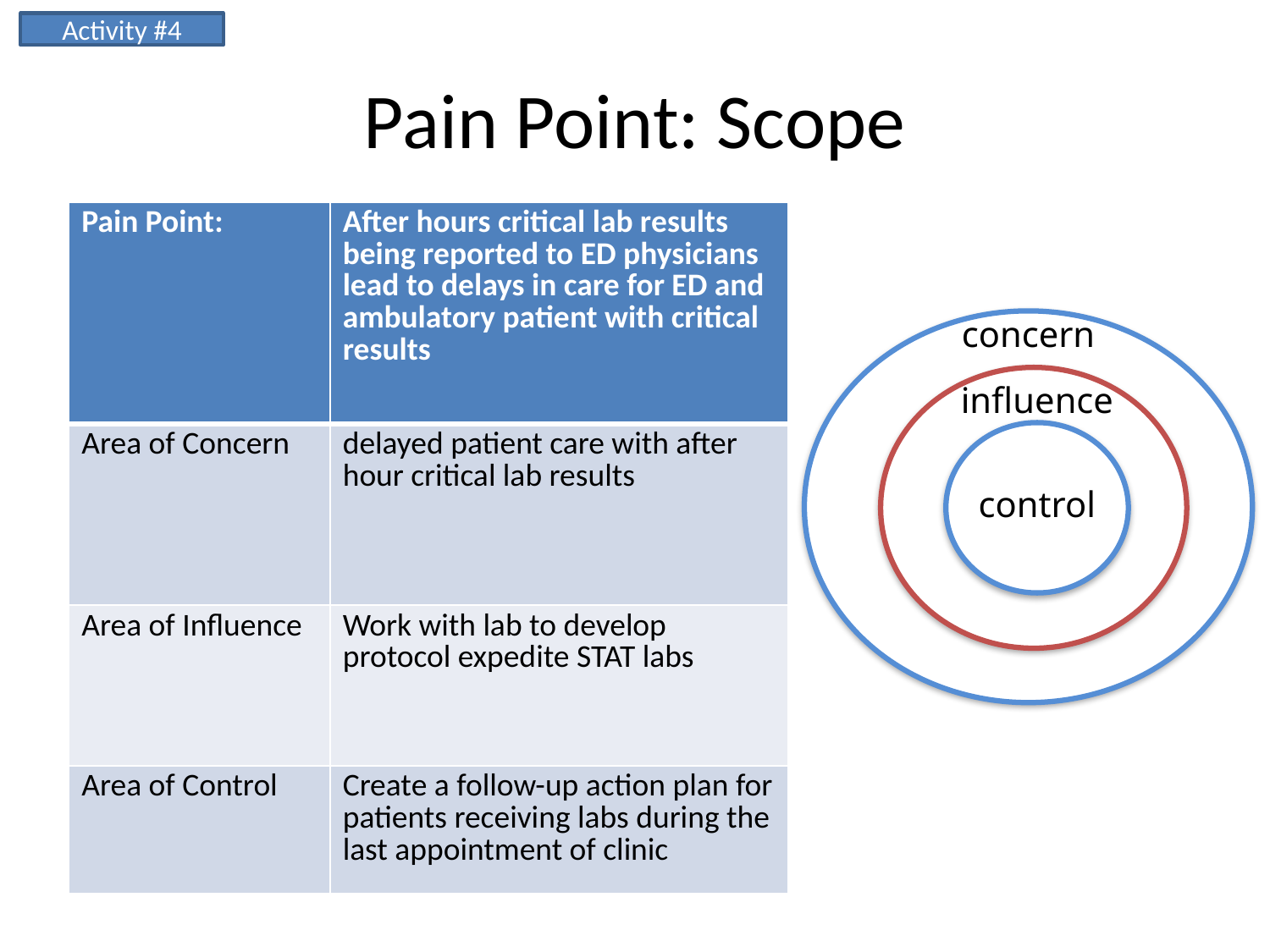

Activity #4
# Pain Point: Scope
| Pain Point: | After hours critical lab results being reported to ED physicians lead to delays in care for ED and ambulatory patient with critical results |
| --- | --- |
| Area of Concern | delayed patient care with after hour critical lab results |
| Area of Influence | Work with lab to develop protocol expedite STAT labs |
| Area of Control | Create a follow-up action plan for patients receiving labs during the last appointment of clinic |
concern
influence
control

## Slide 74
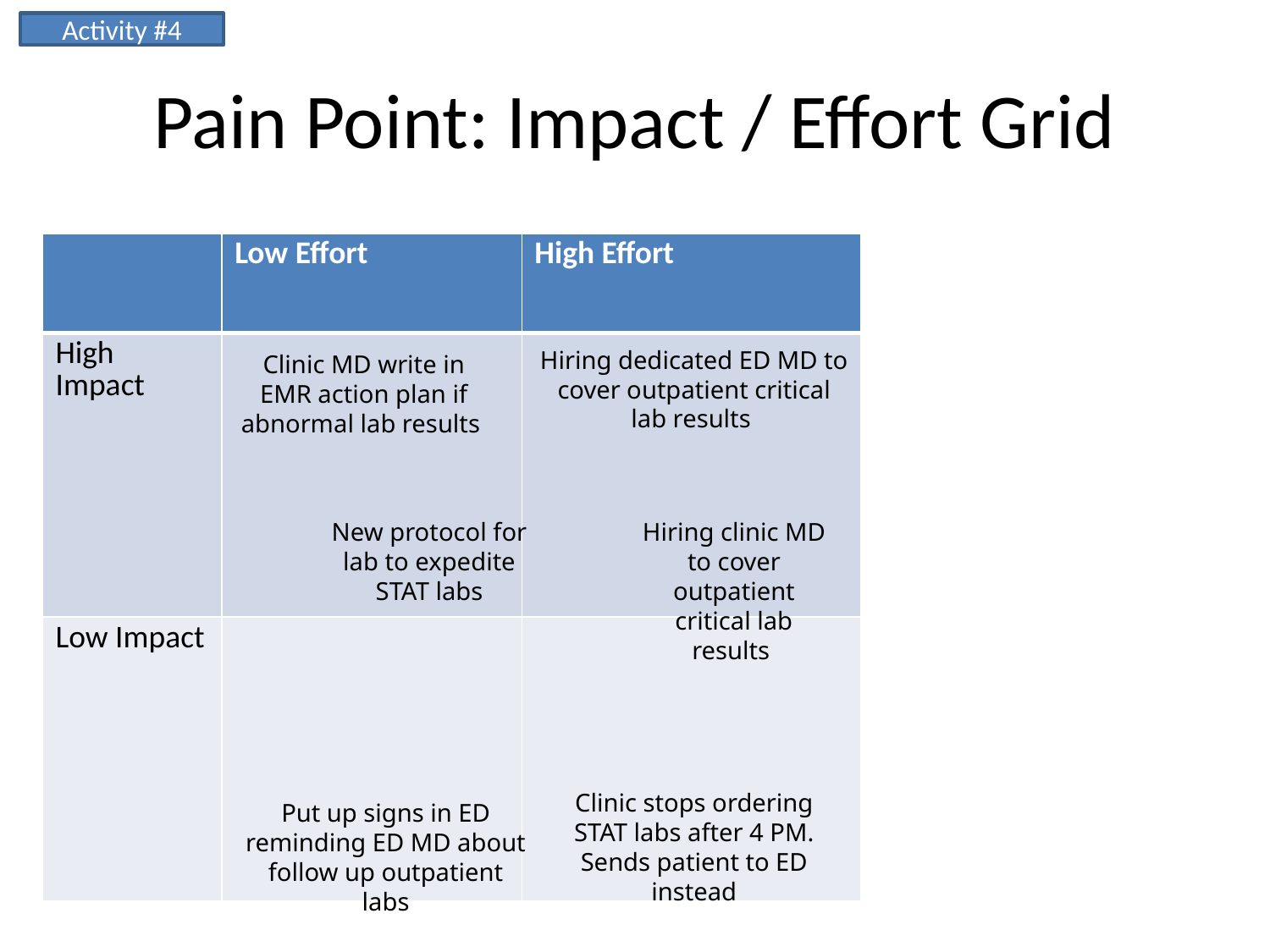

Activity #4
# Pain Point: Impact / Effort Grid
| | Low Effort | High Effort |
| --- | --- | --- |
| High Impact | | |
| Low Impact | | |
Hiring dedicated ED MD to cover outpatient critical lab results
Clinic MD write in EMR action plan if abnormal lab results
New protocol for lab to expedite STAT labs
Hiring clinic MD to cover outpatient critical lab results
Clinic stops ordering STAT labs after 4 PM. Sends patient to ED instead
Put up signs in ED reminding ED MD about follow up outpatient labs

## Slide 75
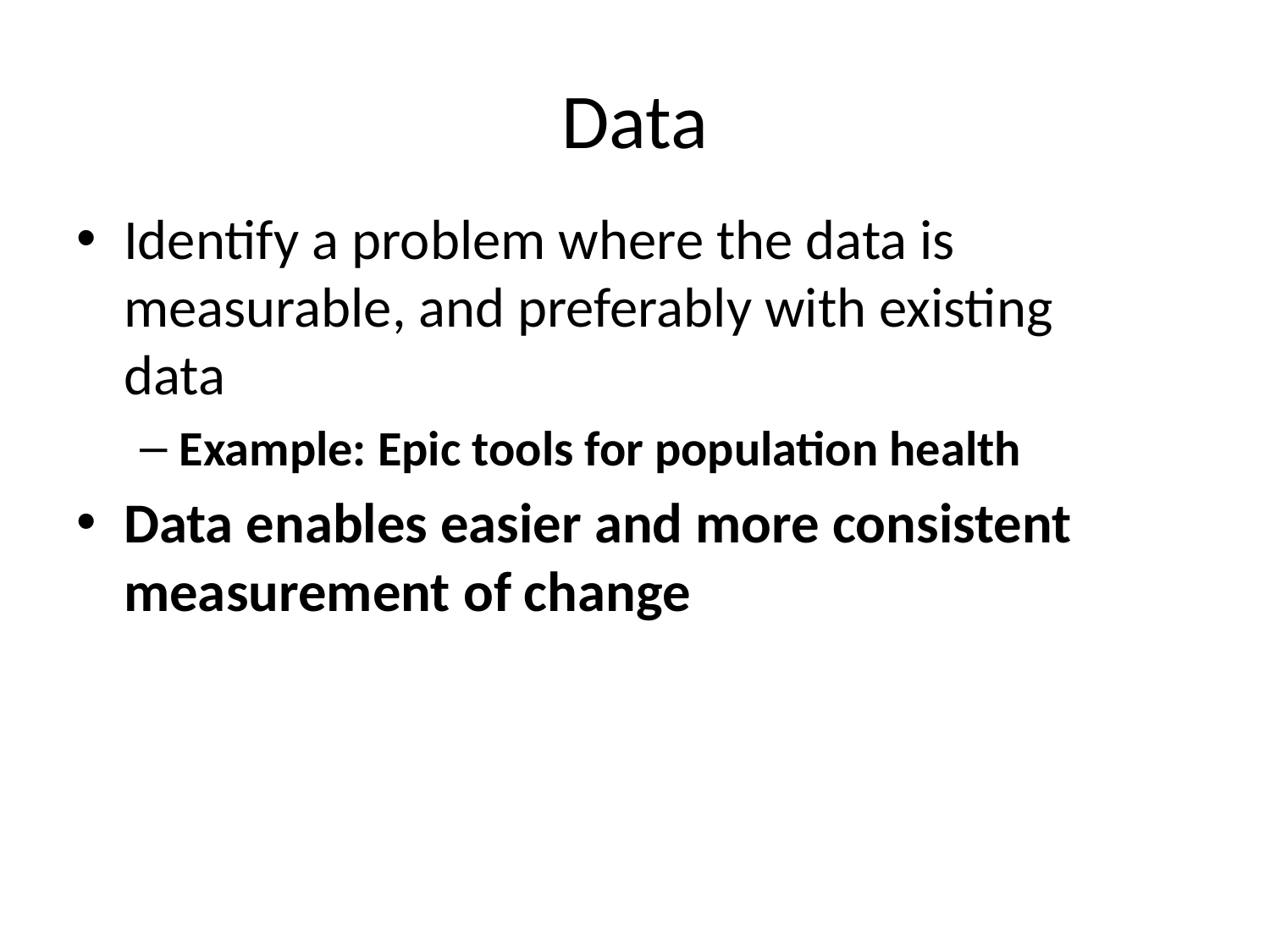

# Data
Identify a problem where the data is measurable, and preferably with existing data
Example: Epic tools for population health
Data enables easier and more consistent measurement of change

## Slide 76
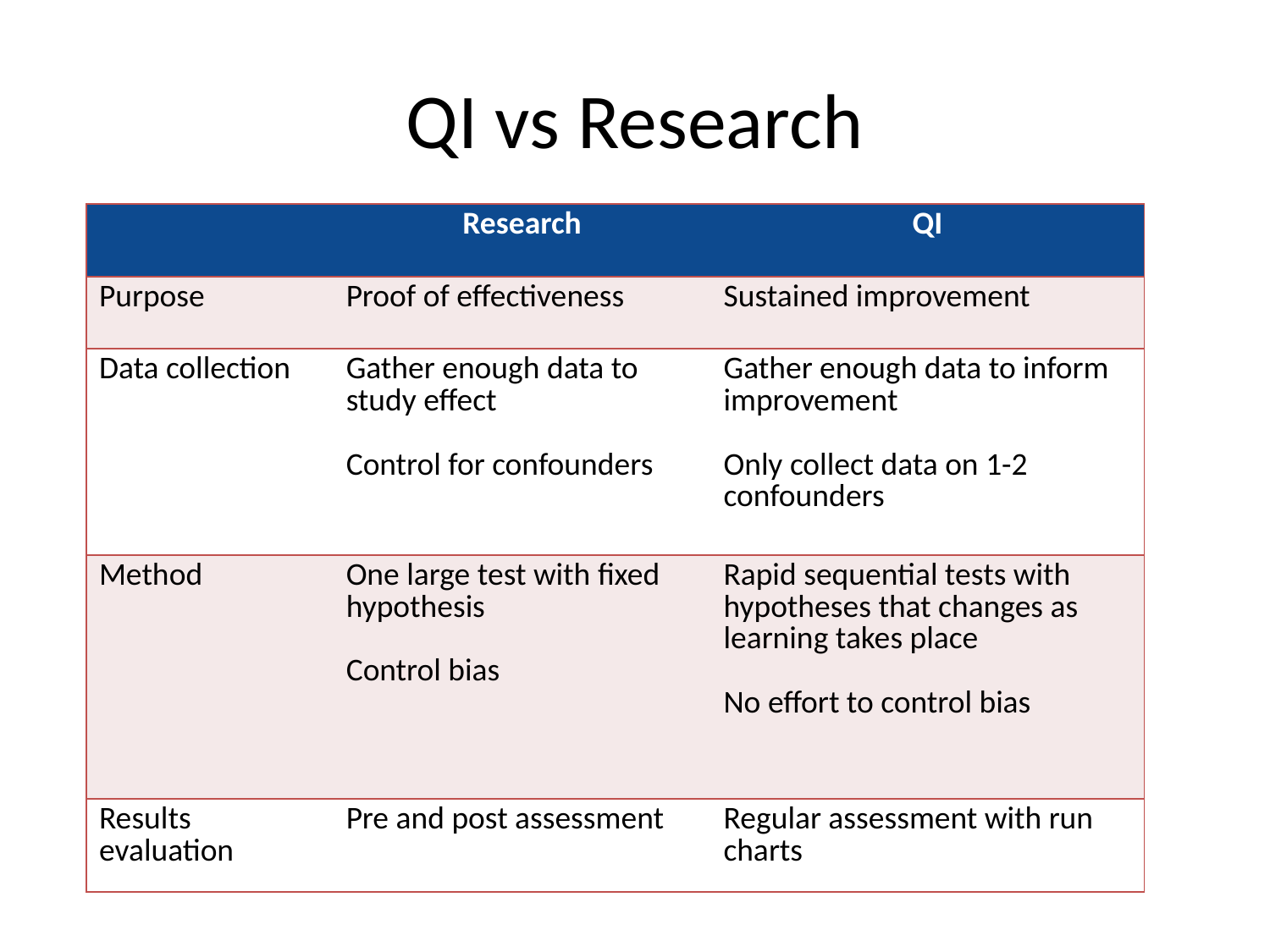

# QI vs Research
| | Research | QI |
| --- | --- | --- |
| Purpose | Proof of effectiveness | Sustained improvement |
| Data collection | Gather enough data to study effect Control for confounders | Gather enough data to inform improvement Only collect data on 1-2 confounders |
| Method | One large test with fixed hypothesis Control bias | Rapid sequential tests with hypotheses that changes as learning takes place No effort to control bias |
| Results evaluation | Pre and post assessment | Regular assessment with run charts |

## Slide 77
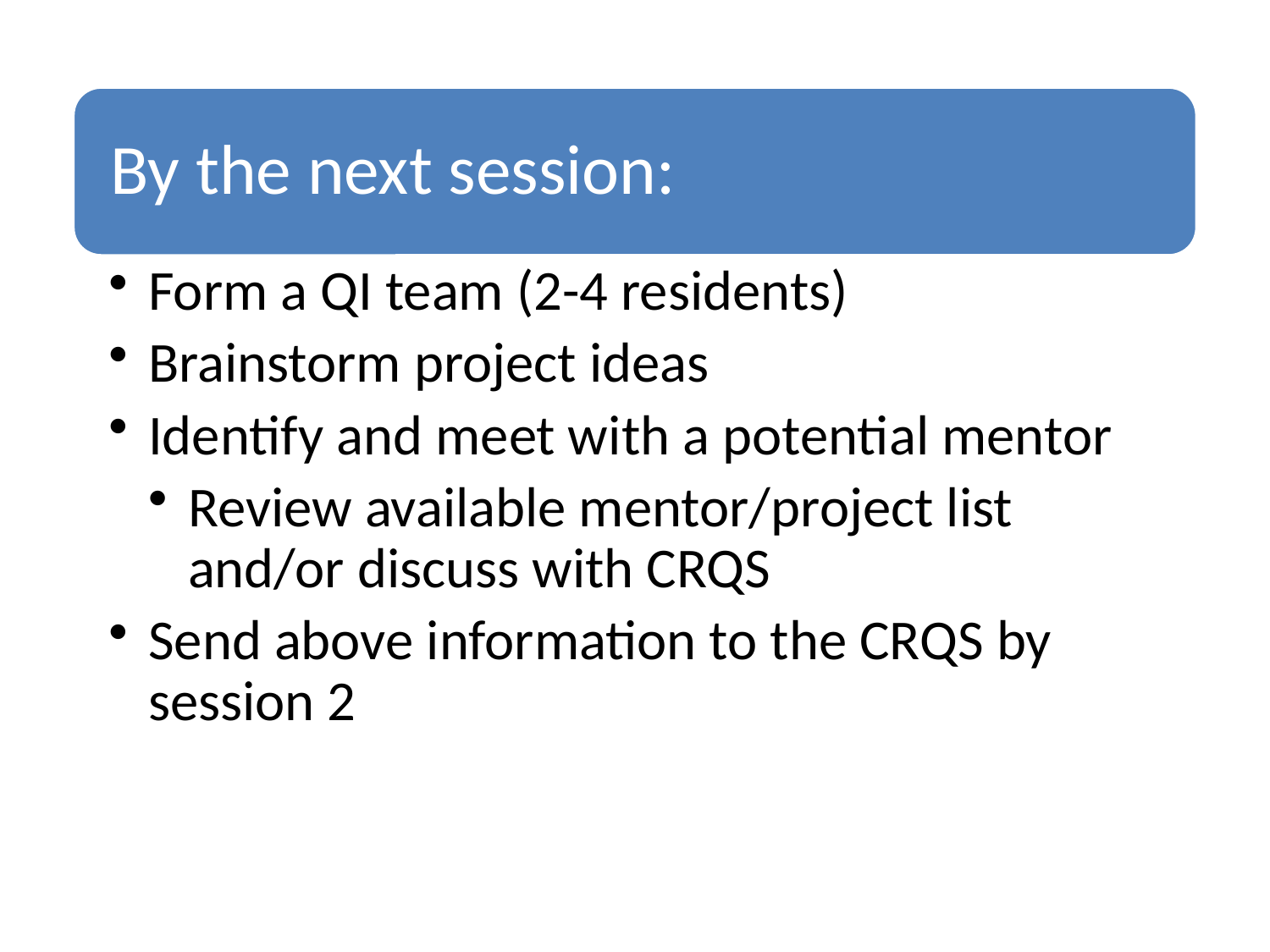

Supplement: Supplementary file 1 — Session 1 Slides.pptxSession 1 Workbook.pptxSession 2 Slides.pptxSession 2 Workbook.pptxSession 3 Slides.pptxSession 4 Work-in-Progress Presentation Template.pptxSession 5 Slides.pptxQI Charter Template.docxFaculty Milestones.docxFaculty Guide.docxResident Survey.docx [file mep_2374-8265.11310-s001.zip › A. Session 1 Slides.pptx]
