# Supplementary material for: Building Resident Quality Improvement Knowledge and Engagement Through a Longitudinal, Mentored, and Experiential Learning-Based Quality Improvement Curriculum
Source: MedEdPORTAL. 2023 Apr 18;19:11310. doi: 10.15766/mep_2374-8265.11310 (PMC10110773; doi:10.15766/mep_2374-8265.11310)
Supplement: Supplementary file 1 — Session 1 Slides.pptxSession 1 Workbook.pptxSession 2 Slides.pptxSession 2 Workbook.pptxSession 3 Slides.pptxSession 4 Work-in-Progress Presentation Template.pptxSession 5 Slides.pptxQI Charter Template.docxFaculty Milestones.docxFaculty Guide.docxResident Survey.docx [file mep_2374-8265.11310-s001.zip › Educational Summary Report_11310.pdf]

MedEdPORTAL® The AAMC Journal of Teaching and Learning Resources

Original Publication Open Access

Building Resident Quality Improvement Knowledge and Engagement Through a Longitudinal, Mentored, and Experiential Learning-Based Quality Improvement Curriculum

Jenny Xiang, MD*, Samantha Magier, MD, ME, Jadry Gruen, MD, Megan Welch, MD, Victor Bilan, MD, Benjamin Rodwin, MD, Alexandra Norcott, MD, Naseema Merchant, MBBS

*Corresponding author: jjxiang@mdanderson.org

Abstract

Introduction: Quality improvement (QI) training is an essential component of resident medical education and a part of the ACGME core competencies. We present our residency's evidence-based QI curriculum, which outlines key components identified in the literature for successful QI education. **Methods:** Our curriculum included a mandatory five-part longitudinal educational series during ambulatory education sessions for second-year residents. Modeled after the Institute for Healthcare Improvement model for improvement and taught by a chief resident, our curriculum introduced residents to key QI concepts through case-based, just-in-time didactics and applied experiential learning via concurrent resident-led longitudinal QI projects. Residents received structured, multilayer mentorship from a faculty mentor in their field of interest and the chief resident of quality and patient safety. Their work-in-progress projects were presented to faculty QI experts and institutional leadership for additional feedback and mentorship. **Results:** Since 2016, a total of 234 internal medicine residents have completed our QI curriculum and developed 67 QI projects, which have been presented at various local, regional, and national conferences. In the 2 most recent academic years, Quality Improvement Knowledge Application Tool Revised (QIKAT-R) scores significantly increased from 4.6 precurriculum to 6.3 postcurriculum ($p < .001$). **Discussion:** A longitudinal, experiential, and mentored QI curriculum teaches residents QI skill sets through incorporating mechanisms associated with successful educational initiatives and adult learning theory. Our QIKAT-R results and project output show that our curriculum is associated with improved trainee QI knowledge and systems-level improvements.

Keywords
Case-Based Learning, Clinical/Procedural Skills Training, Experiential Learning, Internal Medicine, Mentoring/Coaching, Problem-Based Learning, Quality Improvement/Patient Safety

Educational Objectives

After completing the curriculum, learners will be able to:

1. Understand key quality improvement (QI) concepts and principles, as measured by the Quality Improvement Knowledge Application Tool Revised.
2. Apply their QI knowledge by designing and implementing team-based QI projects using the Institute for Healthcare Improvement model for improvement.

Citation:
Xiang J, Magier S, Gruen J, et al. Building resident quality improvement knowledge and engagement through a longitudinal, mentored, and experiential learning-based quality improvement curriculum. *MedEdPORTAL*. 2023;19:11310.
https://doi.org/10.15766/mep_2374-8265.11310

Introduction

To provide high-quality patient care and improve patient outcomes, quality improvement (QI) training has been increasingly recognized as an essential component of resident medical education. Two of the six core competencies for resident training—as put forth by the Accreditation Council for Graduate Medical Education (ACGME)—are problem-based learning and improvement and system-based practice. Residents must demonstrate the ability to analyze the care they provide and play an active role in system improvement projects using QI methods.[1] In addition to the core competencies, the ACGME also lists internal medicine milestones for which residents are expected

Copyright © 2023 Xiang et al. This is an open-access publication distributed under the terms of the Creative Commons Attribution license.

to create, implement, and assess sustainable QI initiatives at the institutional level.[2]

Residency QI curricula reported in the literature exist in varying forms, with examples such as distinct QI rotations, asynchronous teaching via online modules, longitudinal experiential learning, chart audits, and participation in institutional QI initiatives.[3-7] Previous reviews have described characteristics of successful trainee QI curriculums, including learner buy-in, adequate teacher expertise and coaching, mixed teaching methods, inclusion of a QI project with adequate time for project completion, and a supportive institutional culture.[8,9] There currently is no consensus, however, on the most effective components of QI curricula, and few studies have included objective measurements of curricular success.[3] Conversely, challenges to engaging residents in QI that have been previously described in the literature include competing demands, didactics not connected with meaningful work, suboptimal and incomplete experiential learning, lack of clear accountability, lack of timely and relevant data, and lack of faculty coach and role model.[10] Our internal medicine residency program aimed to develop a longitudinal, experiential, and multilayer mentored QI curriculum that would incorporate the key components of successful QI curricula, address some of the barriers identified in the literature, and meet ACGME core competencies and resident milestones. Here, we present our curriculum, which was developed using QI process improvement principles, and demonstrate objective improvement in resident knowledge of QI principles and successful institutional changes.

Methods

Curriculum Overview

The QI curriculum was composed of a mandatory five-part longitudinal series presented in dedicated 3-hour educational sessions during ambulatory education blocks. Our internal medicine residency was divided into four large cohorts rotating through a dedicated 2-week ambulatory clinic every 8 weeks. Residents participated during their second year of residency, and each session was taught to 10-12 second-year residents in each block. Since 2016, the curriculum has been led by the internal medicine chief resident for quality and safety (CRQS) and overseen by their lead QI faculty mentor. The CRQS was an annual chief residency position sponsored by the Department of Veterans Affairs National Center for Patient Safety (NCPS). Each chief resident had previously completed the residency QI curriculum and the Institute of Healthcare Improvement (IHI) Basic Certification in Quality and Safety.[11] The chief resident also concurrently participated during their chief year in a national QI and patient safety curriculum, which was a longitudinal and experiential experience organized by the NCPS CRQS program.

Each of the five QI sessions had specific objectives developed after the IHI model for improvement[12] and incorporated ACGME core competencies and milestones. Residents applied the QI tools they had learned by working on a concurrent longitudinal QI project with dedicated time during these sessions. Each of the five sessions is described below.

Session 1—Introduction to Basic QI Concepts: Residents were given an overview of the QI curriculum and reasons for learning QI. To generate interest in QI training, residents were asked prior to the session to provide examples of challenging aspects of clinical care, referred to as pain points. During the session, the CRQS discussed how QI tools could be applied to address some of these pain points. Using a real-world internal medicine patient care vignette, residents learned common QI tools, including how to define a problem from a QI perspective by identifying key stakeholders, determining root causes through fishbone diagrams and the 5 Whys tool, and creating a problem statement based on their investigations (Appendices A and B).

Session 2—Applying QI Concepts to Resident-Initiated QI Projects: Residents learned how to write SMART (specific, measurable, attainable, relevant, and time-bound) aim statements and brainstormed interventions using IHI change concepts, intervention action hierarchies, and impact-versus-effort grids (Appendix C). The brainstormed QI project ideas were conducted in parallel with the curriculum in groups of two to four residents. Residents were provided with guidance regarding a feasible QI project by discussing the role of personal and stakeholder interest, project scope, and mentorship. Using a dedicated QI workbook with defined prompts (Appendix D), each project group applied the QI tools learned in session 1 to its projects. The groups then presented their projects to their peers for feedback. Residents were expected to identify and meet with a faculty mentor prior to session 3.

Session 3—Data Collection and Interpretation: Residents learned the similarities and differences between clinical research and QI. The remainder of the session focused on data collection and analysis. This included creating outcome, process, and balancing measures and learning how to interpret run charts to identify nonrandom variation in data (Appendix E). Each project team had dedicated time to develop its data collection plan and define project-related measures for its projects using the QI workbook.

Session 4—Project Presentation: The project teams presented their work-in-progress projects to a panel of faculty QI experts

Copyright © 2023 Xiang et al. This is an open-access publication distributed under the terms of the Creative Commons Attribution license.

and clinical leaders for feedback. Components of this 10- to 15-minute presentation included project background, problem statement, process map, fishbone diagram, aim statement, intervention ideas, and a data collection plan. The faculty QI experts asked questions and provided feedback about the projects. Residents used a template (Appendix F) to standardize their project presentations to the QI experts and institutional leaders.

Session 5—Spreading Change: Residents learned how to implement change via PDSA (plan-do-study-act) cycles, change management, and sustainability planning. They were taught common pitfalls for QI projects and how to set up their projects for long-term success (Appendix G). They also completed their QI project charter and developed a road map for project planning for the following year when they would be expected to continue to work on their QI projects (Appendix H).

Multilayer QI Project Mentorship

All resident QI projects received threefold mentorship from a faculty mentor, the CRQS, and institutional QI and clinical leaders. Prior to the start of the QI curriculum annually, the CRQS recruited and developed a list of faculty mentors with prior QI training and/or ongoing institutional QI projects. Residents identified their project and faculty mentor either through this list or independently based on their area of interest. The CQRS provided faculty mentors with information about mentorship expectations and project milestones (Appendix I). The CRQS also checked in with project groups every clinic block and provided added longitudinal mentorship via their QI expertise, including guidance regarding appropriate and feasible project scopes within the time constraints of residents' other clinical responsibilities. Residents presented their work-in-progress projects to local QI experts and clinical leaders for added feedback, organizational knowledge, and institutional support.

Resources

People and responsibilities: The CRQS and their lead QI faculty mentor met every other week for 30 minutes to plan and review the curriculum. The CRQS spent an additional 10-15 hours monthly to develop and coordinate the curriculum and mentor resident projects. Faculty mentors were expected to meet with their resident group at least once every 8 weeks to establish appropriate project scope, investigate the root causes of their project problem, and develop high-impact interventions. Faculty QI experts were additional faculty at our institution with QI expertise and/or leadership roles. They were invited to attend the work-in-progress sessions (four total for all ambulatory blocks) ad hoc. A faculty guide (Appendix J) offered teaching objectives, team goals, and timelines for each session. The QI curriculum was dynamic and underwent real-time and annual changes based on our trainees' educational needs, feedback from trainees and faculty, and results from validated QI skill assessment surveys.

Materials: Materials for the sessions included the following:

- PowerPoint presentations for each session.
- Resident QI workbook.
- Faculty mentor expectations and milestones.
- Potential faculty mentors and project ideas.
- Cloud-based sharing technology so that resident teams and the CRQS could interface and review dedicated project workbooks on an ongoing basis.

Space: Before the COVID-19 pandemic, the sessions required a conference room with projector for ambulatory sessions. During and after the COVID-19 pandemic, sessions were conducted as virtual meetings on Zoom.

Data Collection and Analysis

Two resident cohorts completed the validated Quality Improvement Knowledge Application Tool Revised (QIKAT-R) prior to and at the completion of the five-part education series for the 2020-2022 academic years. A publicly available tool assessing the global application of core QI skills, the QIKAT-R had been previously used in this pre-post testing format.[13] The QIKAT-R was graded on a scale of 0 (poor) to 9 (excellent). The CRQS distributed three unique QIKAT-R scenarios to each resident both before and after the curriculum. The CRQS was masked to the resident who completed each QIKAT-R but not to pre-post assessment. One additional investigator also scored the QIKAT-R and was masked both to pre-post intervention and to the resident. For data analysis, pre- and postcurriculum QIKAT-R results were analyzed via unpaired *t* tests using GraphPad Prism 8. In the 2020-2022 academic years, residents were also offered optional surveys (Appendix K) at the end of their QI sessions to seek their overall impression and feedback. Resident projects were collated based on themes such as guideline concordance, communication, wellness, electronic medical record documentation, and more.

Results

The CQRS position was introduced to our residency program in 2016, and the curriculum was developed and piloted during the 2016-2017 academic year. Using feedback from residents and faculty, the curriculum underwent iterative revisions. The full curriculum and workbook were presented to the first cohort of second-year residents starting in the 2020-2021 academic

Copyright © 2023 Xiang et al. This is an open-access publication distributed under the terms of the Creative Commons Attribution license.

year. Since 2016, a total of 234 internal medicine residents have completed our QI curriculum and developed 67 QI projects. Project themes have included improving transitions of care, reducing resident burnout, standardizing documentation, improving interdisciplinary communication, increasing age-appropriate cancer screening, and more. Our trainees have presented their QI work at various local, regional, and national venues. Prior to the COVID-19 pandemic, all 11 of the QI projects in the 2018-2019 academic year were presented at the institution's annual Graduate Medical Education QI conference, three projects were presented at regional or national conferences, and one project was published as a journal article. Since the COVID-19 pandemic, nine QI projects have been presented at the institution's conferences and two projects at regional or national conferences. In addition, several QI projects have been adapted at a systems level, including usage of fecal immunochemical testing for colorectal cancer screening at one of the resident clinic sites and a process for designating surrogate decision markers on inpatient medicine services, the latter of which received an award for best abstract at our institutional quality conference.

After initiation of the full curriculum in the 2020-2021 academic year, 59 residents (71%) completed the QIKAT-R precurriculum, and 65 residents (78%) completed the evaluation postcurriculum. The mean QIKAT-R scores were 4.56 prior to adoption of the curriculum and 6.48 postcurriculum ($p < .001$).

Two resident cohorts of 83 total residents from the 2020-2022 academic years were also asked to rate the quality and effectiveness of the curriculum. Using a 5-point Likert scale (1 = *poor,* 5 = *excellent*), residents who completed anonymous surveys rated their satisfaction with the QI curriculum as 4.10 ($n = 61$, 73% response rate). On another 5-point Likert scale (1 = *strongly disagree,* 5 = *strongly agree*), self-assessed understanding of writing an effective problem statement was rated 4.73 ($n = 15$, 18% response rate), writing an effective aim statement was rated 4.47 ($n = 17$, 20% response rate), stakeholder identification was rated 4.67 ($n = 15$, 18% response rate), creating fishbone diagrams was rated 4.07 ($n = 42$, 51% response rate), creating process maps was rated 4.29 ($n = 42$, 51% response rate), and identifying interventions was rated 4.47 ($n = 17$, 20% response rate).

Discussion

We developed and implemented a longitudinal, experiential, and mentored QI curriculum that was offered during dedicated ambulatory educational sessions for internal medicine residents. Our QIKAT-R results, resident feedback, and project output demonstrate that the curriculum led to improved trainee QI knowledge and systems-level improvements.

Our curriculum contains many of the mechanisms associated with successful QI curricula and addresses many of the challenges to resident engagement identified in the previous literature, along with additional features adding to the richness of the curriculum and the resident experience.[10,14] The curricular strategy is a hybrid teaching model using just-in-time learning through combining didactic lectures on key QI concepts with experiential, hands-on learning to reinforce important QI principles. Our curriculum covers topics ranging from continuous process improvement to process mapping and change management; a prior study showed that only a quarter to a half of resident curricula contain these topics.[9] Our curriculum is also highly structured, with clear learning objectives for each session and project deliverables provided through the QI workbook, work-in-progress presentations, and QI charters. Given competing demands with clinical workload, residents have protected time during embedded ambulatory education sessions for project work and operate in teams to facilitate project progress and completion. Furthermore, adult learning theories show that individuals learn best when they need to acquire the knowledge and skills for goal fulfillment.[3,15] Therefore, we use real-world internal medicine cases and resident-generated examples of clinical challenges and pain points to increase resident buy-in and interest in QI. Moreover, during our curriculum, residents are given the opportunity to develop their own projects based on their clinical interests.

Mentorship and bidirectional alignment of institutional projects and learner-selected QI projects are often cited as keys to successful QI curricula, but their impact has not been objectively measured.[9] We believe the structured multilayer mentorship from faculty and the CRQS within our curriculum is a critical component for successful resident QI projects. We have found that proactively recruiting and providing a list of faculty mentors with ongoing institutional QI projects and interest in trainee participation eases the identification of an appropriate mentor and aligns resident work with institutional priorities. Faculty and CRQS mentorship also result in appropriate project scoping and more effective interventions due to their prior clinical and research experiences and institutional knowledge. Stakeholder and institutional support for resident-initiated projects also eases implementation of project interventions. Therefore, faculty mentorship aids in removing or minimizing barriers to QI project work that would otherwise lead to resident frustration and disengagement with QI if left unaddressed. The CRQS's

Copyright © 2023 Xiang et al. This is an open-access publication distributed under the terms of the Creative Commons Attribution license.

providing faculty mentors with milestones sets clear expectations (i.e., outcome and process measures) and facilitates project navigation. The residents receive additional feedback from other faculty QI experts and clinical leaders during the work-in-progress session, which helps to generate institutional interest and support for resident projects and demonstrates to the residents the importance of QI within our department. This added layer of mentorship and feedback by local QI experts and clinical leaders is unique to our curriculum and, to our knowledge, has not been previously described in the literature. We also feel that inclusion and leadership of the CRQS—who is also receiving mentorship and gaining additional higher-level QI skills—are critical components. Prior to implementation of this position in 2016, only a handful of residents had successfully completed a successful QI project, with most working individually with a faculty member.

The limitations of our curriculum include the lack of a dedicated staff member to assist with access and retrieval of institutional data, which has been further exacerbated during the COVID-19 pandemic due to shifts in institutional priorities. We have addressed several of these delays through novel electronic health record education sessions that teach Epic self-reporting tools for timely data access and retrieval given Epic's use at our institution.[16] Our program's analysis and objective success were also affected by a loss of dedicated ambulatory time for QI education during the COVID-19 pandemic. The pandemic resulted in shifts in staffing requirements at our institution, which were reflected in decreased scholarly output due to competing clinical responsibilities. Additionally, while we appreciate the importance of interdisciplinary engagement for successful QI projects, it remains challenging to coordinate this during ambulatory education sessions given competing clinical and educational demands. Rather, residents are encouraged to reach out to interdisciplinary stakeholders and meet outside of dedicated time when needed. Furthermore, the anonymous resident surveys had variable response rates and therefore may not fully capture resident understanding of key QI concepts. However, most residents had a favorable view of the QI curriculum, and approximately three-quarters of trainees completed the previously validated QIKAT-R with a significant increase in their scores postcurriculum. The cases provided in our curriculum are built on real-world experiences at our institution, which helps with resident engagement for our program but may not necessarily be applicable to other centers. Therefore, in our faculty guide (Appendix J), we have provided examples of how our cases can be updated for other specialties for increased engagement.

Despite these challenges, we believe our curriculum is a comprehensive introduction to QI for residents. Through our longitudinal curriculum and with faculty and institutional support, our residents apply core QI concepts taught in didactics to their own QI projects and thereby implement meaningful change at our institution. Our curriculum adds a new dimension to the existing published QI educational content by introducing multiple avenues for QI mentorship, creating flexible time for project work, developing a process for resident accountability and mentor guidelines, incorporating flexible and standardized project work tools, and generating institutional support for residents to disseminate their QI work at various local, regional, and national meetings. We believe our curriculum fulfills the ACGME core competencies and can serve as a model for other clinical training programs.

Appendices

A. Session 1 Slides.pptx
B. Session 1 Workbook.pptx
C. Session 2 Slides.pptx
D. Session 2 Workbook.pptx
E. Session 3 Slides.pptx
F. Session 4 Work-in-Progress Presentation Template.pptx
G. Session 5 Slides.pptx
H. QI Charter Template.docx
I. Faculty Milestones.docx
J. Faculty Guide.docx
K. Resident Survey.docx

All appendices are peer reviewed as integral parts of the Original Publication.

Jenny Xiang, MD: Chief Resident, Department of Medicine, Yale School of Medicine and VA Connecticut Healthcare System; ORCID: https://orcid.org/0000-0003-4390-7641

Samantha Magier, MD, ME: Chief Resident, Department of Medicine, Yale School of Medicine and VA Connecticut Healthcare System

Jadry Gruen, MD: Cardiology Fellow, Division of Cardiovascular Medicine, Penn Medicine

Megan Welch, MD: Cardiology Fellow, Cardiology Division, Massachusetts General Hospital

Victor Bilan, MD: Hematology/Oncology Fellow, Department of Medical Oncology, Sidney Kimmel Medical College at Thomas Jefferson University

Copyright © 2023 Xiang et al. This is an open-access publication distributed under the terms of the Creative Commons Attribution license.

Benjamin Rodwin, MD: Assistant Professor, Department of Medicine, Yale School of Medicine and VA Connecticut Healthcare System

Alexandra Norcott, MD: Clinical Instructor, Division of Geriatric and Palliative Medicine, Department of Internal Medicine, University of Michigan Medical School

Naseema Merchant, MBBS: Assistant Professor, Department of Medicine, Yale School of Medicine and VA Connecticut Healthcare System

Disclosures
None to report.

Funding/Support
None to report.

Prior Presentations
Xiang JJ, Gruen J, Merchant NB. Engaging residents in quality improvement (QI) through a longitudinal, mentored, and experiential learning based QI curriculum. *J Gen Intern Med.* 2022;37(suppl 2):S623. Society of General Internal Medicine abstract 3709668.

Ethical Approval
Reported as not applicable.

References

1. Accreditation Council for Graduate Medical Education. *ACGME Common Program Requirements (Residency)*. Accreditation Council for Graduate Medical Education; 2020.
2. Accreditation Council for Graduate Medical Education. *Internal Medicine Milestones*. Accreditation Council for Graduate Medical Education; 2020.
3. Jones AC, Shipman SA, Ogrinc G. Key characteristics of successful quality improvement curricula in physician education: a realist review. *BMJ Qual Saf*. 2015;24(1):77-88. https://doi.org/10.1136/bmjqs-2014-002846
4. Fok MC, Wong RY. Impact of a competency based curriculum on quality improvement among internal medicine residents. *BMC Med Educ*. 2014;14:252. https://doi.org/10.1186/s12909-014-0252-7
5. Dulay M, Saxe JM, Odden K, et al. Promoting quality improvement in primary care through a longitudinal, project-based, interprofessional curriculum. *MedEdPORTAL*. 2020;16:10932. https://doi.org/10.15766/mep_2374-8265.10932
6. Tapper E, Sullivan A, Tess A. Teaching quality improvement on the wards: how we do it. *MedEdPORTAL*. 2015;11:10211. https://doi.org/10.15766/mep_2374-8265.10211
7. Werner JA. An integrated, multimodal resident curriculum in patient safety and quality improvement. *MedEdPORTAL*. 2017;13:10641. https://doi.org/10.15766/mep_2374-8265.10641
8. Starr SR, Kautz JM, Sorita A, et al. Quality improvement education for health professionals: a systematic review. *Am J Med Qual*. 2016;31(3):209-216. https://doi.org/10.1177/1062860614566445
9. Wong BM, Etchells EE, Kuper A, Levinson W, Shojania KG. Teaching quality improvement and patient safety to trainees: a systematic review. *Acad Med*. 2010;85(9):1425-1439. https://doi.org/10.1097/ACM.0b013e3181e2d0c6
10. Koller JP, Cochran KA, Headrick LA. Practical strategies to enhance resident engagement in clinical quality improvement. *BMC Med Educ*. 2022;22:96. https://doi.org/10.1186/s12909-022-03134-y
11. IHI Open School online courses curriculum. Institute for Healthcare Improvement. Accessed January 26, 2023. https://www.ihi.org/education/ihi-open-school/Pages/Curriculum.aspx
12. How to improve. Institute for Healthcare Improvement. Accessed January 26, 2023. https://www.ihi.org/resources/Pages/HowtoImprove/default.aspx
13. Singh MK, Ogrinc G, Cox KR, et al. The Quality Improvement Knowledge Application Tool Revised (QIKAT-R). *Acad Med*. 2014;89(10):1386-1391. https://doi.org/10.1097/ACM.0000000000000456
14. Brown A, Lafreniere K, Freedman D, et al. A realist synthesis of quality improvement curricula in undergraduate and postgraduate medical education: what works, for whom, and in what contexts? *BMJ Qual Saf*. 2021;30(4):337-352. https://doi.org/10.1136/bmjqs-2020-010887
15. Smith SR, Bakshi R. Promoting resident involvement in quality improvement initiatives through faculty involvement and curriculum. *J Grad Med Educ*. 2015;7(1):119-120. https://doi.org/10.4300/JGME-D-14-00508.1
16. Xiang JJ, Park J, McCann J, et al. Teaching practice-based learning and improvement and digital health through Epic self-reporting tools training. *J Gen Intern Med.* 2022;37(suppl 2):S655. Society of General Internal Medicine abstract 3709617.

Received: August 17, 2022
Accepted: January 8, 2023
Published: April 18, 2023

Copyright © 2023 Xiang et al. This is an open-access publication distributed under the terms of the Creative Commons Attribution license.
